# Which functional tasks present the largest deficits for patients with total hip arthroplasty before and six months after surgery? A study of the timed up-and-go test phases

**DOI:** 10.1371/journal.pone.0255037

**Published:** 2021-09-10

**Authors:** Xavier Gasparutto, Mathieu Gueugnon, Davy Laroche, Pierre Martz, Didier Hannouche, Stéphane Armand

**Affiliations:** 1 Kinesiology Laboratory, Geneva University Hospitals and University of Geneva, Geneva, Switzerland; 2 INSERM, CIC 1432, Centre for Clinical Investigation, Module Plurithématique, Plateforme d’Investigation Technologique, University Hospital of Dijon-Burgundy, Dijon, France; 3 INSERM, UMR 1093-CAPS, Faculty of Sports, University of Burgundy, Dijon, France; 4 Division of Orthopaedic Surgery and Musculoskeletal Trauma Care, University Hospital of Dijon-Burgundy, Dijon, France; 5 Division of Orthopaedic Surgery and Musculoskeletal Trauma Care, Surgery Department, Geneva University Hospitals and University of Geneva, Geneva, Switzerland; University of Innsbruck, AUSTRIA

## Abstract

Six to eight months after total hip arthroplasty, patients only attain 80% of the functional level of control groups. Understanding which functional tasks are most affected could help reduce this deficit by guiding rehabilitation towards them. The timed up-and-go test bundles multiple tasks together in one test and is a good indicator of a patient’s overall level of function. Previously, biomechanical analysis of its phases was used to identify specific functional deficits in pathological populations. To the best of our knowledge, this analysis has never been performed in patients who have undergone total hip arthroplasty. Seventy-one total hip arthroplasty patients performed an instrumented timed up-and-go test in a gait laboratory before and six months after surgery; fifty-two controls performed it only once. Biomechanical features were selected to analyse the test’s four phases (sit-to-stand, walking, turning, turn-to-sit) and mean differences between groups were evaluated for each phase. On average, six months after surgery, patients’ overall test time rose to 80% of the mean of the control group. The walking phase was revealed as the main deficiency before and after surgery (-41 ± 47% and -22 ± 32% slower, respectively). High standard deviations indicated that variability between patients was high. On average, patients showed improved results in every phase of the timed up-and-go test six months after surgery, but residual deficits in function differed between those phases. This simple test could be appropriate for quantifying patient-specific deficits in function and hence guiding and monitoring post-operative rehabilitation in clinical settings.

## Introduction

Total hip arthroplasty (THA) is a common, cost-effective and usually highly successful surgical procedure to alleviate pain and improve motor function [1] (‘motor function’ will be referred to as ‘function’ throughout this paper to improve readability). Although THA improves function for patients with end-stage hip osteoarthritis (OA), patients have not fully recovered function six months and one year after surgery when compared to healthy control groups [2, 3]. On average, patient function increases from 70% of the level of healthy control groups before surgery to 80% six to eight months afterwards [2]. Understanding which domains of function (e.g. strength, balance or mobility) are most improved by THA and which retain the most significant post-surgical deficits could help to map this residual loss of function and guide patient-specific rehabilitation.

Several types of evaluation of function exist. Each covers different domains of functioning as defined by the World Health Organization’s International Classification of Functioning and Disability (WHO ICF) [4]. The most common tools are: (i) patient-reported outcomes (PROMs) [5] (e.g. the Hip disability and Osteoarthritis Outcome Score [6]) that evaluate multiple domains of function through self-administered questionnaires (e.g. personal factors, activities, participation); (ii) clinical evaluations that evaluate body function (e.g. hip range of motion); and, (iii) medical imaging that evaluates body structure (e.g. femoral offset). Clinical tests like the 6-minute walk test, the 30-second chair stand test or the Timed Up-and-Go (TUG) test—often described as *performance-based tests* [7]—are used to evaluate patients’ capacity as defined by the WHO ICF, i.e. *what a person can do in a standardized evaluation setting* [4].

The TUG test is a robust indicator of a patient’s overall level of motor function [8] as it groups together basic mobility skills needed for activities of daily living in a simple, practical test [9]. Participants start sitting in an armchair, stand up, walk three meters, turn back and return to sit in the armchair [9]. The test is recommended by the Osteoarthritis Research Society International [10] for assessing physical function in people diagnosed with hip OA, and it is a noted test of ambulatory activity covering the specific ICF codes of “changing basic body position” (d410), “walking” (d450) and “moving around” (d455), and of strength, agility and dynamic balance [10]. It is widely used to assess mobility in rehabilitation [11].

In the context of THA, pre-surgery TUG test results have been identified as predictive of post-surgery hospital length of stay [12], predictive of ambulatory status six months after a primary THA [13] and associated with a risk of post-surgery deep vein thrombosis [14]. However, as underlined by previous authors [15–17], using the TUG’s total time as the sole outcome provides limited information. Indeed, the analysis of the TUG’s phase, known as instrumented TUG (iTUG) [18], could provide a deeper understanding of a patient’s function before and after treatment. For instance, the iTUG test was shown capable of discriminating between patients with early-to-moderate Parkinson’s disease and healthy controls, whereas the total TUG time could not [18, 19]. The iTUG test also showed that slower total TUG times in a group of obese women compared to a control group were explained by the walking and turning phases, not by the sit-to-stand and stand-to-sit phases [17]. To the best of our knowledge, no studies of the TUG test’s different phases have been performed previously on patients who have end-stage OA or have undergone a THA. A study to identify the most difficult tasks facing patients before and after surgery could help to target patient-specific rehabilitation.

The present study’s objectives were: 1) to assess in which phases of the TUG test patients presented with significant functional deficits compared to a control group, before and after surgery; 2) to assess how these phases changed after surgery; and 3) to compare changes and deficits in total TUG time with changes and deficits in the functional outcomes of the different phases. To this end, we performed a biomechanical analysis of the TUG test for a group of patients before and six months after surgery, and their outcomes were compared with those of a control group.

## Material & methods

### Participants

This study included 71 THA patients and 52 controls. Participants’ characteristics are reported in Table 1. Surgeries were performed by two surgeons using three different approaches: a mini-invasive Rottinger approach (n = 38) [20], a mini-posterior approach (n = 29) [21] or a lateral approach (n = 4), with a dual-mobility cup implanted in all cases. The local ethics committee approved the study (Human Welfare Protection Committee EST I, in Dijon), and it was recorded as a clinical trial (NCT02042586), with all participants giving their written informed consent.

**Table 1:** Participants’ characteristics.

	Patients	Control Group	*p*-value
**General Characteristics**			
Sex (M/F)	29/42	18/34	0.607
Age (years)	68.5 (8.9)	65.3 (8.6)	0.054
Height (cm)	162.8 (8.0)	163.4 (8.3)	0.703
Weight (kg)	75.6 (17.7)	66.9 (13.0)	0.001
OA Side (L/R)	(32/39)	-	
**Kellgren and Lawrence classification**			
II	14	-	
III	31	-	
IV	21	-	
NA	5	-	
**Surgical Approach**			
Rottinger	38	-	
Mini-posterior	29	-	
Lateral	4	-	

Sex distribution was tested using the χ^2^ test, and differences in age, height and weight used Student’s t-tests.

NA = not available.

All the patients included in this study were diagnosed with primary hip OA, according to the American College of Rheumatology (ACR) criteria [22], and had stage II, III or IV hip OA according to the Kellgren and Lawrence classification. Mean patient age was 68.5 years old (SD = 8.9 years, range = 46–85 years), with 42 women and 29 men. All had undergone conservative treatment for at least three months before they were considered for a hip replacement, and all were able to understand simple instructions. Exclusion criteria included being younger than 25 years old or older than 85, previous hip surgery, bilateral hip OA, concomitant knee OA, pregnancy or breast-feeding, inflammatory disorders, rapidly progressive hip OA, hip dysplasia, neurological disease, motor neuron disease and all disorders that might interfere with or be worsened by a gait analysis. A control group of participants in the same age range was collected. Their exclusion criteria included OA in the lower limbs, neurological disease, motor neuron disease or disorders that might interfere with or be worsened by a gait analysis.

### Measurement protocol

Participants were equipped with 35 reflective skin markers attached over their whole body according to the Conventional Gait Model [23]. Markers’ trajectories were measured at 100 Hz using an eight-camera optoelectronic system (MXT 40, Vicon, Oxford, UK), filtered at 6 Hz using a fourth-order Butterworth design and then occlusions were corrected using marker intercorrelations [24].

**Fig 1:**
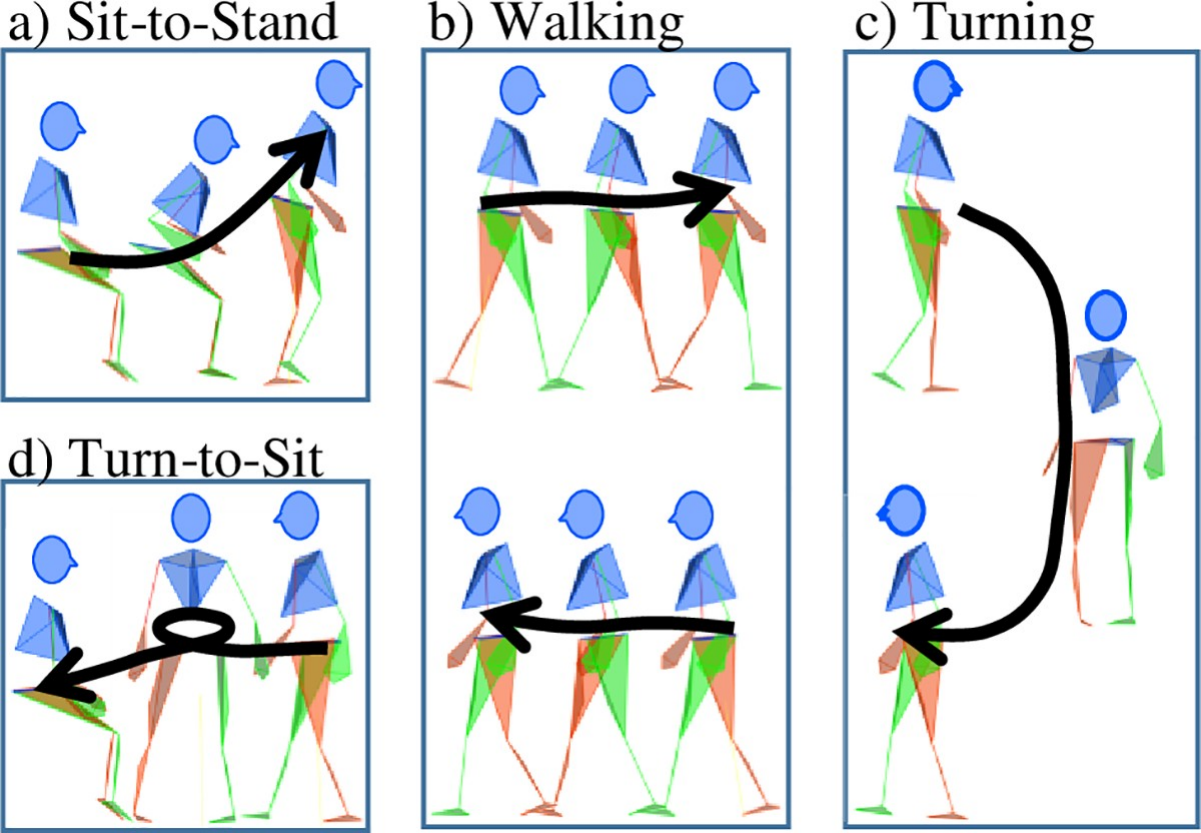
The four phases of the timed-up and go test.

Participants were asked to perform the TUG test. They sat in an armchair with a seat at 47 cm off the ground, stood up, walked to a line on the ground three meters away, turned around and came back to sit in the chair [9] (Fig 1). The whole task was made at a self-selected speed, and participants were allowed to use the armrests. Instructions were as follows: “At the start signal, stand up from the chair without using the armrests, if possible, then walk towards the line in front of you, turn back at the line and come back to the chair and sit down, without using the armrests, if possible.” Participants performed ten TUG tests, and the fastest of the first three tests was retained for analysis. When sitting, the participant’s back could not touch the armchair’s backrest because of the motion capture equipment. Patients performed the test before surgery (M0) and six months after surgery (M6), whereas the control group performed the test only once.

### Analysis

The TUG test was divided into four phases (Fig 1): sit-to-stand, walking (back and forth), turn and turn-to-sit [15]. The second turn and stand-to-sit phases were grouped together as a turn-to-sit phase due to the difficulty in discriminating between the end of the turn and the start of the sitting phase [15]. The beginning and end of the TUG test phases were identified using a custom algorithm based on an existing algorithm [25] (S1 File). Work by Beyea et al. [25] was adapted to identify the start and end of turns and the end of the TUG test. Indeed, when checked visually, Beyea’s algorithm identified timepoints outside of the phases for those events with the study population. The visual checks of the algorithm’s results were assessed using Mokka software [26] and specific plots (S1 and S2 Figs).

The time required to complete each phase and an appropriate set of each phase’s biomechanical features were selected for analysis. Feature were selected based on the literature [18, 19, 27–35], and grouped into two categories: 1) quality of movement (joint angles and distances) [36] and 2) speed (linear and angular velocities). We selected a total of 38 features covering the four phases (Table 2) in addition to the total TUG test time and the time for each phase. The reasons for choosing these features and their definitions are detailed in S2 File.

**Table 2:** Biomechanical features for each phase.

	Quality	Speed
**Sit-to-Stand**	Peak obliquity thorax	Peak vertical velocity thorax
	Range obliquity thorax	Peak vertical velocity pelvis
	Peak flexion thorax	Peak extension velocity of pathological hip
	Width base of support	Peak extension velocity of contralateral hip
	Length base of support	
**Walking**	RMS obliquity thorax	Peak forward velocity thorax
	Range obliquity thorax	Mean forward velocity thorax
	Lateral RMS C7	Peak forward velocity pelvis
	Lateral range C7	Mean forward velocity pelvis
	Range of flex/ext pathological hip	
	Range of flex/ext contralateral hip	
**Turn**	Number of steps	Peak angular velocity thorax
	Side of turn	Mean angular velocity thorax
		Peak angular velocity pelvis
		Mean angular velocity pelvis
**Turn-to-sit**	Peak obliquity thorax	Peak vertical velocity thorax
	Range obliquity thorax	Peak vertical velocity pelvis
	Peak flexion thorax	Peak extension velocity of pathological hip
	Distance chair to start of turn	Peak extension velocity of contralateral hip
	Number of steps	Peak angular velocity thorax
		Mean angular velocity thorax
		Peak angular velocity pelvis
		Mean angular velocity pelvis

RMS = root mean square; flex/ext = flexion/extension.

### Statistics

The difference in sex distribution was tested using a **χ**^**2**^ test. The Kolmogorov–Smirnoff [37] test was used to assess the normality of the age, weight and height distributions among patients and controls. Differences between groups were then tested using unpaired Student’s t-tests.

A principal component analysis (PCA) was performed so that only the most relevant biomechanical features were retained for analysis (excluding phase completion times). To account for scale differences, features were centred and reduced by subtracting the mean and dividing by the standard deviation (SD). All three test groups (patients at M0, patients at M6 and the controls) were included in the analysis. The number of principal components was chosen using Cattell’s scree test [38]. One feature was selected per principal component and per phase based on the maximal cos2 value and if this value was above 0.20.

The statistical differences between groups for the selected features were assessed using a paired-sample Student’s t-test when comparing the patient groups and using an unpaired Student’s t-test when comparing patient and control groups. The Holm method was used to assess the level of statistical significance (α = 0.05) [39], and Cohen’s d was used to assess the effect size. To evaluate the average difference in patient function from control group function, the values of the features at M0 and M6 were reported as percentages of the control group mean. Lower function among patients than among the control group was described as a deficit. Depending on the feature, lower function could be a higher value (e.g. phase duration) or a lower value (e.g. peak vertical velocity of the thorax for the sit-to-stand phase). Changes between M0 and M6 were expressed as percentages of a feature’s value at M0 to express post-surgery increases or decreases in function. Furthermore, the percentage of patients with a similar level of function to the control group (i.e. within 1 SD of the control group’s mean) was also evaluated, as was the percentage of patients whose function increased between M0 and M6. Pearson correlations were calculated between the total TUG time and selected features for percentage differences to the control group and the percentage of change between M0 and M6. The level of significance was adjusted using the Holm method (α = 0.05) [39]. Only significant correlations were reported.

Finally, the percentages of each feature selected using PCA were averaged for each phase to give one overall figure per phase. Paired Student’s t-tests were performed to assess whether the average differences compared to the control group, or between M0 and M6, differed significantly between the TUG test’s different phases. To account for multiple testing, the Holm’s method was used to adjust the *p*-values (α = 0.05) [39].

## Results

The patient and control groups did not differ significantly in sex, age or height but differed significantly in weight (Table 1).

### Principal component analysis

The complete results of the PCA are presented in S1 Table. Cattell’s scree test selected three principal components, representing 56.4% of the total variance. The first principal component (39.1% of variance) was mainly associated with speed parameters, and the second (10.0% of variance) and third (7.3% of variance) with quality parameters.

**Fig 2:**
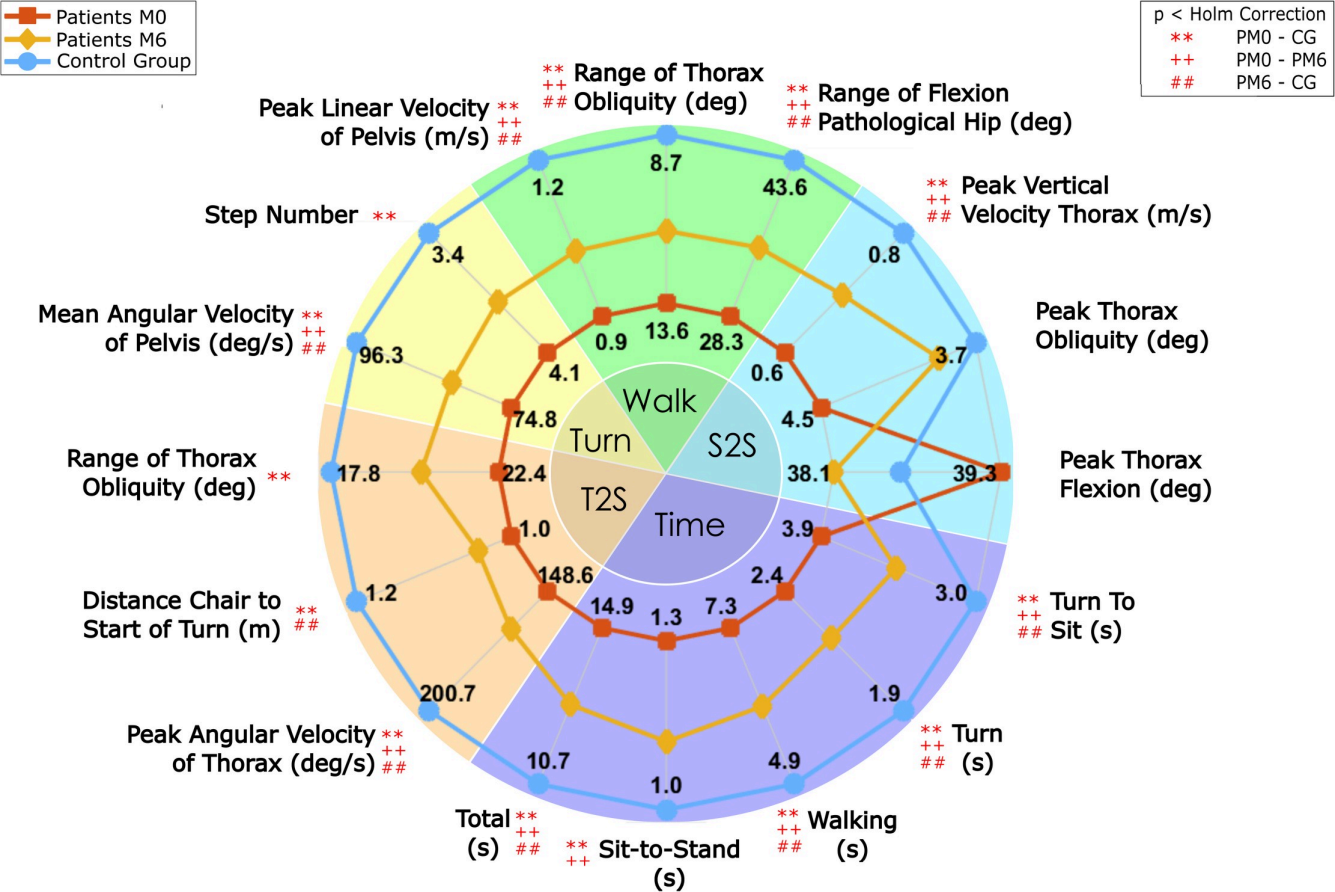
Overview of each phase’s duration and the features selected using PCA for all three groups: Patients before surgery (PM0), patients six months after surgery (PM6) and Control Group (CG). Feature values representing higher levels of function are on the outer edge, and lower levels of function are on the inner edge. S2S = Sit-to-Stand; T2S = turn-to-sit.

The biomechanical features selected for each phase are presented in Table 3, and the average scores for those features across the three groups are reported in Fig 2 and Table 4. Each patient’s values are compared to the control group’s level in S3 Fig.

**Table 3:** Features selected using principal component analysis.

	Category	Feature	PC	Cos2
**Sit-to-Stand**	Quality	Peak flexion thorax	2	0.45
		Peak obliquity thorax obliquity	3	0.28
	Speed	Peak vertical velocity thorax	1	0.61
**Walking**	Quality	Range of flex/ext pathological hip	1	0.50
		Range obliquity thorax	3	0.30
	Speed	Peak forward velocity pelvis	1	0.86
**Turning**	Quality	Step number	1	0.21
	Speed	Mean angular velocity pelvis	1	0.66
**Turn-to-Sit**	Quality	Range obliquity thorax	2	0.66
		Distance chair to start of turn	1	0.23
	Speed	Peak angular velocity thorax	1	0.76

PC is the principal component associated with the feature; cos2 (є [0,1]) shows the importance of the feature considered on the associated principal component.

**Table 4:** Mean values (standard deviation) for patients before surgery (M0), patients six months after surgery (M6) and the control group.

Feature Categories	Features	Patients M0			Patients M6			Control Group			Group Comparison	Cohen’s d		
		Mean	SD	95%CI	Mean	SD	95%CI	Mean	SD	95%CI		a	b	c
**Total**	** **													
Speed	Time (s)	14.9	4.1	13.9 to 15.8	12.9	2.8	12.2 to 13.5	10.7	2.1	10.2 to 11.3	a, b, c	1.2	0.7	0.8
**Sit-to- Stand**														
Quality	Peak thorax flexion (deg)	39.3	10.0	37.0 to 41.6	38.1	8.9	36.0 to 40.1	38.6	10.4	35.7 to 41.4	-	-	-	-
	Peak thorax obliquity (deg)	4.5	2.4	4.0 to 5.1	3.9	2.1	3.4 to 4.3	3.7	1.8	3.1 to 4.1	-	-	-	-
Speed	Peak vertical velocity thorax (m/s)	0.56	0.19	0.52 to 0.60	0.66	0.17	0.62 to 0.70	0.77	0.17	0.72 to 0.82	a, b, c	1.1	0.6	0.6
	Duration (s)	1.3	0.4	1.2 to 1.4	1.1	0.3	1.0 to 1.2	1.0	0.2	0.9 to 1.0	a, b	0.9	0.4	-
**Walk**														
Quality	Range of flex/ext pathological hip (deg)	28.3	7.8	26.5 to 30.2	35.0	7.1	33.4 to 36.7	43.6	5.1	42.2 to 45.0	a, b, c	2.2	0.9	1.3
	Range thorax obliquity (deg)	13.6	5.9	12.2 to 15.0	11.5	3.9	10.6 to 12.4	8.7	3.3	7.8 to 9.7	a, b, c	1.0	0.4	0.7
Speed	Peak forward velocity pelvis (m/s)	0.91	0.20	0.86 to 0.96	1.02	0.21	0.97 to 1.07	1.19	0.20	1.14 to 1.24	a, b, c	1.4	0.6	0.8
	Duration (s)	7.3	2.7	6.7 to 7.9	6.1	1.8	5.6 to 6.5	4.9	1.3	4.5 to 5.2	a, b, c	1.1	0.6	0.7
**Turn**														
Quality	Number of steps	4.1	0.9	3.9 to 4.3	3.9	0.9	3.6 to 4.1	3.4	0.9	3.2 to 3.7	a	0.8	-	-
Speed	Mean angular velocity pelvis (deg/s)	74.8	19.2	70.3 to 79.3	83.1	21.1	78.2 to 88.1	96.3	20.3	90.1 to 101.8	a, b, c	1.1	0.4	0.6
	Duration (s)	2.4	0.5	2.3 to 2.5	2.2	0.5	2.1 to 2.3	1.9	0.4	1.8 to 2.0	a, b, c	1.1	0.4	0.7
**Turn-to-Sit**														
Quality	Range thorax obliquity (deg)	22.3	9.0	20.3 to 24.5	20.3	7.8	18.4 to 22.1	17.8	6.8	16.0 to 19.7	a	0.6	-	-
	Distance chair to start turn (m)	1.03	0.20	0.98 to 1.08	1.06	0.17	1.02 to 1.10	1.17	0.16	1.13 to 1.21	a, c	0.8	-	0.7
Speed	Peak angular velocity thorax (deg/s)	148.6	37.6	139.9 to 157.4	164.9	41.9	155.2 to 174.7	200.7	37.2	190.6 to 210.8	a, b, c	1.4	0.6	0.9
	Duration (s)	3.9	1.1	3.7 to 4.2	3.5	0.8	3.3 to 3.7	3.0	0.6	2.8 to 3.2	a, b, c	1.0	0.5	0.6

Significant differences between groups, as per the Holm method, are reported between patients at M0 and the control group as “a”, between patients at M0 and patients at M6 as “b”, and between patients at M6 and the control group as “c”. Cohen’s d was reported for significant differences.

### Patients’ functional deficits compared to the control group

#### Deficits before surgery

The test phase with the largest mean deficit before surgery was the walking phase, at -41% (Table 5). Compared with the other phases, the walking phase deficit was statistically larger than the sit-to-stand, turning and turn-to-sit phases, as per the Holm method. The other phases did not differ significantly from each other. Only 8% of patients had a range of hip flexion–extension during walking similar to the control group’s level, whereas 31% to 69% of patients’ other features were at the control level (Table 6).

**Table 5:** Mean difference ± SD (95% confidence interval) between groups by phase expressed in percentages.

Tasks	Patients M0 vs CG			Patients M0 vs M6			Patients M6 vs CG		
	Mean	SD	95%CI	Mean	SD	95%CI	Mean	SD	95%CI
Sit-to-Stand (%)	-21	± 46	(-31 to -10)	8	± 48	(-4 to 19)	-9	± 37	(-17 to 0)
Walk (%)	-41	± 47	(-52 to -30)	16	± 35	(8 to 24)	-22	± 32	(-30 to -15)
Turn (%)	-24	± 25	(-29 to -18)	8	± 26	(2 to 14)	-14	± 24	(-20 to -8)
Turn-to-Sit (%)	-27	± 38	(-36 to -19)	8	± 26	(2 to 14)	-16	± 32	(-23 to -8)

**Table 6:** Mean, SD and 95% confidence interval of the significant differences between groups, i.e. differences between patient and control groups and between patients before and six months after surgery.

Feature Categories	Features	Patients M0 vs CG					Patients M6 vs M0					Patients M6 vs CG				
		Mean	SD	95%CI	% > CG level	R total time	Mean	SD	95%CI	%Δ >0	R total time	Mean	SD	95%CI	% > CG level	R total time
**Total**	** **															
Speed	Time (%)	-39	38	-48 to -30	34	-	11	17	7 to 15	72	-	-20	26	-26 to -14	56	-
**Sit to Stand**																
Quality	Peak thorax flexion (%)	2	26	-4 to 8	87	-	0	24	-6 to 6	38	-	-1	23	-7 to 4	86	-
	Peak thorax obliquity (%)	-24	65	-39 to -9	75	-	-5	74	-22 to 12	59	-	-6	57	-19 to 8	83	-
Speed	Peak vertical velocity thorax (%)	-27	25	-33 to -21	41	0.59	26	37	18 to 35	73	0.63	-14	22	-19 to -9	61	0.65
	Time (%)	-33	46	-44 to -22	46	0.71	8	33	1 to 16	70	0.57	-13	33	-21 to -6	68	0.62
**Walk**																
Quality	Range of flex/ext pathological hip (%)	-35	18	-39 to -31	8	0.37	31	41	22 to 41	82	-	-20	16	-23 to -16	27	0.40
	Range thorax obliquity (%)	-56	67	-71 to -40	49	-	5	42	-4 to 15	69	-	-32	45	-42 to -21	61	-
Speed	Peak forward velocity pelvis (%)	-24	17	-27 to -20	31	0.87	15	22	10 to 20	79	0.77	-14	18	-18 to -10	51	0.78
	Time (%)	-49	55	-62 to -36	39	0.95	11	26	5 to 17	73	0.83	-25	38	-33 to -16	62	0.92
**Turn**																
Quality	Number of steps (%)	-20	26	-26 to -14	69	-	4	28	-3 to 10	42	-	-12	27	-18 to -5	77	0.45
Speed	Mean angular velocity pelvis (%)	-22	20	-27 to -18	41	0.61	14	28	8 to 21	68	0.52	-14	22	-19 to -9	61	0.64
	Time (%)	-28	28	-35 to -21	41	0.68	6	23	1 to 11	66	0.49	-17	26	-23 to -11	58	0.68
**Turn to Sit**																
Quality	Range thorax obliquity (%)	-25	51	-37 to -14	69	-	3	31	-4 to 11	52	-	-14	44	-24 to -3	76	-
	Distance chair to start turn (%)	-12	17	-16 to -8	51	0.49	6	22	1 to 11	61	-	-9	15	-13 to -6	63	0.45
Speed	Peak angular velocity thorax (%)	-26	19	-30 to -22	31	0.73	13	23	8 to 18	72	0.67	-18	21	-23 to -13	46	0.78
	Time (%)	-31	37	-39 to -22	44	0.76	8	21	3 to 13	68	0.71	-16	27	-22 to -9	65	0.76

“% > CG” represents the number of patients with function similar to the control group’s level (within 1 SD of the mean) for that feature, and “% Δ > 0” represents the number of patients with an improvement in that feature’s function at M6. “R total time” represents the feature’s coefficient of correlation with total test time. Features with no significant differences between the groups considered are reported with light grey background.

#### Residual deficits six months after surgery

The largest mean residual deficit at M6 was for the walking phase (-22%, 95%CI: -30% to -15%, Table 5). Using the Holm method, this deficit was significantly greater than the sit-to-stand and turning phase deficits, but not greater than the turn-to-sit phase deficit. Only 27% of patients had a range of hip flexion–extension during walking similar to controls, whereas, for other features with significant differences, 46% to 65% of patients had a similar level of function to controls (Table 6).

### Change in function six months after surgery

The walking phase showed the greatest mean improvement in function (16%, 95%CI: 8% to 24%, Table 5), although this was not statistically significant. The sit-to-stand, turning and turn-to-sit phases showed similar increases (8%, Table 5).

### Total TUG times

Mean total TUG times were 14.9 ± 4.1 s and 12.9 ± 2.8 s for patients at M0 and M6, respectively, and 10.7 ± 2.1 s for the control group (Table 4). On average, patients were -39% (95%CI: -48% to -30%) slower than the control group before surgery, and they had improved their total time by 11% (95%CI: 7% to 15%) six months after surgery. However, they were still -20% (95%CI: -26% to -14%) slower than the control group (Table 6). These differences were all statistically significant. The majority of patients (72%) improved their total time between M0 and M6. At M0, 34% of patients had a functional level similar to the control group, rising to 56% at M6 (Table 6).

#### Associations between deficits and changes in features and deficits and changes in total TUG times (Table 6)

The deficits and changes in total TUG time were mainly associated with speed parameters, especially with the deficits and changes in the duration of the walking phase and with the peak forward velocity of the pelvis (Table 6). The deficits in the range of flexion–extension of the patient’s pathological hip during walking at M0 and M6, the deficit in the number of steps taken during the turning phase at M6, and the deficits in the distance from the chair to the start of the turn at M0 and M6 were the only quality parameters associated with the difference in total time that displayed moderate correlations (Table 6). The change in total TUG time between M0 and M6 was only associated with speed parameters. The highest correlations were with the change in the time of the walking phase and the change in peak walking speed.

## Discussion

The present study assessed the change in function among a group of patients with THA, before and six months after surgery, and the functional deficits in comparison to a control group by the means of an instrumented TUG test. The test was divided into four phases [15], representing different activities of daily living (sit-to-stand, walking, turning and turn-to-sit); multiple biomechanical features quantifying the motor functions were selected using PCA and, the features for each phase were compared between groups. Finally, mean pre- to post-surgery changes and deficits in features, phase times and phase functional outcomes—between patients at those times and compared with the control group—were assessed to evaluate links between them and deficits and changes in total TUG time.

### Patients’ functional deficits compared to the control group

Results suggested that the walking task represented the main limitation to patients with THA both before and six months after their surgery. The changes in walking revealed in this study were consistent with a recent literature review showing that six months post-surgery, gait speed and the pathological hip’s range of flexion–extension increased, although they remained lower than control group levels [3]. Our study’s results showed that the hip range of flexion–extension during walking seemed to be an appropriate feature of motor function with which to study the effects of THA. Indeed, this feature presented the lowest percentage of patients with a level of function similar to that of the control group, both before and after surgery (8% and 27%, respectively), as well as the greatest number of patients with a positive change in function after surgery (82%). This suggests that, despite their deficits six months after surgery, THA has a positive effect on patients’ sagittal hip kinematics during walking.

### Changes in function six months after surgery

There were no significant differences in the changes between the phases, although the walking phase showed the largest mean increase in function. Thus, THA seems to improve patients’ motor functions homogeneously across the different phases of the TUG test.

### Changes and deficits in total TUG time compared to changes and deficits in functional outcomes

The changes and deficits in total TUG time were mainly strongly to moderately correlated with the changes and deficits in the test’s speed features, but also with a few features representing the quality of movements (pathological hip ranges of motion at M0 and M6, number of steps during the turn at M6, and distances from the chair to the start of turn at M0 and M6). More specifically, the changes and deficits in total test time were strongly correlated with the changes and deficits in the duration of the walking phase, the TUG test’s longest phase. Total TUG test time could be a good indicator of speed parameters, but it does not seem to reflect comparably the quality of movements, such as the presence of compensatory movements (e.g. high range of thorax obliquity). Moreover, Caronni et al. [15] showed that an improvement in total TUG time was not associated with improvements in all the test’s phases. Thus, this feature’s utility seems limited in improving our understanding of a patient’s level of function across multiple domains.

Nevertheless, the residual deficit in total TUG time was 20% compared to the control group mean. This is consistent with the 20% residual deficit in function reported by Vissers et al. six to eight months post-surgery [2], and it supports a previous study suggesting that the total time of the TUG test could be a good indicator of the overall level of motor function [8].

### Outcome variability

Although THA patients’ function improved in every phase of the TUG test after their surgery, on average, they nevertheless presented with deficits in each one of those phases when compared to the control group before and six months after their surgery. This supports previous studies showing improvements in function but an incomplete recovery six months after surgery [2]. However, the SDs of the percentage differences between groups were very high, e.g. the SD of the deficits in total TUG time at M0 was 38% and the mean deficit was 39%. These high SDs suggest great variability in patient profiles. Indeed, at M6, some patients had similar levels of function to the control group, whereas others showed no improvements in function (S3 Fig). Understanding the preoperative factors at the origin of those differences in profile could help to identify the patients at risk of having a lower level of function after surgery and improve patient rehabilitation.

The high variability resulting from the analysis of the TUG test’s phases suggests that it could be used to identify patient-specific levels of function. Identifying a functional deficit in a particular phase might help target the functional domain in which the patient is most limited. For example, a patient with a large post-surgical deficit in the sit-to-stand phase could be oriented towards specific strength training to reduce this limitation [33]. Such tests could also be used to monitor patients’ rehabilitation processes post-surgery.

The PCA showed that the largest part of the variance between groups was associated with speed features. This seems to indicate that patients improve the speed of their movements more than they improve their quality at six months after surgery. One explanation could be that the reduction in post-surgery pain leads to improved hip function and thus to increases in speed, but that the compensations developed before surgery, to avoid pain in the arthritic hip, might take more than six months to correct. Nevertheless, the range of thorax obliquity was significantly lower after surgery for the test’s walking and turn-to-sit phases, which indicates a decrease in compensatory movements during walking and less asymmetry during the sitting phase.

Surprisingly, the direction of the turn was not a relevant parameter for this pathology. Indeed, at M0 and M6, half the patients turned towards their pathological side and the other half towards their non-pathological side.

### Implementation in clinical settings

The TUG test could be performed with a simpler, low-cost setup by using Inertial Measurement Units (IMUs), as previously done with other pathological populations [18]. Only three IMUs placed on the thorax, pelvis and thigh of the pathological hip would be necessary to measure all the features used in the present study. The iTUG test could be a simple and cost-effective means of assessing the functional deficits of patients with hip OA before and after THA, and it could help healthcare professionals to target patient-specific rehabilitation to close the gaps in function with asymptomatic controls. As recommended by the COSMIN taxonomy, the duration of the phases and the features should be assessed for reliability, validity and responsiveness before they are translated to clinical practice [40]. Recently, and with encouraging results, responsiveness to changes in multiple iTUG test parameters and the minimal detectable change in total TUG time have been evaluated among older adults (63.9 ± 6.1 years) undergoing a six-week physiotherapy programme [41]. Indeed, responsiveness to change was shown to have small to moderate effect sizes, and the minimal detectable change in total TUG time was 0.77 s. Moreover, Smith et al. [41] obtained positive feedback from patients and clinicians when implementing such an iTUG test in clinical settings, suggesting its good feasibility and acceptability. Nevertheless, further studies are needed to identify whether using such tests before surgery could help predict patients’ post-surgical function.

### Limitations

The present study’s main limitation is its lack of one-year and ongoing follow-up to assess changes in patient function in the long term, which is key to understanding the success of THA [42].

The patient population and the control group had a significant difference in terms of weight, but greater weight has been reported as a risk factor for hip OA [43], which could explain this.

The duration of test phases and the time differences between groups were relatively small, especially for the sit-to-stand and turning phases. Thus, those results should be taken with care as the difference could be within measurement accuracy of the algorithm. Moreover, data centring and reduction led to the selection of parameters with non-clinically meaningful differences. Indeed, the PCA identified parameters with differences that were within the range of measurement accuracy (1 degree), e.g. the peak thorax flexion and range of thorax obliquity during the sit-to-stand phase. Future studies could use measurement accuracy as a criterion during the feature selection.

The features measured in the present study were chosen based on previous ones, but other features might also be relevant when assessing levels of function in each phase. For example, we observed visually that patients moved within a continuum between two strategies in the turn-to-sit phase: those who seemed the most impaired walked right up to the chair, turned and then sat down, and those who seemed less impaired could turn and sit in one continuous movement, including one or more backward steps. These two strategies were similar to those described by Weiss et al. [35] among elderly patients: the distinct transition strategy and the overlapping transition strategy. The “distance chair to start of turn” feature was chosen with the assumption that a longer distance would imply that patients turned and sat simultaneously, i.e. using an overlapping transition strategy. This feature was continuous, as this study aimed to assess percentages of differences and change, but a categorical feature classifying patients’ strategies could be of interest to understand patients’ motor function.

## Conclusion

This study showed that, on average, patients with THA presented deficits in all four phases of the TUG test when compared to a control group before and six months after surgery, even though their motor function improved in all those phases after surgery. Walking seemed to be the main limitation facing patients before and six months after surgery. Furthermore, the large variability in functional level observed between patients indicates that the iTUG test was able to capture that variety among this population. Thus, this test could be appropriate for evaluating patient-specific levels of function in clinical settings. A biomechanical analysis of the TUG test with a simple IMU-based measurement system could provide cost-effective, relevant standard information for identifying patients’ specific functional deficits and could help target and monitor patient-specific rehabilitation in clinical practice.

## Supporting information

S1 Fig Figures to check the phases of patients. (PDF)

S2 Fig Figures to check the phases of the control group. (PDF)

S3 Fig Comparison of patient’s features and times of phases with the level of the control group. (PDF)

S1 Table Results of the principal component analysis. (PDF)

S1 File Algorithm for the detection of the timed up-and-go phases. (PDF)

S2 File Features description and selection. (PDF)

## References

[1] Liu X, Zi Y, Xiang L, Wang Y. Total hip arthroplasty: a review of advances, advantages and limitations. International journal of clinical and experimental medecine. 2015;8: 27–36. 25784971 PMC4358426

[2] Vissers MM, Bussmann JB, Verhaar JAN, Arends LR, Furlan AD, Reijman M. Recovery of Physical Functioning After Total Hip Arthroplasty: Systematic Review and Meta-Analysis of the Literature. Physical Therapy. 2011;91: 615–629. doi: 10.2522/ptj.20100201 21393418

[3] Bahl JS, Nelson MJ, Taylor M, Solomon LB, Arnold JB, Thewlis D. Biomechanical changes and recovery of gait function after total hip arthroplasty for osteoarthritis: a systematic review and meta-analysis. Osteoarthritis and Cartilage. 2018;26: 847–863. doi: 10.1016/j.joca.2018.02.897 29474993

[4] World Health Organization. How to use the ICF: A practical manual for using the International Classification of Functioning, Disability and Health (ICF). Geneva; 2013.

[5] Lovelock TM, Broughton NS, Williams CM. The Popularity of Outcome Measures for Hip and Knee Arthroplasties. Journal of Arthroplasty. 2018;33: 273–276. doi: 10.1016/j.arth.2017.08.024 28939034

[6] Nilsdotter AK, Lohmander LS, Klässbo M, Roos EM. Hip disability and osteoarthritis outcome score (HOOS)–validity and responsiveness in total hip replacement. BMC Musculoskeletal Disorders. 2003;4: 10. doi: 10.1186/1471-2474-4-10 12777182 PMC161815

[7] Dayton MR, Judd DL, Hogan CA, Stevens-Lapsley JE. Performance-Based Versus Self-Reported Outcomes Using the Hip Disability and Osteoarthritis Outcome Score after Total Hip Arthroplasty. American Journal of Physical Medicine and Rehabilitation. 2016;95: 132–138. doi: 10.1097/PHM.0000000000000357 26259051 PMC4772958

[8] Gandhi R, Tsvetkov D, Davey JR, Syed KA, Mahomed NN. Relationship between self-reported and performance-based tests in a hip and knee joint replacement population. Clinical Rheumatology. 2009;28: 253–257. doi: 10.1007/s10067-008-1021-y 18853222

[9] Podsiadlo D, Richardson S. The Timed “Up & Go”: A Test of Basic Functional Mobility for Frail Elderly Persons. Journal of the American Geriatrics Society. 1991;39: 142–148. doi: 10.1111/j.1532-5415.1991.tb01616.x 1991946

[10] Dobson F, Bennell K, Hinman RS, Abbott JH, Roos EM. Recommended performance-based tests to assess physical function in people diagnosed with hip or knee osteoarthritis. Melbourne; 2013. doi: 10.1016/j.joca.2013.05.002 23680877

[11] Caronni A, Sterpi I, Antoniotti P, Aristidou E, Nicolaci F, Picardi M, et al. Criterion validity of the instrumented Timed Up and Go test: A partial least square regression study. Gait & Posture. 2018;61: 287–293. doi: 10.1016/j.gaitpost.2018.01.015 29413799

[12] Petis SM, Howard JL, Lanting BA, Somerville LE, Vasarhelyi EM. Perioperative Predictors of Length of Stay After Total Hip Arthroplasty. The Journal of Arthroplasty. 2016;31: 1427–1430. doi: 10.1016/j.arth.2016.01.005 26869060

[13] Nankaku M, Tsuboyama T, Akiyama H, Kakinoki R, Fujita Y, Nishimura J, et al. Preoperative Prediction of Ambulatory Status at 6 Months After Total Hip Arthroplasty. Physical Therapy. 2012;93: 1–6. doi: 10.2522/ptj.20120016 23001525

[14] Sasaki K, Senda M, Nishida K, Ota H. Preoperative time required for the timed “up and go” test in women with hip osteoarthritis could predict a deep venous thrombosis complication after total hip arthroplasty. Acta medica Okayama. 2010;64: 197–201. doi: 10.18926/AMO/40012 20596131

[15] Caronni A, Picardi M, Aristidou E, Antoniotti P, Pintavalle G, Redaelli V, et al. How do patients improve their timed up and go test? Responsiveness to rehabilitation of the TUG test in elderly neurological patients. Gait & Posture. 2019;70: 33–38. doi: 10.1016/j.gaitpost.2019.02.010 30802642

[16] Coulthard JT, Treen TT, Oates AR, Lanovaz JL. Evaluation of an inertial sensor system for analysis of timed-up-and-go under dual-task demands. Gait & Posture. 2015;41: 882–887. doi: 10.1016/j.gaitpost.2015.03.009 25827680

[17] Cimolin V, Cau N, Malchiodi G, Aspesi V, Merenda V, Galli M, et al. Do wearable sensors add meaningful information to the Timed Up and Go test? A study on obese women. Journal of Electromyography and Kinesiology. 2019;44: 78–85. doi: 10.1016/j.jelekin.2018.12.001 30551006

[18] Salarian A, Horak FB, Zampieri C, Carlson-Kuhta P, Nutt JG, Aminian K. iTUG, a Sensitive and Reliable Measure of Mobility. IEEE Transactions on Neural Systems and Rehabilitation Engineering. 2010;18: 303–310. doi: 10.1109/TNSRE.2010.2047606 20388604 PMC2922011

[19] Zampieri C, Salarian A, Carlson-kuhta P, Aminian K, Nutt JG, Horak FB. The instrumented timed up and go test: potential outcome measure for disease modifying therapies in Parkinson’s disease. Journal of Neurology, Neurosurgery, and Psychiatry. 2010;81: 171–176. doi: 10.1136/jnnp.2009.173740 19726406 PMC3065923

[20] Hansen BJ, Hallows RK, Kelley SS. The Rottinger approach for total hip arthroplasty: Technique and review of the literature. Current Reviews in Musculoskeletal Medicine. 2011;4: 132–138. doi: 10.1007/s12178-011-9093-8 21826433 PMC3261250

[21] Moore AT. The self-locking metal hip prosthesis. Journal of Bone and Joint Surgery. 1957;39: 811–827. 13438939

[22] Altman R, Alarcón G, Appelrouth D, Bloch D, Borenstein D, Brandt K, et al. The American College of Rheumatology criteria for the classification and reporting of osteoarthritis of the hip. Arthritis & Rheumatism. 1991;34: 505–514. doi: 10.1002/art.1780340502 2025304

[23] Baker R, Leboeuf F, Reay J, Sangeux M. The Conventional Gait Model—Success and Limitations. In: Müller B, Wolf SI, editors. Handbook of Human Motion. Springer International Publishing AG; 2017. pp. 1–19. doi: 10.1007/978-3-319-30808-1_25–2

[24] Gløersen Ø, Federolf P. Predicting Missing Marker Trajectories in Human Motion Data Using Marker Intercorrelations. PloS one. 2016. doi: 10.1371/journal.pone.0152616 27031243 PMC4816448

[25] Beyea J, Mcgibbon CA, Sexton A, Noble J, Connell CO. Convergent Validity of a Wearable Sensor System for Measuring Sub-Task Performance during the Timed Up-And-Go Test. Sensors. 2017;17: 1–18. doi: 10.3390/s17040934 28441748 PMC5426930

[26] Barre A, Armand S. Biomechanical ToolKit: Open-source framework to visualize and process biomechanical data. Computer Methods and Programs in Biomedicine. 2014;114: 80–87. doi: 10.1016/j.cmpb.2014.01.012 24548899

[27] Janssen WGM, Bussmann HBJ, Stam HJ. Determinants of the sit-to-stand movement: A review. Physical Therapy. 2002;82: 866–879. doi: 10.1093/ptj/82.9.866 12201801

[28] Abujaber SB, Marmon AR, Pozzi F, Rubano JJ, Zeni JA. Sit-To-Stand Biomechanics Before and After Total Hip Arthroplasty. Journal of Arthroplasty. 2015;30: 2027–2033. doi: 10.1016/j.arth.2015.05.024 26117068 PMC4640963

[29] Aissaoui R, Dansereau J. Biomechanical analysis and modelling of sit to stand task: a literature review. Proceedings of the IEEE International Conference on Systems, Man and Cybernetics. 1999;1: 141–146. doi: 10.1109/icsmc.1999.814072

[30] Alexander NB, Schultz AB, Warwich DN. Rising from a chair: Effects of age functional ability on performance biomechanics. Journals of Gerontology. 1991;46: 91–98. doi: 10.1093/geronj/46.3.m91 2030272

[31] Nankaku M, Tsuboyama T, Kakinoki R, Kawanabe K, Kanzaki H, Mito Y, et al. Gait analysis of patients in early stages after total hip arthroplasty: Effect of lateral trunk displacement on walking efficiency. Journal of Orthopaedic Science. 2007;12: 550–554. doi: 10.1007/s00776-007-1178-2 18040637

[32] Hollands KL, Hollands MA, Zietz D, Miles Wing A, Wright C, Van Vliet P. Kinematics of turning 180° during the timed up and go in stroke survivors with and without falls history. Neurorehabilitation and Neural Repair. 2010;24: 358–367. doi: 10.1177/1545968309348508 19822720

[33] Schot PK, Knutzen KM, Poole SM, Mrotek LA. Sit-to-stand performance of older adults following strength training. Research Quarterly for Exercise and Sport. 2003;74: 1–8. doi: 10.1080/02701367.2003.10609058 12659470

[34] Boonstra MC, Schreurs BW, Verdonschot N. The Sit-to-Stand Movement: Differences in Performance Between Patients After Primary Total Hip Arthroplasty and Revision Total Hip Arthroplasty With Acetabular Bone Impaction Grafting. Physical Therapy. 2011;91: 547–554. doi: 10.2522/ptj.20090376 21350030

[35] Weiss A, Mirelman A, Giladi N, Barnes LL, Bennett DA, Buchman AS, et al. Transition between the Timed Up and Go turn to sit subtasks: is timing everything? Journal of the American Medical Directors Association. 2016;17. doi: 10.1016/j.jamda.2015.07.005 27569715 PMC5004894

[36] Baker R, McGinley JL, Schwartz MH, Beynon S, Rozumalski A, Graham HK, et al. The Gait Profile Score and Movement Analysis Profile. Gait & Posture. 2009;30: 265–269. doi: 10.1016/j.gaitpost.2009.05.020 19632117

[37] Massey FJ. The Kolmogorov-Smirnov Test for Goodness of Fit. Journal of the American Statistical Association. 1951;46: 68–78. doi: 10.1080/01621459.1951.10500769

[38] Cattell RB. The Scree Test For The Number Of Factors. Multivariate Behavioral Research. 1966;1: 245–276. doi: 10.1207/s15327906mbr0102_10 26828106

[39] Holm S. A Simple Sequentially Rejective Multiple Test Procedure. Scandinavian Journal of Statistics. 1979;6: 65–70. doi: 10.2307/4615733

[40] Mokkink LB, Terwee CB, Patrick DL, Alonso J, Stratford PW, Knol DL, et al. The COSMIN checklist for assessing the methodological quality of studies on measurement properties of health status measurement instruments: An international Delphi study. Quality of Life Research. 2010;19: 539–549. doi: 10.1007/s11136-010-9606-8 20169472 PMC2852520

[41] Smith E, Cunningham C, Greene BR, Mccarthy U, Catherine P. Detecting subtle mobility changes among older adults: the Quantitative Timed Up and Go test. Aging Clinical and Experimental Research. 2020. doi: 10.1007/s40520-020-01733-7 33098079

[42] Rylander JH. CORR Insights®: Gait Analysis Reveals that Total Hip Arthroplasty Increases Power Production in the Hip During Level Walking and Stair Climbing. Clinical orthopaedics and related research. 2019;477: 1848–1850. doi: 10.1097/CORR.0000000000000865 31335602 PMC7000027

[43] Aresti N, Kassam J, Nicholas N, Achan P. Hip osteoarthritis. BMJ (Online). 2016;354: 1–10. doi: 10.1136/bmj.i3405 27383835

