# Supplementary material for: Which functional tasks present the largest deficits for patients with total hip arthroplasty before and six months after surgery? A study of the timed up-and-go test phases
Source: PLoS One. 2021 Sep 10;16(9):e0255037. doi: 10.1371/journal.pone.0255037 (PMC8432811; doi:10.1371/journal.pone.0255037)
Supplement: S1 Fig — (PDF) [file pone.0255037.s001.pdf]

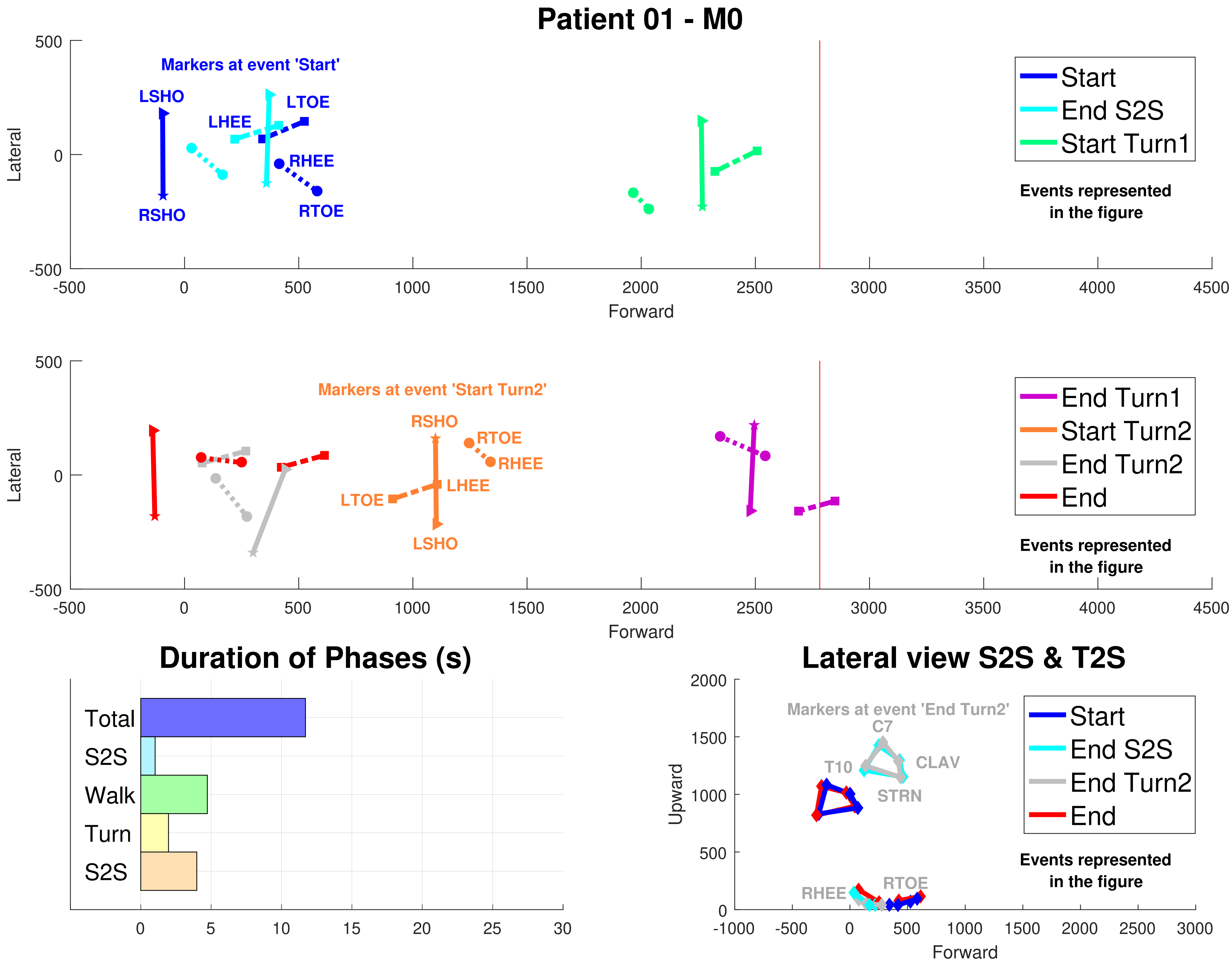

Patient 01 - M0
Markers at event 'Start'
LSHO
LHEE
LTOE
RHEE
RSHO
RTOE
Start
End S2S
Start Turn1
Events represented in the figure
Lateral
Forward
Markers at event 'Start Turn2'
RSHO
RTOE
RHEE
LHEE
LTOE
LSHO
End Turn1
Start Turn2
End Turn2
End
Events represented in the figure
Duration of Phases (s)
Total
S2S
Walk
Turn
S2S
Lateral view S2S & T2S
Markers at event 'End Turn2'
C7
T10
CLAV
STRN
RHEE
RTOE
Start
End S2S
End Turn2
End
Events represented in the figure
Upward
Forward

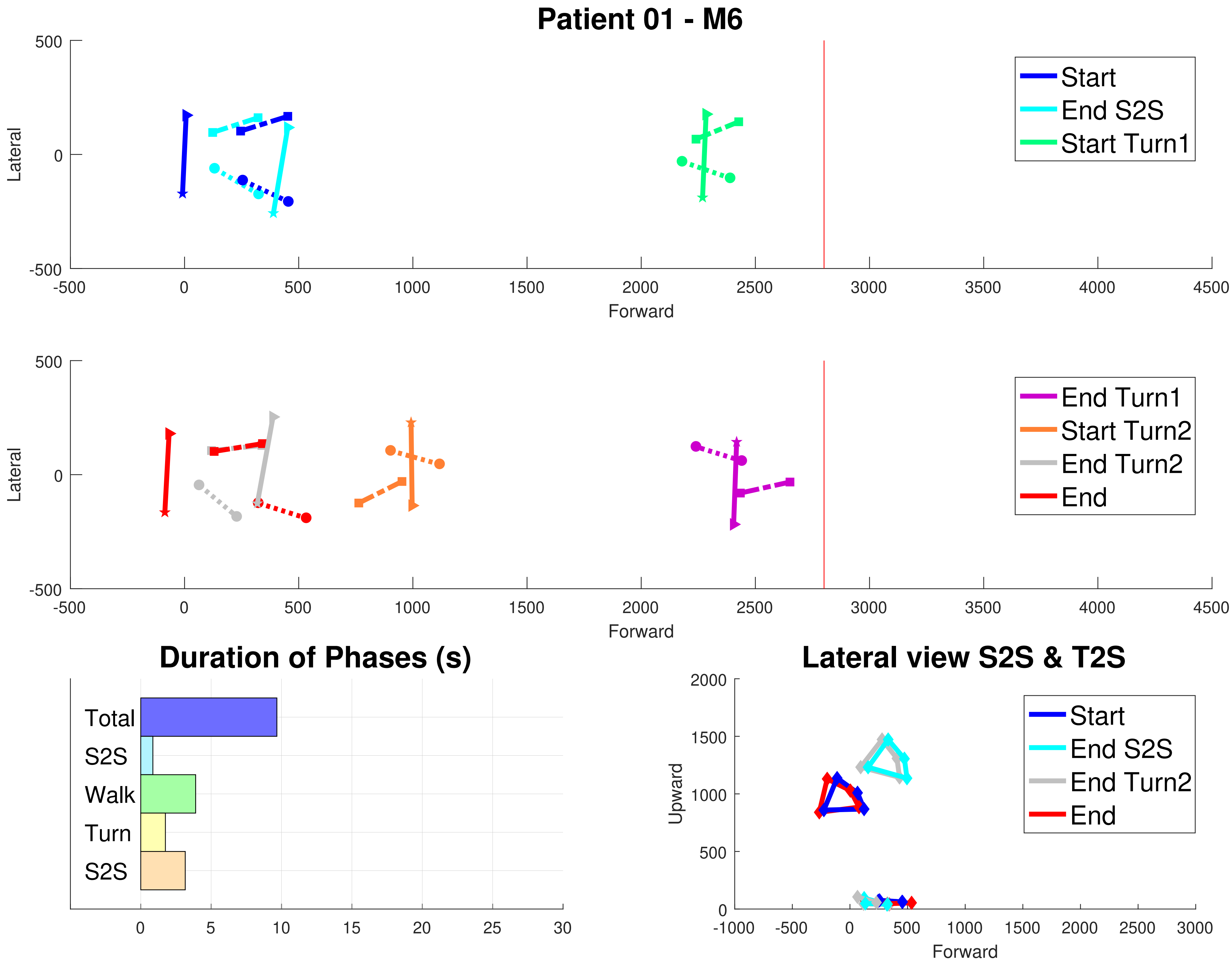

Patient 01 - M6
Lateral
Forward
Start
End S2S
Start Turn1
End Turn1
Start Turn2
End Turn2
End
500
0
-500
-500
0
500
1000
1500
2000
2500
3000
3500
4000
4500
Duration of Phases (s)
Total
S2S
Walk
Turn
S2S
0
5
10
15
20
25
30
Lateral view S2S & T2S
Upward
Forward
2000
1500
1000
500
0
-1000
-500
0
500
1000
1500
2000
2500
3000
Start
End S2S
End Turn2
End

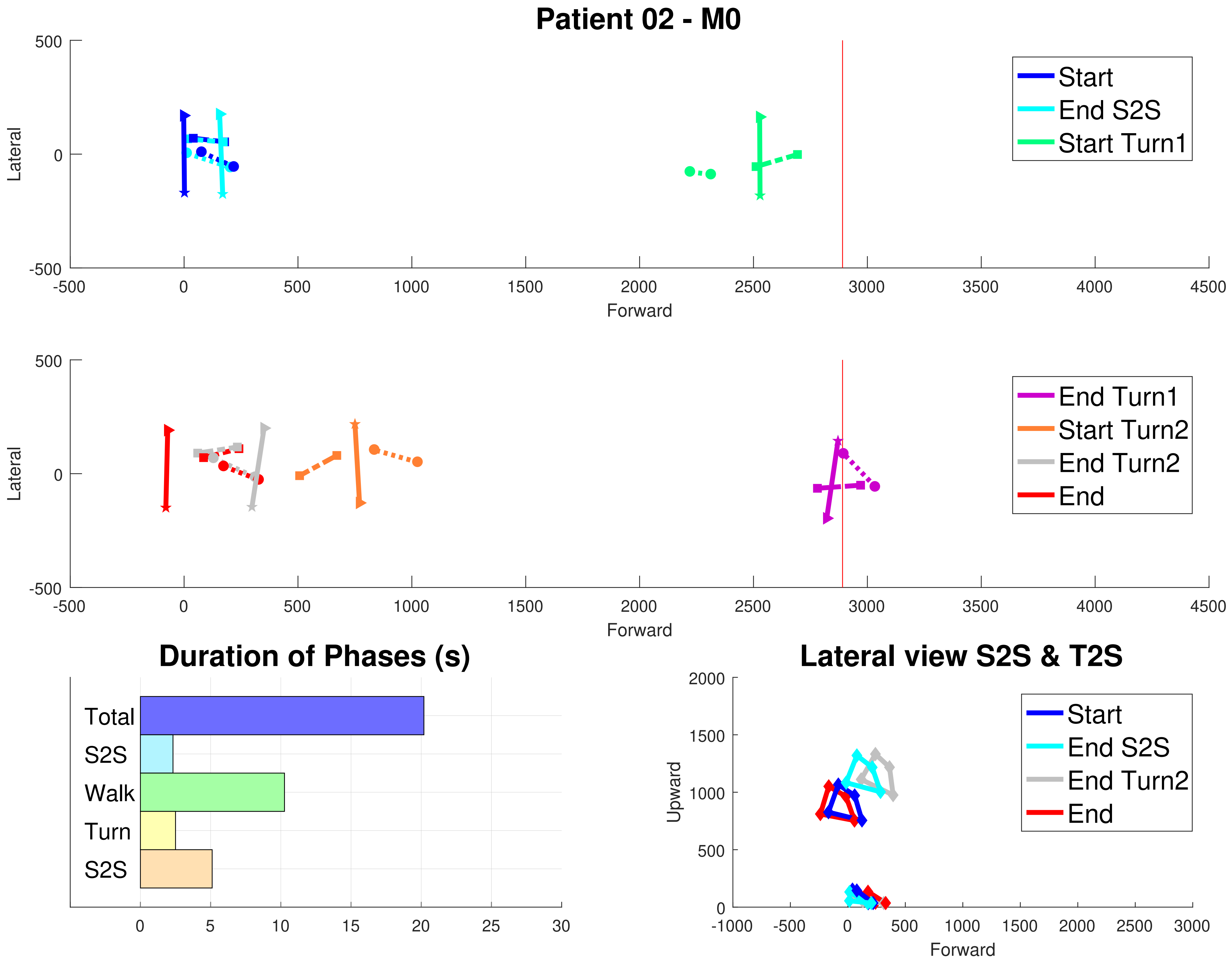
Patient 02 - M0
Lateral
Forward
Start
End S2S
Start Turn1
End Turn1
Start Turn2
End Turn2
End
Duration of Phases (s)
Total
S2S
Walk
Turn
S2S
Lateral view S2S & T2S
Upward
Forward
Start
End S2S
End Turn2
End

Patient 02 - M6

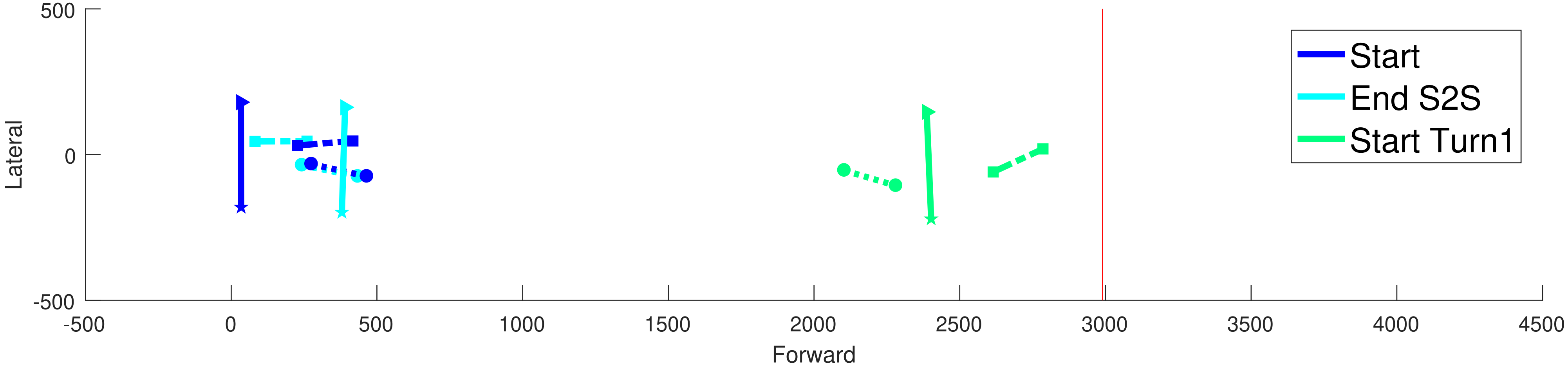

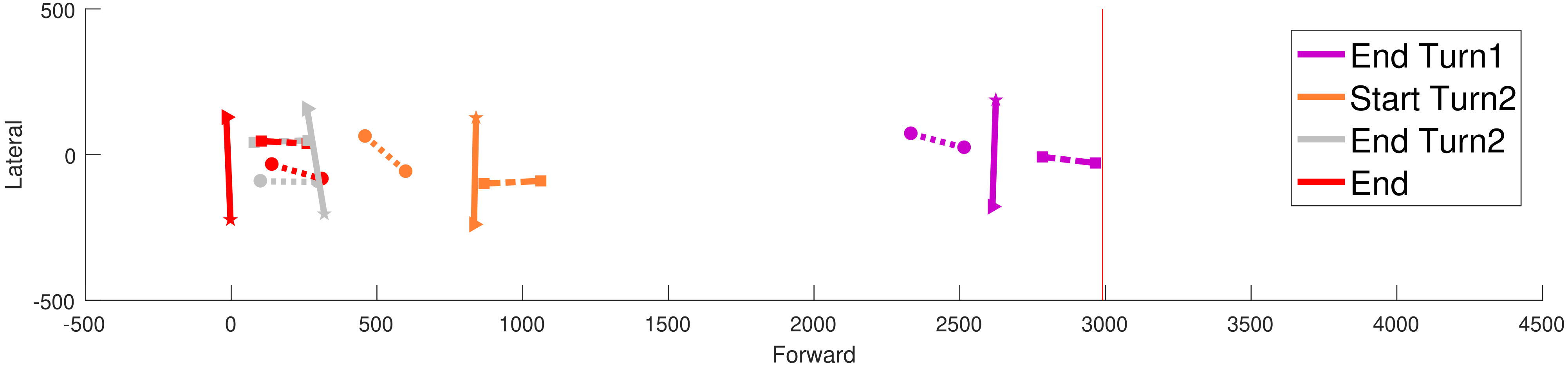

Duration of Phases (s)

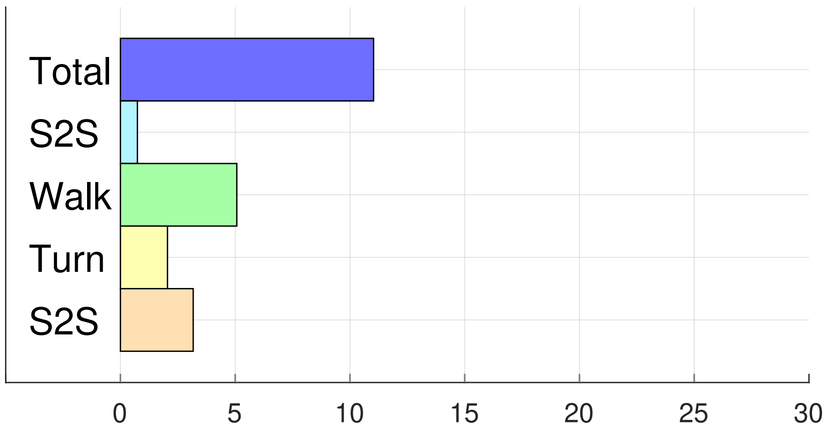

Lateral view S2S & T2S

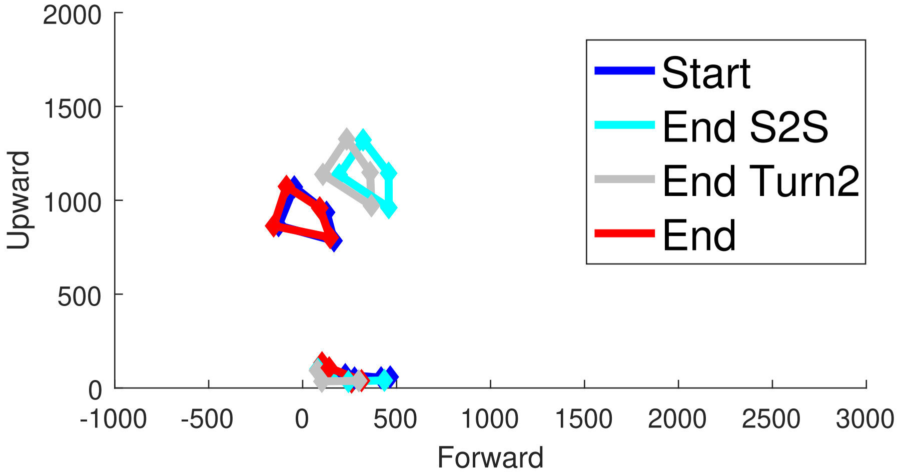

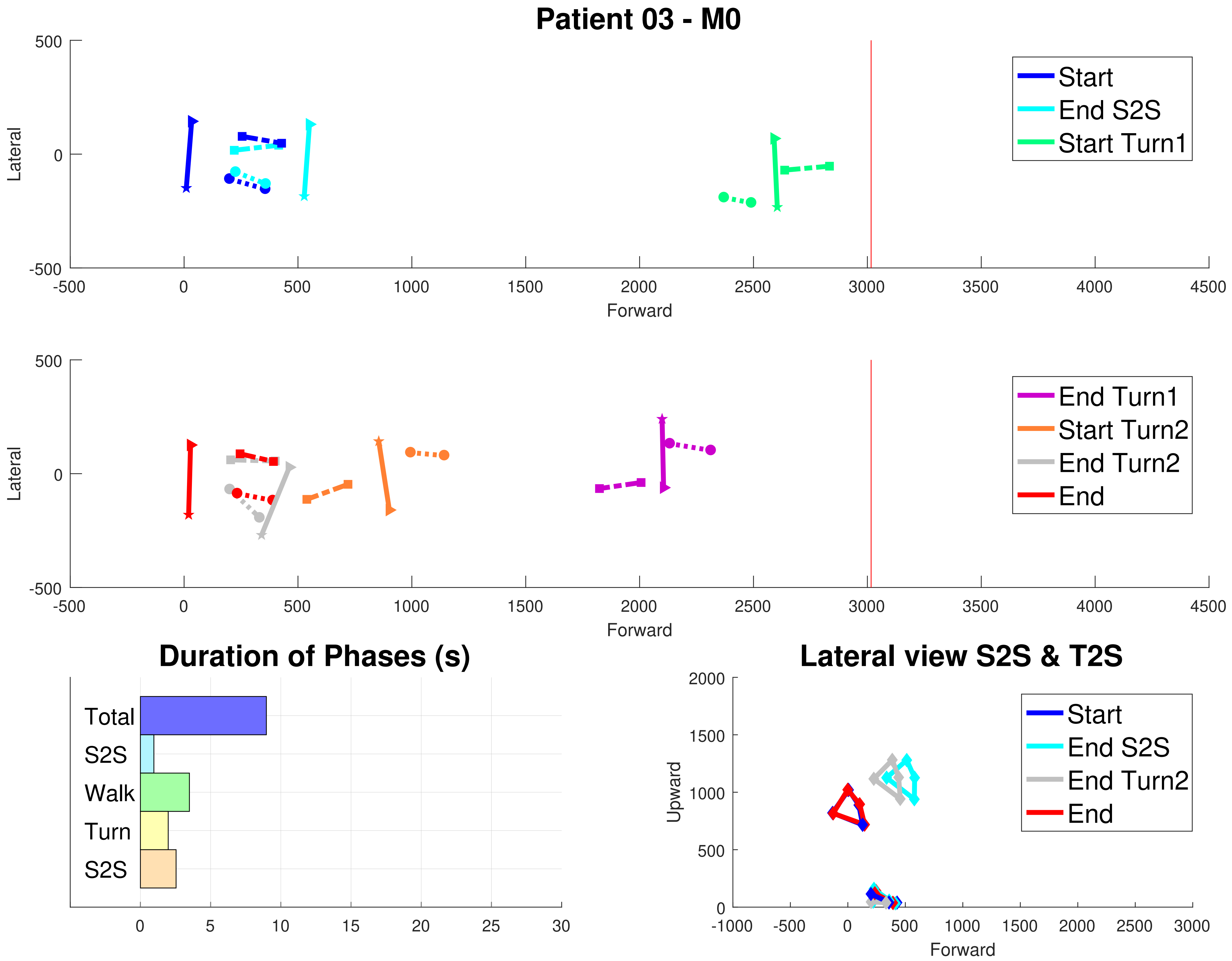
Patient 03 - M0
Lateral
Forward
Start
End S2S
Start Turn1
End Turn1
Start Turn2
End Turn2
End
Duration of Phases (s)
Total
S2S
Walk
Turn
S2S
Lateral view S2S & T2S
Upward
Forward
Start
End S2S
End Turn2
End

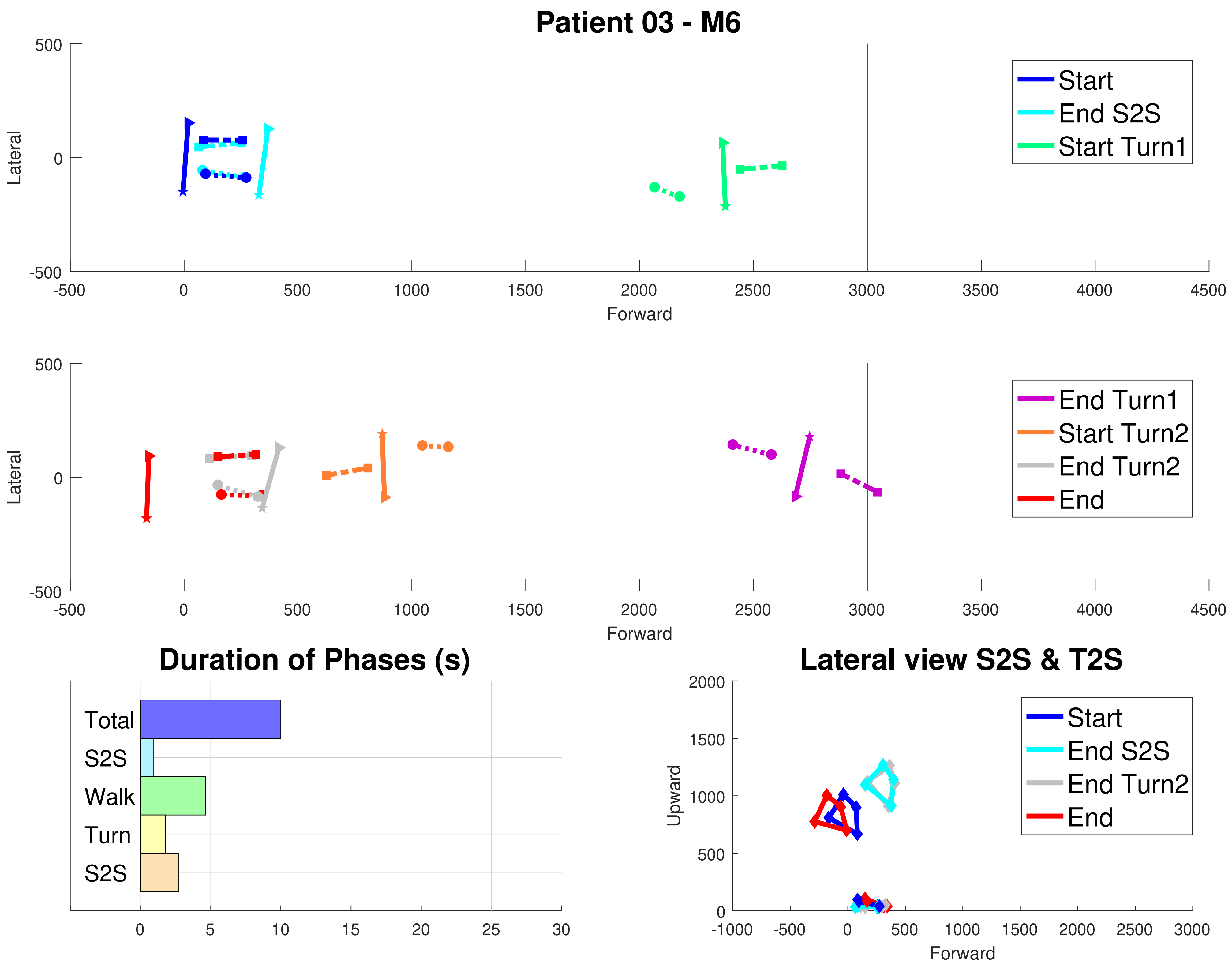

Patient 03 - M6
Lateral
Forward
Start
End S2S
Start Turn1
End Turn1
Start Turn2
End Turn2
End
Duration of Phases (s)
Total
S2S
Walk
Turn
S2S
Lateral view S2S & T2S
Upward
Forward
Start
End S2S
End Turn2
End

Patient 04 - M0

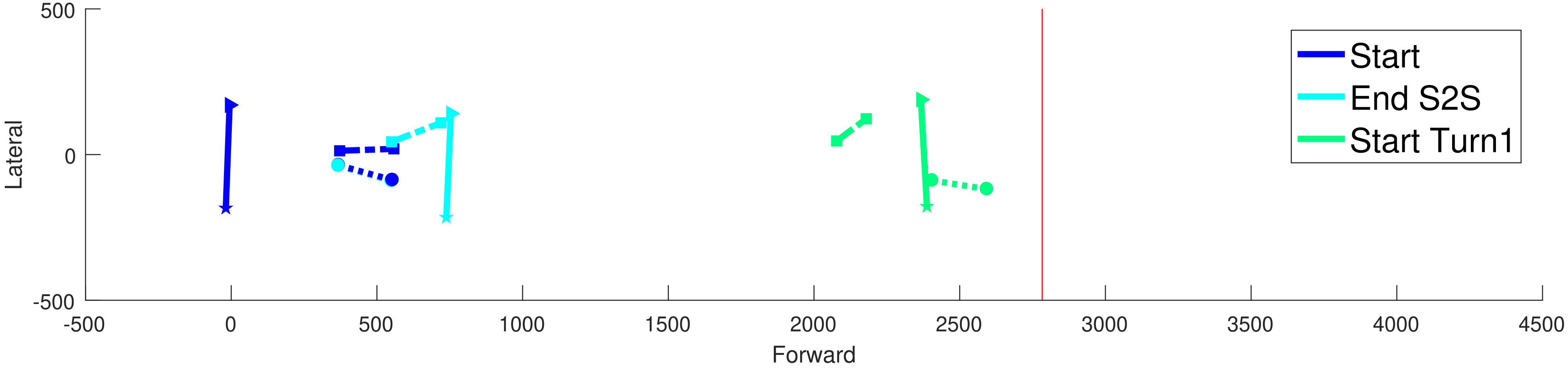

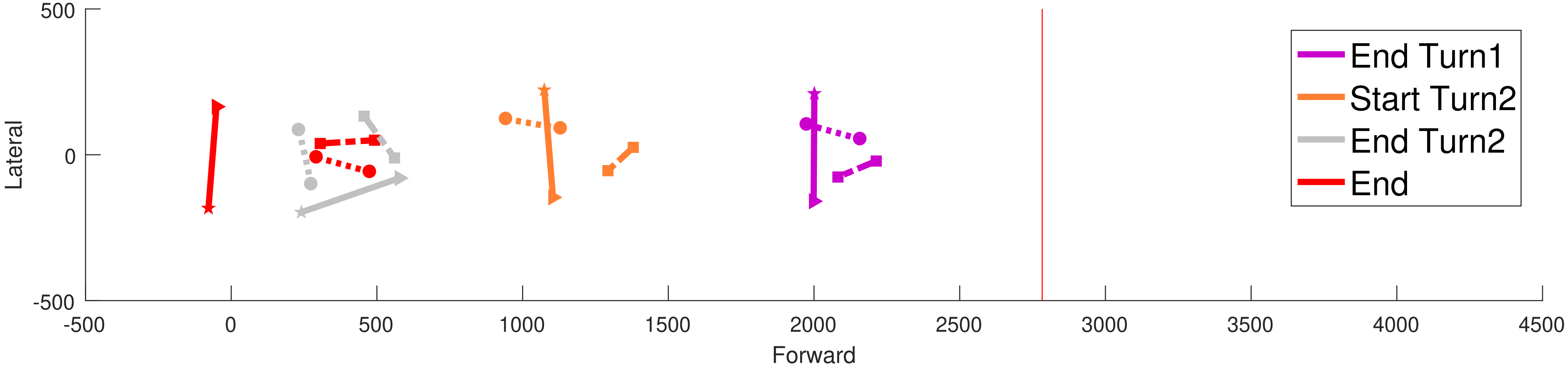

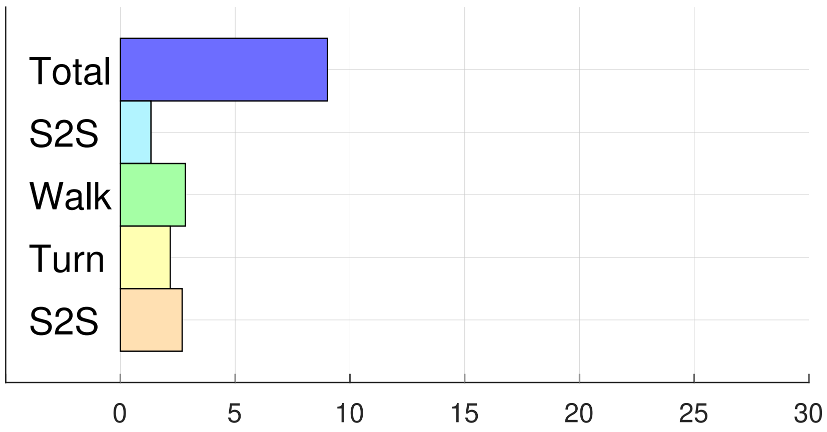

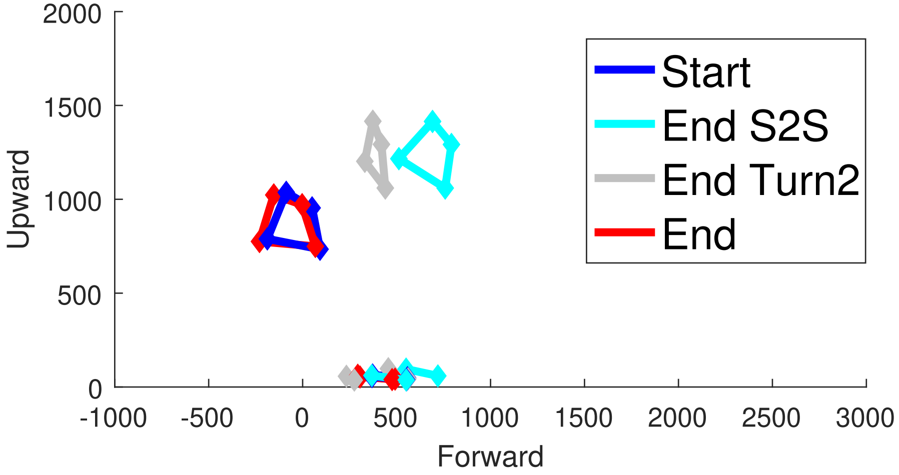

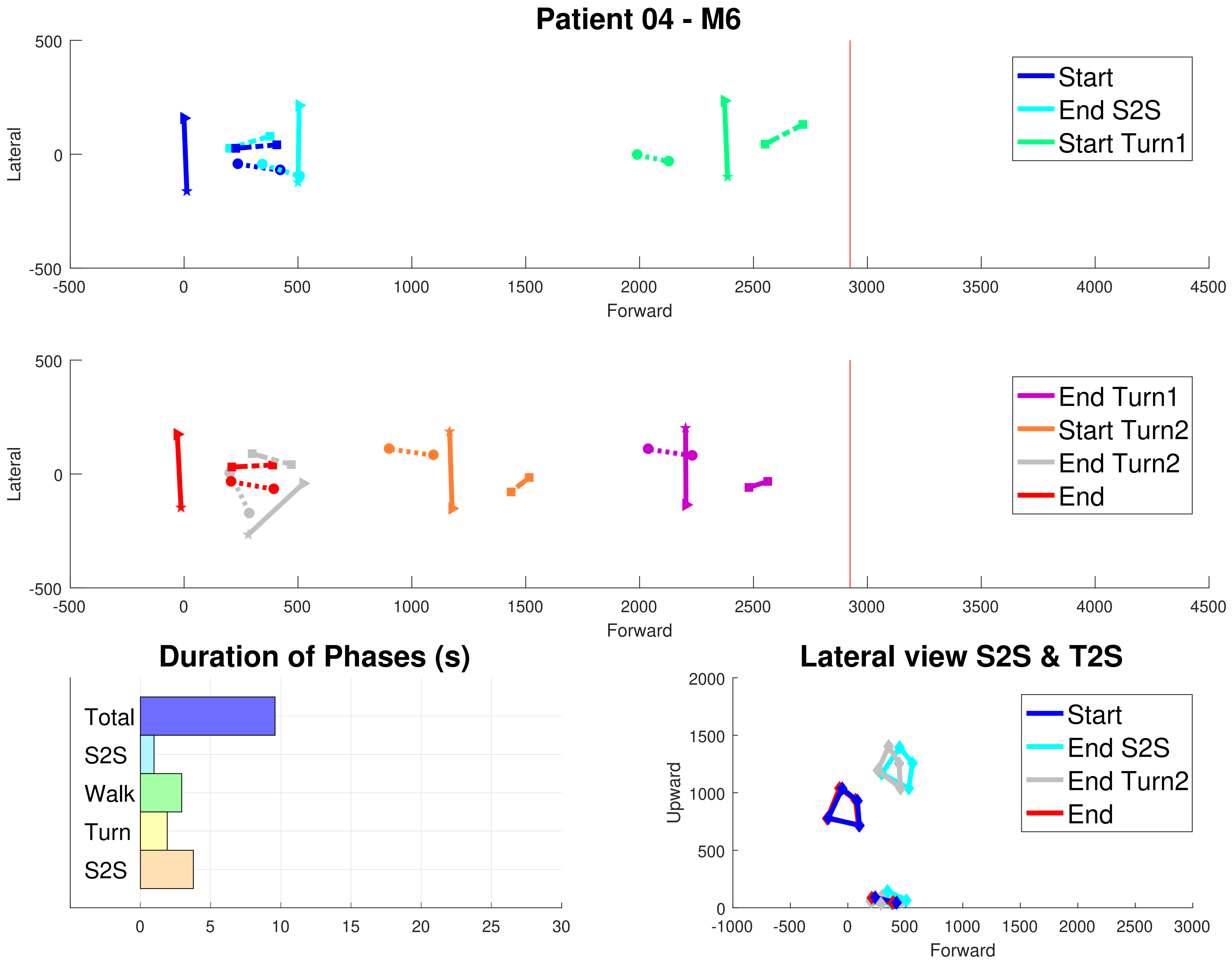

Patient 04 - M6
Lateral
Forward
Start
End S2S
Start Turn1
End Turn1
Start Turn2
End Turn2
End
Duration of Phases (s)
Total
S2S
Walk
Turn
S2S
Lateral view S2S & T2S
Upward

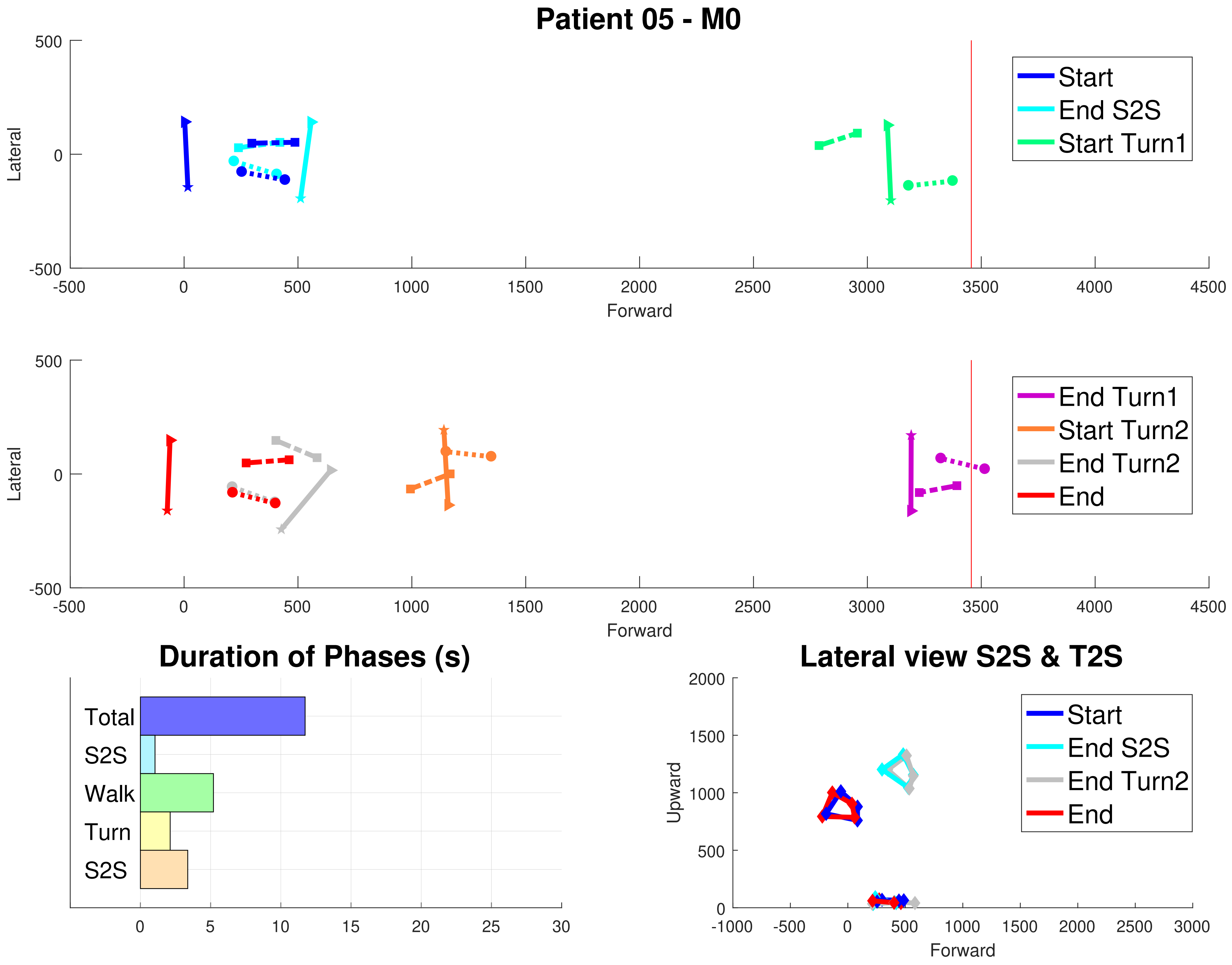

Patient 05 - M0
Lateral
Forward
Start
End S2S
Start Turn1
End Turn1
Start Turn2
End Turn2
End
Duration of Phases (s)
Total
S2S
Walk
Turn
S2S
Lateral view S2S & T2S
Upward
Forward
Start
End S2S
End Turn2
End

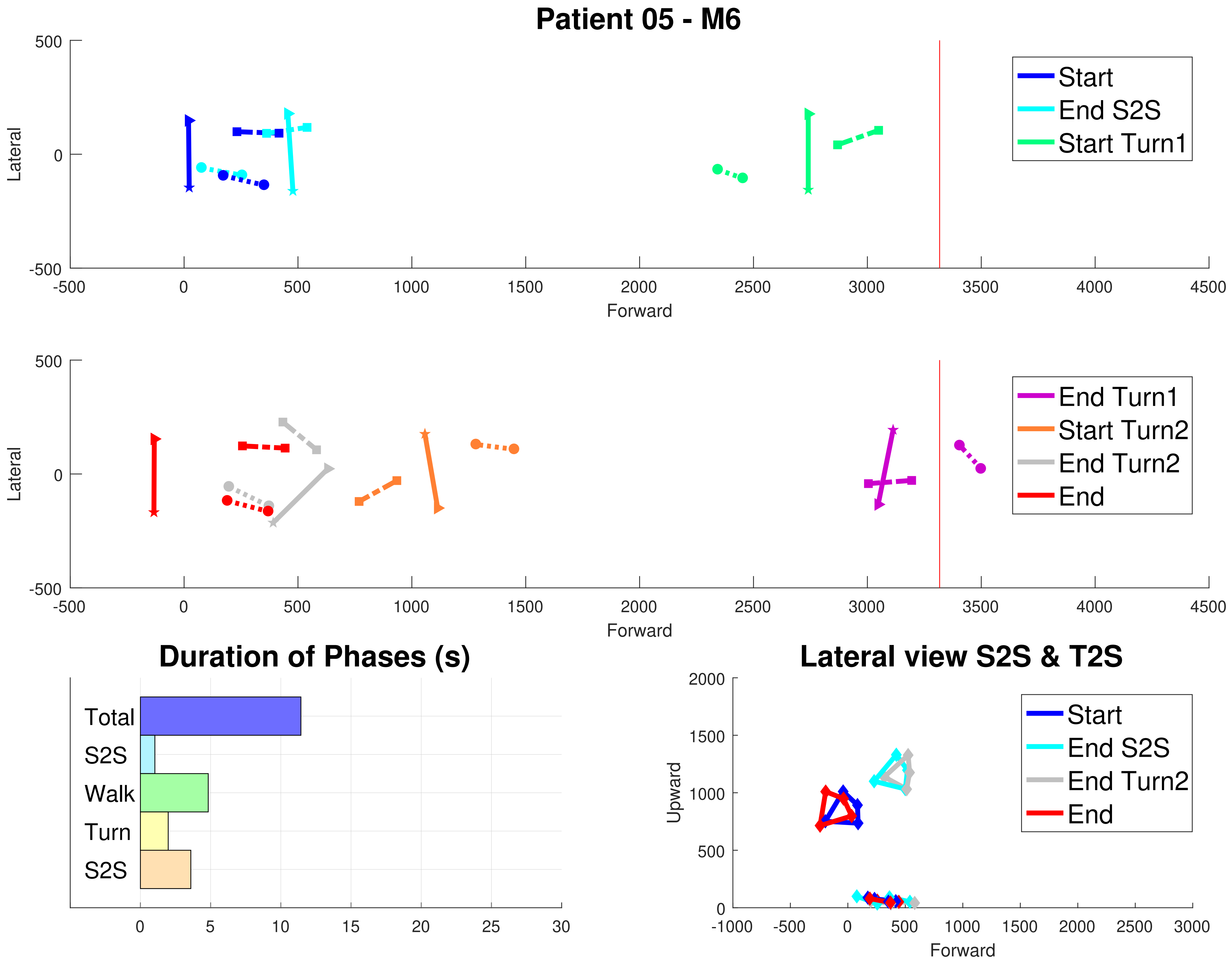

Patient 05 - M6
Lateral
Forward
Start
End S2S
Start Turn1
End Turn1
Start Turn2
End Turn2
End
Duration of Phases (s)
Total
S2S
Walk
Turn
S2S
Lateral view S2S & T2S
Upward

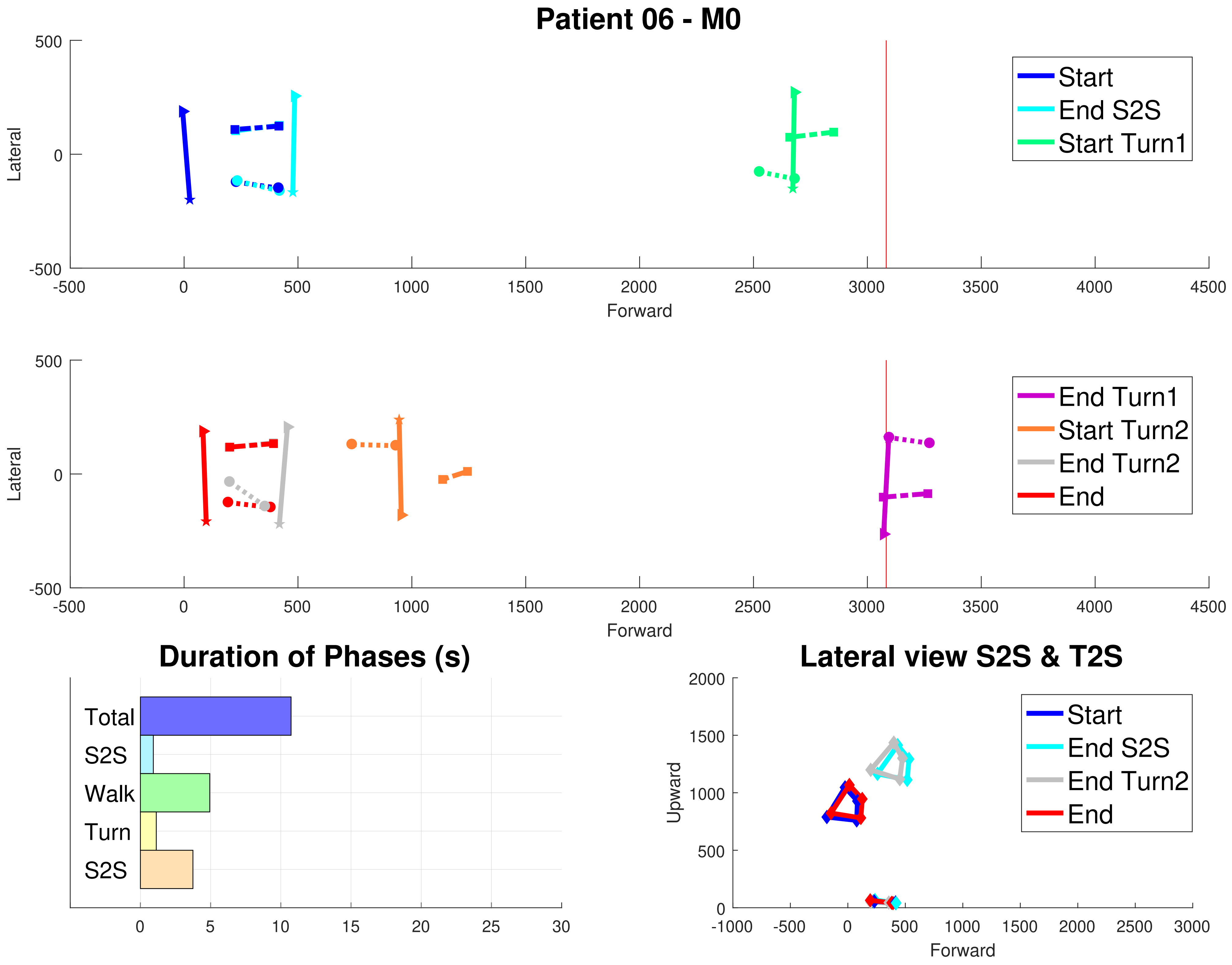
Patient 06 - M0
Lateral
Forward
500
0
-500
-500
0
500
1000
1500
2000
2500
3000
3500
4000
4500
Start
End S2S
Start Turn1
End Turn1
Start Turn2
End Turn2
End
Duration of Phases (s)
Total
S2S
Walk
Turn
S2S
0
5
10
15
20
25
30
Lateral view S2S & T2S
Upward
2000
1500
1000
500
0
-1000
-500
0
500
1000
1500
2000
2500
3000
Start
End S2S
End Turn2
End

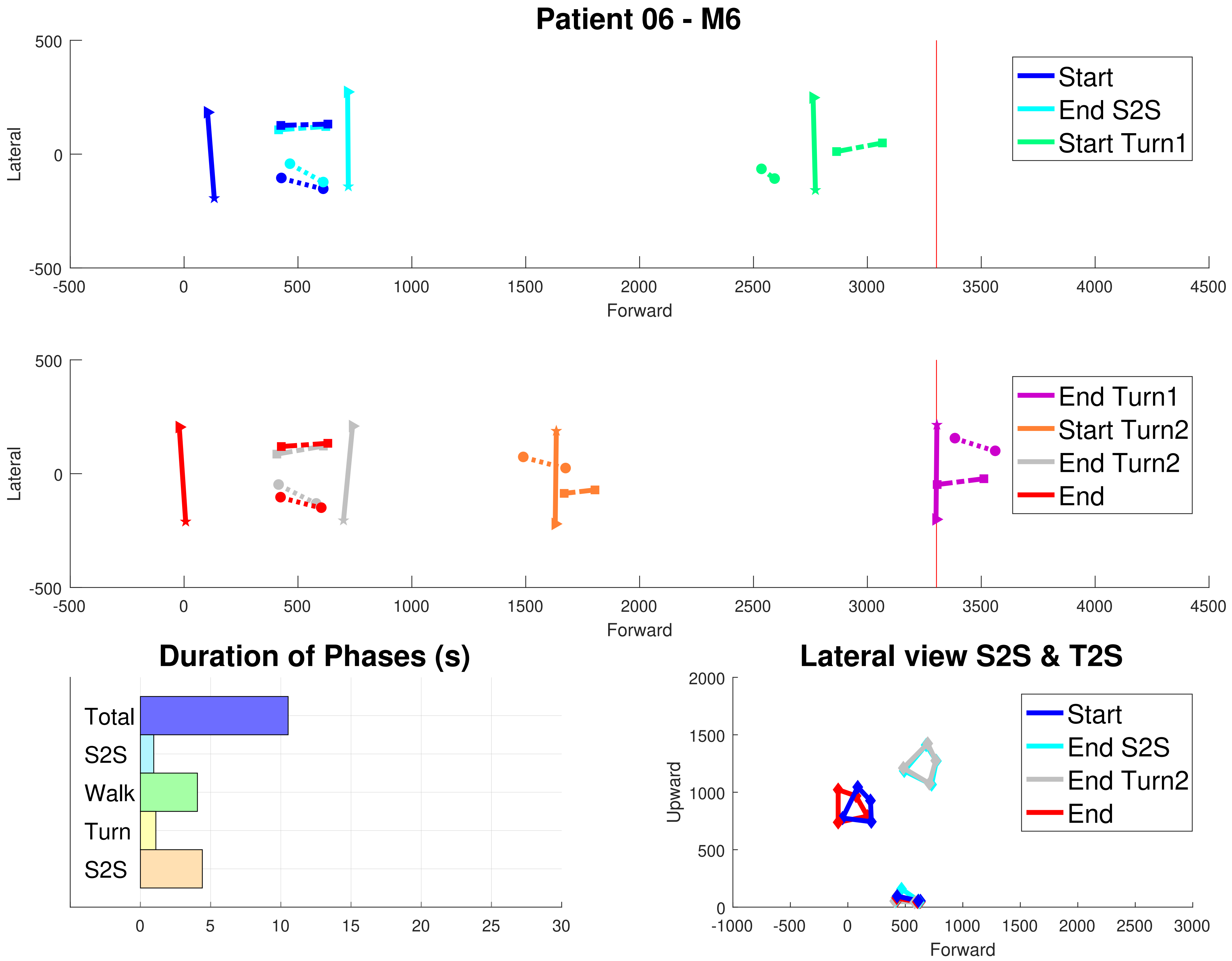

Patient 06 - M6
Lateral
Forward
Start
End S2S
Start Turn1
End Turn1
Start Turn2
End Turn2
End
Duration of Phases (s)
Total
S2S
Walk
Turn
S2S
Lateral view S2S & T2S
Upward
Forward
Start
End S2S
End Turn2
End

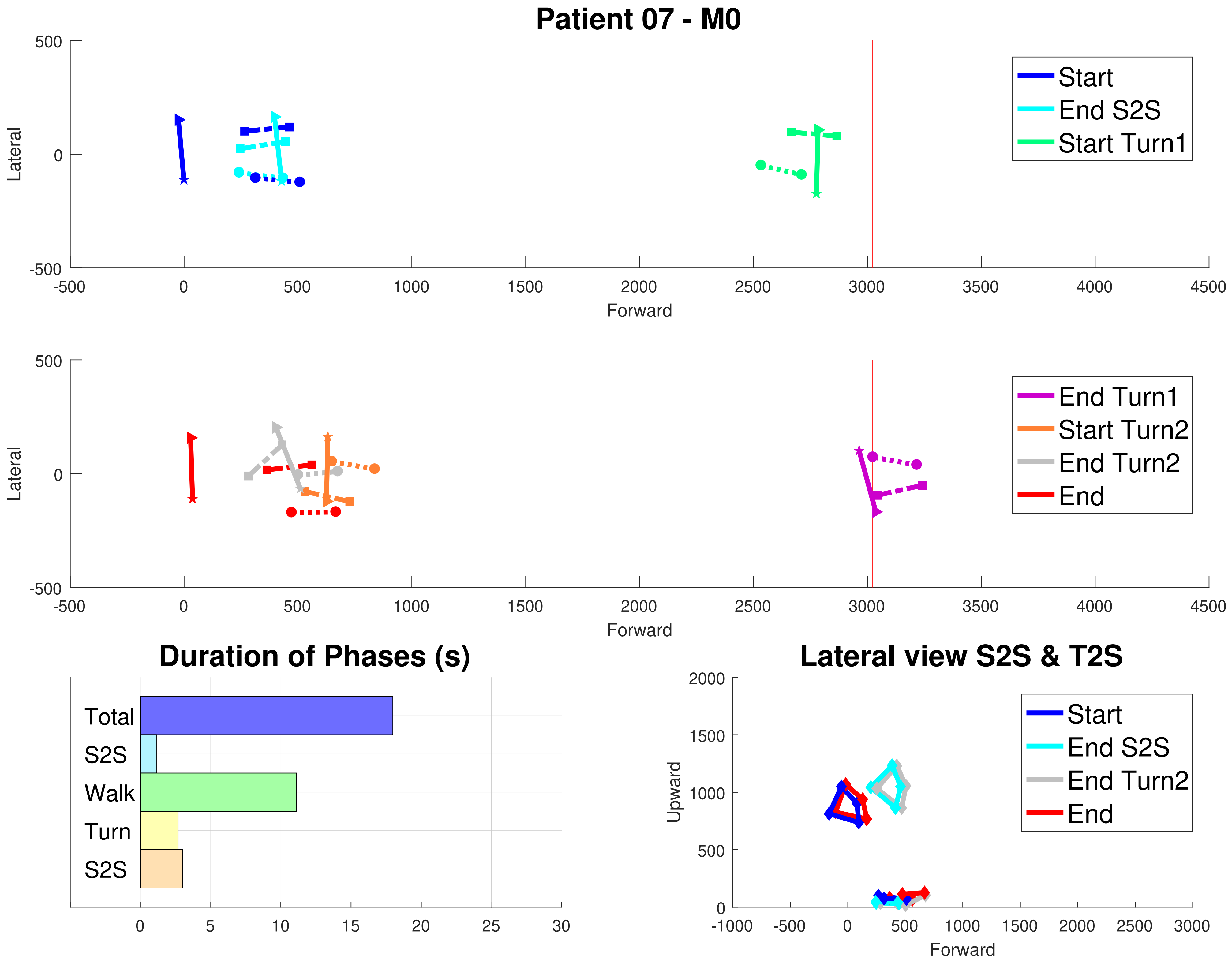

Patient 07 - M0
Lateral
Forward
Start
End S2S
Start Turn1
End Turn1
Start Turn2
End Turn2
End
Duration of Phases (s)
Total
S2S
Walk
Turn
S2S
Lateral view S2S & T2S
Upward
Forward
Start
End S2S
End Turn2
End

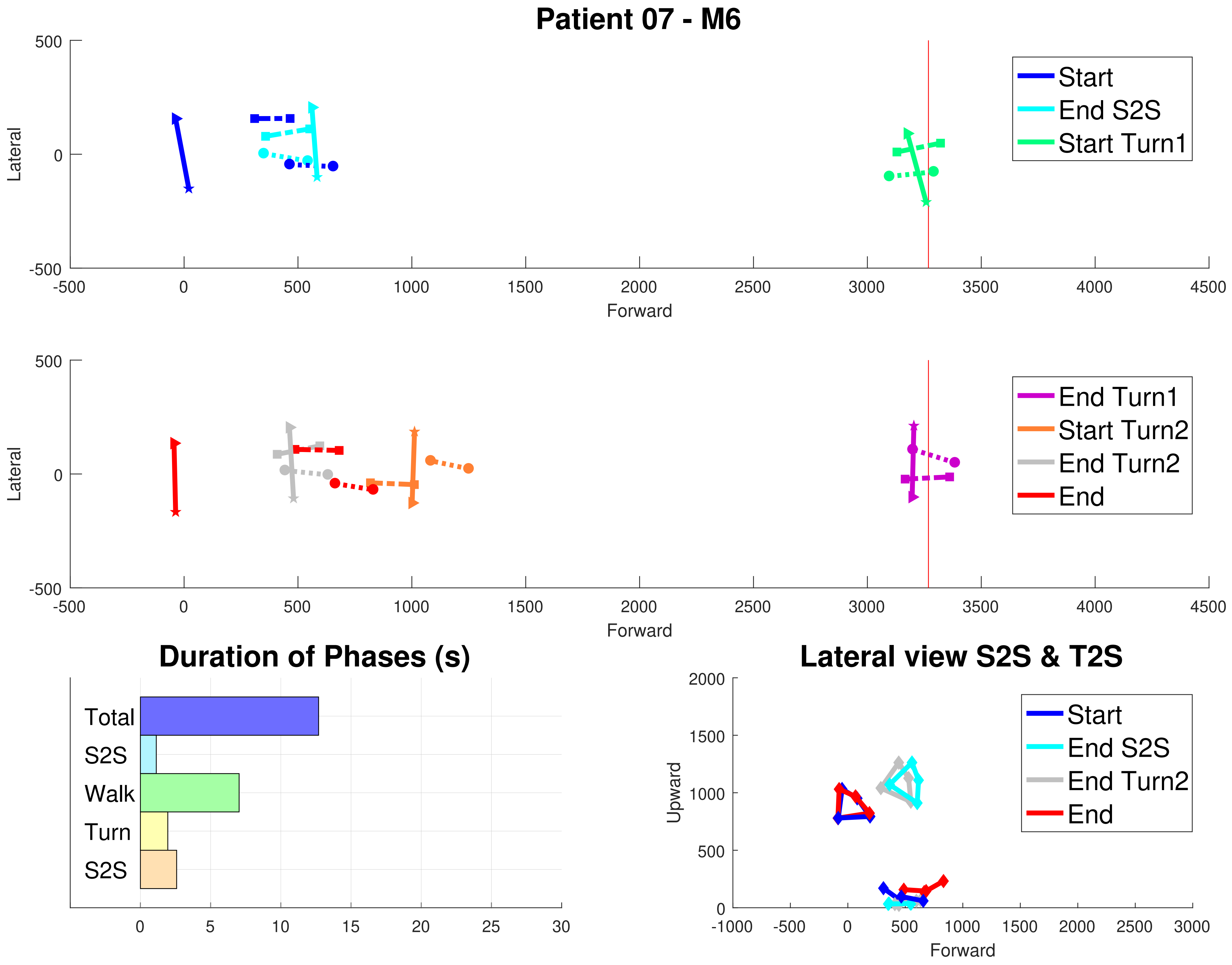

Patient 07 - M6
Lateral
Forward
500
0
-500
-500
0
500
1000
1500
2000
2500
3000
3500
4000
4500
Start
End S2S
Start Turn1
End Turn1
Start Turn2
End Turn2
End
Duration of Phases (s)
Total
S2S
Walk
Turn
S2S
0
5
10
15
20
25
30
Lateral view S2S & T2S
Upward
2000
1500
1000
500
-1000
-500
3000
Start
End S2S
End Turn2
End

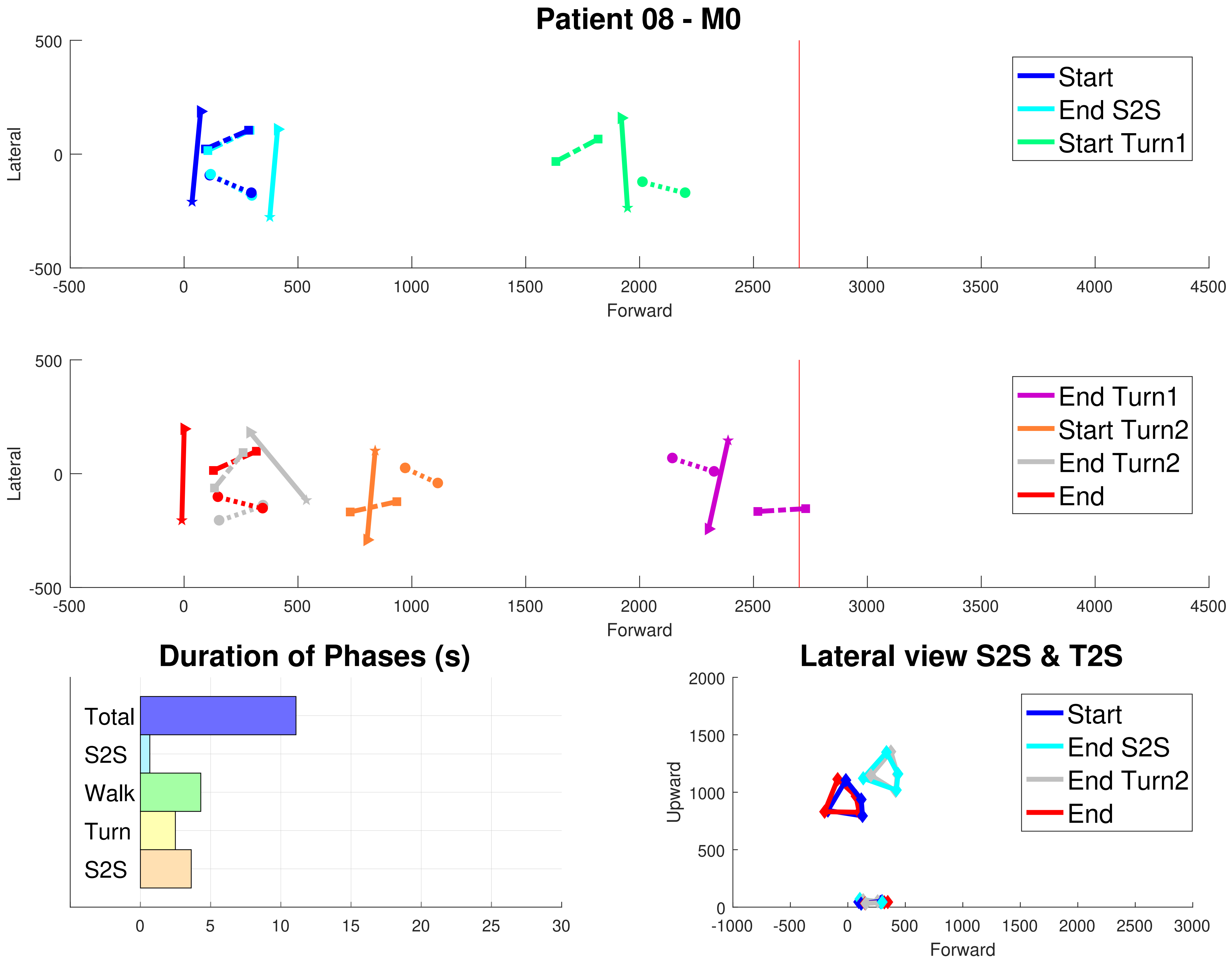
Patient 08 - M0
Lateral
Forward
500
0
-500
-500
0
500
1000
1500
2000
2500
3000
3500
4000
4500
Start
End S2S
Start Turn1
End Turn1
Start Turn2
End Turn2
End
Duration of Phases (s)
Total
S2S
Walk
Turn
S2S
0
5
10
15
20
25
30
Lateral view S2S & T2S
Upward
2000
1500
1000
500
-1000
-500
Start
End S2S
End Turn2
End

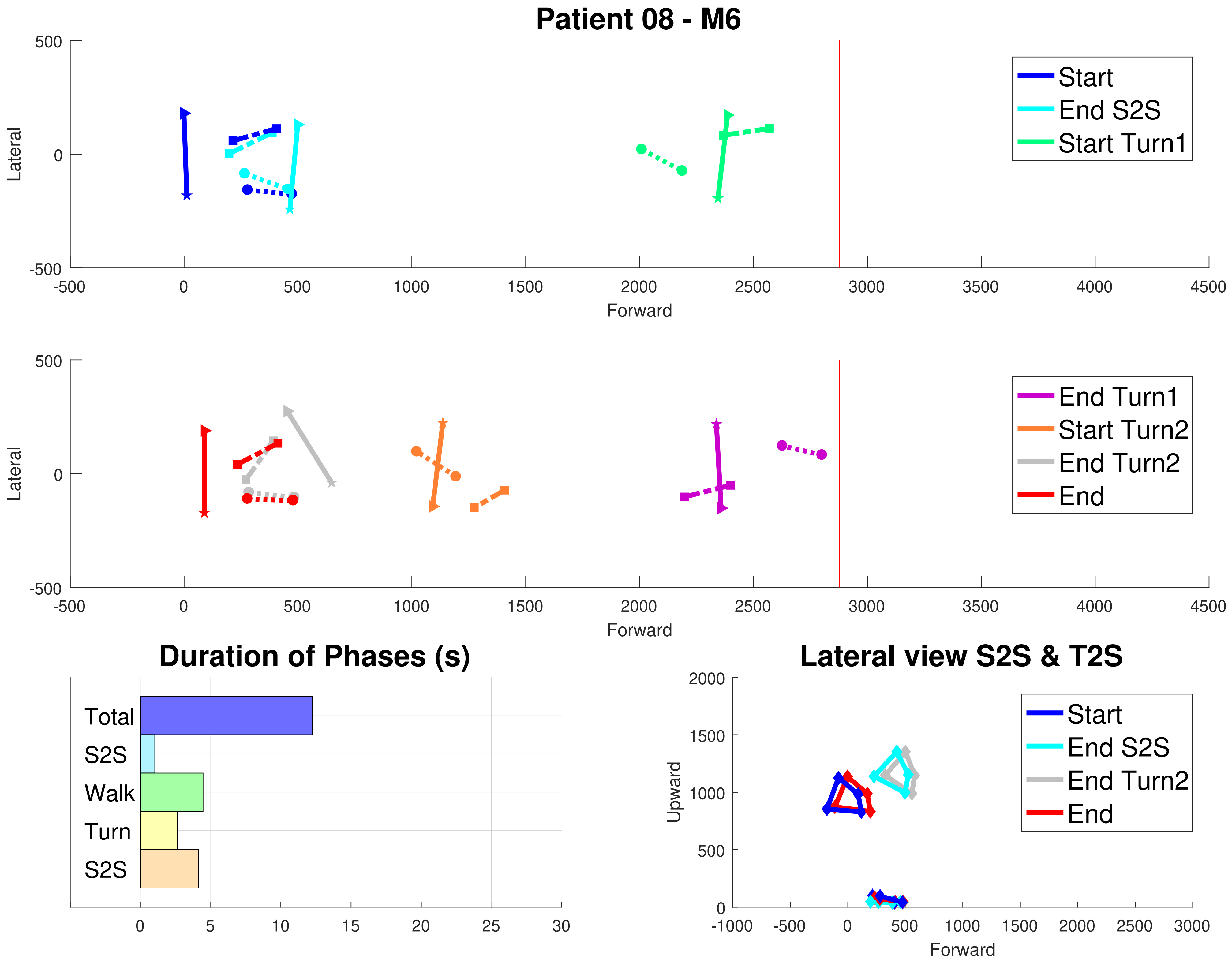
Patient 08 - M6
Start
End S2S
Start Turn1
Lateral
Forward
End Turn1
Start Turn2
End Turn2
End
Duration of Phases (s)
Total
S2S
Walk
Turn
S2S
Lateral view S2S & T2S
Upward
Start
End S2S
End Turn2
End

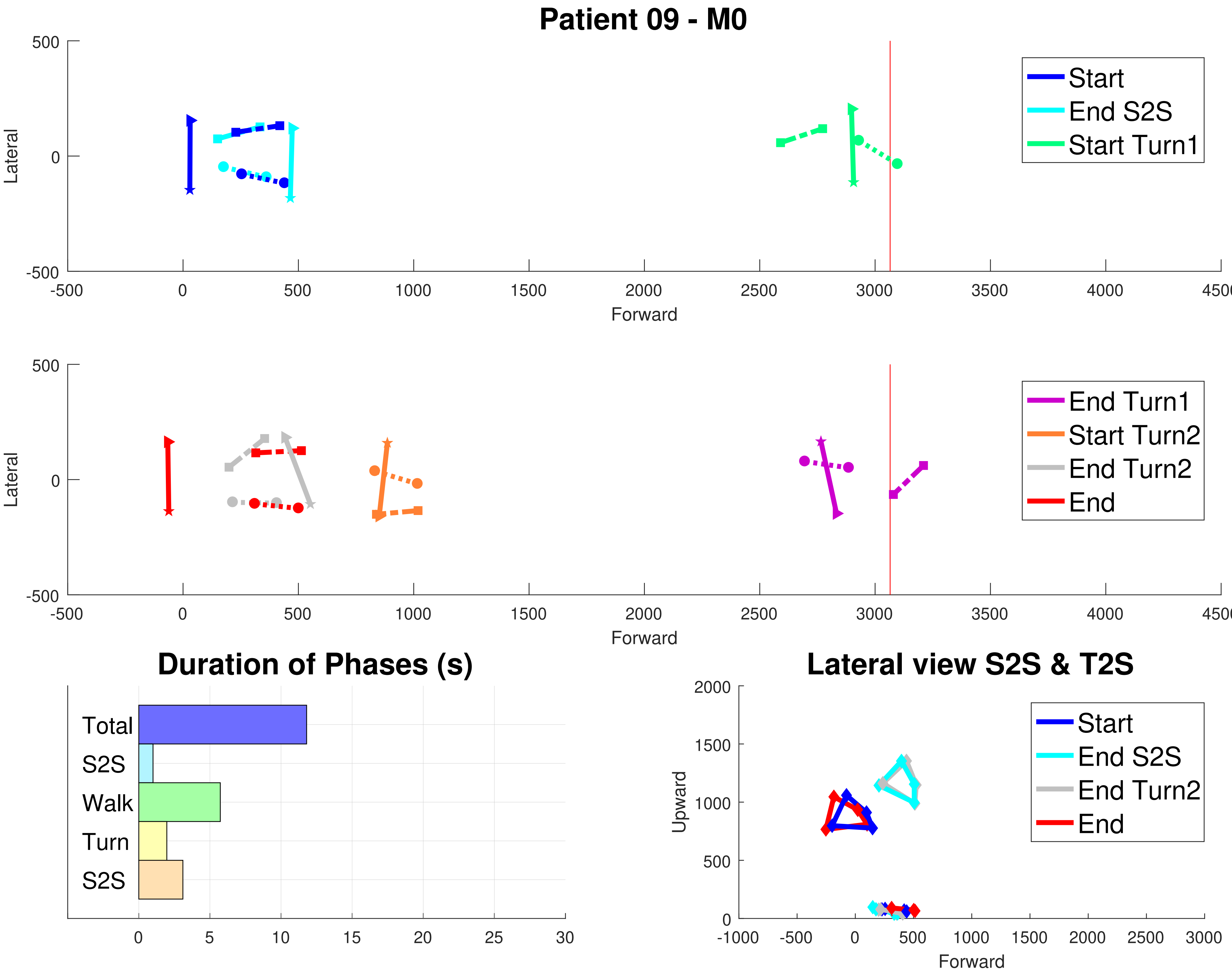
Patient 09 - M0
Lateral
Forward
500
0
-500
-500
0
500
1000
1500
2000
2500
3000
3500
4000
4500
Start
End S2S
Start Turn1
End Turn1
Start Turn2
End Turn2
End
Duration of Phases (s)
Total
S2S
Walk
Turn
S2S
0
5
10
15
20
25
30
Lateral view S2S & T2S
Upward
2000
1500
1000
500
0
-1000
-500
0
500
1000
1500
2000
2500
3000
Start
End S2S
End Turn2
End

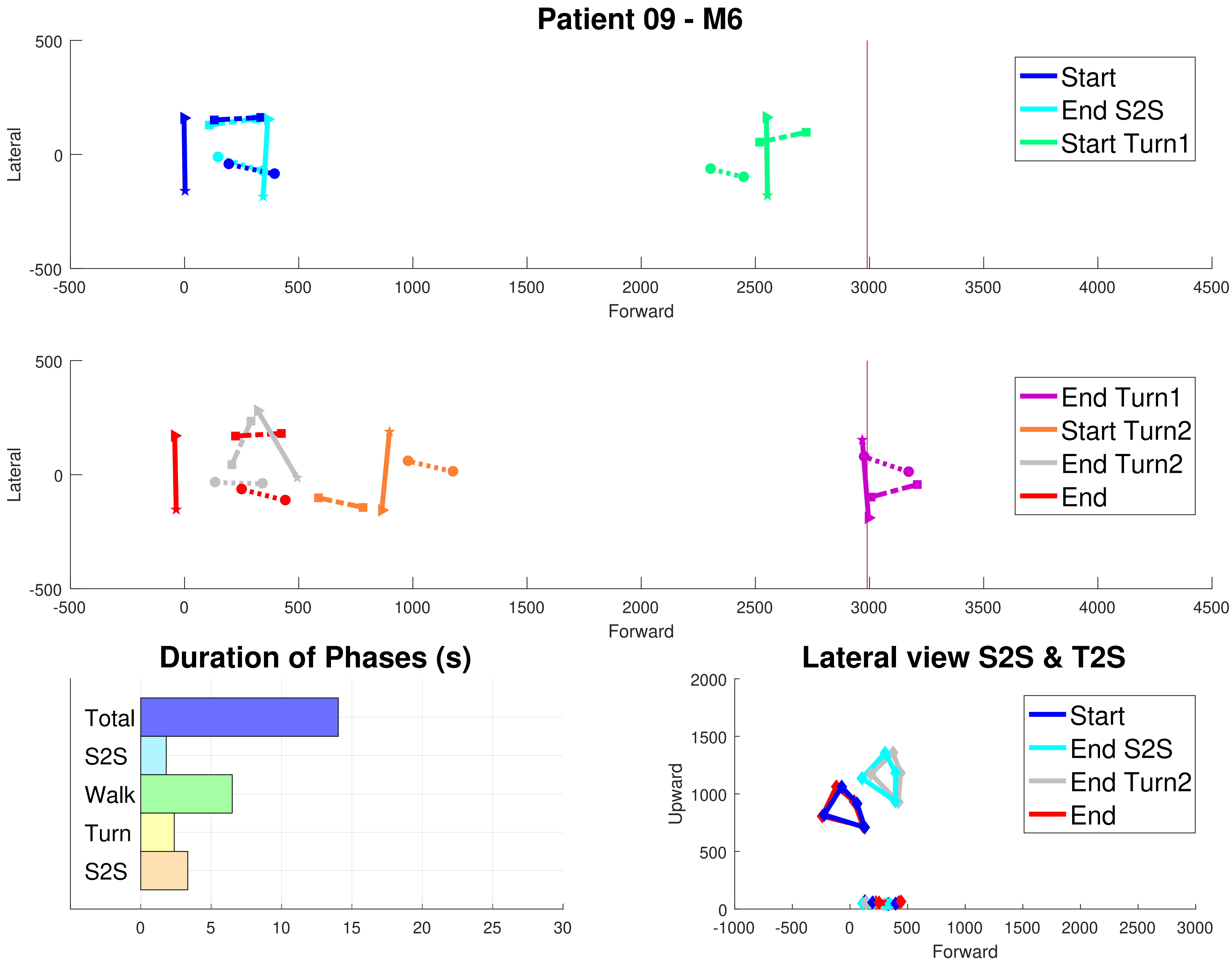

Patient 09 - M6
Lateral
Forward
Start
End S2S
Start Turn1
End Turn1
Start Turn2
End Turn2
End
Duration of Phases (s)
Total
S2S
Walk
Turn
S2S
Lateral view S2S & T2S
Upward
Start
End S2S
End Turn2
End

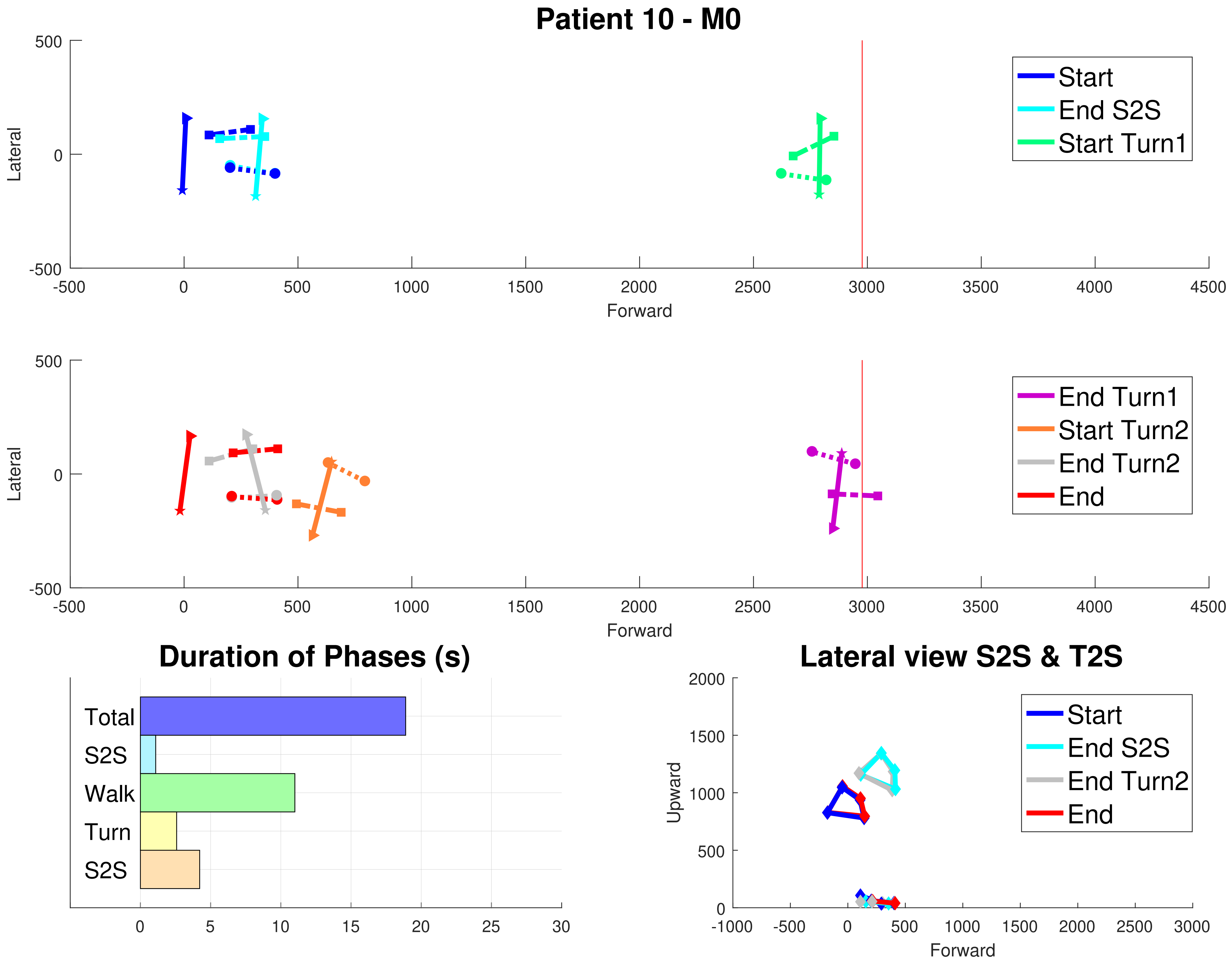

Patient 10 - M0
Lateral
Forward
Start
End S2S
Start Turn1
End Turn1
Start Turn2
End Turn2
End
Duration of Phases (s)
Total
S2S
Walk
Turn
S2S
Lateral view S2S & T2S
Upward
Forward
Start
End S2S
End Turn2
End

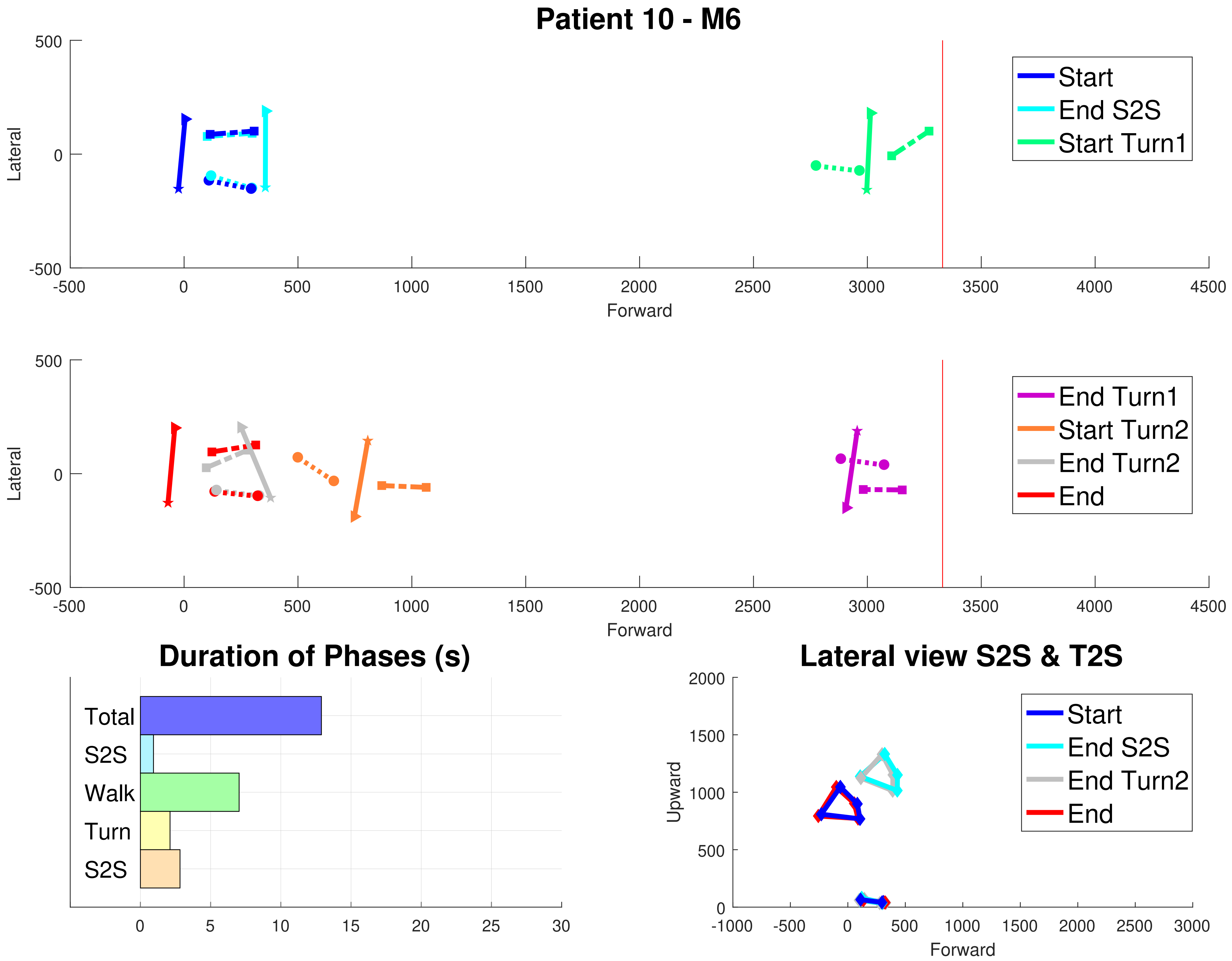

Patient 10 - M6
Lateral
Forward
Start
End S2S
Start Turn1
End Turn1
Start Turn2
End Turn2
End
Duration of Phases (s)
Total
S2S
Walk
Turn
S2S
Lateral view S2S & T2S
Upward
Forward
Start
End S2S
End Turn2
End

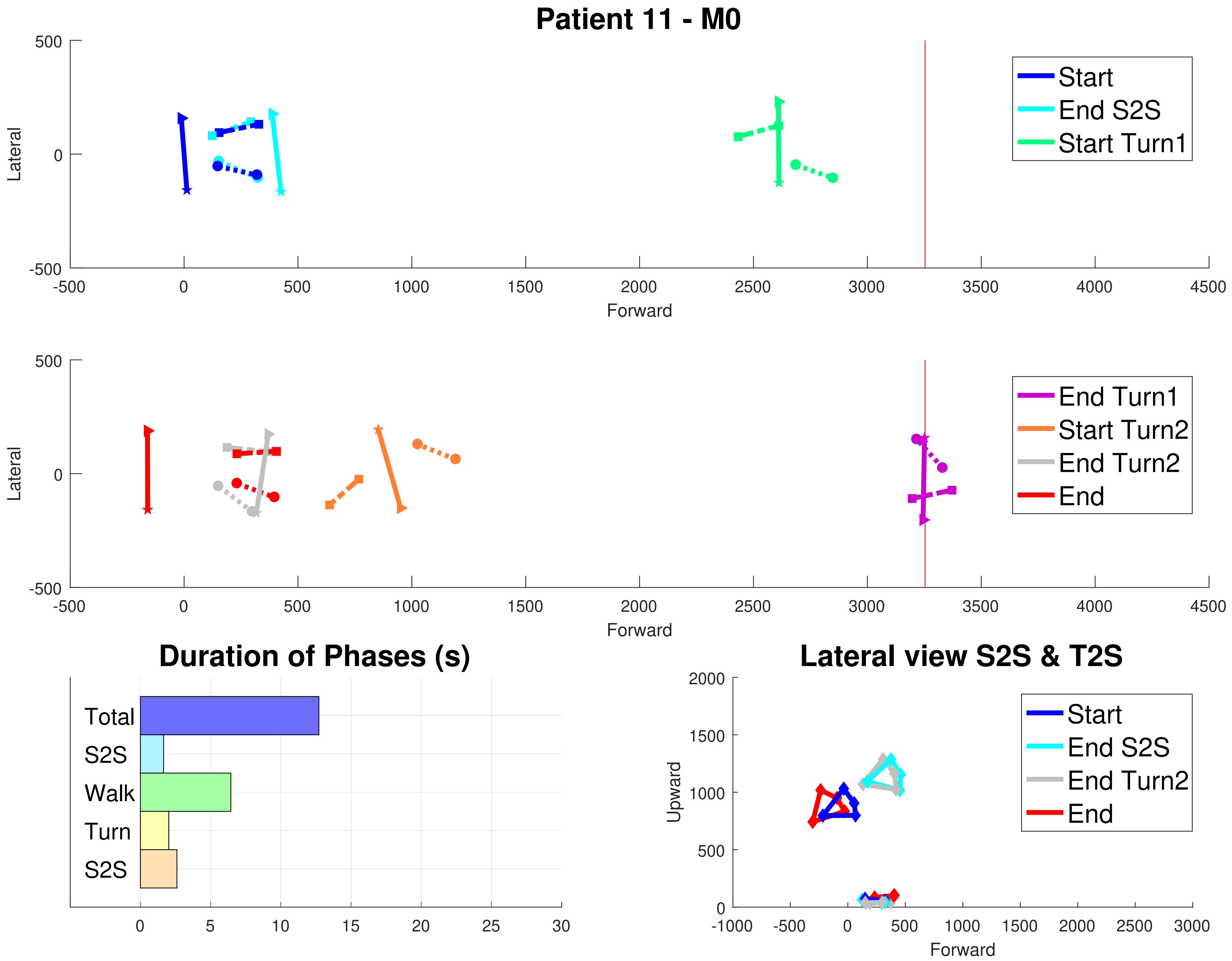
Patient 11 - M0
Lateral
Forward
Start
End S2S
Start Turn1
End Turn1
Start Turn2
End Turn2
End
Duration of Phases (s)
Total
S2S
Walk
Turn
S2S
Lateral view S2S & T2S
Upward
Forward
Start
End S2S
End Turn2
End

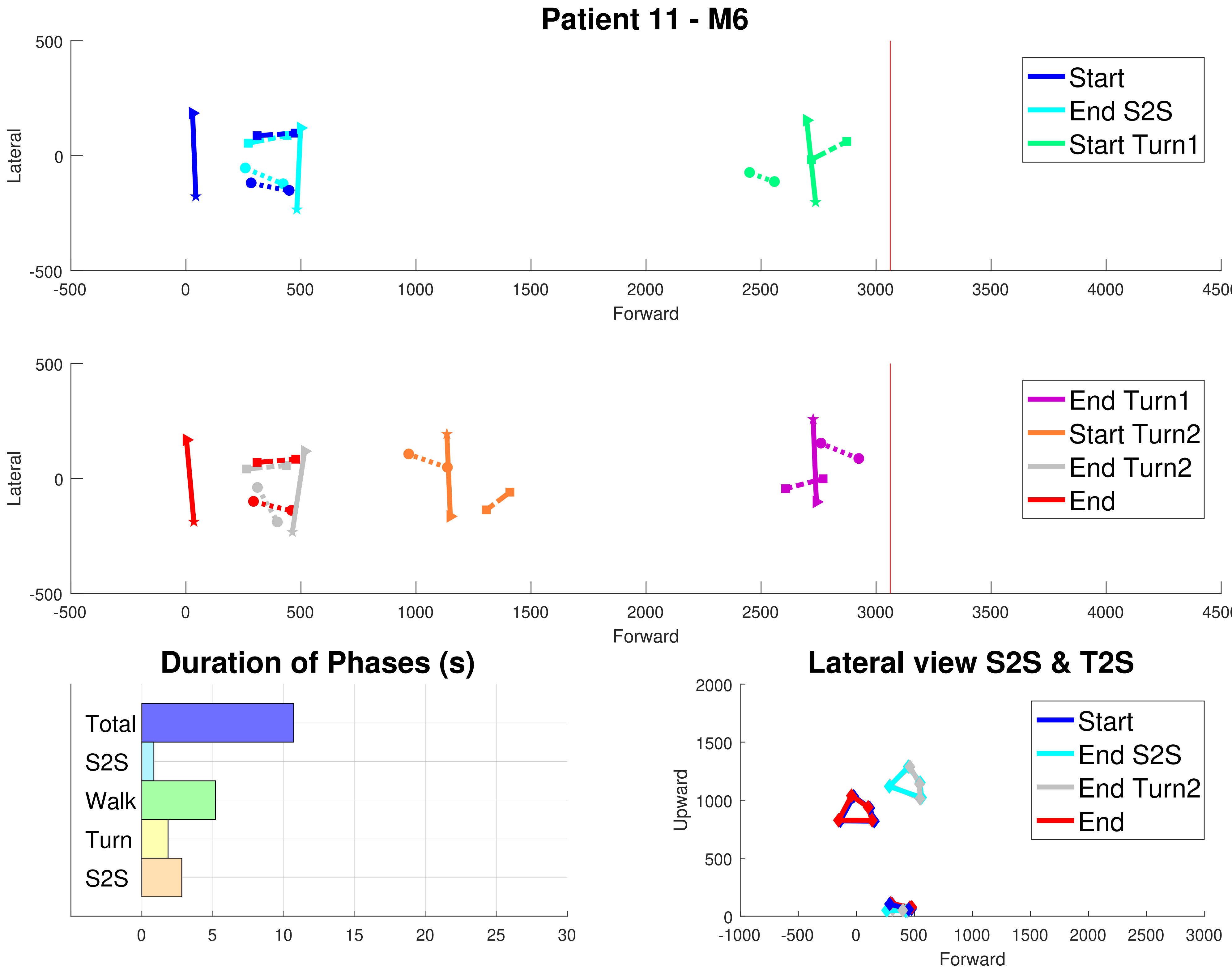
Patient 11 - M6
Lateral
Forward
Start
End S2S
Start Turn1
End Turn1
Start Turn2
End Turn2
End
Duration of Phases (s)
Total
S2S
Walk
Turn
S2S
Lateral view S2S & T2S
Upward
Forward
Start
End S2S
End Turn2
End

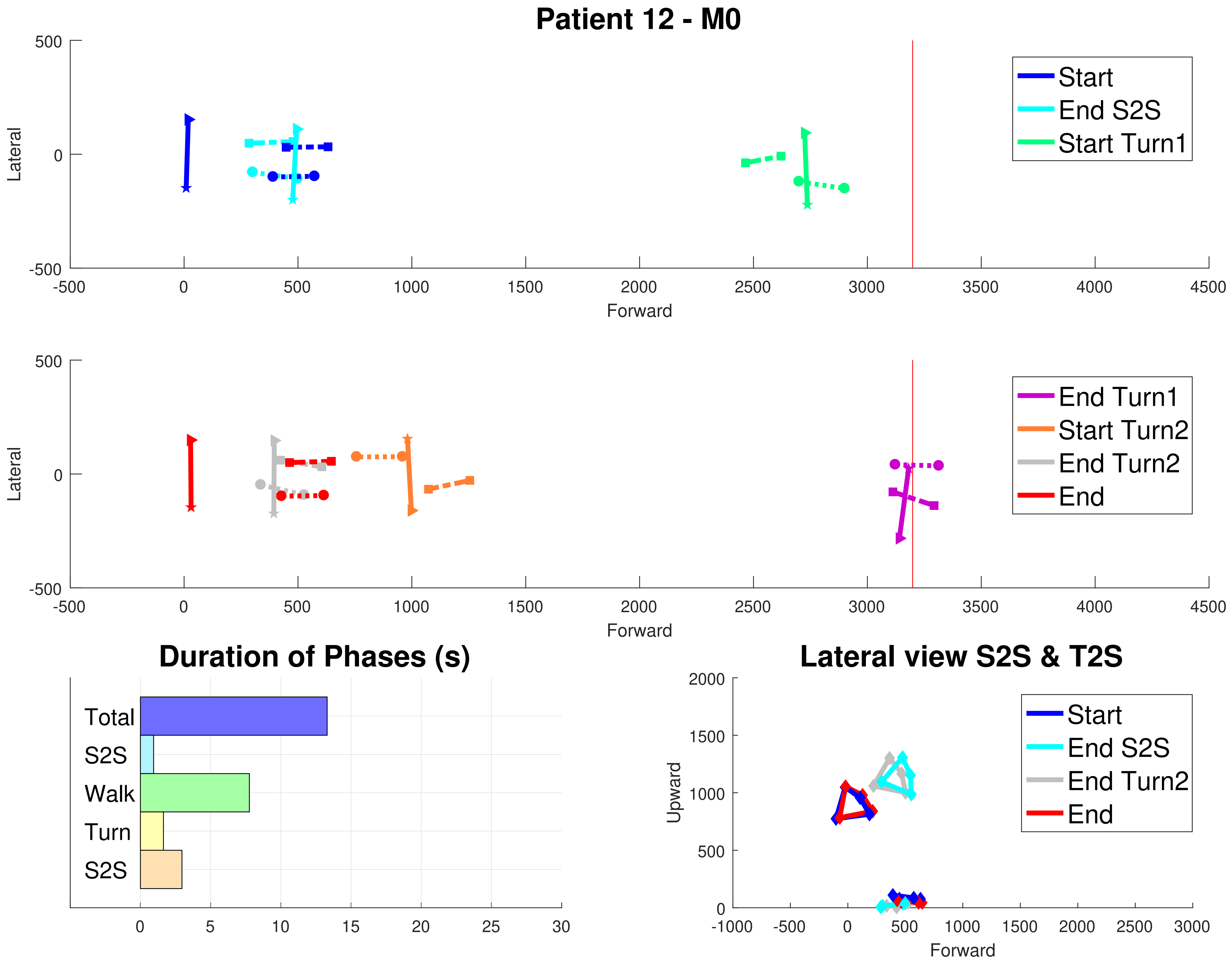

Patient 12 - M0
Lateral
Forward
Start
End S2S
Start Turn1
End Turn1
Start Turn2
End Turn2
End
Duration of Phases (s)
Total
S2S
Walk
Turn
S2S
Lateral view S2S & T2S
Upward
Forward
Start
End S2S
End Turn2
End

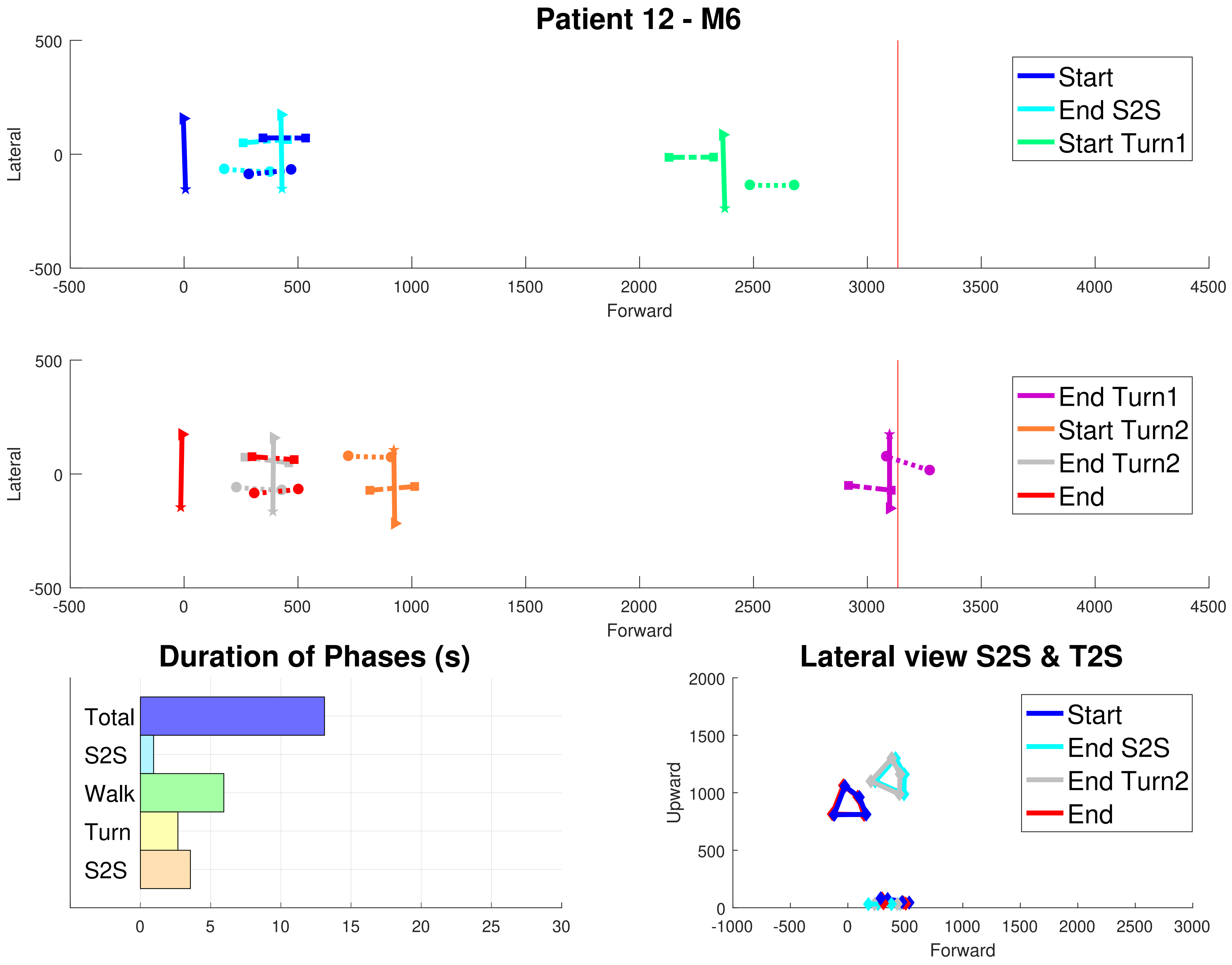

Patient 12 - M6
Lateral
Forward
500
0
-500
-500
0
500
1000
1500
2000
2500
3000
3500
4000
4500
Start
End S2S
Start Turn1
End Turn1
Start Turn2
End Turn2
End
Duration of Phases (s)
Total
S2S
Walk
Turn
S2S
0
5
10
15
20
25
30
Lateral view S2S & T2S
Upward
2000
1500
1000
500
0
-1000
-500
0
500
1000
1500
2000
2500
3000
Start
End S2S
End Turn2
End

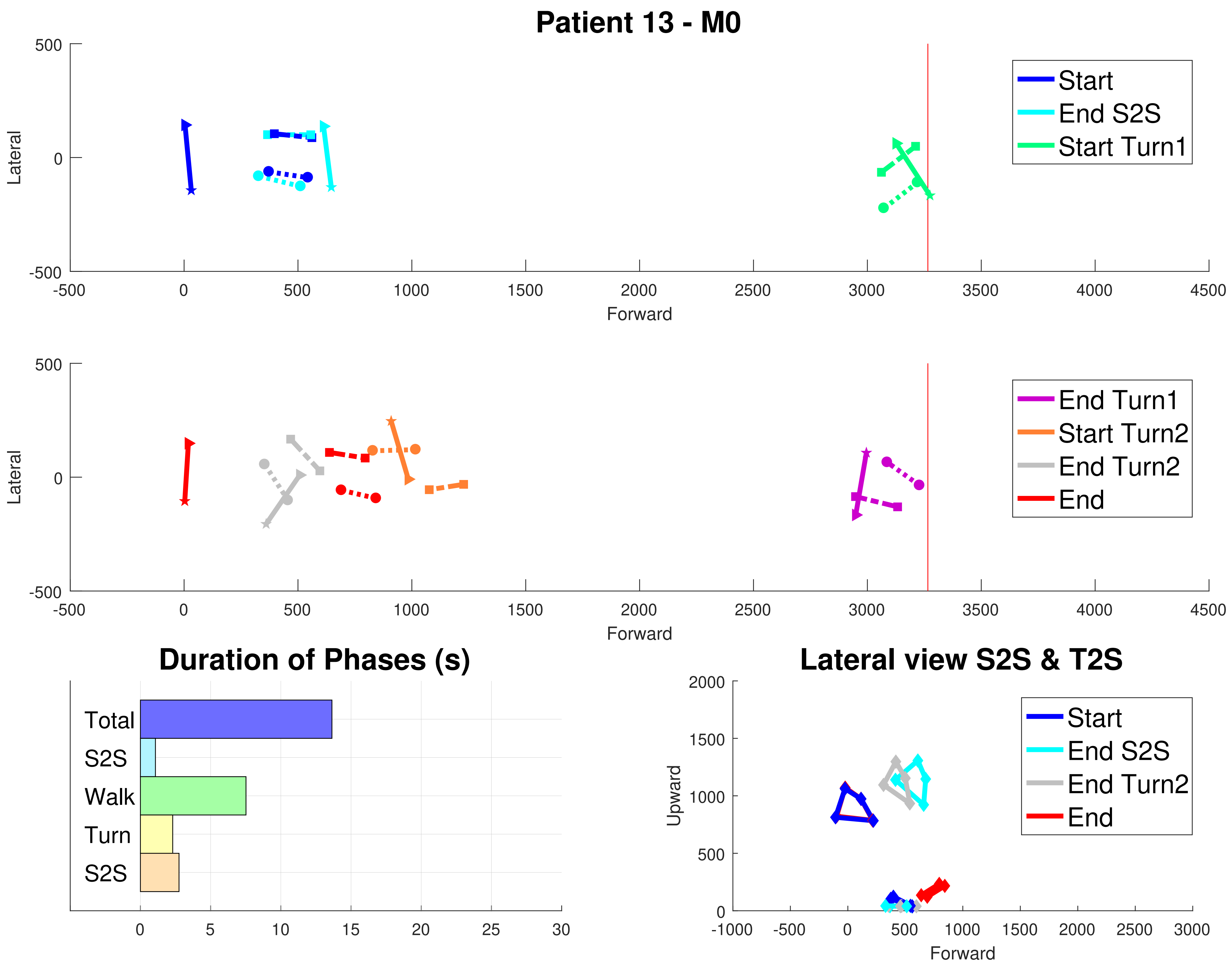
Patient 13 - M0
Lateral
Forward
Start
End S2S
Start Turn1
End Turn1
Start Turn2
End Turn2
End
Duration of Phases (s)
Total
S2S
Walk
Turn
S2S
Lateral view S2S & T2S
Upward
Forward
Start
End S2S
End Turn2
End

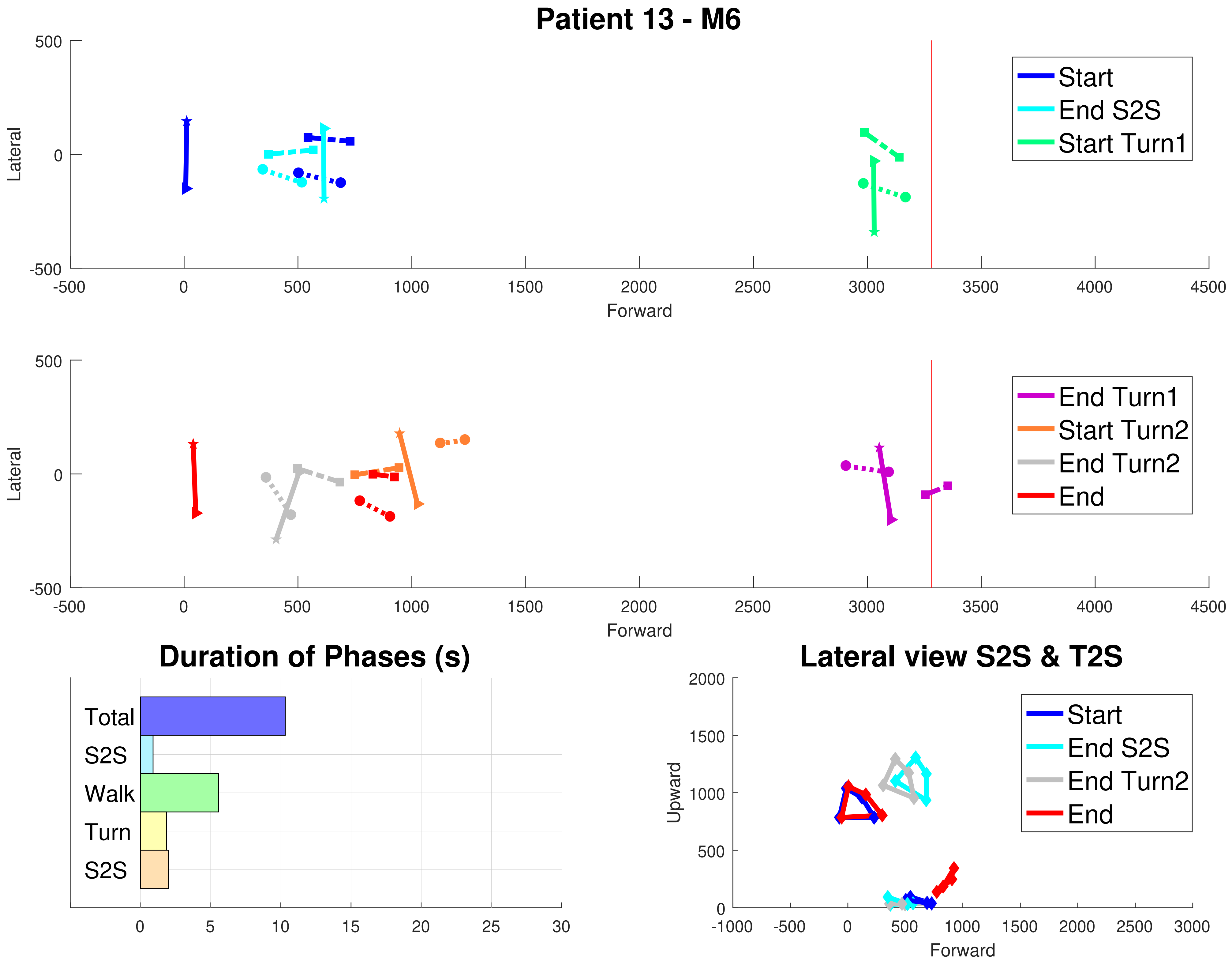

Patient 13 - M6
Lateral
Forward
Start
End S2S
Start Turn1
End Turn1
Start Turn2
End Turn2
End
Duration of Phases (s)
Total
S2S
Walk
Turn
S2S
Lateral view S2S & T2S
Upward
Forward
Start
End S2S
End Turn2
End

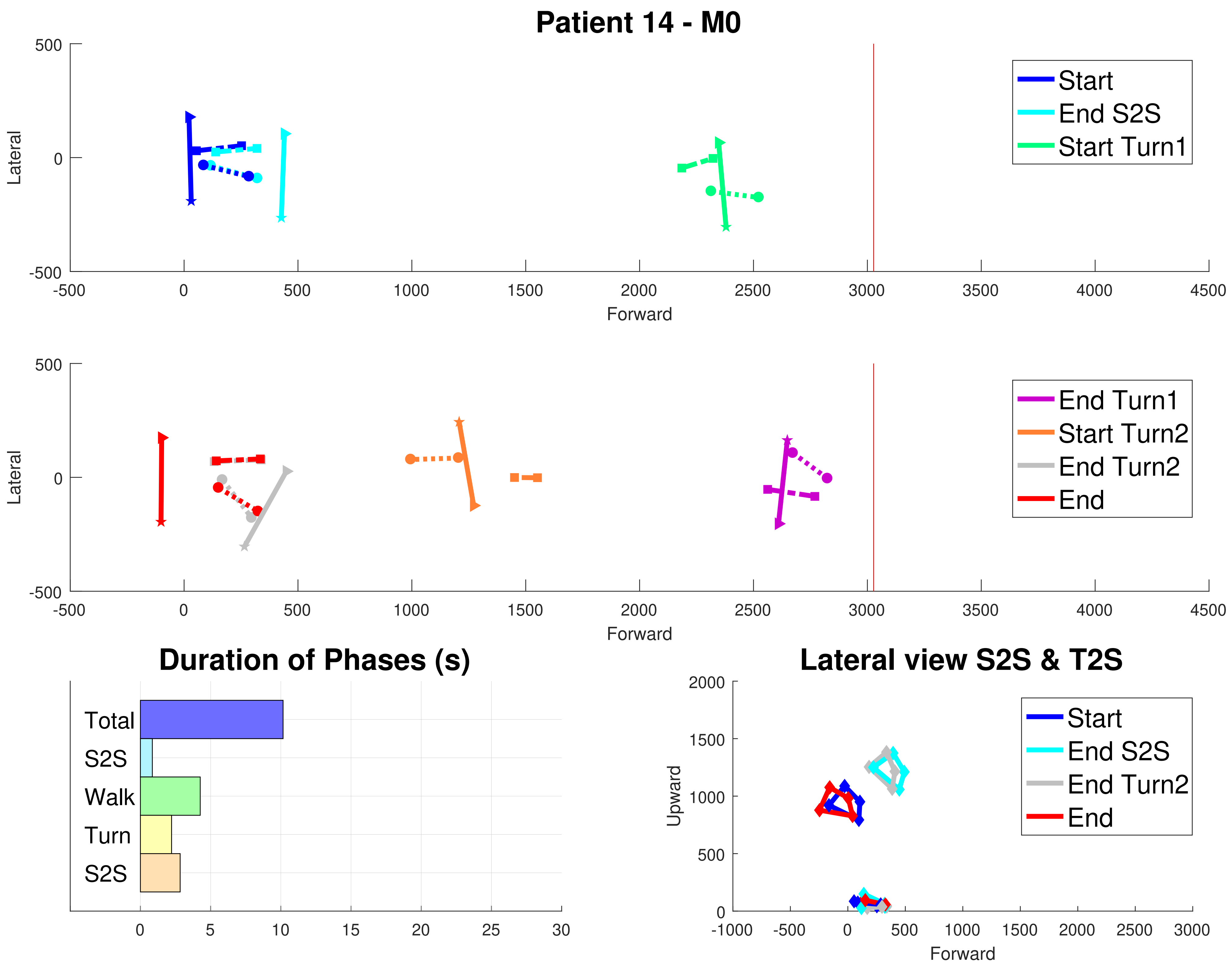
Patient 14 - M0
Lateral
Forward
Start
End S2S
Start Turn1
End Turn1
Start Turn2
End Turn2
End
Duration of Phases (s)
Total
S2S
Walk
Turn
S2S
Lateral view S2S & T2S
Upward
Forward
Start
End S2S
End Turn2
End

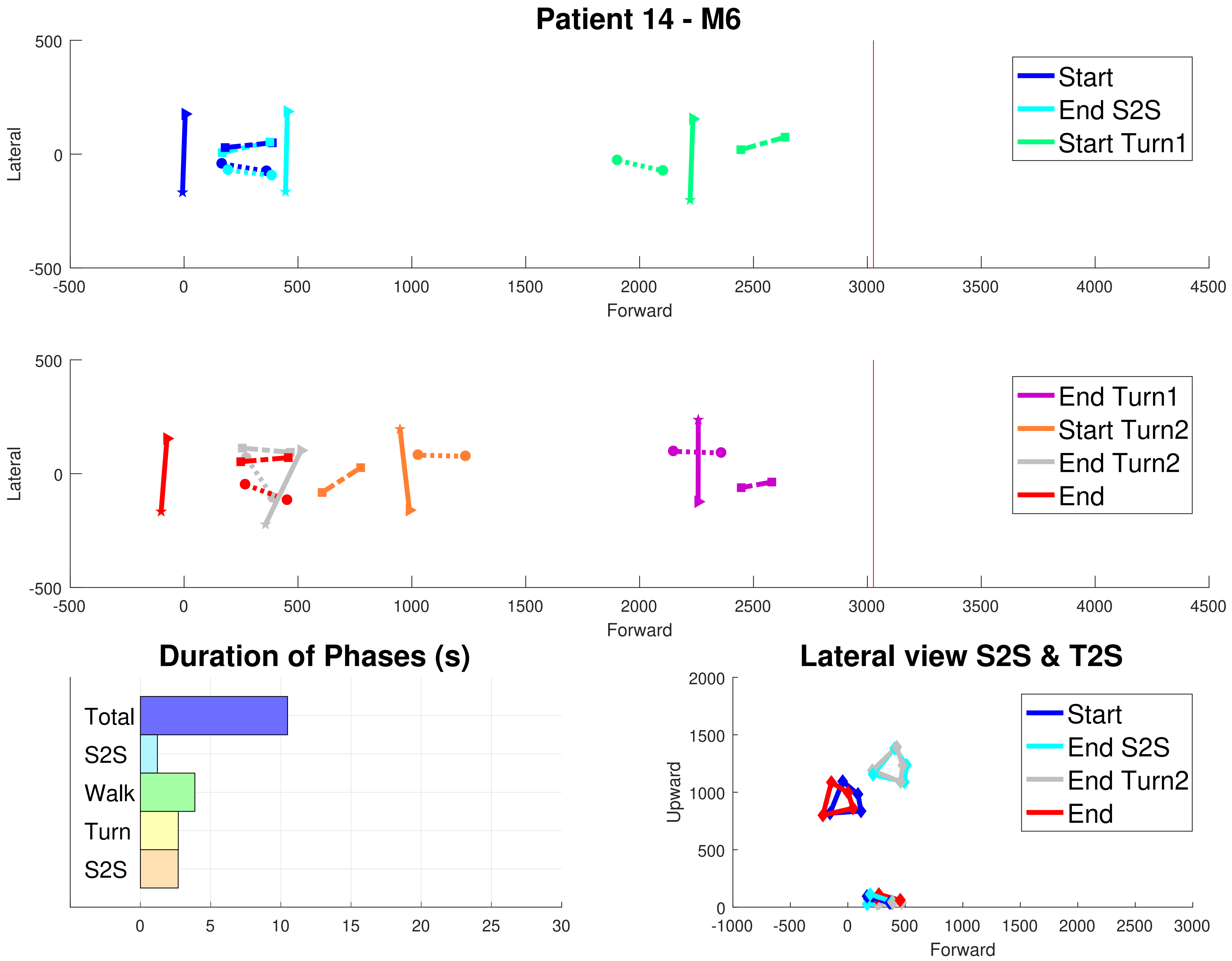

Patient 14 - M6
Lateral
Forward
Start
End S2S
Start Turn1
End Turn1
Start Turn2
End Turn2
End
Duration of Phases (s)
Total
S2S
Walk
Turn
S2S
Lateral view S2S & T2S
Upward
Forward
Start
End S2S
End Turn2
End

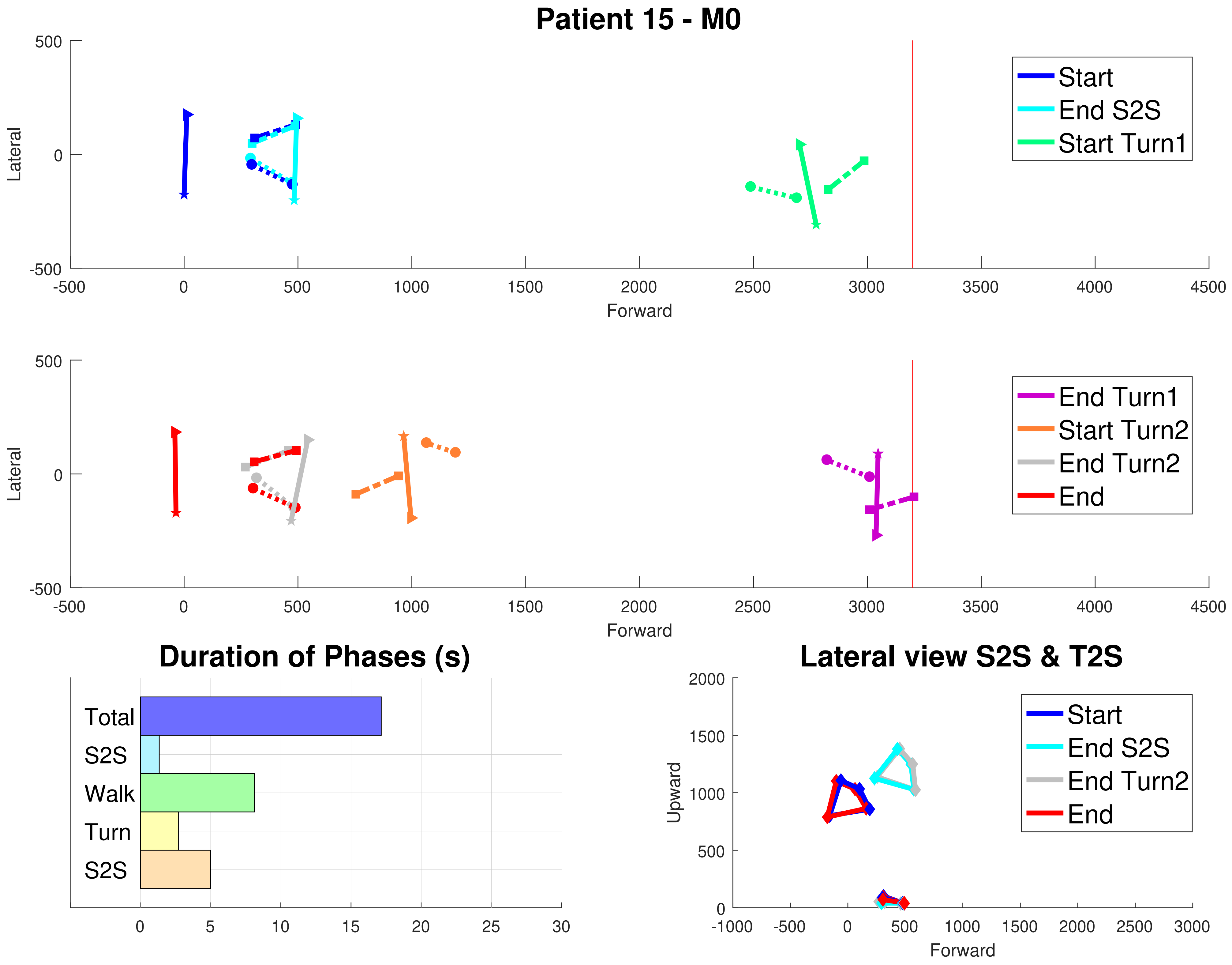
Patient 15 - M0
Lateral
Forward
Start
End S2S
Start Turn1
End Turn1
Start Turn2
End Turn2
End
Duration of Phases (s)
Total
S2S
Walk
Turn
S2S
Lateral view S2S & T2S
Upward
Forward
Start
End S2S
End Turn2
End

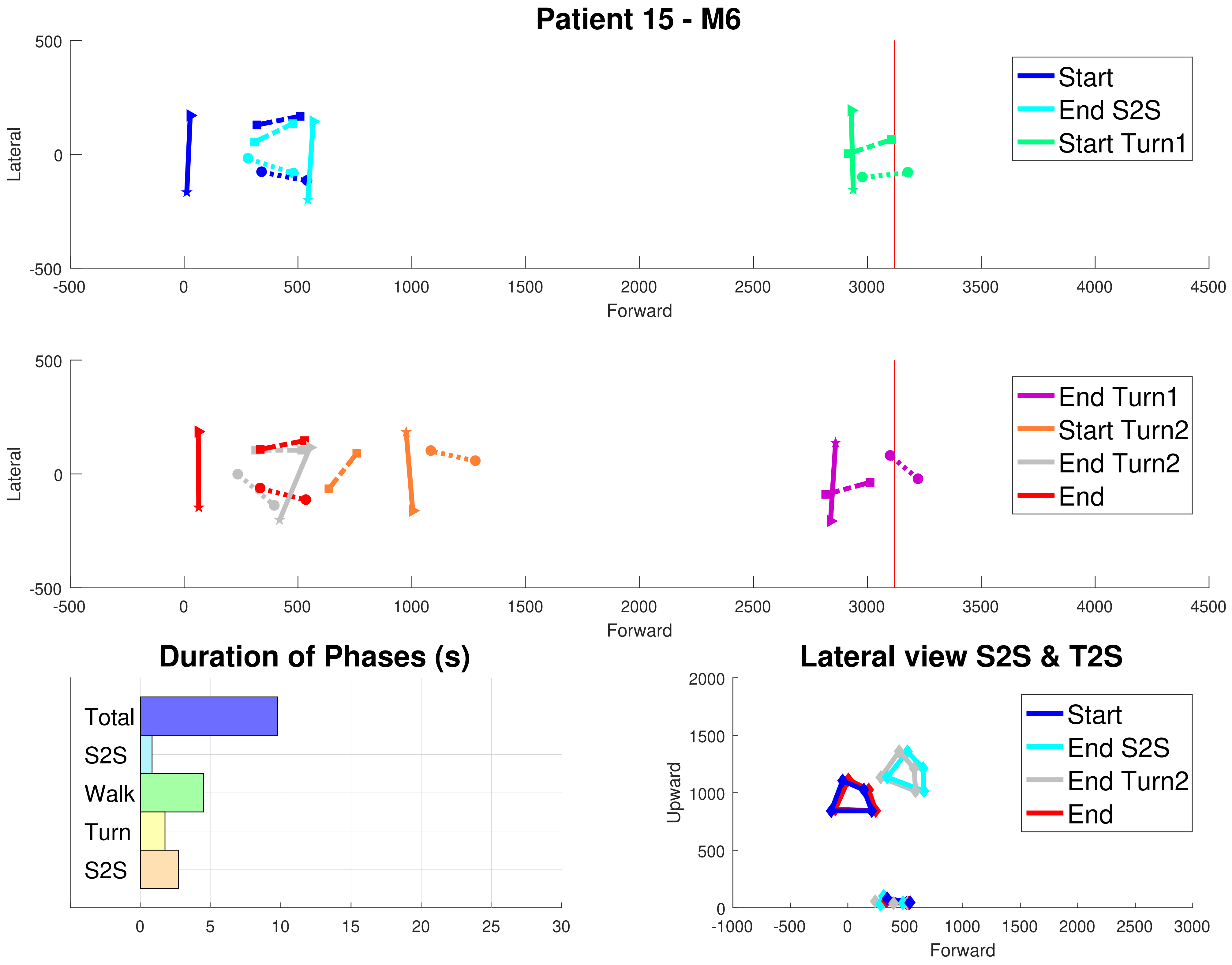

Patient 15 - M6
Lateral
Forward
Start
End S2S
Start Turn1
End Turn1
Start Turn2
End Turn2
End
Duration of Phases (s)
Total
S2S
Walk
Turn
S2S
Lateral view S2S & T2S
Upward
Forward
Start
End S2S
End Turn2
End

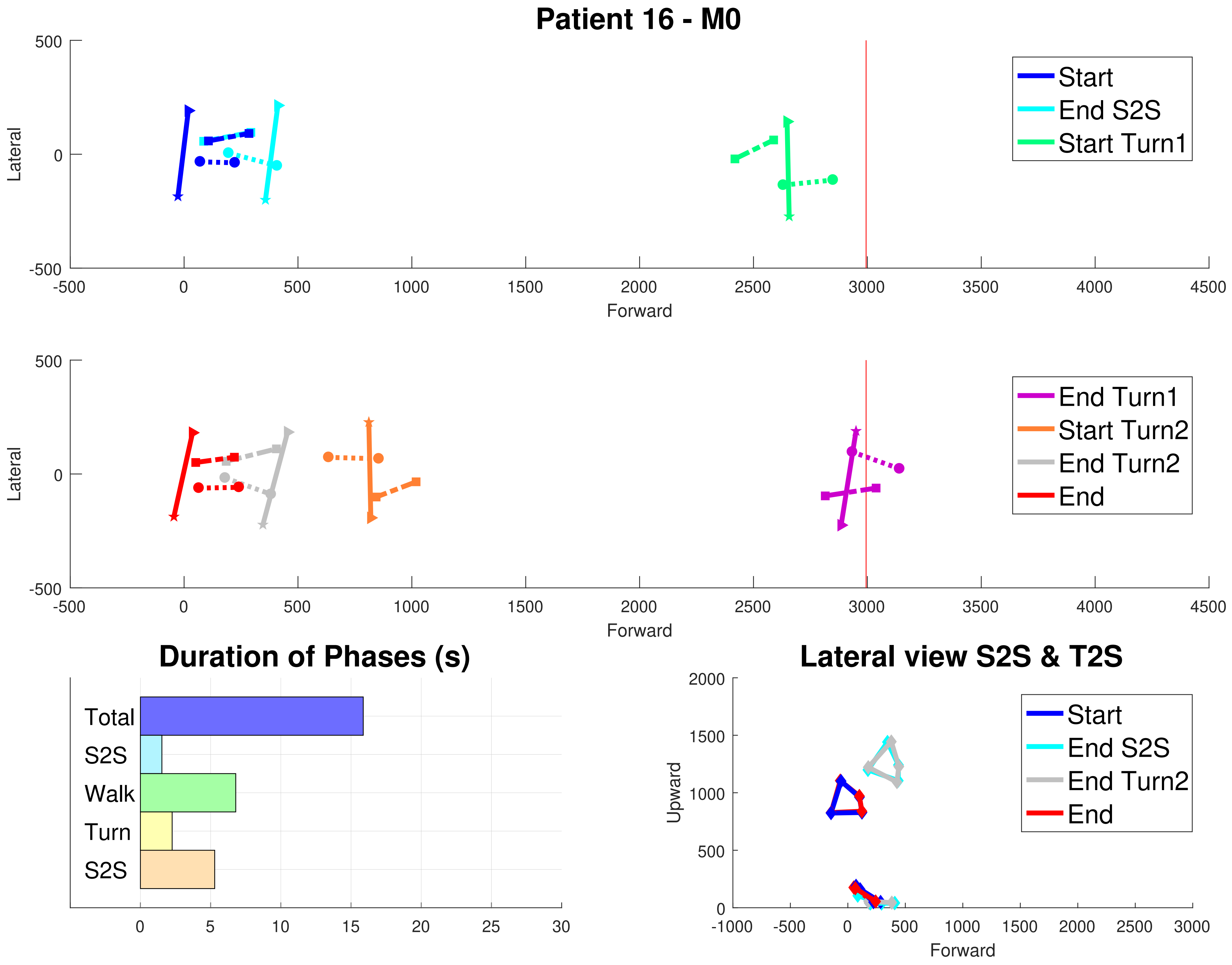
Patient 16 - M0
Lateral
Forward
Start
End S2S
Start Turn1
End Turn1
Start Turn2
End Turn2
End
Duration of Phases (s)
Total
S2S
Walk
Turn
S2S
Lateral view S2S & T2S
Upward
Forward
Start
End S2S
End Turn2
End

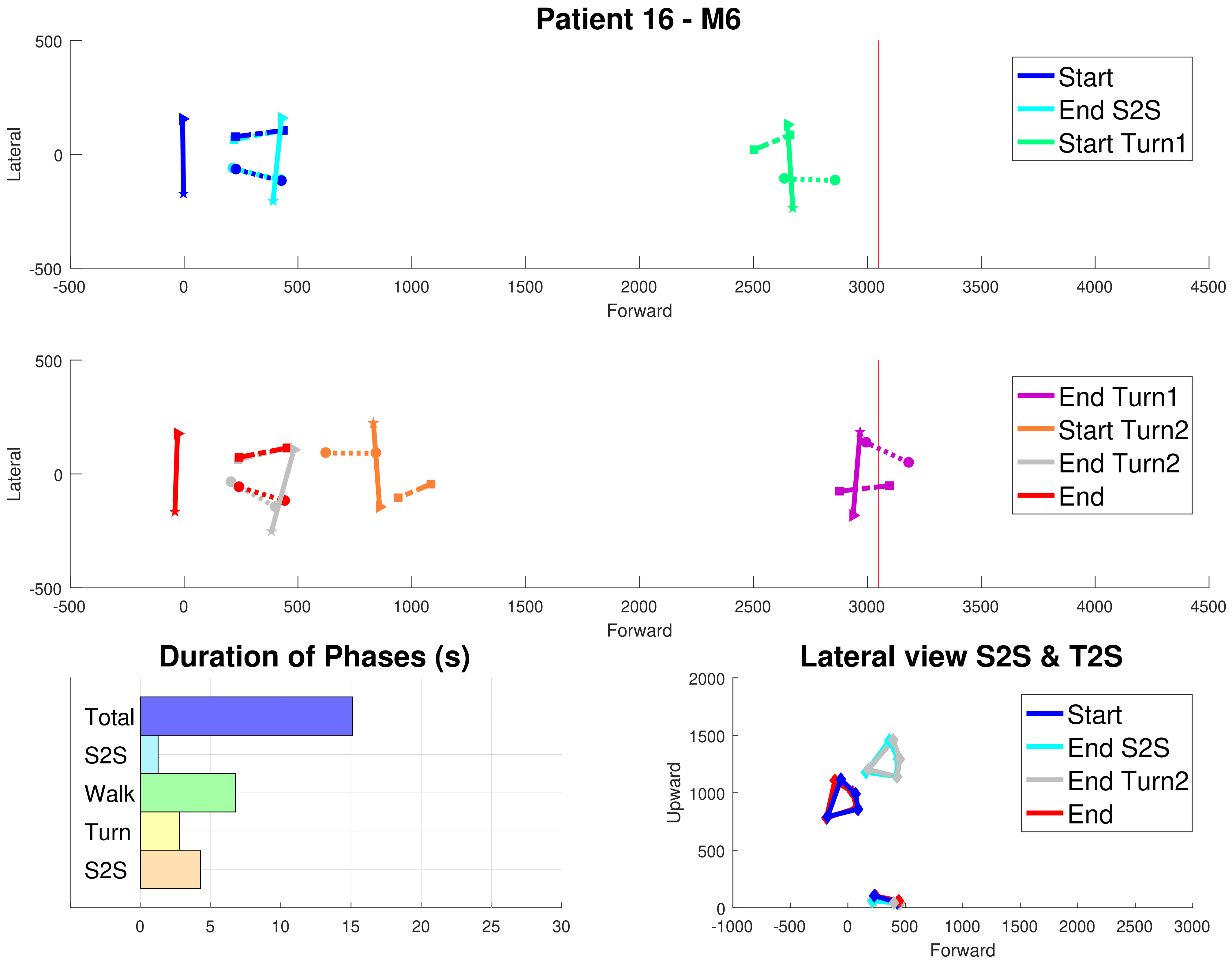

Patient 16 - M6
Lateral
Forward
Start
End S2S
Start Turn1
End Turn1
Start Turn2
End Turn2
End
Duration of Phases (s)
Total
S2S
Walk
Turn
S2S
Lateral view S2S & T2S
Upward

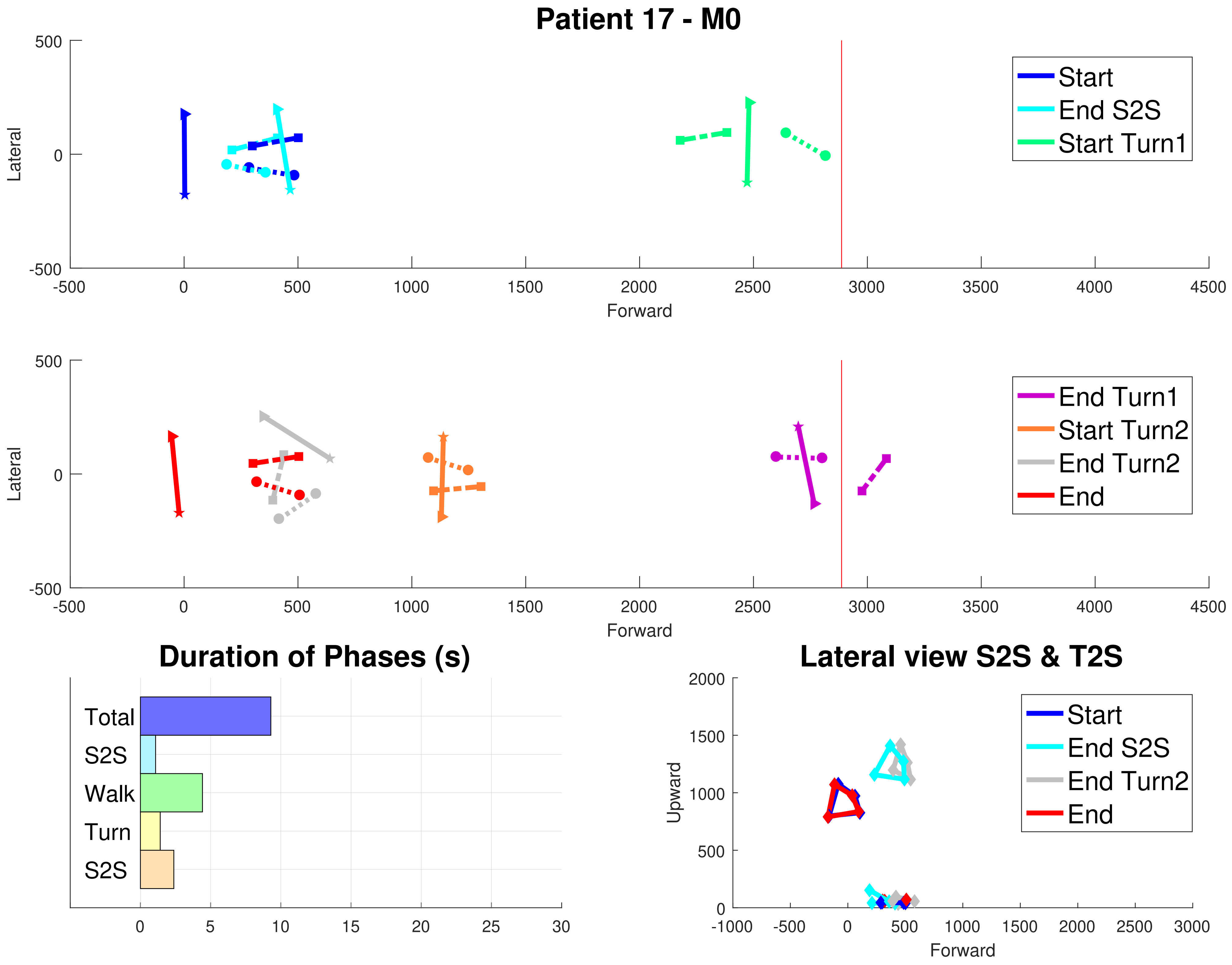
Patient 17 - M0
Lateral
Forward
Start
End S2S
Start Turn1
End Turn1
Start Turn2
End Turn2
End
Duration of Phases (s)
Total
S2S
Walk
Turn
S2S
Lateral view S2S & T2S
Upward
Forward
Start
End S2S
End Turn2
End

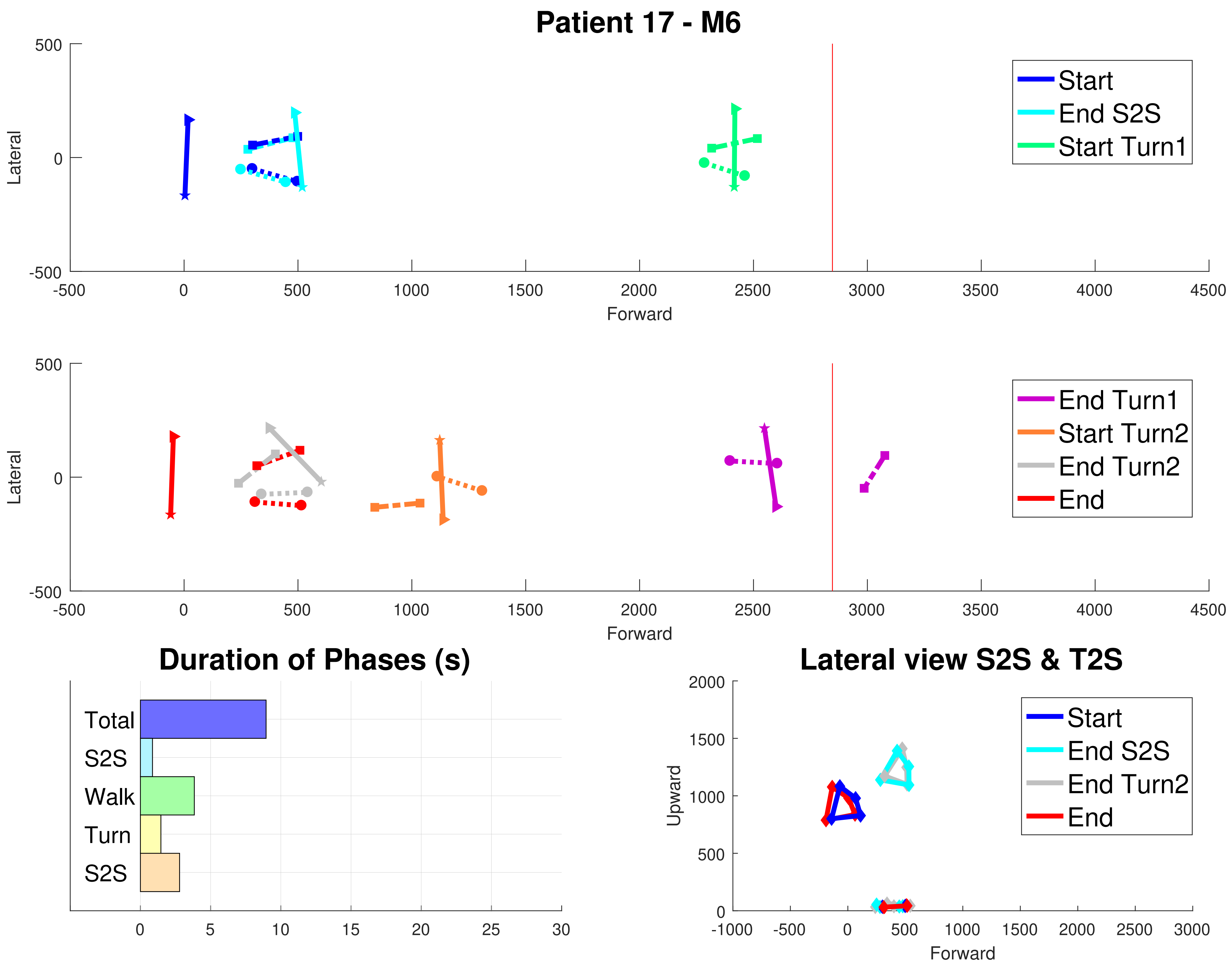
Patient 17 - M6
Lateral
Forward
Start
End S2S
Start Turn1
End Turn1
Start Turn2
End Turn2
End
Duration of Phases (s)
Total
S2S
Walk
Turn
S2S
Lateral view S2S & T2S
Upward
Forward
Start
End S2S
End Turn2
End

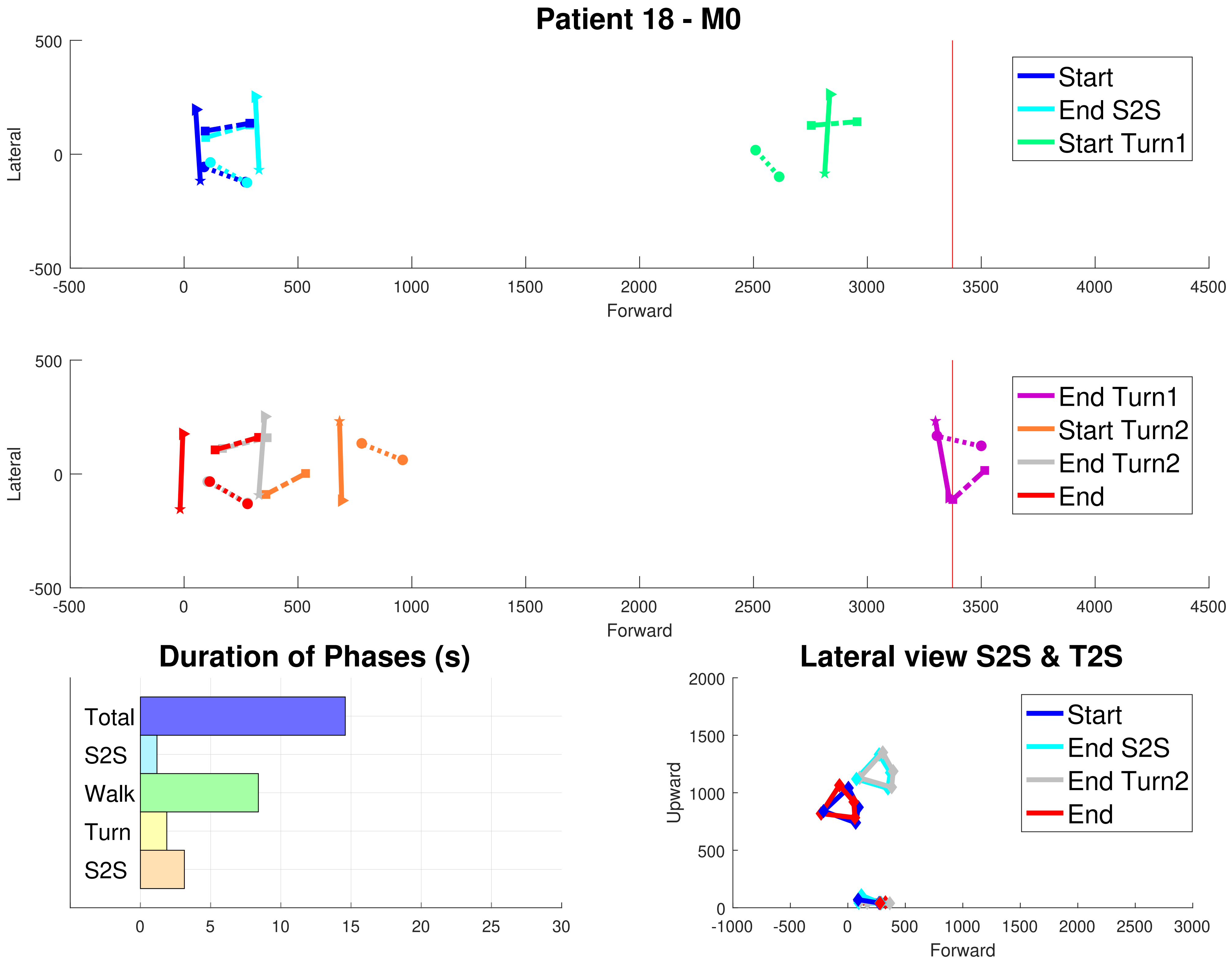
Patient 18 - M0
Lateral
Forward
500
0
-500
-500
0
500
1000
1500
2000
2500
3000
3500
4000
4500
Start
End S2S
Start Turn1
End Turn1
Start Turn2
End Turn2
End
Duration of Phases (s)
Total
S2S
Walk
Turn
S2S
0
5
10
15
20
25
30
Lateral view S2S & T2S
Upward
2000
1500
1000
500
0
-1000
-500
0
500
1000
1500
2000
2500
3000
Forward
Start
End S2S
End Turn2
End

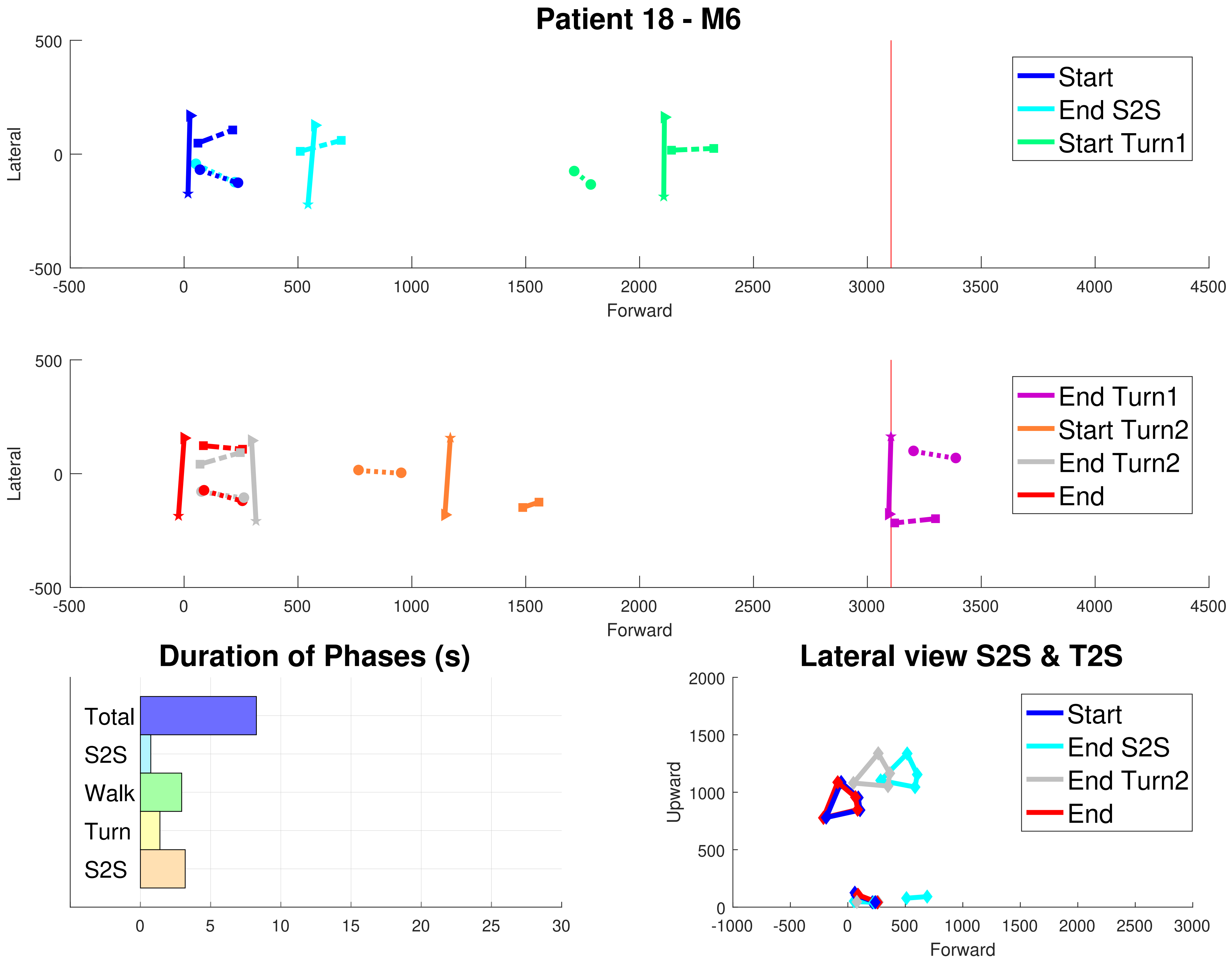

Patient 18 - M6
Lateral
Forward
Start
End S2S
Start Turn1
End Turn1
Start Turn2
End Turn2
End
Duration of Phases (s)
Total
S2S
Walk
Turn
S2S
Lateral view S2S & T2S
Upward
Forward
Start
End S2S
End Turn2
End

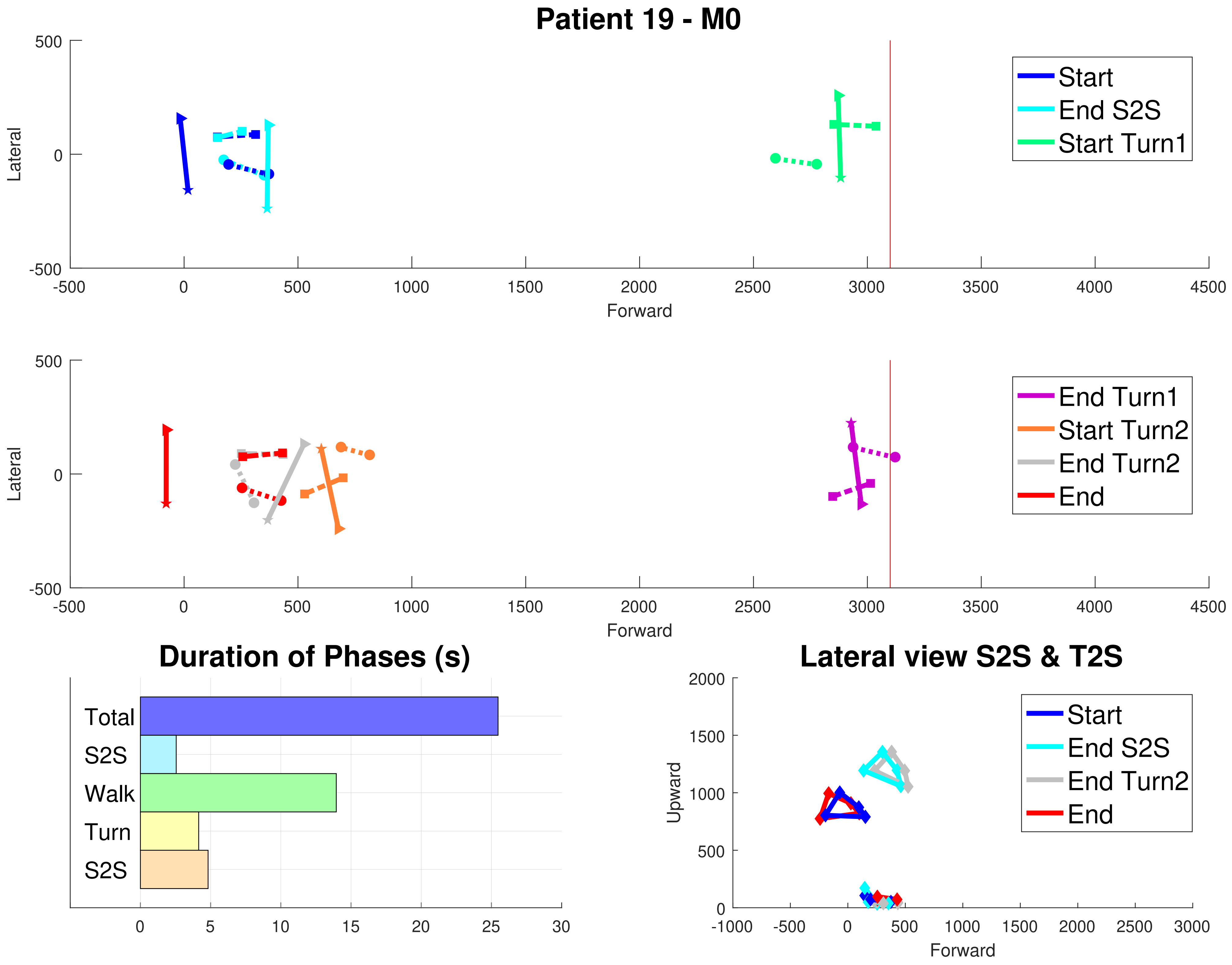
Patient 19 - M0
Lateral
Forward
Start
End S2S
Start Turn1
End Turn1
Start Turn2
End Turn2
End
Duration of Phases (s)
Total
S2S
Walk
Turn
S2S
Lateral view S2S & T2S
Upward
Forward
Start
End S2S
End Turn2
End

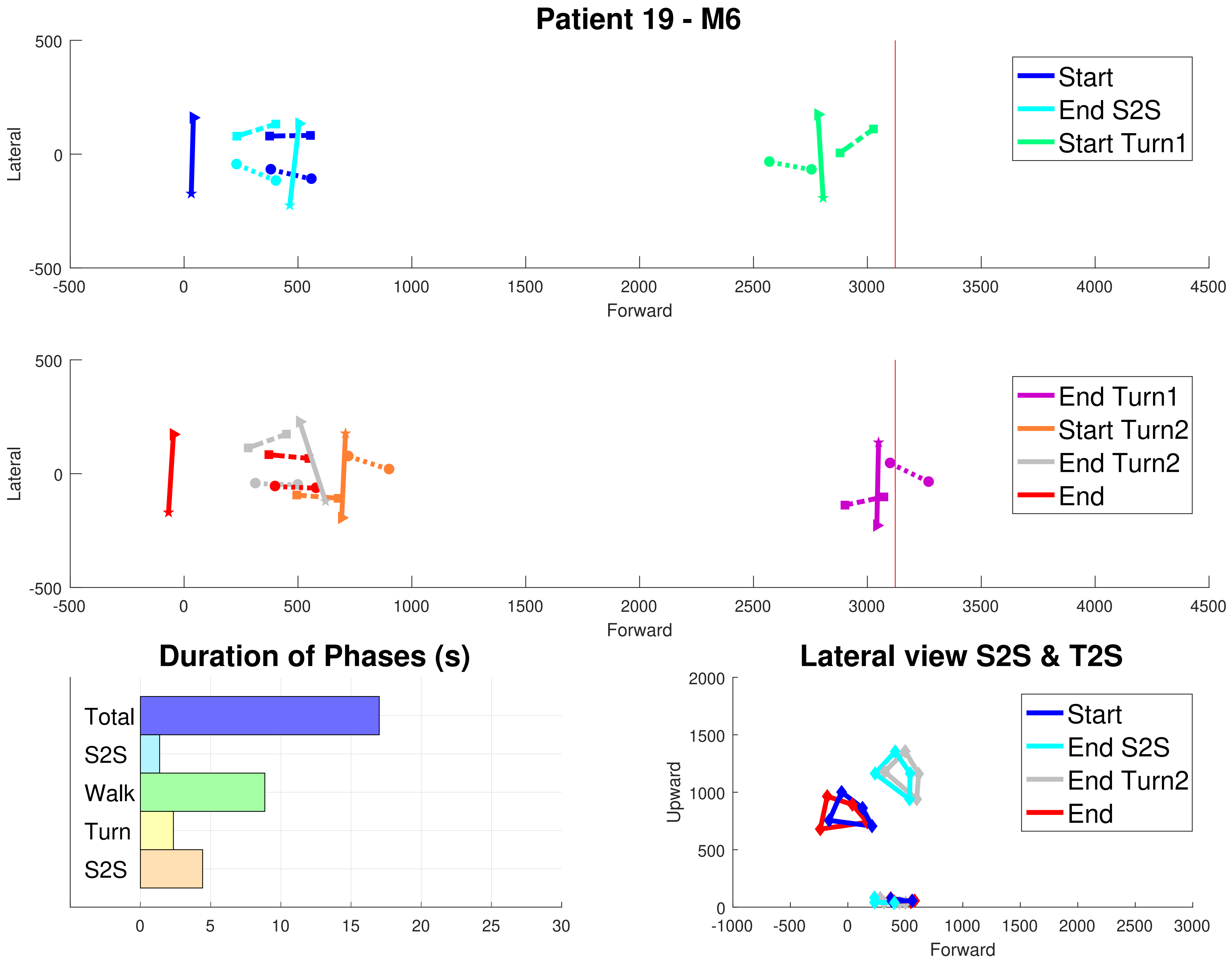
Patient 19 - M6
Lateral
Forward
Start
End S2S
Start Turn1
End Turn1
Start Turn2
End Turn2
End
Duration of Phases (s)
Total
S2S
Walk
Turn
S2S
Lateral view S2S & T2S
Upward
Forward
Start
End S2S
End Turn2
End

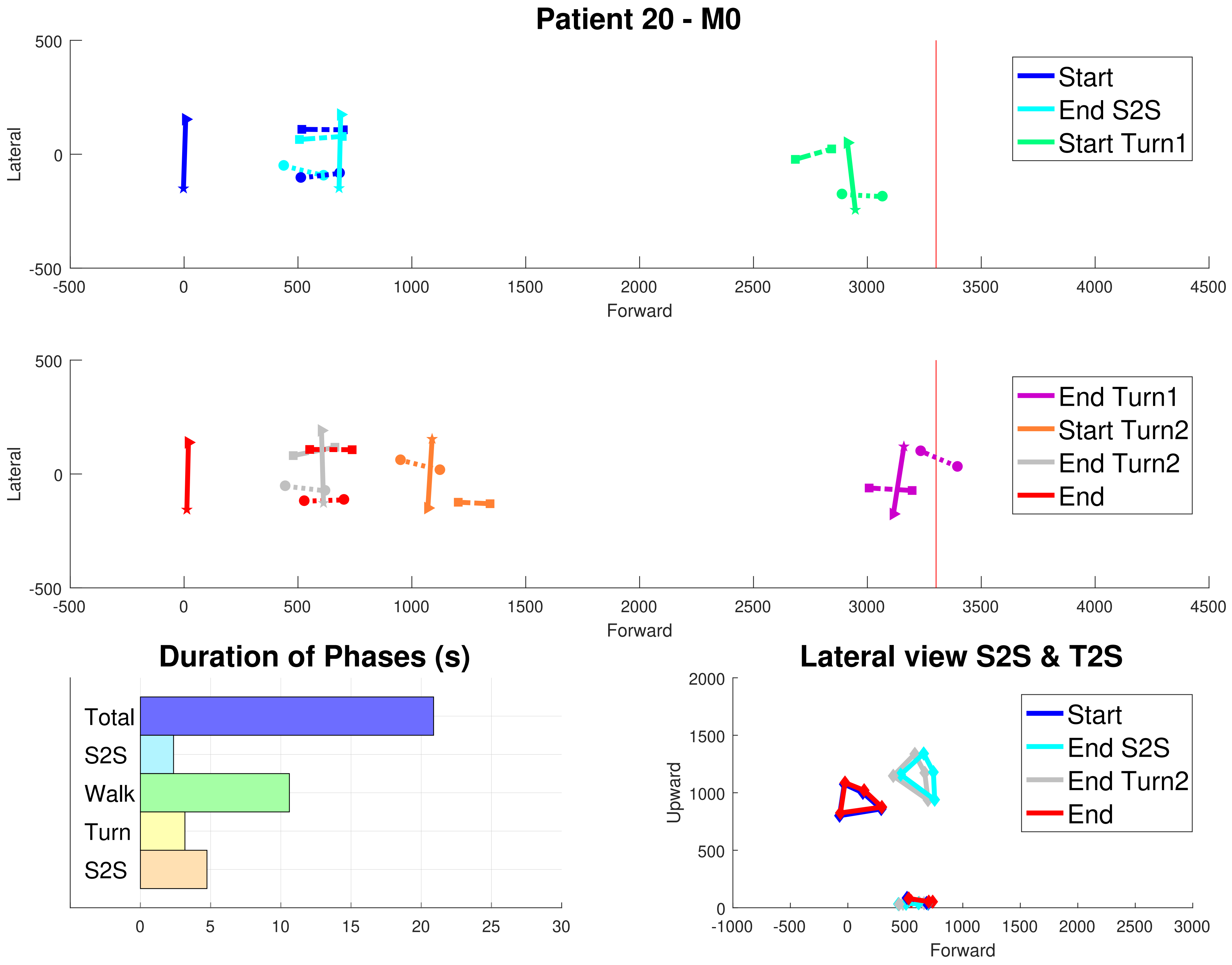

Patient 20 - M0
Lateral
Forward
Start
End S2S
Start Turn1
End Turn1
Start Turn2
End Turn2
End
Duration of Phases (s)
Total
S2S
Walk
Turn
S2S
Lateral view S2S & T2S
Upward
Forward
Start
End S2S
End Turn2
End

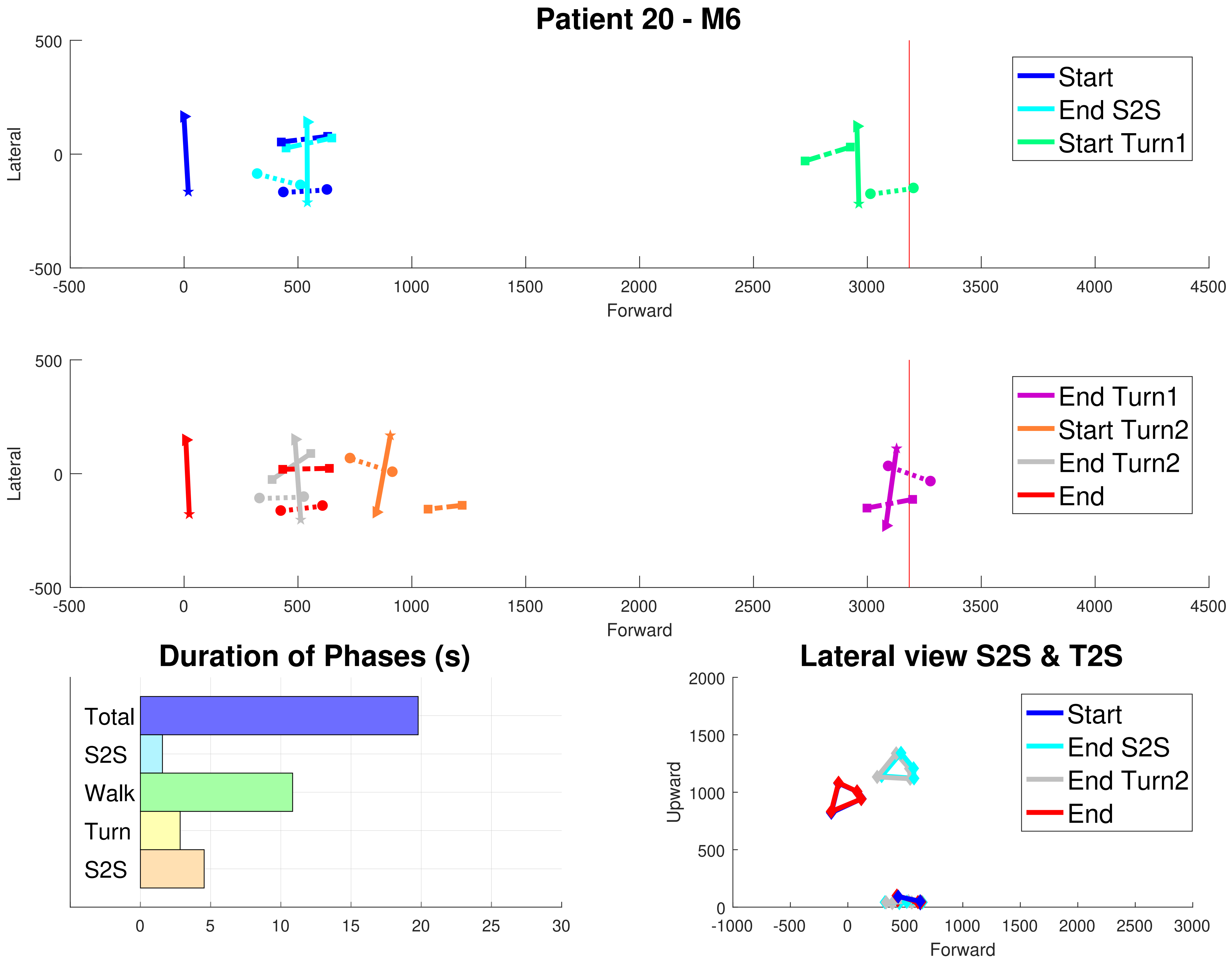

Patient 20 - M6
Lateral
Forward
500
0
-500
-500
0
500
1000
1500
2000
2500
3000
3500
4000
4500
Start
End S2S
Start Turn1
End Turn1
Start Turn2
End Turn2
End
Duration of Phases (s)
Total
S2S
Walk
Turn
S2S
0
5
10
15
20
25
30
Lateral view S2S & T2S
Upward
2000
1500
1000
500
-1000
-500
Start
End S2S
End Turn2
End

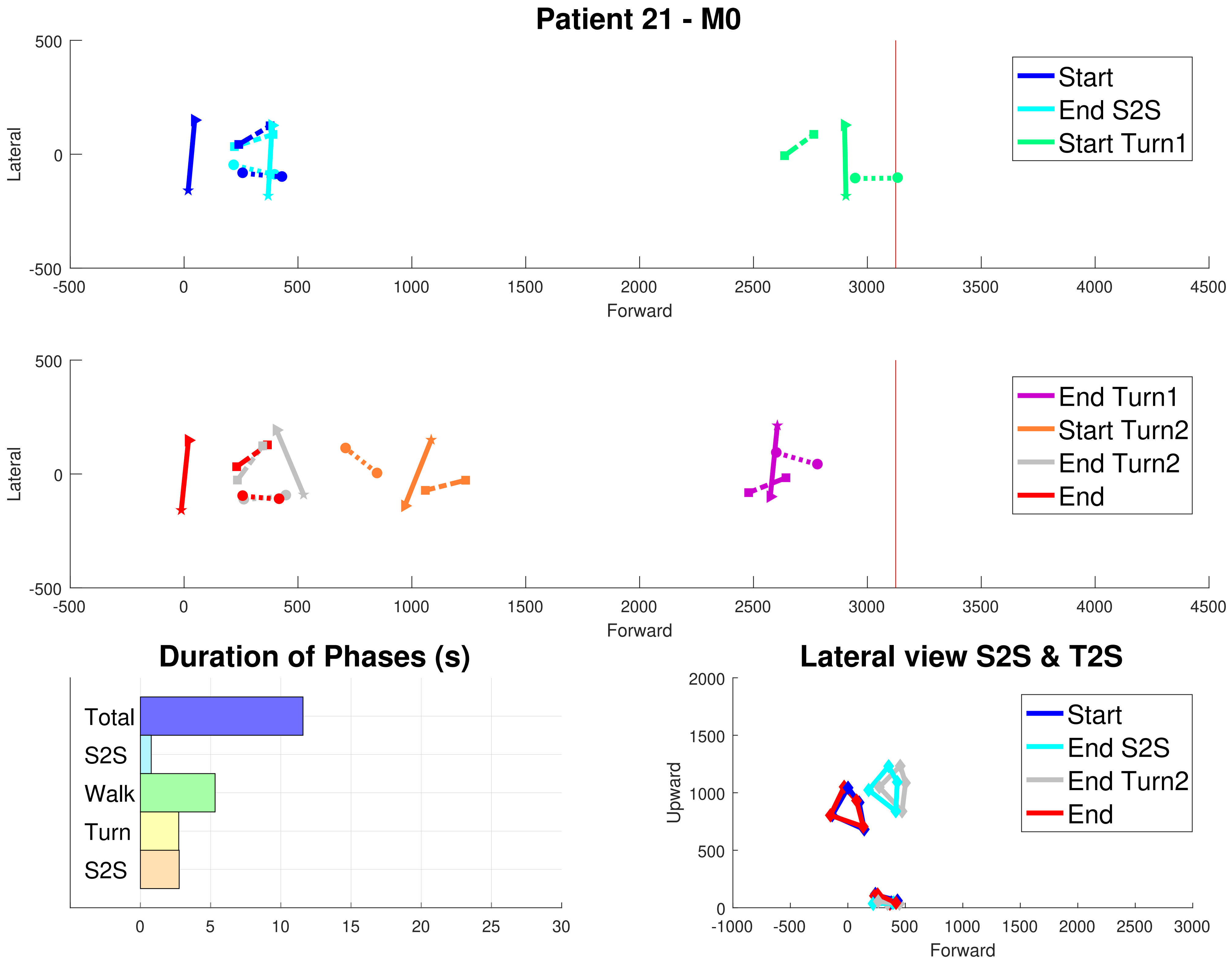

Patient 21 - M0
Lateral
Forward
Start
End S2S
Start Turn1
End Turn1
Start Turn2
End Turn2
End
Duration of Phases (s)
Total
S2S
Walk
Turn
S2S
Lateral view S2S & T2S
Upward
Forward
Start
End S2S
End Turn2
End

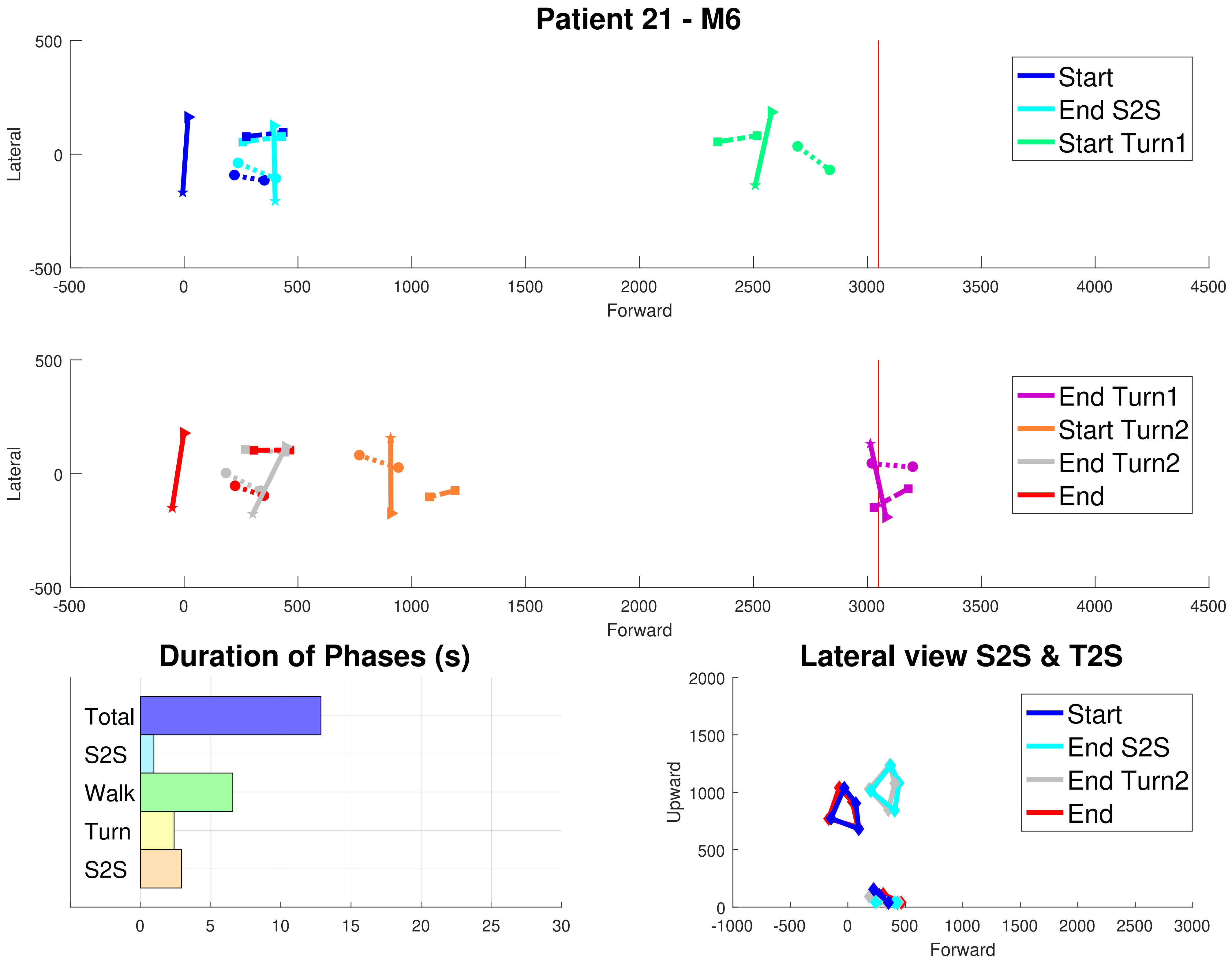

Patient 21 - M6
Lateral
Forward
Start
End S2S
Start Turn1
End Turn1
Start Turn2
End Turn2
End
Duration of Phases (s)
Total
S2S
Walk
Turn
S2S
Lateral view S2S & T2S
Upward
Forward
Start
End S2S
End Turn2
End

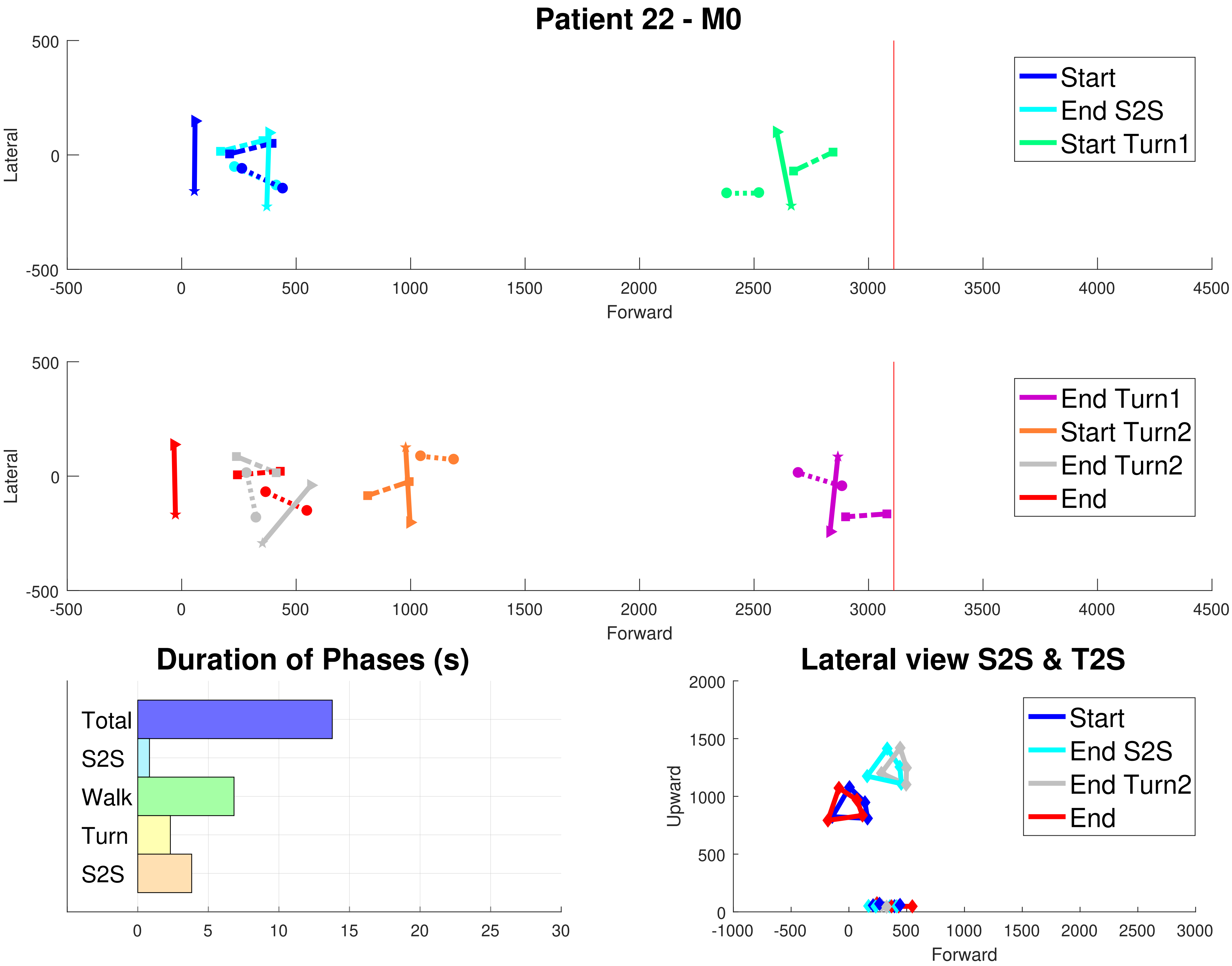
Patient 22 - M0
Lateral
Forward
Start
End S2S
Start Turn1
End Turn1
Start Turn2
End Turn2
End
500
0
-500
-500
0
500
1000
1500
2000
2500
3000
3500
4000
4500
Duration of Phases (s)
Total
S2S
Walk
Turn
S2S
0
5
10
15
20
25
30
Lateral view S2S & T2S
Upward
2000
1500
1000
500
0
-1000
-500
0
500
1000
1500
2000
2500
3000
Forward
Start
End S2S
End Turn2
End

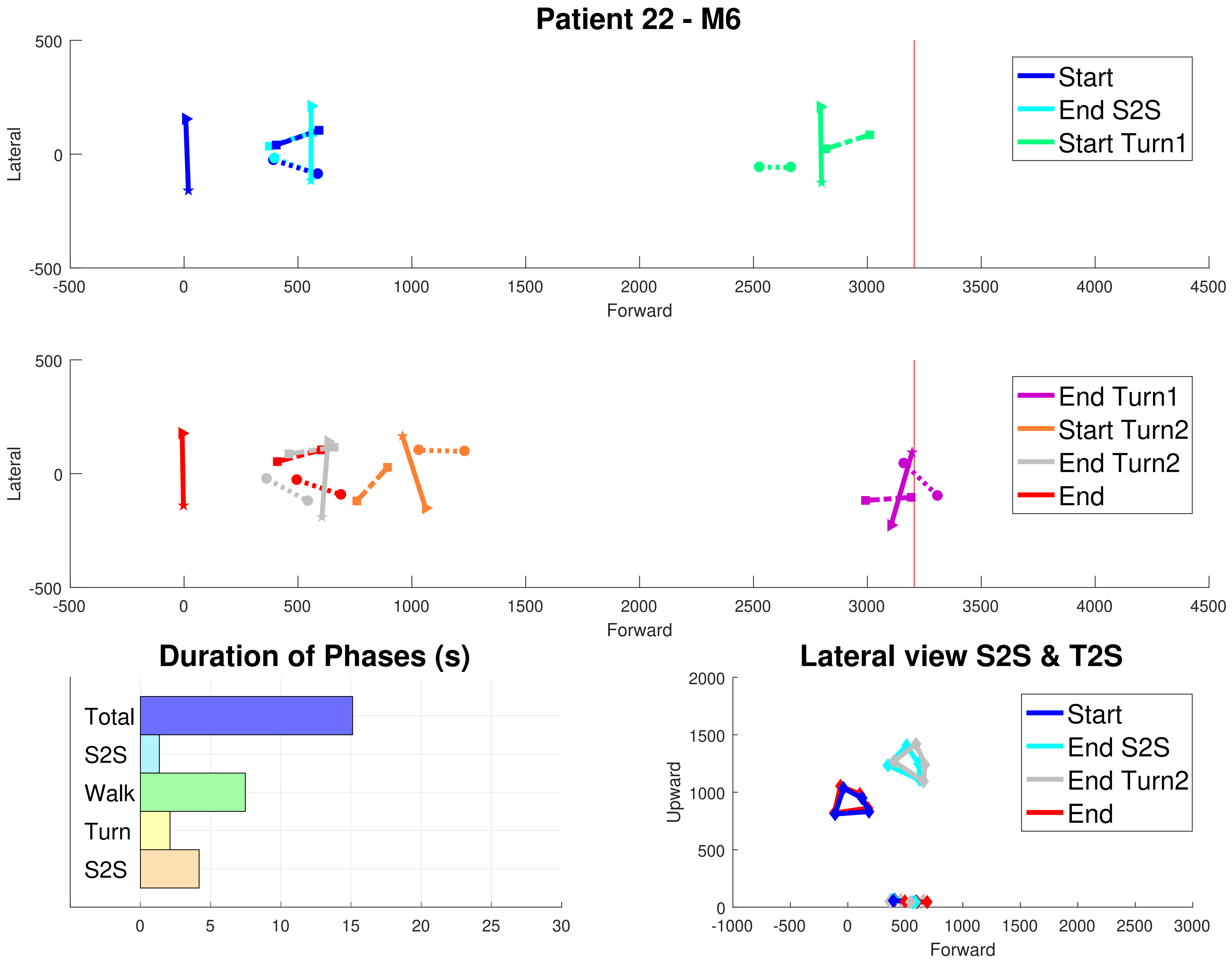

Patient 22 - M6
Lateral
Forward
Start
End S2S
Start Turn1
End Turn1
Start Turn2
End Turn2
End
Duration of Phases (s)
Total
S2S
Walk
Turn
S2S
Lateral view S2S & T2S
Upward
Forward
Start
End S2S
End Turn2
End

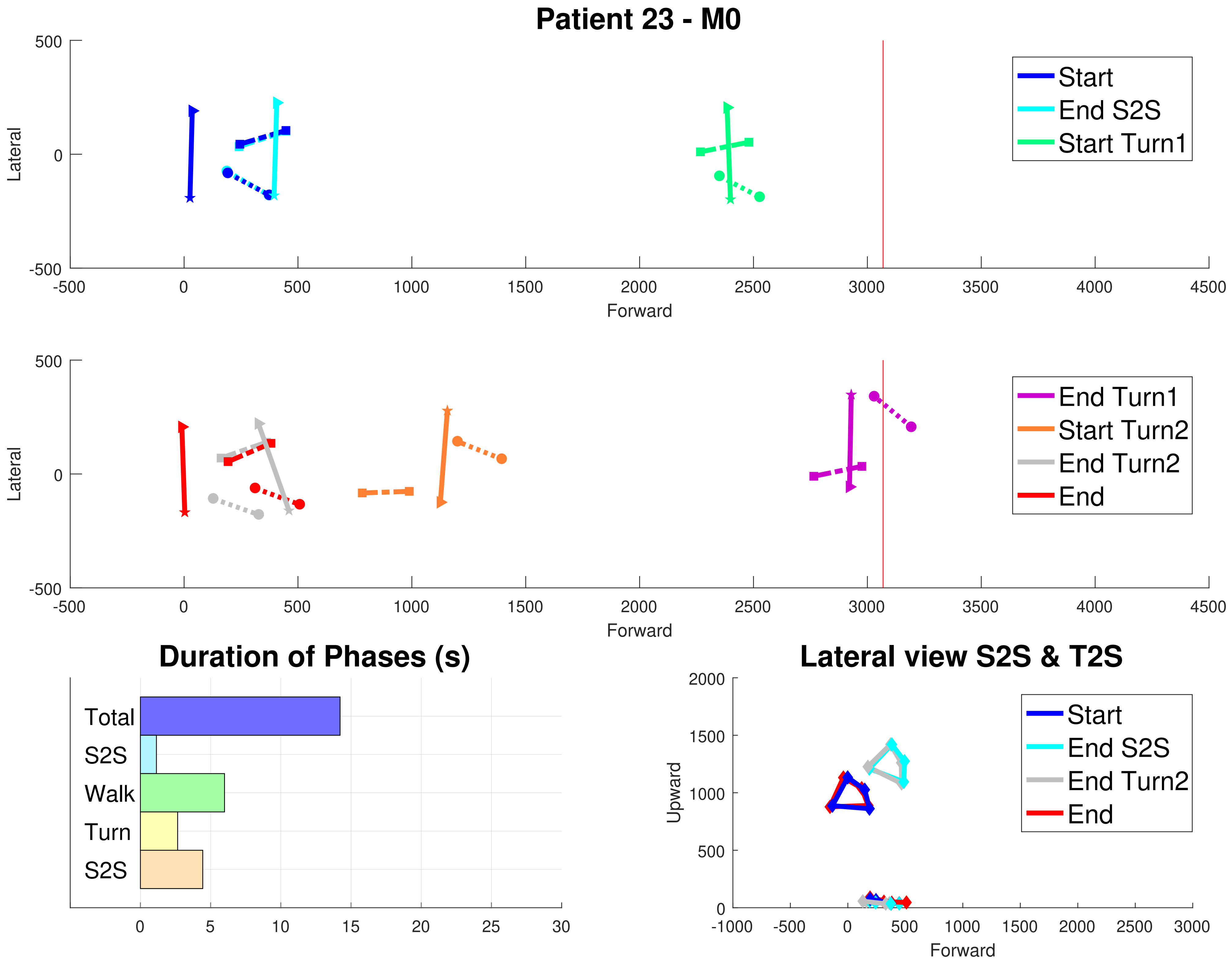
Patient 23 - M0
Lateral
Forward
Start
End S2S
Start Turn1
End Turn1
Start Turn2
End Turn2
End
500
0
-500
-500
0
500
1000
1500
2000
2500
3000
3500
4000
4500
Duration of Phases (s)
Total
S2S
Walk
Turn
S2S
0
5
10
15
20
25
30
Lateral view S2S & T2S
Upward
2000
1500
1000
500
0
-1000
-500
0
500
1000
1500
2000
2500
3000

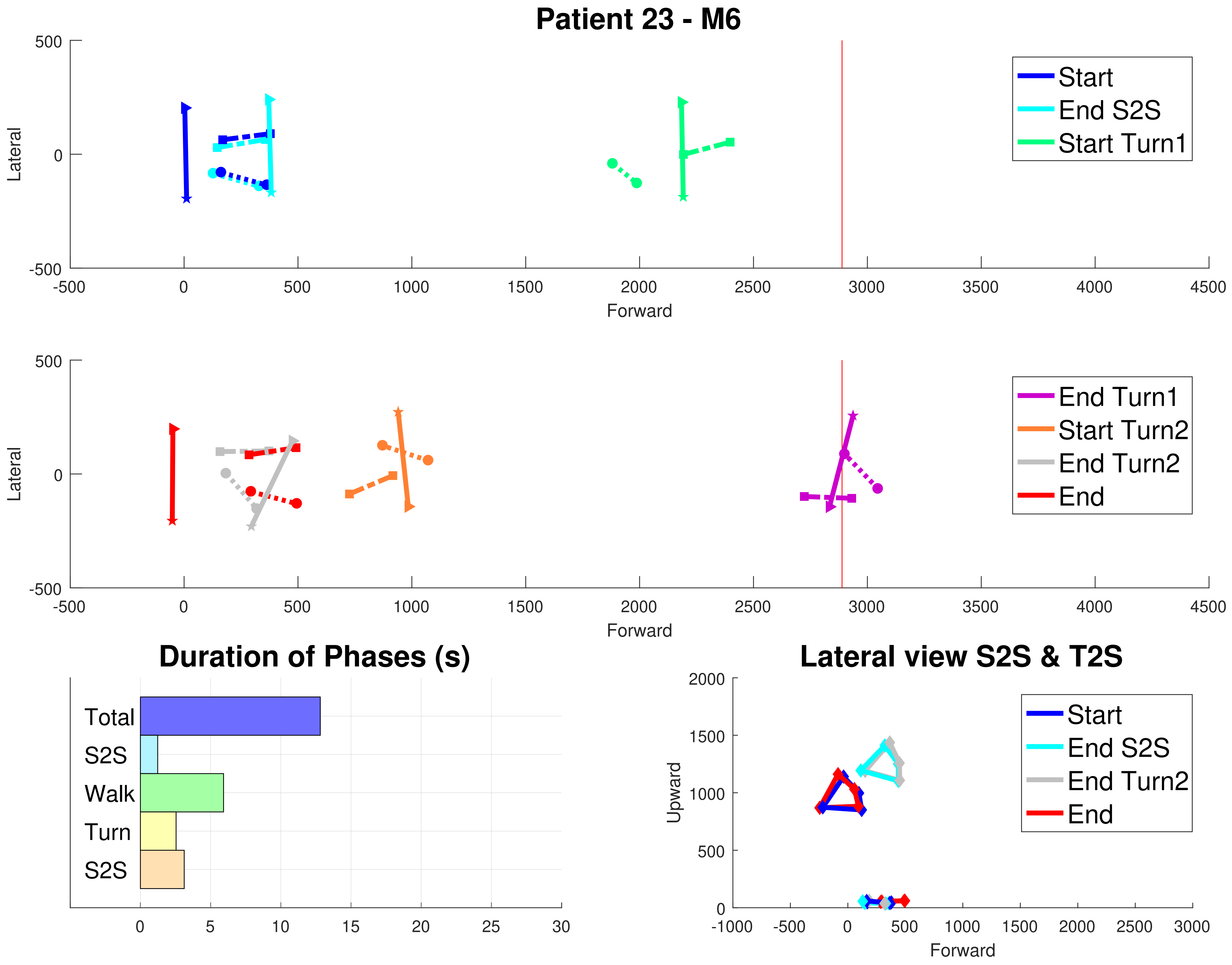

Patient 23 - M6
Lateral
Forward
Start
End S2S
Start Turn1
End Turn1
Start Turn2
End Turn2
End
Duration of Phases (s)
Total
S2S
Walk
Turn
S2S
Lateral view S2S & T2S
Upward
Forward
Start
End S2S
End Turn2
End

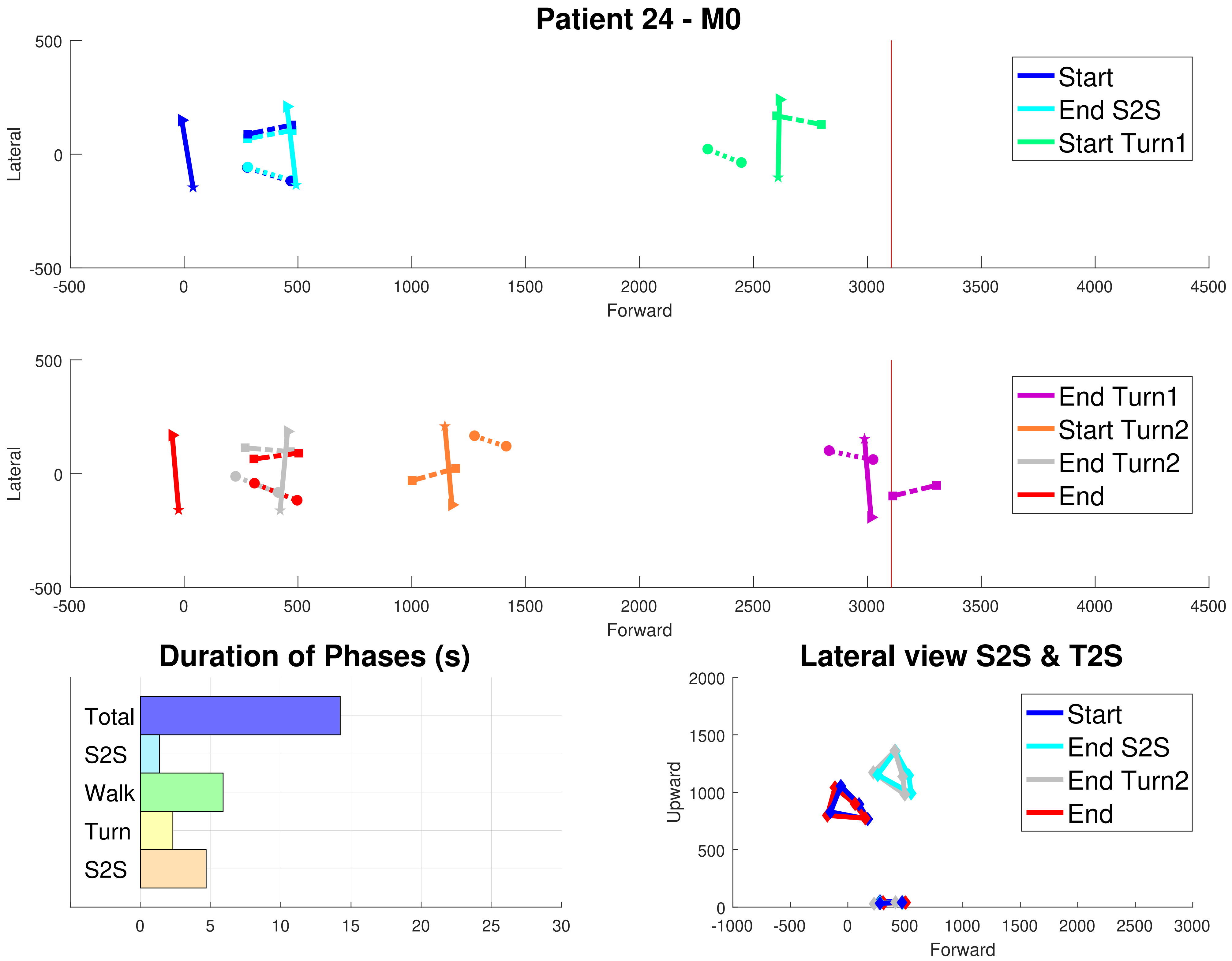
Patient 24 - M0
Lateral
Forward
500
0
-500
-500 0 500 1000 1500 2000 2500 3000 3500 4000 4500
Start
End S2S
Start Turn1
End Turn1
Start Turn2
End Turn2
End
Duration of Phases (s)
Total
S2S
Walk
Turn
S2S
0 5 10 15 20 25 30
Lateral view S2S & T2S
Upward
2000
1500
1000
500
0
-1000 -500 0 500 1000 1500 2000 2500 3000
Start
End S2S
End Turn2
End

Patient 24 - M6

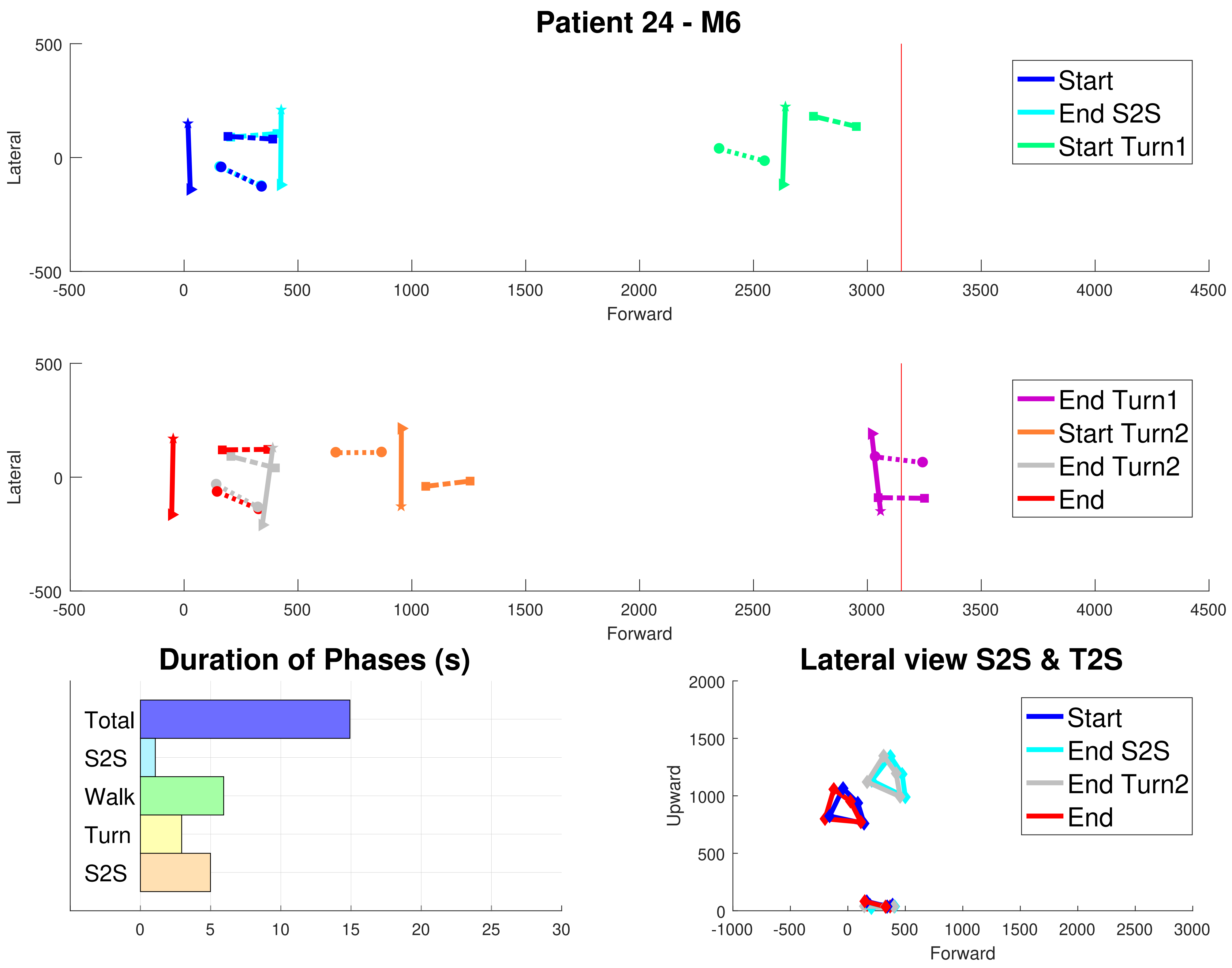

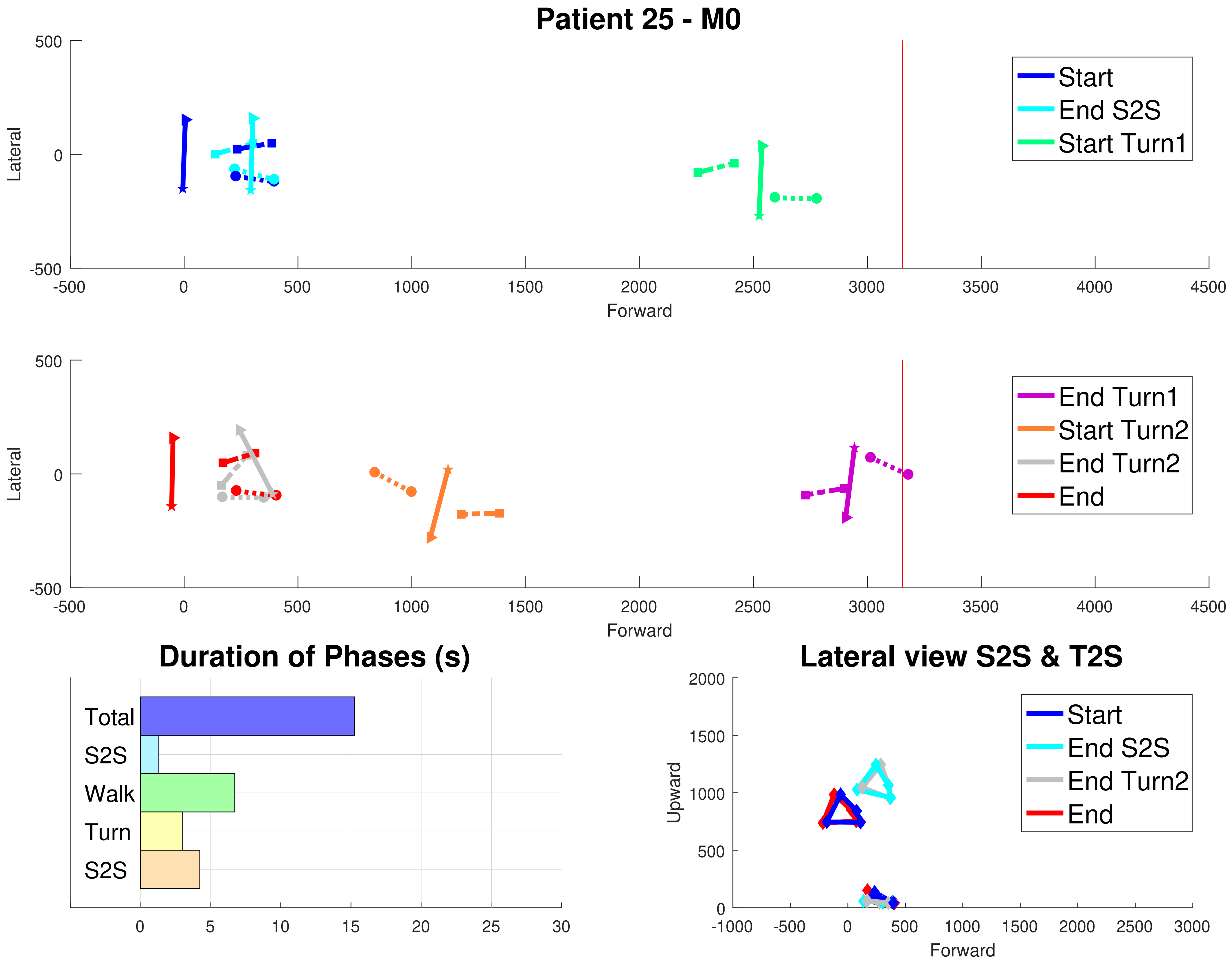
Patient 25 - M0
Lateral
Forward
Start
End S2S
Start Turn1
End Turn1
Start Turn2
End Turn2
End
Duration of Phases (s)
Total
S2S
Walk
Turn
S2S
Lateral view S2S & T2S
Upward
Forward
Start
End S2S
End Turn2
End

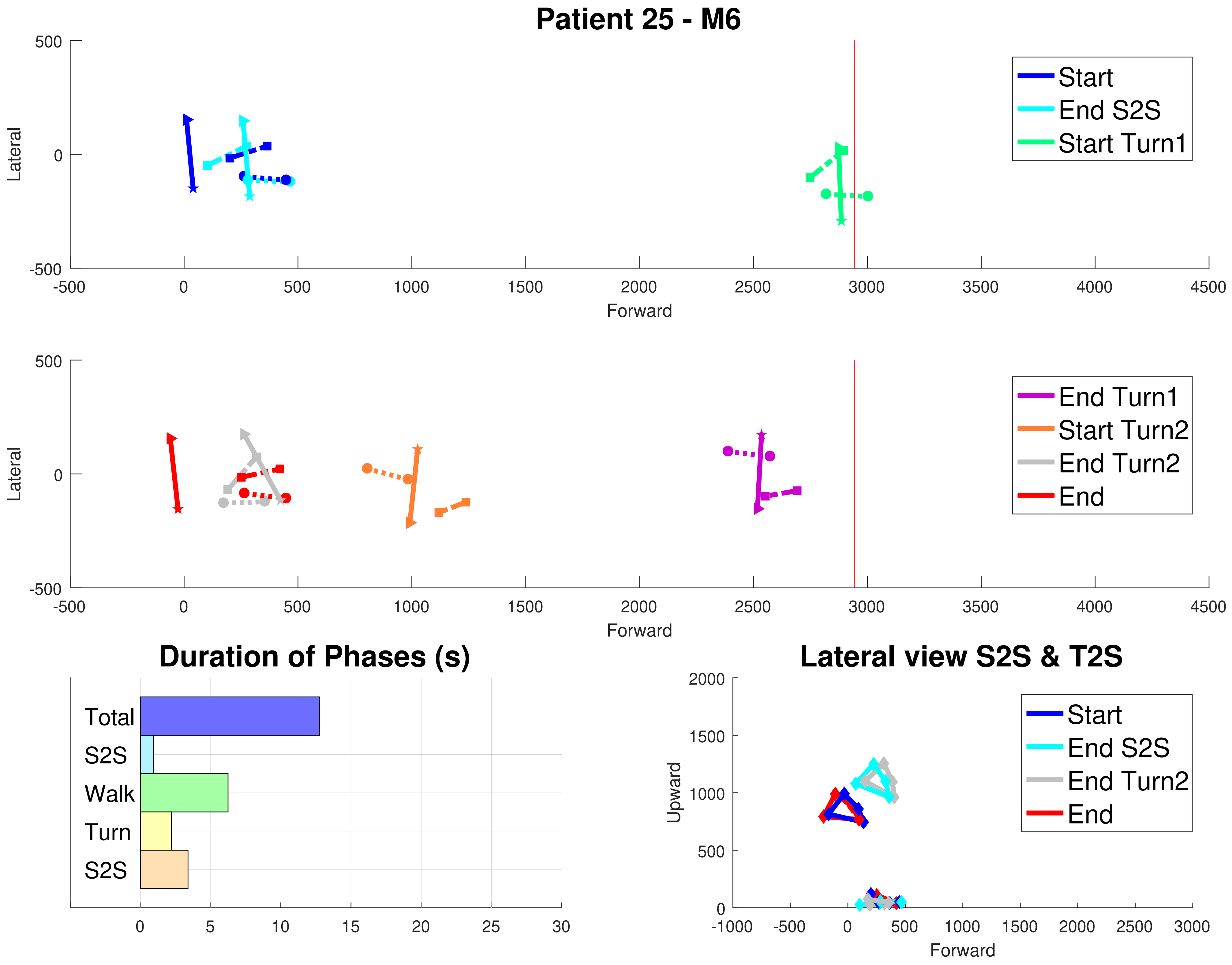
Patient 25 - M6
Lateral
Forward
Start
End S2S
Start Turn1
End Turn1
Start Turn2
End Turn2
End
Duration of Phases (s)
Total
S2S
Walk
Turn
S2S
Lateral view S2S & T2S
Upward
Forward
Start
End S2S
End Turn2
End

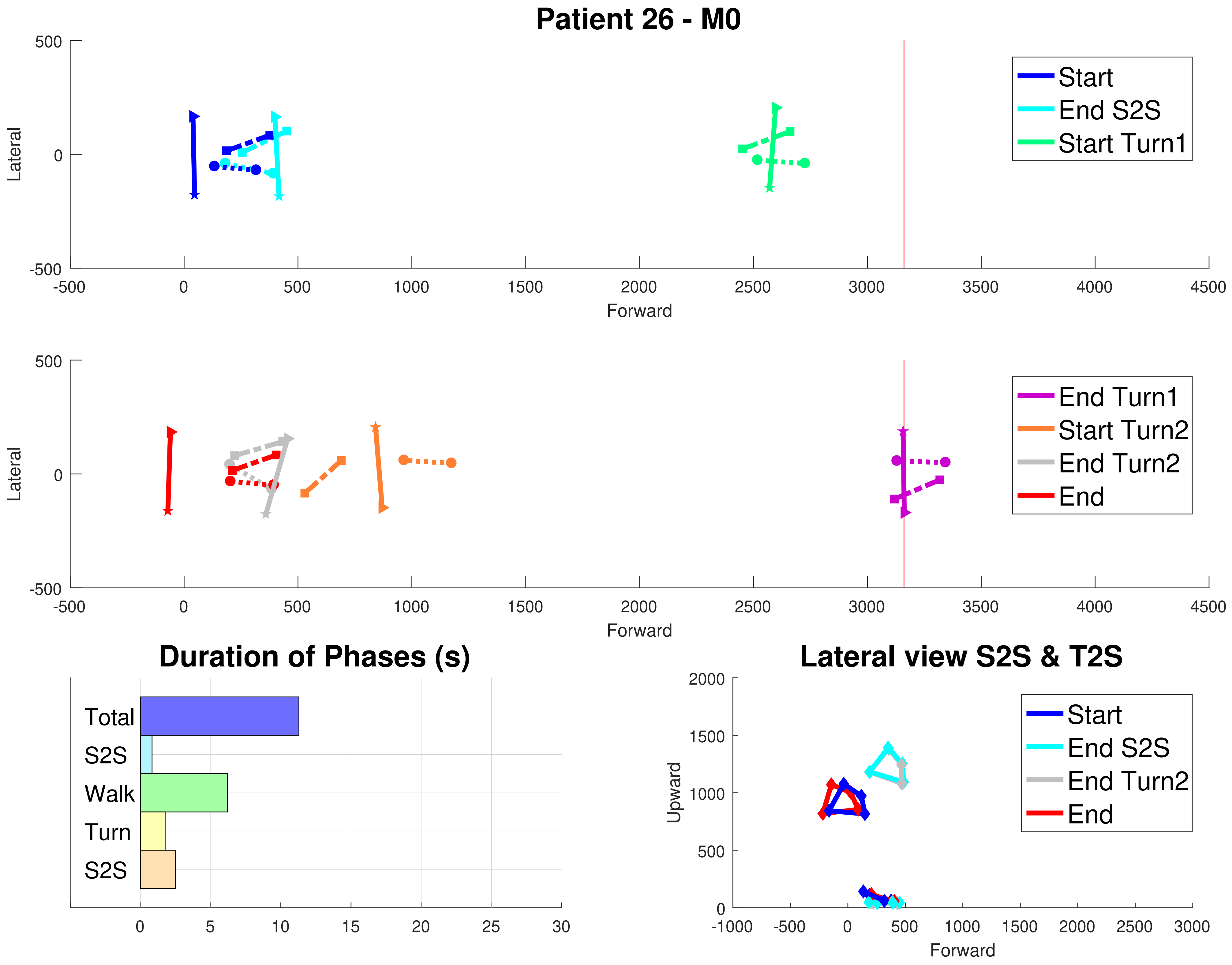
Patient 26 - M0
Lateral
Forward
Start
End S2S
Start Turn1
End Turn1
Start Turn2
End Turn2
End
Duration of Phases (s)
Total
S2S
Walk
Turn
S2S
Lateral view S2S & T2S
Upward
Forward
Start
End S2S
End Turn2
End

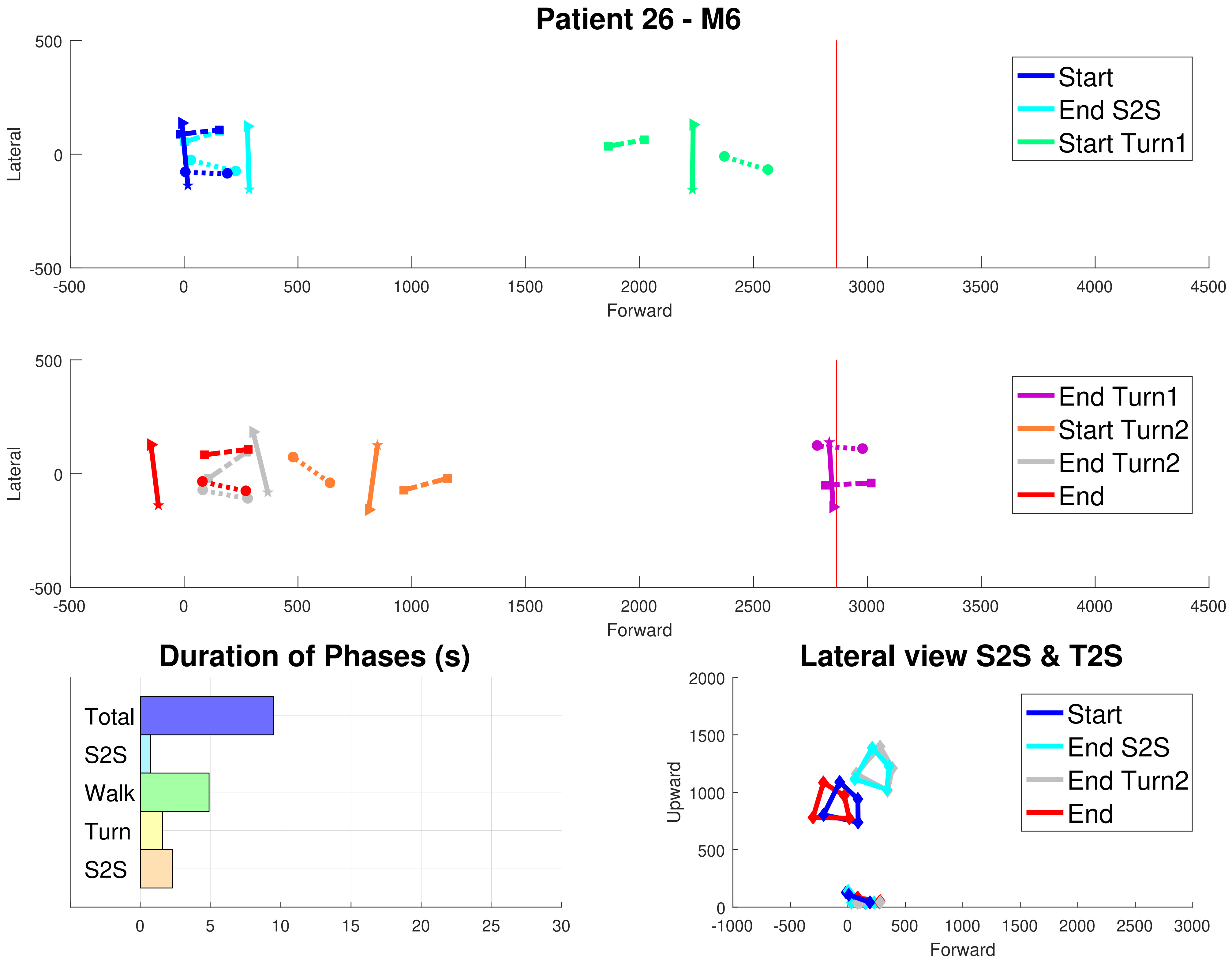

Patient 26 - M6
Lateral
Forward
Start
End S2S
Start Turn1
End Turn1
Start Turn2
End Turn2
End
Duration of Phases (s)
Total
S2S
Walk
Turn
S2S
Lateral view S2S & T2S
Upward
Forward
Start
End S2S
End Turn2
End

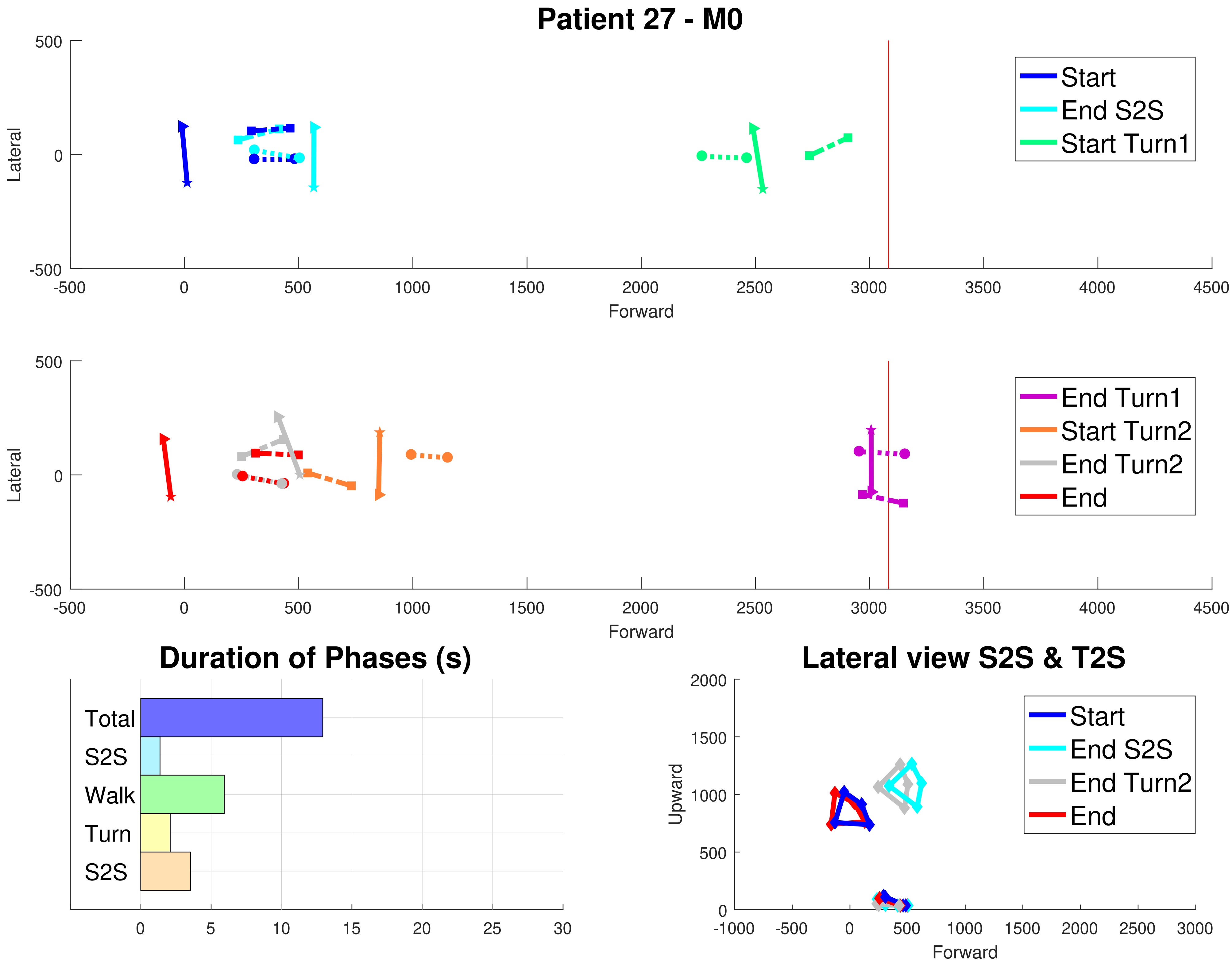
Patient 27 - M0
Lateral
Forward
Start
End S2S
Start Turn1
End Turn1
Start Turn2
End Turn2
End
Duration of Phases (s)
Total
S2S
Walk
Turn
S2S
Lateral view S2S & T2S
Upward
Forward
Start
End S2S
End Turn2
End

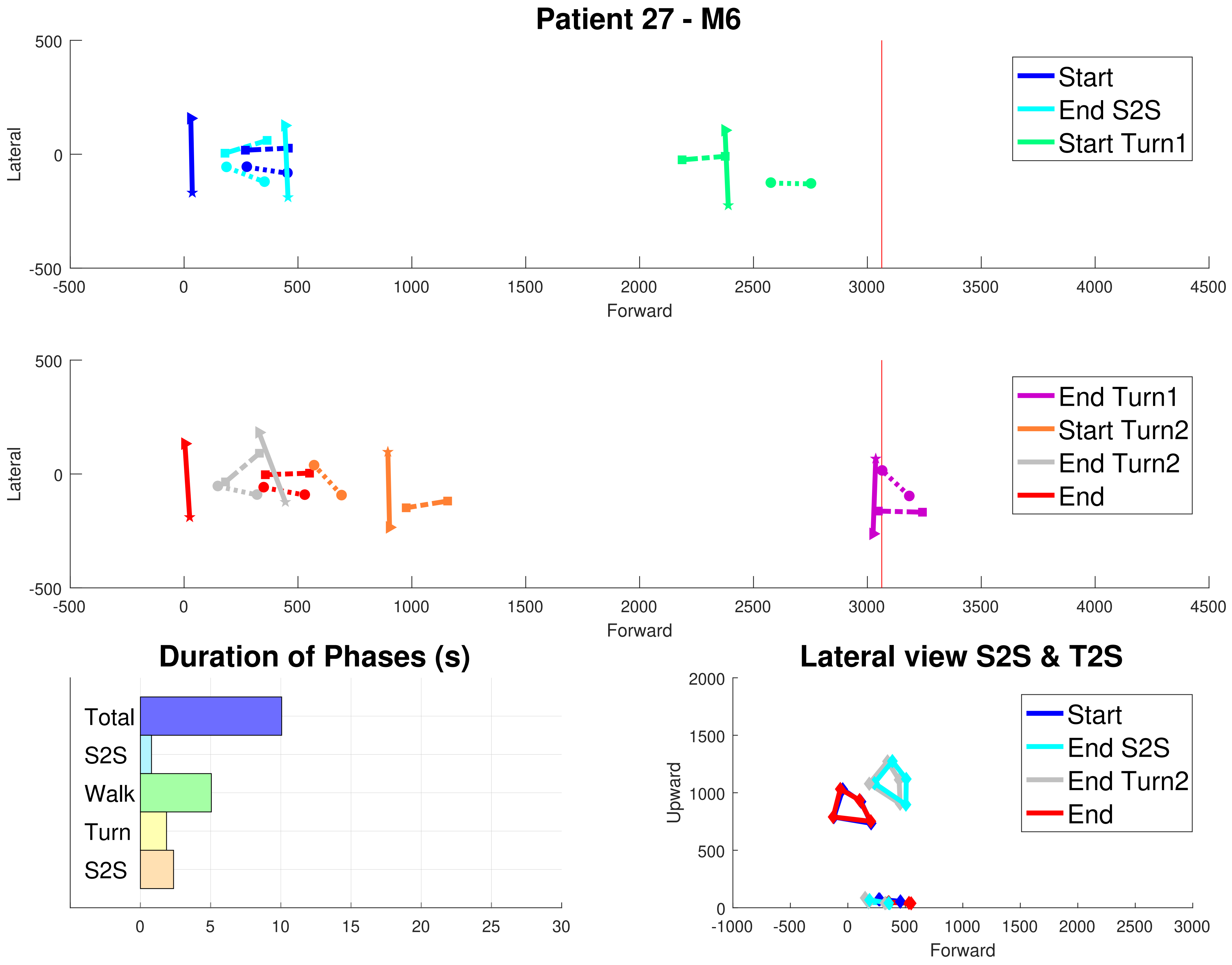
Patient 27 - M6
Lateral
Forward
Start
End S2S
Start Turn1
End Turn1
Start Turn2
End Turn2
End
Duration of Phases (s)
Total
S2S
Walk
Turn
S2S
Lateral view S2S & T2S
Upward

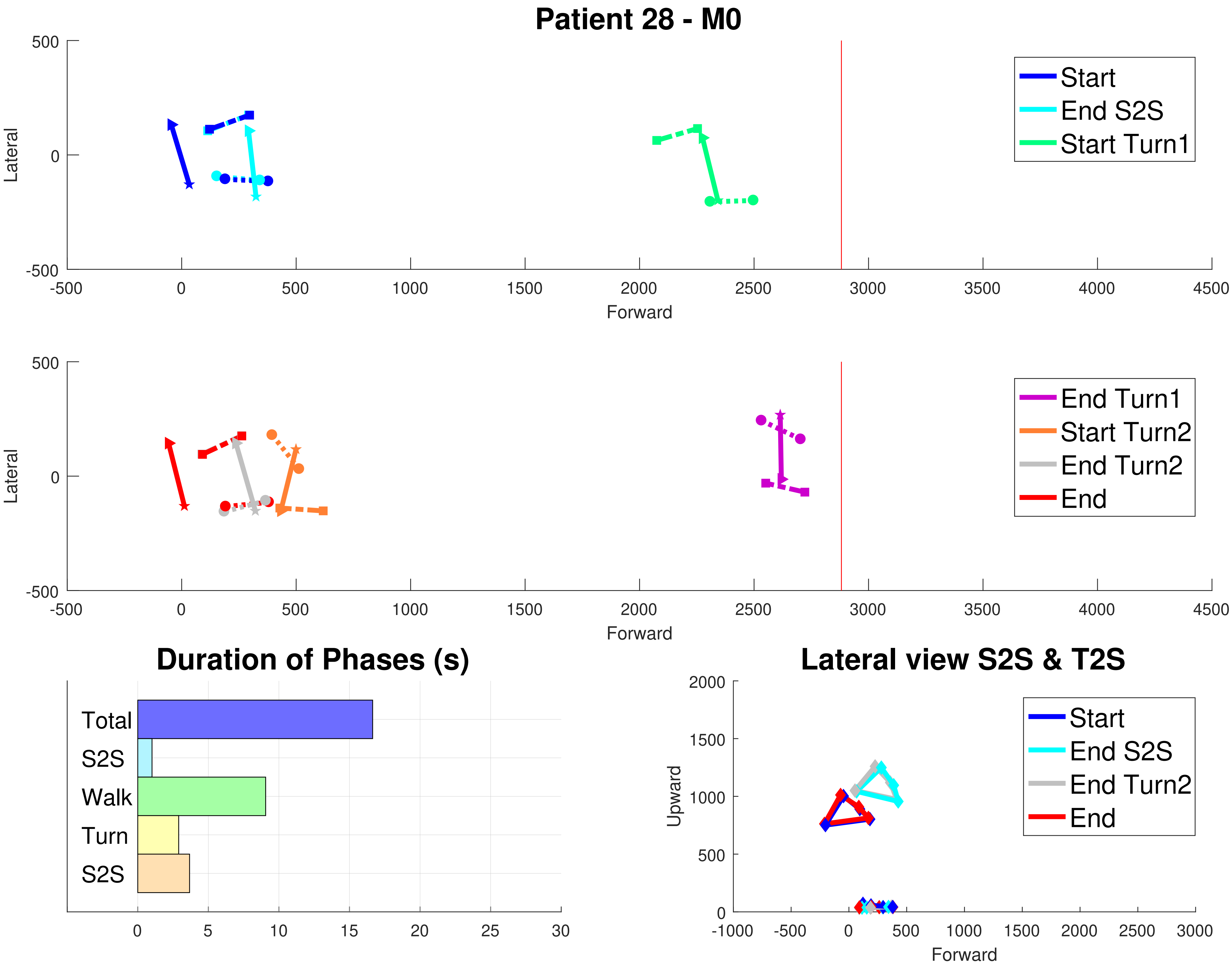
Patient 28 - M0
Lateral
Forward
Start
End S2S
Start Turn1
End Turn1
Start Turn2
End Turn2
End
Duration of Phases (s)
Total
S2S
Walk
Turn
S2S
Lateral view S2S & T2S
Upward
Forward
Start
End S2S
End Turn2
End

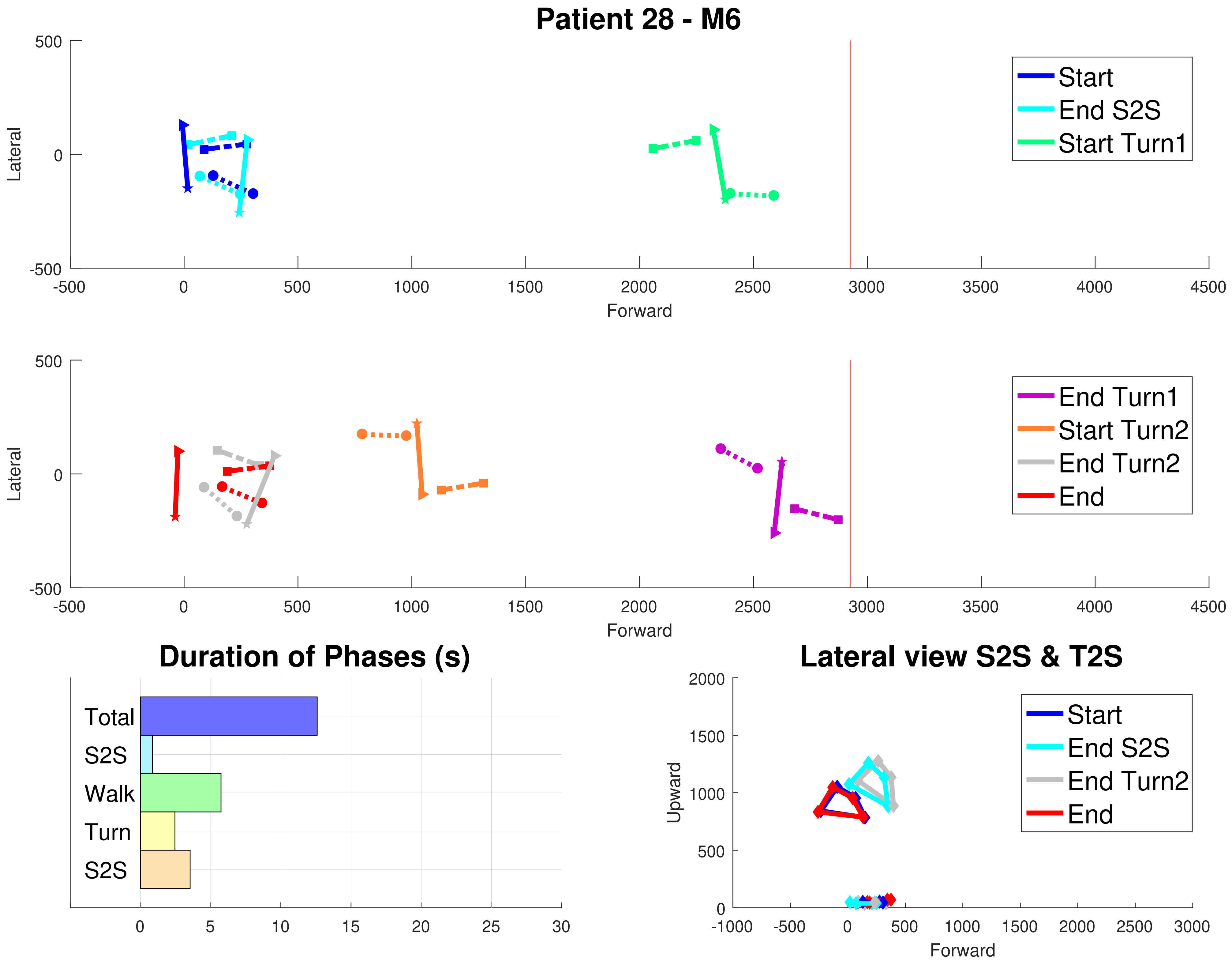

Patient 28 - M6
Lateral
Forward
Start
End S2S
Start Turn1
End Turn1
Start Turn2
End Turn2
End
Duration of Phases (s)
Total
S2S
Walk
Turn
S2S
Lateral view S2S & T2S
Upward
Forward
Start
End S2S
End Turn2
End

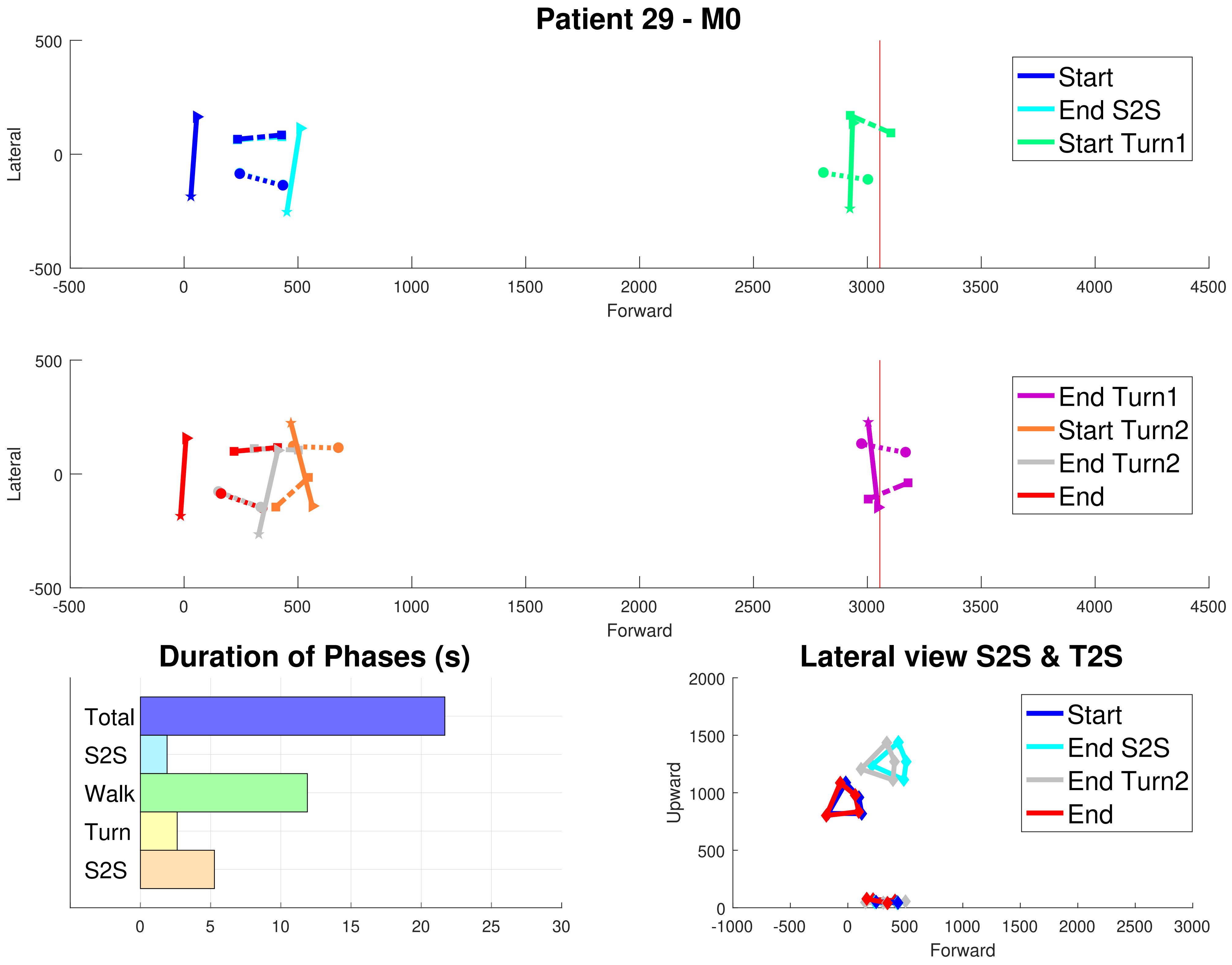

Patient 29 - M0
Lateral
Forward
Start
End S2S
Start Turn1
End Turn1
Start Turn2
End Turn2
End
Duration of Phases (s)
Total
S2S
Walk
Turn
S2S
Lateral view S2S & T2S
Upward

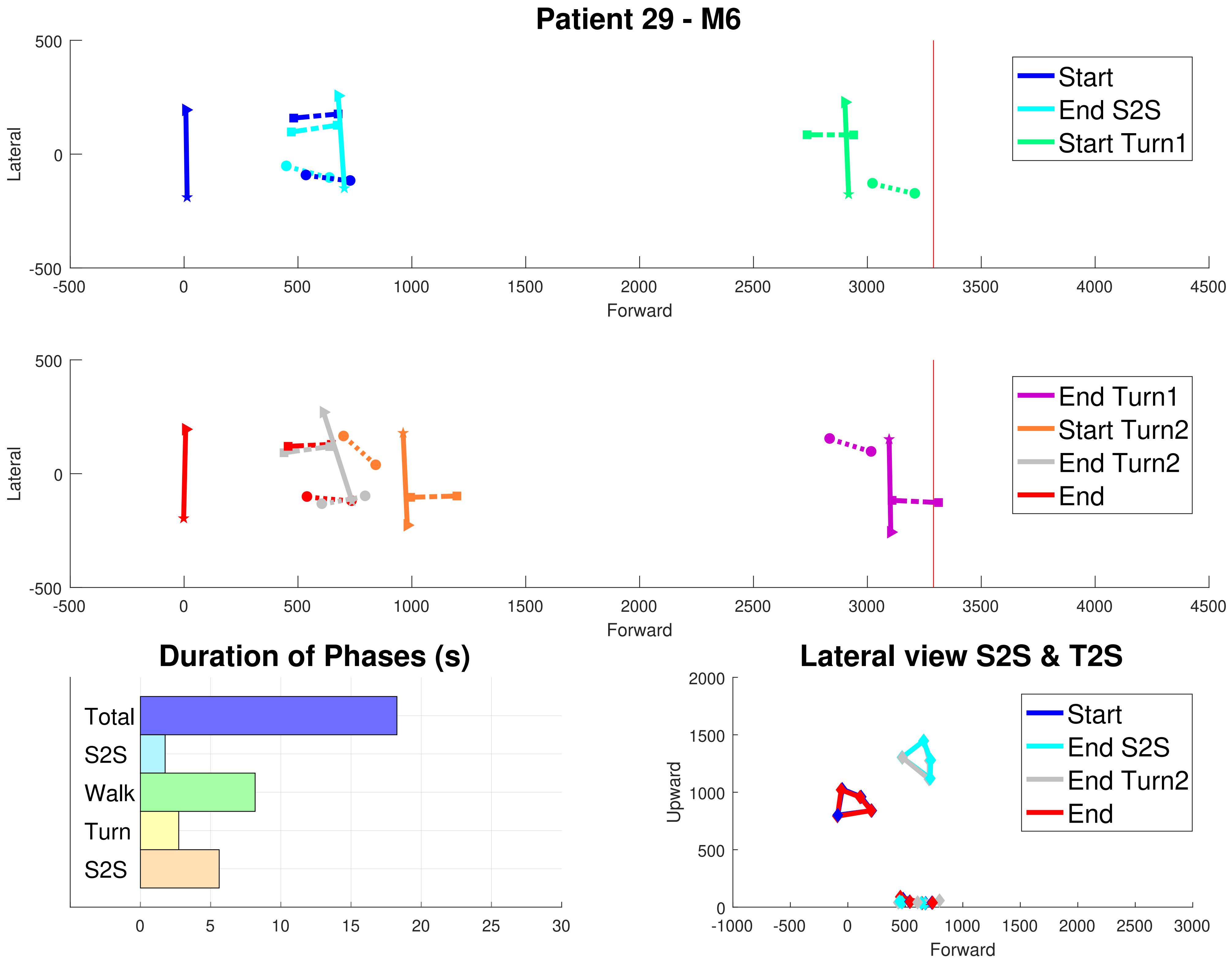

Patient 29 - M6
Lateral
Forward
Start
End S2S
Start Turn1
End Turn1
Start Turn2
End Turn2
End
Duration of Phases (s)
Total
S2S
Walk
Turn
S2S
Lateral view S2S & T2S
Upward
Forward
Start
End S2S
End Turn2
End

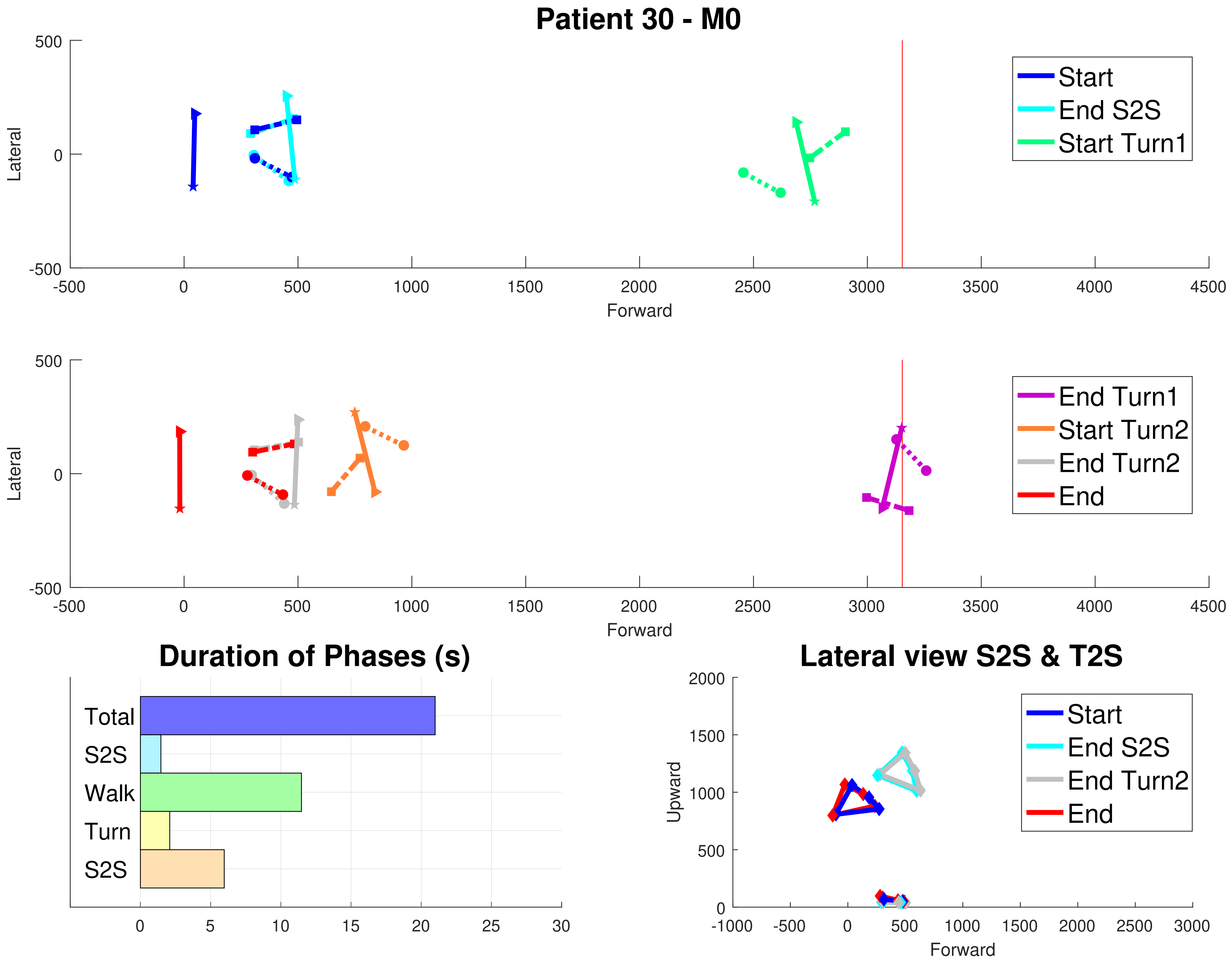

Patient 30 - M0
Lateral
Forward
Start
End S2S
Start Turn1
End Turn1
Start Turn2
End Turn2
End
Duration of Phases (s)
Total
S2S
Walk
Turn
S2S
Lateral view S2S & T2S
Upward

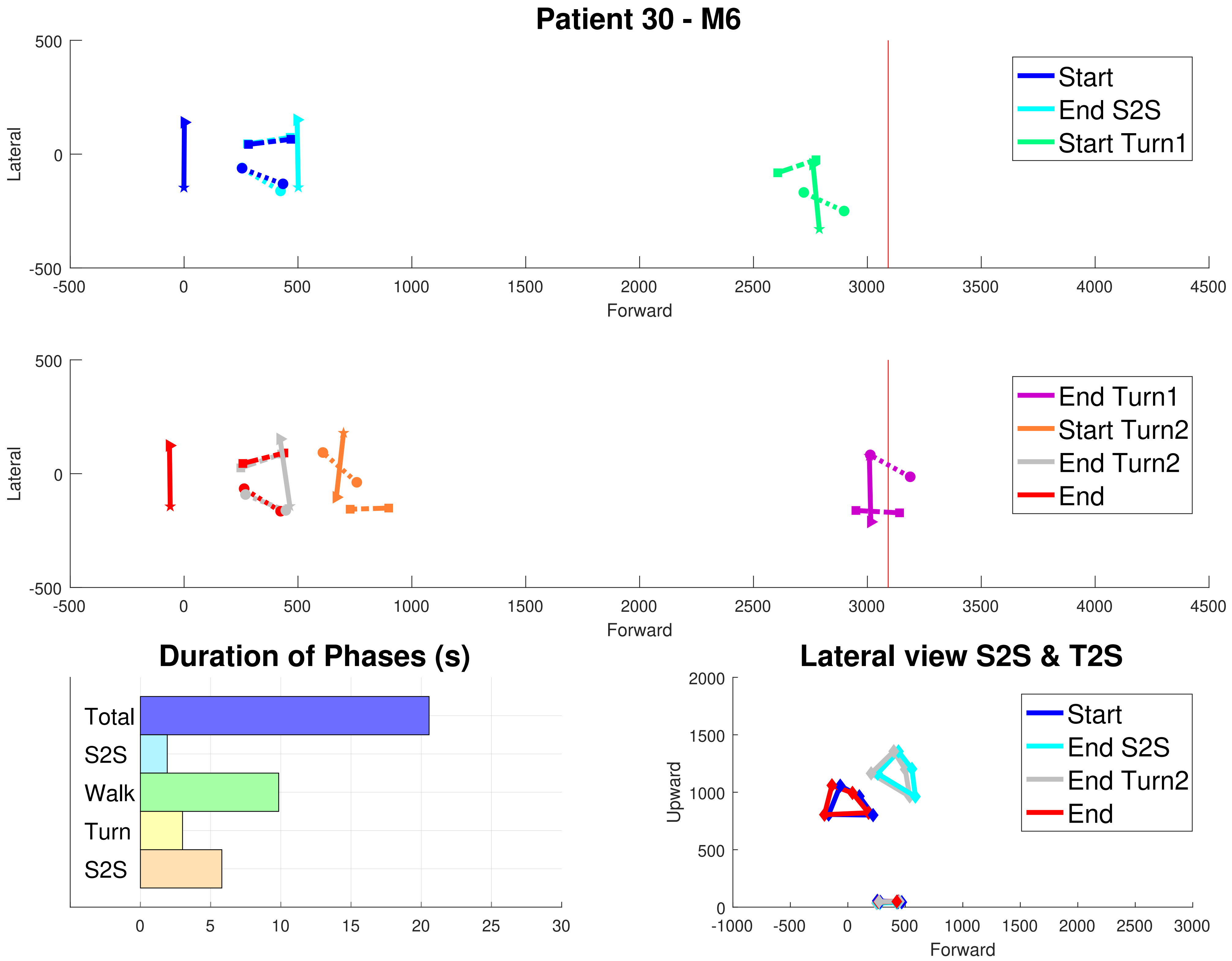

Patient 30 - M6
Lateral
Forward
Start
End S2S
Start Turn1
End Turn1
Start Turn2
End Turn2
End
Duration of Phases (s)
Total
S2S
Walk
Turn
S2S
Lateral view S2S & T2S
Upward
Start
End S2S
End Turn2
End

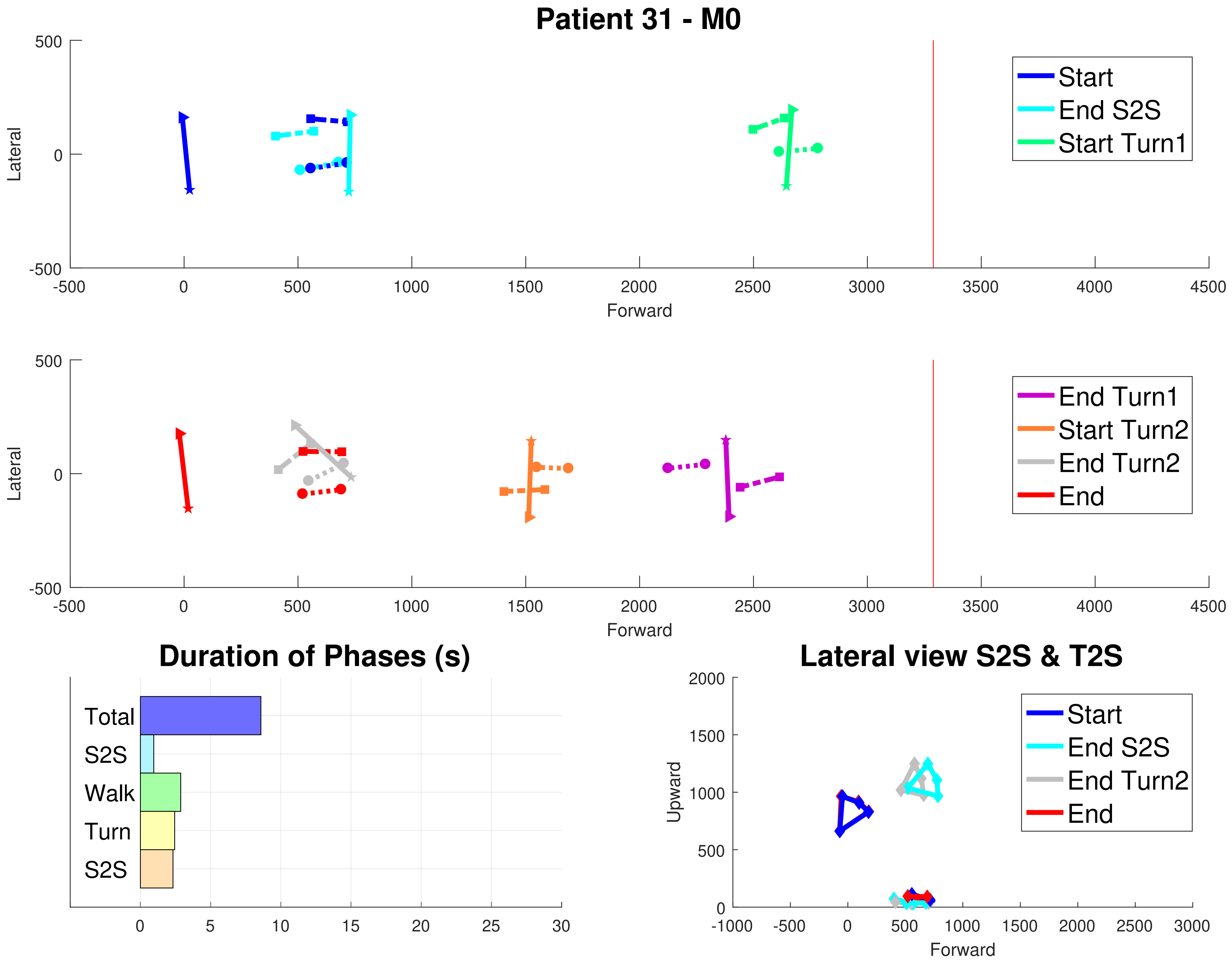
Patient 31 - M0
Lateral
Forward
Start
End S2S
Start Turn1
End Turn1
Start Turn2
End Turn2
End
Duration of Phases (s)
Total
S2S
Walk
Turn
S2S
Lateral view S2S & T2S
Upward
Forward
Start
End S2S
End Turn2
End

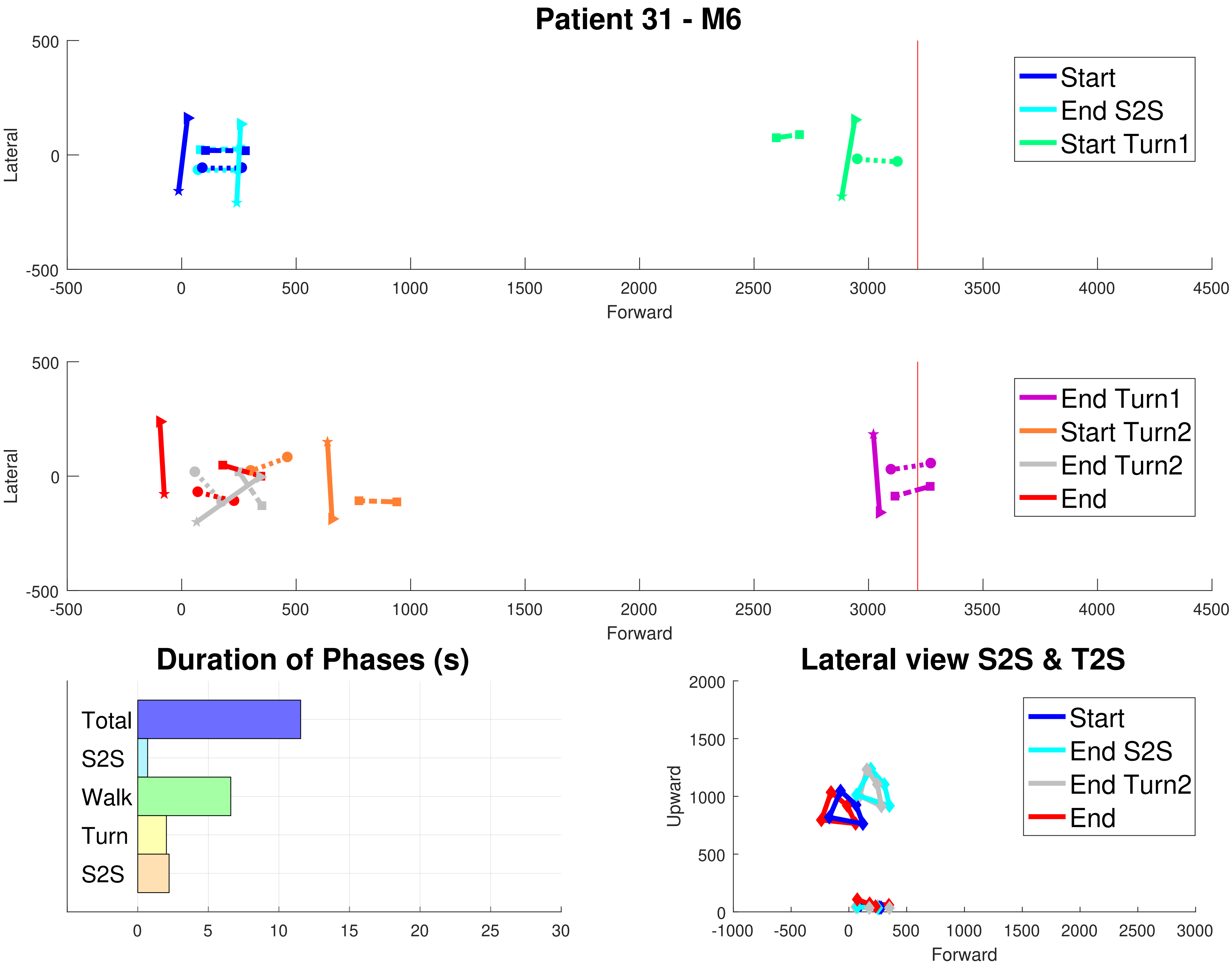

Patient 31 - M6
Start
End S2S
Start Turn1
End Turn1
Start Turn2
End Turn2
End
Lateral
Forward
Duration of Phases (s)
Total
S2S
Walk
Turn
S2S
Lateral view S2S & T2S
Upward
Start
End S2S
End Turn2
End

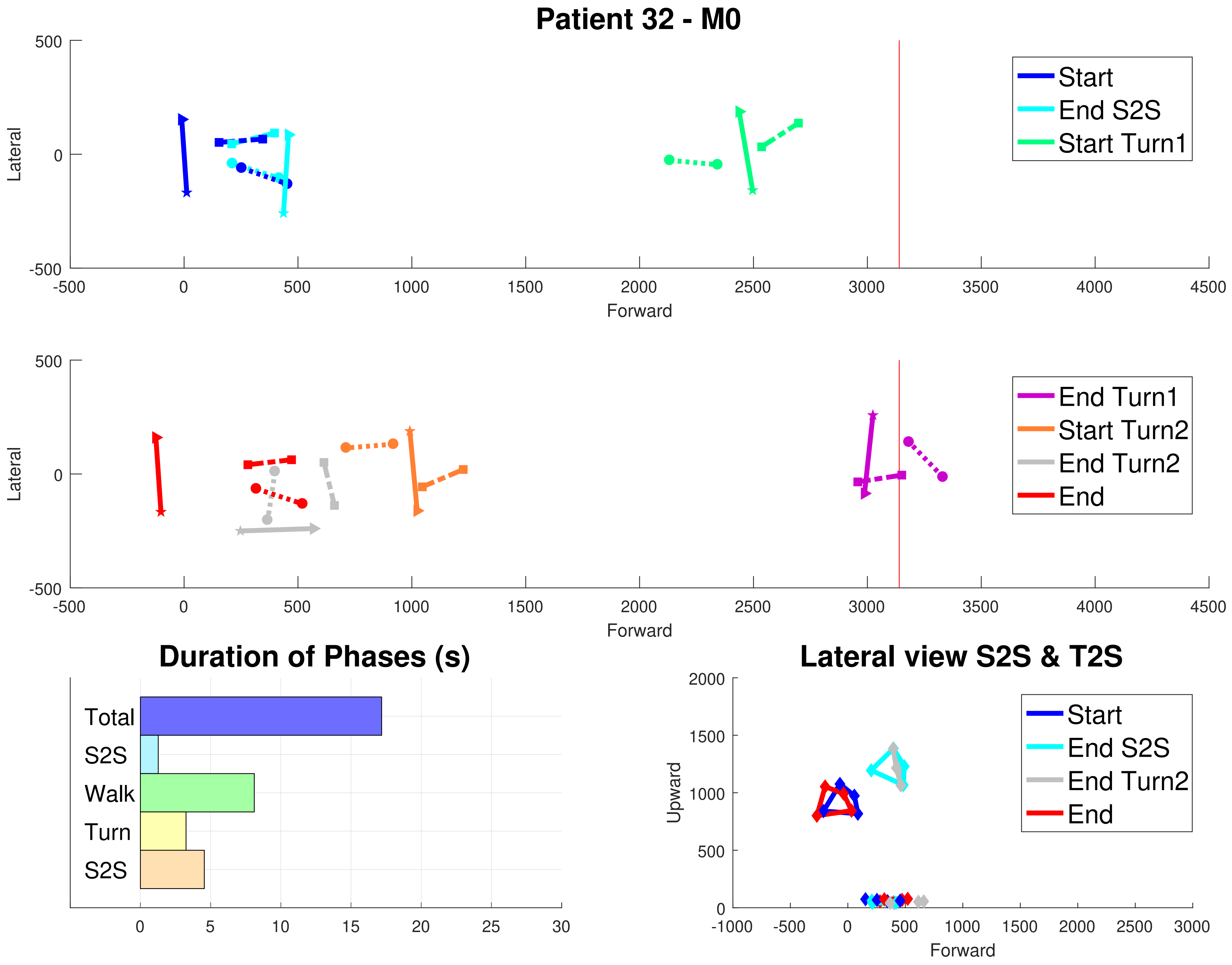
Patient 32 - M0
Lateral
Forward
Start
End S2S
Start Turn1
End Turn1
Start Turn2
End Turn2
End
Duration of Phases (s)
Total
S2S
Walk
Turn
S2S
Lateral view S2S & T2S
Upward
Forward
Start
End S2S
End Turn2
End

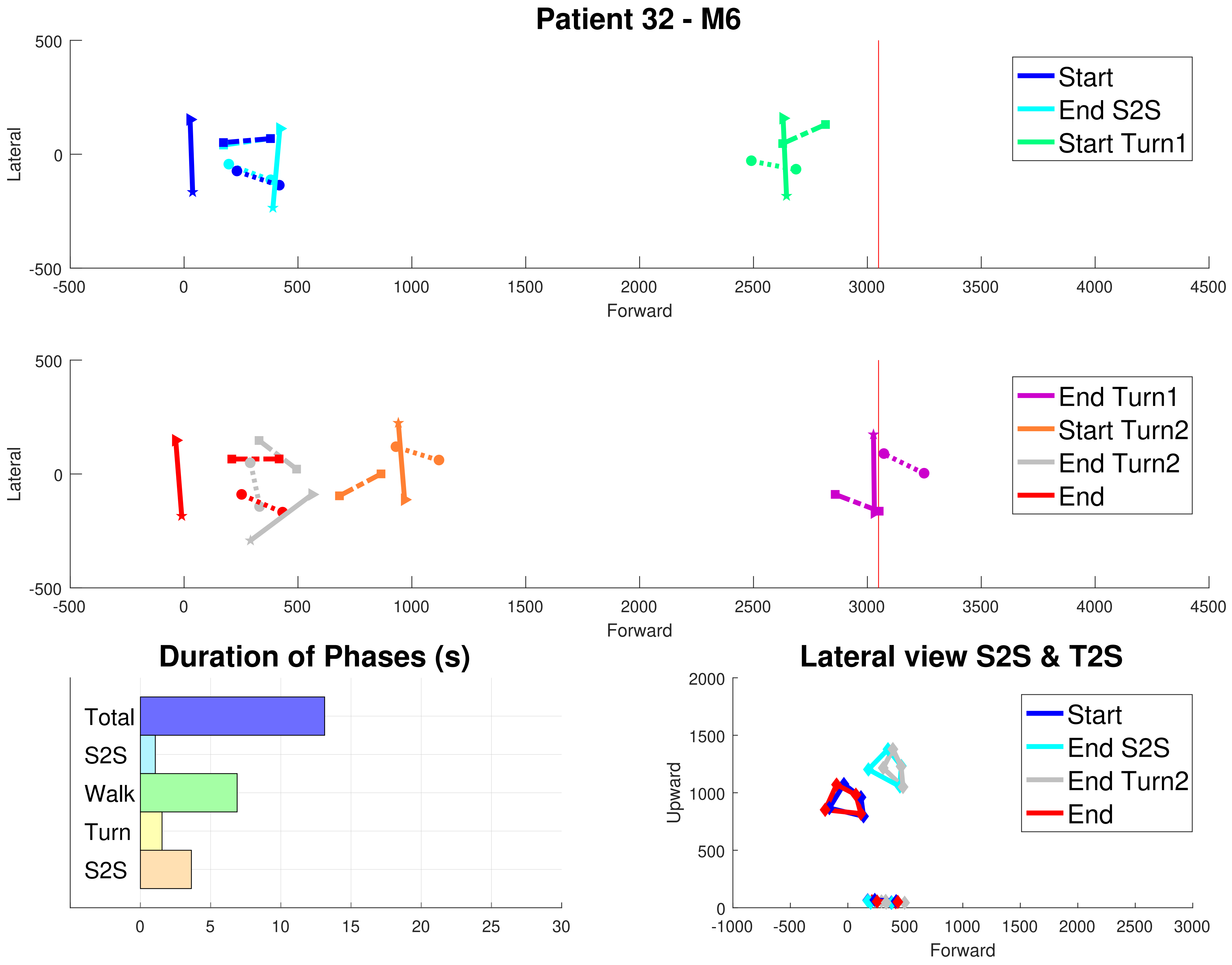

Patient 32 - M6
Lateral
Forward
Start
End S2S
Start Turn1
End Turn1
Start Turn2
End Turn2
End
Duration of Phases (s)
Total
S2S
Walk
Turn
S2S
Lateral view S2S & T2S
Upward
Forward
Start
End S2S
End Turn2
End

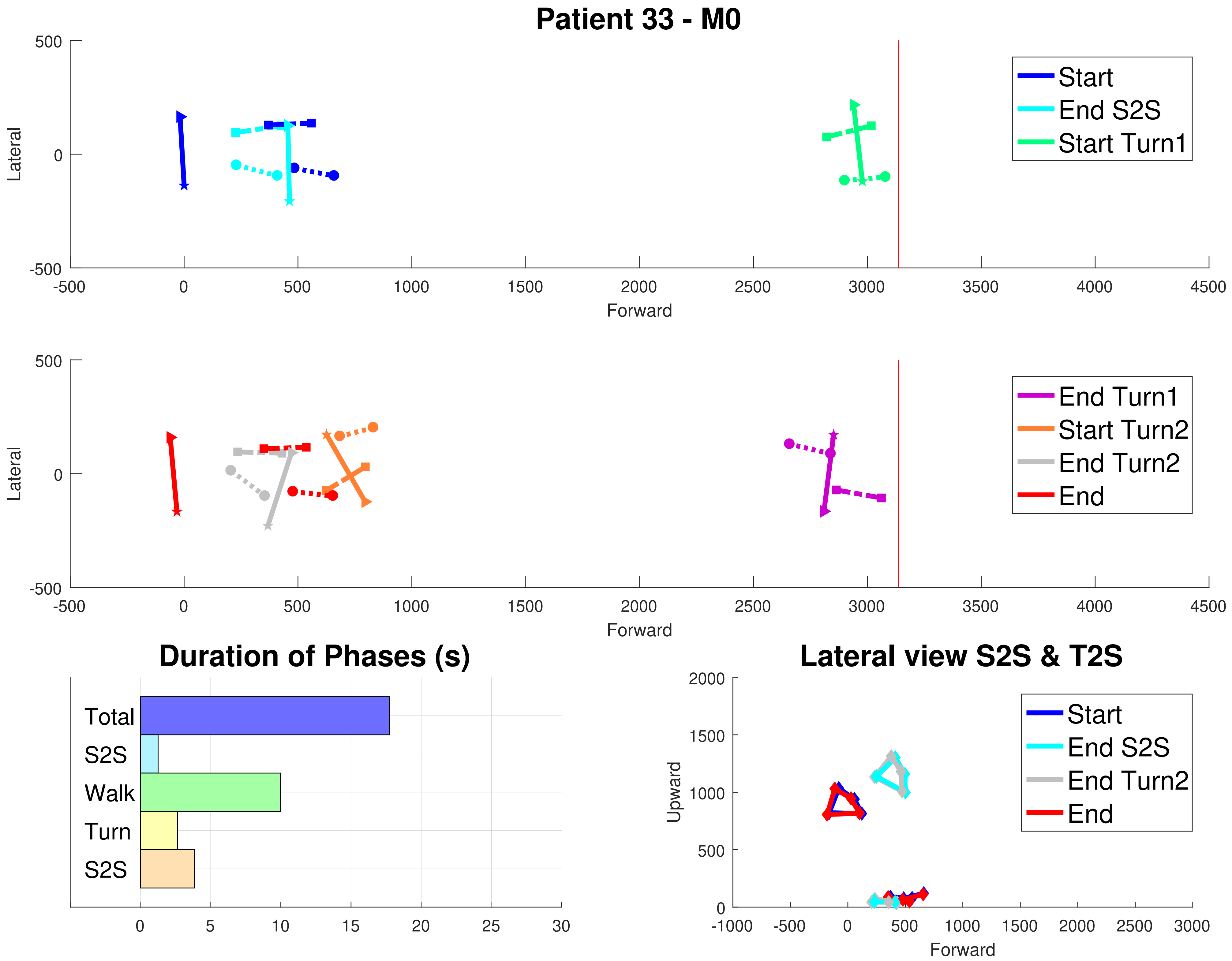

Patient 33 - M0
Lateral
Forward
Start
End S2S
Start Turn1
End Turn1
Start Turn2
End Turn2
End
Duration of Phases (s)
Total
S2S
Walk
Turn
S2S
Lateral view S2S & T2S
Upward
Forward
Start
End S2S
End Turn2
End

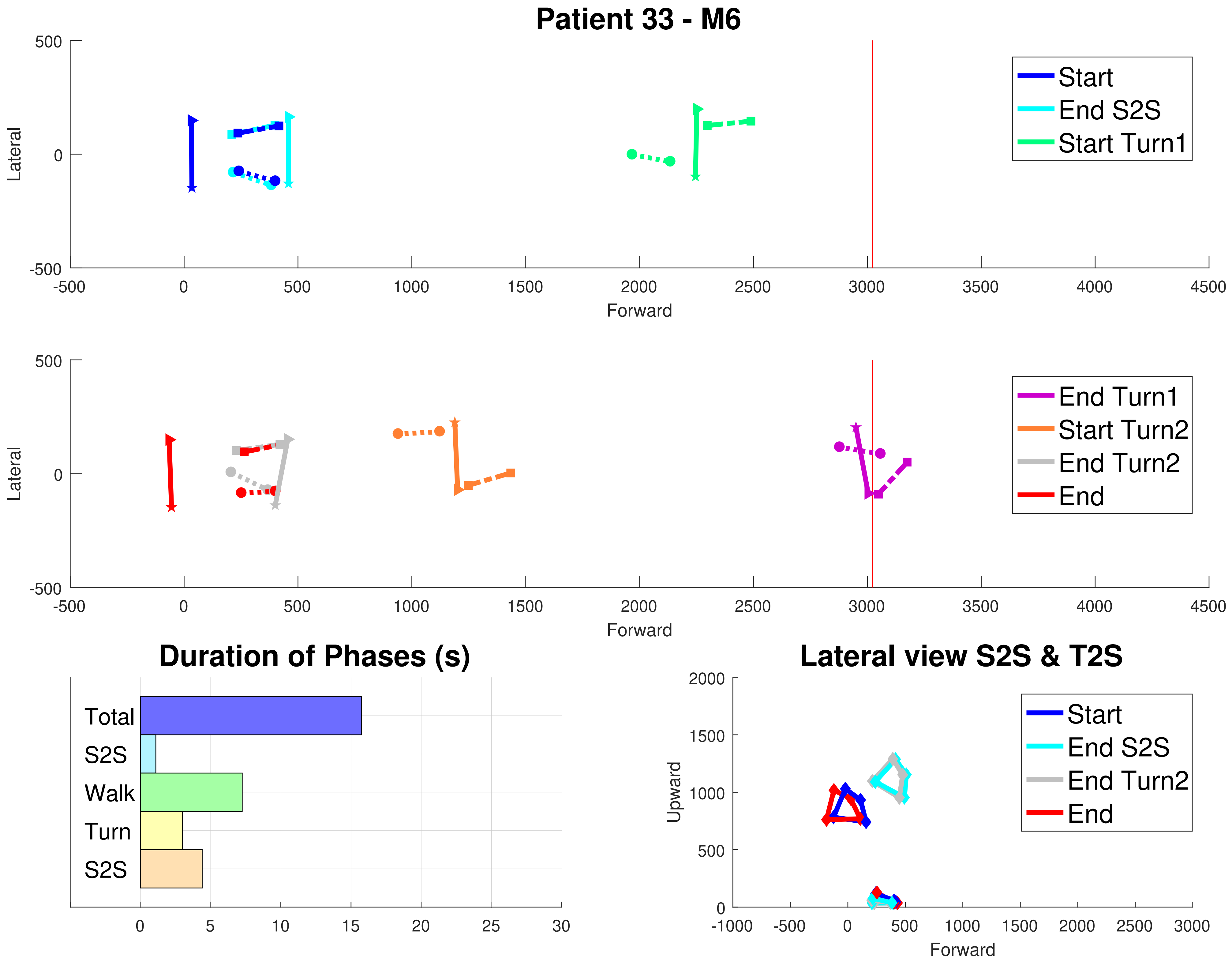

Patient 33 - M6
Lateral
Forward
Start
End S2S
Start Turn1
End Turn1
Start Turn2
End Turn2
End
Duration of Phases (s)
Total
S2S
Walk
Turn
S2S
Lateral view S2S & T2S
Upward
Forward
Start
End S2S
End Turn2
End

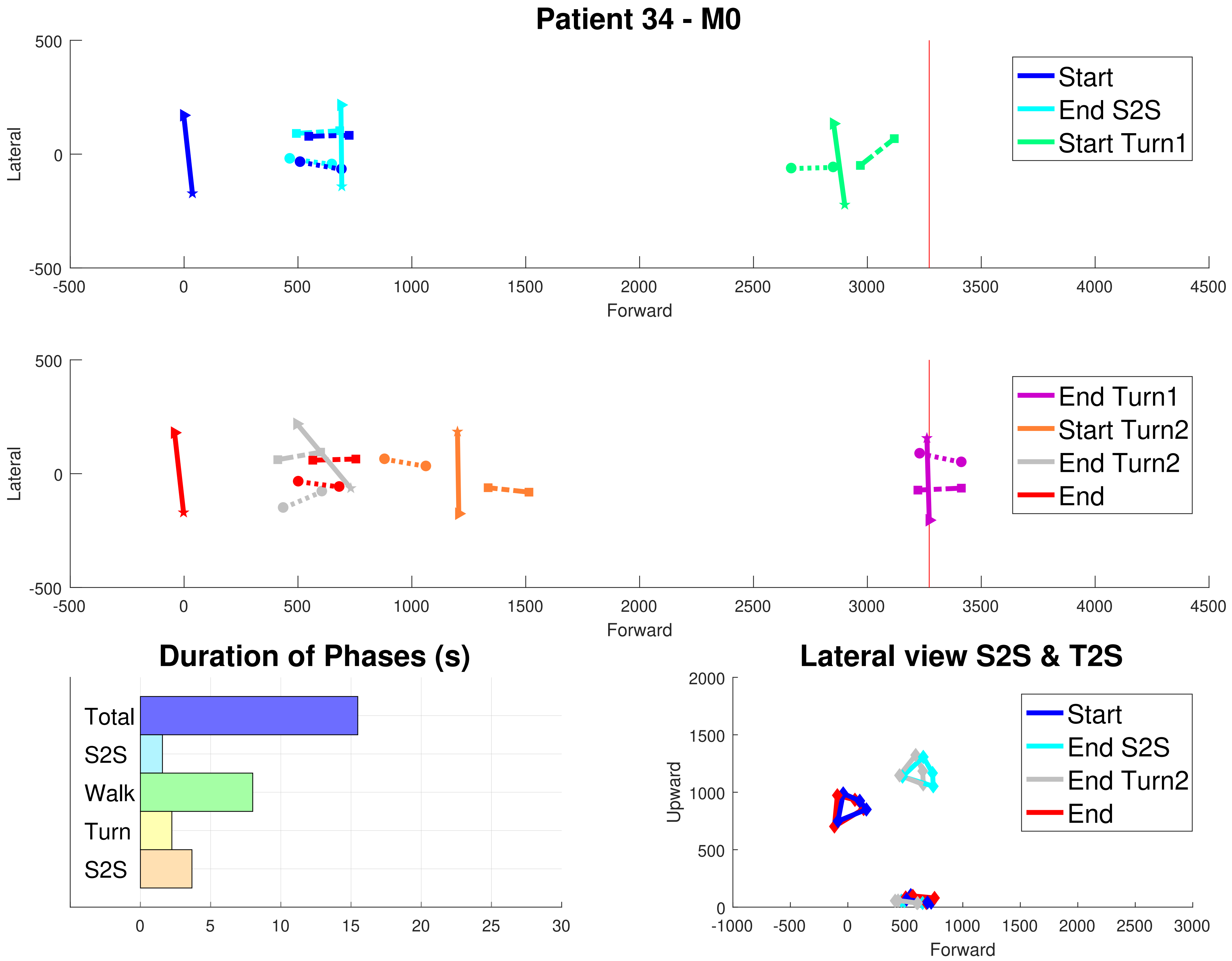

Patient 34 - M0
Lateral
Forward
Start
End S2S
Start Turn1
End Turn1
Start Turn2
End Turn2
End
Duration of Phases (s)
Total
S2S
Walk
Turn
S2S
Lateral view S2S & T2S
Upward

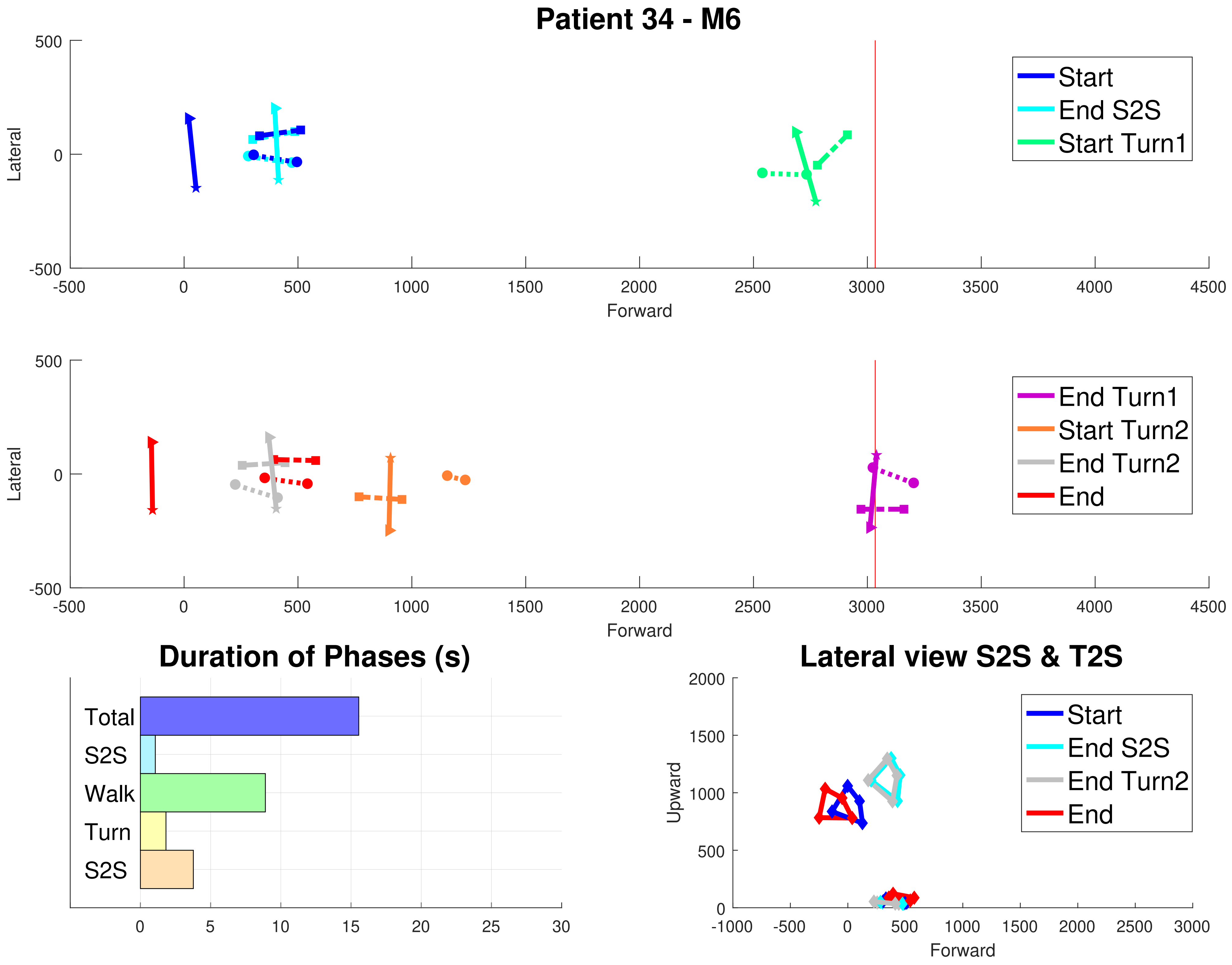

Patient 34 - M6
Lateral
Forward
500
0
-500
-500
0
500
1000
1500
2000
2500
3000
3500
4000
4500
Start
End S2S
Start Turn1
End Turn1
Start Turn2
End Turn2
End
Duration of Phases (s)
Total
S2S
Walk
Turn
S2S
0
5
10
15
20
25
30
Lateral view S2S & T2S
Upward
2000
1500
1000
500
-1000
-500
Start
End S2S
End Turn2
End

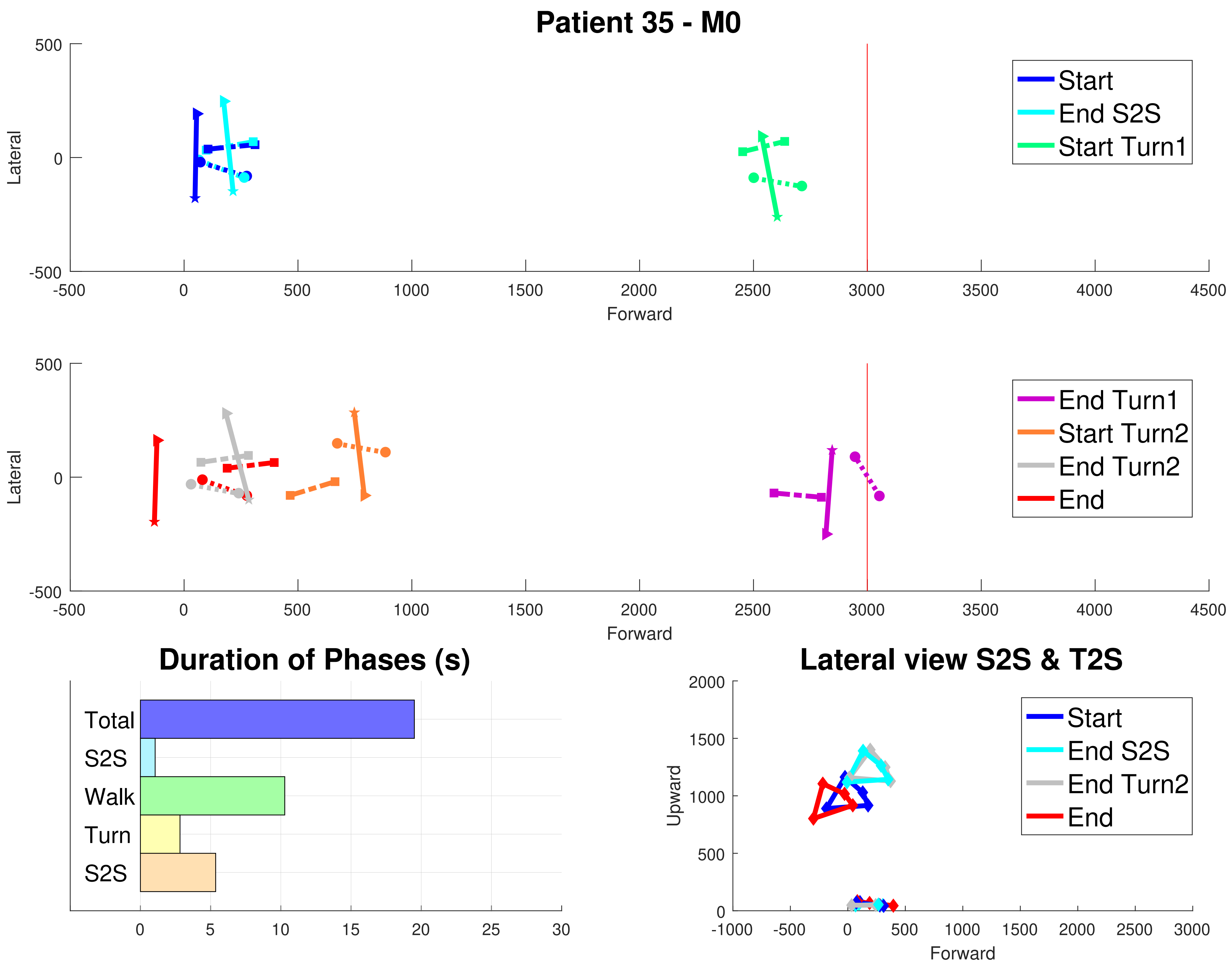

Patient 35 - M0
Lateral
Forward
500
0
-500
-500
0
500
1000
1500
2000
2500
3000
3500
4000
4500
Start
End S2S
Start Turn1
End Turn1
Start Turn2
End Turn2
End
Duration of Phases (s)
Total
S2S
Walk
Turn
S2S
0
5
10
15
20
25
30
Lateral view S2S & T2S
Upward
2000
1500
1000
500
-1000
-500
Start
End S2S
End Turn2
End

Patient 35 - M6

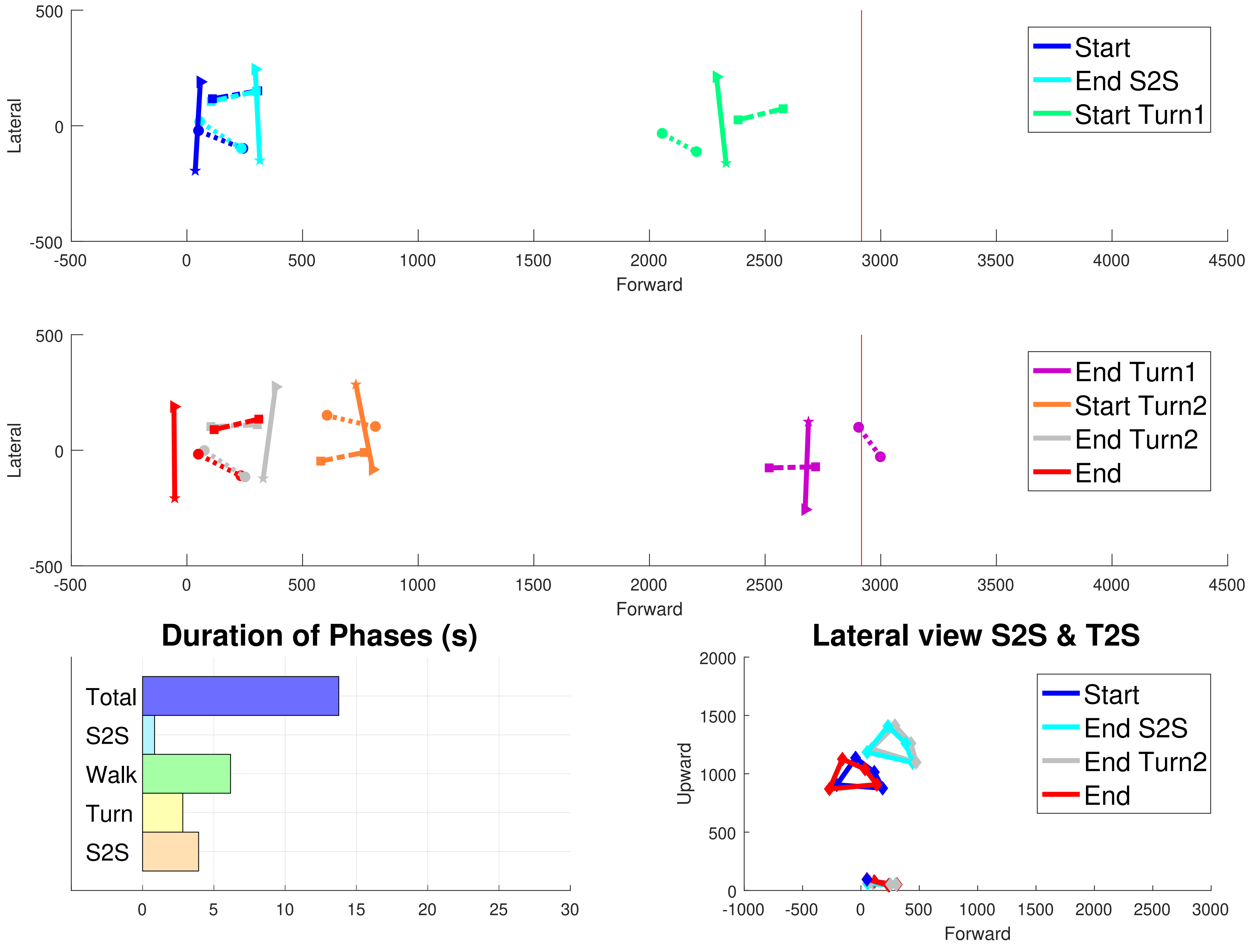

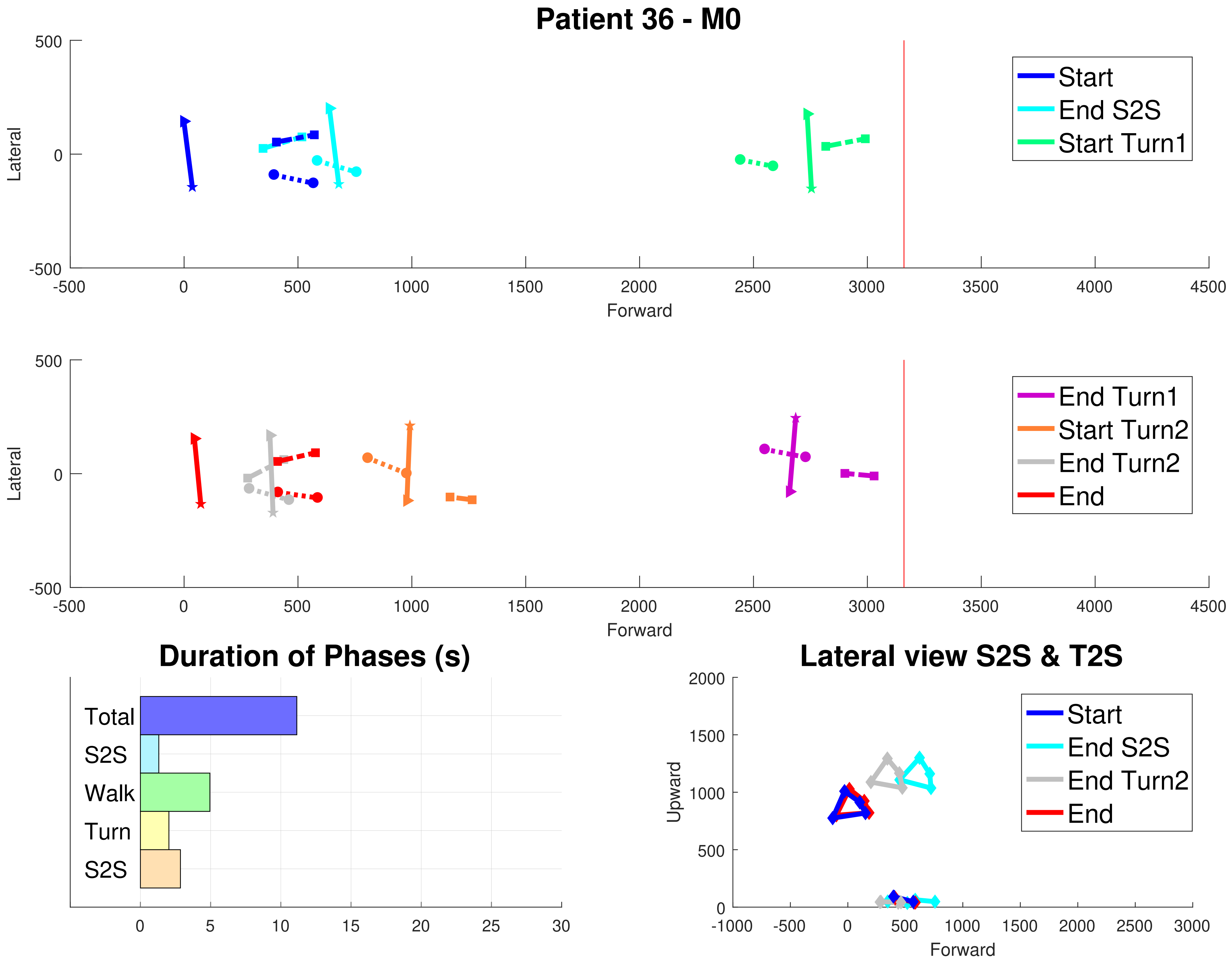

Patient 36 - M0
Lateral
Forward
Start
End S2S
Start Turn1
End Turn1
Start Turn2
End Turn2
End
Duration of Phases (s)
Total
S2S
Walk
Turn
S2S
Lateral view S2S & T2S
Upward
Forward
Start
End S2S
End Turn2
End

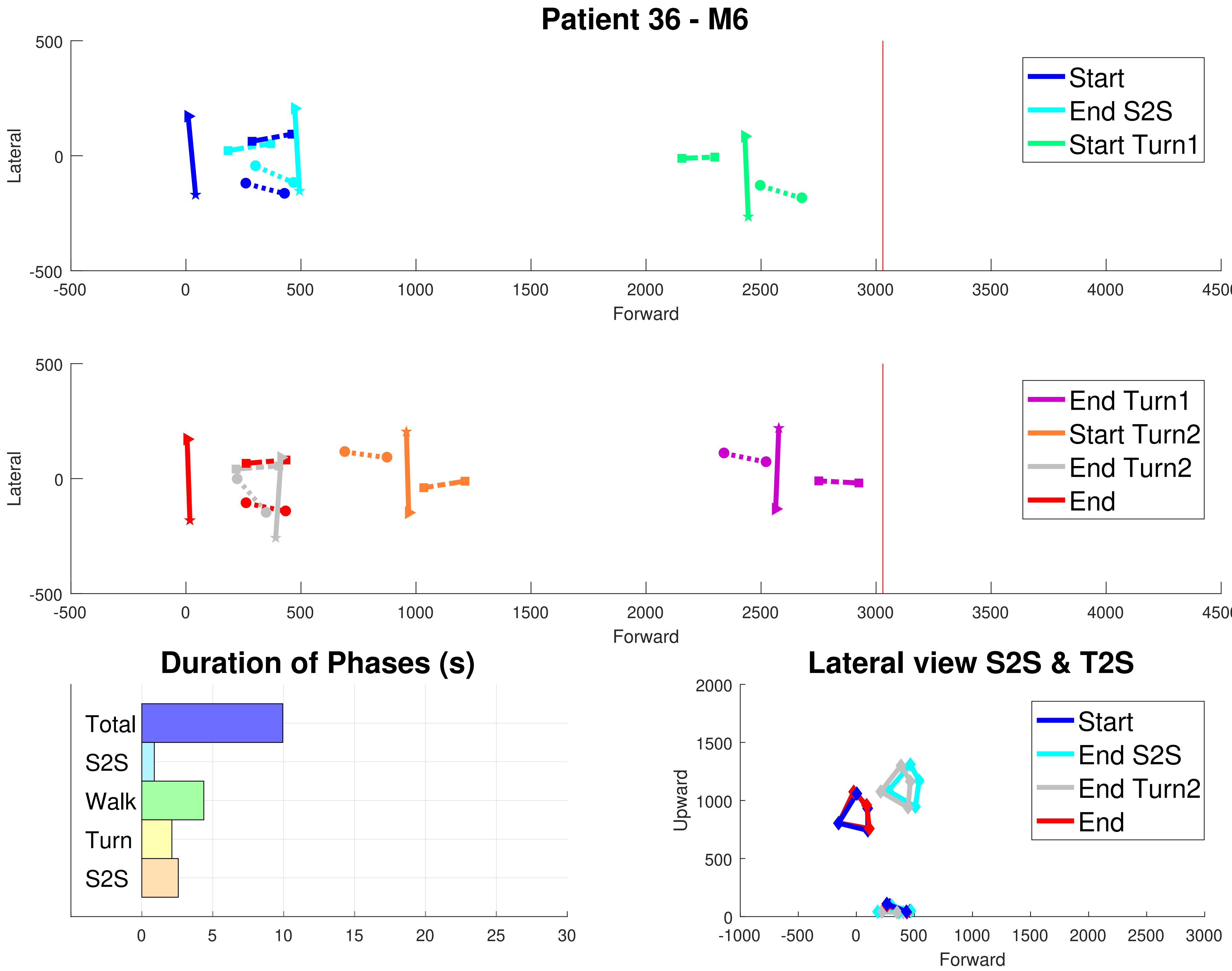
Patient 36 - M6
Start
End S2S
Start Turn1
Lateral
Forward
End Turn1
Start Turn2
End Turn2
End
Duration of Phases (s)
Total
S2S
Walk
Turn
S2S
Lateral view S2S & T2S
Upward
Forward
Start
End S2S
End Turn2
End

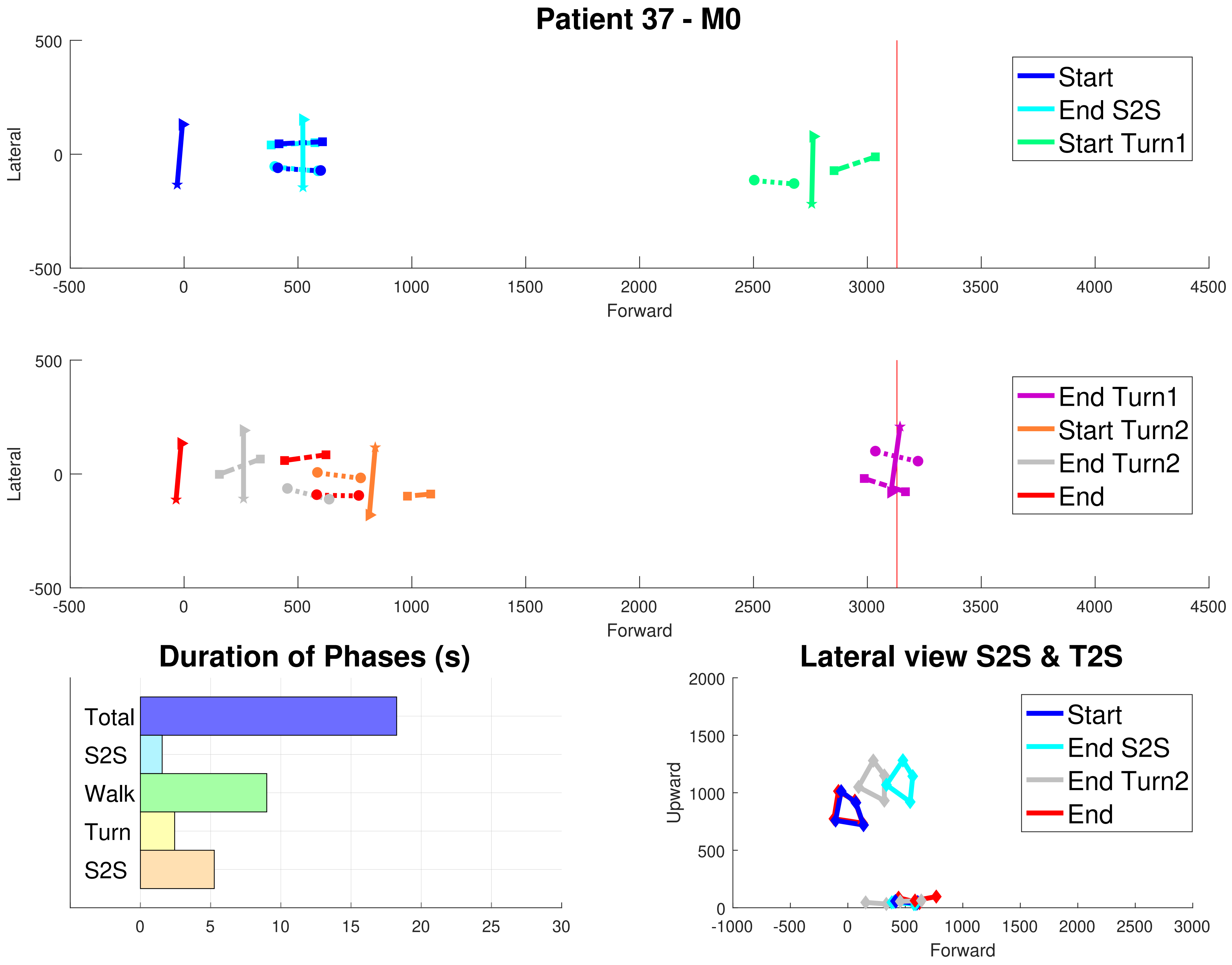

Patient 37 - M0
Lateral
Forward
Start
End S2S
Start Turn1
End Turn1
Start Turn2
End Turn2
End
500
0
-500
-500
0
500
1000
1500
2000
2500
3000
3500
4000
4500
Duration of Phases (s)
Total
S2S
Walk
Turn
S2S
0
5
10
15
20
25
30
Lateral view S2S & T2S
Upward
2000
1500
1000
500
0
-1000
-500
0
500
1000
1500
2000
2500
3000

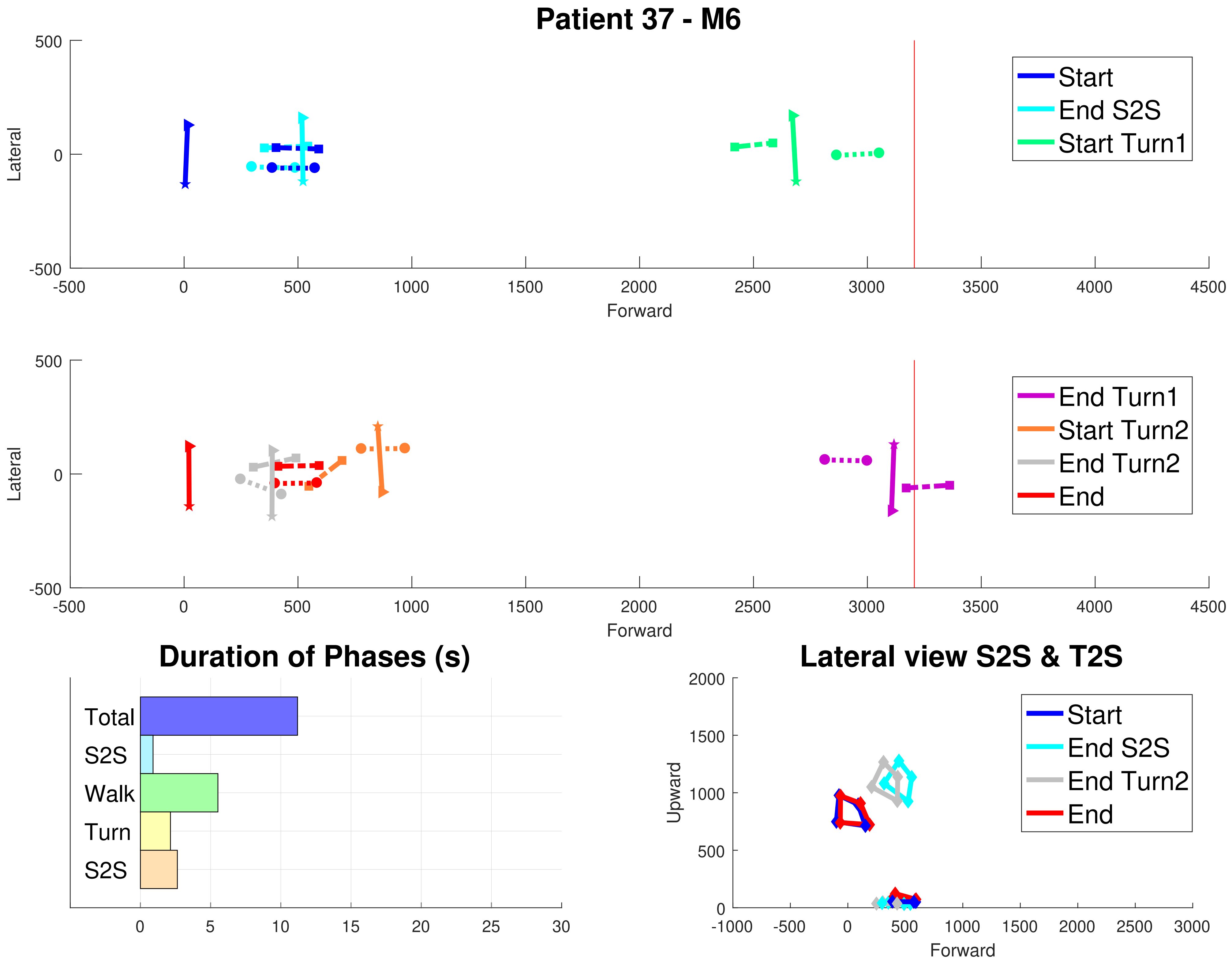

Patient 37 - M6
Lateral
Forward
Start
End S2S
Start Turn1
End Turn1
Start Turn2
End Turn2
End
Duration of Phases (s)
Total
S2S
Walk
Turn
S2S
Lateral view S2S & T2S
Upward
Forward
Start
End S2S
End Turn2
End

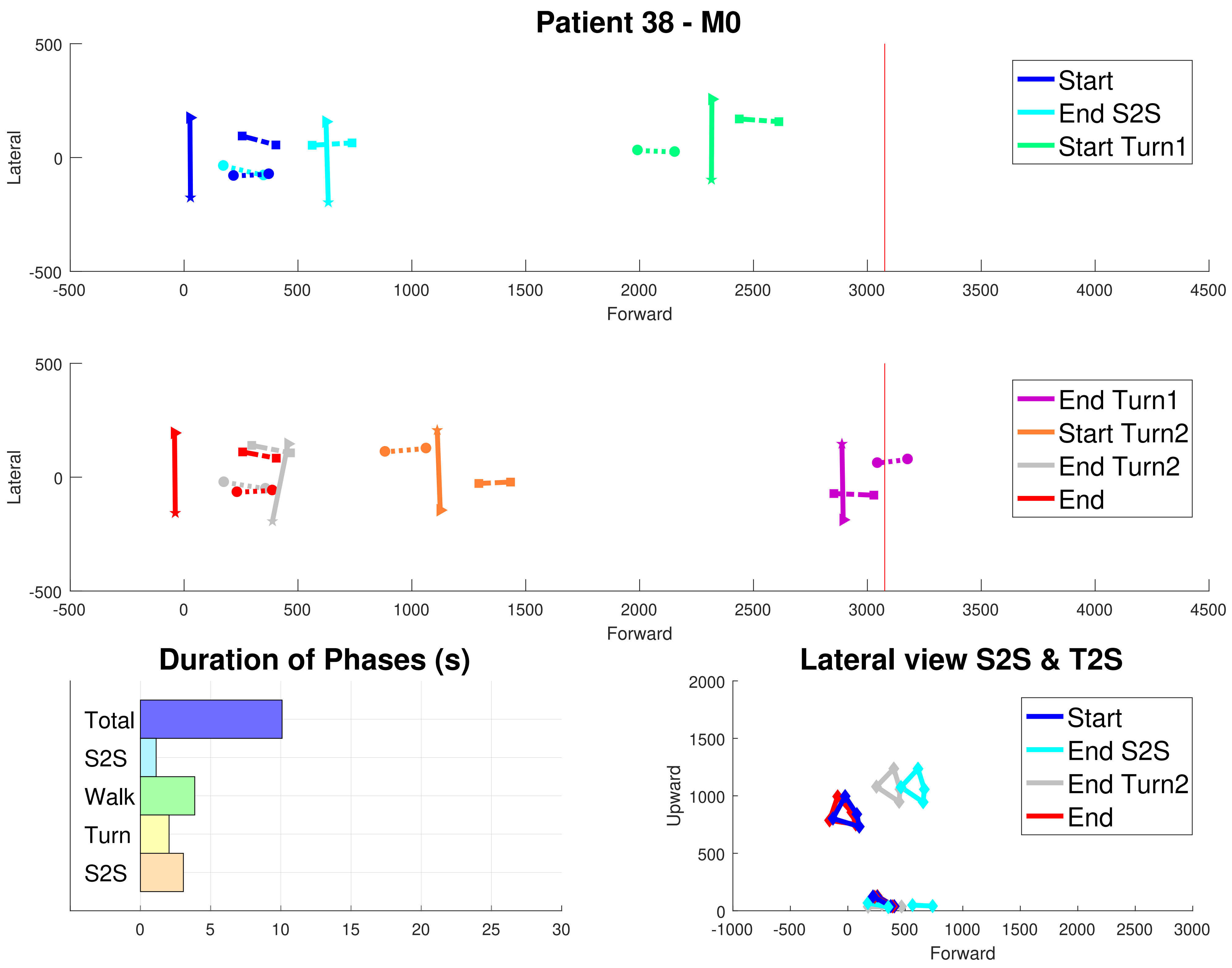
Patient 38 - M0
Lateral
Forward
Start
End S2S
Start Turn1
End Turn1
Start Turn2
End Turn2
End
Duration of Phases (s)
Total
S2S
Walk
Turn
S2S
Lateral view S2S & T2S
Upward
Forward
Start
End S2S
End Turn2
End

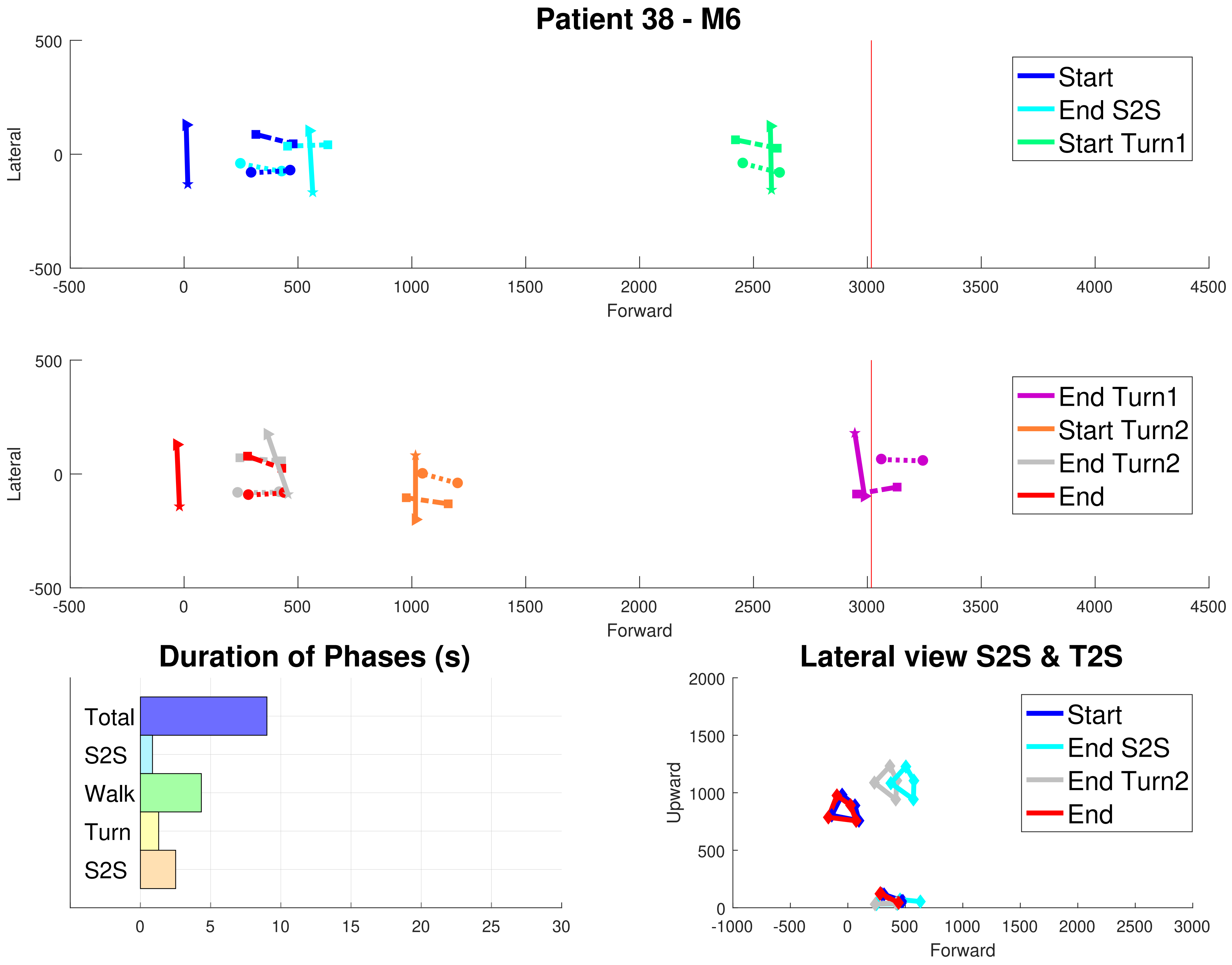
Patient 38 - M6
Lateral
Forward
Start
End S2S
Start Turn1
End Turn1
Start Turn2
End Turn2
End
Duration of Phases (s)
Total
S2S
Walk
Turn
S2S
Lateral view S2S & T2S
Upward
Forward
Start
End S2S
End Turn2
End

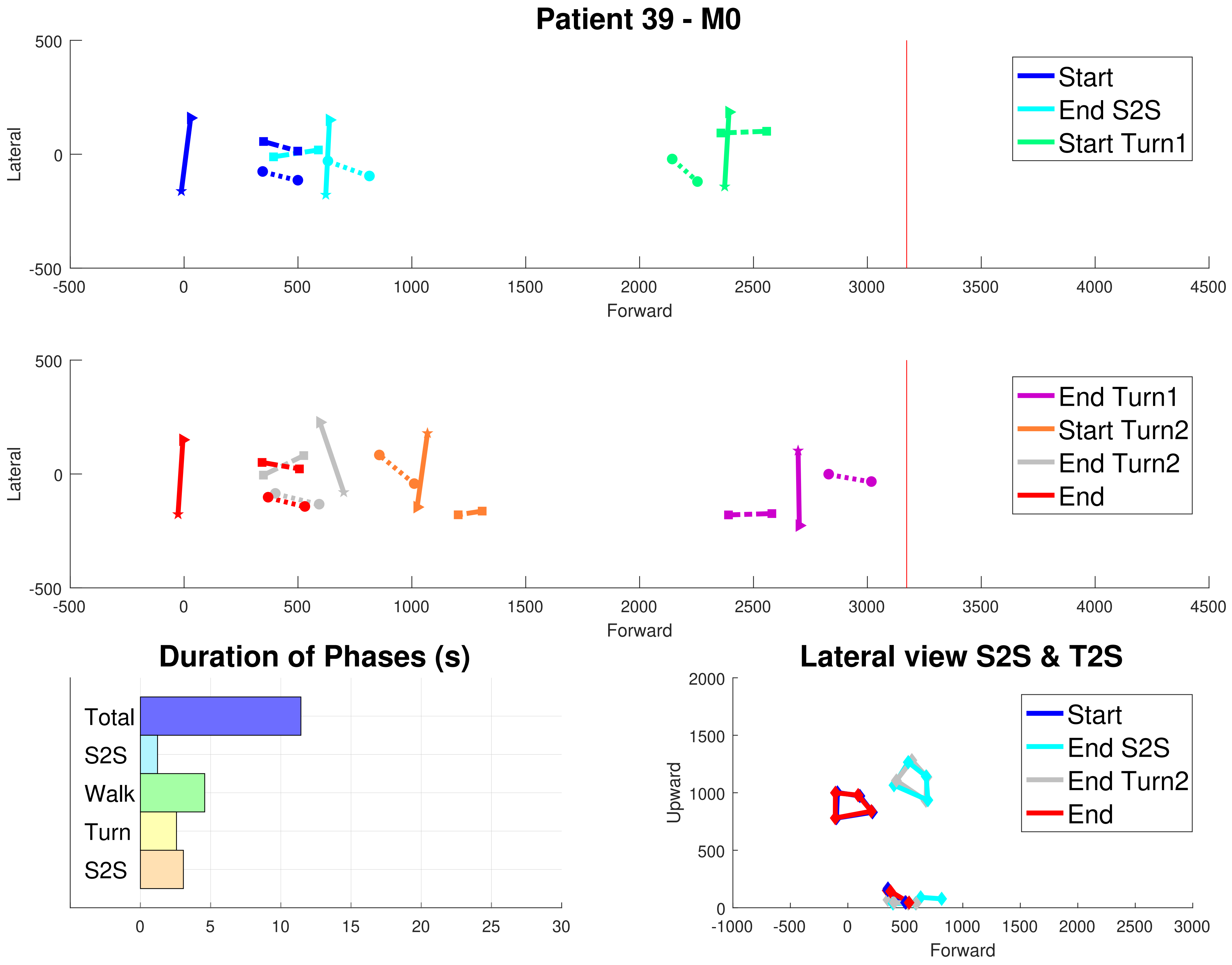
Patient 39 - M0
Lateral
Forward
Start
End S2S
Start Turn1
End Turn1
Start Turn2
End Turn2
End
Duration of Phases (s)
Total
S2S
Walk
Turn
S2S
Lateral view S2S & T2S
Upward
Forward
Start
End S2S
End Turn2
End

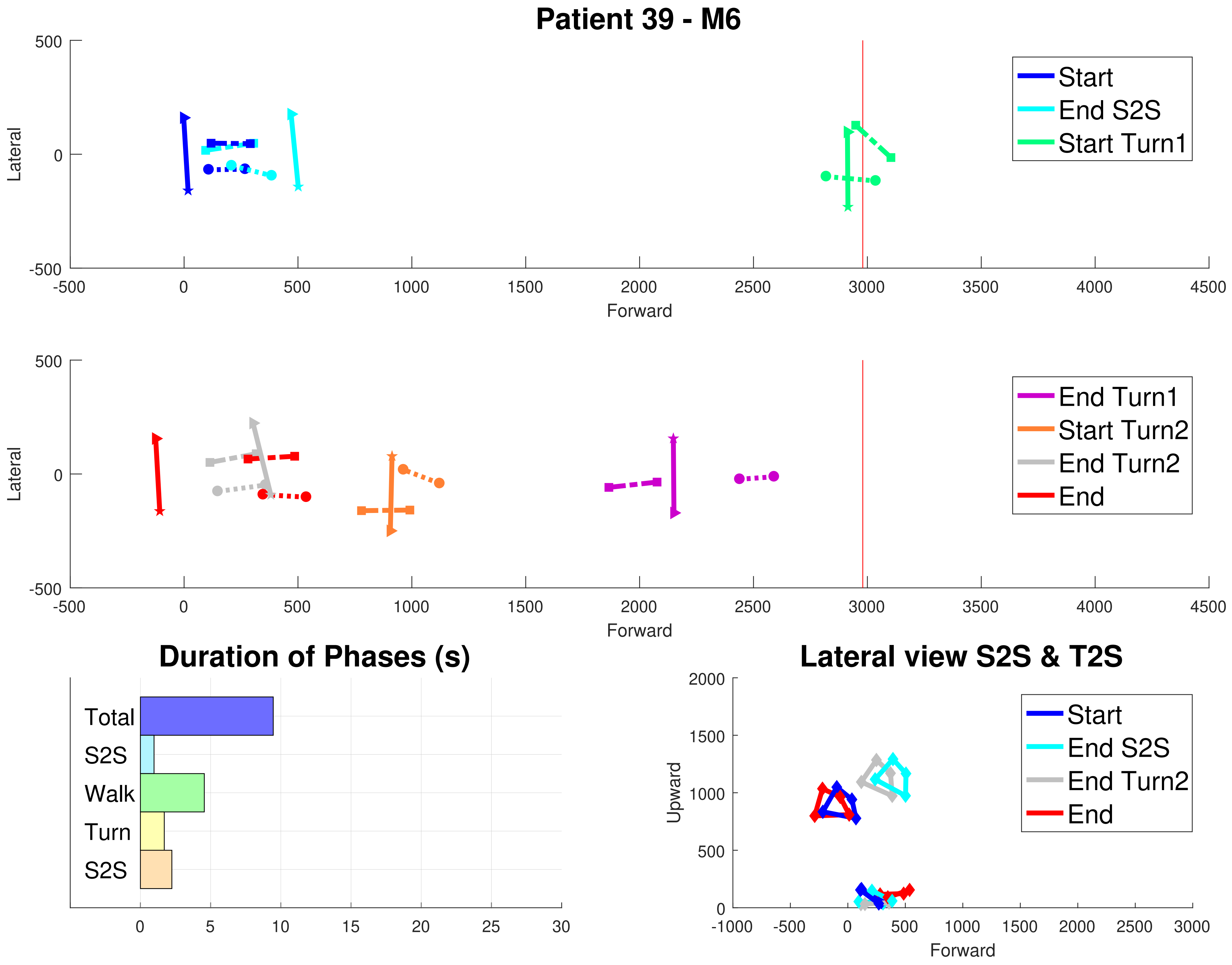

Patient 39 - M6
Lateral
Forward
Start
End S2S
Start Turn1
End Turn1
Start Turn2
End Turn2
End
Duration of Phases (s)
Total
S2S
Walk
Turn
S2S
Lateral view S2S & T2S
Upward
Start
End S2S
End Turn2
End

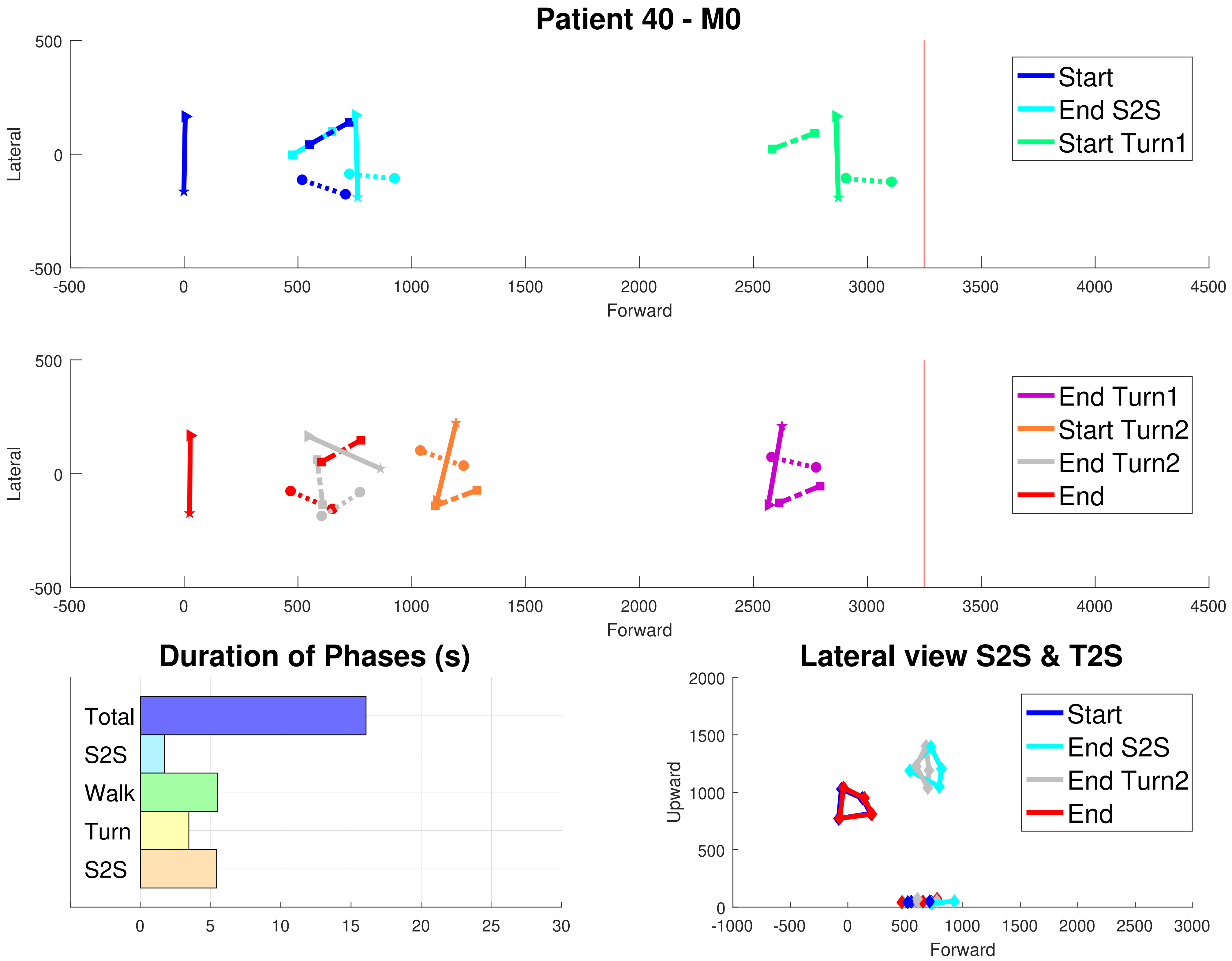
Patient 40 - M0
Lateral
Forward
Start
End S2S
Start Turn1
End Turn1
Start Turn2
End Turn2
End
Duration of Phases (s)
Total
S2S
Walk
Turn
S2S
Lateral view S2S & T2S
Upward
Forward
Start
End S2S
End Turn2
End

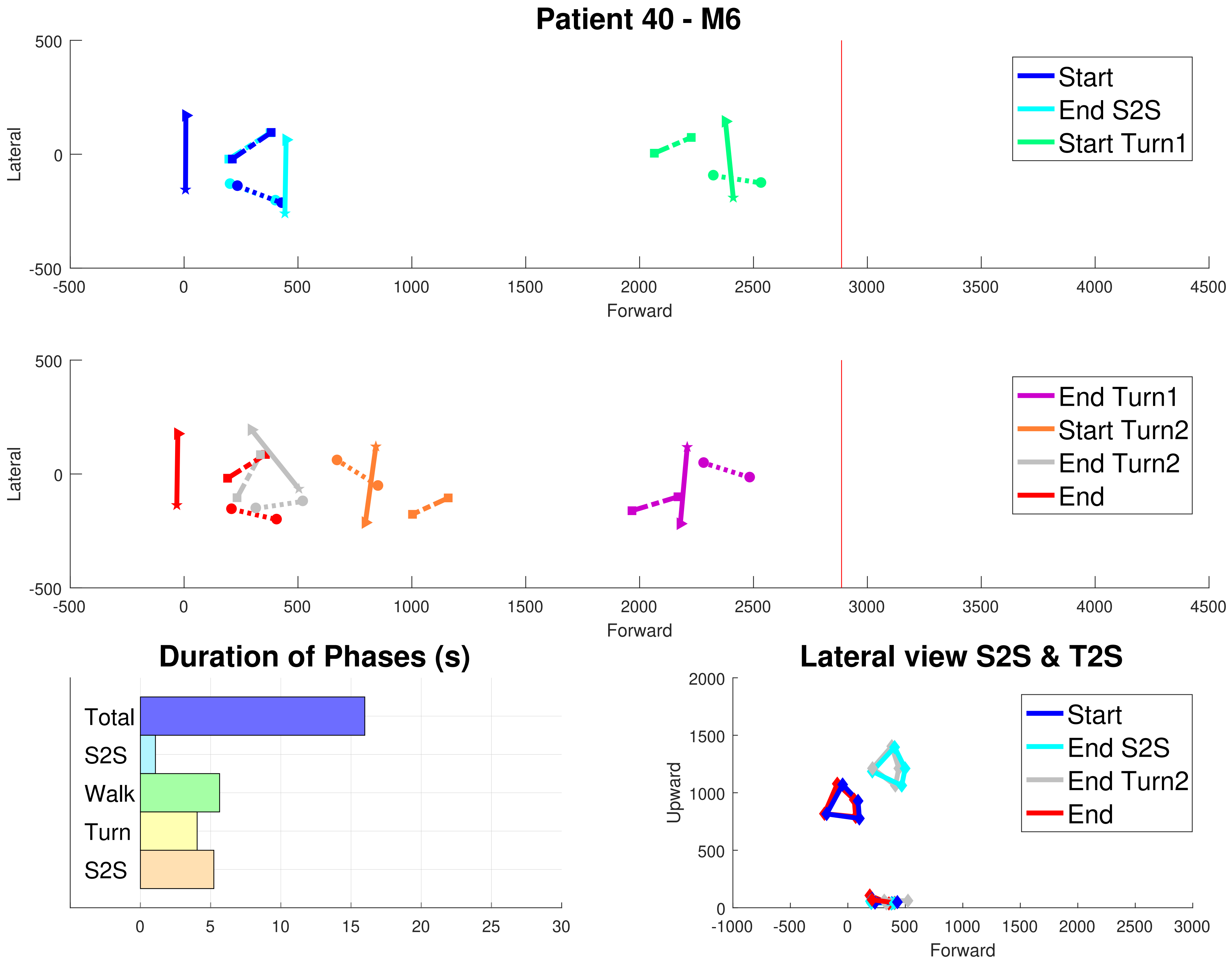

Patient 40 - M6
Lateral
Forward
Start
End S2S
Start Turn1
End Turn1
Start Turn2
End Turn2
End
Duration of Phases (s)
Total
S2S
Walk
Turn
S2S
Lateral view S2S & T2S
Upward

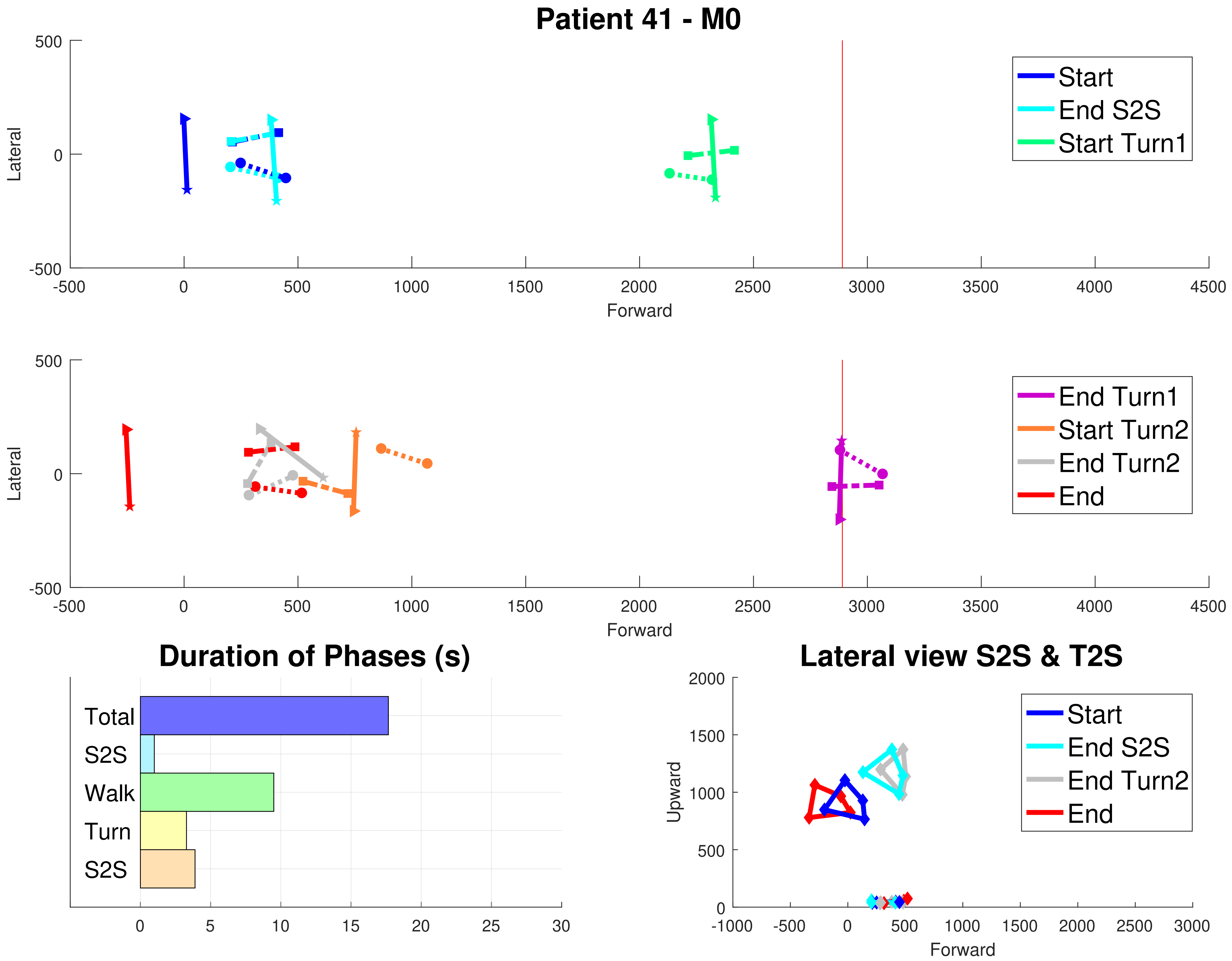

Patient 41 - M0
Lateral
Forward
Start
End S2S
Start Turn1
End Turn1
Start Turn2
End Turn2
End
Duration of Phases (s)
Total
S2S
Walk
Turn
S2S
Lateral view S2S & T2S
Upward
Forward
Start
End S2S
End Turn2
End

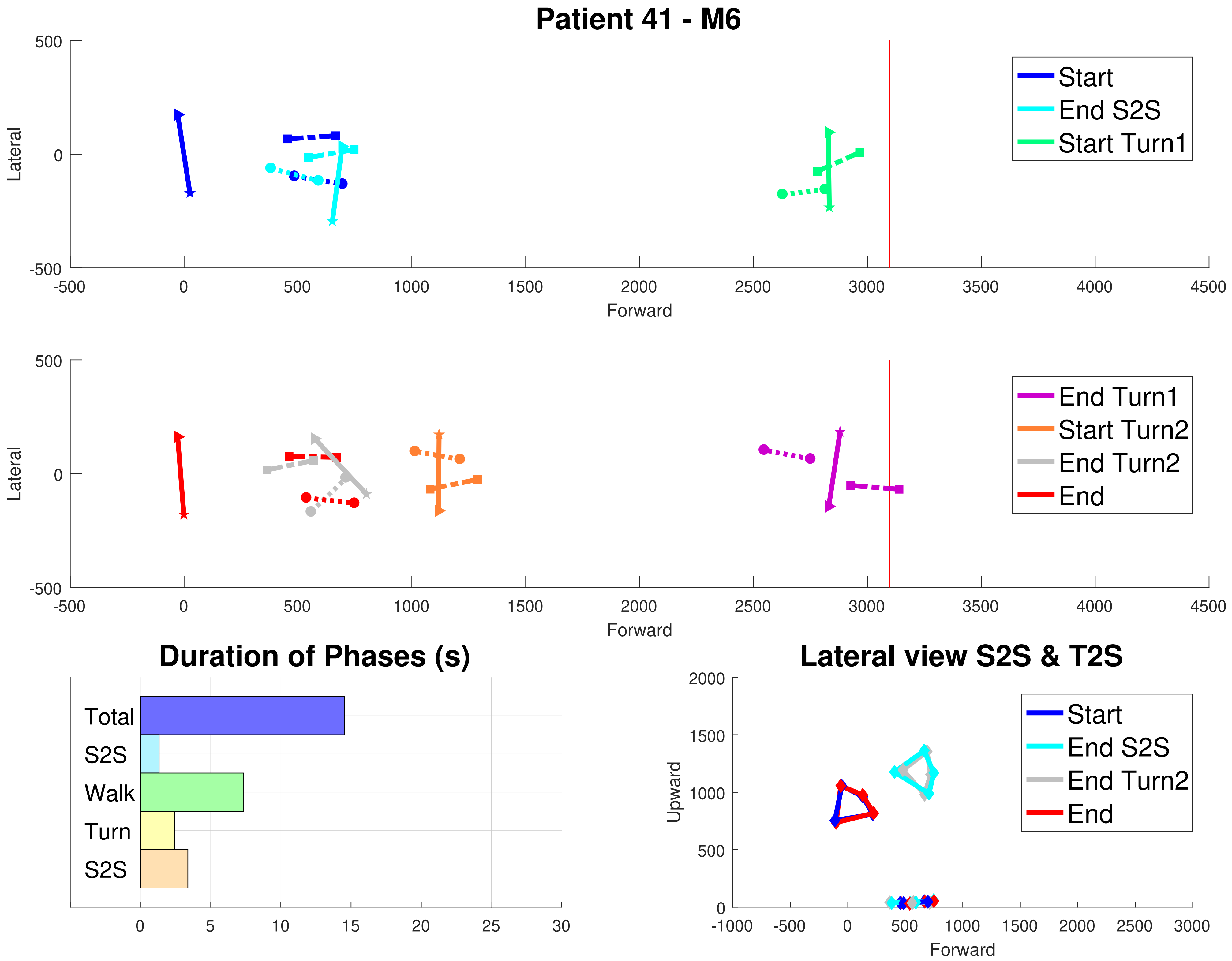

Patient 41 - M6
Lateral
Forward
Start
End S2S
Start Turn1
End Turn1
Start Turn2
End Turn2
End
Duration of Phases (s)
Total
S2S
Walk
Turn
S2S
Lateral view S2S & T2S
Upward

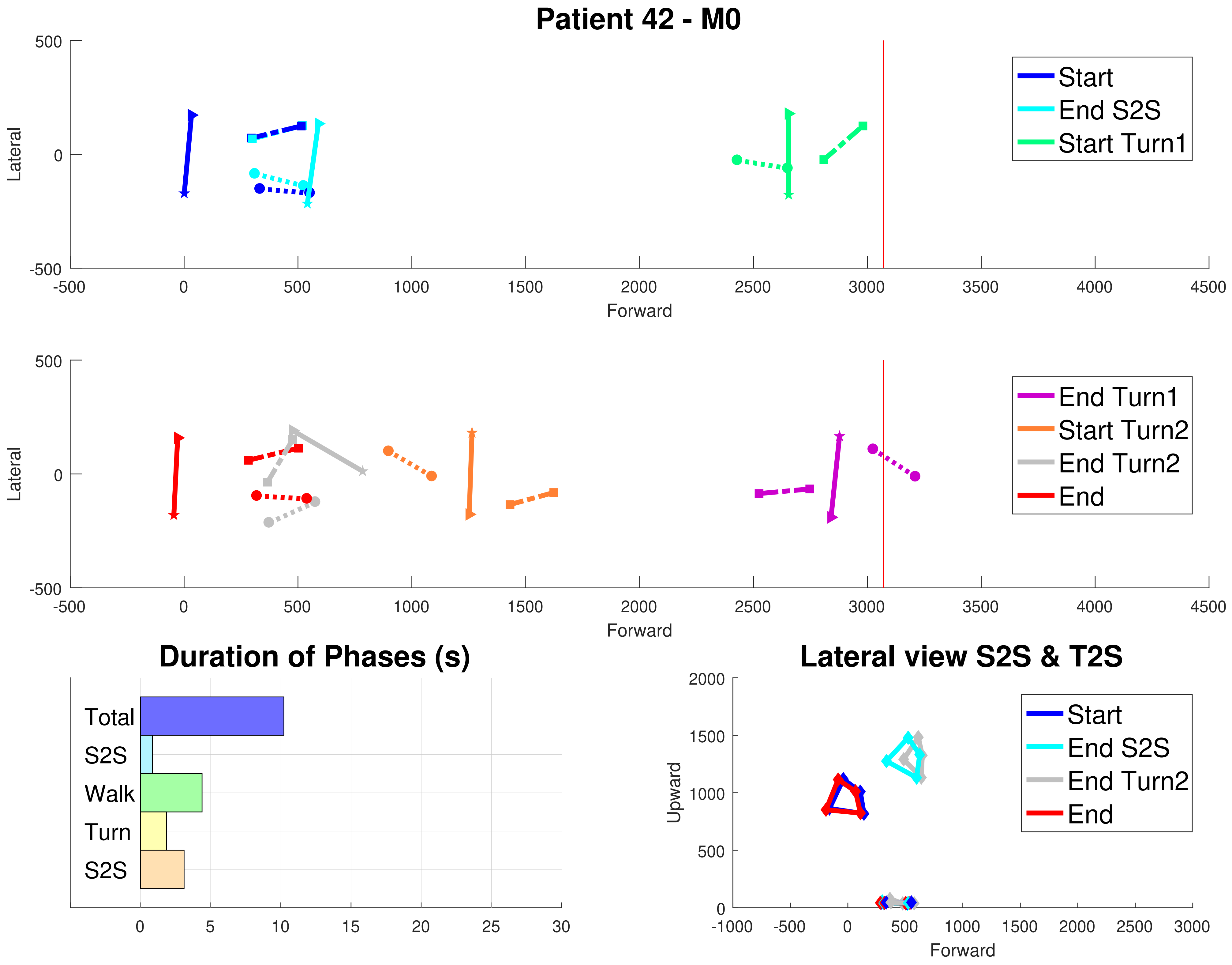

Patient 42 - M0
Lateral
Forward
Start
End S2S
Start Turn1
End Turn1
Start Turn2
End Turn2
End
Duration of Phases (s)
Total
S2S
Walk
Turn
S2S
Lateral view S2S & T2S
Upward
Forward
Start
End S2S
End Turn2
End

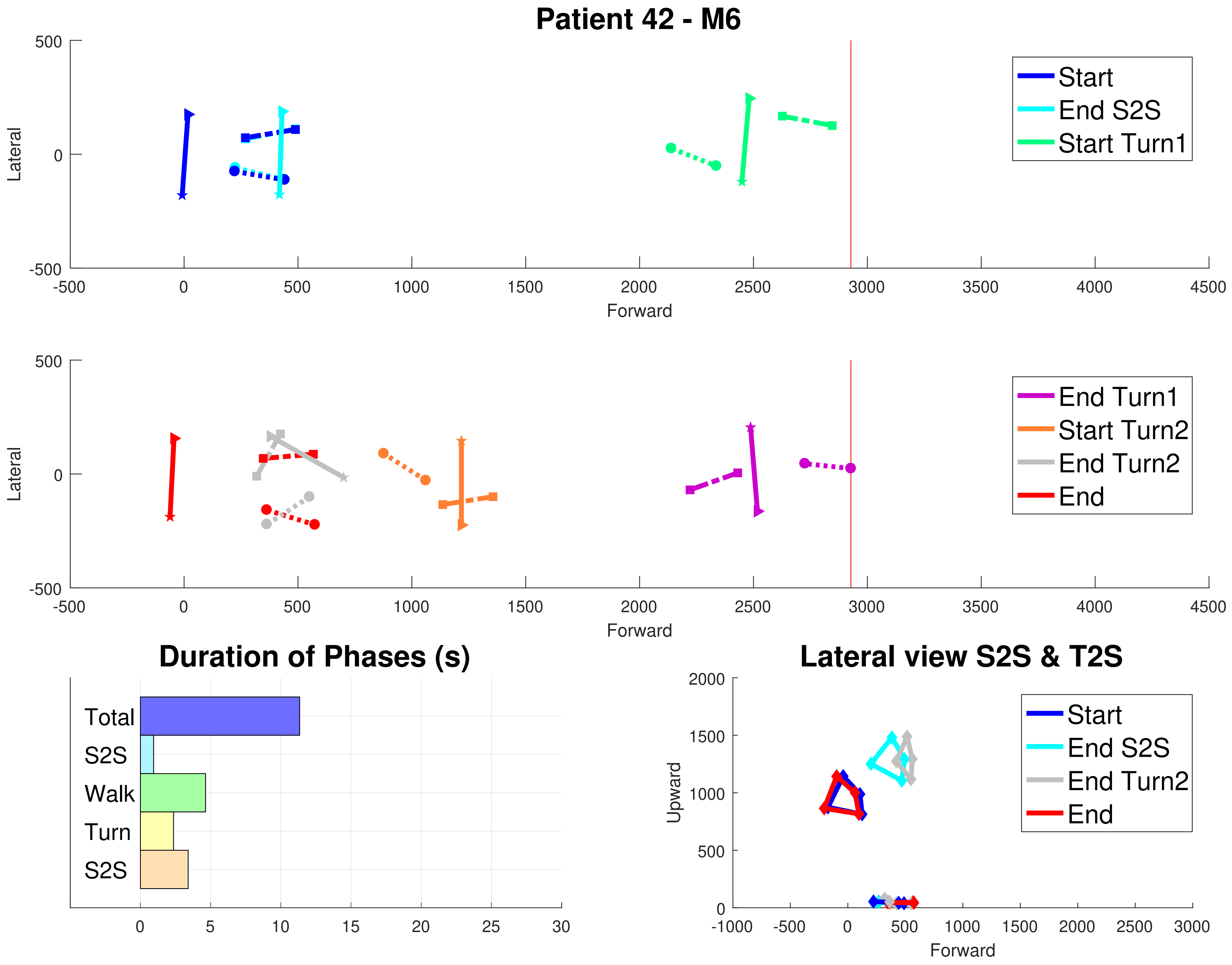
Patient 42 - M6
Lateral
Forward
500
0
-500
-500
0
500
1000
1500
2000
2500
3000
3500
4000
4500
Start
End S2S
Start Turn1
End Turn1
Start Turn2
End Turn2
End
Duration of Phases (s)
Total
S2S
Walk
Turn
S2S
0
5
10
15
20
25
30
Lateral view S2S & T2S
Upward
2000
1500
1000
500
-1000
-500
Start
End S2S
End Turn2
End

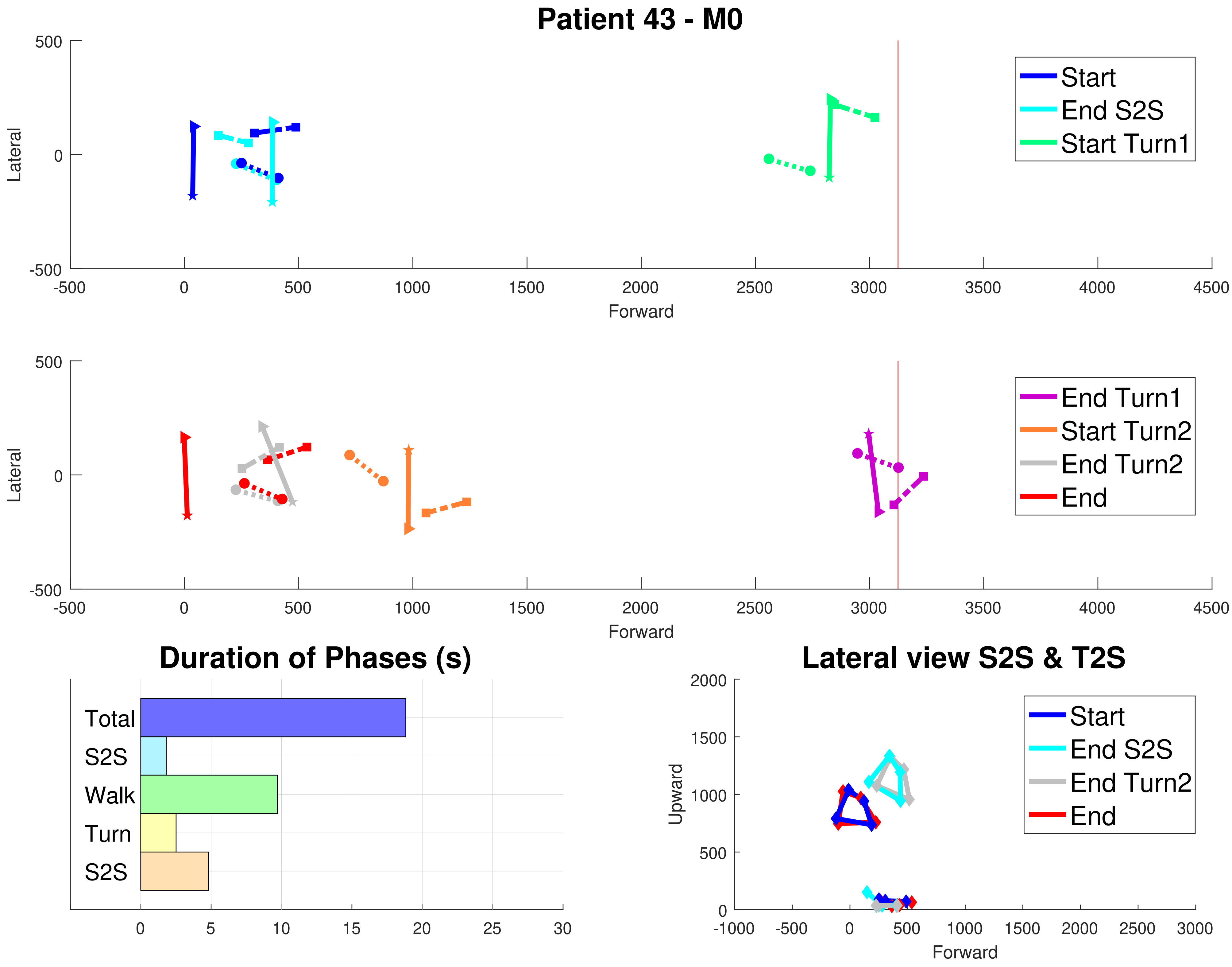

Patient 43 - M0
Lateral
Forward
Start
End S2S
Start Turn1
End Turn1
Start Turn2
End Turn2
End
Duration of Phases (s)
Total
S2S
Walk
Turn
S2S
Lateral view S2S & T2S
Upward
Forward
Start
End S2S
End Turn2
End

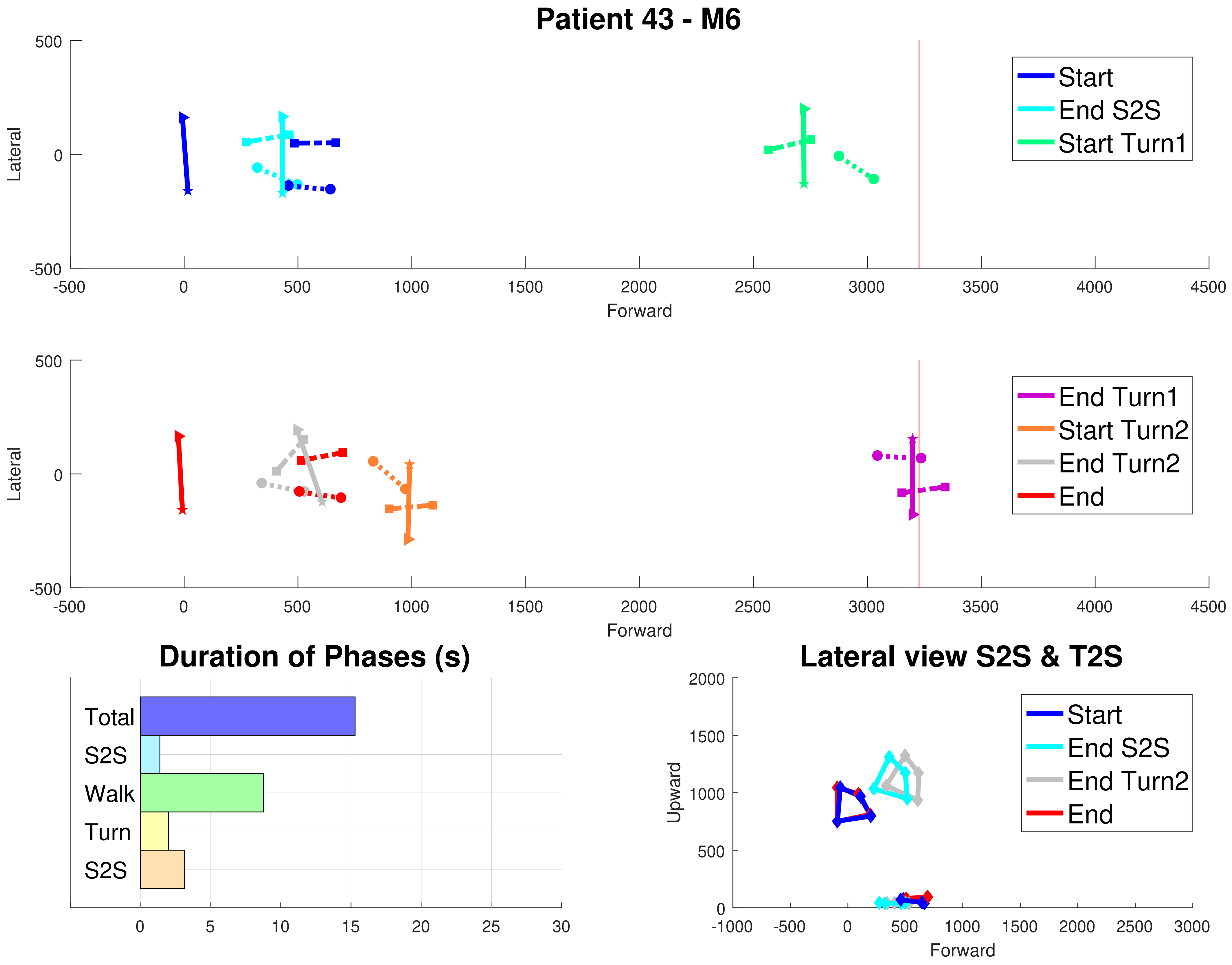

Patient 43 - M6
Lateral
Forward
Start
End S2S
Start Turn1
End Turn1
Start Turn2
End Turn2
End
Duration of Phases (s)
Total
S2S
Walk
Turn
S2S
Lateral view S2S & T2S
Upward
Forward
Start
End S2S
End Turn2
End

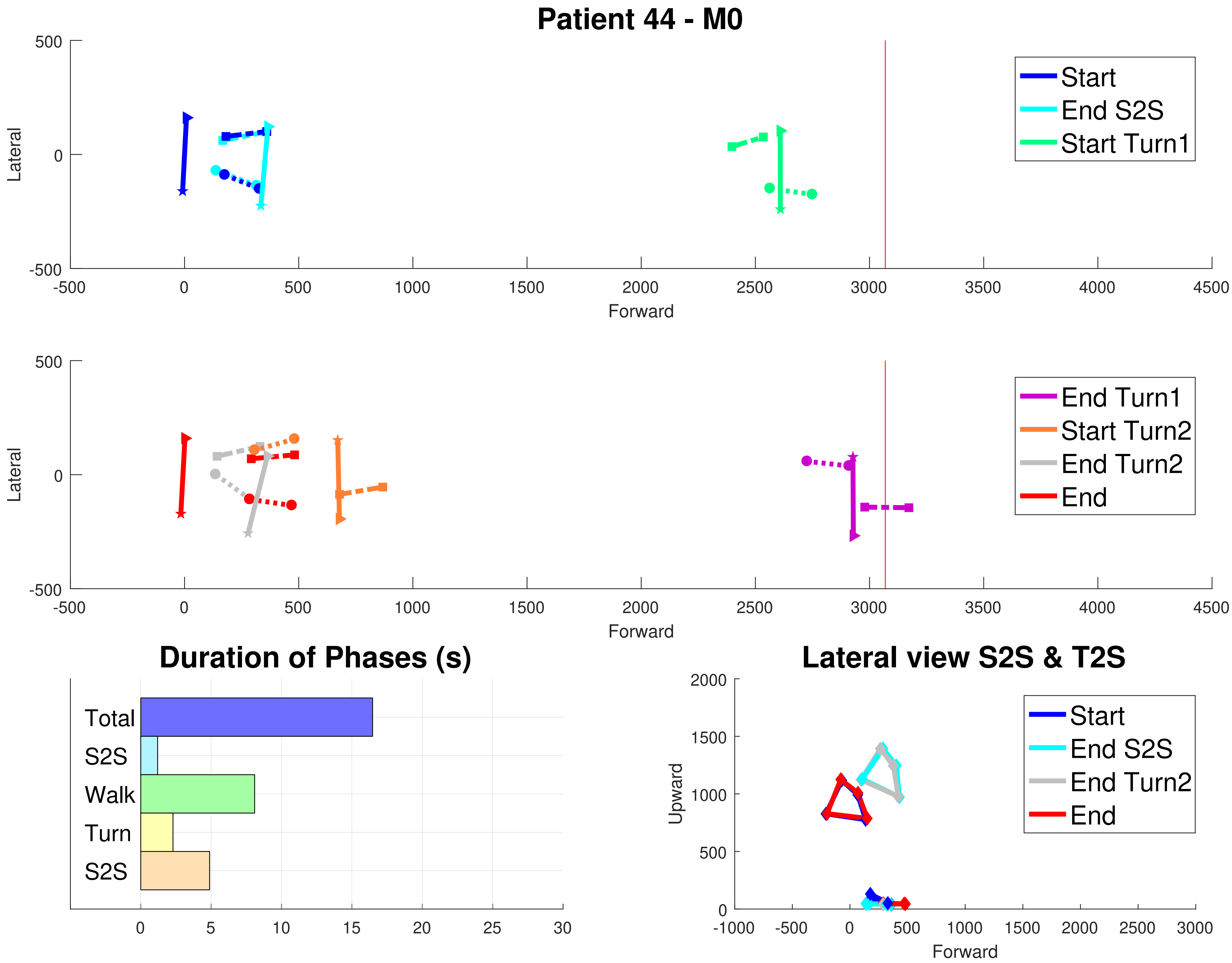

Patient 44 - M0
Lateral
Forward
Start
End S2S
Start Turn1
End Turn1
Start Turn2
End Turn2
End
Duration of Phases (s)
Total
S2S
Walk
Turn
S2S
Lateral view S2S & T2S
Upward
Forward
Start
End S2S
End Turn2
End

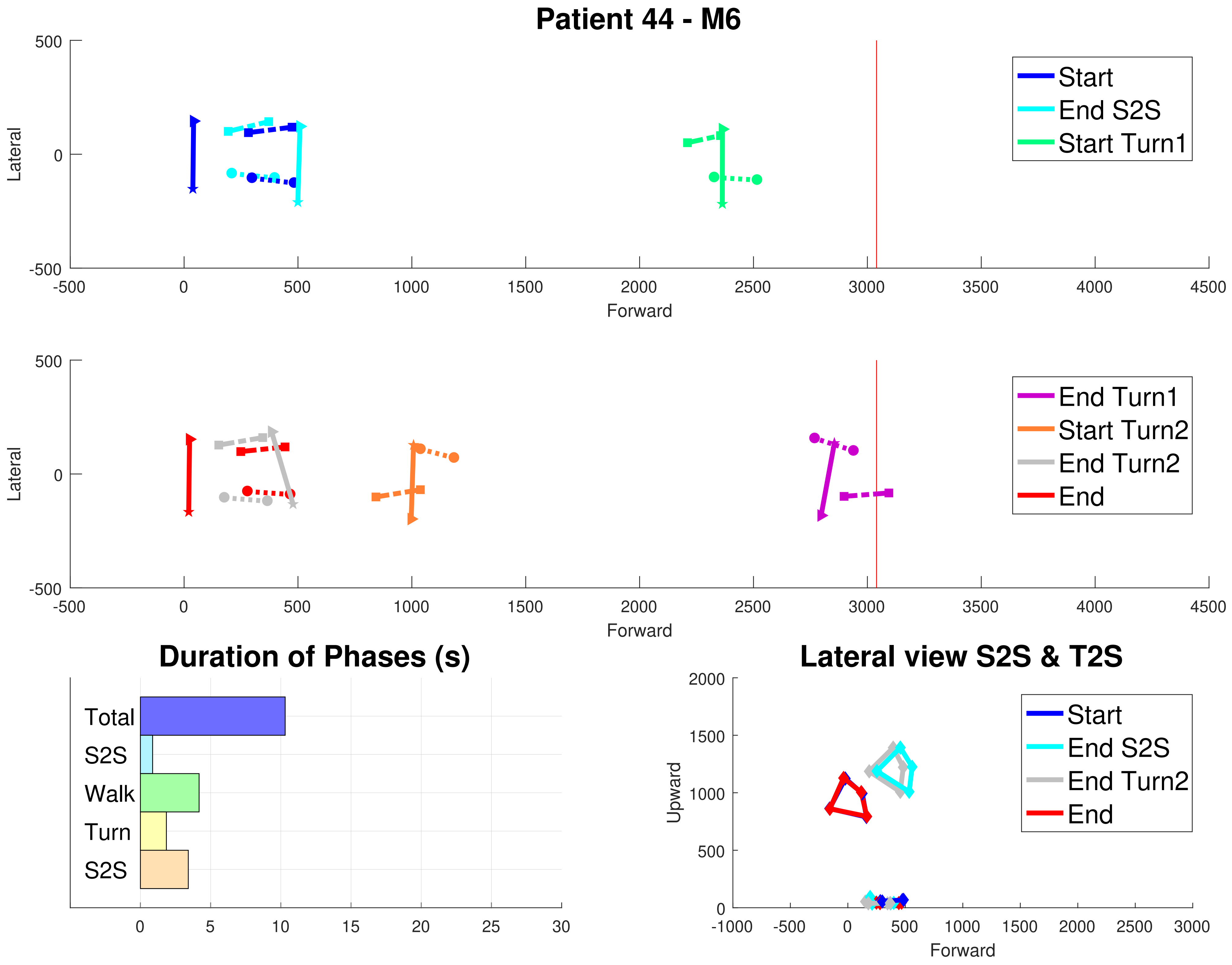

Patient 44 - M6
Lateral
Forward
Start
End S2S
Start Turn1
End Turn1
Start Turn2
End Turn2
End
Duration of Phases (s)
Total
S2S
Walk
Turn
S2S
Lateral view S2S & T2S
Upward

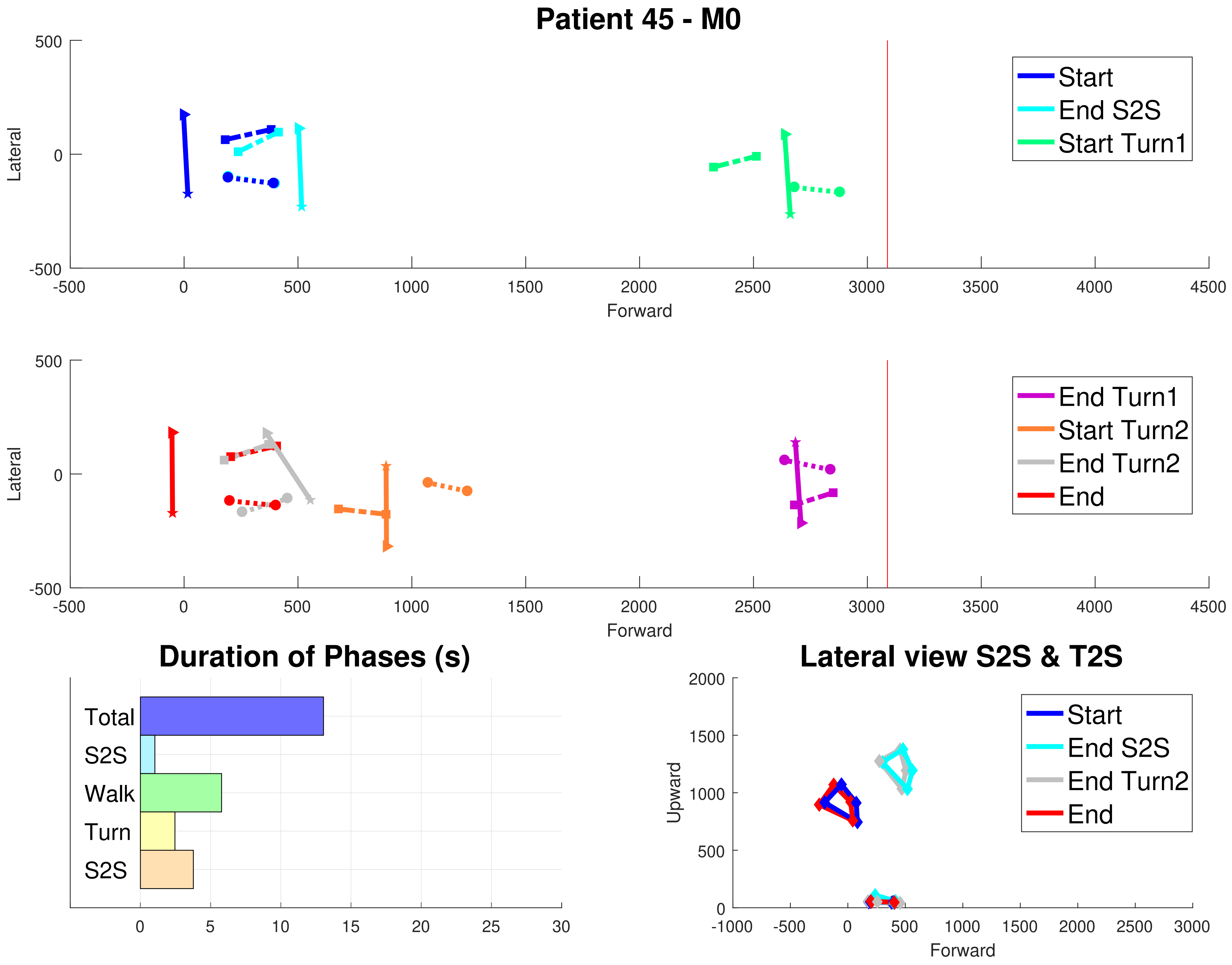

Patient 45 - M0
Lateral
Forward
Start
End S2S
Start Turn1
End Turn1
Start Turn2
End Turn2
End
Duration of Phases (s)
Total
S2S
Walk
Turn
S2S
Lateral view S2S & T2S
Upward

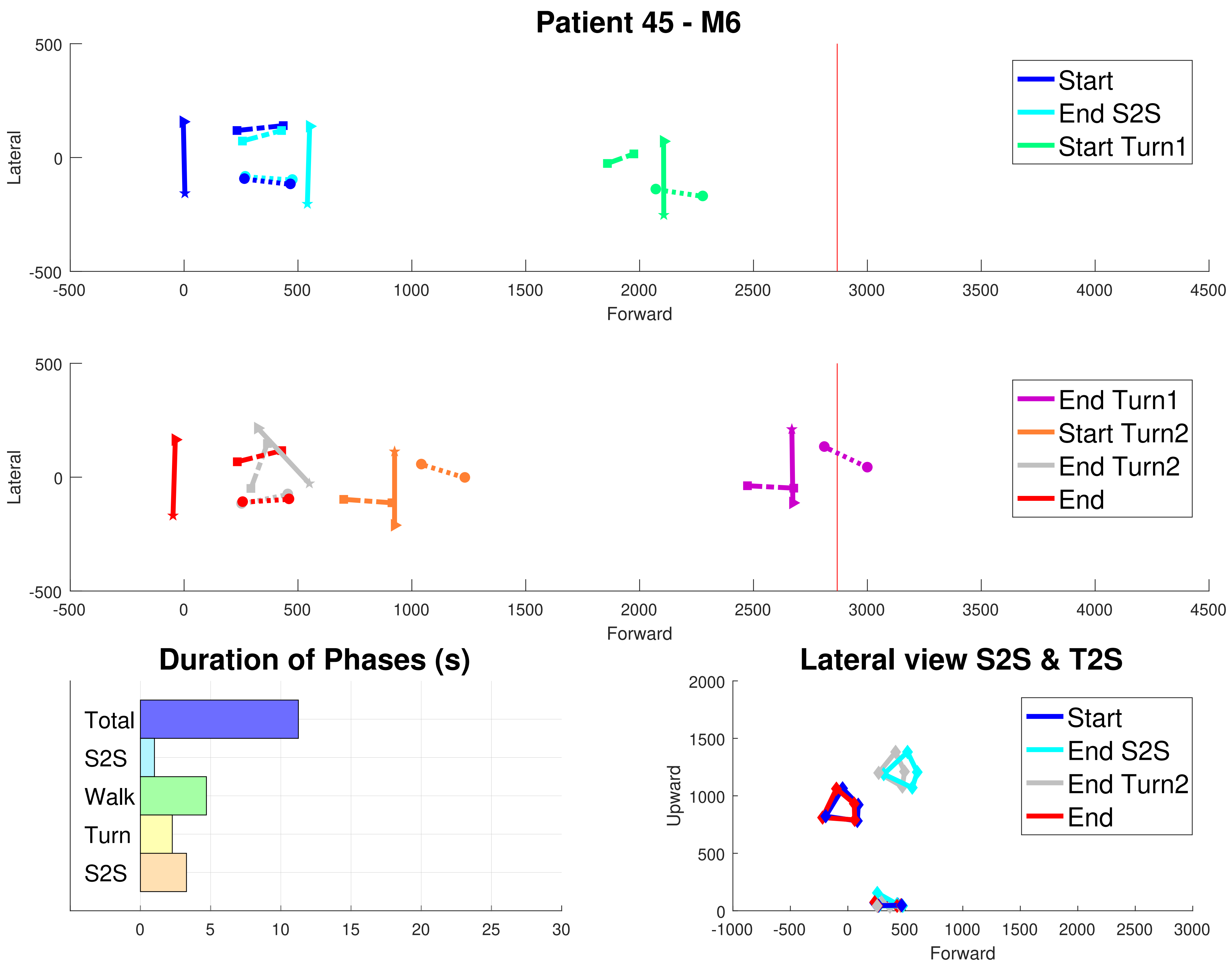
Patient 45 - M6
Lateral
Forward
Start
End S2S
Start Turn1
End Turn1
Start Turn2
End Turn2
End
Duration of Phases (s)
Total
S2S
Walk
Turn
S2S
Lateral view S2S & T2S
Upward
Forward
Start
End S2S
End Turn2
End

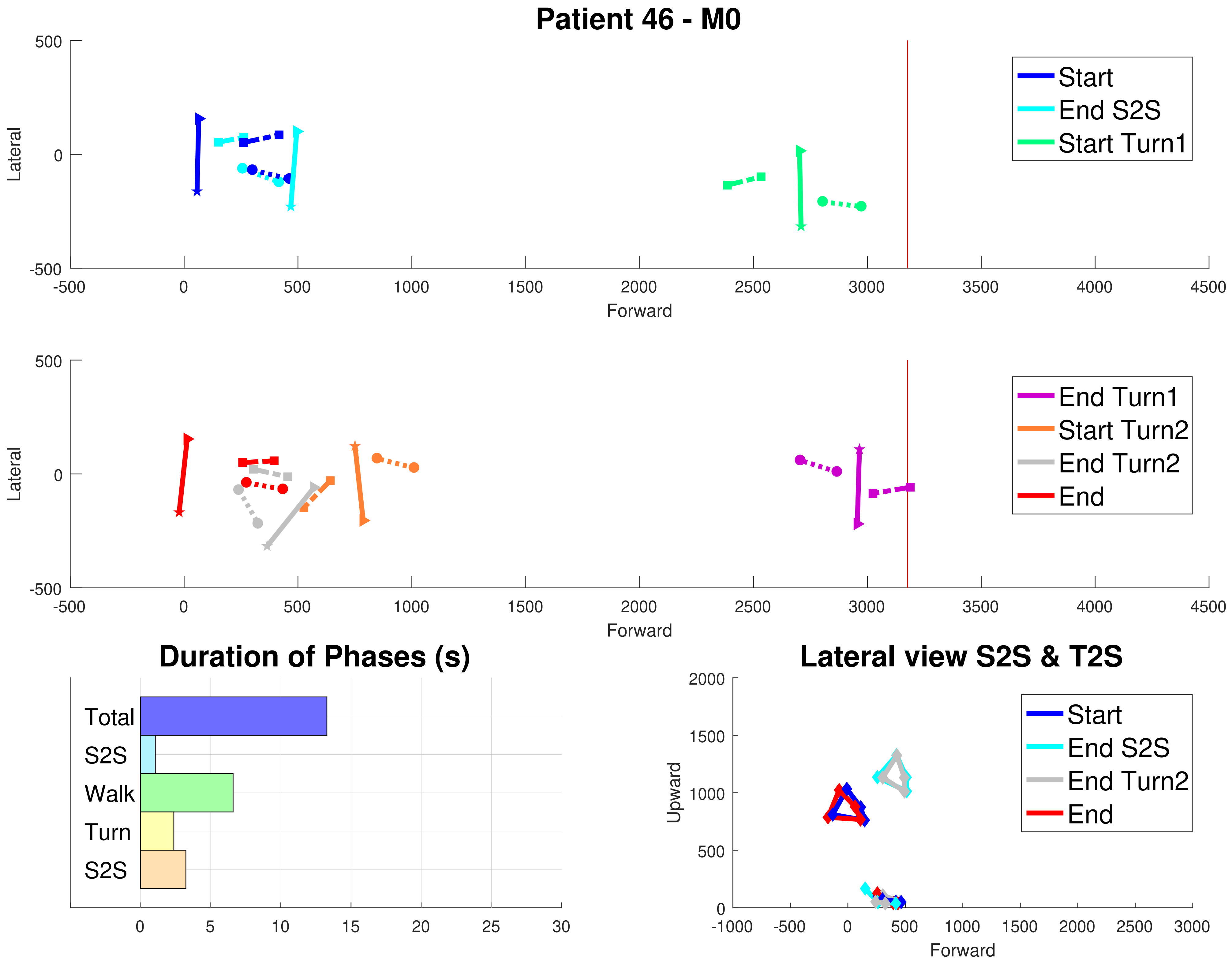
Patient 46 - M0
Lateral
Forward
Start
End S2S
Start Turn1
End Turn1
Start Turn2
End Turn2
End
Duration of Phases (s)
Total
S2S
Walk
Turn
S2S
Lateral view S2S & T2S
Upward
Forward
Start
End S2S
End Turn2
End

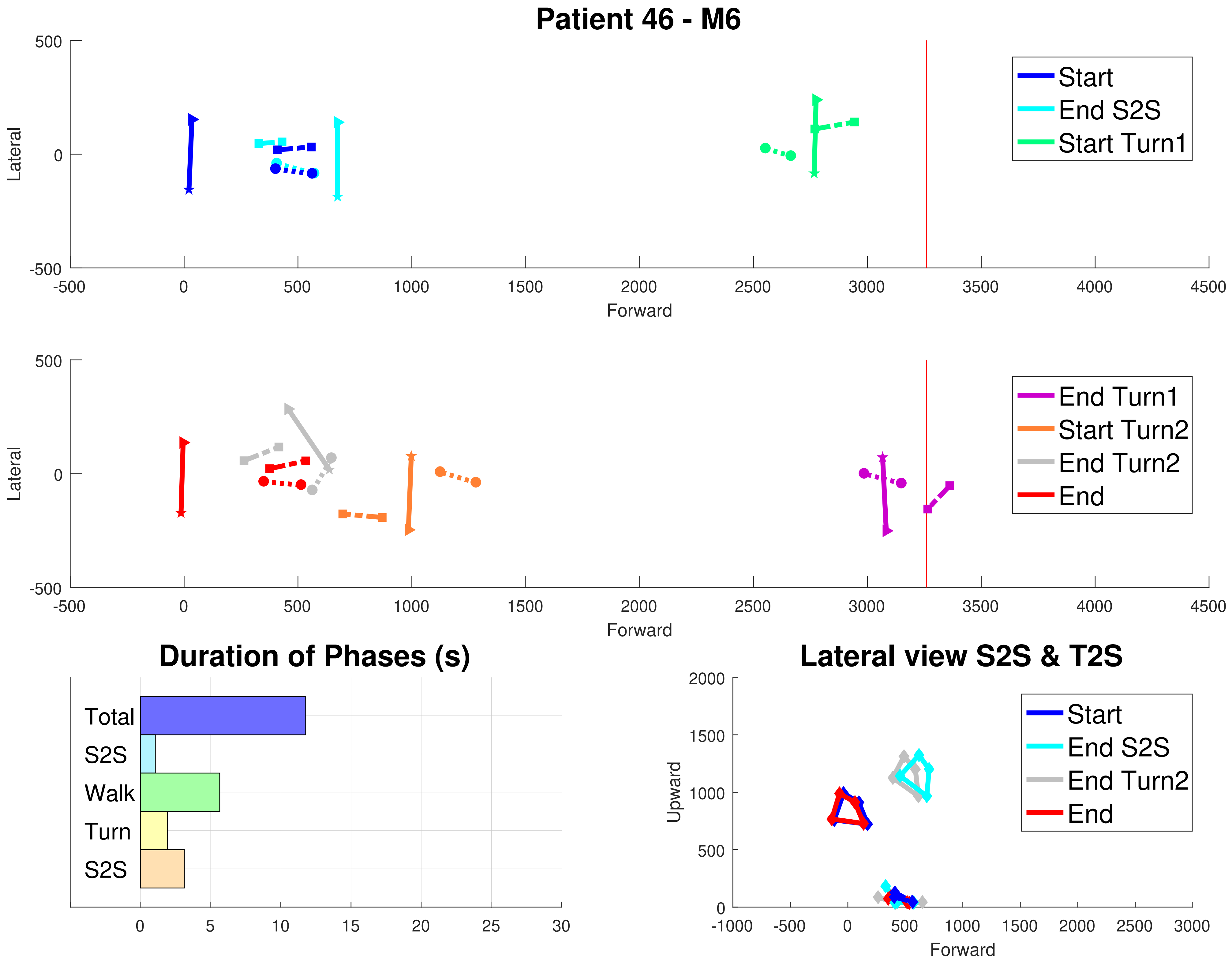
Patient 46 - M6
Lateral
Forward
Start
End S2S
Start Turn1
End Turn1
Start Turn2
End Turn2
End
Duration of Phases (s)
Total
S2S
Walk
Turn
S2S
Lateral view S2S & T2S
Upward
Forward
Start
End S2S
End Turn2
End

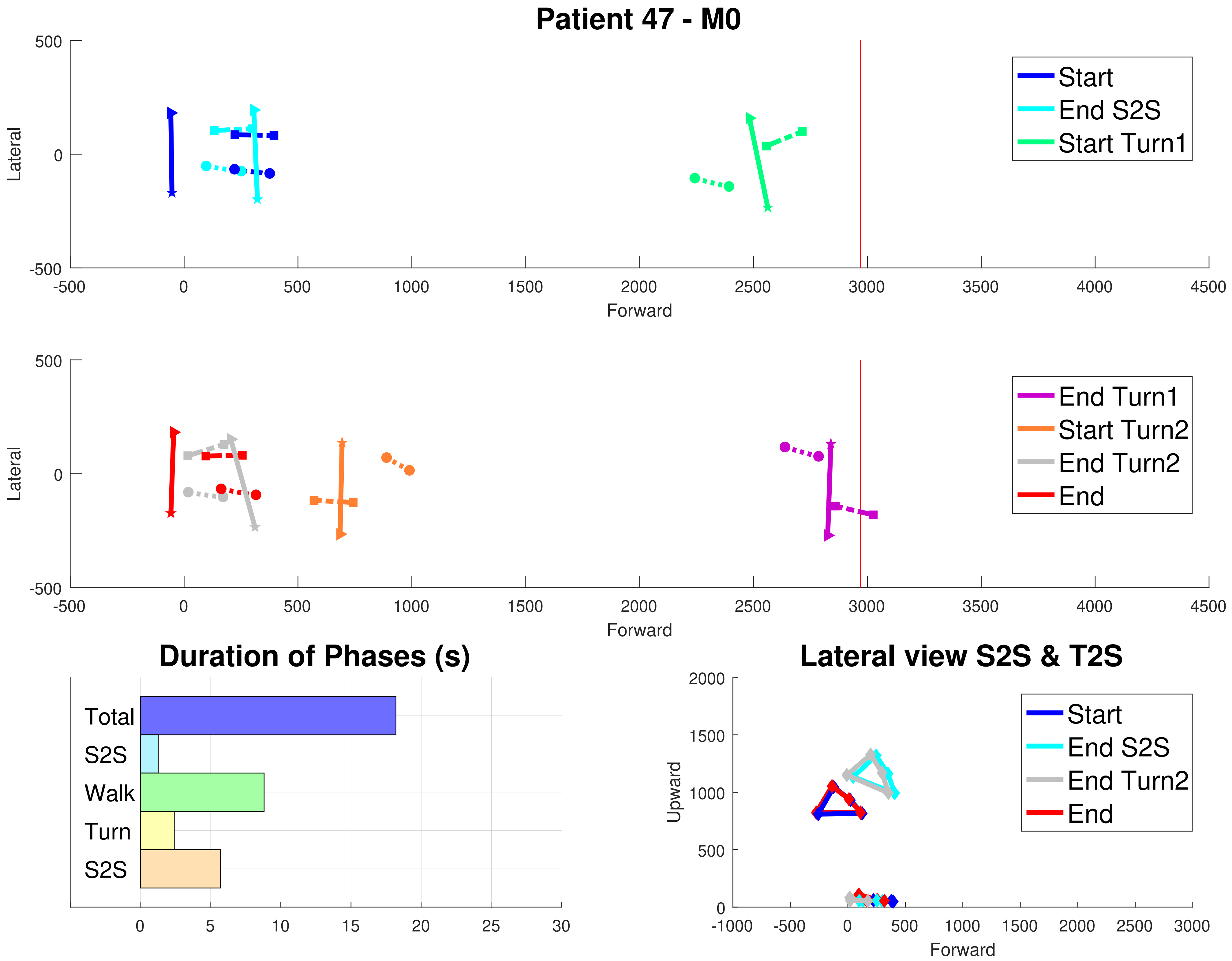

Patient 47 - M0
Lateral
Forward
Start
End S2S
Start Turn1
End Turn1
Start Turn2
End Turn2
End
Duration of Phases (s)
Total
S2S
Walk
Turn
S2S
Lateral view S2S & T2S
Upward
Forward
Start
End S2S
End Turn2
End

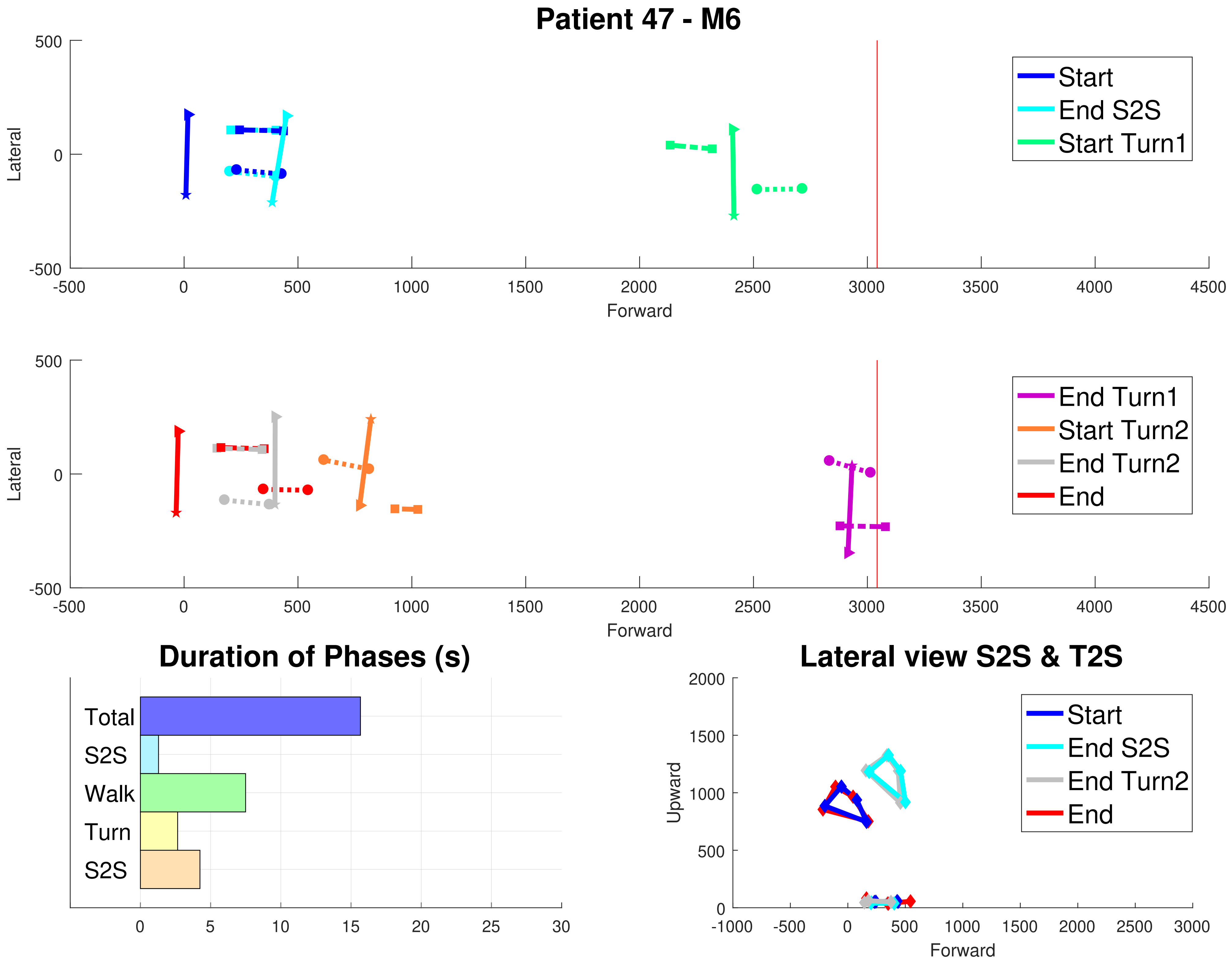

Patient 47 - M6
Lateral
Forward
Start
End S2S
Start Turn1
End Turn1
Start Turn2
End Turn2
End
Duration of Phases (s)
Total
S2S
Walk
Turn
S2S
Lateral view S2S & T2S
Upward
Forward
Start
End S2S
End Turn2
End

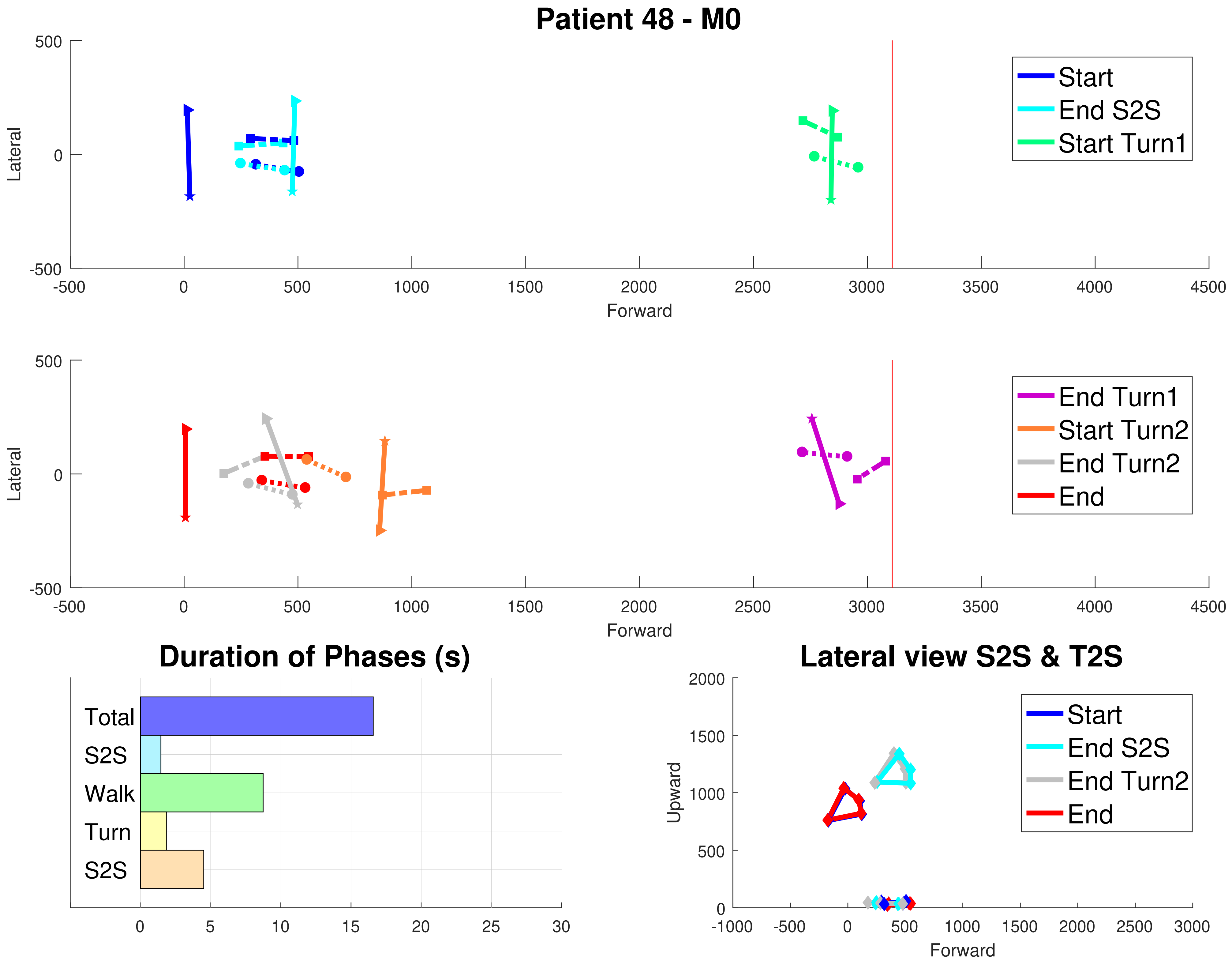

Patient 48 - M0
Lateral
Forward
Start
End S2S
Start Turn1
End Turn1
Start Turn2
End Turn2
End
Duration of Phases (s)
Total
S2S
Walk
Turn
S2S
Lateral view S2S & T2S
Upward
Forward
Start
End S2S
End Turn2
End

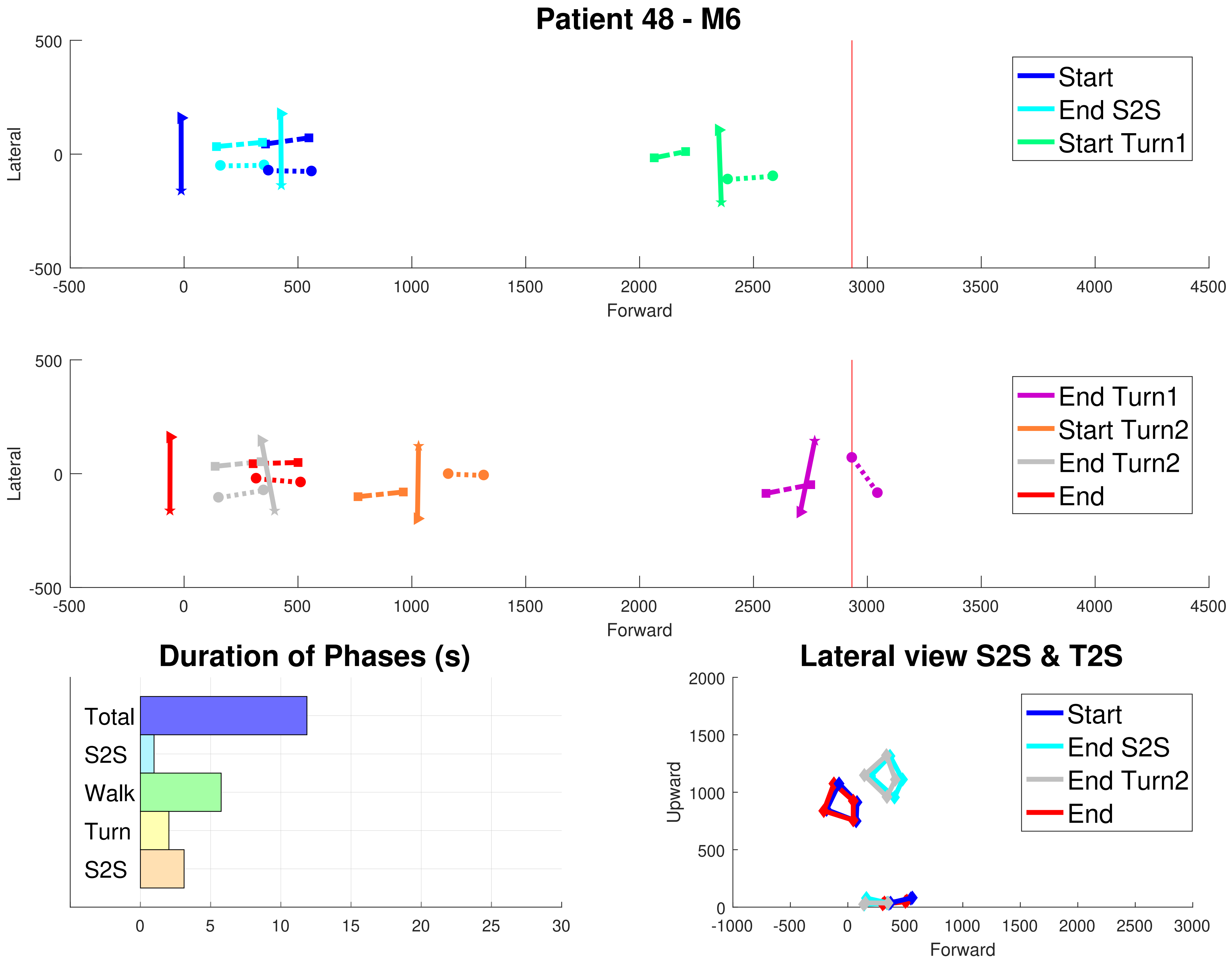
Patient 48 - M6
Lateral
Forward
Start
End S2S
Start Turn1
End Turn1
Start Turn2
End Turn2
End
Duration of Phases (s)
Total
S2S
Walk
Turn
S2S
Lateral view S2S & T2S
Upward
Forward
Start
End S2S
End Turn2
End

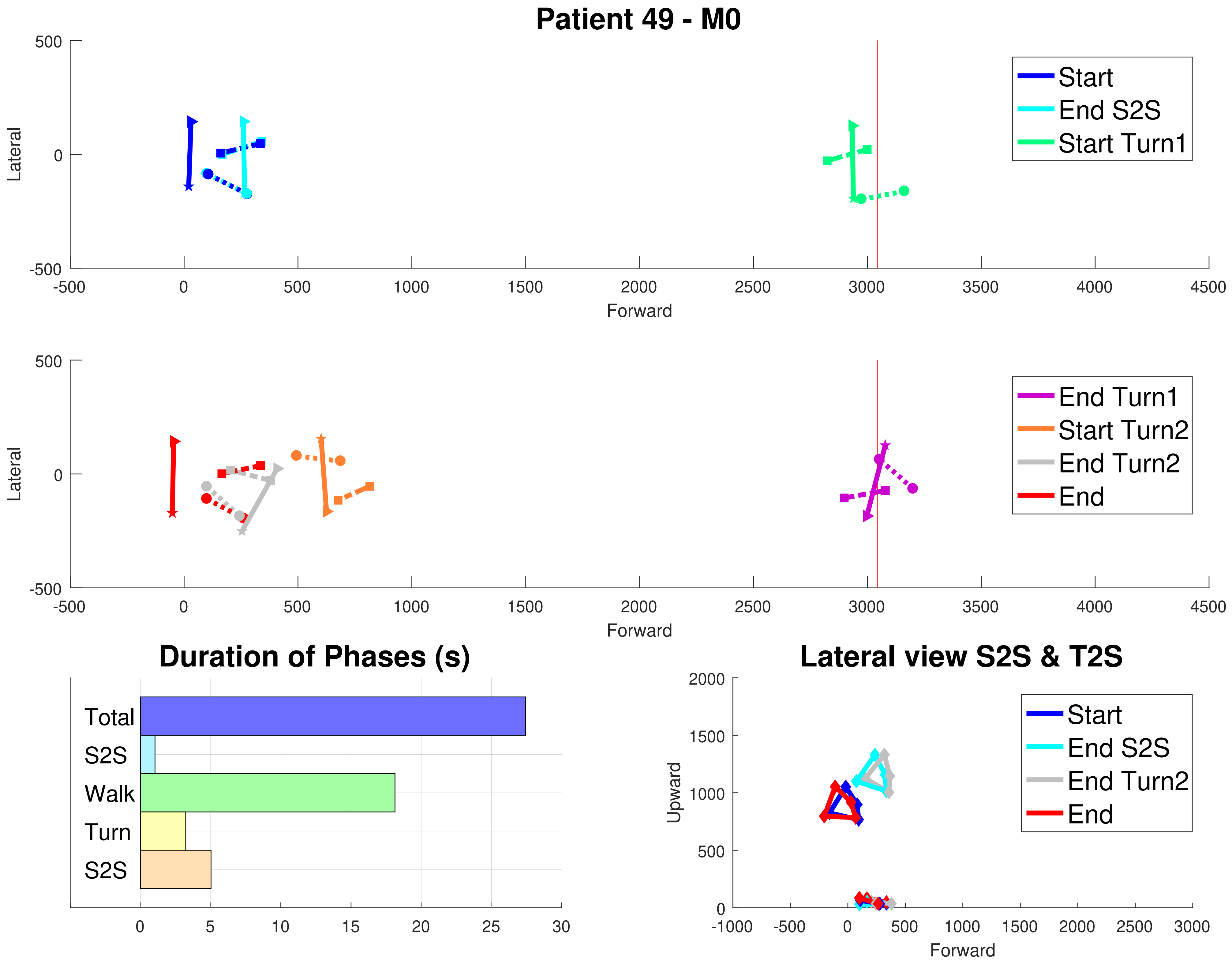
Patient 49 - M0
Lateral
Forward
Start
End S2S
Start Turn1
End Turn1
Start Turn2
End Turn2
End
Duration of Phases (s)
Total
S2S
Walk
Turn
S2S
Lateral view S2S & T2S
Upward
Forward
Start
End S2S
End Turn2
End

Patient 49 - M6

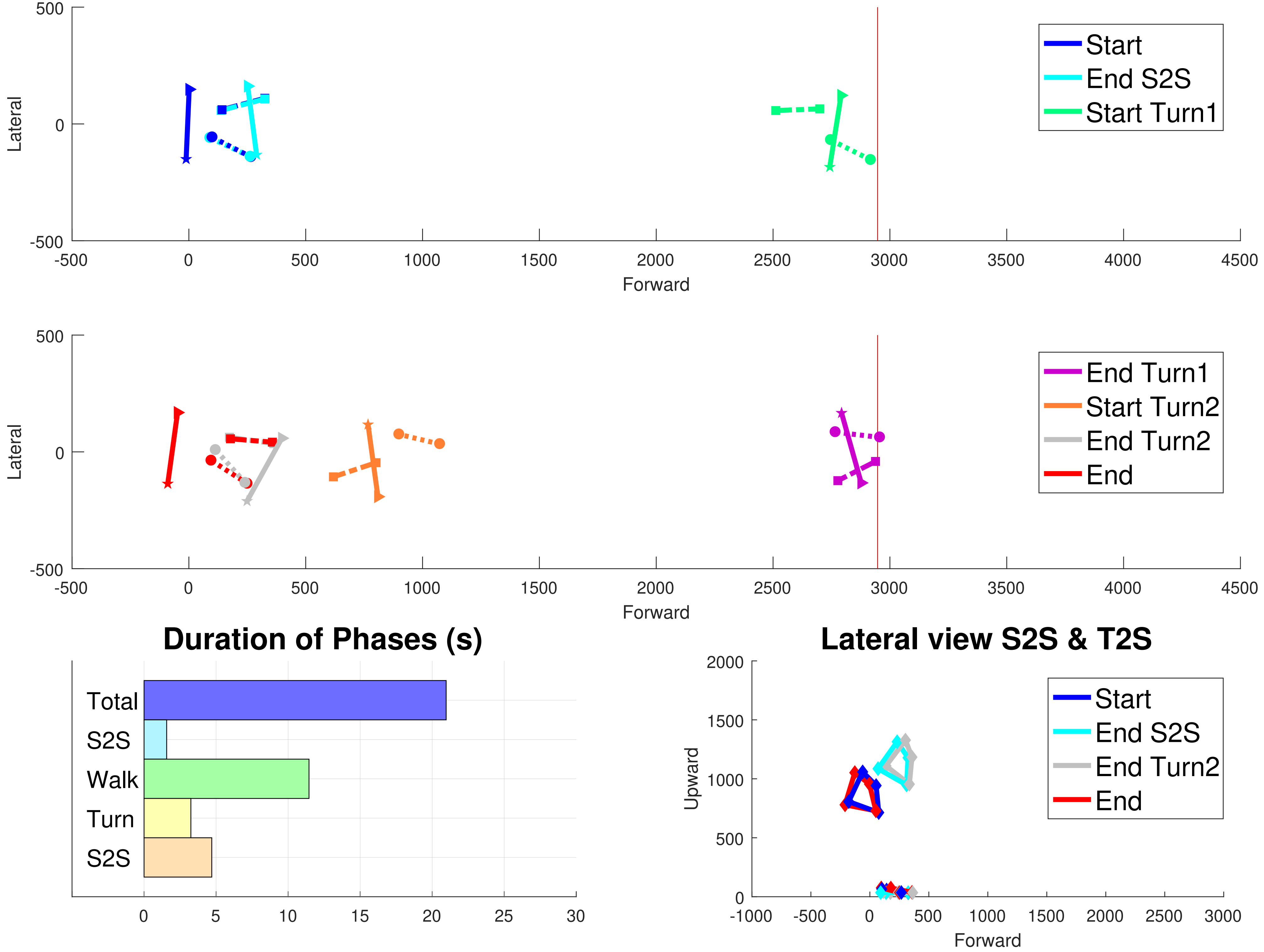

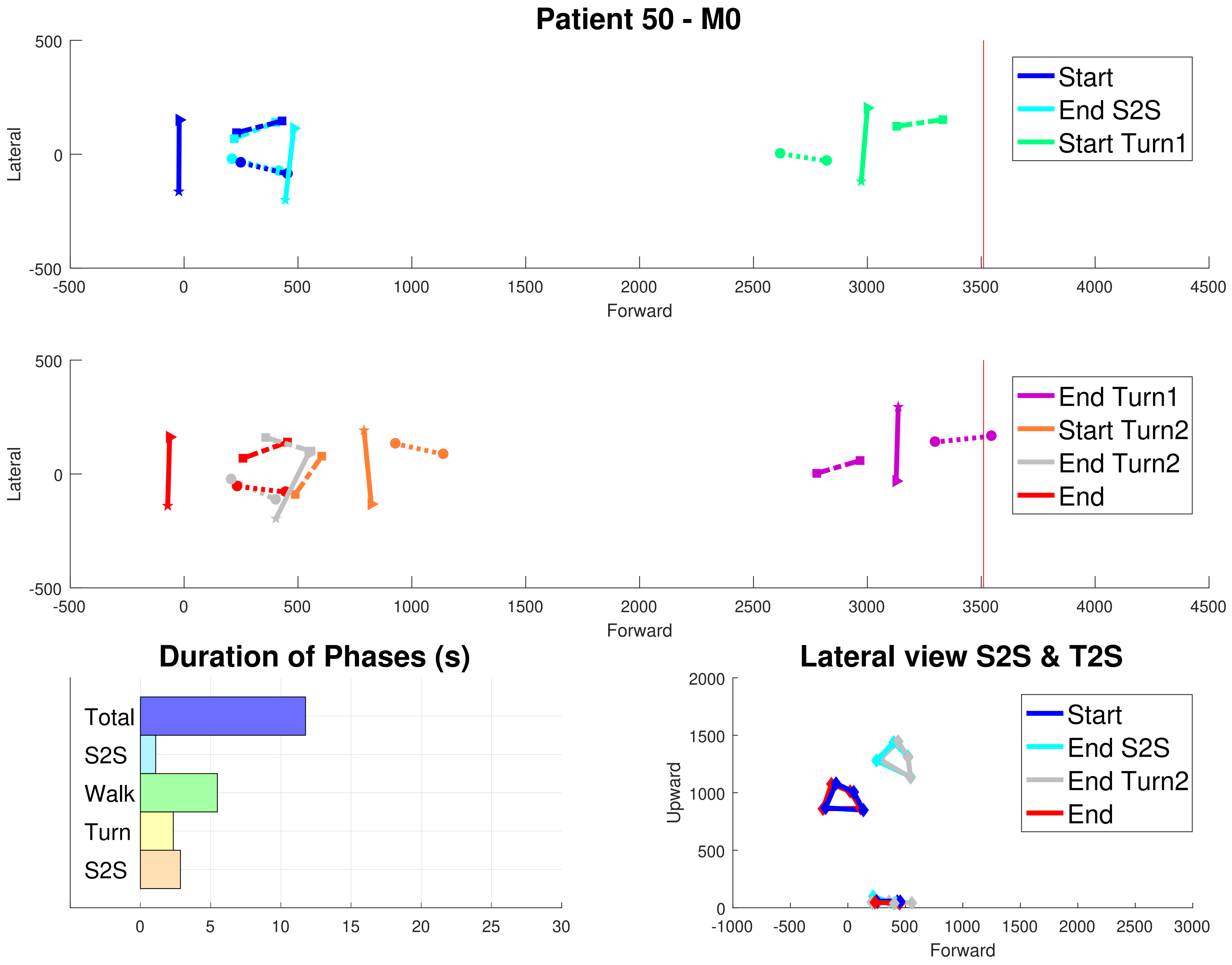
Patient 50 - M0
Lateral
Forward
500
0
-500
-500
0
500
1000
1500
2000
2500
3000
3500
4000
4500
Start
End S2S
Start Turn1
End Turn1
Start Turn2
End Turn2
End
Duration of Phases (s)
Total
S2S
Walk
Turn
S2S
0
5
10
15
20
25
30
Lateral view S2S & T2S
Upward
2000
1500
1000
500
-1000
-500
3000
Start
End S2S
End Turn2
End

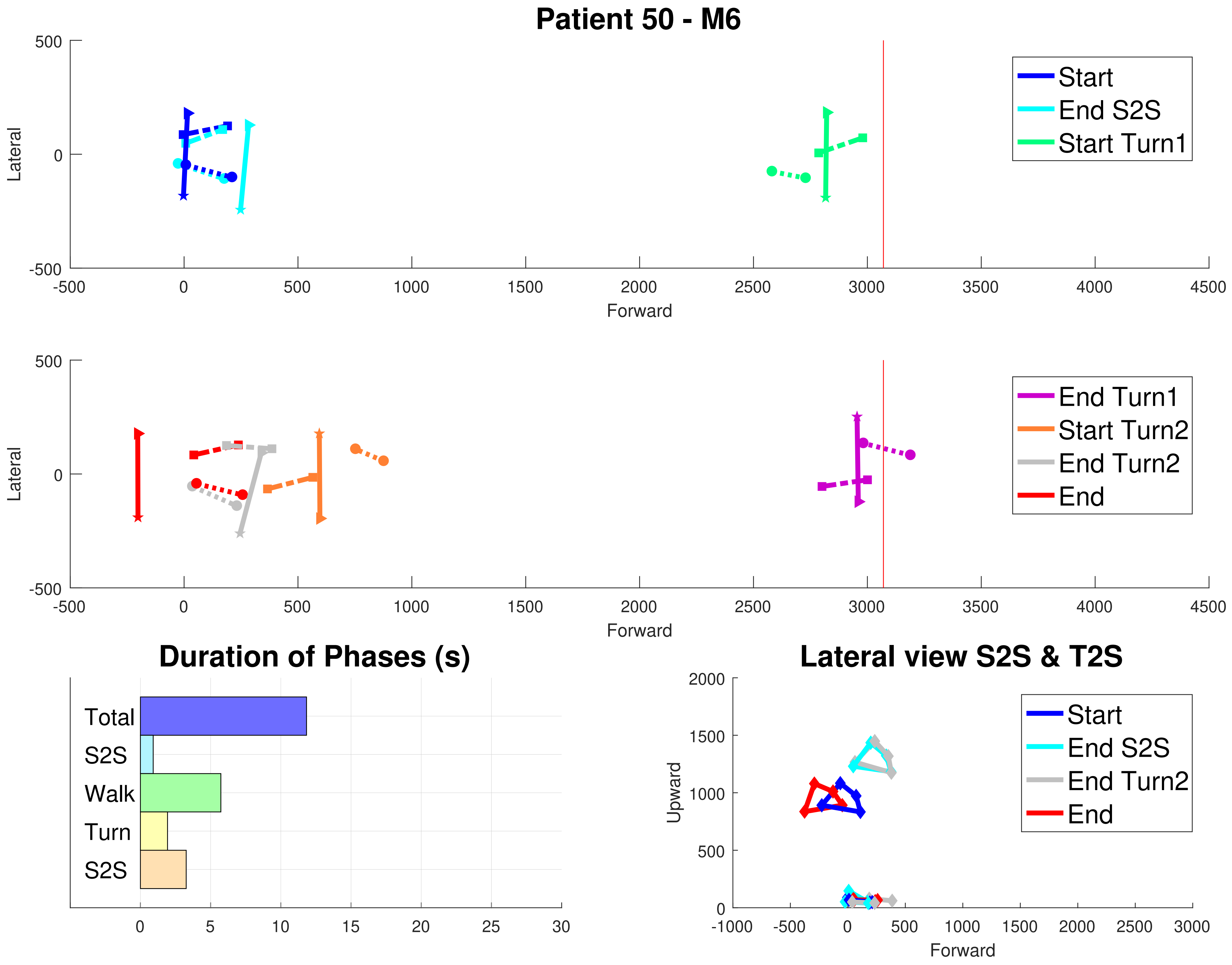
Patient 50 - M6
Lateral
Forward
Start
End S2S
Start Turn1
End Turn1
Start Turn2
End Turn2
End
Duration of Phases (s)
Total
S2S
Walk
Turn
S2S
Lateral view S2S & T2S
Upward
Forward
Start
End S2S
End Turn2
End

Patient 51 - M0

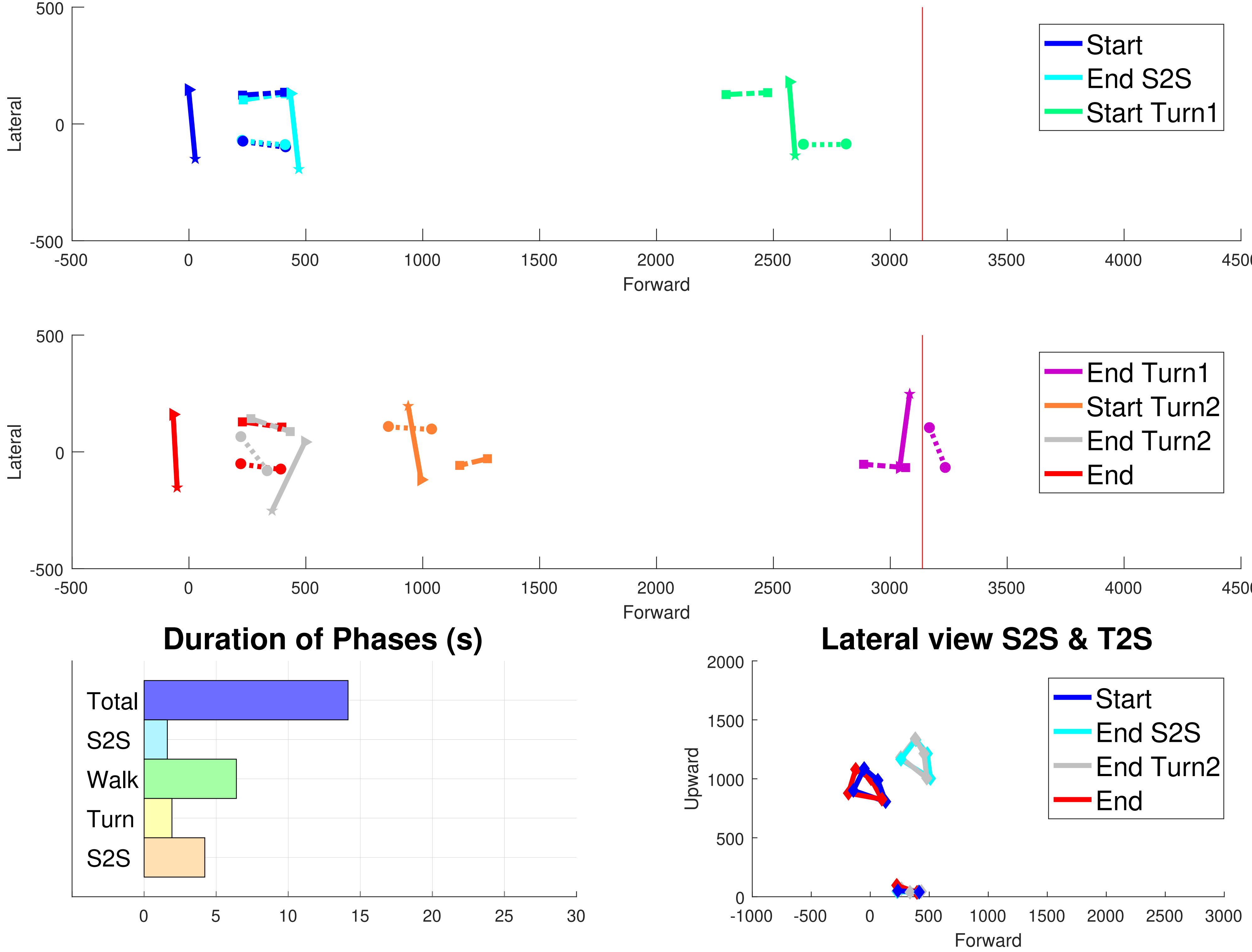

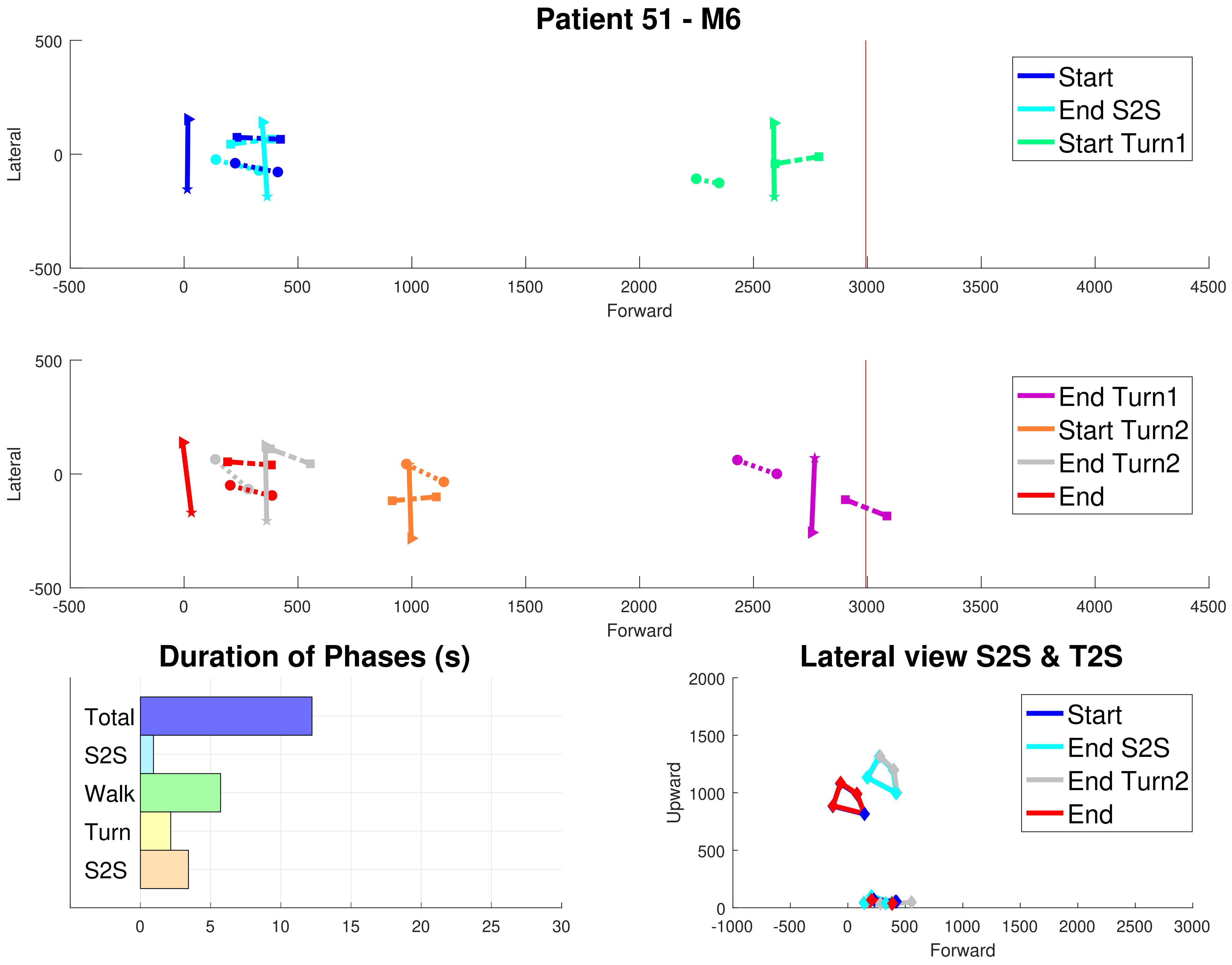
Patient 51 - M6
Lateral
Forward
Start
End S2S
Start Turn1
End Turn1
Start Turn2
End Turn2
End
Duration of Phases (s)
Total
S2S
Walk
Turn
S2S
Lateral view S2S & T2S
Upward
Forward
Start
End S2S
End Turn2
End

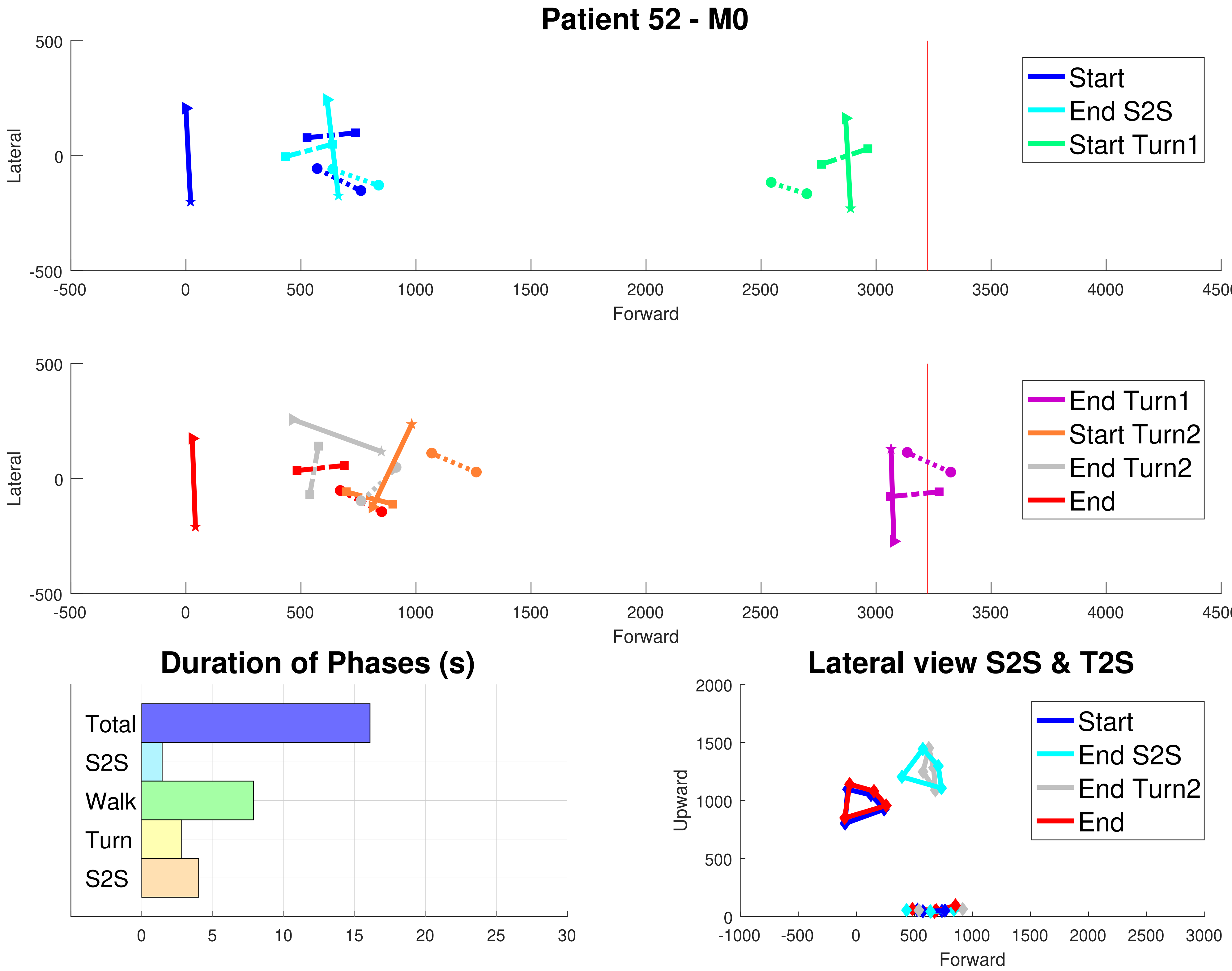

Patient 52 - M0
Lateral
Forward
Start
End S2S
Start Turn1
End Turn1
Start Turn2
End Turn2
End
Duration of Phases (s)
Total
S2S
Walk
Turn
S2S
Lateral view S2S & T2S
Upward
Forward
Start
End S2S
End Turn2
End

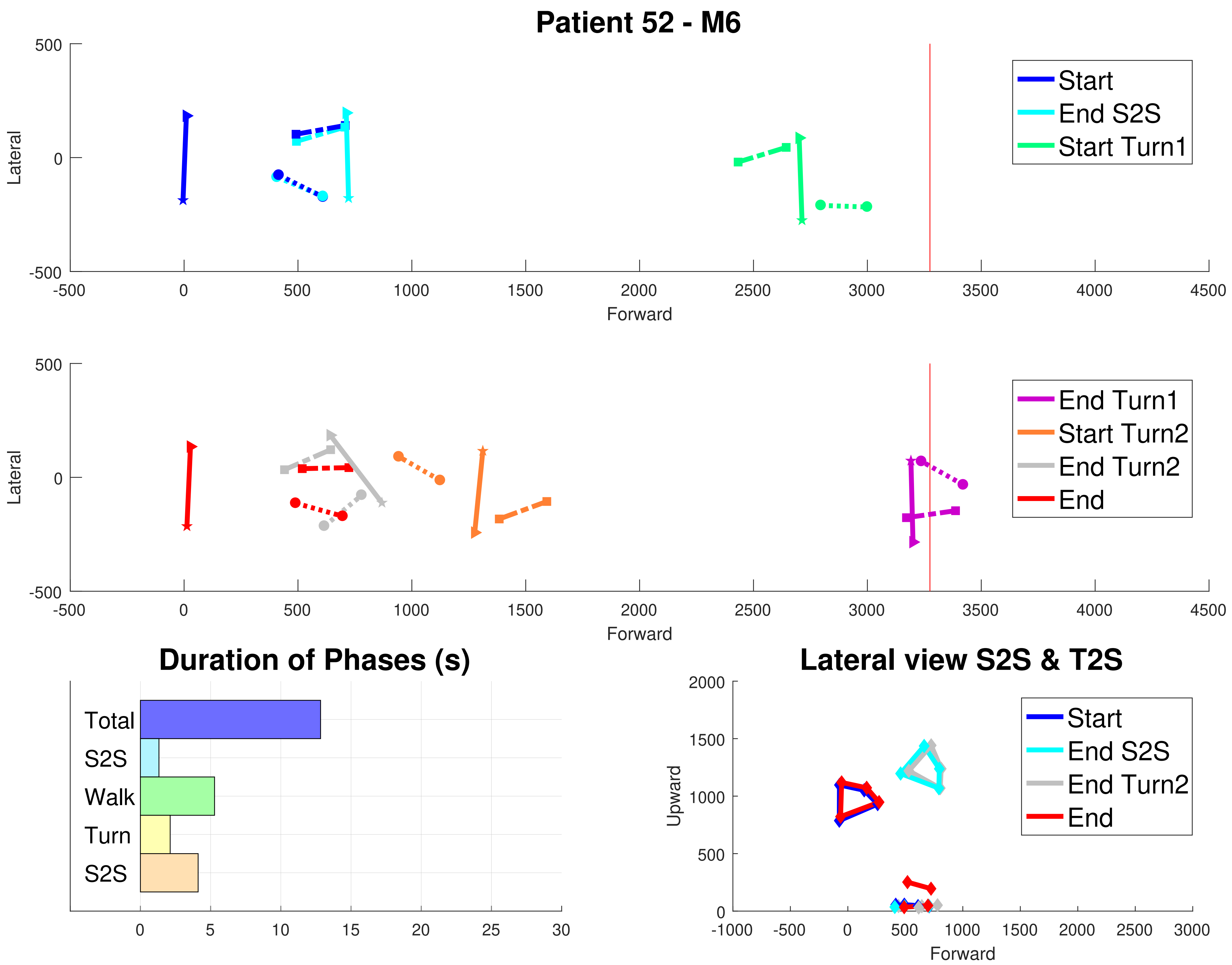

Patient 52 - M6
Lateral
Forward
Start
End S2S
Start Turn1
End Turn1
Start Turn2
End Turn2
End
Duration of Phases (s)
Total
S2S
Walk
Turn
S2S
Lateral view S2S & T2S
Upward

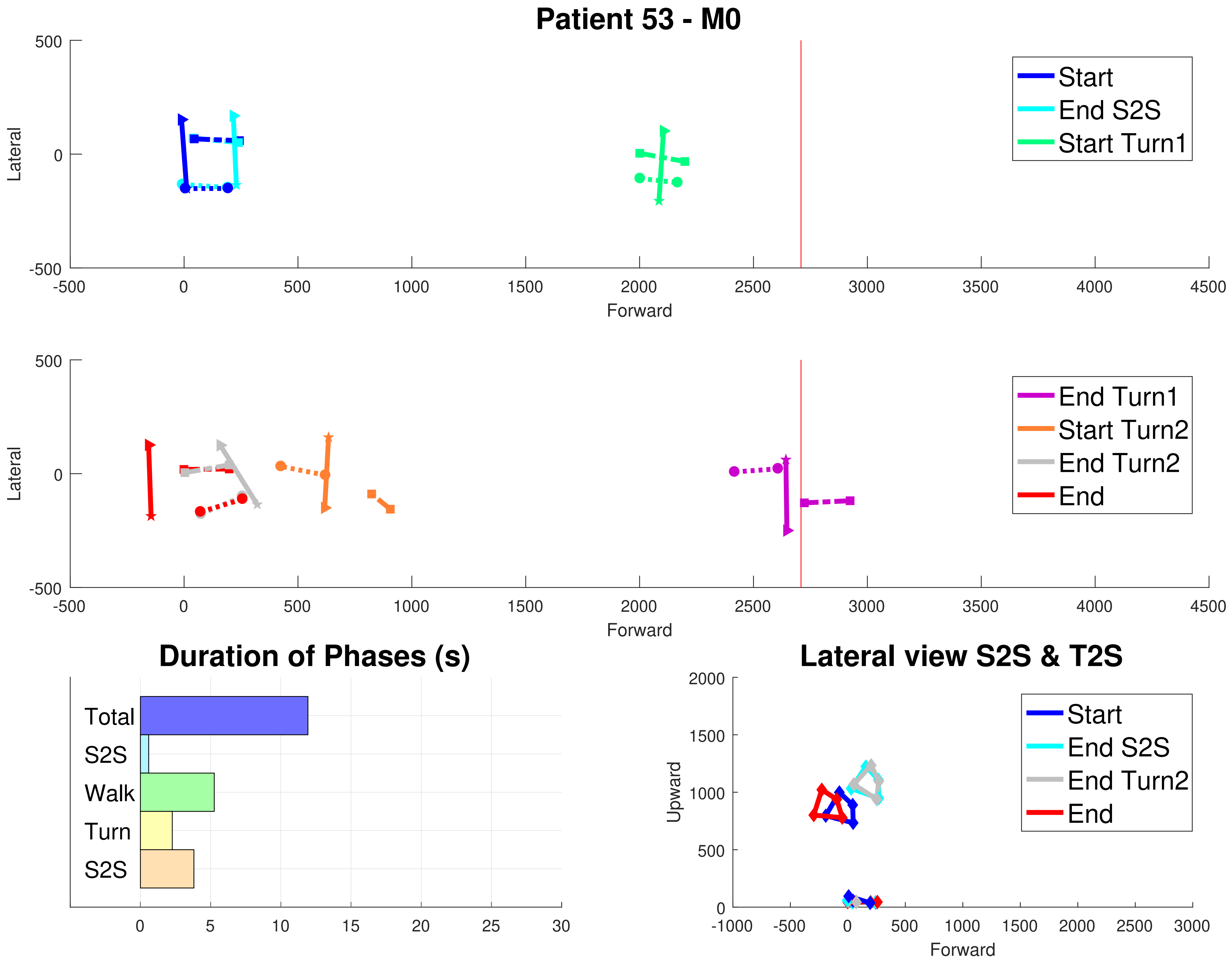
Patient 53 - M0
Lateral
Forward
Start
End S2S
Start Turn1
End Turn1
Start Turn2
End Turn2
End
Duration of Phases (s)
Total
S2S
Walk
Turn
S2S
Lateral view S2S & T2S
Upward
Forward
Start
End S2S
End Turn2
End

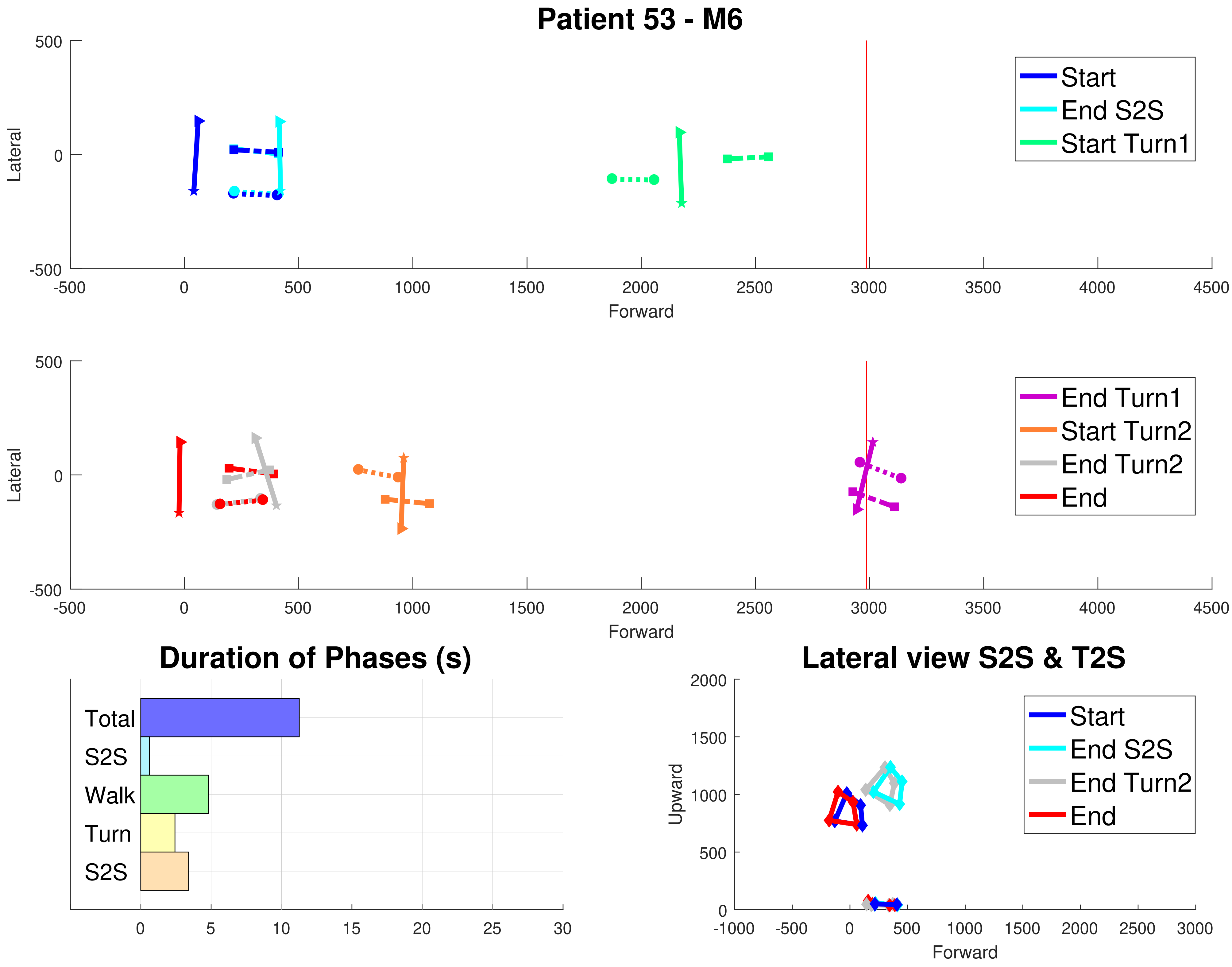
Patient 53 - M6
Lateral
Forward
Start
End S2S
Start Turn1
End Turn1
Start Turn2
End Turn2
End
Duration of Phases (s)
Total
S2S
Walk
Turn
S2S
Lateral view S2S & T2S
Upward
Forward
Start
End S2S
End Turn2
End

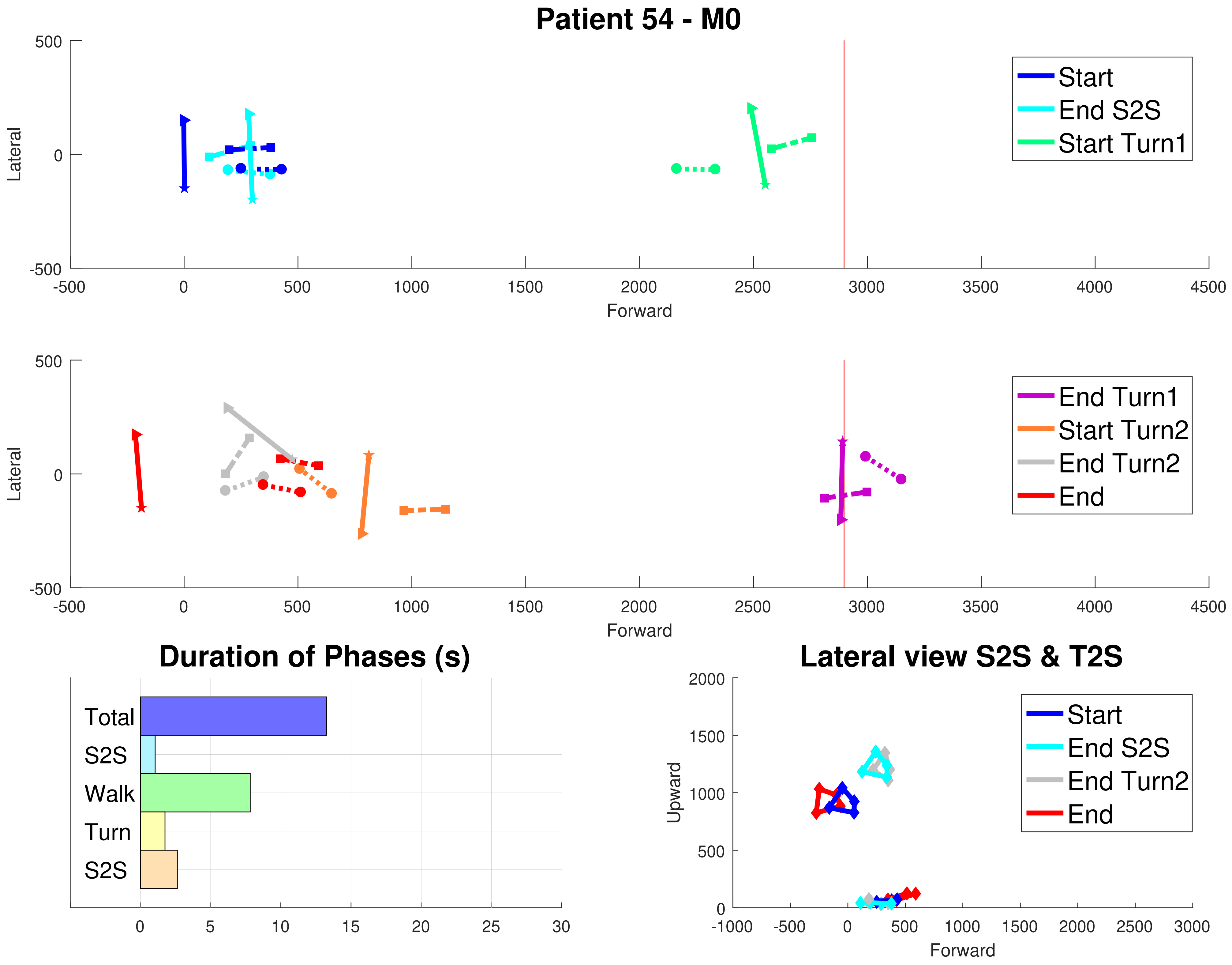

Patient 54 - M0
Lateral
Forward
Start
End S2S
Start Turn1
End Turn1
Start Turn2
End Turn2
End
Duration of Phases (s)
Total
S2S
Walk
Turn
S2S
Lateral view S2S & T2S
Upward
Forward
Start
End S2S
End Turn2
End

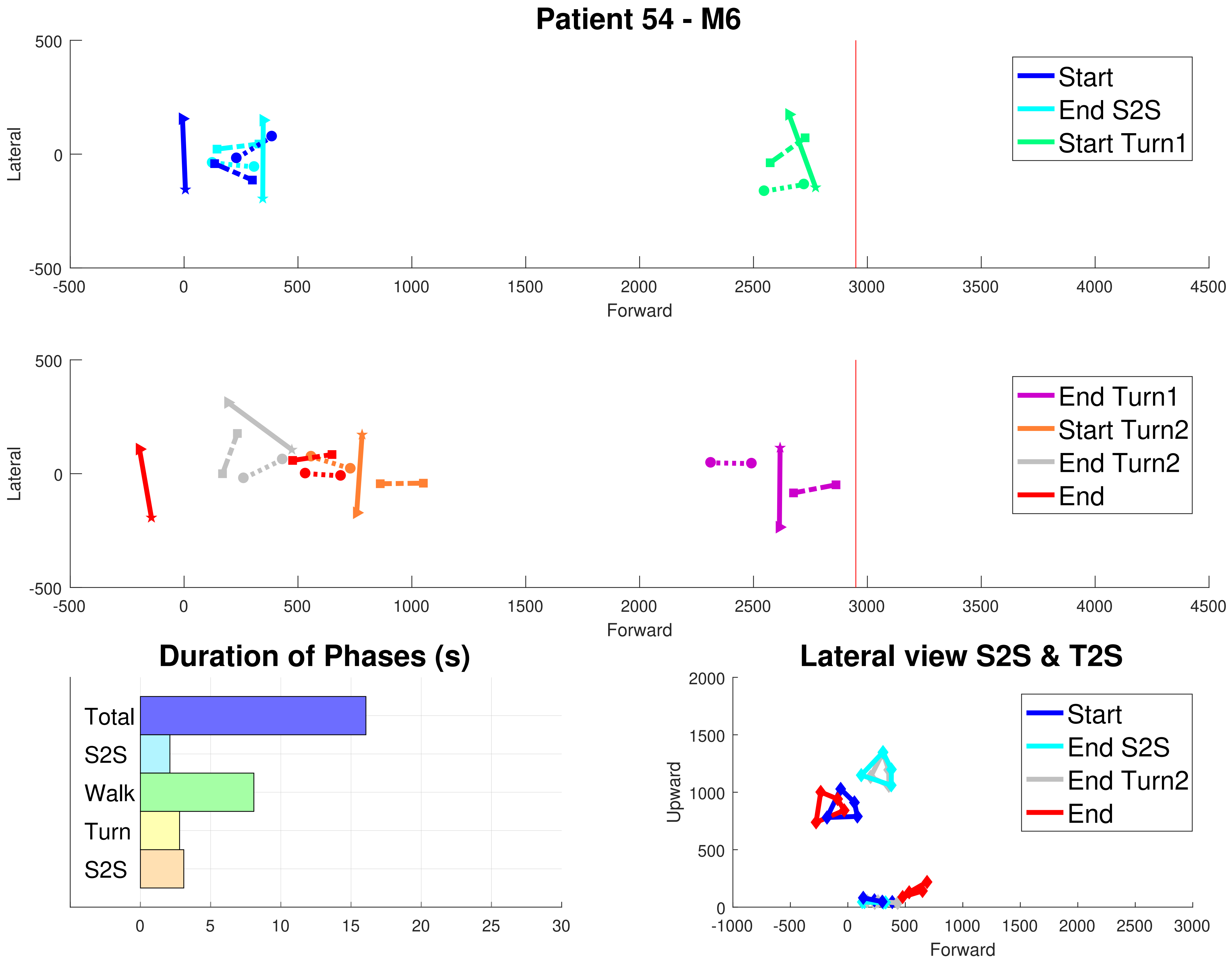
Patient 54 - M6
Lateral
Forward
Start
End S2S
Start Turn1
End Turn1
Start Turn2
End Turn2
End
Duration of Phases (s)
Total
S2S
Walk
Turn
S2S
Lateral view S2S & T2S
Upward
Forward
Start
End S2S
End Turn2
End

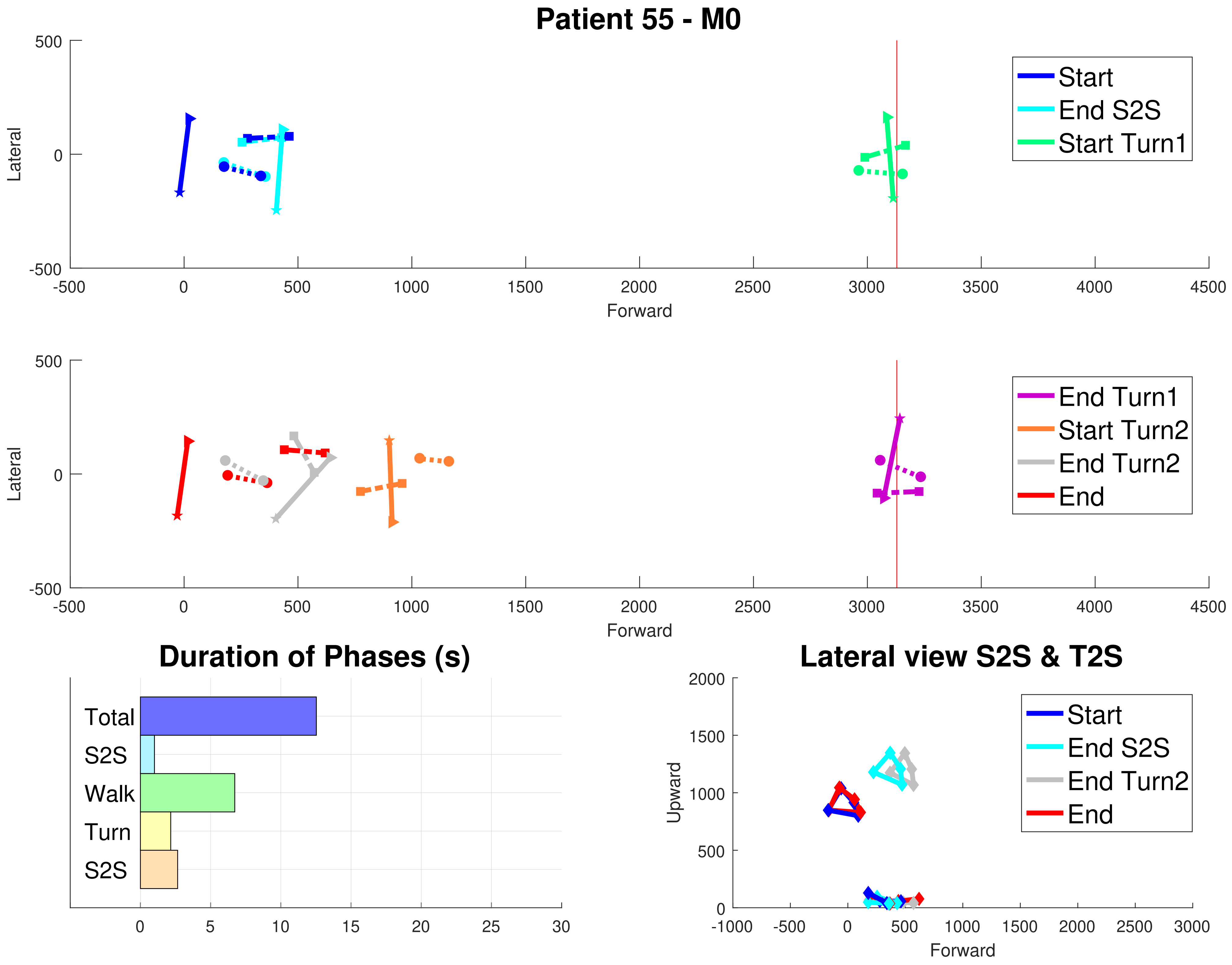

Patient 55 - M0
Lateral
Forward
Start
End S2S
Start Turn1
End Turn1
Start Turn2
End Turn2
End
Duration of Phases (s)
Total
S2S
Walk
Turn
S2S
Lateral view S2S & T2S
Upward
Forward
Start
End S2S
End Turn2
End

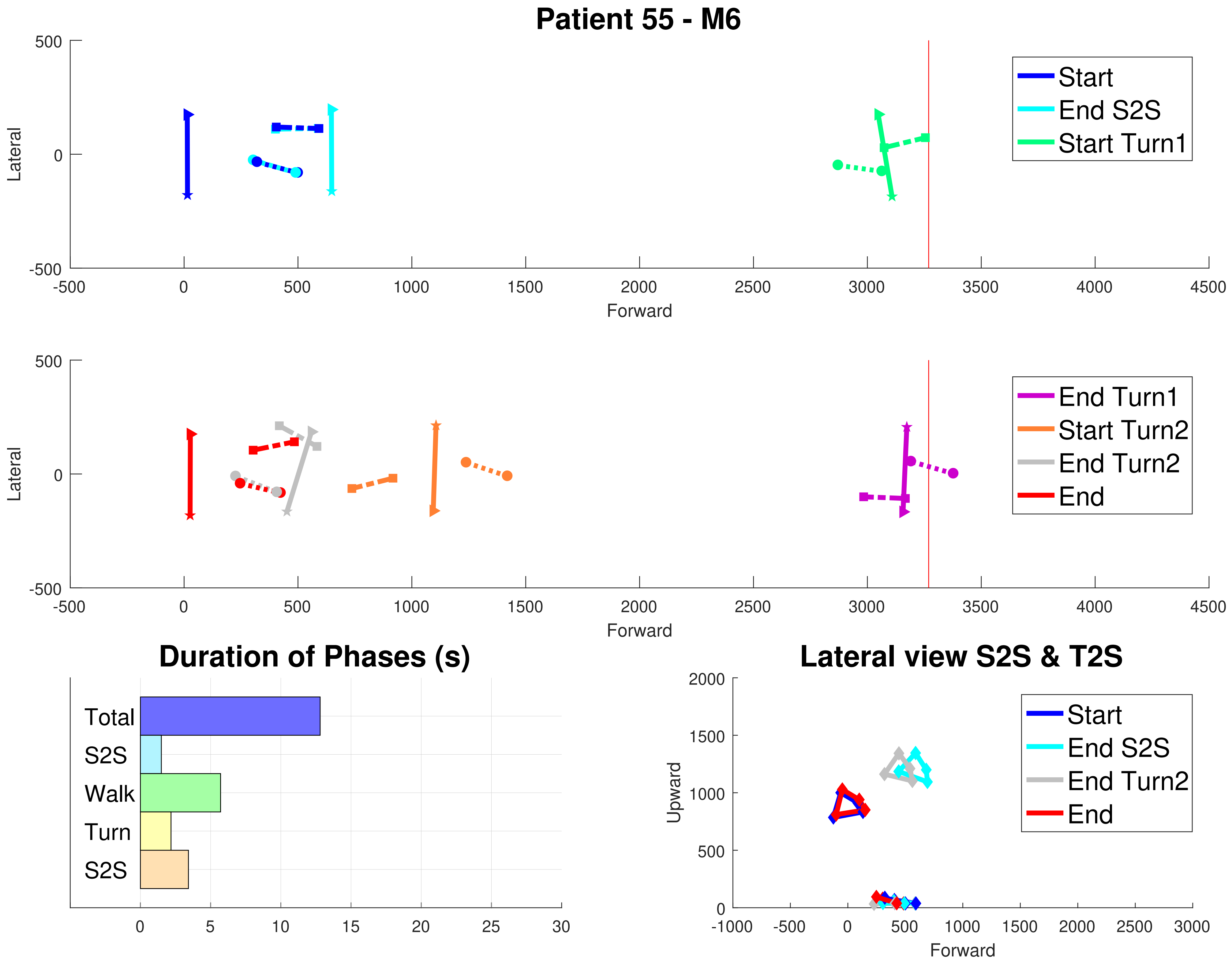

Patient 55 - M6
Lateral
Forward
Start
End S2S
Start Turn1
End Turn1
Start Turn2
End Turn2
End
Duration of Phases (s)
Total
S2S
Walk
Turn
S2S
Lateral view S2S & T2S
Upward
Forward
Start
End S2S
End Turn2
End

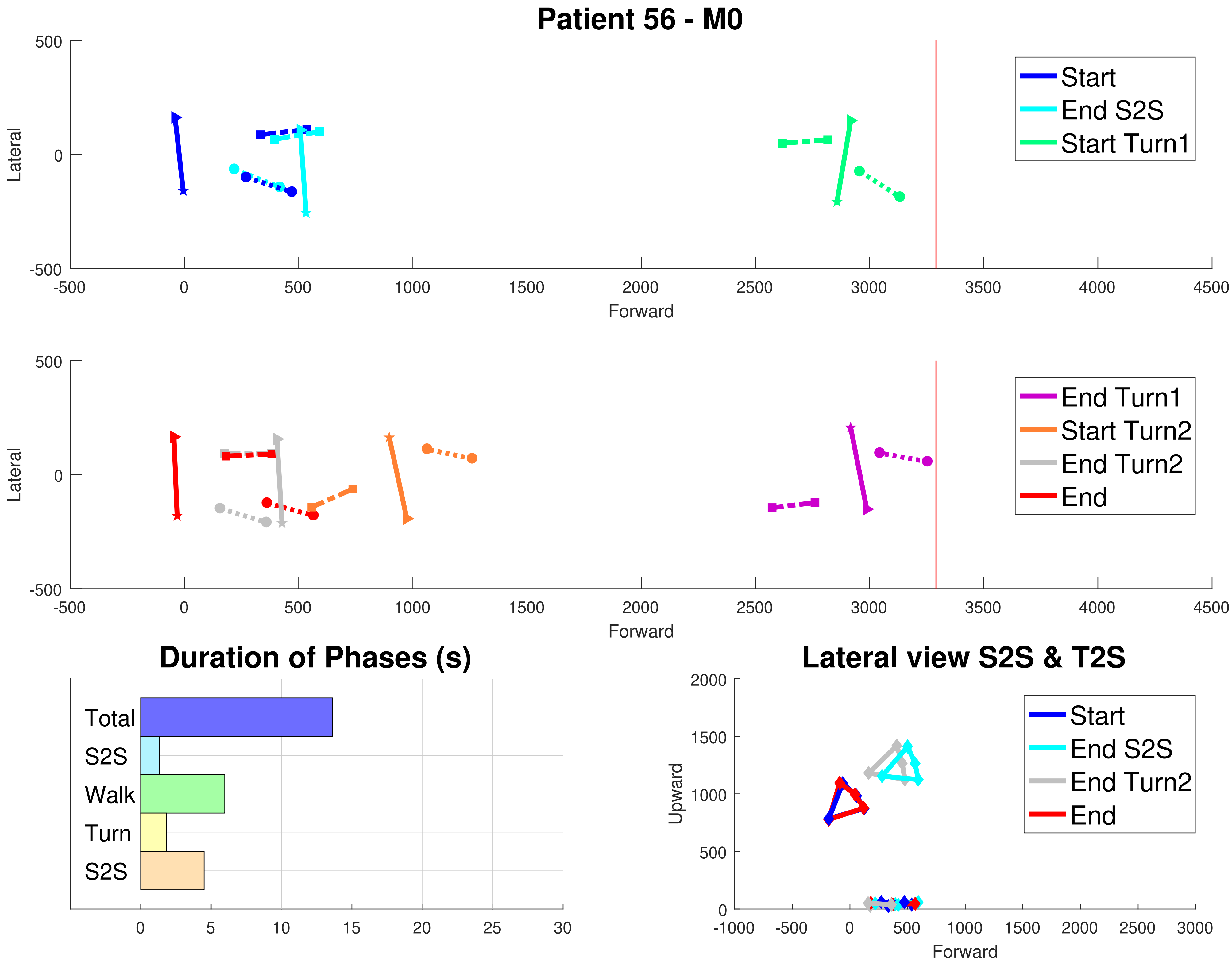

Patient 56 - M0
Lateral
Forward
Start
End S2S
Start Turn1
End Turn1
Start Turn2
End Turn2
End
Duration of Phases (s)
Total
S2S
Walk
Turn
S2S
Lateral view S2S & T2S
Upward

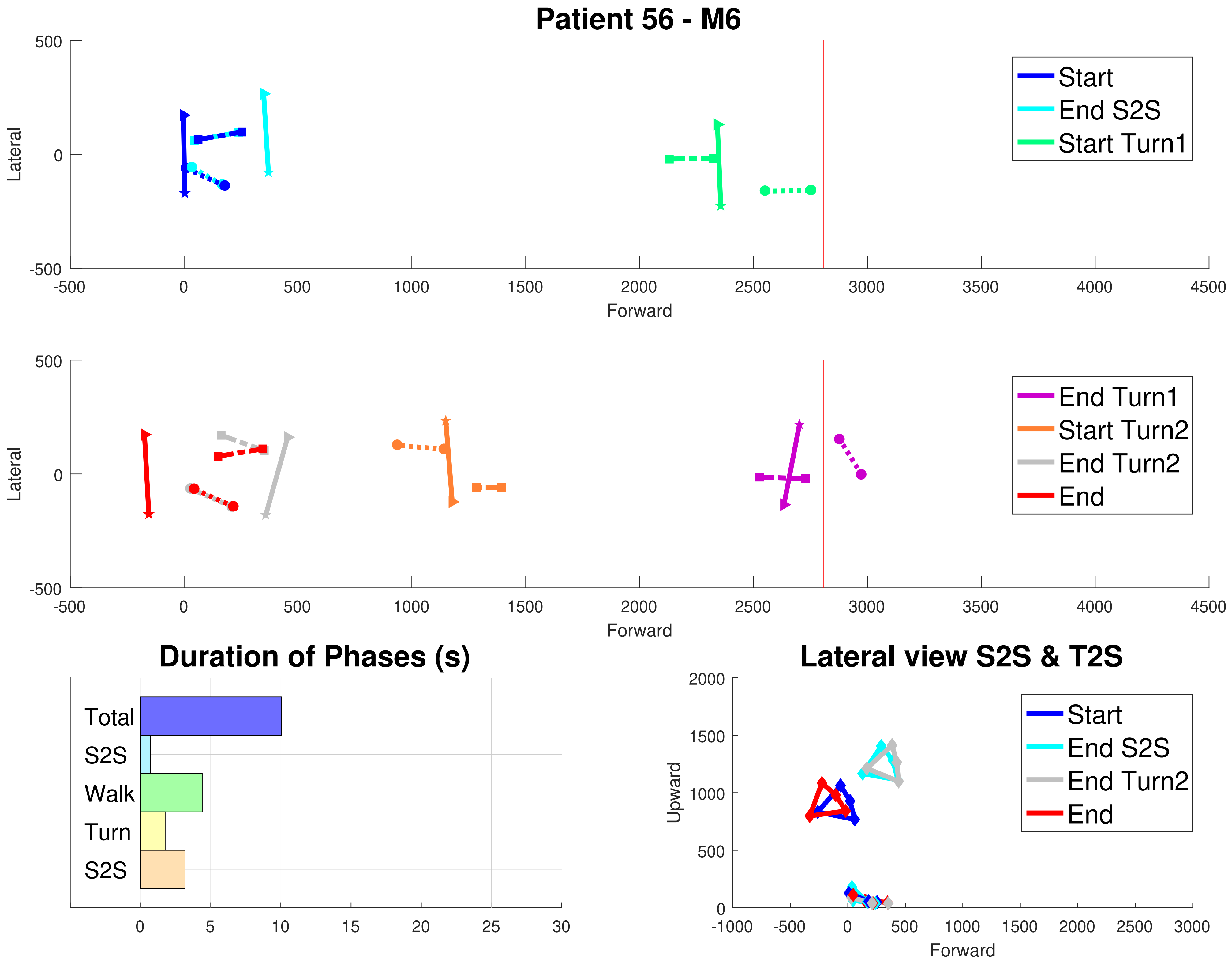

Patient 56 - M6
Lateral
Forward
Start
End S2S
Start Turn1
End Turn1
Start Turn2
End Turn2
End
Duration of Phases (s)
Total
S2S
Walk
Turn
S2S
Lateral view S2S & T2S
Upward
Forward
Start
End S2S
End Turn2
End

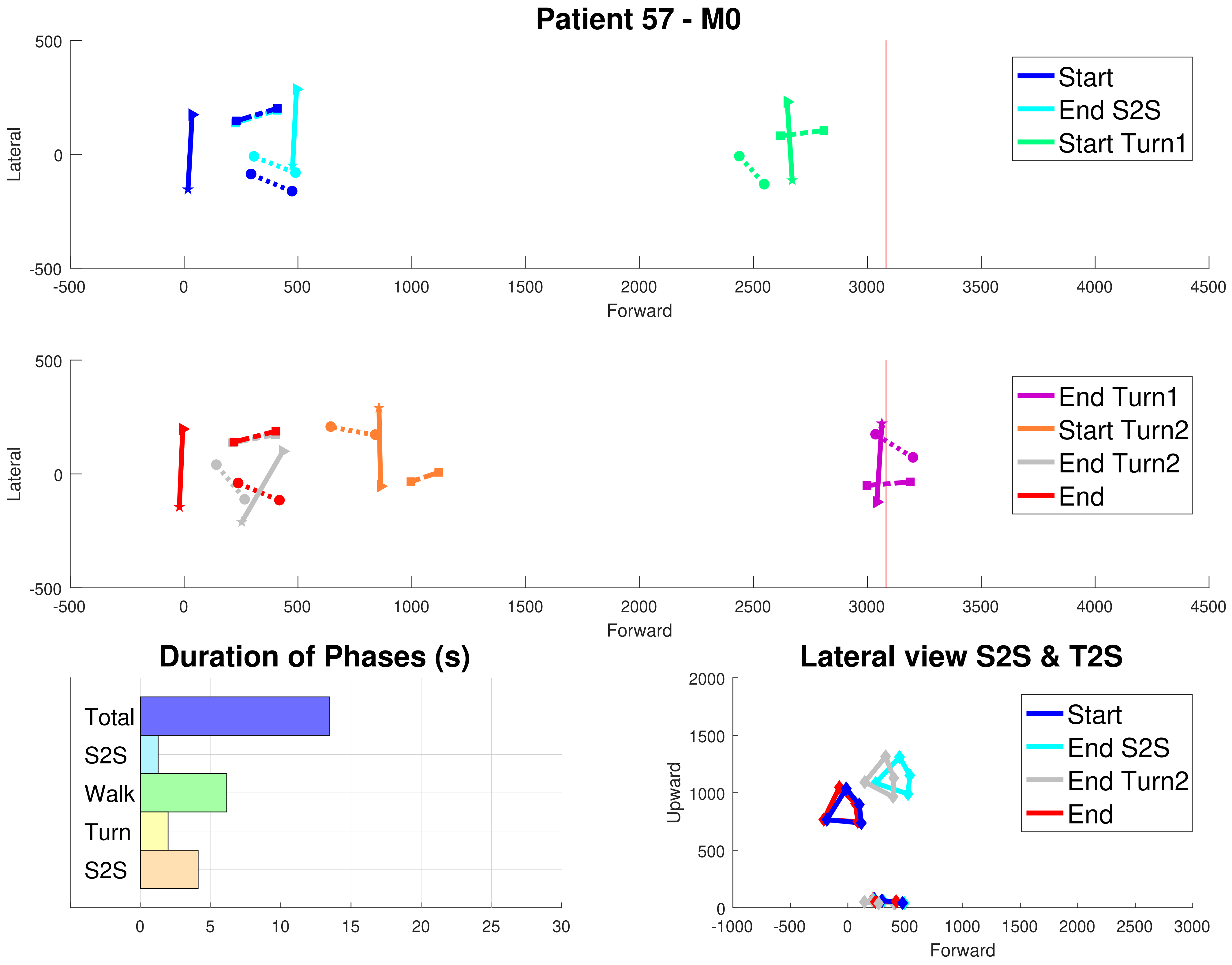
Patient 57 - M0
Lateral
Forward
Start
End S2S
Start Turn1
End Turn1
Start Turn2
End Turn2
End
Duration of Phases (s)
Total
S2S
Walk
Turn
S2S
Lateral view S2S & T2S
Upward
Forward
Start
End S2S
End Turn2
End

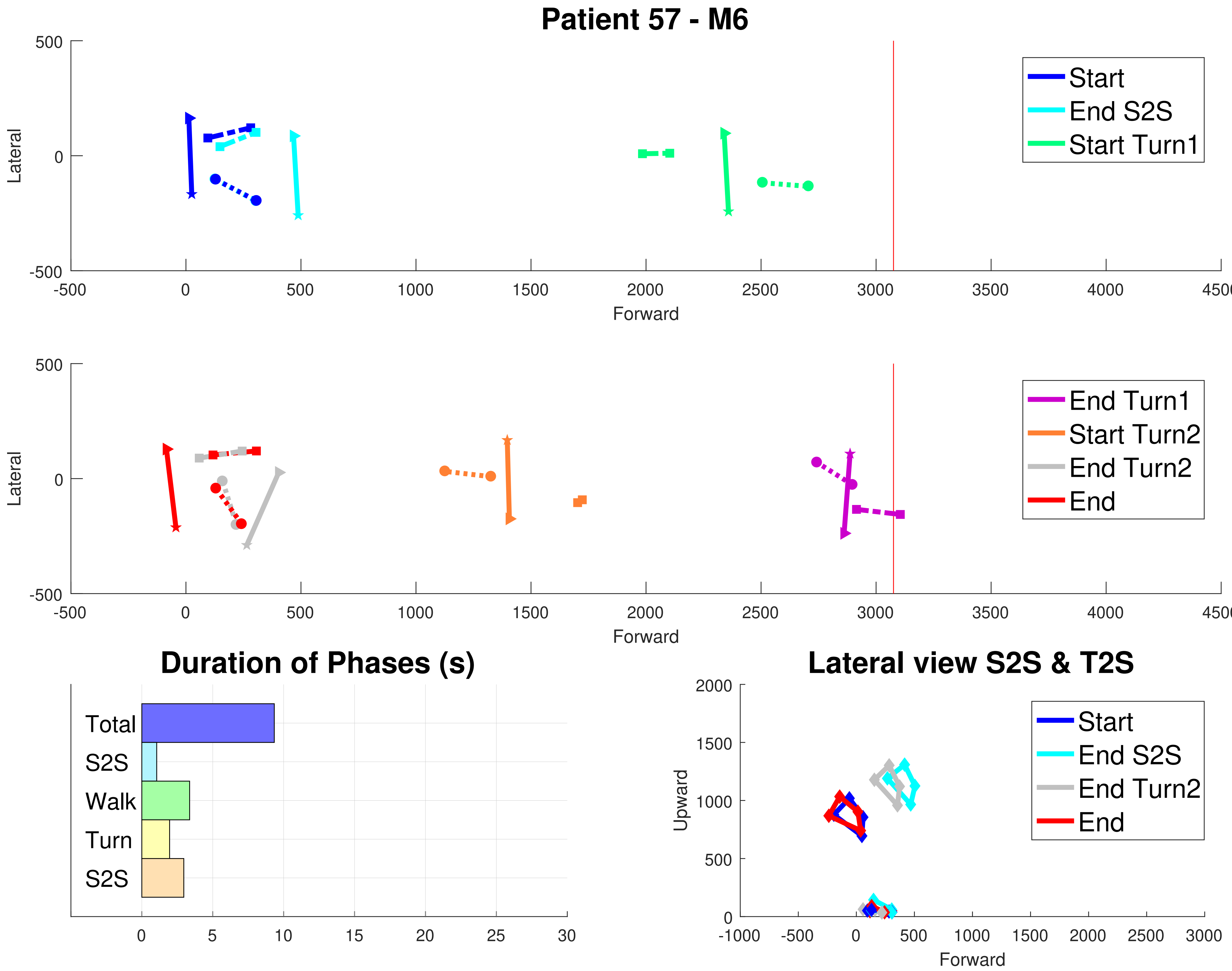
Patient 57 - M6
Lateral
Forward
Start
End S2S
Start Turn1
End Turn1
Start Turn2
End Turn2
End
Duration of Phases (s)
Total
S2S
Walk
Turn
S2S
Lateral view S2S & T2S
Upward
Forward
Start
End S2S
End Turn2
End

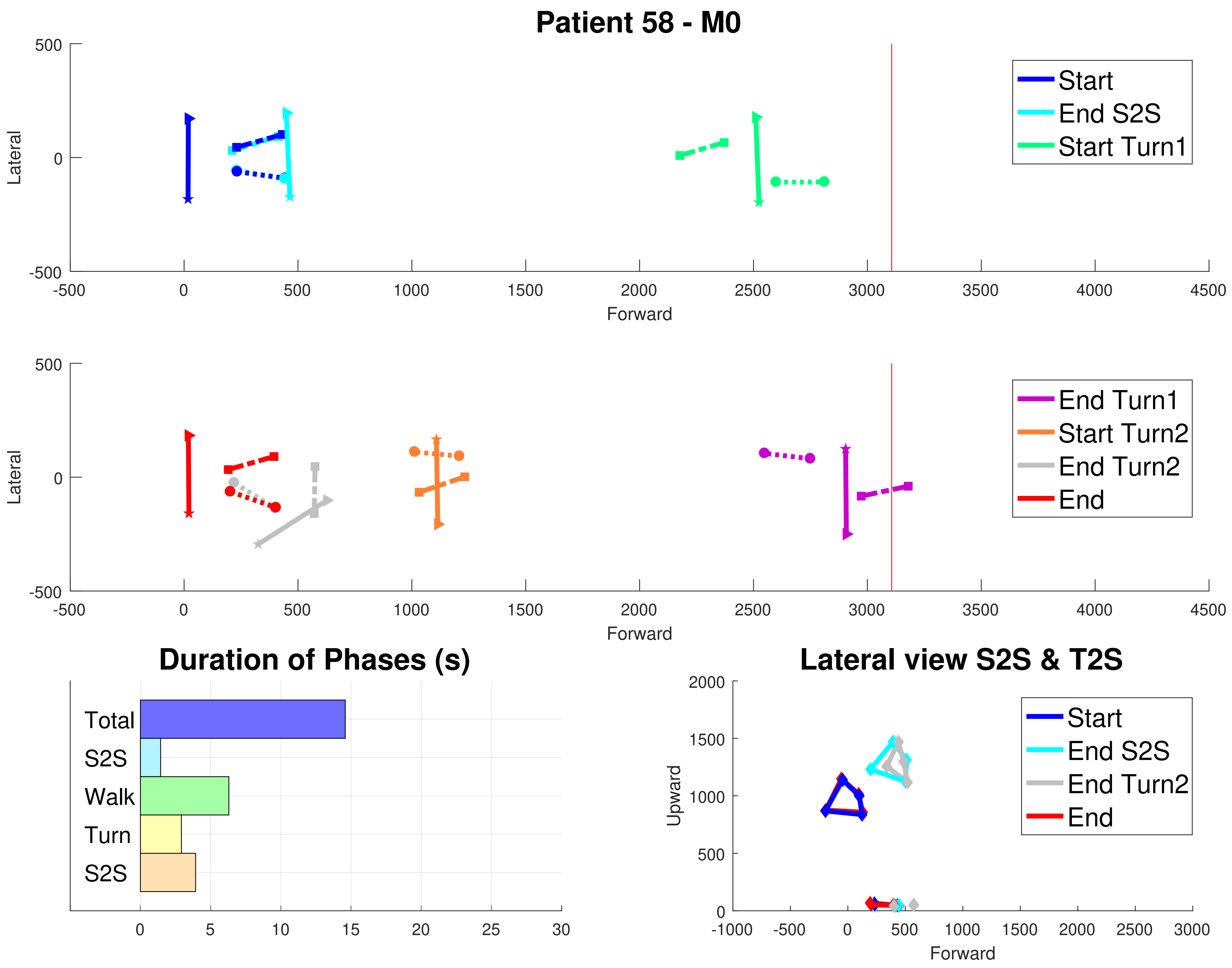

Patient 58 - M0
Lateral
Forward
Start
End S2S
Start Turn1
End Turn1
Start Turn2
End Turn2
End
Duration of Phases (s)
Total
S2S
Walk
Turn
S2S
Lateral view S2S & T2S
Upward
Forward
Start
End S2S
End Turn2
End

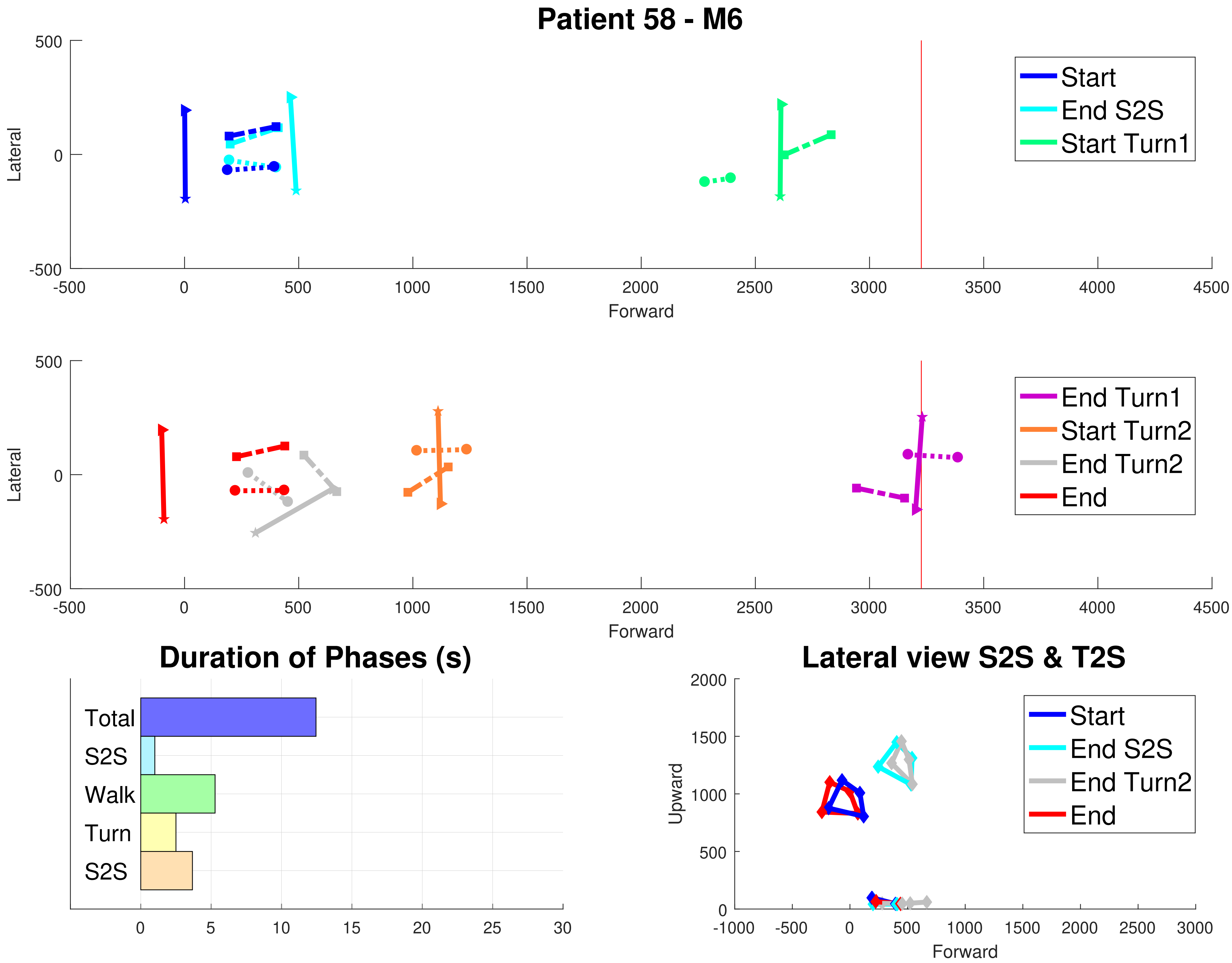

Patient 58 - M6
Lateral
Forward
Start
End S2S
Start Turn1
End Turn1
Start Turn2
End Turn2
End
Duration of Phases (s)
Total
S2S
Walk
Turn
S2S
Lateral view S2S & T2S
Upward
Forward
Start
End S2S
End Turn2
End

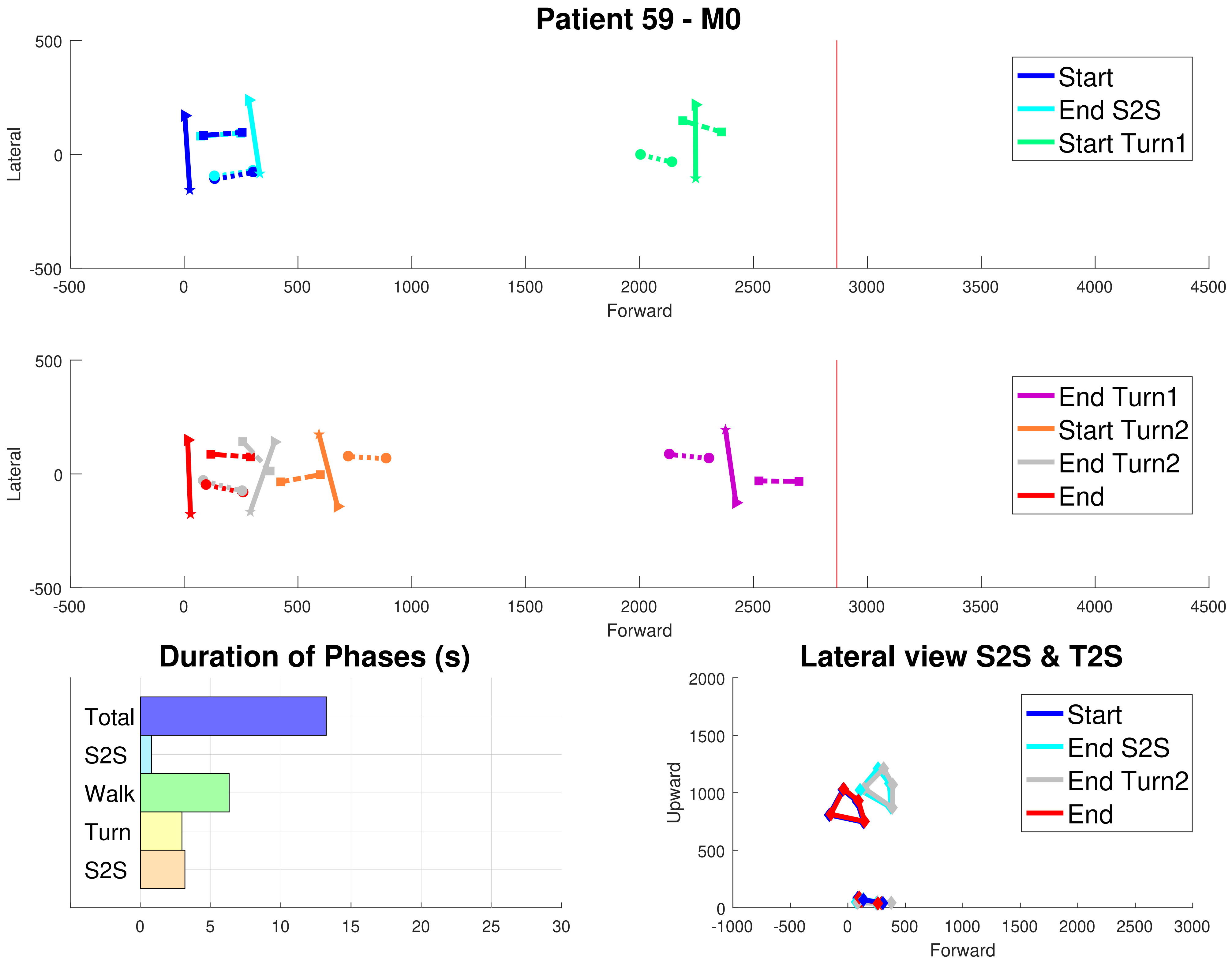

Patient 59 - M0
Lateral
Forward
Start
End S2S
Start Turn1
End Turn1
Start Turn2
End Turn2
End
Duration of Phases (s)
Total
S2S
Walk
Turn
S2S
Lateral view S2S & T2S
Upward
Start
End S2S
End Turn2
End

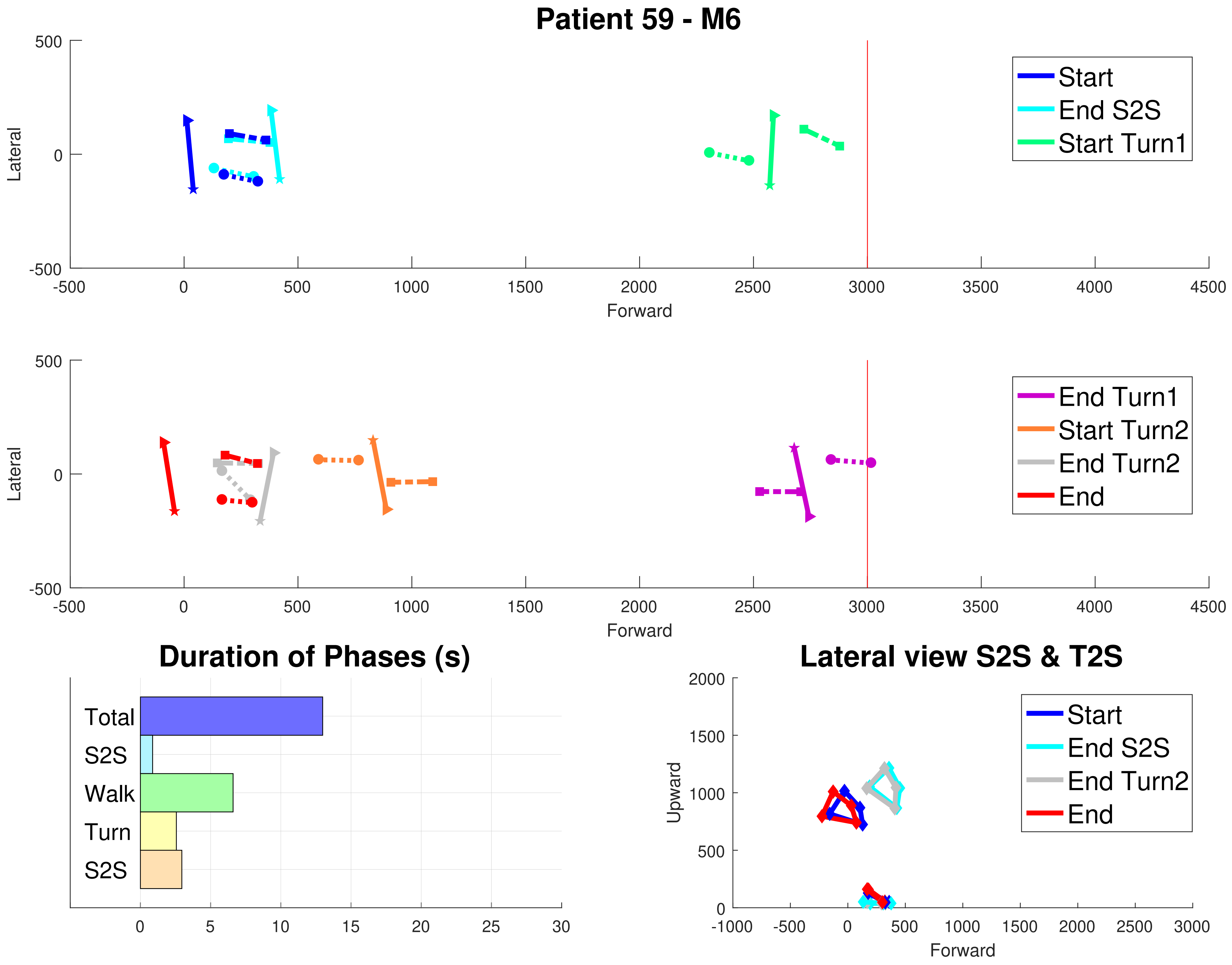

Patient 59 - M6
Lateral
Forward
Start
End S2S
Start Turn1
End Turn1
Start Turn2
End Turn2
End
Duration of Phases (s)
Total
S2S
Walk
Turn
S2S
Lateral view S2S & T2S
Upward
Forward
Start
End S2S
End Turn2
End

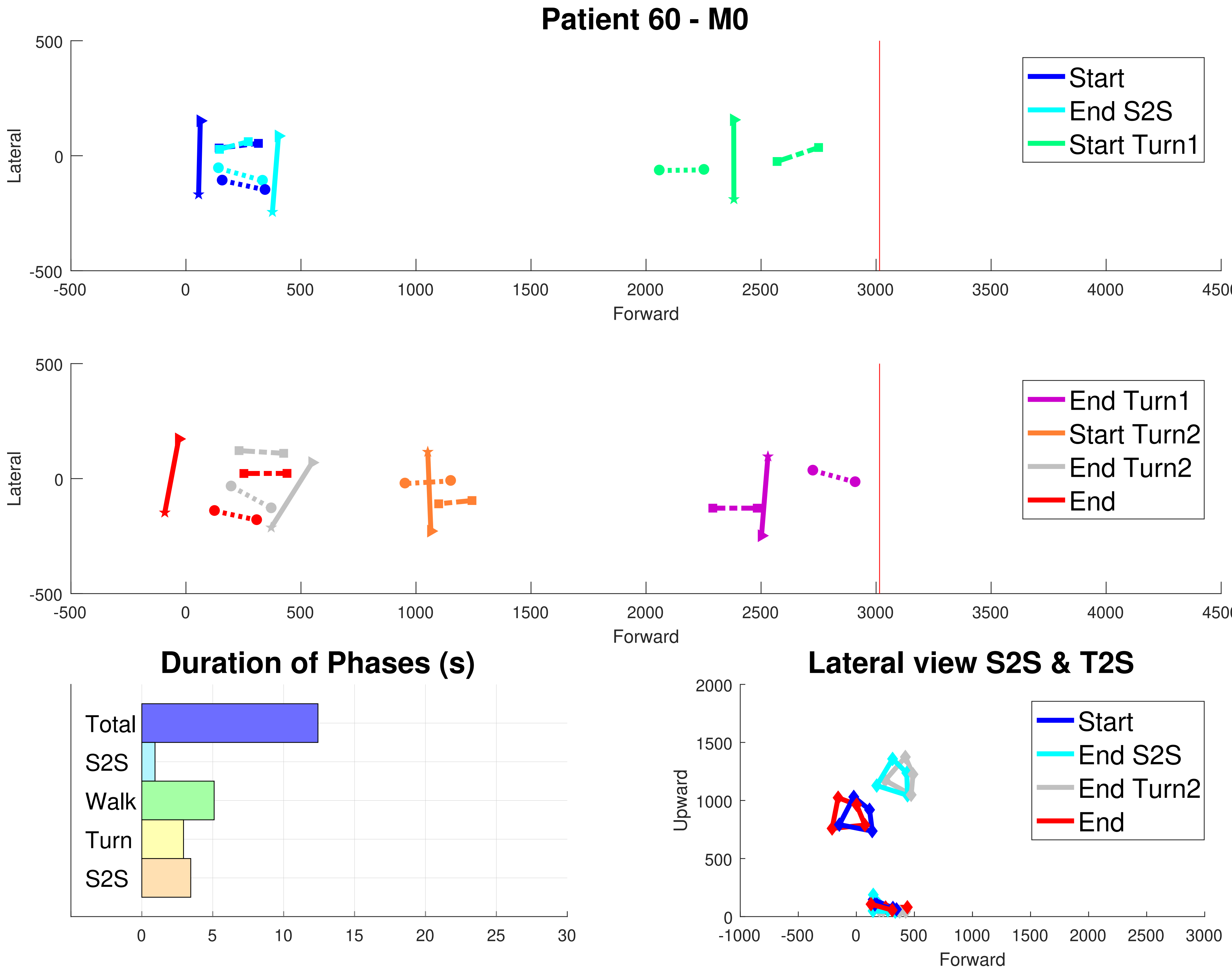

Patient 60 - M0
Lateral
Forward
Start
End S2S
Start Turn1
End Turn1
Start Turn2
End Turn2
End
Duration of Phases (s)
Total
S2S
Walk
Turn
S2S
Lateral view S2S & T2S
Upward
Forward
Start
End S2S
End Turn2
End

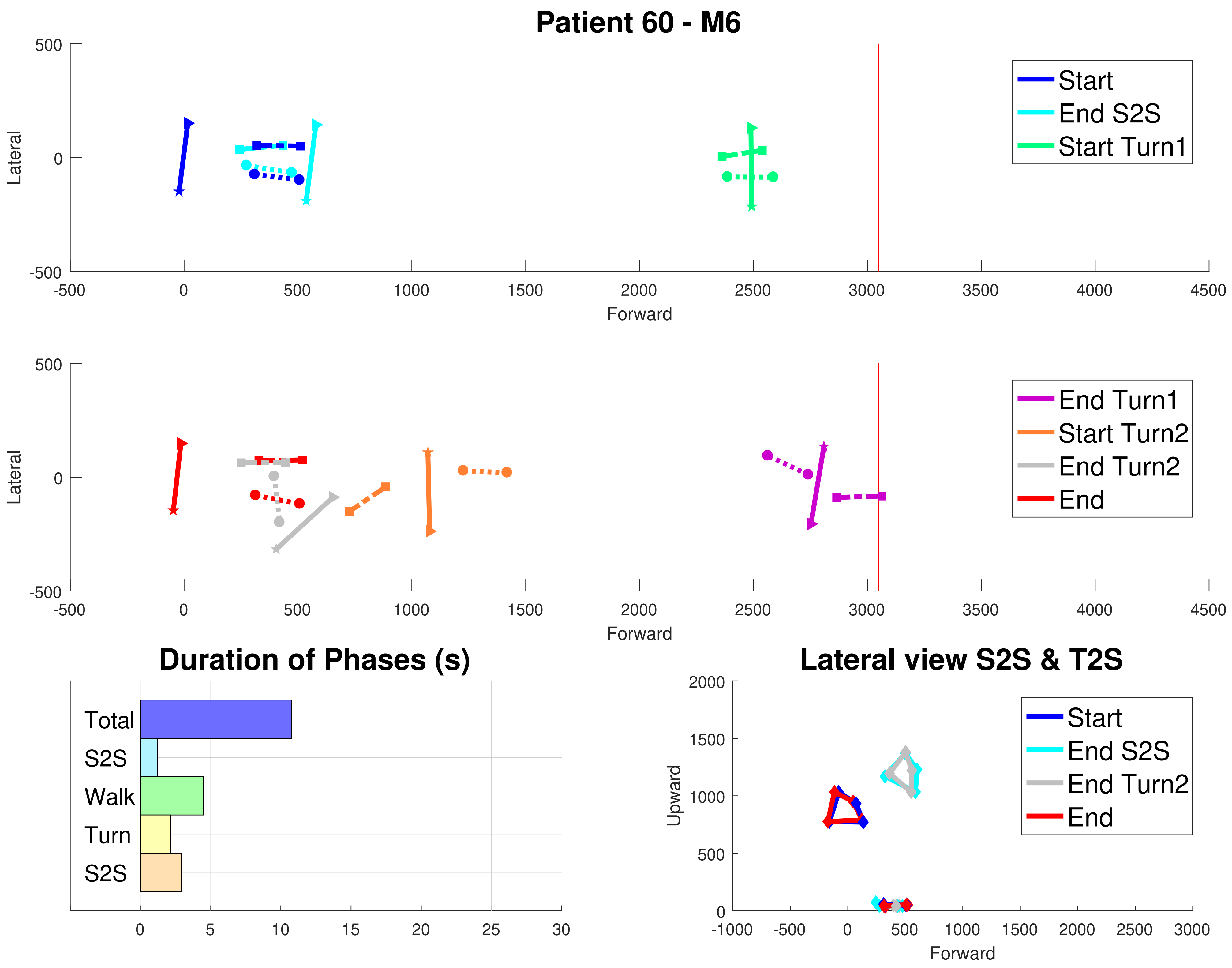
Patient 60 - M6
Lateral
Forward
Start
End S2S
Start Turn1
End Turn1
Start Turn2
End Turn2
End
Duration of Phases (s)
Total
S2S
Walk
Turn
S2S
Lateral view S2S & T2S
Upward
Forward
Start
End S2S
End Turn2
End

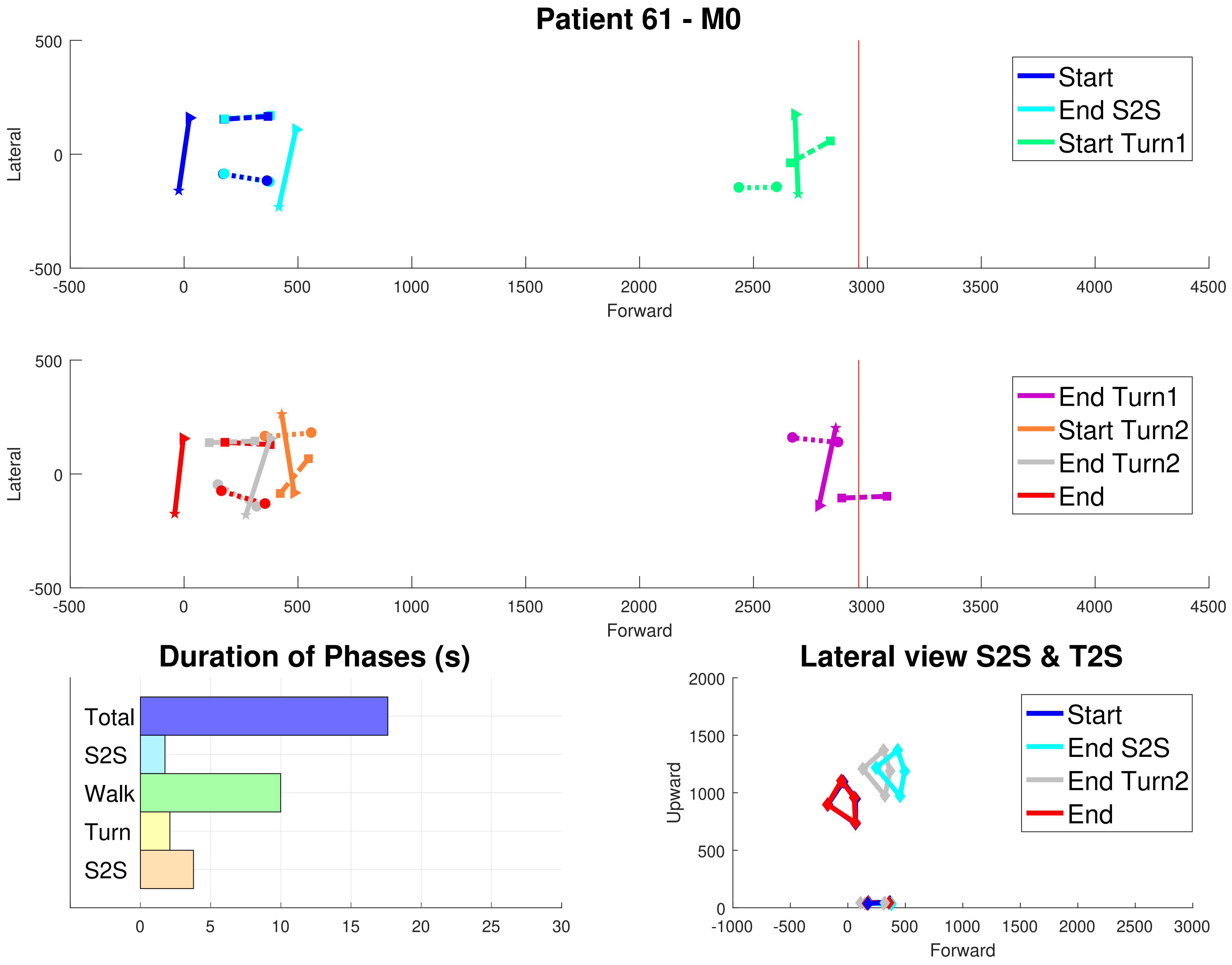
Patient 61 - M0
Start
End S2S
Start Turn1
Lateral
Forward
End Turn1
Start Turn2
End Turn2
End
Duration of Phases (s)
Total
S2S
Walk
Turn
S2S
Lateral view S2S & T2S
Upward

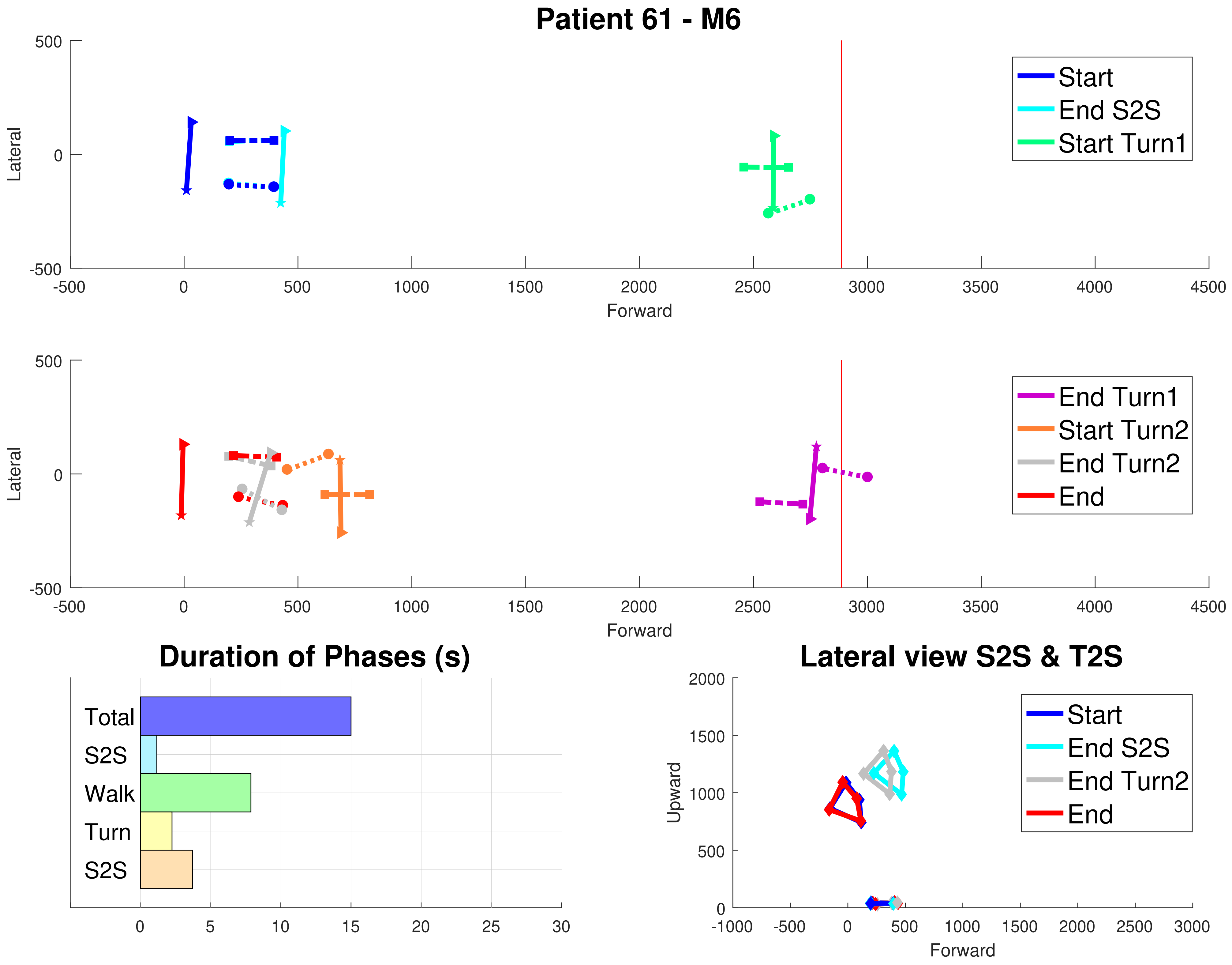
Patient 61 - M6
Lateral
Forward
Start
End S2S
Start Turn1
End Turn1
Start Turn2
End Turn2
End
Duration of Phases (s)
Total
S2S
Walk
Turn
S2S
Lateral view S2S & T2S
Upward
Forward
Start
End S2S
End Turn2
End

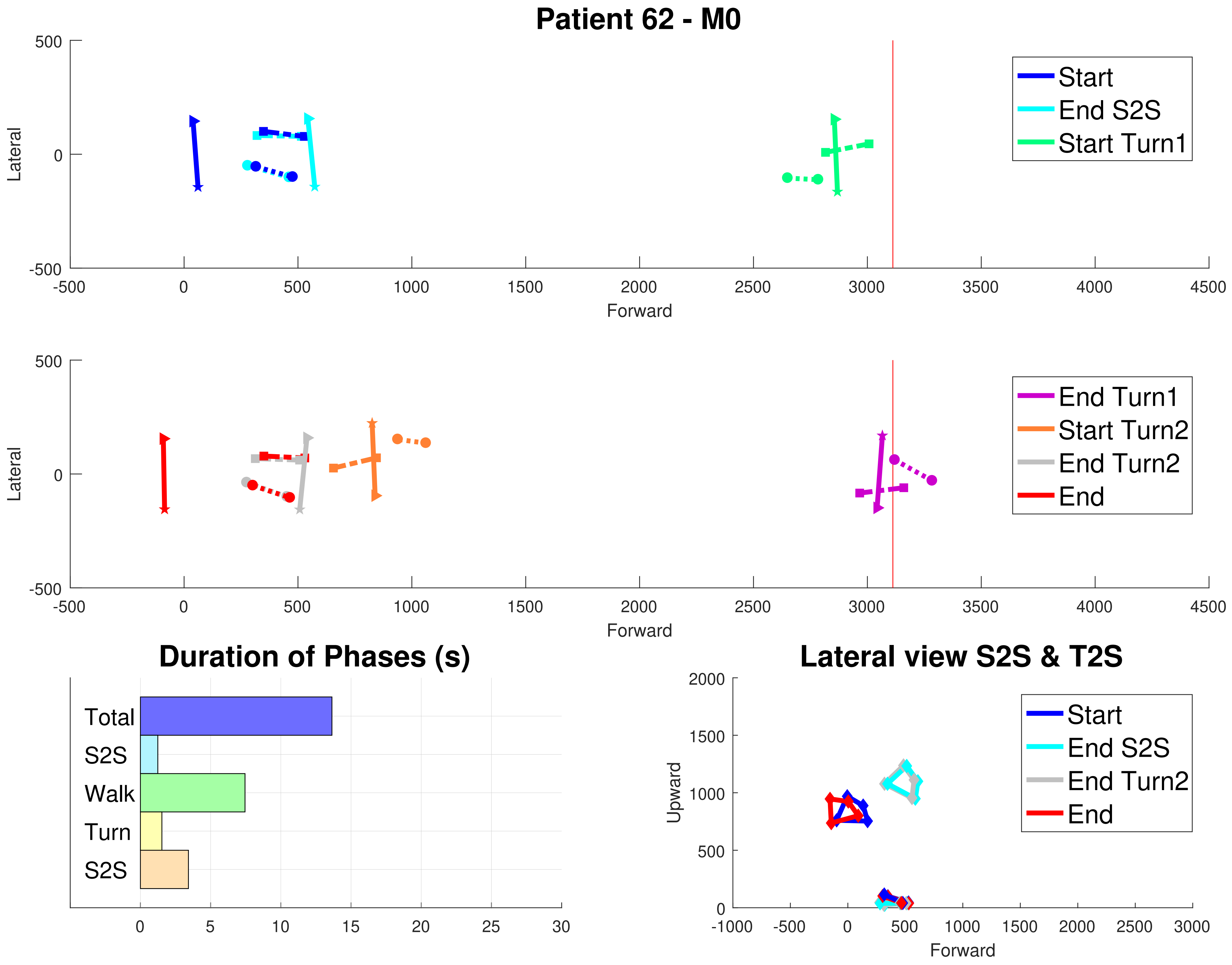

Patient 62 - M0
Lateral
Forward
Start
End S2S
Start Turn1
End Turn1
Start Turn2
End Turn2
End
Duration of Phases (s)
Total
S2S
Walk
Turn
S2S
Lateral view S2S & T2S
Upward
Forward
Start
End S2S
End Turn2
End

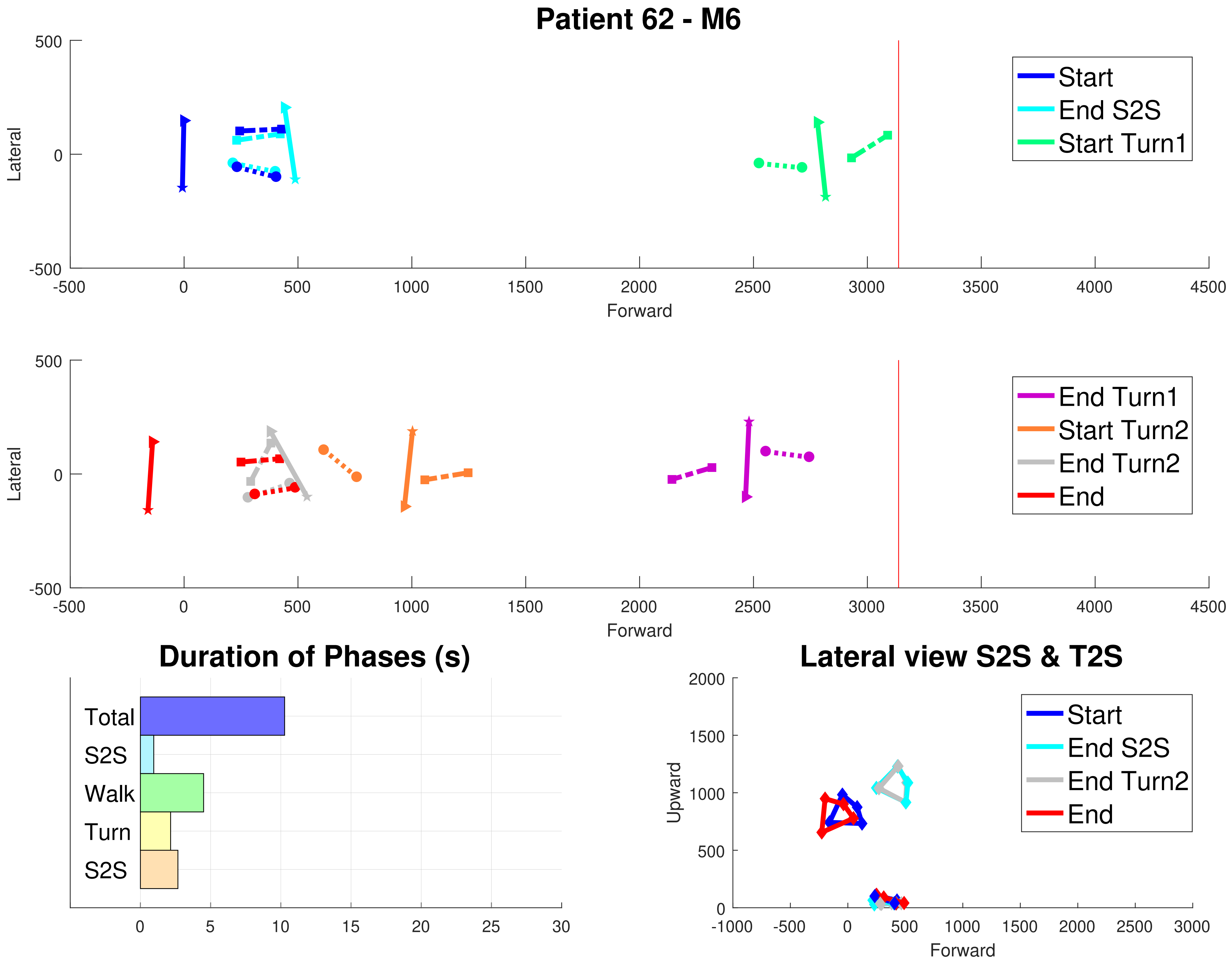
Patient 62 - M6
Lateral
Forward
Start
End S2S
Start Turn1
End Turn1
Start Turn2
End Turn2
End
Duration of Phases (s)
Total
S2S
Walk
Turn
S2S
Lateral view S2S & T2S
Upward
Forward

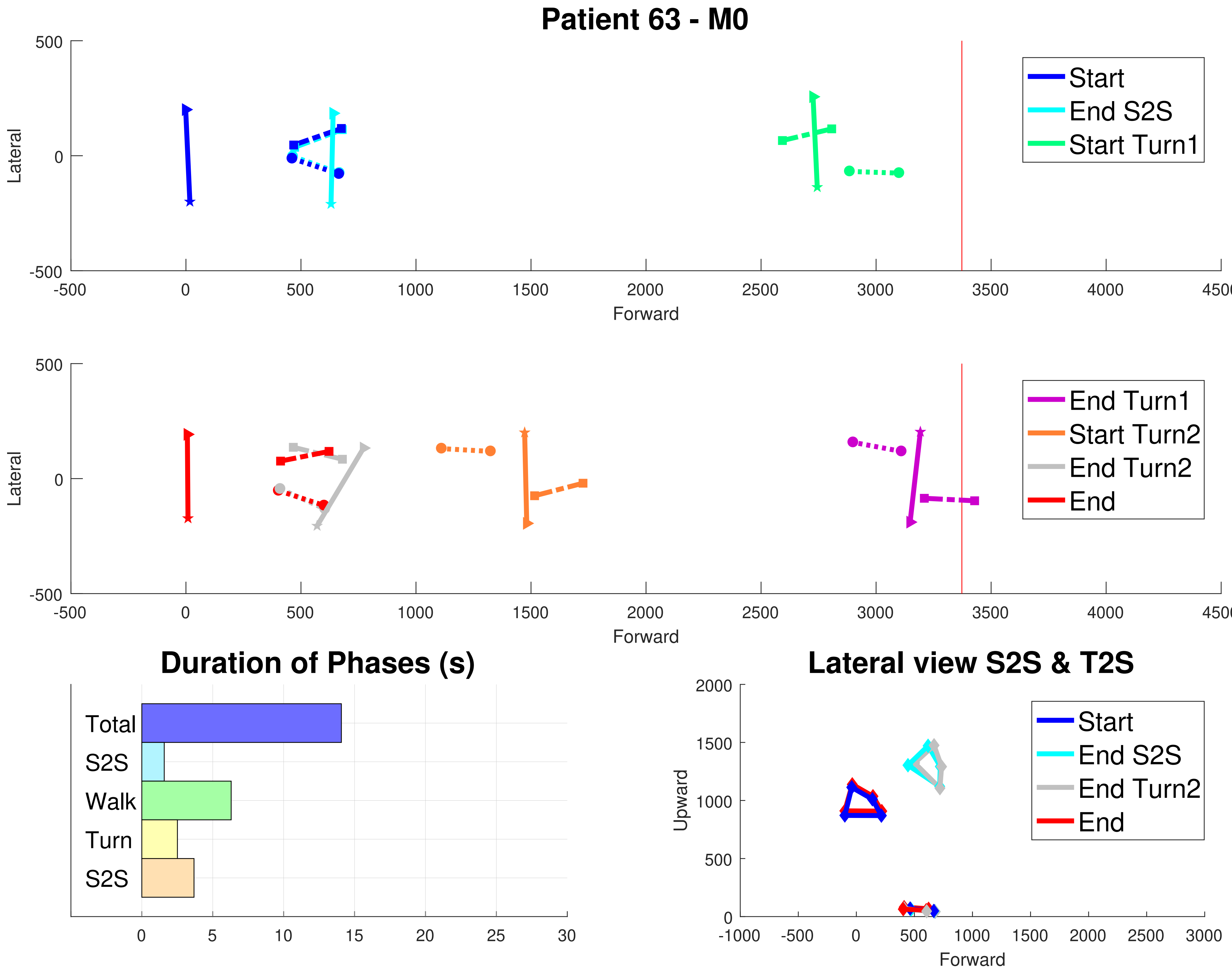
Patient 63 - M0
Lateral
Forward
Start
End S2S
Start Turn1
End Turn1
Start Turn2
End Turn2
End
Duration of Phases (s)
Total
S2S
Walk
Turn
S2S
Lateral view S2S & T2S
Upward
Forward
Start
End S2S
End Turn2
End

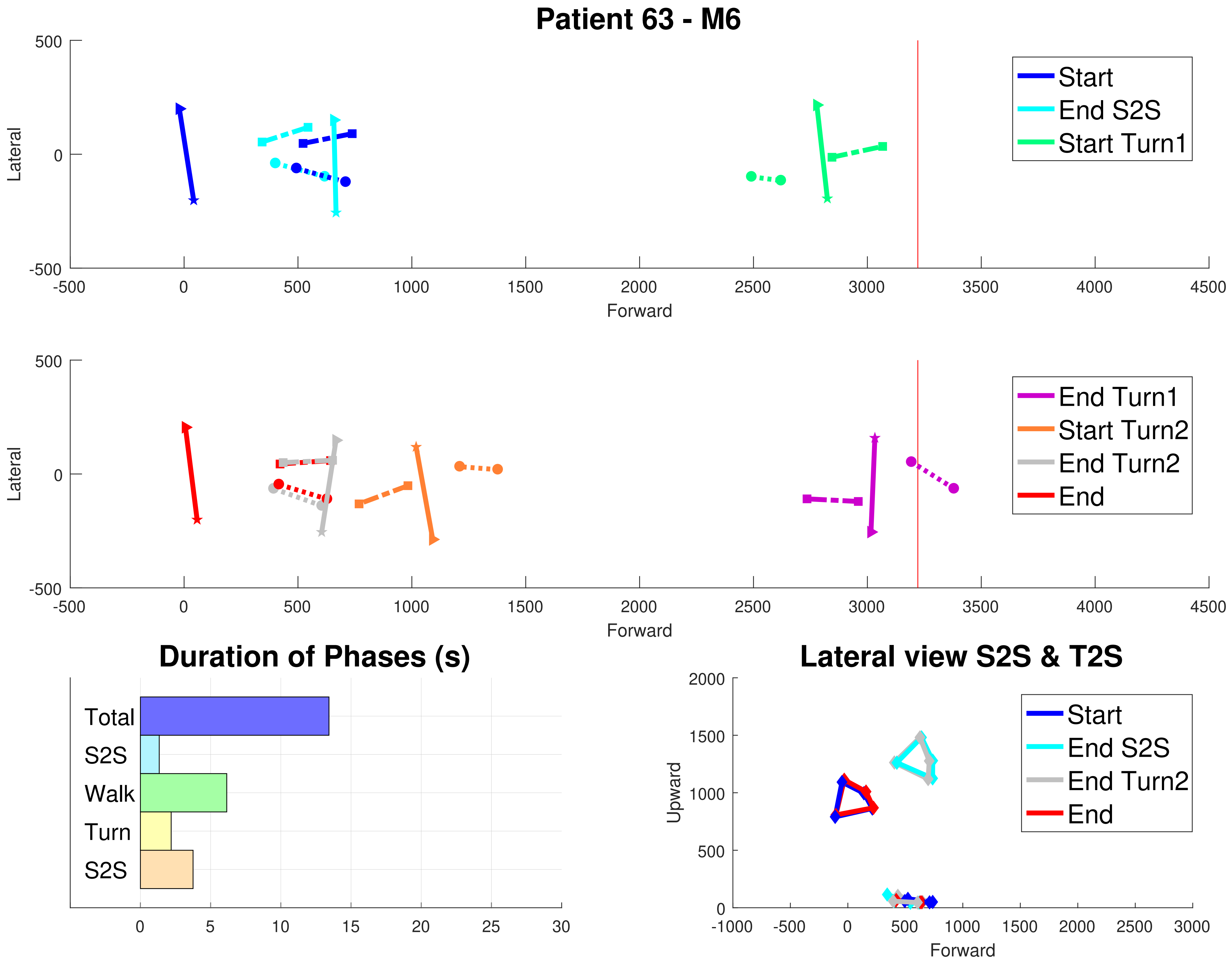

Patient 63 - M6
Lateral
Forward
Start
End S2S
Start Turn1
End Turn1
Start Turn2
End Turn2
End
Duration of Phases (s)
Total
S2S
Walk
Turn
S2S
Lateral view S2S & T2S
Upward
Forward
Start
End S2S
End Turn2
End

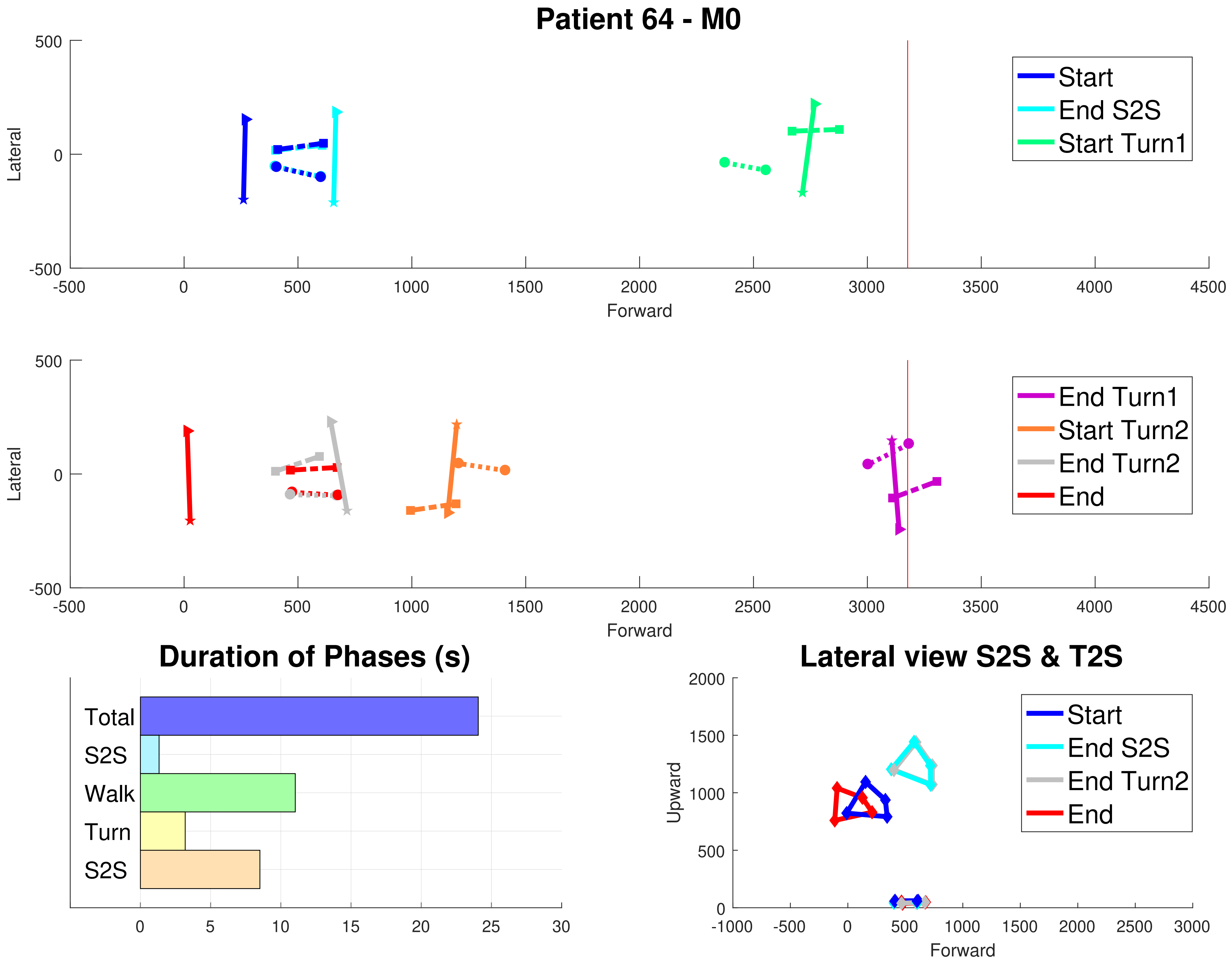
Patient 64 - M0
Lateral
Forward
Start
End S2S
Start Turn1
End Turn1
Start Turn2
End Turn2
End
Duration of Phases (s)
Total
S2S
Walk
Turn
S2S
Lateral view S2S & T2S
Upward
Forward
Start
End S2S
End Turn2
End

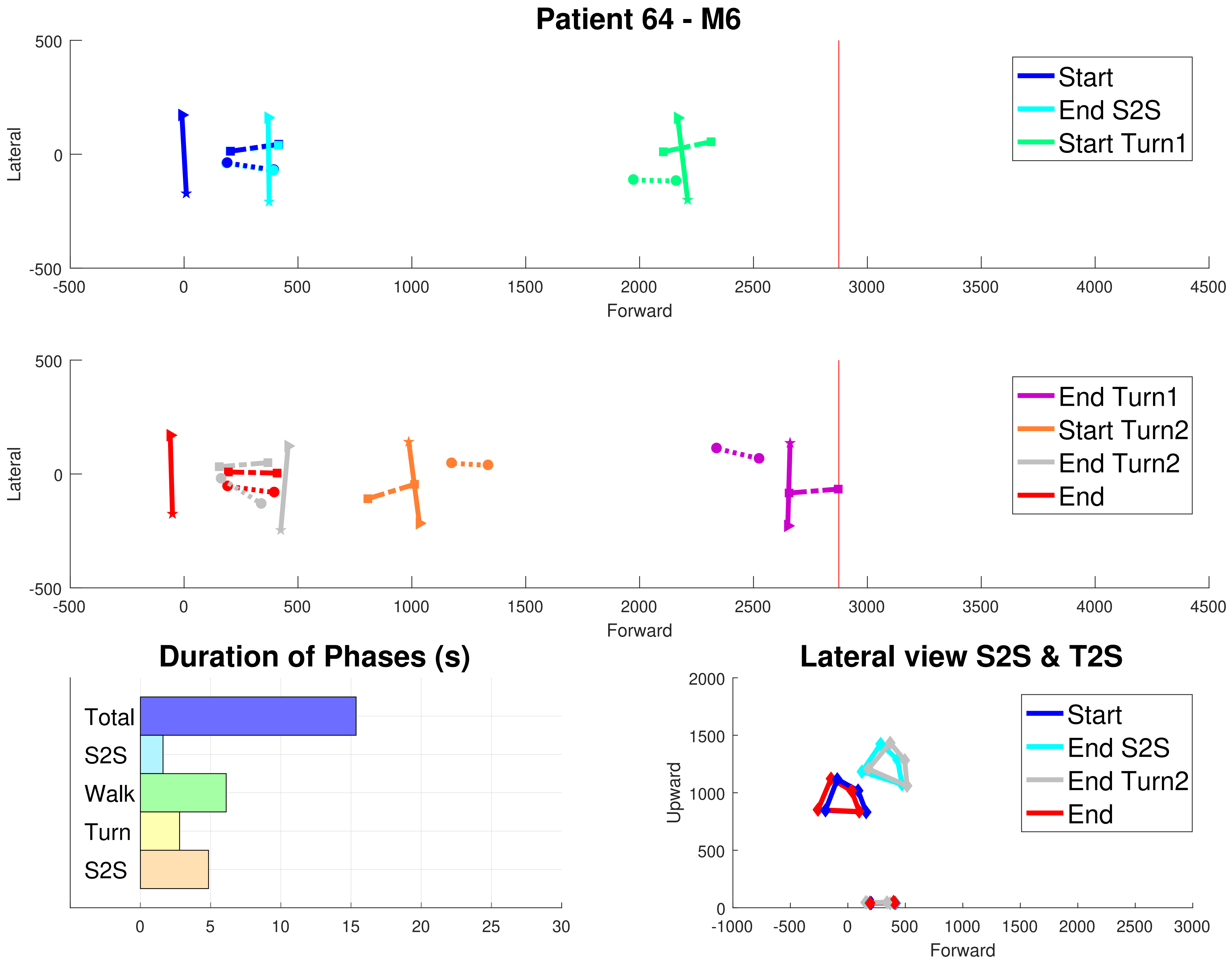

Patient 64 - M6
Lateral
Forward
500
0
-500
-500
0
500
1000
1500
2000
2500
3000
3500
4000
4500
Start
End S2S
Start Turn1
End Turn1
Start Turn2
End Turn2
End
Duration of Phases (s)
Total
S2S
Walk
Turn
S2S
0
5
10
15
20
25
30
Lateral view S2S & T2S
Upward
2000
1500
1000
500
-1000
3000

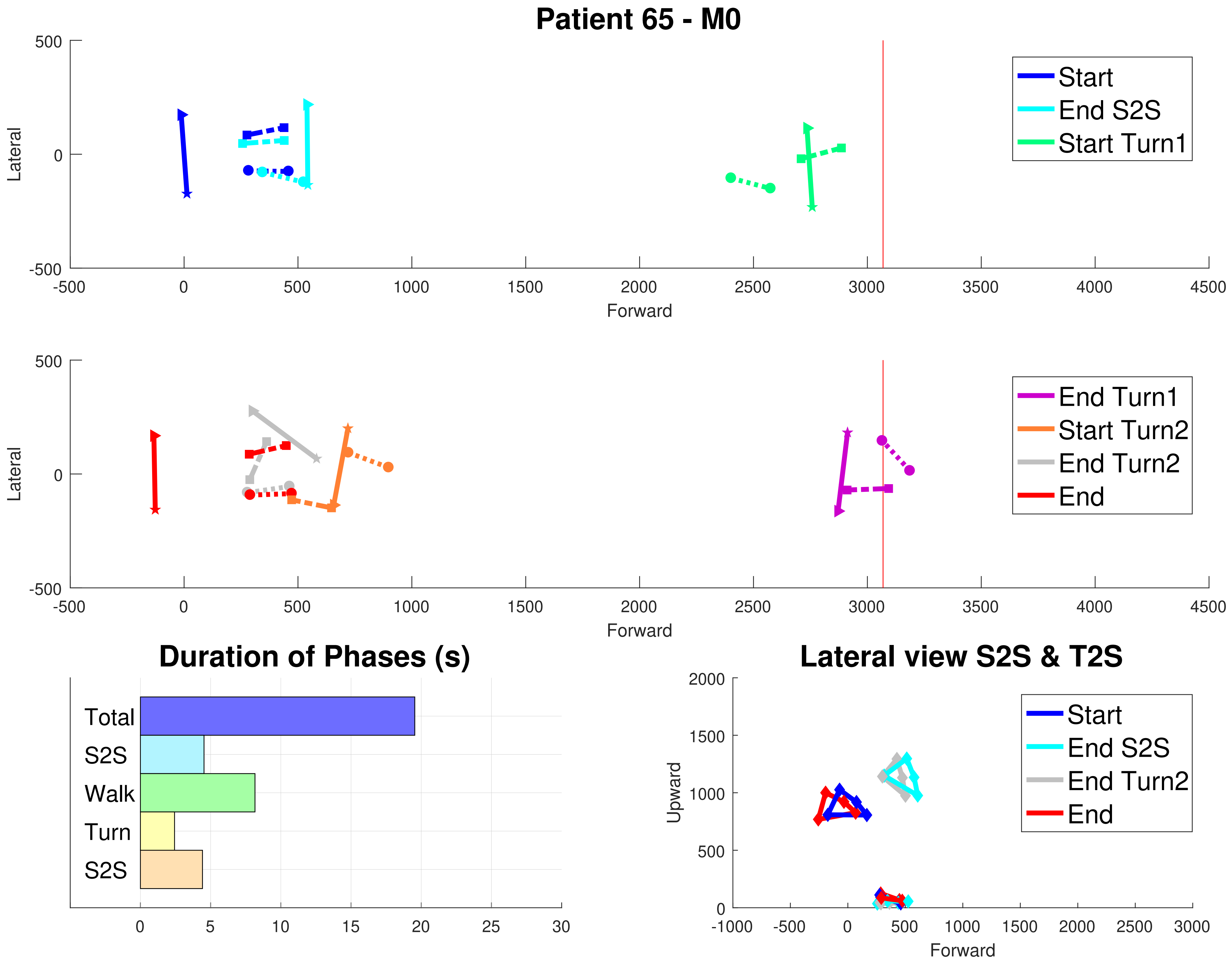

Patient 65 - M0
Lateral
Forward
Start
End S2S
Start Turn1
End Turn1
Start Turn2
End Turn2
End
Duration of Phases (s)
Total
S2S
Walk
Turn
S2S
Lateral view S2S & T2S
Upward

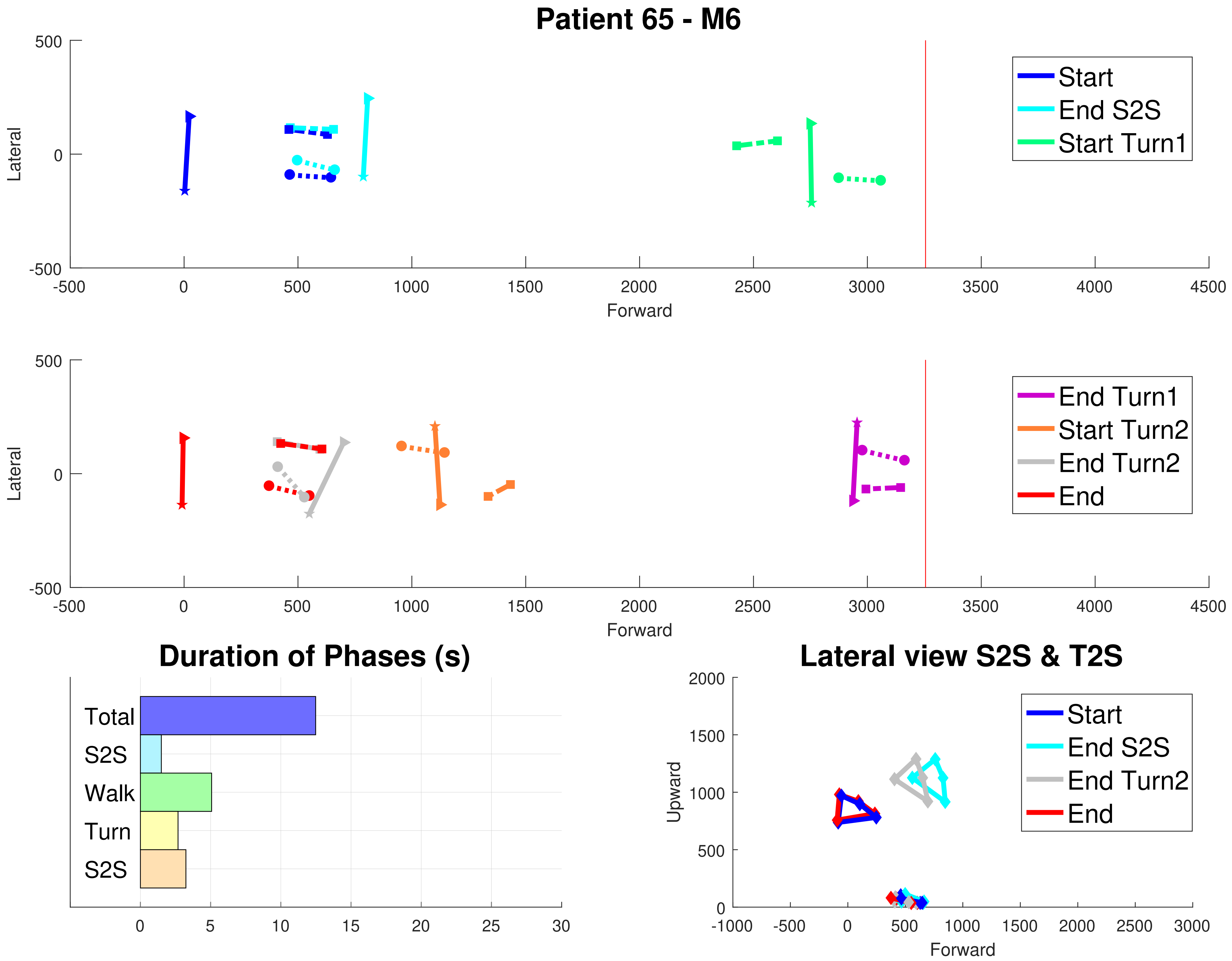

Patient 65 - M6
Lateral
Forward
Start
End S2S
Start Turn1
End Turn1
Start Turn2
End Turn2
End
Duration of Phases (s)
Total
S2S
Walk
Turn
S2S
Lateral view S2S & T2S
Upward
Forward
Start
End S2S
End Turn2
End

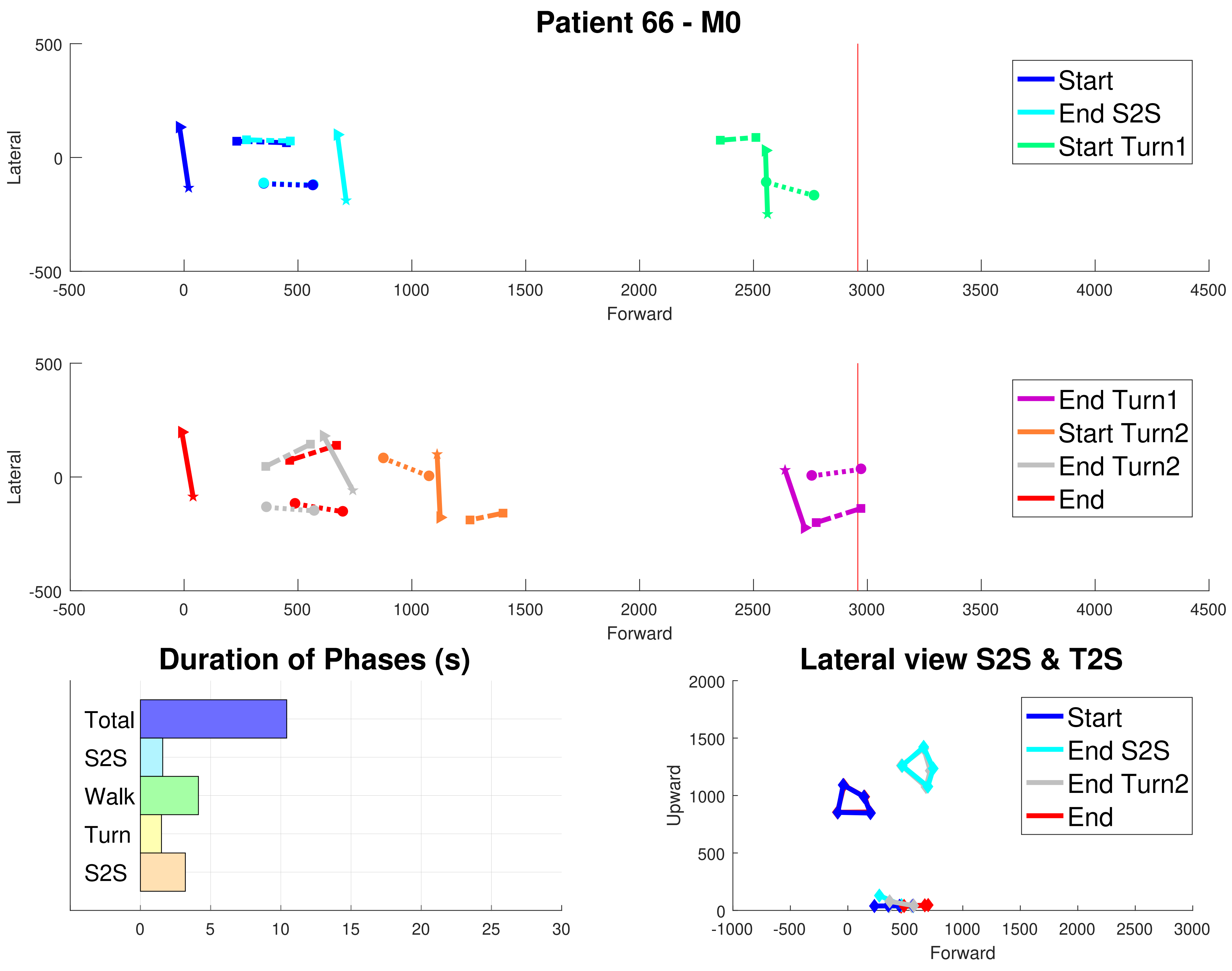

Patient 66 - M0
Lateral
Forward
Start
End S2S
Start Turn1
End Turn1
Start Turn2
End Turn2
End
Duration of Phases (s)
Total
S2S
Walk
Turn
S2S
Lateral view S2S & T2S
Upward
Forward
Start
End S2S
End Turn2
End

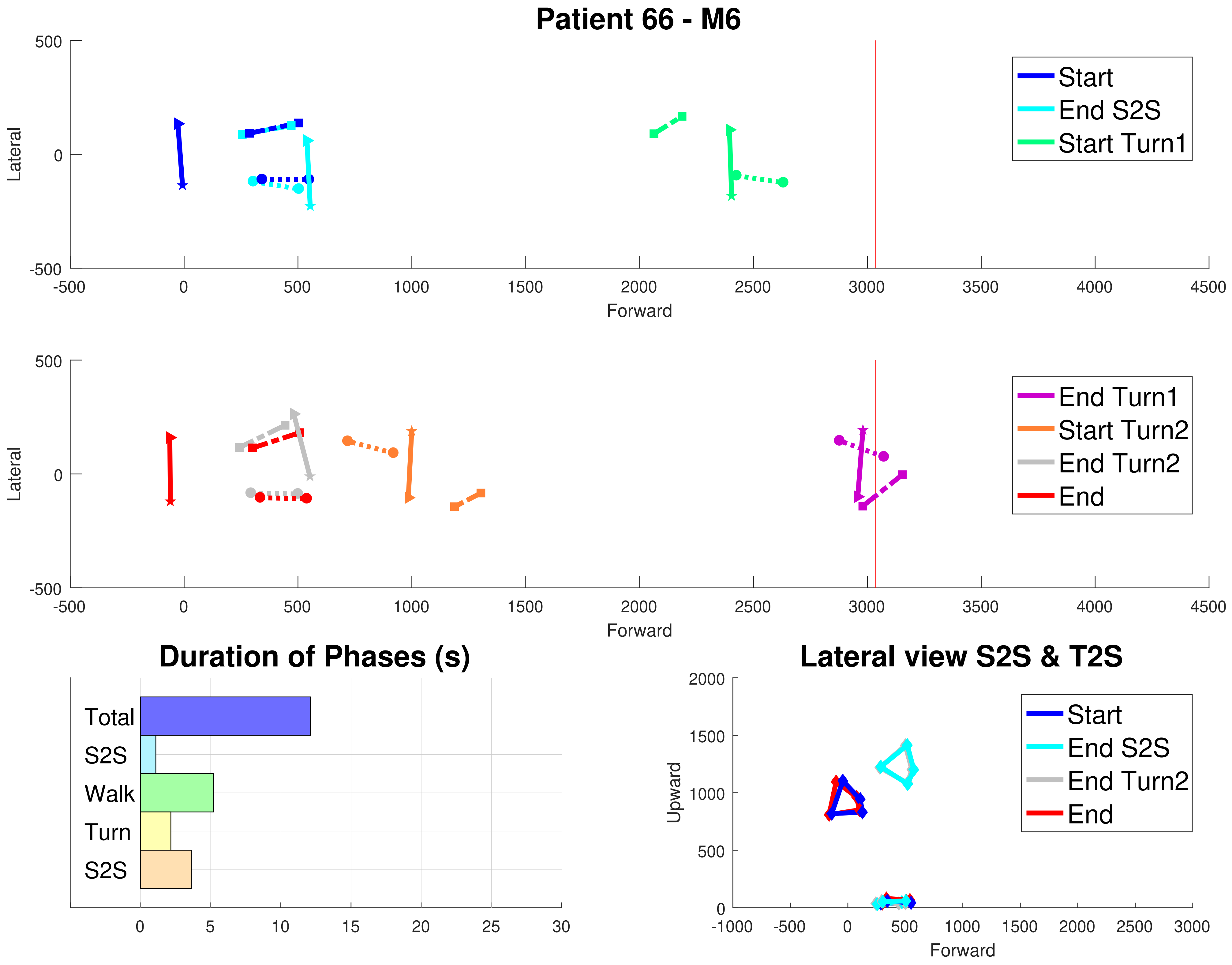

Patient 66 - M6
Lateral
Forward
Start
End S2S
Start Turn1
End Turn1
Start Turn2
End Turn2
End
Duration of Phases (s)
Total
S2S
Walk
Turn
S2S
Lateral view S2S & T2S
Upward
Forward
Start
End S2S
End Turn2
End

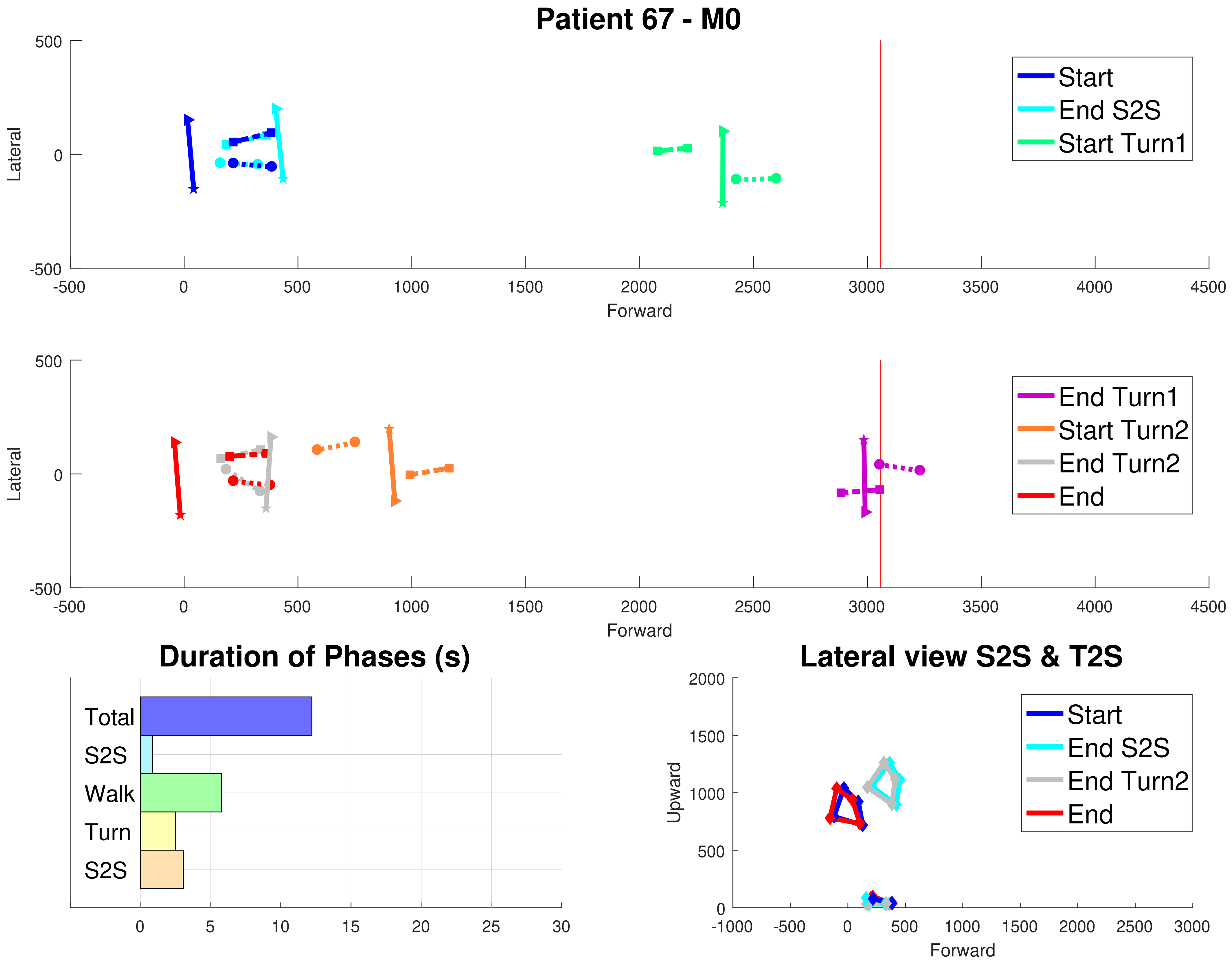
Patient 67 - M0
Lateral
Forward
Start
End S2S
Start Turn1
End Turn1
Start Turn2
End Turn2
End
Duration of Phases (s)
Total
S2S
Walk
Turn
S2S
Lateral view S2S & T2S
Upward
Start
End S2S
End Turn2
End

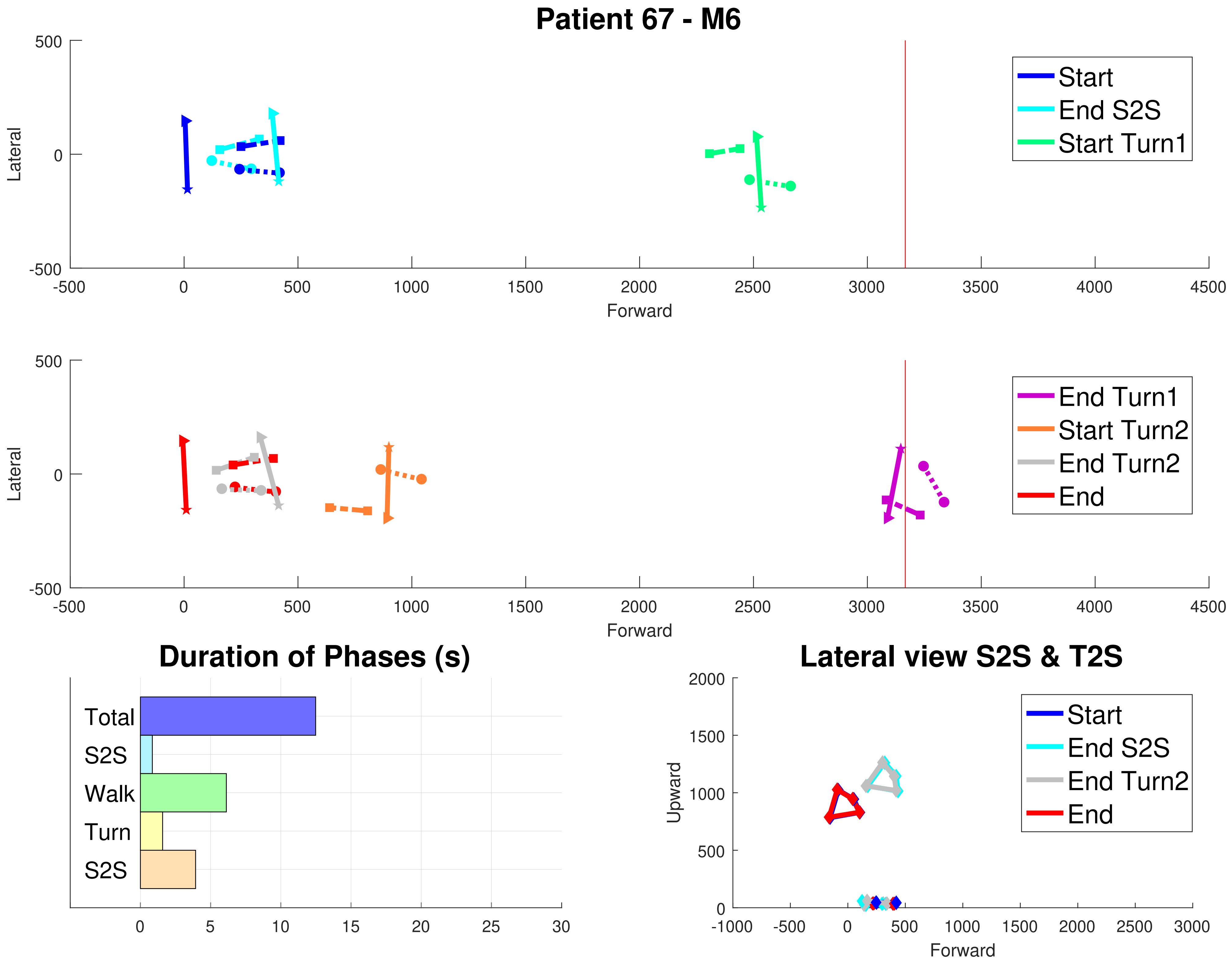
Patient 67 - M6
Lateral
Forward
Start
End S2S
Start Turn1
End Turn1
Start Turn2
End Turn2
End
Duration of Phases (s)
Total
S2S
Walk
Turn
S2S
Lateral view S2S & T2S
Upward
Forward
Start
End S2S
End Turn2
End

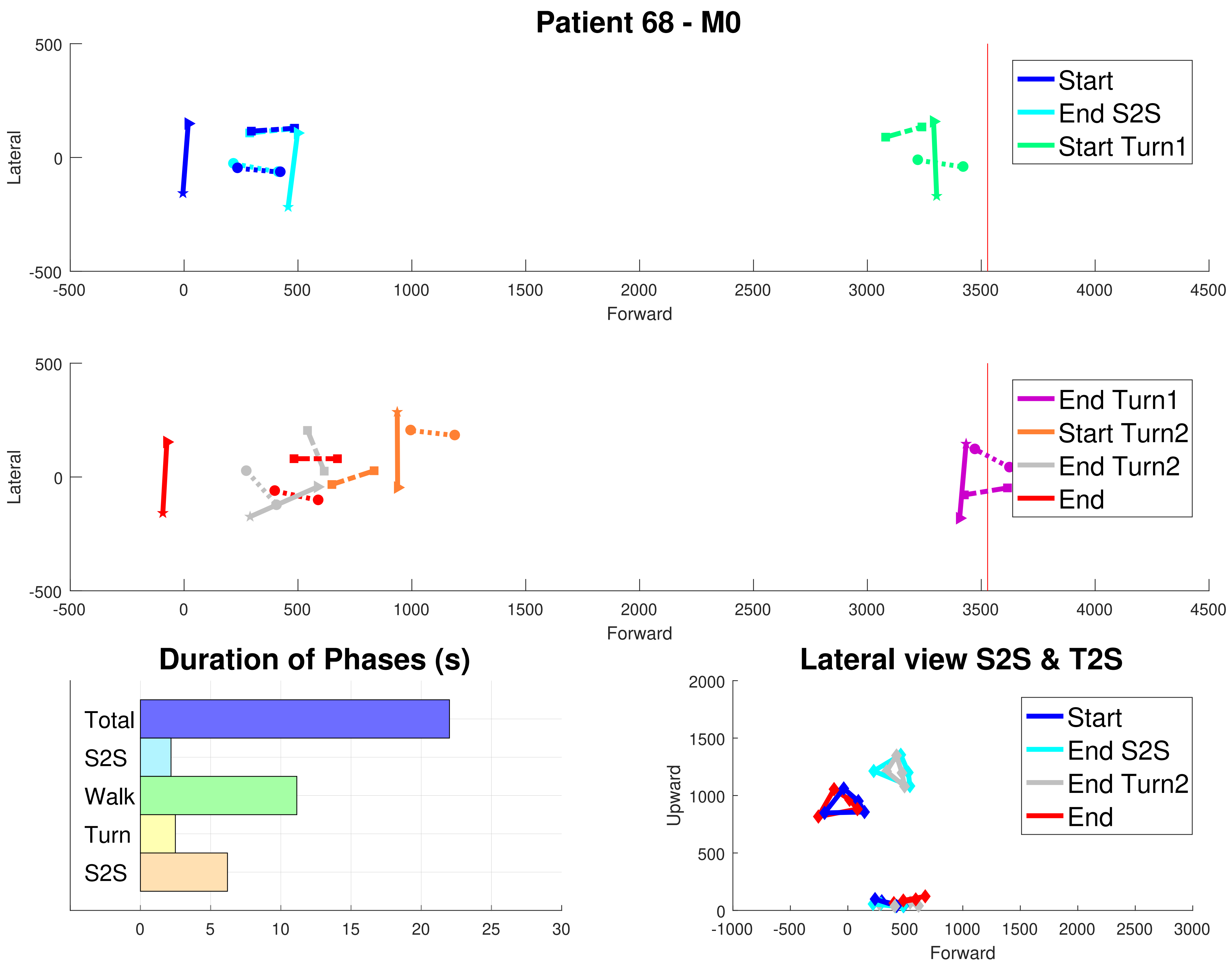

Patient 68 - M0
Lateral
Forward
Start
End S2S
Start Turn1
End Turn1
Start Turn2
End Turn2
End
Duration of Phases (s)
Total
S2S
Walk
Turn
S2S
Lateral view S2S & T2S
Upward
Forward
Start
End S2S
End Turn2
End

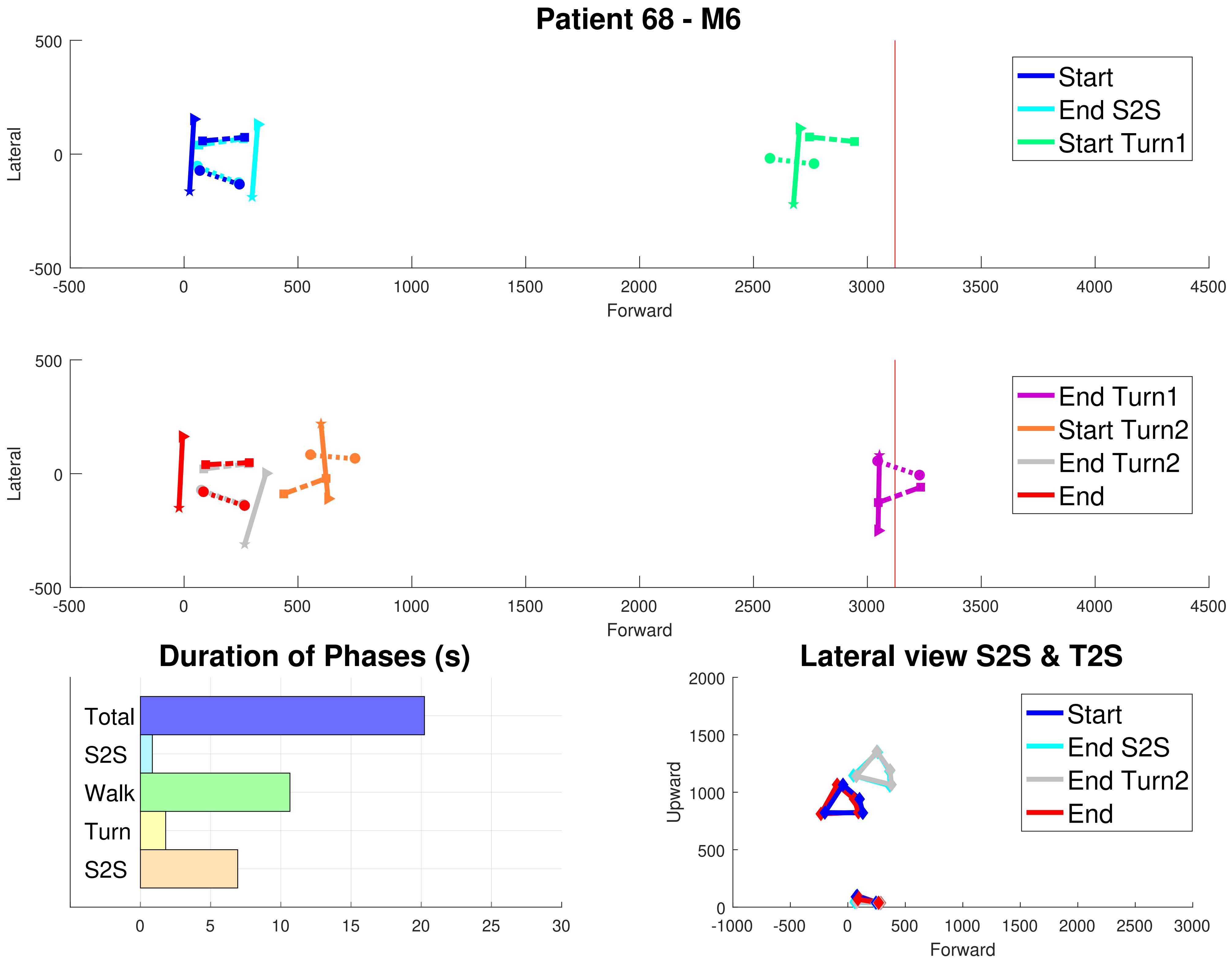

Patient 68 - M6
Lateral
Forward
Start
End S2S
Start Turn1
End Turn1
Start Turn2
End Turn2
End
Duration of Phases (s)
Total
S2S
Walk
Turn
S2S
Lateral view S2S & T2S
Upward

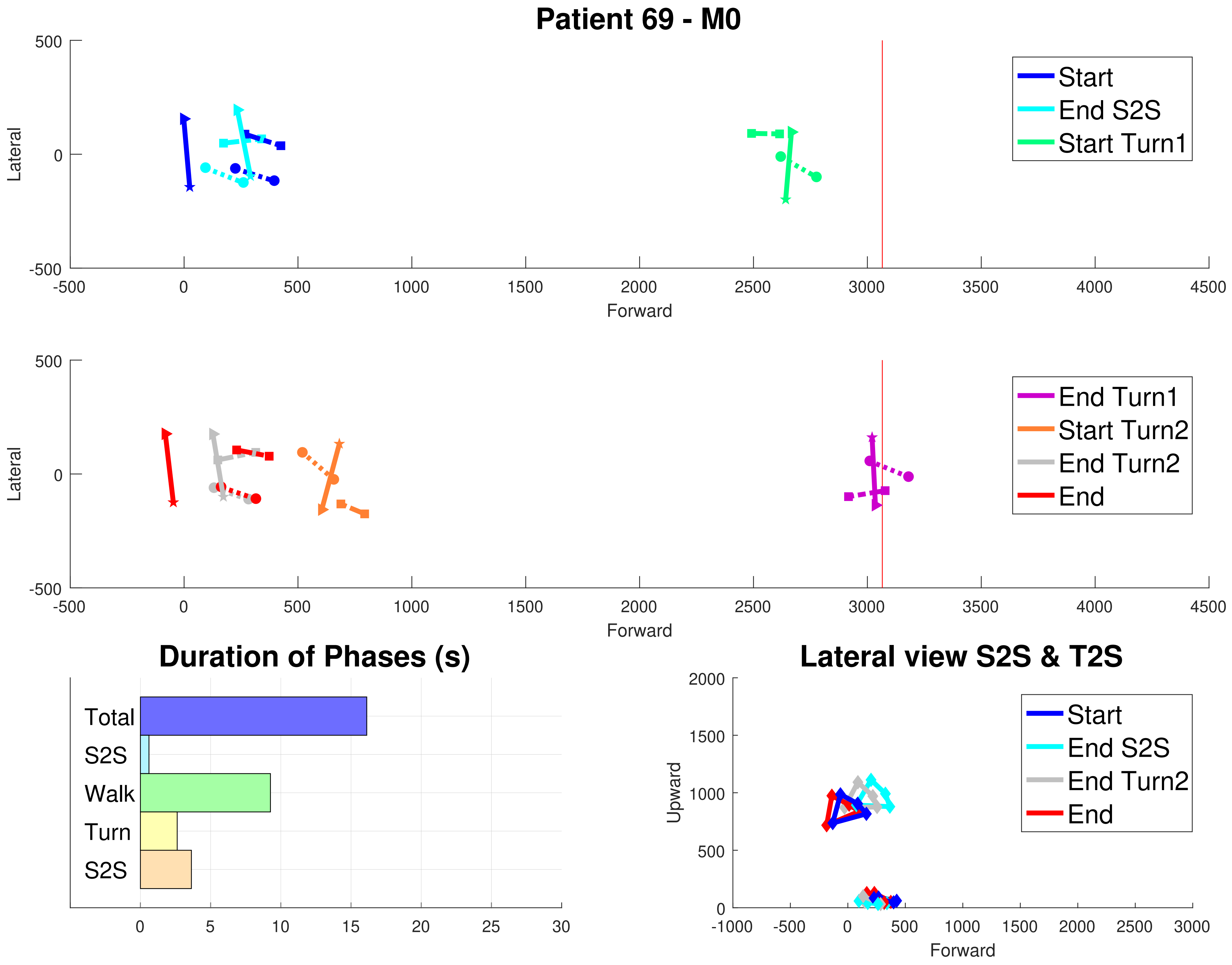

Patient 69 - M0
Lateral
Forward
Start
End S2S
Start Turn1
End Turn1
Start Turn2
End Turn2
End
Duration of Phases (s)
Total
S2S
Walk
Turn
S2S
Lateral view S2S & T2S
Upward

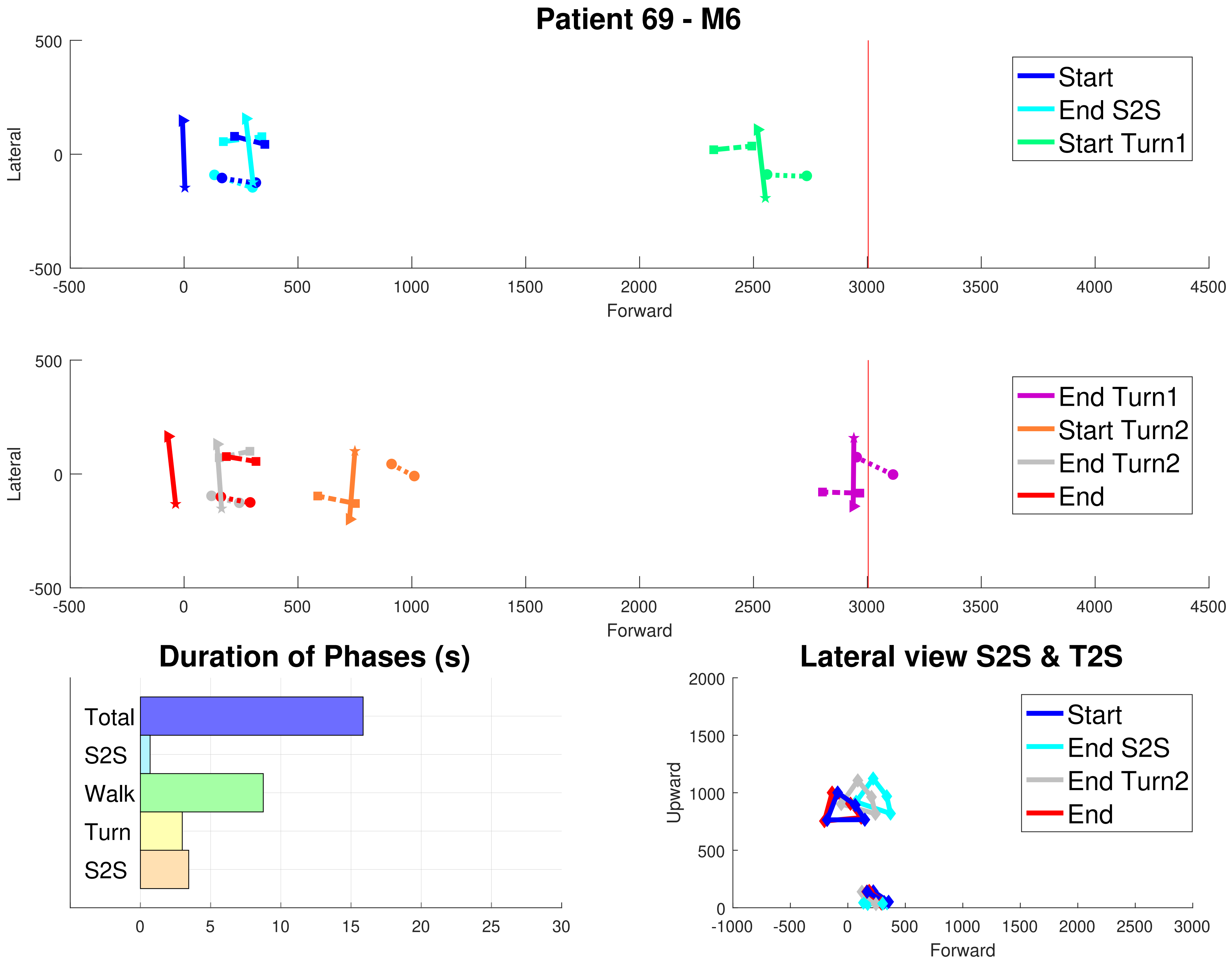

Patient 69 - M6
Lateral
Forward
Start
End S2S
Start Turn1
End Turn1
Start Turn2
End Turn2
End
Duration of Phases (s)
Total
S2S
Walk
Turn
S2S
Lateral view S2S & T2S
Upward
Forward
Start
End S2S
End Turn2
End

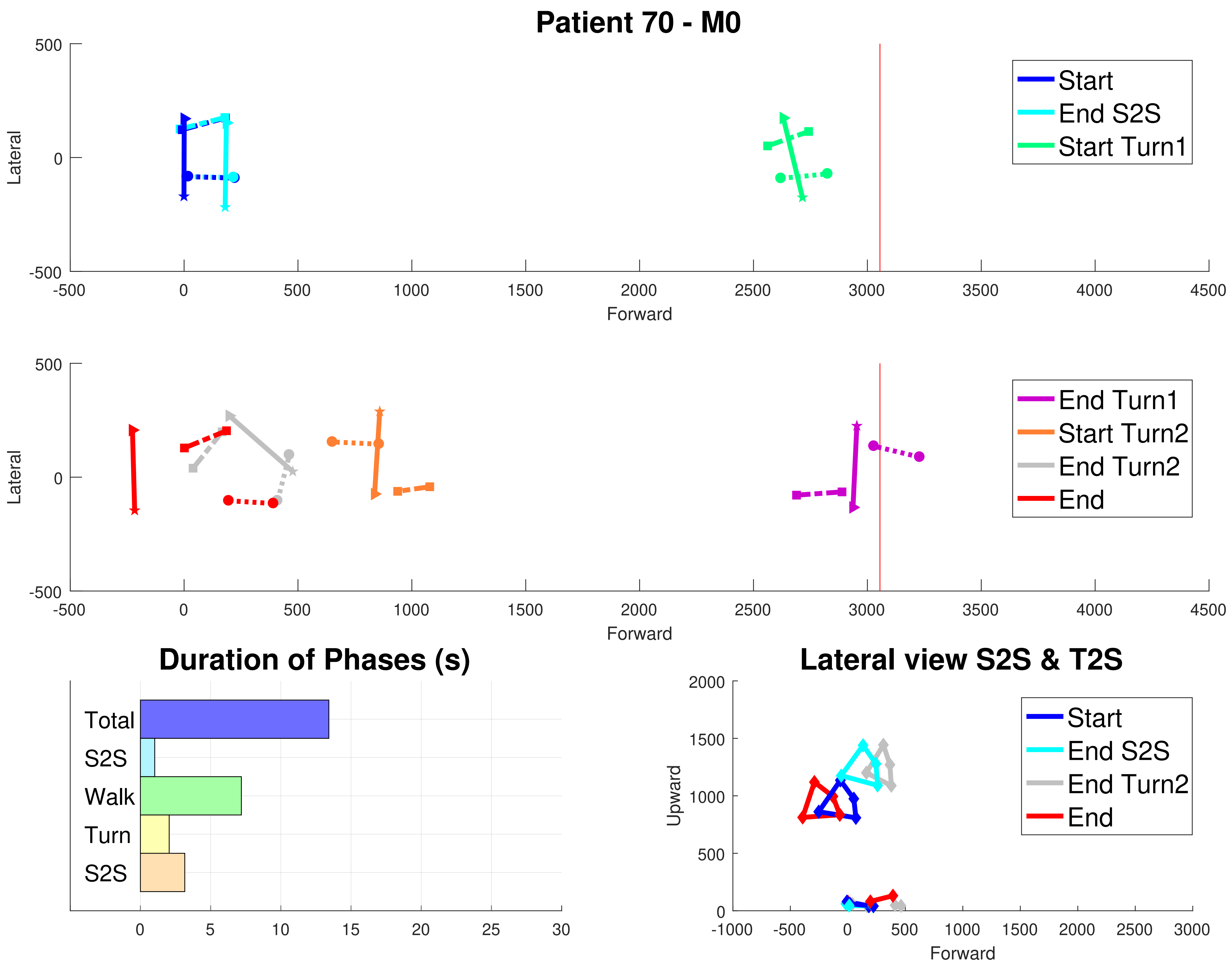
Patient 70 - M0
Lateral
Forward
Start
End S2S
Start Turn1
End Turn1
Start Turn2
End Turn2
End
Duration of Phases (s)
Total
S2S
Walk
Turn
S2S
Lateral view S2S & T2S
Upward
Forward
Start
End S2S
End Turn2
End

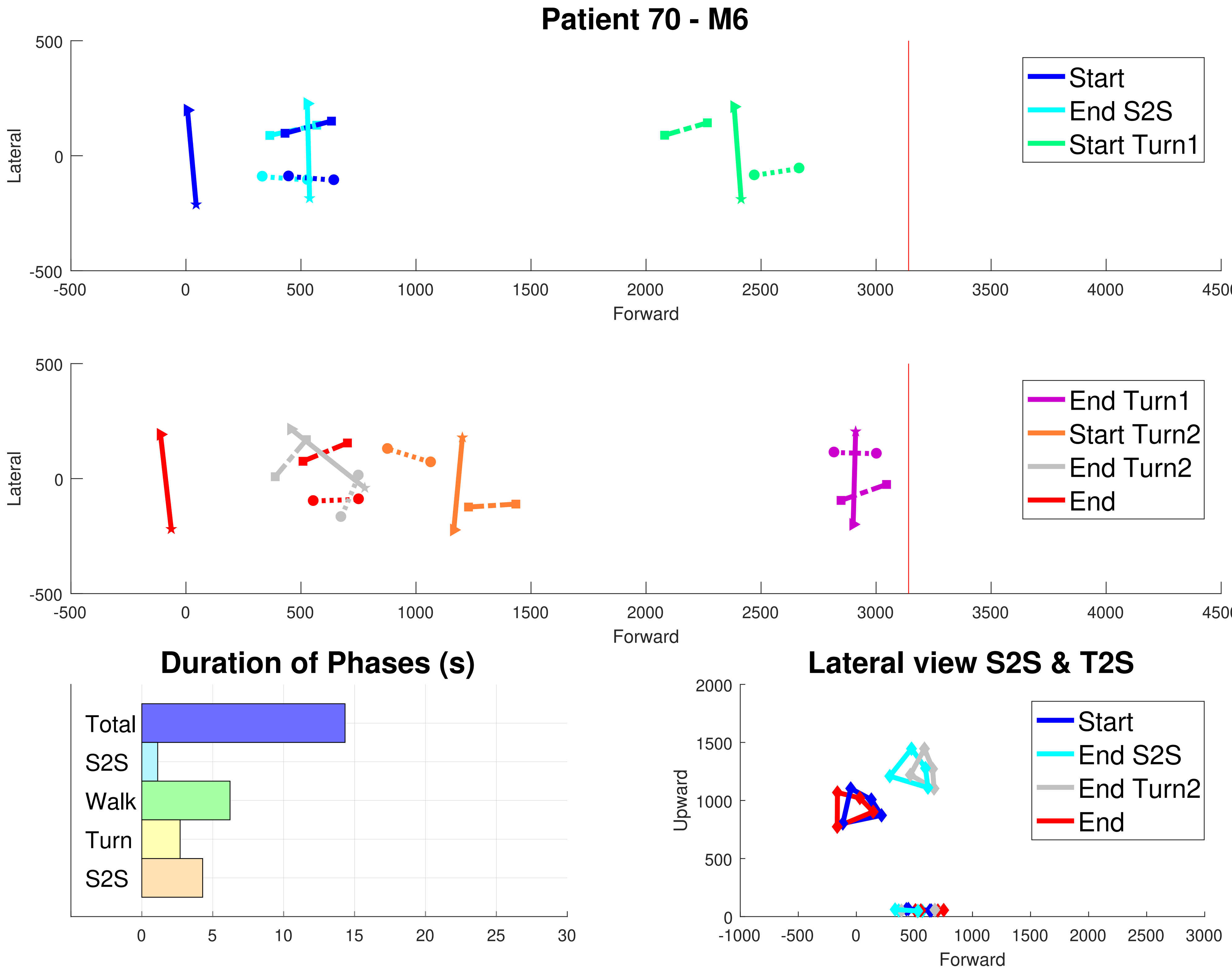

Patient 70 - M6
Lateral
Forward
Start
End S2S
Start Turn1
End Turn1
Start Turn2
End Turn2
End
Duration of Phases (s)
Total
S2S
Walk
Turn
S2S
Lateral view S2S & T2S
Upward
Forward
Start
End S2S
End Turn2
End

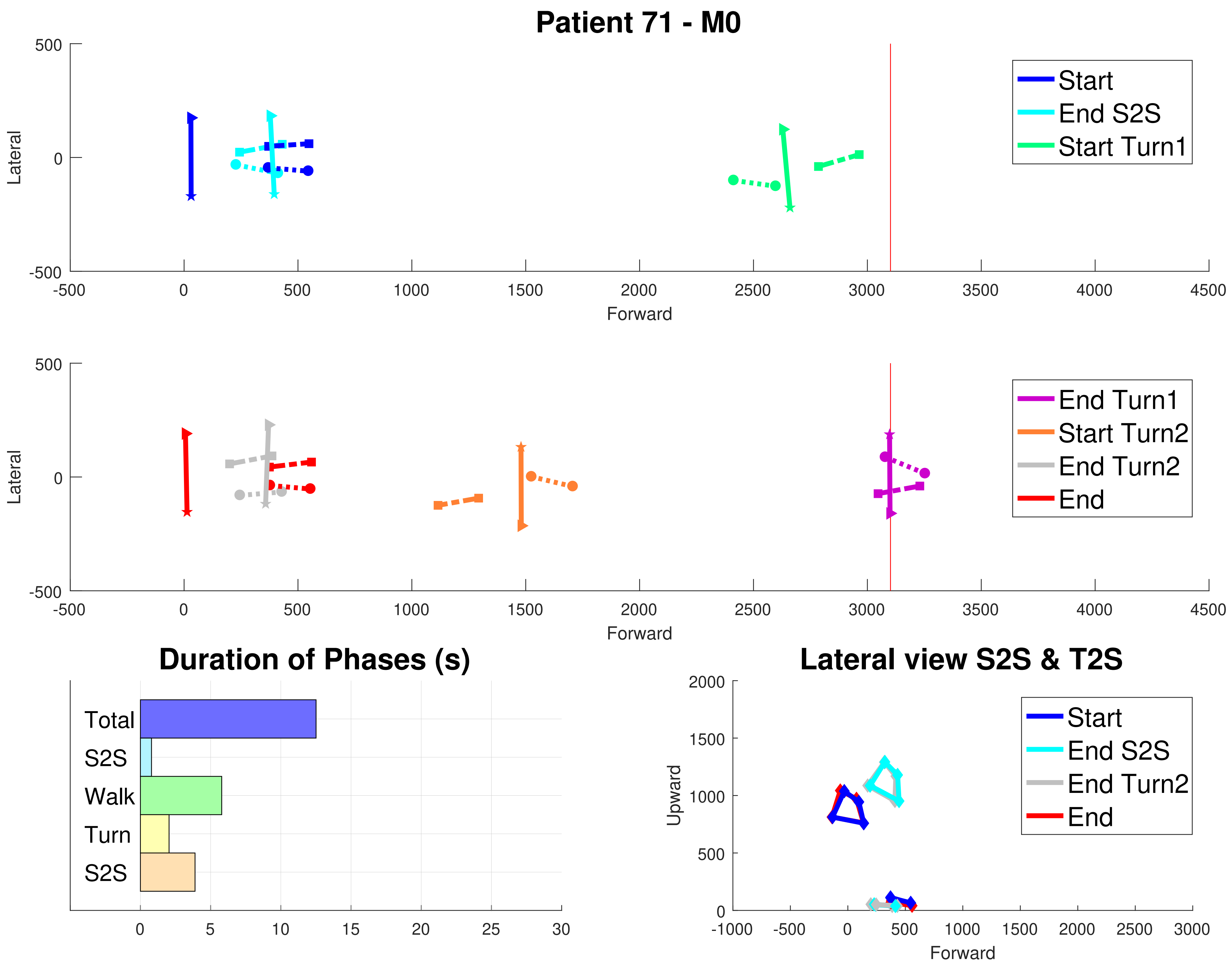
Patient 71 - M0
Lateral
Forward
500
0
-500
-500
0
500
1000
1500
2000
2500
3000
3500
4000
4500
Start
End S2S
Start Turn1
End Turn1
Start Turn2
End Turn2
End
Duration of Phases (s)
Total
S2S
Walk
Turn
S2S
0
5
10
15
20
25
30
Lateral view S2S & T2S
Upward
2000
1500
1000
500
-1000
-500
Start
End S2S
End Turn2
End

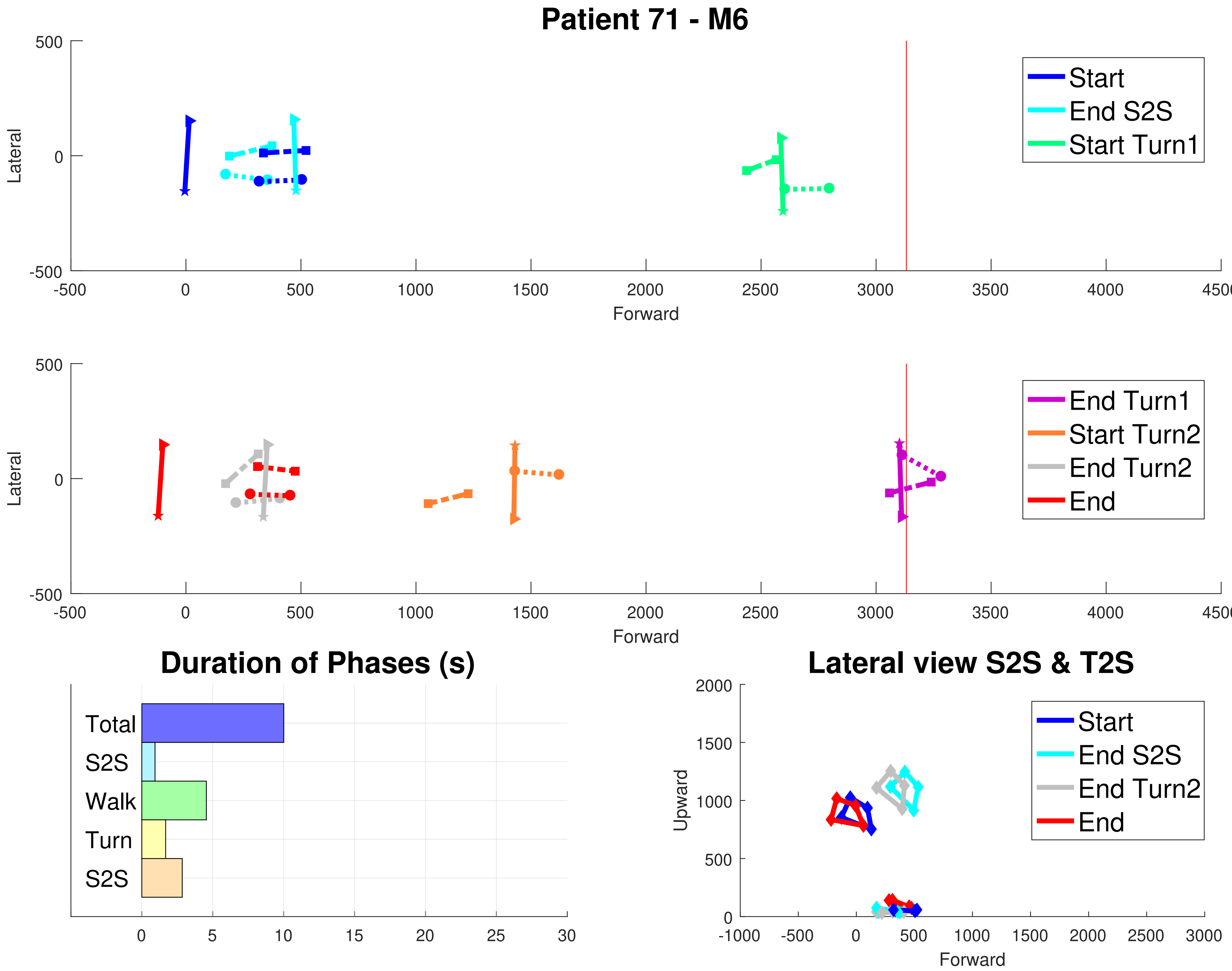

Patient 71 - M6
Lateral
Forward
Start
End S2S
Start Turn1
End Turn1
Start Turn2
End Turn2
End
500
0
-500
-500
0
500
1000
1500
2000
2500
3000
3500
4000
4500
Duration of Phases (s)
Total
S2S
Walk
Turn
S2S
0
5
10
15
20
25
30
Lateral view S2S & T2S
Upward
2000
1500
1000
500
0
-1000
-500
0
500
1000
1500
2000
2500
3000
Forward
Start
End S2S
End Turn2
End
