# Supplementary material for: Which functional tasks present the largest deficits for patients with total hip arthroplasty before and six months after surgery? A study of the timed up-and-go test phases
Source: PLoS One. 2021 Sep 10;16(9):e0255037. doi: 10.1371/journal.pone.0255037 (PMC8432811; doi:10.1371/journal.pone.0255037)
Supplement: S2 Fig — (PDF) [file pone.0255037.s002.pdf]

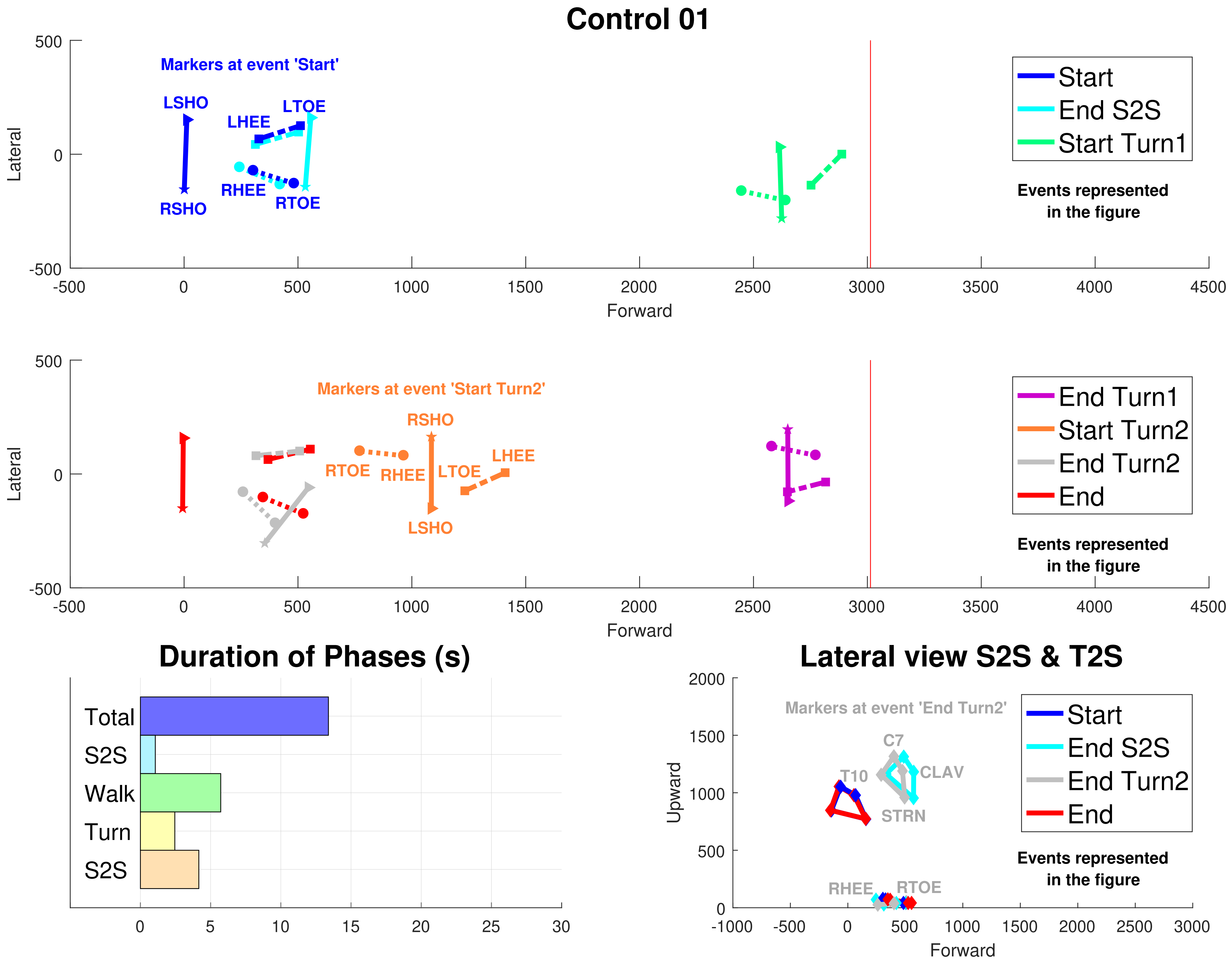
Control 01
Markers at event 'Start'
LSHO
LHEE
LTOE
RHEE
RTOE
RSHO
Start
End S2S
Start Turn1
Events represented in the figure
Lateral
Forward
500
0
-500
-500
0
500
1000
1500
2000
2500
3000
3500
4000
4500
Markers at event 'Start Turn2'
RSHO
LHEE
RTOE
RHEE
LTOE
LSHO
End Turn1
Start Turn2
End Turn2
End
Events represented in the figure
Lateral
Forward
Duration of Phases (s)
Total
S2S
Walk
Turn
S2S
0
5
10
15
20
25
30
Lateral view S2S & T2S
Markers at event 'End Turn2'
C7
T10
CLAV
STRN
RHEE
RTOE
Start
End S2S
End Turn2
End
Events represented in the figure
Upward
Forward
2000
1500
1000
500
0
-1000
-500
0
500
1000
1500
2000
2500
3000

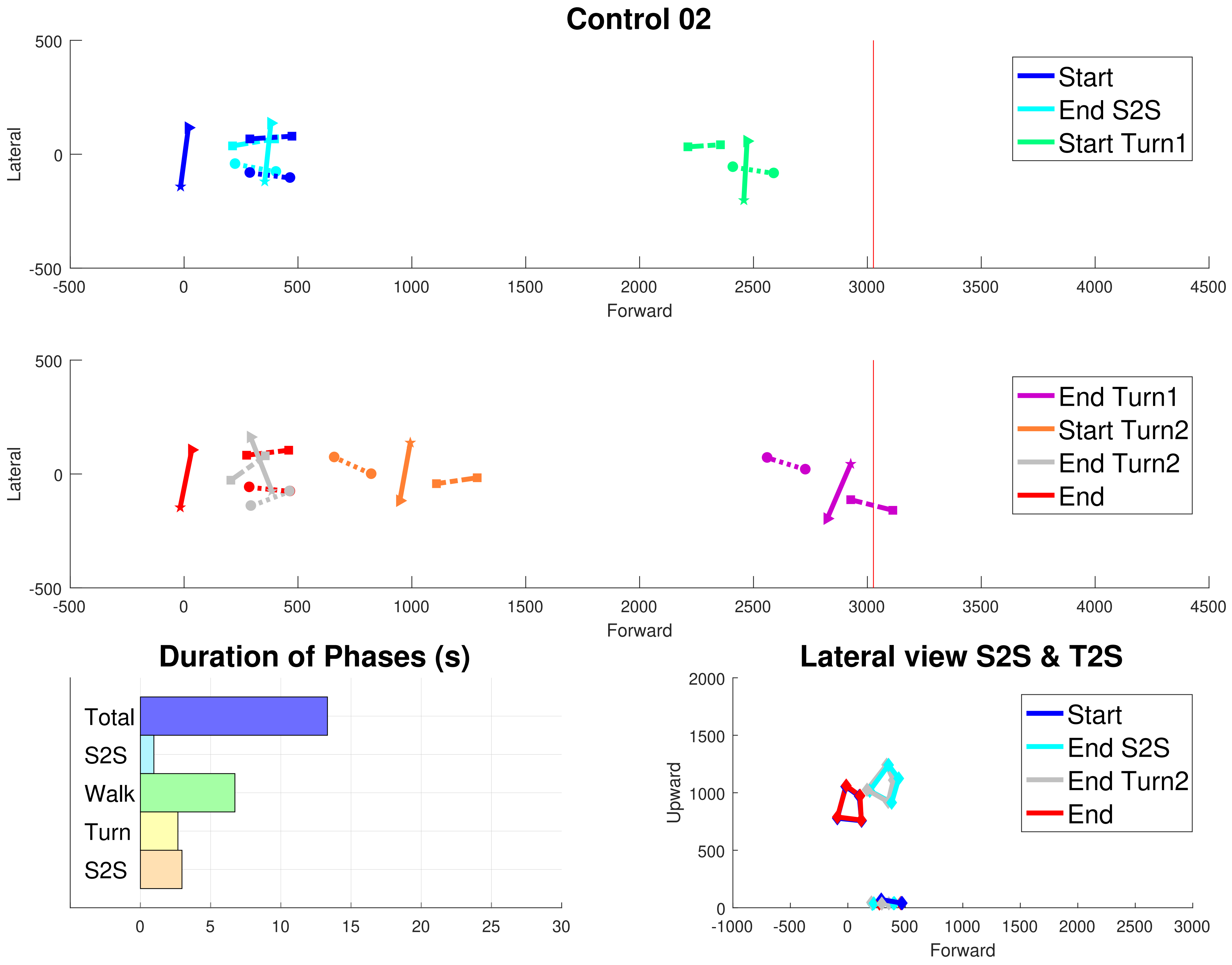

Control 02
Start
End S2S
Start Turn1
End Turn1
Start Turn2
End Turn2
End
Lateral
Forward
Duration of Phases (s)
Total
S2S
Walk
Turn
S2S
Lateral view S2S & T2S
Upward

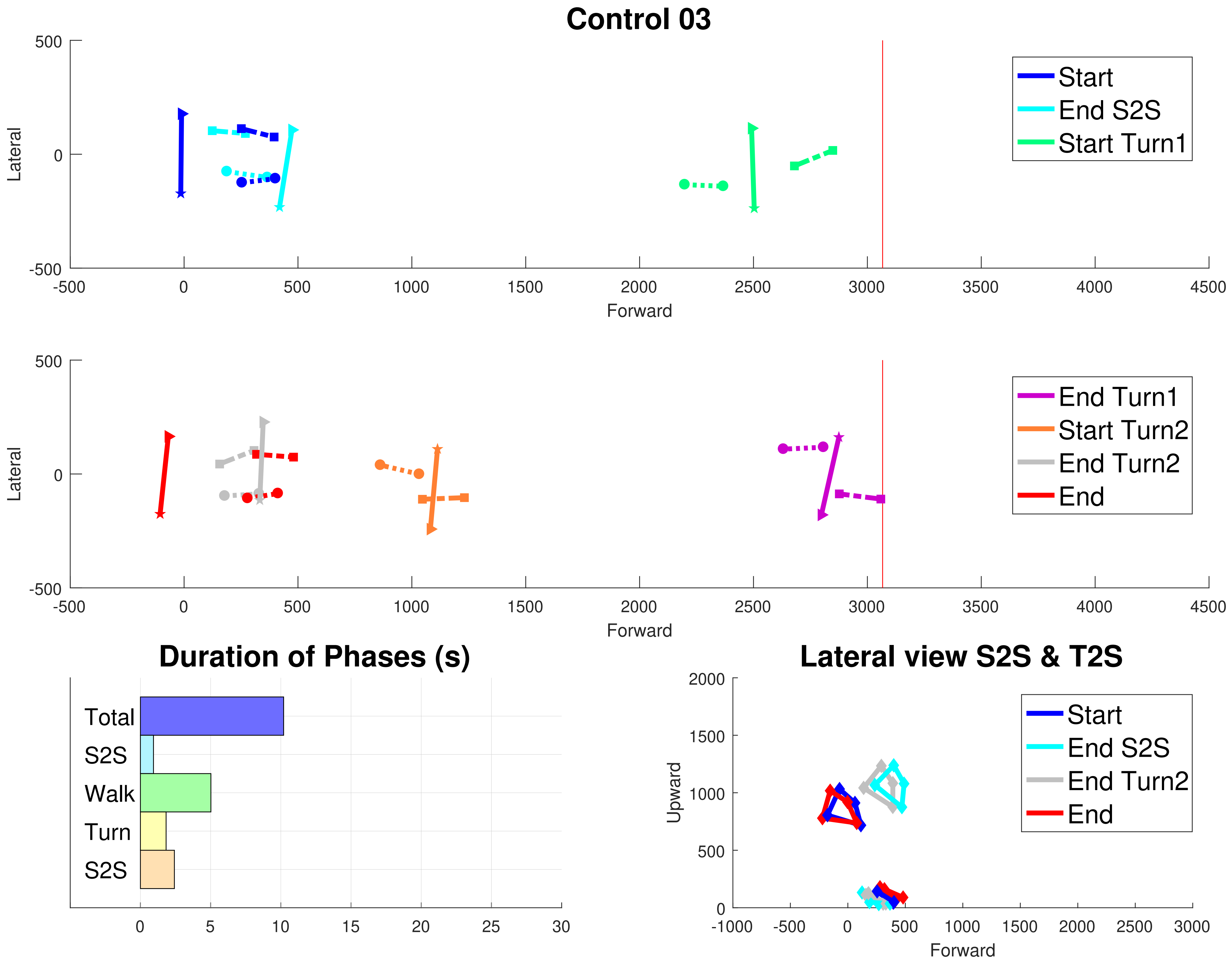

Control 03
Start
End S2S
Start Turn1
Lateral
Forward
500
0
-500
-500
0
500
1000
1500
2000
2500
3000
3500
4000
4500
End Turn1
Start Turn2
End Turn2
End
Lateral
Forward
Duration of Phases (s)
Total
S2S
Walk
Turn
S2S
0
5
10
15
20
25
30
Lateral view S2S & T2S
Start
End S2S
End Turn2
End
Upward
Forward
2000
1500
1000
500
0
-1000
-500
0
500
1000
1500
2000
2500
3000

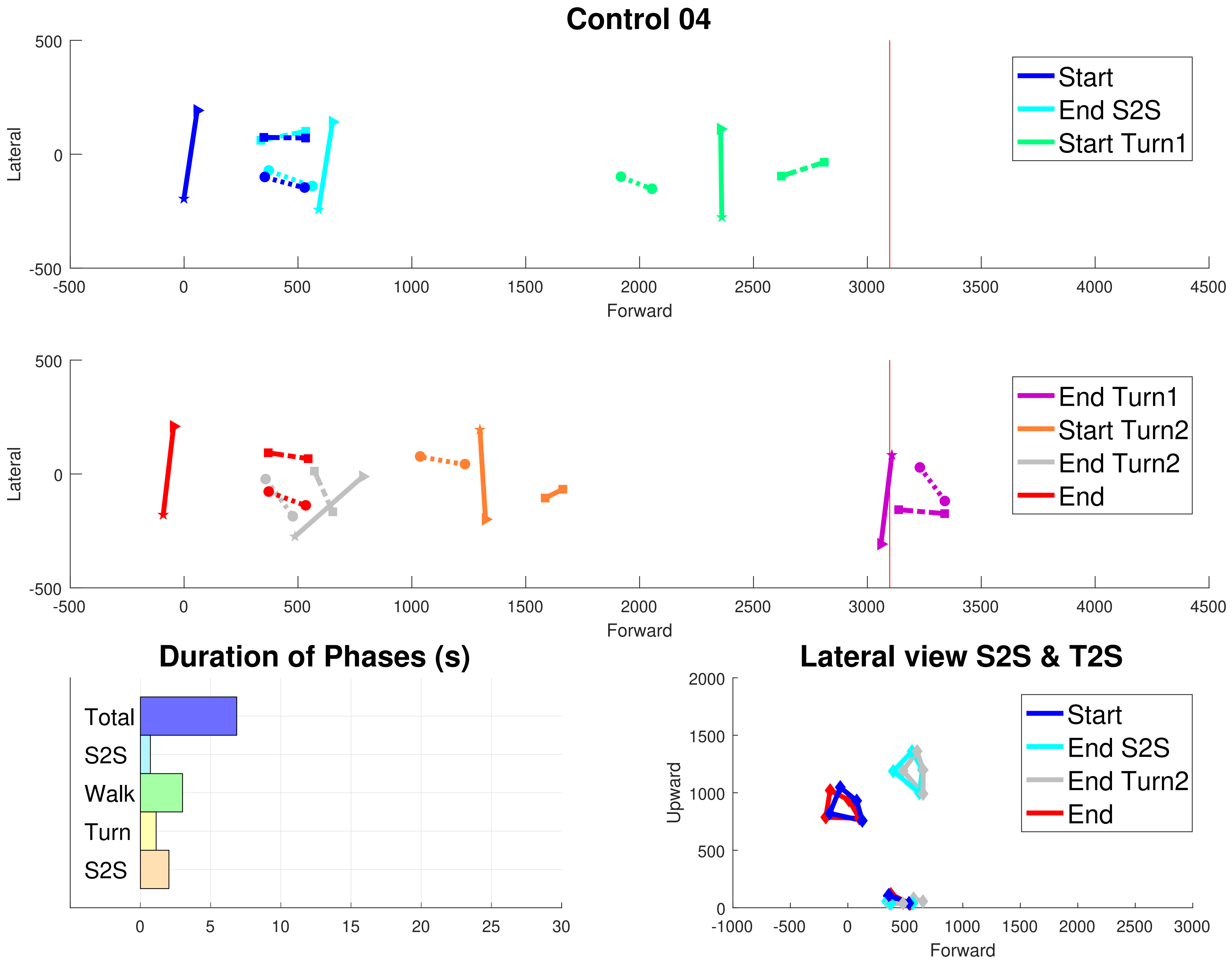

Control 04
Start
End S2S
Start Turn1
End Turn1
Start Turn2
End Turn2
End
Lateral
Forward
Duration of Phases (s)
Total
S2S
Walk
Turn
S2S
Lateral view S2S & T2S
Start
End S2S
End Turn2
End
Upward
Forward

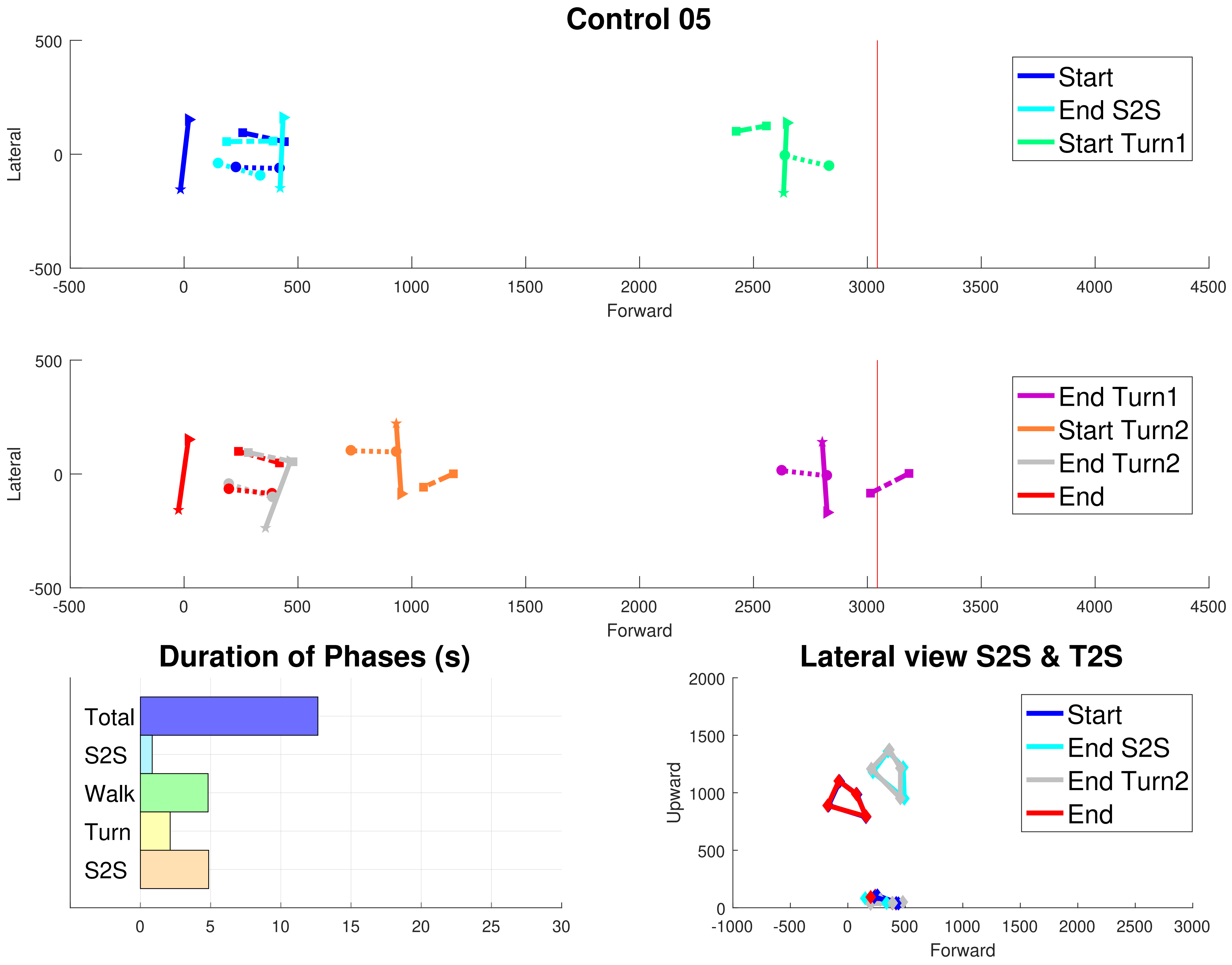
Control 05
Lateral
Forward
500
0
-500
-500
0
500
1000
1500
2000
2500
3000
3500
4000
4500
Start
End S2S
Start Turn1
End Turn1
Start Turn2
End Turn2
End
Duration of Phases (s)
Total
S2S
Walk
Turn
S2S
0
5
10
15
20
25
30
Lateral view S2S & T2S
Upward
2000
1500
1000
500
0
-1000
-500
0
500
1000
1500
2000
2500
3000
Start
End S2S
End Turn2
End

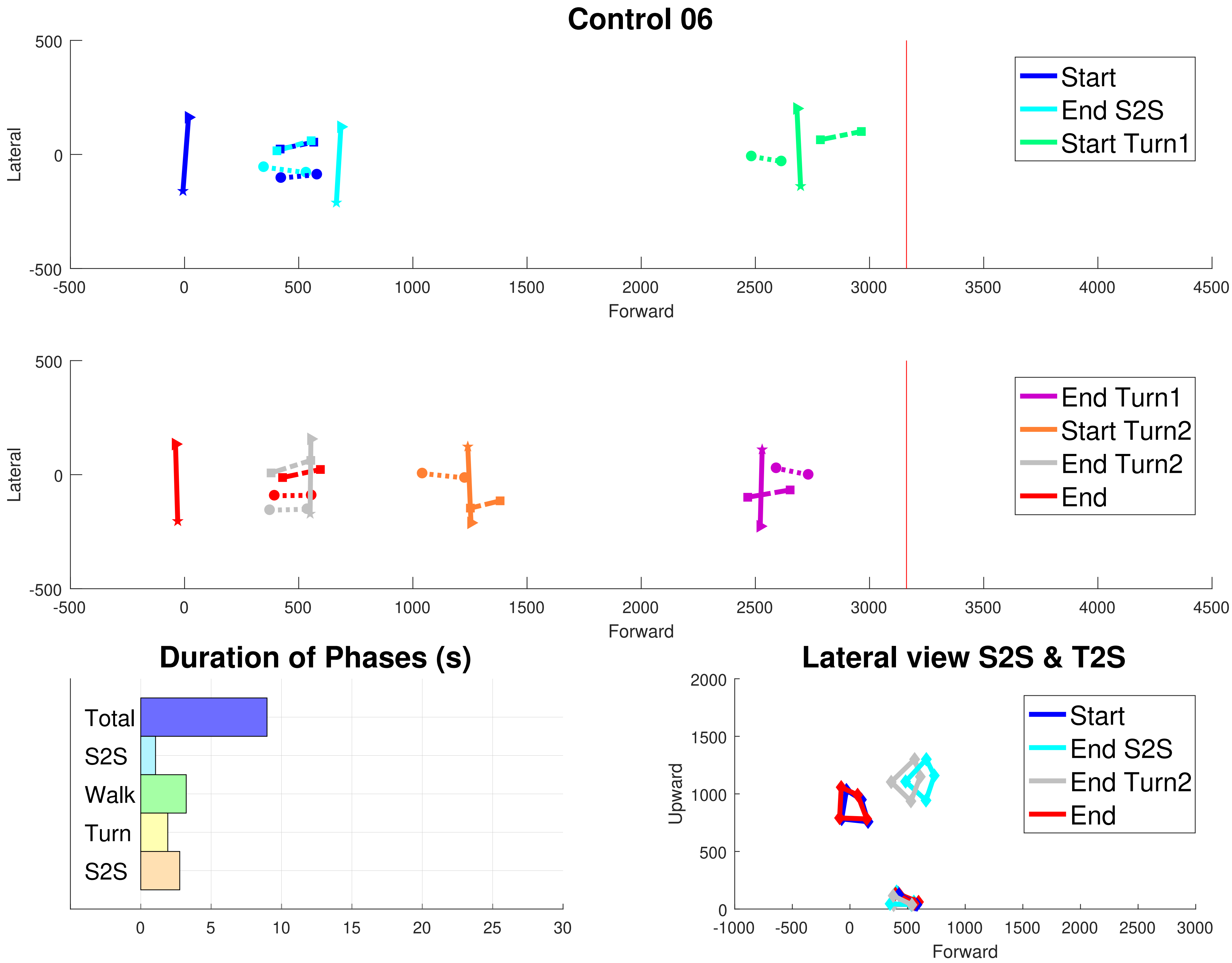
Control 06
Lateral
Forward
Start
End S2S
Start Turn1
End Turn1
Start Turn2
End Turn2
End
500
0
-500
-500
0
500
1000
1500
2000
2500
3000
3500
4000
4500
Duration of Phases (s)
Total
S2S
Walk
Turn
S2S
0
5
10
15
20
25
30
Lateral view S2S & T2S
Upward
Forward
Start
End S2S
End Turn2
End
2000
1500
1000
500
0
-1000
-500
0
500
1000
1500
2000
2500
3000

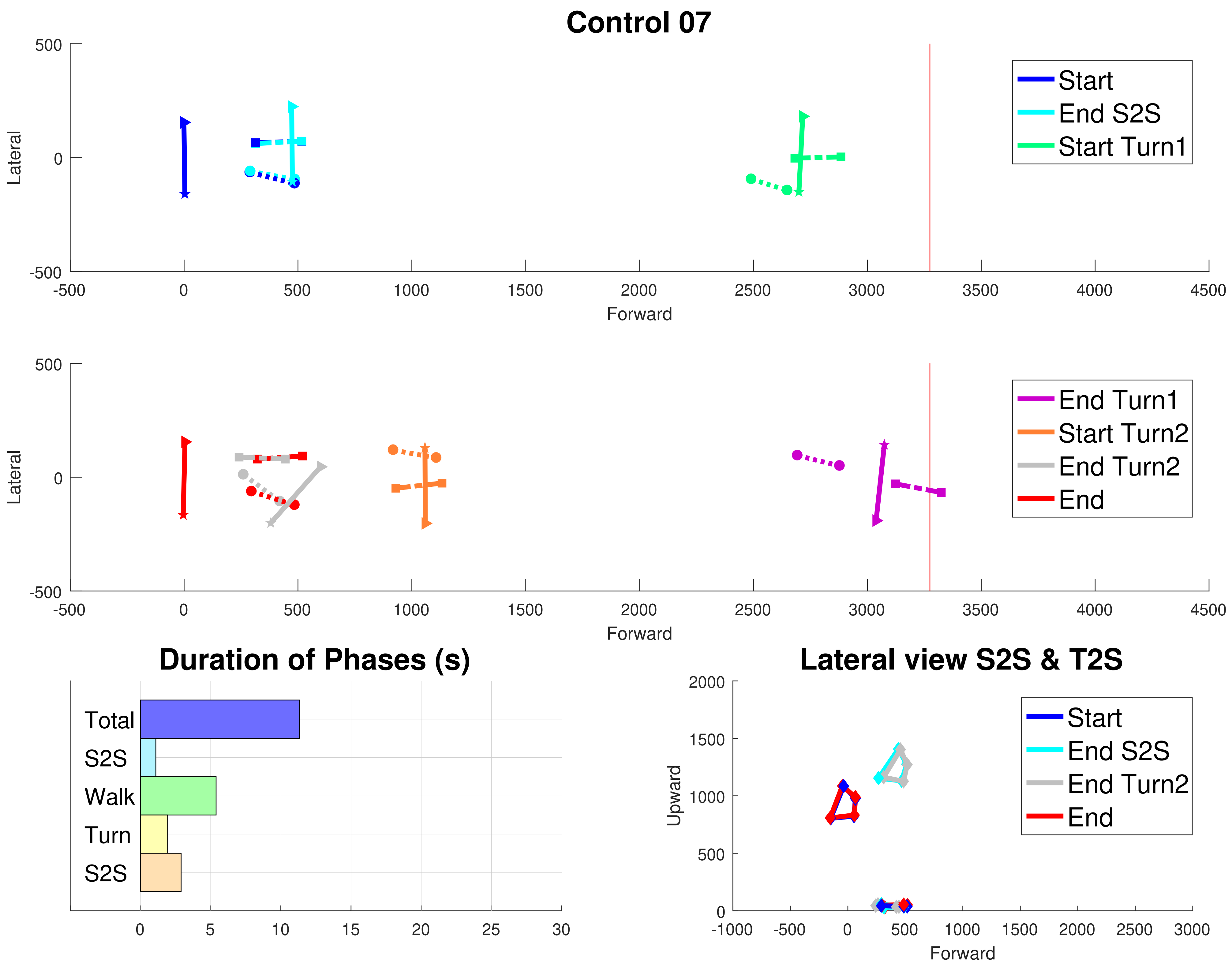
Control 07
Lateral
Forward
Start
End S2S
Start Turn1
End Turn1
Start Turn2
End Turn2
End
Duration of Phases (s)
Total
S2S
Walk
Turn
S2S
Lateral view S2S & T2S
Upward
Forward
Start
End S2S
End Turn2
End

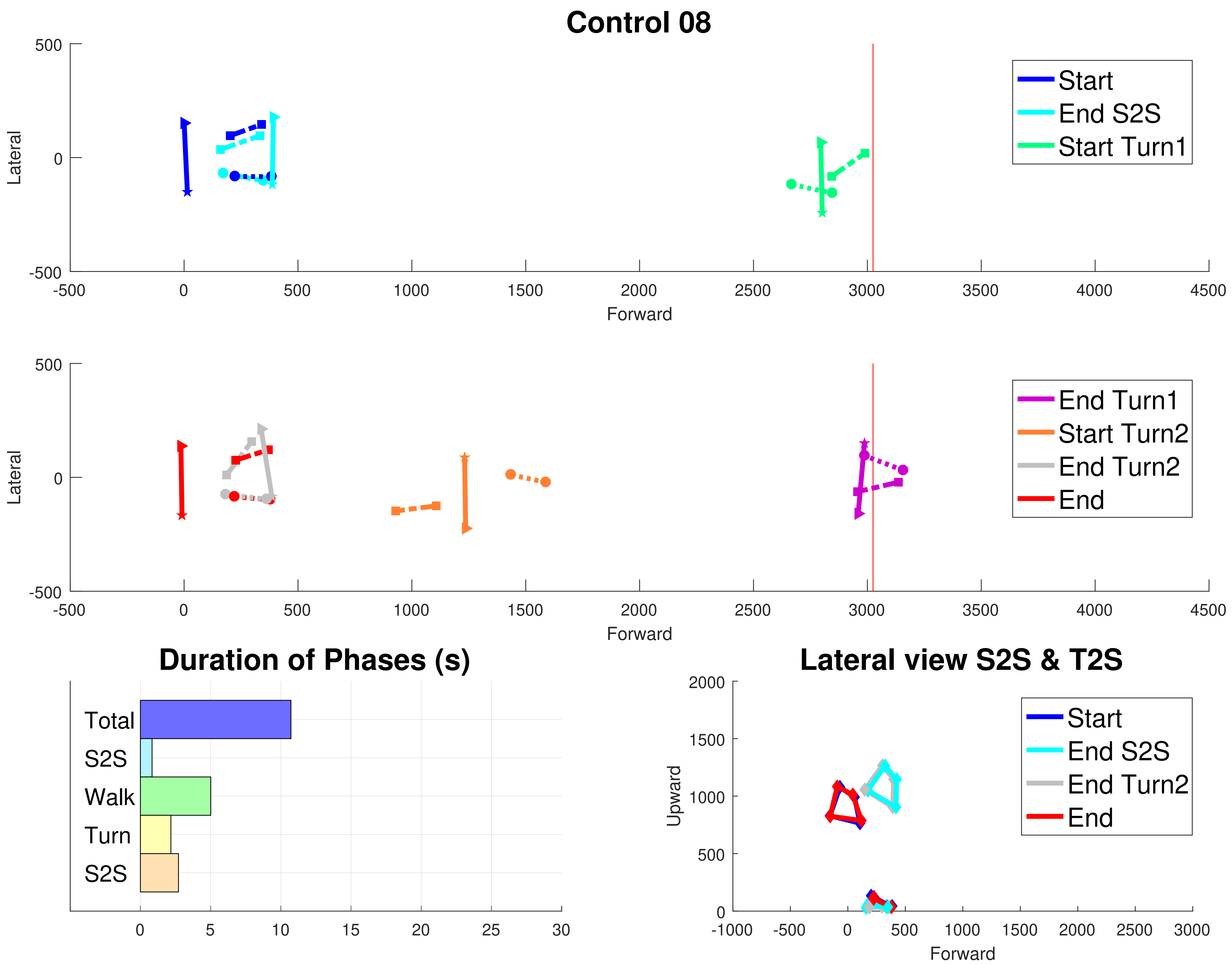

Control 08
Lateral
Forward
Start
End S2S
Start Turn1
End Turn1
Start Turn2
End Turn2
End
Duration of Phases (s)
Total
S2S
Walk
Turn
S2S
Lateral view S2S & T2S
Upward

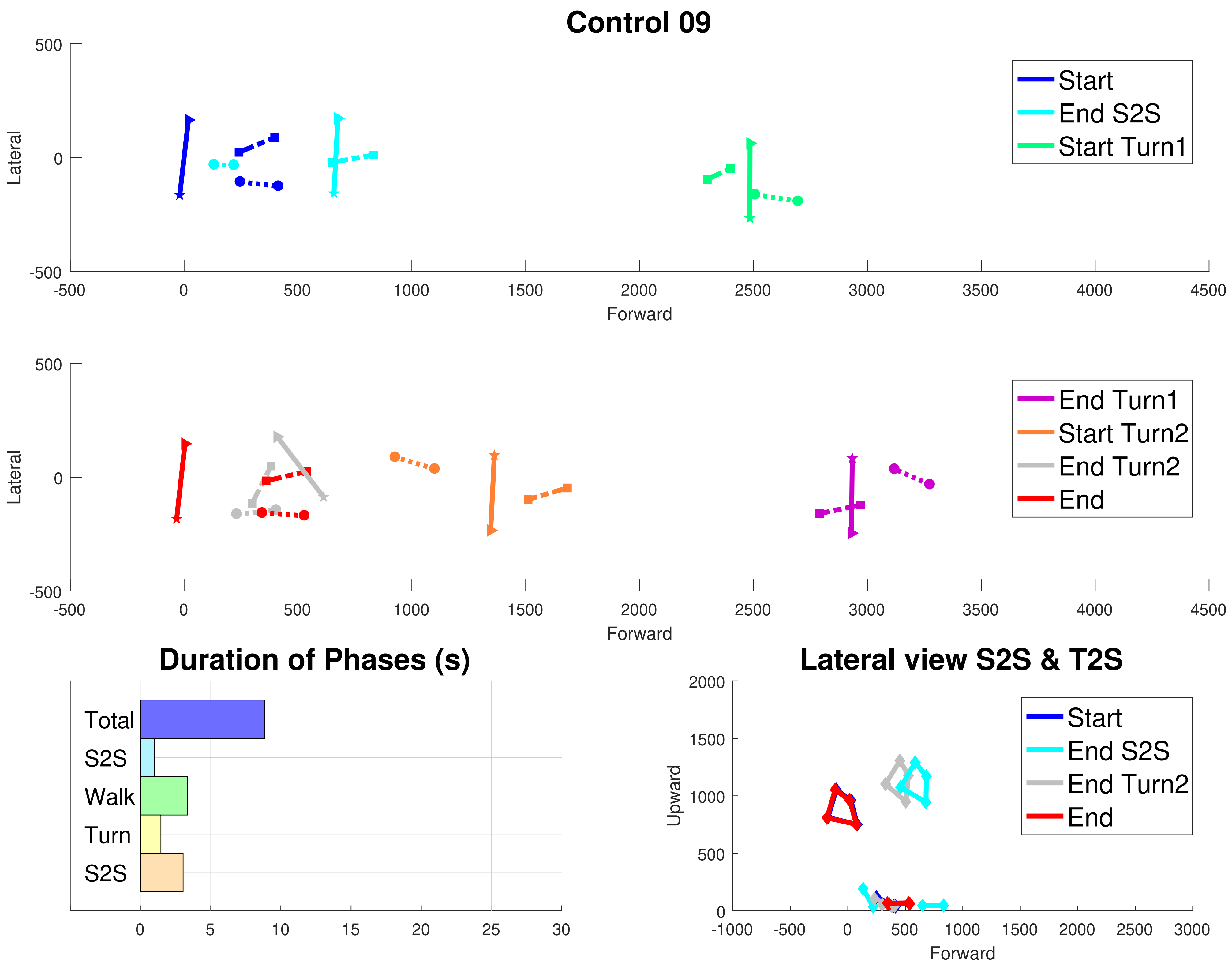
Control 09
Lateral
Forward
Start
End S2S
Start Turn1
End Turn1
Start Turn2
End Turn2
End
Duration of Phases (s)
Total
S2S
Walk
Turn
S2S
Lateral view S2S & T2S
Upward
Forward
Start
End S2S
End Turn2
End

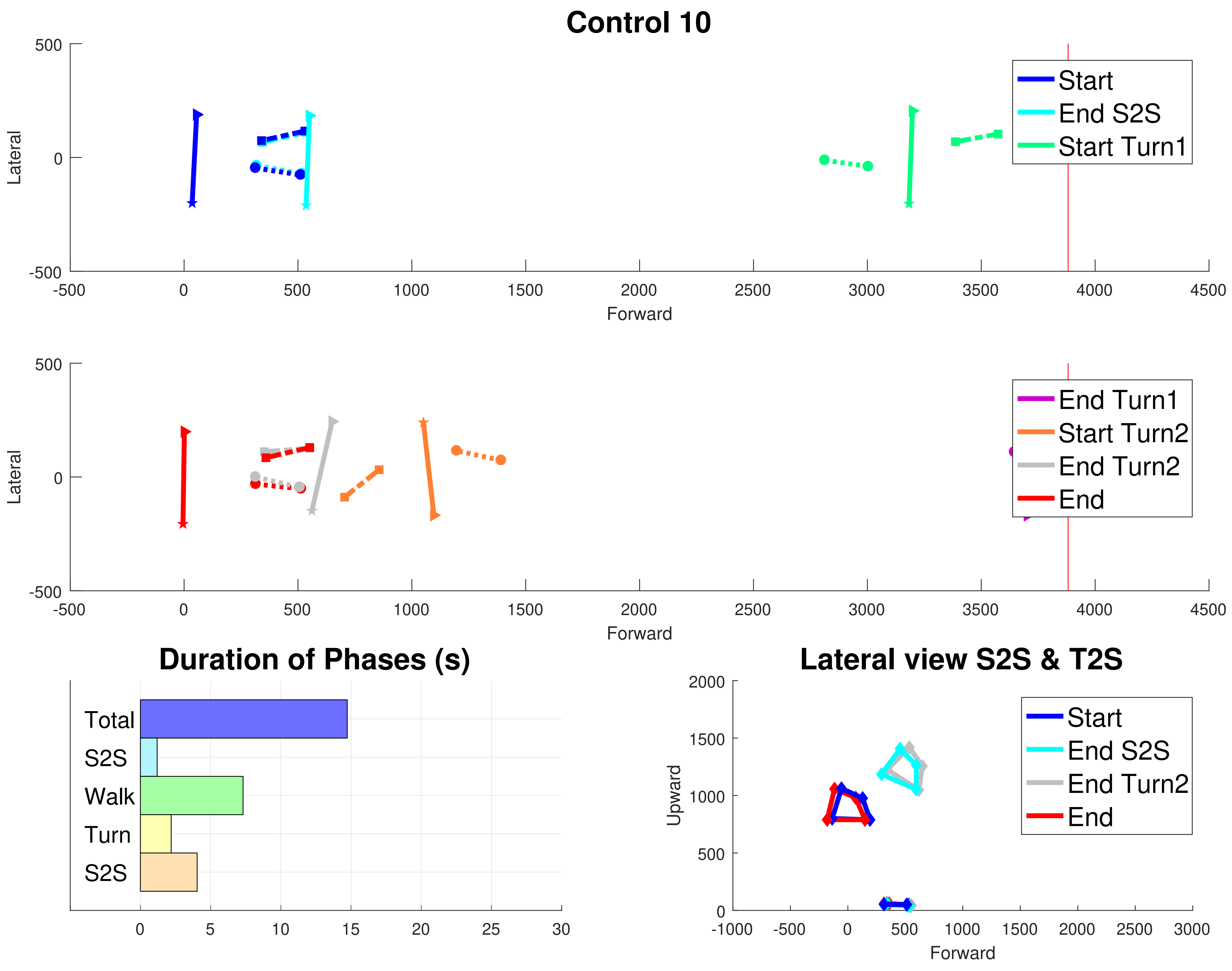
Control 10
Start
End S2S
Start Turn1
Lateral
Forward
End Turn1
Start Turn2
End Turn2
End
Duration of Phases (s)
Total
S2S
Walk
Turn
S2S
Lateral view S2S & T2S
Upward
Start
End S2S
End Turn2
End

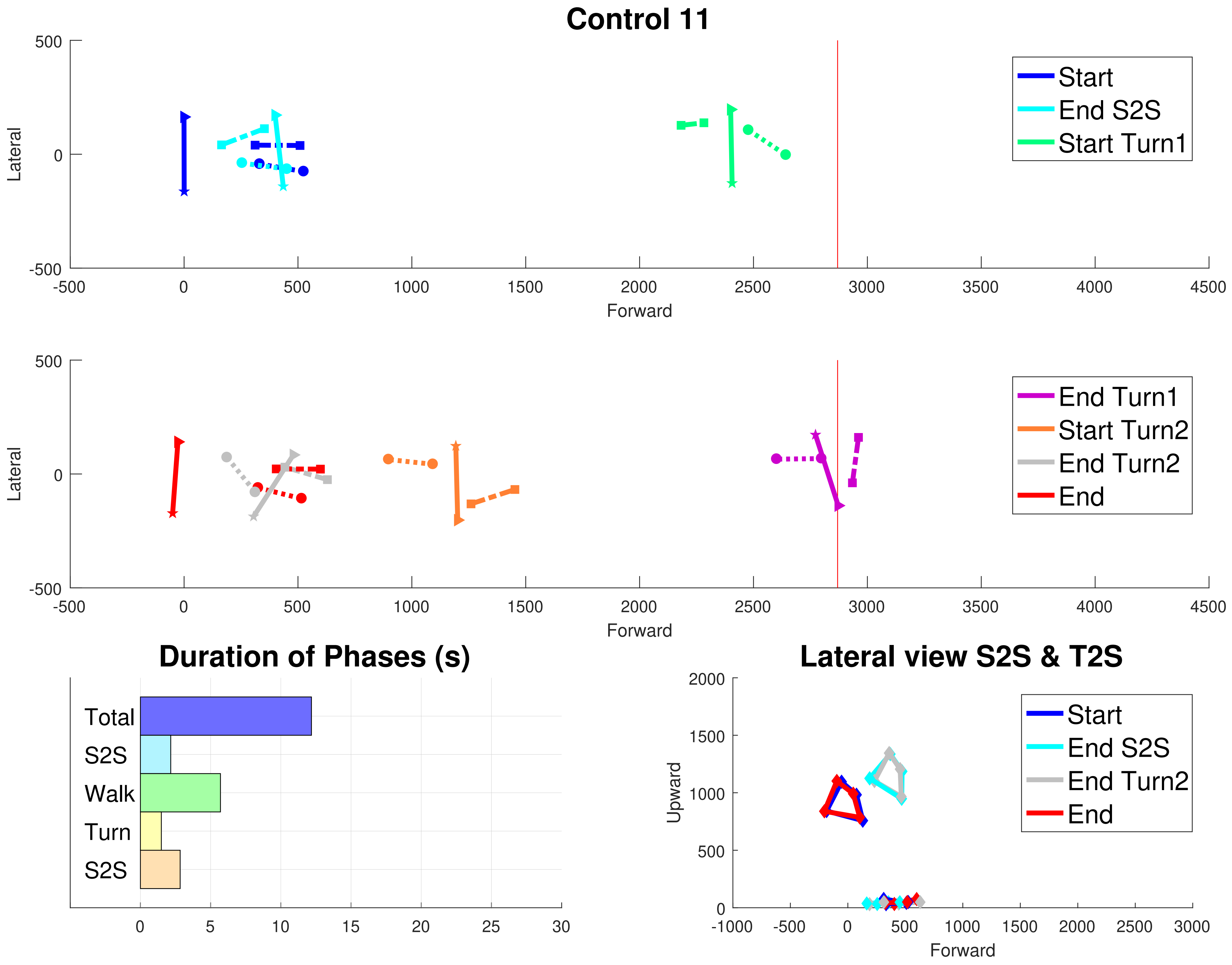
Control 11
Lateral
Forward
Start
End S2S
Start Turn1
End Turn1
Start Turn2
End Turn2
End
Duration of Phases (s)
Total
S2S
Walk
Turn
S2S
Lateral view S2S & T2S
Upward
Forward
Start
End S2S
End Turn2
End

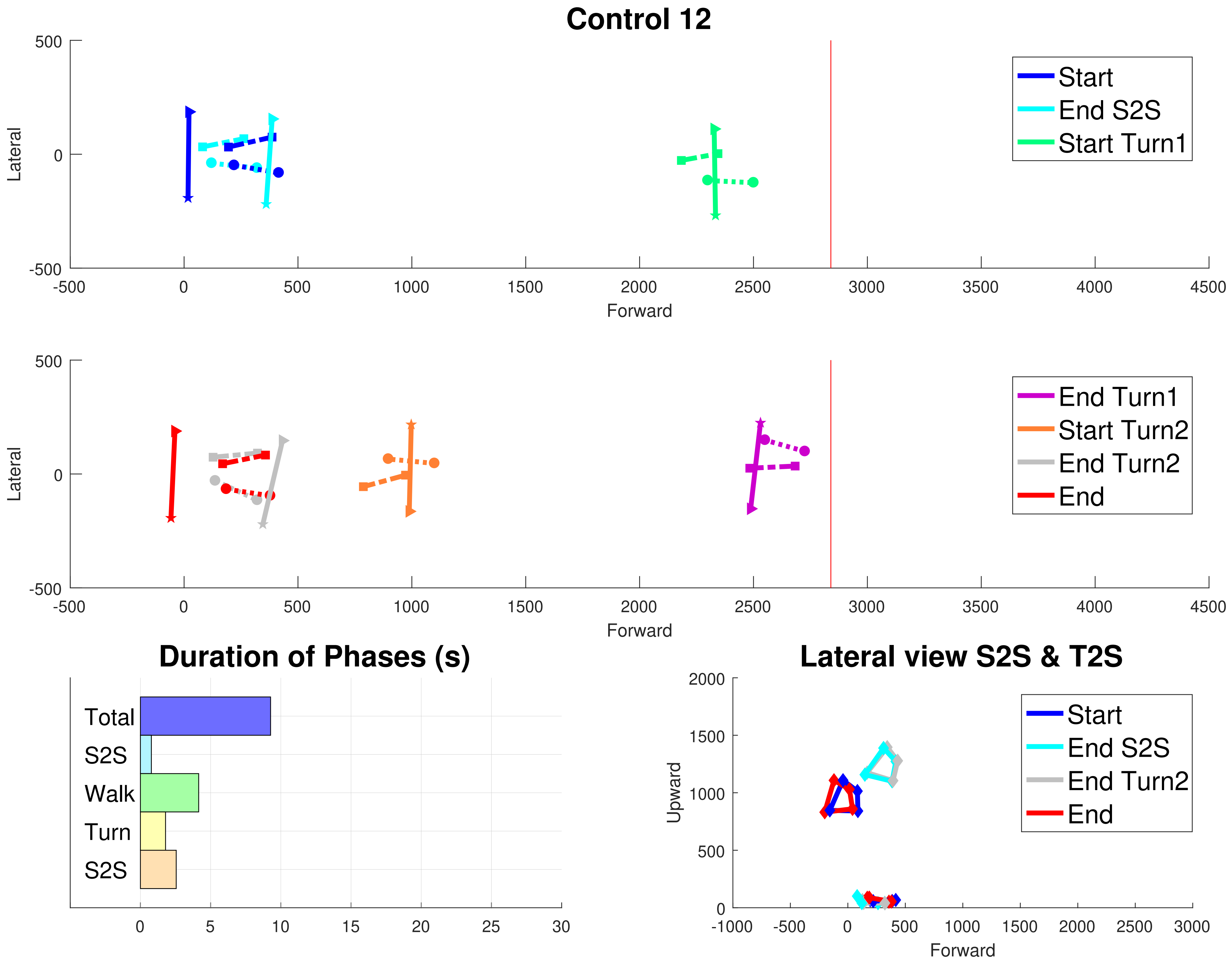
Control 12
Lateral
Forward
Start
End S2S
Start Turn1
End Turn1
Start Turn2
End Turn2
End
Duration of Phases (s)
Total
S2S
Walk
Turn
S2S
Lateral view S2S & T2S
Upward
Forward
Start
End S2S
End Turn2
End

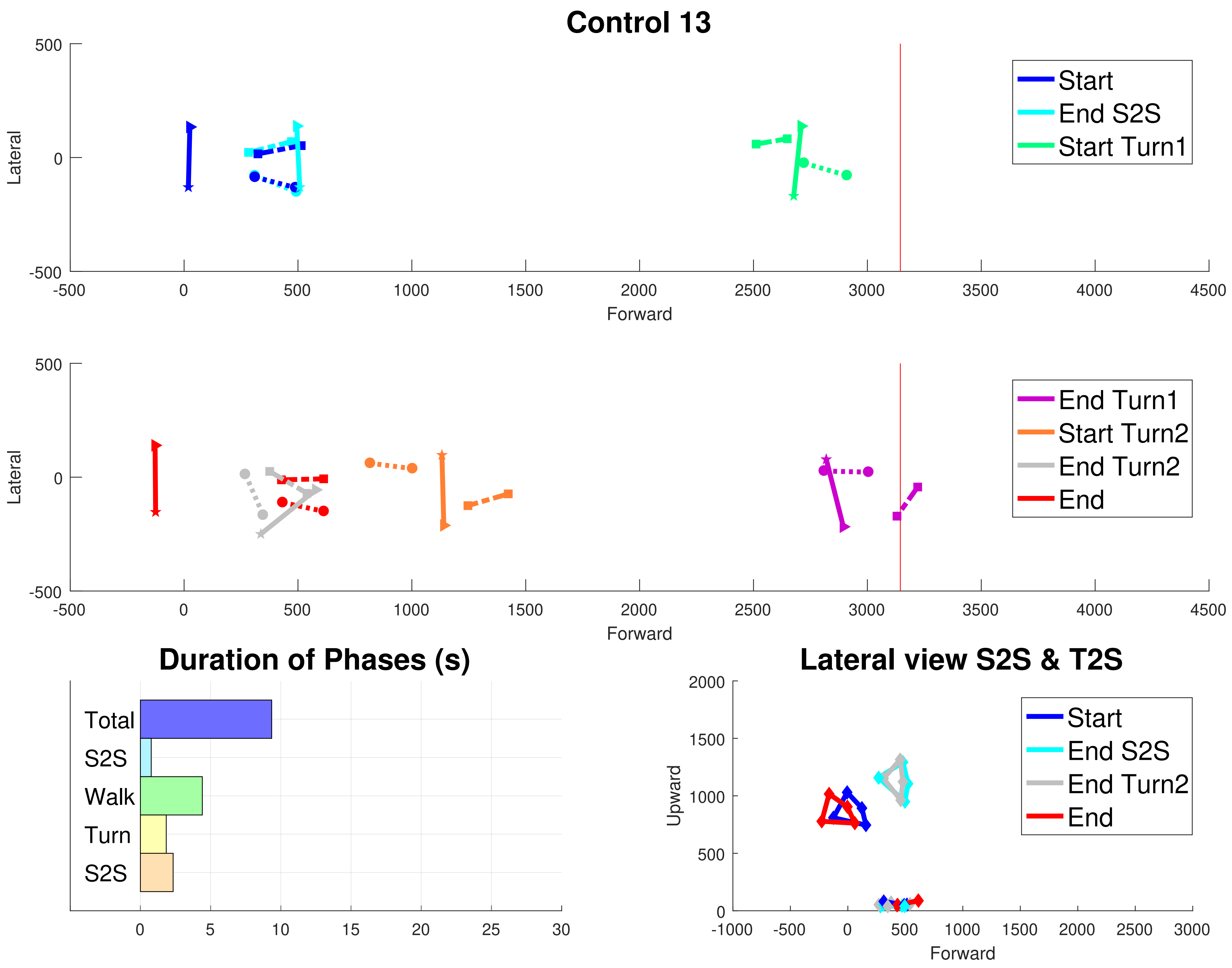

Control 13
Lateral
Forward
Start
End S2S
Start Turn1
End Turn1
Start Turn2
End Turn2
End
Duration of Phases (s)
Total
S2S
Walk
Turn
S2S
Lateral view S2S & T2S
Upward
Forward
Start
End S2S
End Turn2
End

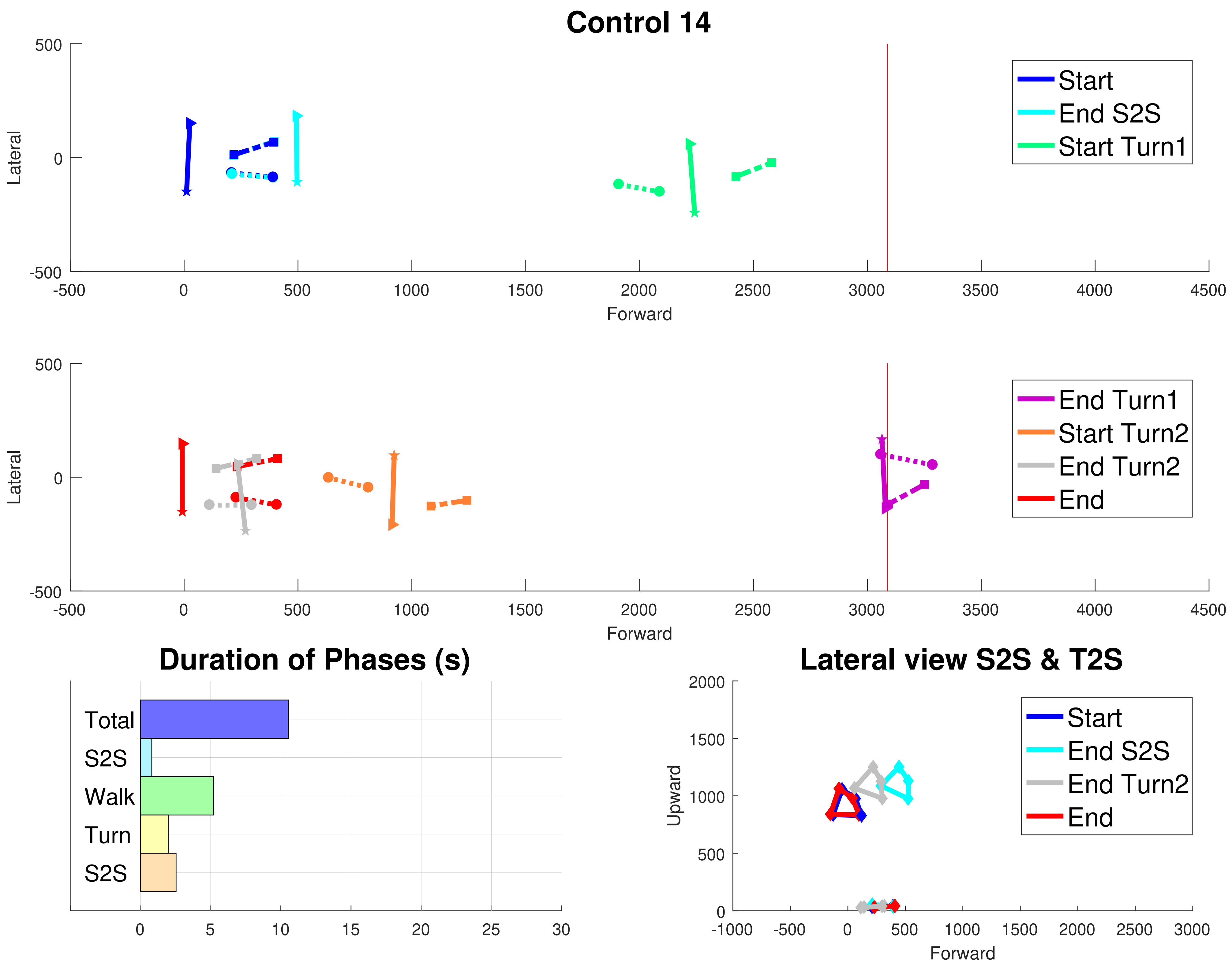
Control 14
Lateral
Forward
Start
End S2S
Start Turn1
End Turn1
Start Turn2
End Turn2
End
Duration of Phases (s)
Total
S2S
Walk
Turn
S2S
Lateral view S2S & T2S
Upward
Start
End S2S
End Turn2
End

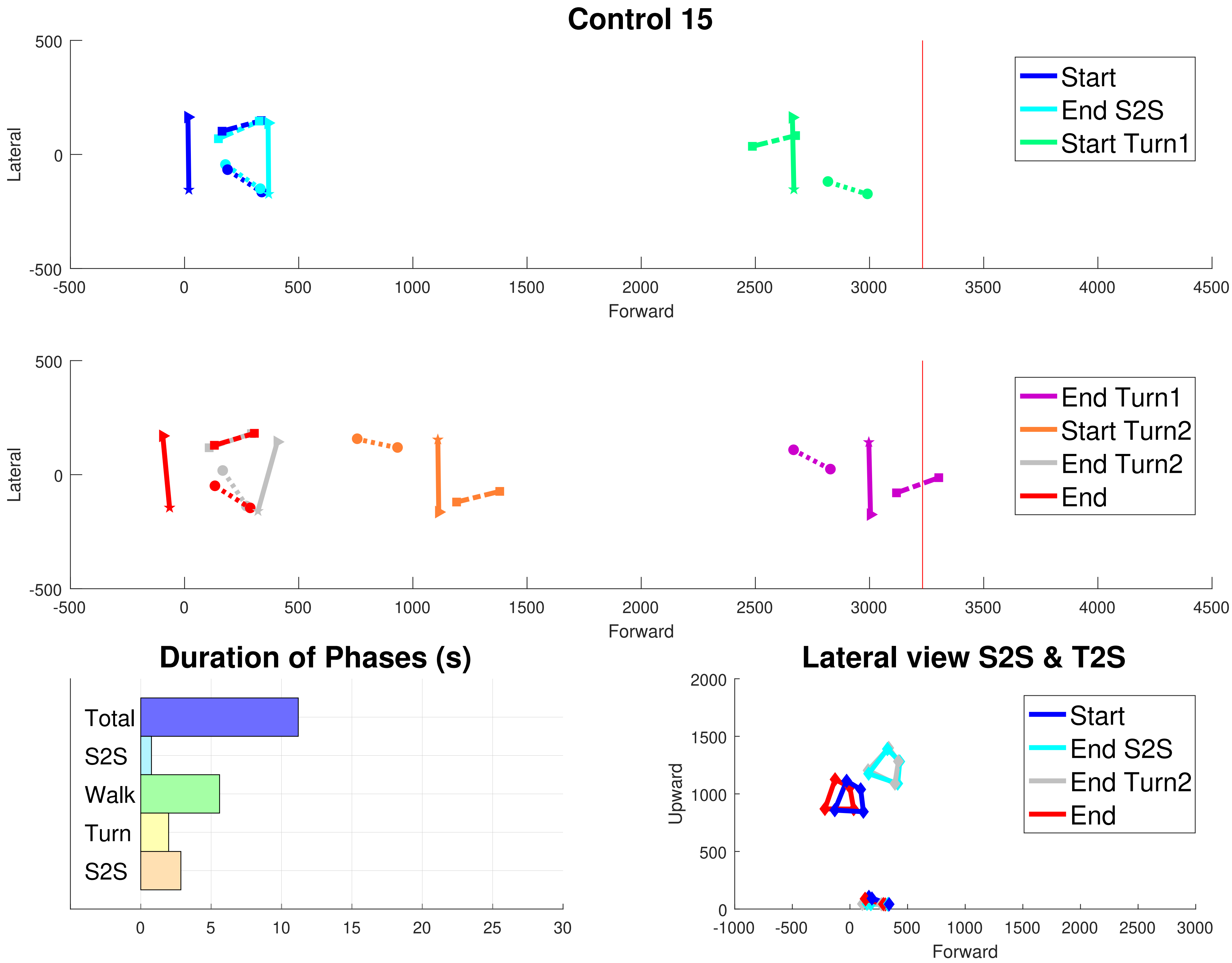

Control 15
Lateral
Forward
Start
End S2S
Start Turn1
End Turn1
Start Turn2
End Turn2
End
Duration of Phases (s)
Total
S2S
Walk
Turn
S2S
Lateral view S2S & T2S
Upward
Forward
Start
End S2S
End Turn2
End

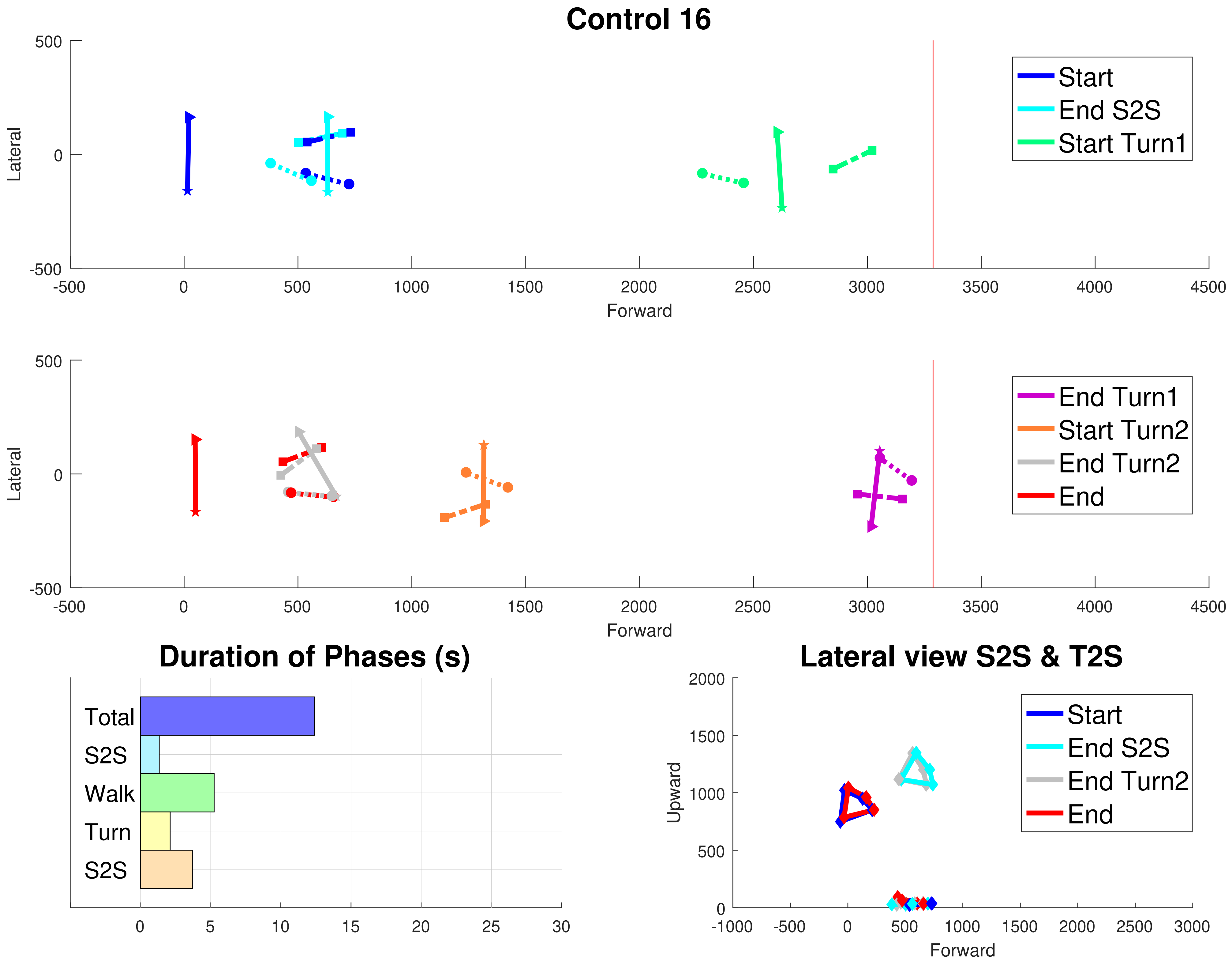
Control 16
Lateral
Forward
Start
End S2S
Start Turn1
End Turn1
Start Turn2
End Turn2
End
Duration of Phases (s)
Total
S2S
Walk
Turn
S2S
Lateral view S2S & T2S
Upward
Forward
Start
End S2S
End Turn2
End

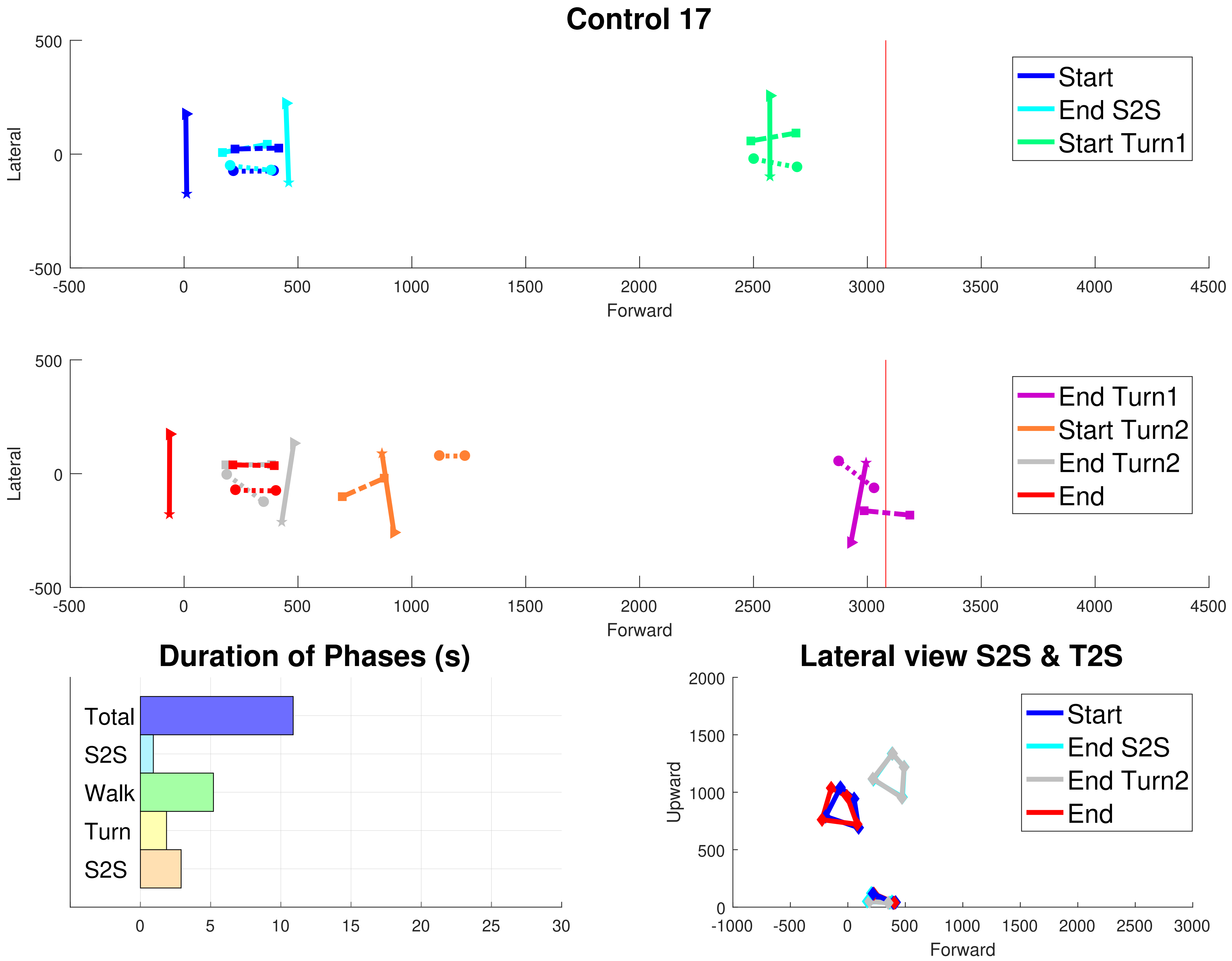
Control 17
Start
End S2S
Start Turn1
Lateral
Forward
End Turn1
Start Turn2
End Turn2
End
Lateral
Forward
Duration of Phases (s)
Total
S2S
Walk
Turn
S2S
Lateral view S2S & T2S
Start
End S2S
End Turn2
End
Upward
Forward

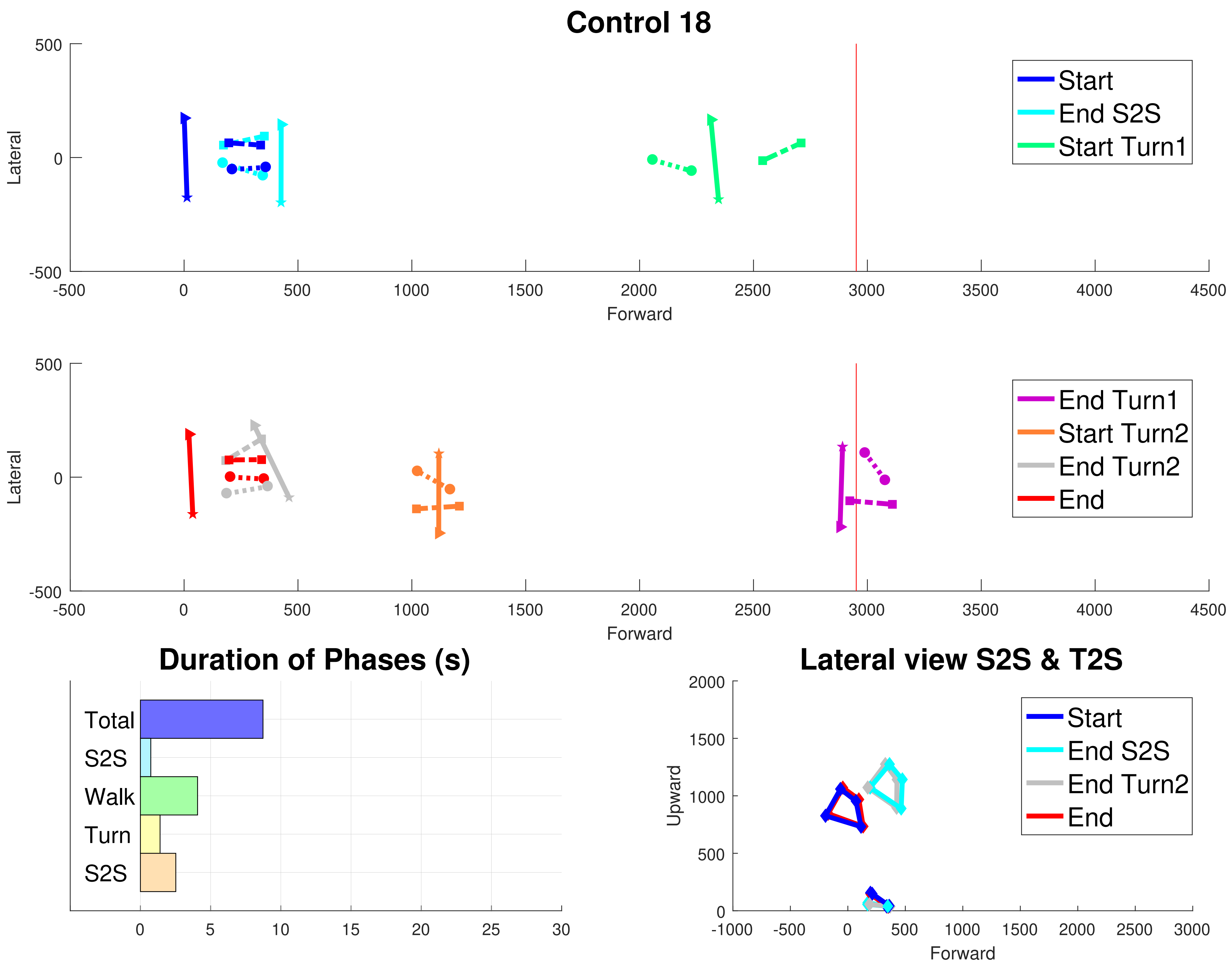

Control 18
Lateral
Forward
Start
End S2S
Start Turn1
End Turn1
Start Turn2
End Turn2
End
Duration of Phases (s)
Total
S2S
Walk
Turn
S2S
Lateral view S2S & T2S
Upward
Forward
Start
End S2S
End Turn2
End

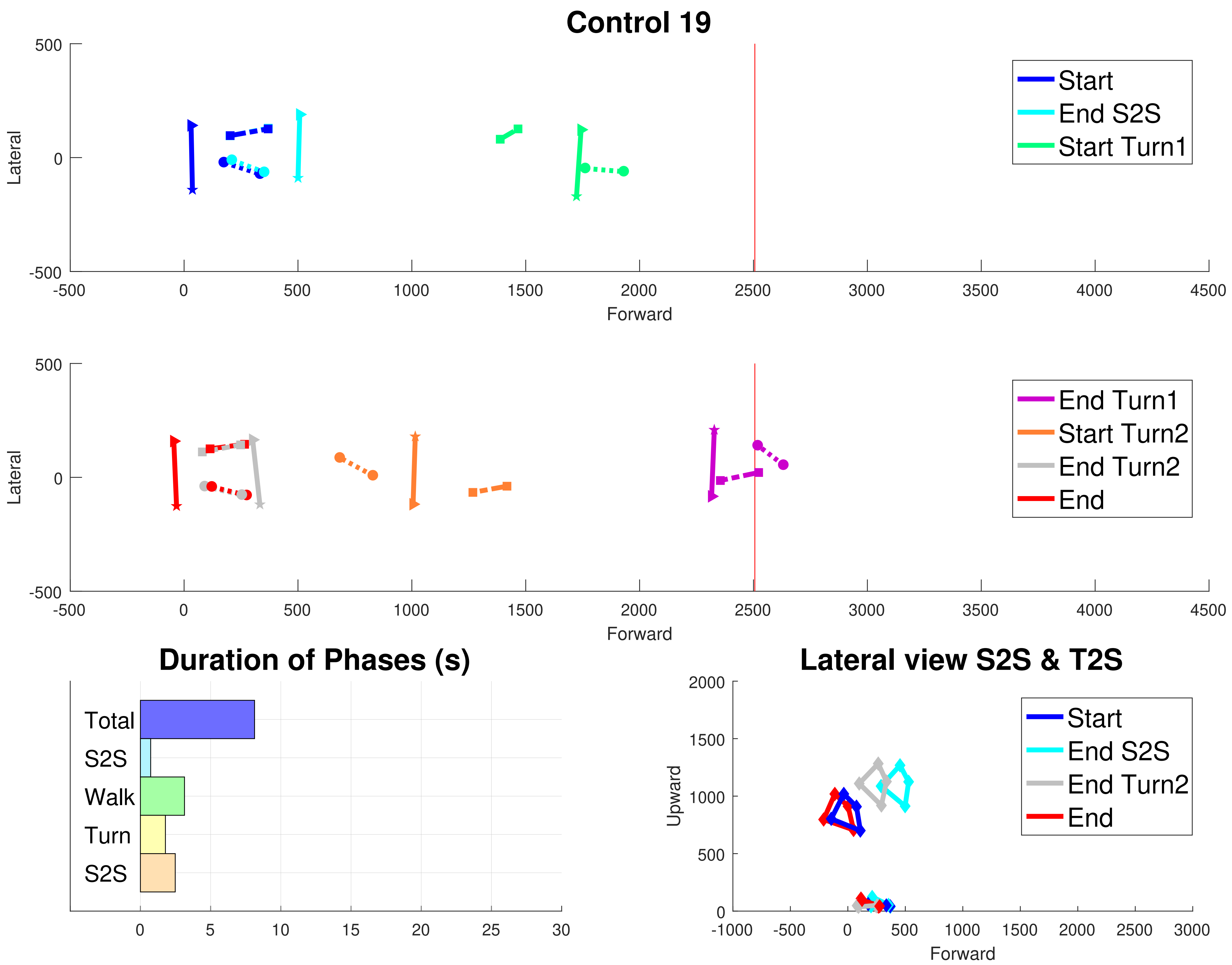

Control 19
Lateral
Forward
Start
End S2S
Start Turn1
End Turn1
Start Turn2
End Turn2
End
Duration of Phases (s)
Total
S2S
Walk
Turn
S2S
Lateral view S2S & T2S
Upward
Forward
Start
End S2S
End Turn2
End

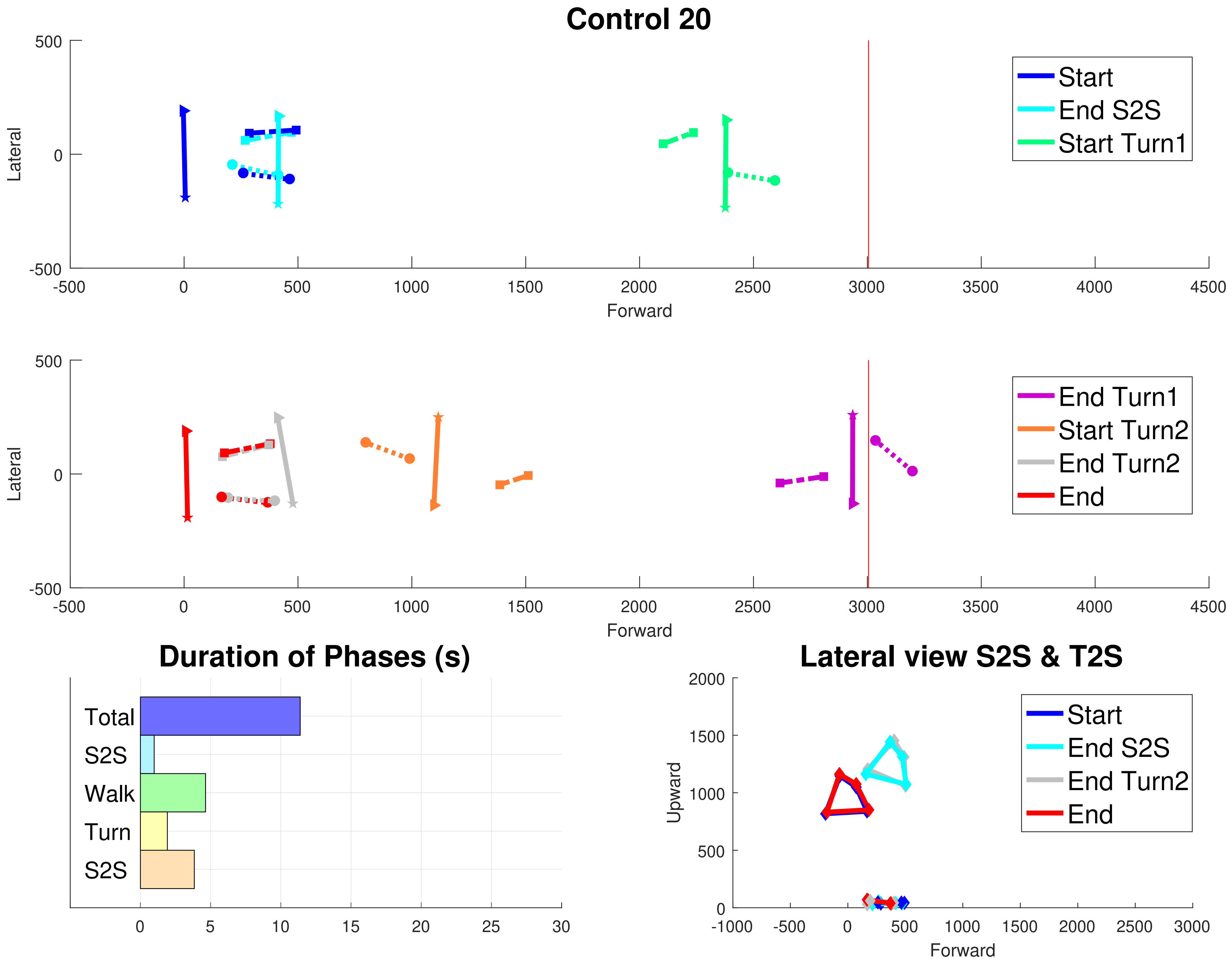
Control 20
Start
End S2S
Start Turn1
End Turn1
Start Turn2
End Turn2
End
Lateral
Forward
Duration of Phases (s)
Total
S2S
Walk
Turn
S2S
Lateral view S2S & T2S
Start
End S2S
End Turn2
End
Upward
Forward

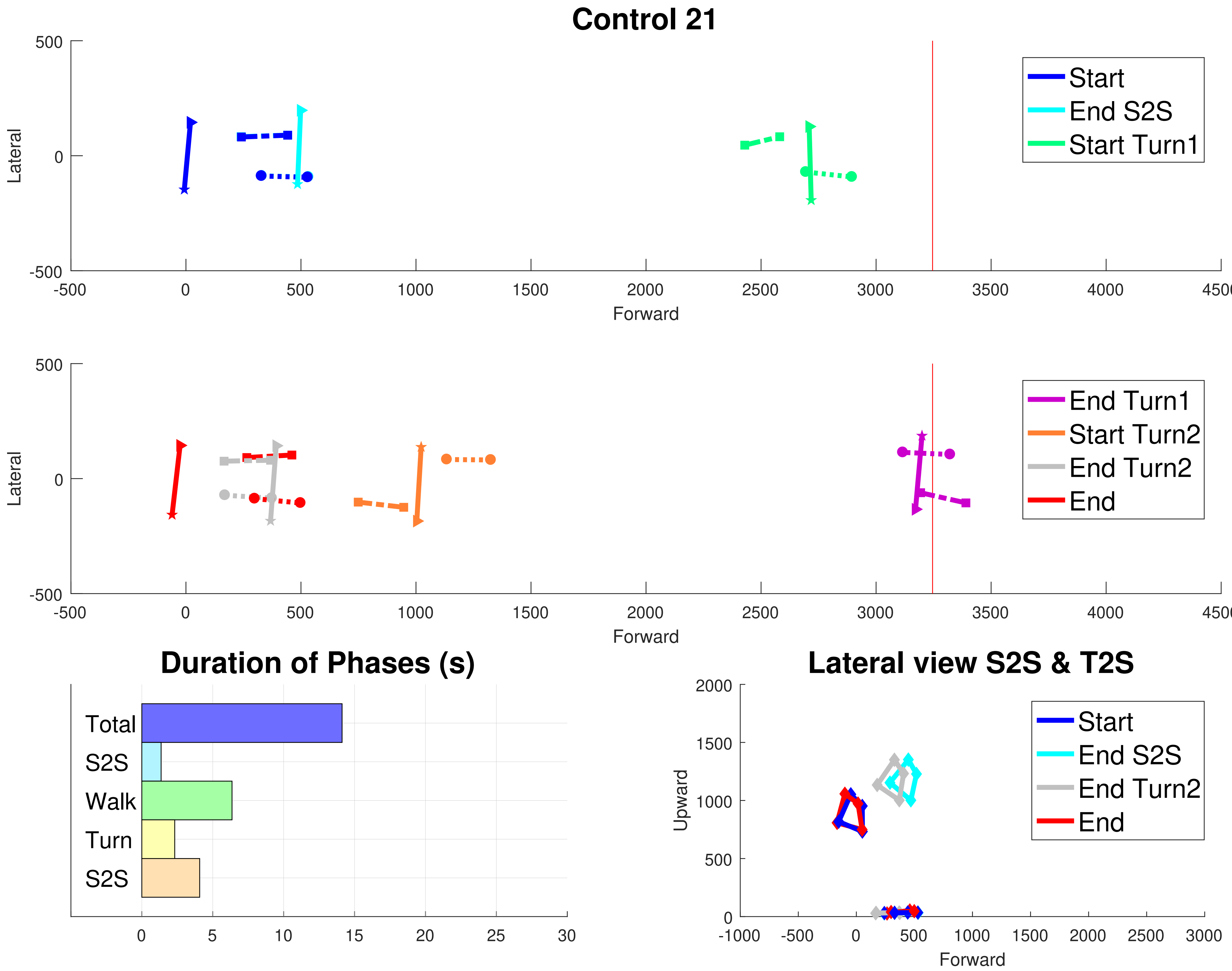

Control 21
Lateral
Forward
Start
End S2S
Start Turn1
End Turn1
Start Turn2
End Turn2
End
Duration of Phases (s)
Total
S2S
Walk
Turn
S2S
Lateral view S2S & T2S
Upward
Forward
Start
End S2S
End Turn2
End

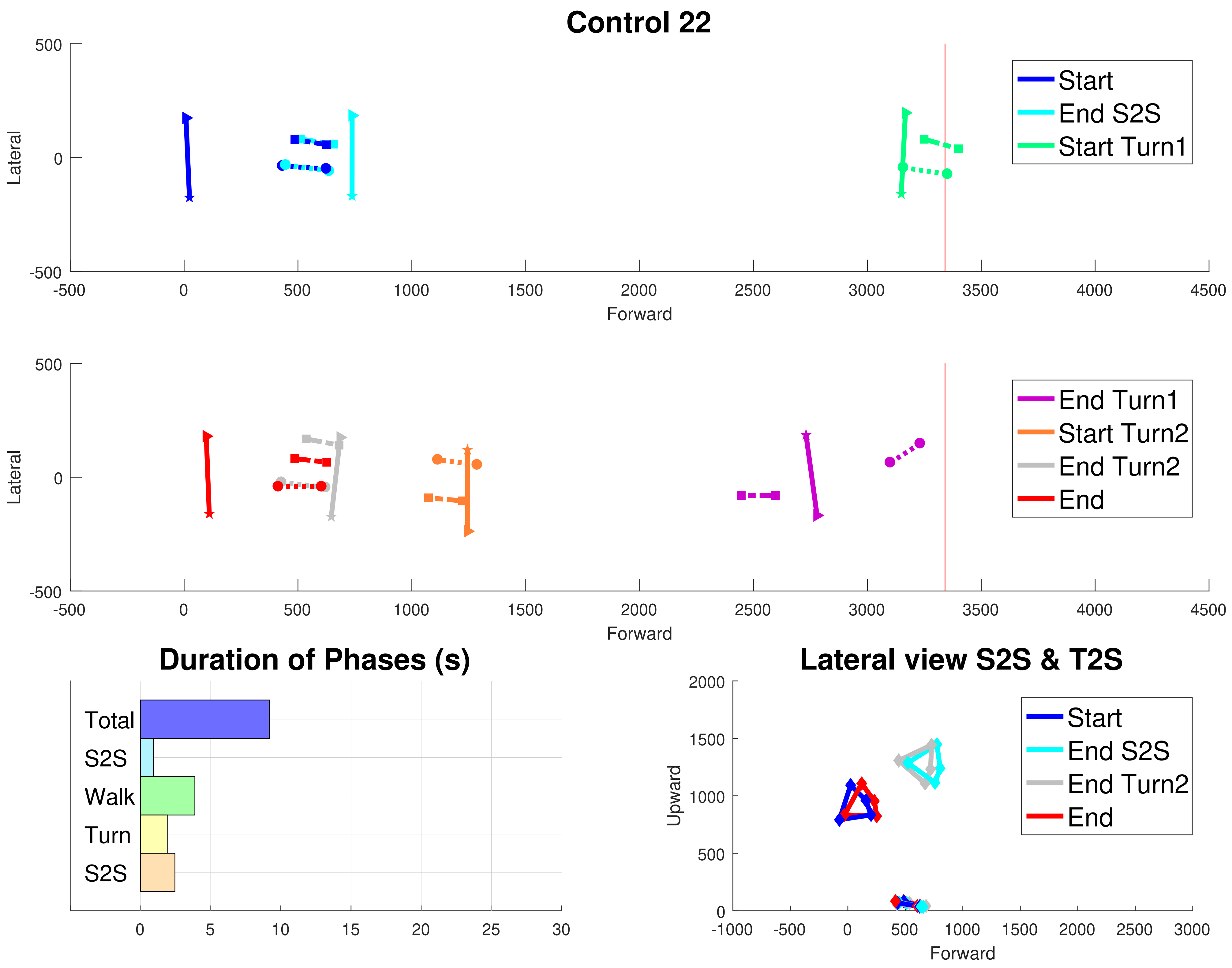
Control 22
Lateral
Forward
Start
End S2S
Start Turn1
End Turn1
Start Turn2
End Turn2
End
Duration of Phases (s)
Total
S2S
Walk
Turn
S2S
Lateral view S2S & T2S
Upward
Forward
Start
End S2S
End Turn2
End

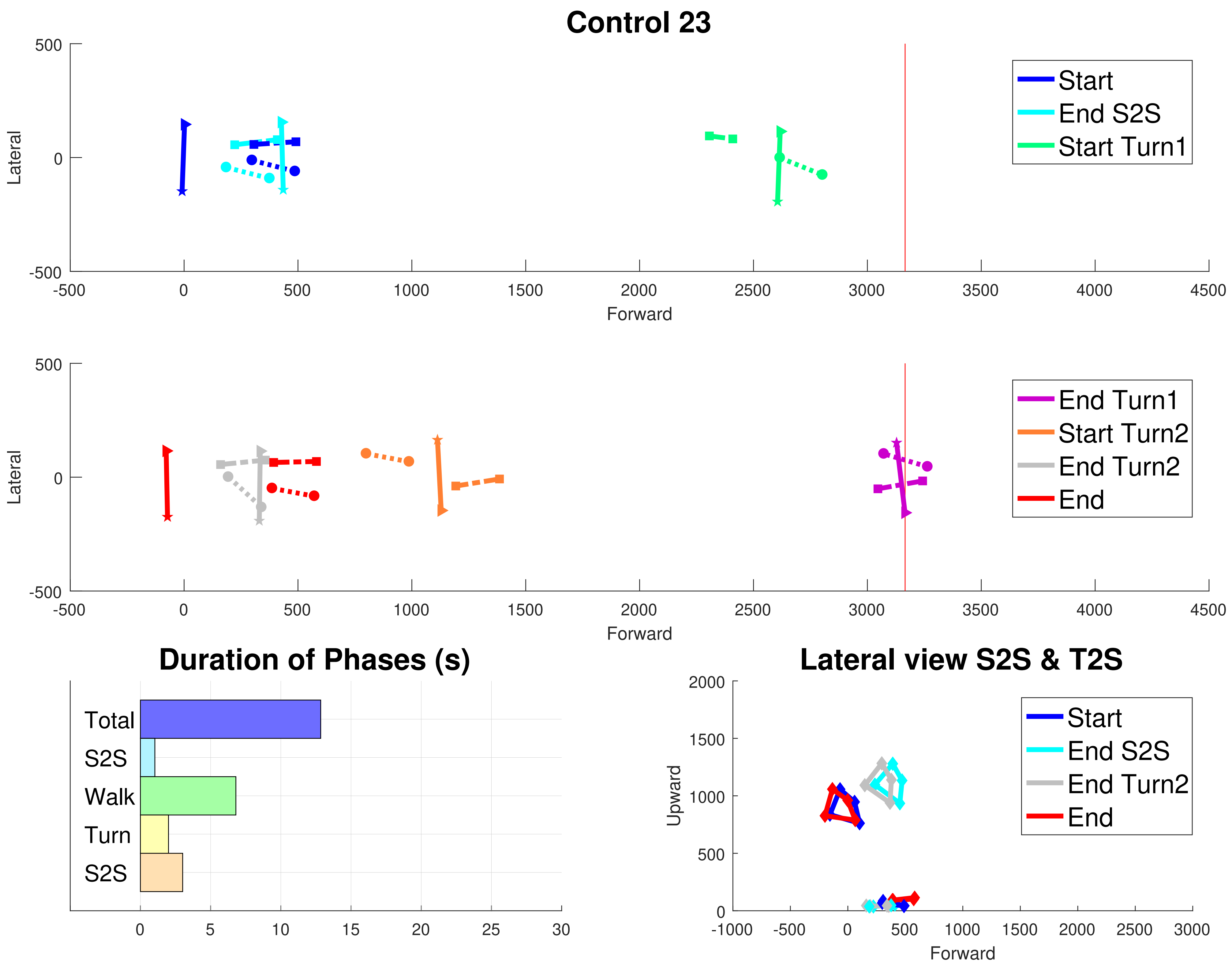
Control 23
Lateral
Forward
Start
End S2S
Start Turn1
End Turn1
Start Turn2
End Turn2
End
Duration of Phases (s)
Total
S2S
Walk
Turn
S2S
Lateral view S2S & T2S
Upward
Forward
Start
End S2S
End Turn2
End

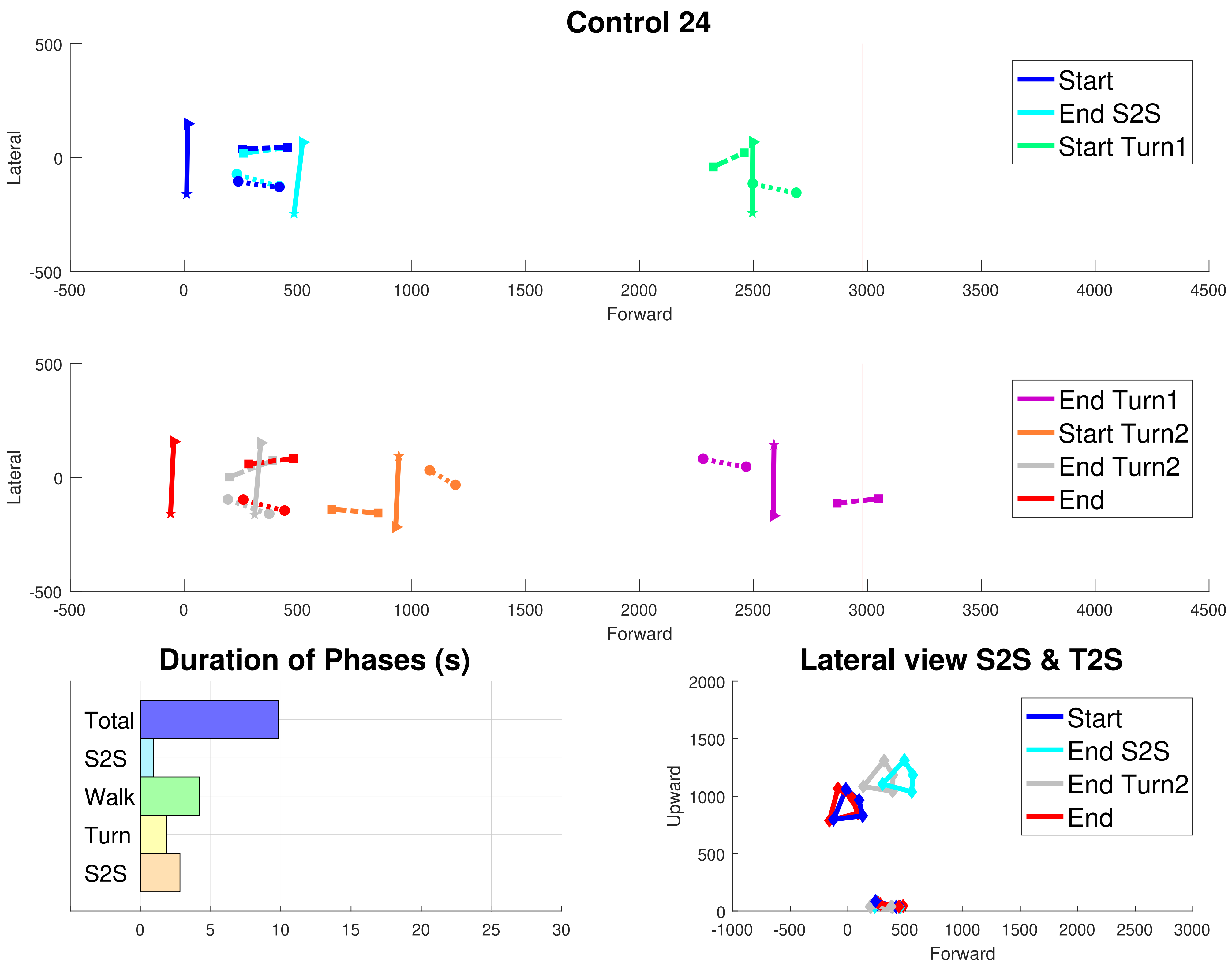
Control 24
Lateral
Forward
Start
End S2S
Start Turn1
End Turn1
Start Turn2
End Turn2
End
Duration of Phases (s)
Total
S2S
Walk
Turn
S2S
Lateral view S2S & T2S
Upward
Forward
Start
End S2S
End Turn2
End

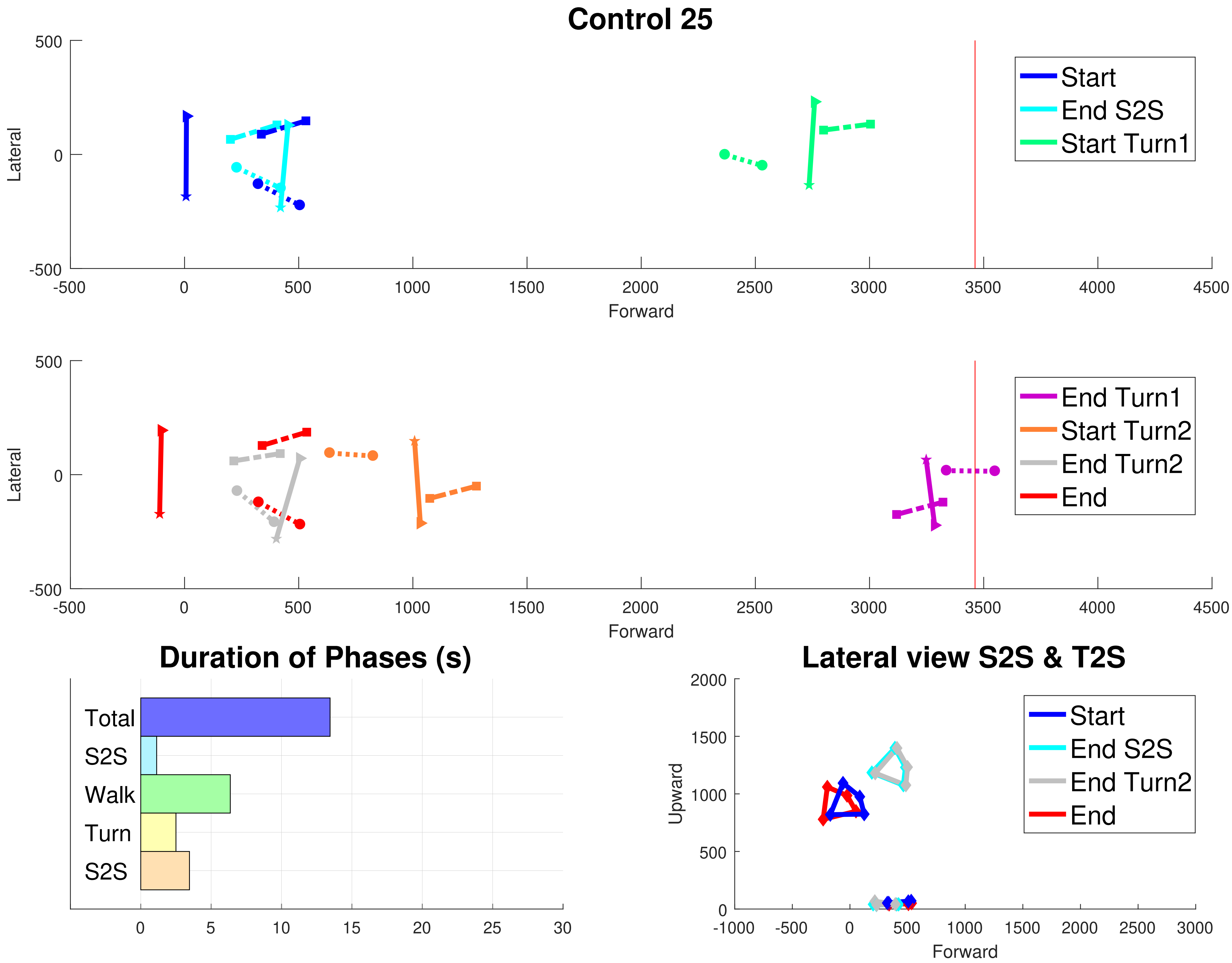
Control 25
Lateral
Forward
Start
End S2S
Start Turn1
End Turn1
Start Turn2
End Turn2
End
Duration of Phases (s)
Total
S2S
Walk
Turn
S2S
Lateral view S2S & T2S
Upward
Forward
Start
End S2S
End Turn2
End

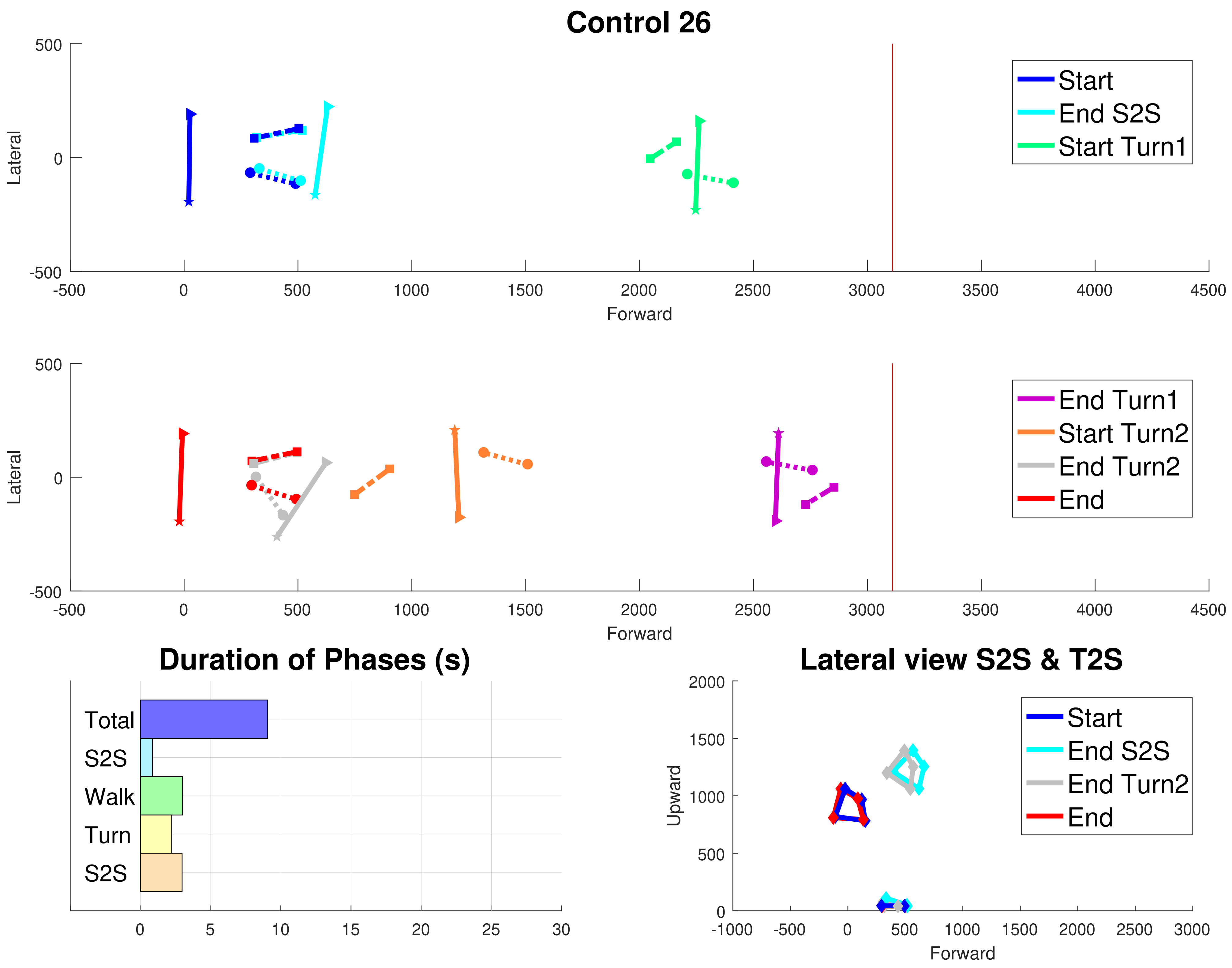
Control 26
Lateral
Forward
Start
End S2S
Start Turn1
End Turn1
Start Turn2
End Turn2
End
Duration of Phases (s)
Total
S2S
Walk
Turn
S2S
Lateral view S2S & T2S
Upward
Forward
Start
End S2S
End Turn2
End

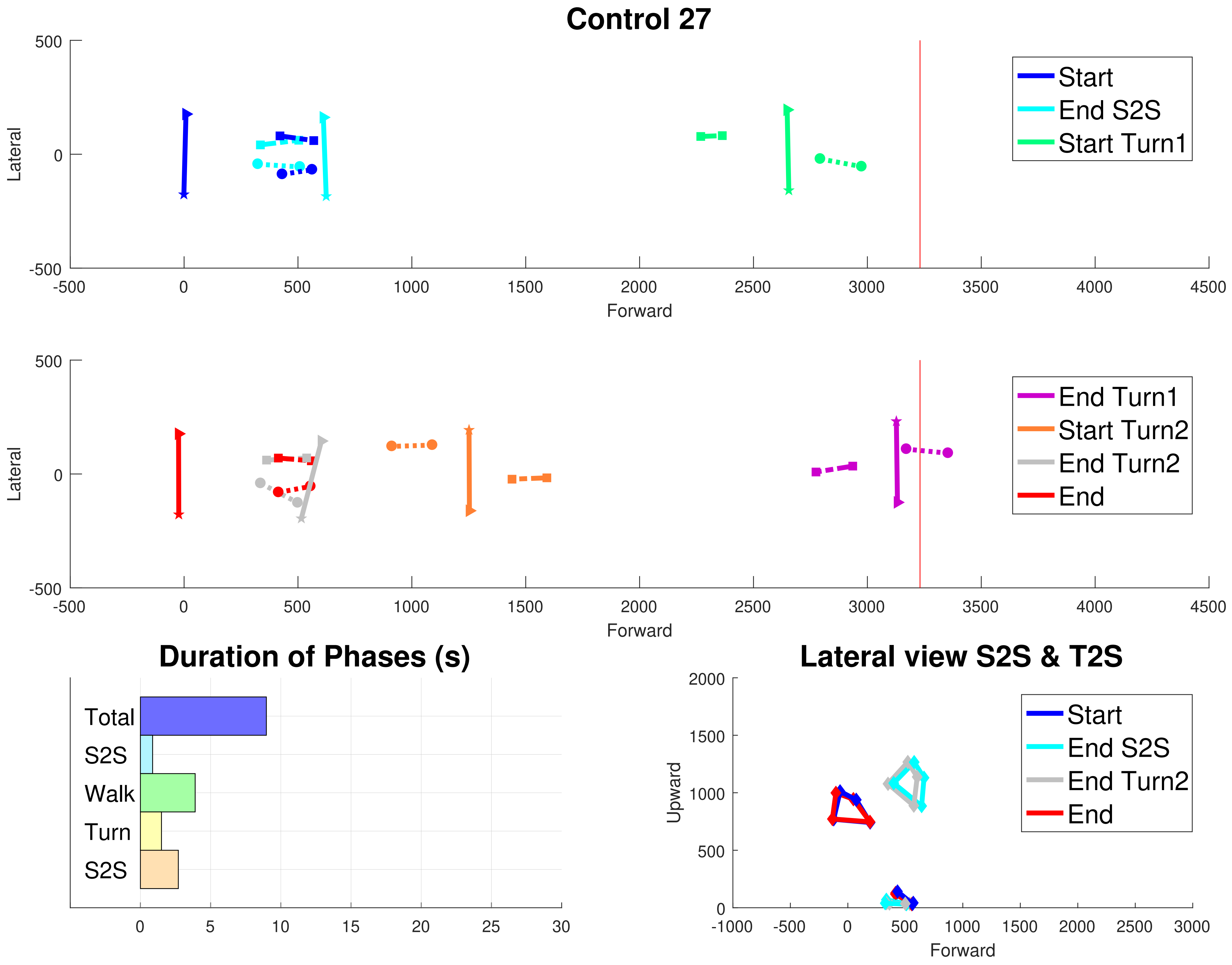

Control 27
Lateral
Forward
500
0
-500
-500
0
500
1000
1500
2000
2500
3000
3500
4000
4500
Start
End S2S
Start Turn1
End Turn1
Start Turn2
End Turn2
End
Duration of Phases (s)
Total
S2S
Walk
Turn
S2S
0
5
10
15
20
25
30
Lateral view S2S & T2S
Upward
2000
1500
1000
500
-1000
Start
End S2S
End Turn2
End

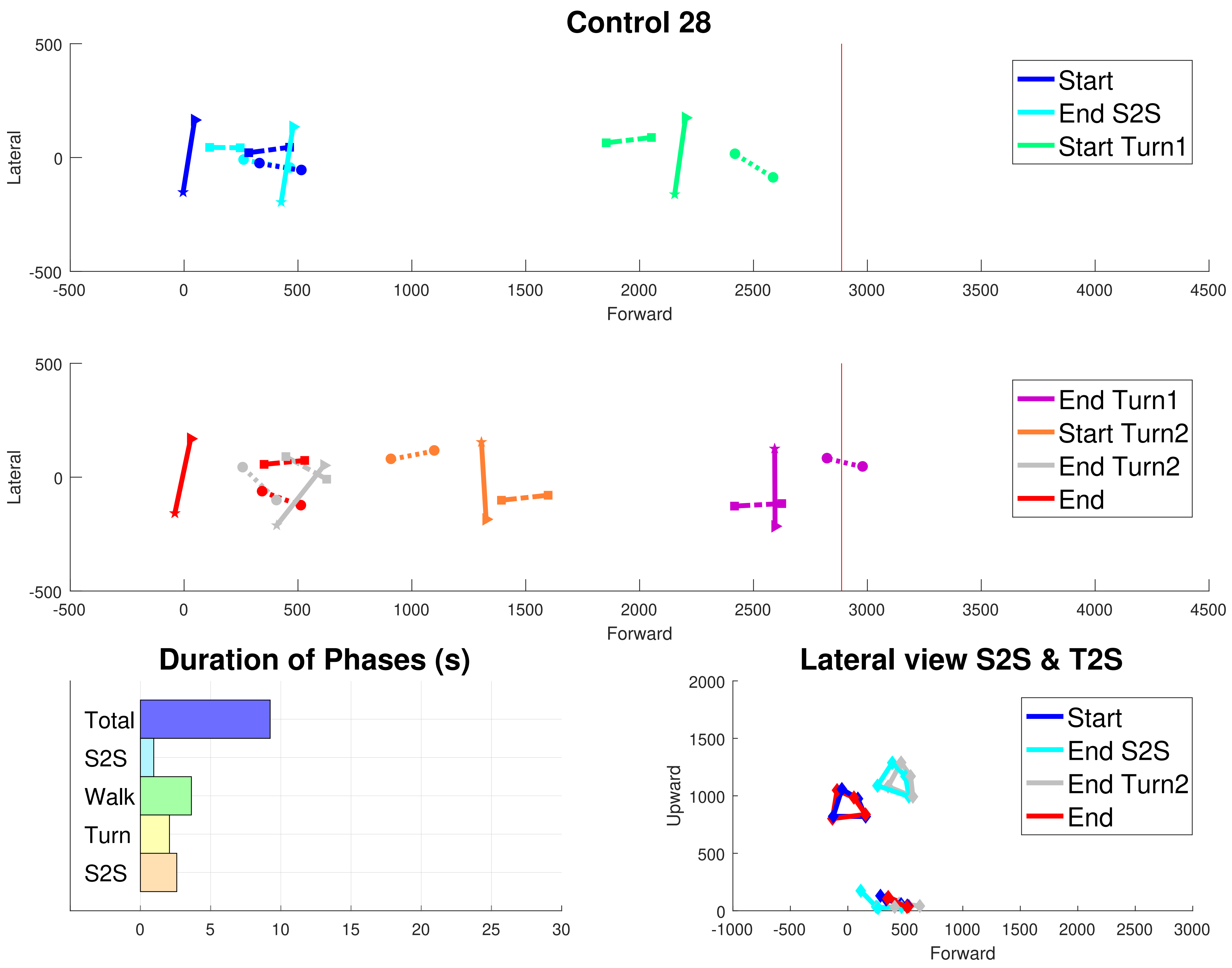
Control 28
Start
End S2S
Start Turn1
Lateral
Forward
End Turn1
Start Turn2
End Turn2
End
Duration of Phases (s)
Total
S2S
Walk
Turn
S2S
Lateral view S2S & T2S
Upward
Forward

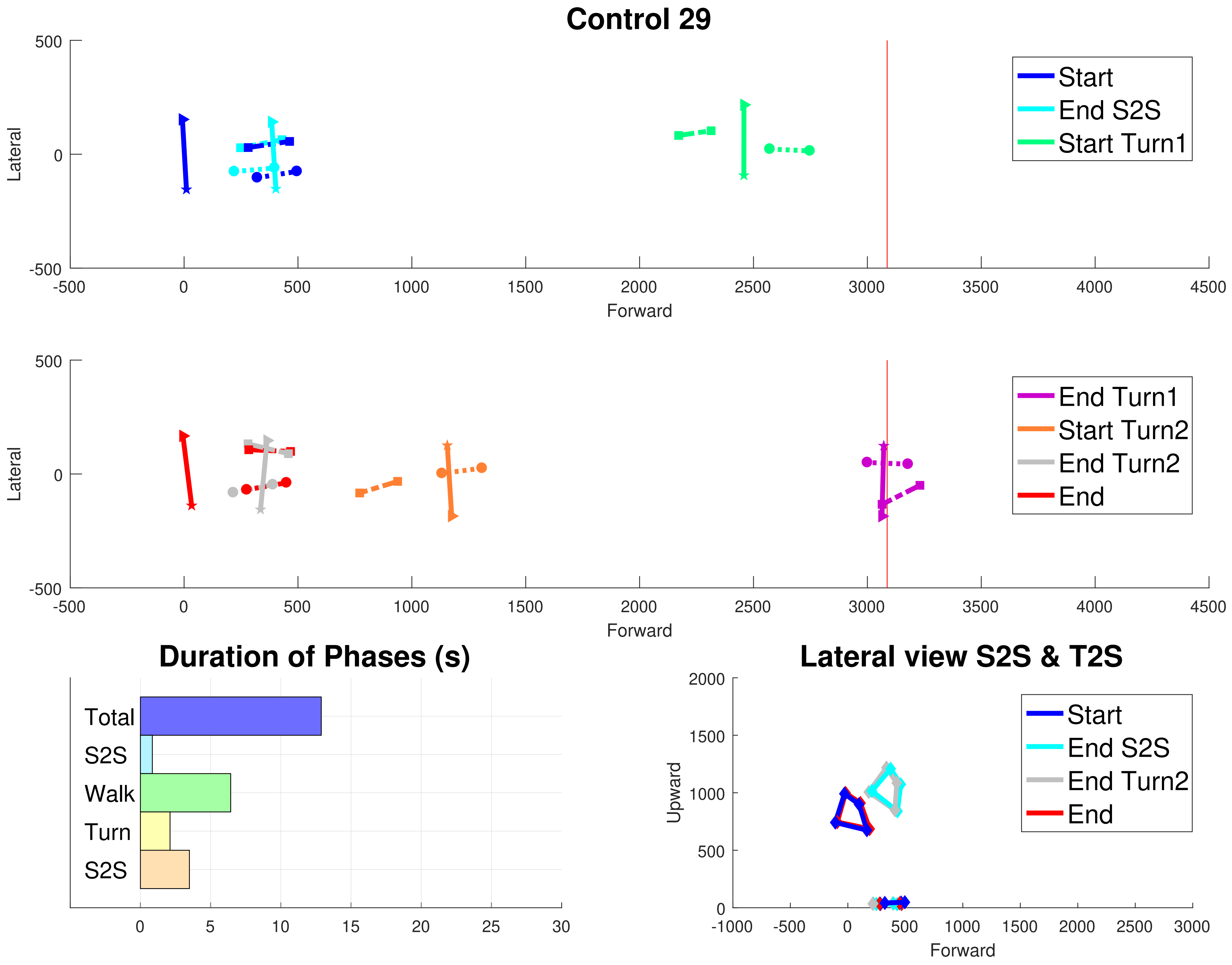

Control 29
Lateral
Forward
Start
End S2S
Start Turn1
End Turn1
Start Turn2
End Turn2
End
Duration of Phases (s)
Total
S2S
Walk
Turn
S2S
Lateral view S2S & T2S
Upward
Forward
Start
End S2S
End Turn2
End

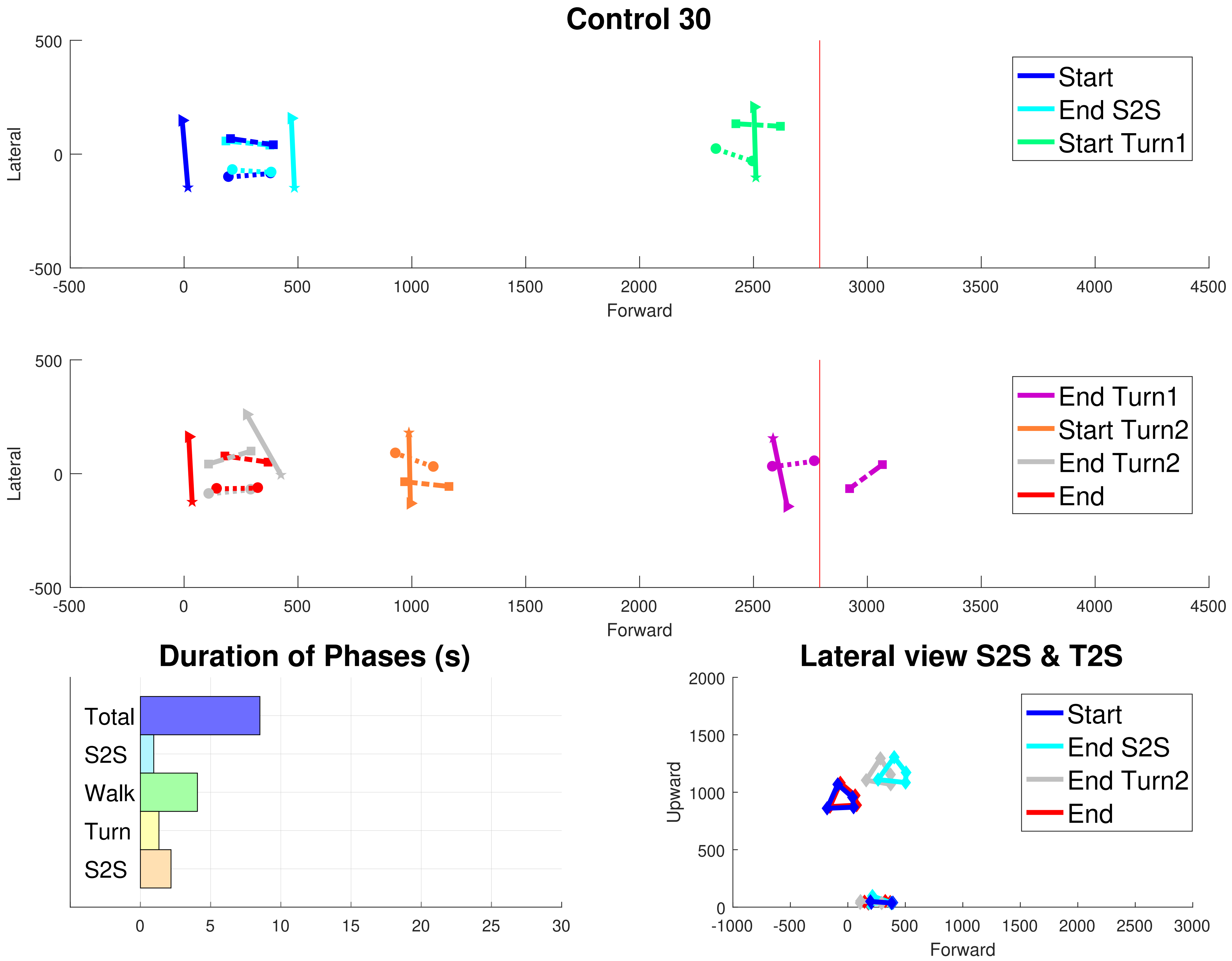
Control 30
Lateral
Forward
Start
End S2S
Start Turn1
End Turn1
Start Turn2
End Turn2
End
Duration of Phases (s)
Total
S2S
Walk
Turn
S2S
Lateral view S2S & T2S
Upward
Forward
Start
End S2S
End Turn2
End

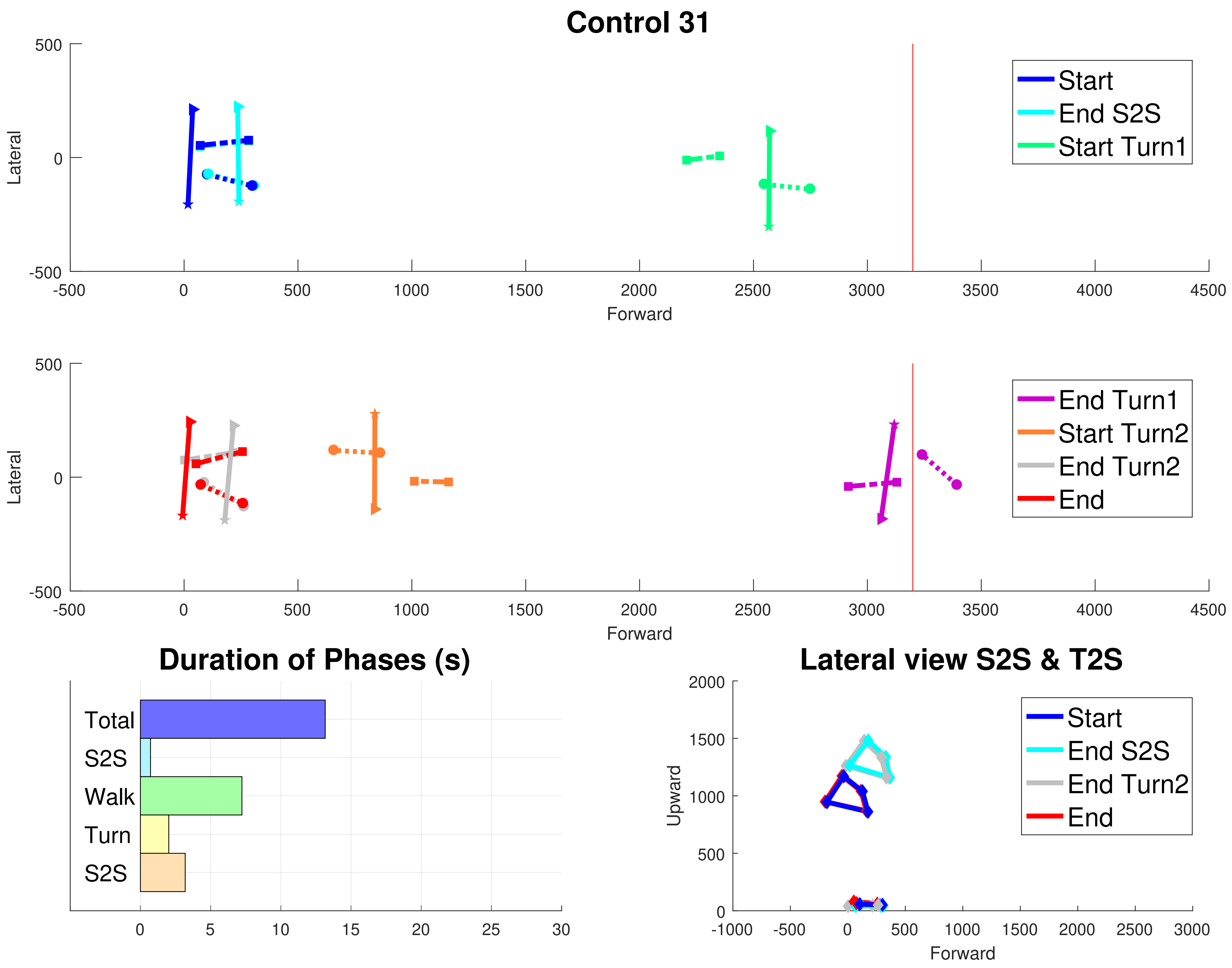

Control 31
Lateral
Forward
Start
End S2S
Start Turn1
End Turn1
Start Turn2
End Turn2
End
500
0
-500
-500
0
500
1000
1500
2000
2500
3000
3500
4000
4500
Duration of Phases (s)
Total
S2S
Walk
Turn
S2S
0
5
10
15
20
25
30
Lateral view S2S & T2S
Upward
2000
1500
1000
500
0
-1000
-500
0
500
1000
1500
2000
2500
3000
Forward
Start
End S2S
End Turn2
End

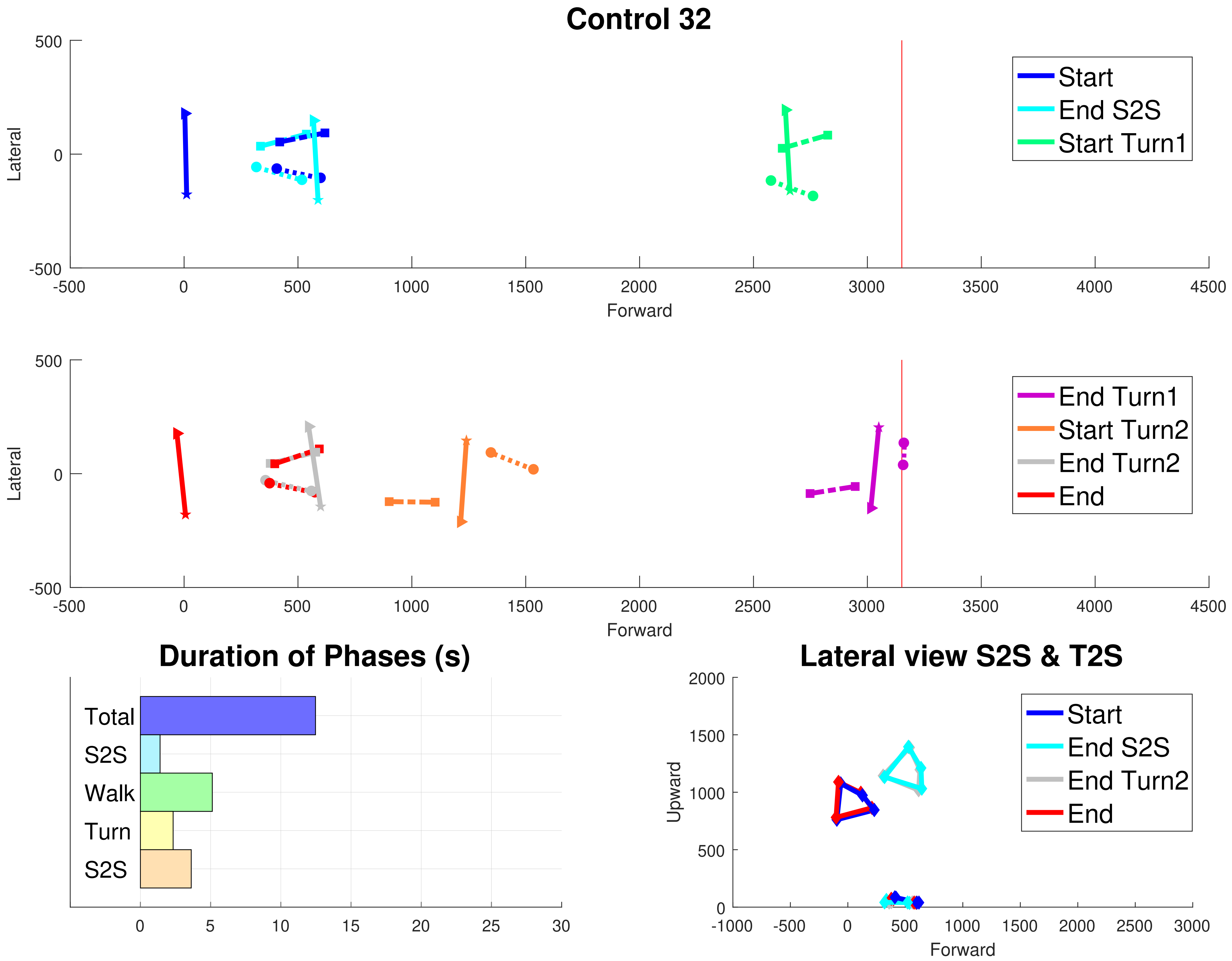

Control 32
Lateral
Forward
500
0
-500
-500
0
500
1000
1500
2000
2500
3000
3500
4000
4500
Start
End S2S
Start Turn1
End Turn1
Start Turn2
End Turn2
End
Duration of Phases (s)
Total
S2S
Walk
Turn
S2S
0
5
10
15
20
25
30
Lateral view S2S & T2S
Upward
2000
1500
1000
500
0
-1000
-500
0
500
1000
1500
2000
2500
3000
Start
End S2S
End Turn2
End

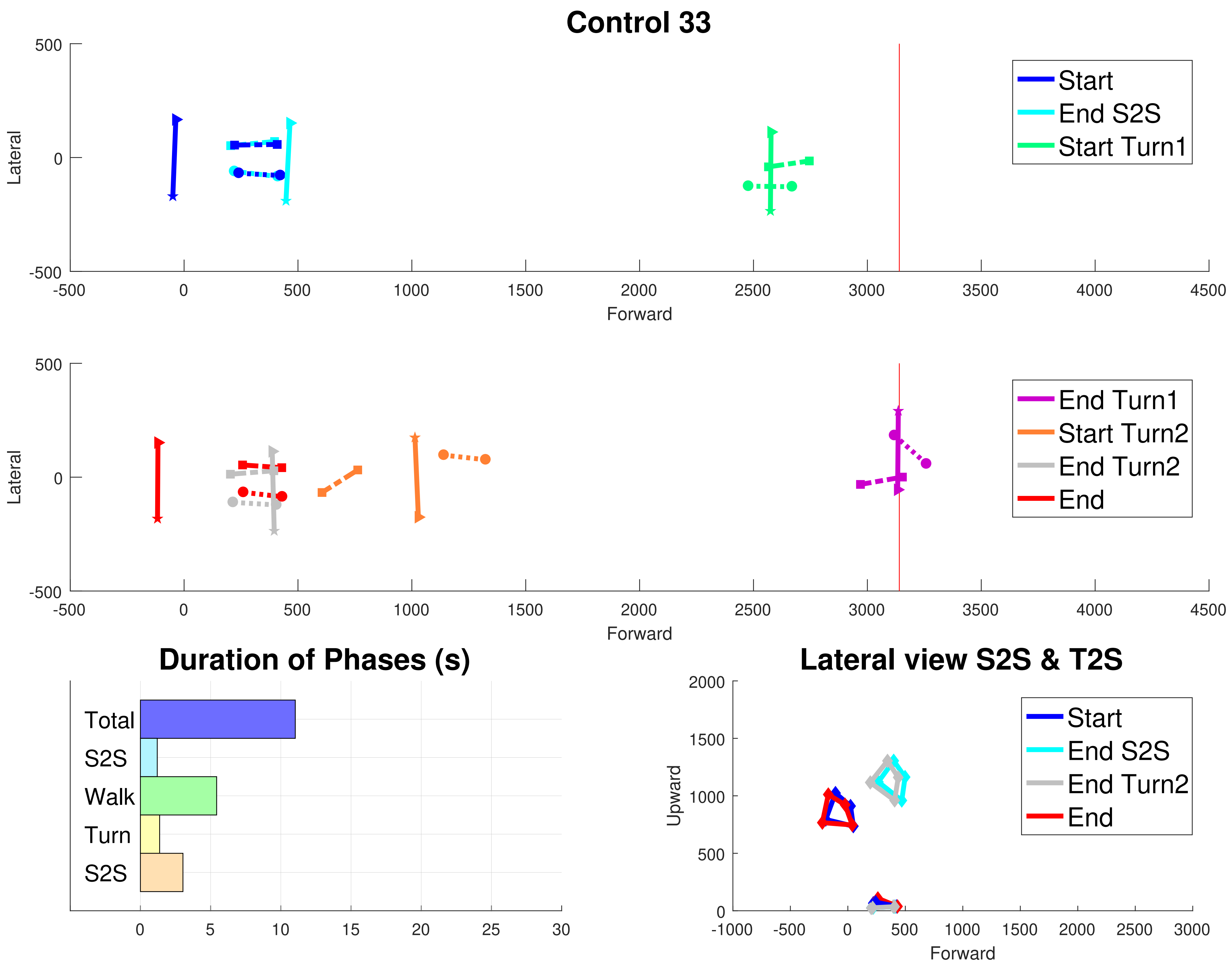

Control 33
Start
End S2S
Start Turn1
Lateral
Forward
500
0
-500
-500
0
500
1000
1500
2000
2500
3000
3500
4000
4500
End Turn1
Start Turn2
End Turn2
End
Duration of Phases (s)
Total
S2S
Walk
Turn
S2S
0
5
10
15
20
25
30
Lateral view S2S & T2S
Upward
2000
1500
1000
500
0
-1000
-500
0
500
1000
1500
2000
2500
3000
Start
End S2S
End Turn2
End

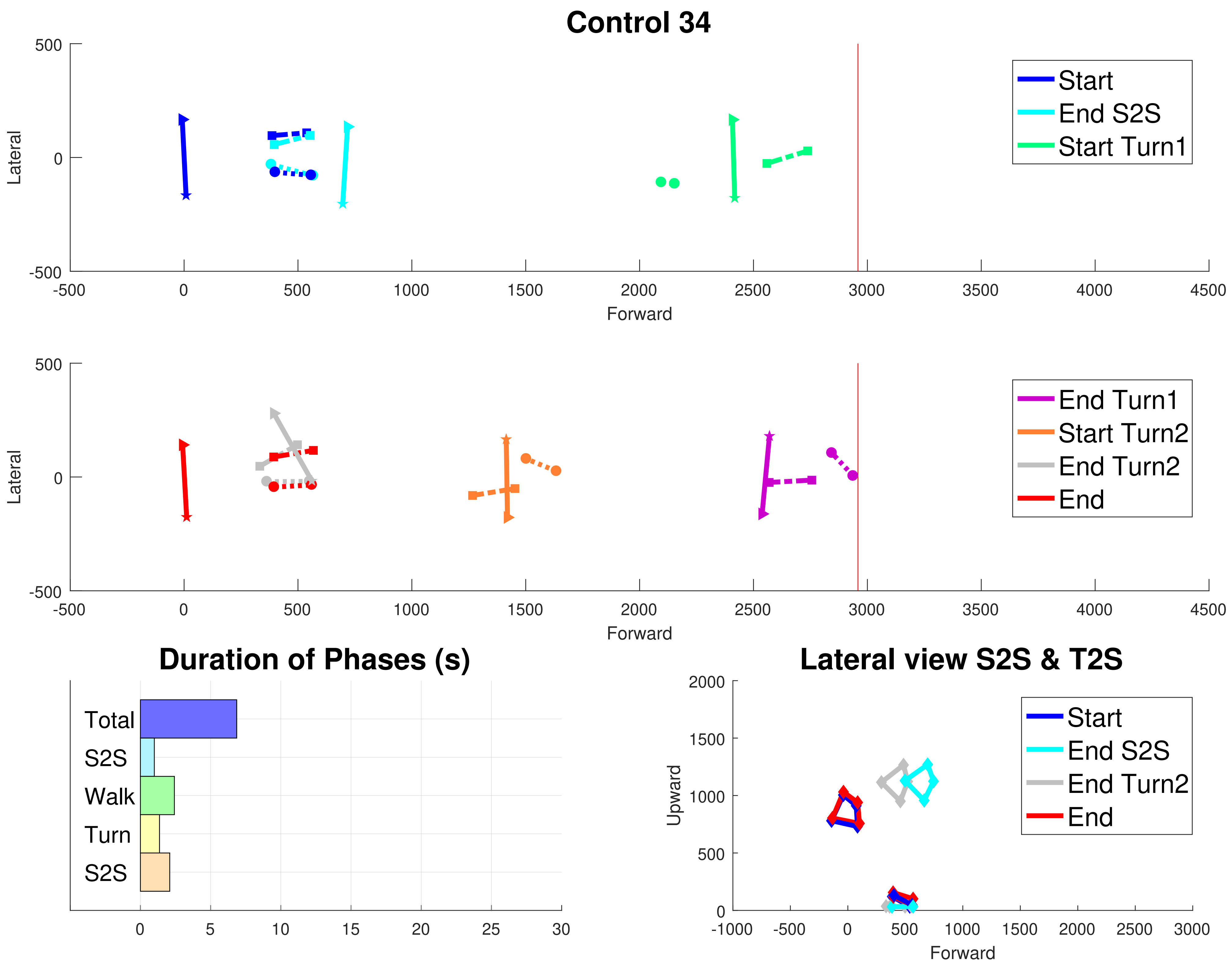

Control 34
Lateral
Forward
Start
End S2S
Start Turn1
End Turn1
Start Turn2
End Turn2
End
Duration of Phases (s)
Total
S2S
Walk
Turn
S2S
Lateral view S2S & T2S
Upward
Forward
Start
End S2S
End Turn2
End

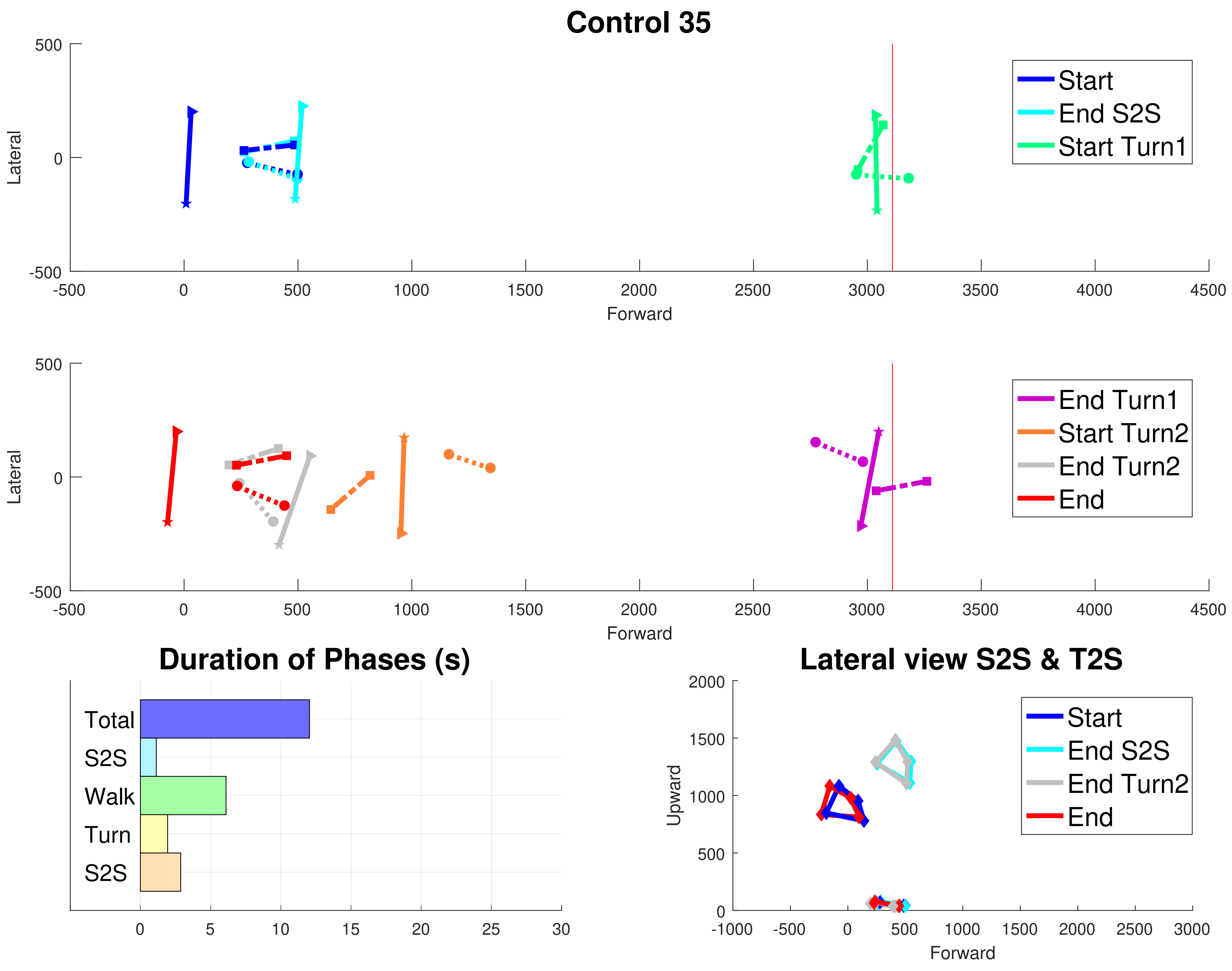

Control 35
Lateral
Forward
Start
End S2S
Start Turn1
End Turn1
Start Turn2
End Turn2
End
Duration of Phases (s)
Total
S2S
Walk
Turn
S2S
Lateral view S2S & T2S
Upward
Forward
Start
End S2S
End Turn2
End

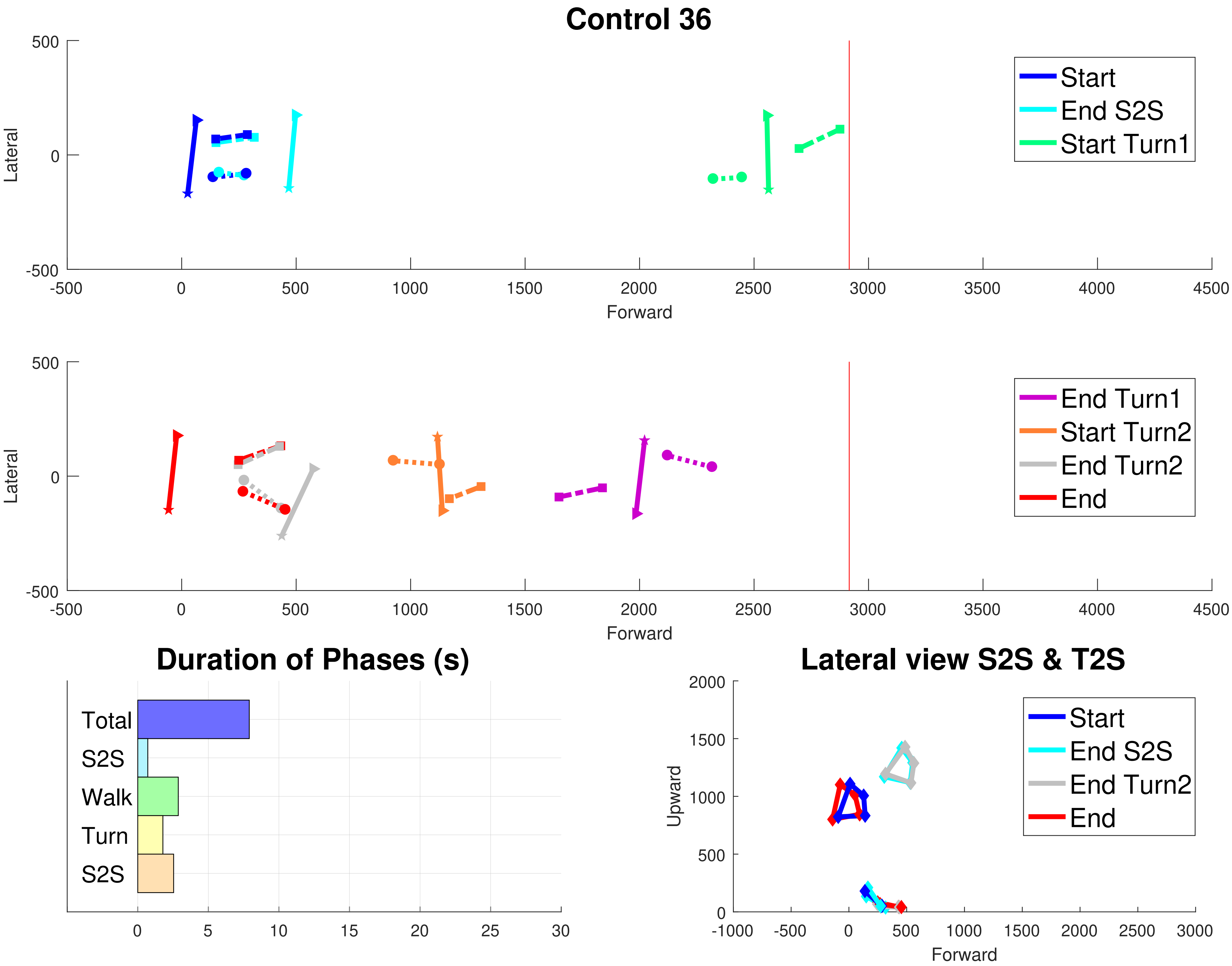
Control 36
Lateral
Forward
500
0
-500
-500
0
500
1000
1500
2000
2500
3000
3500
4000
4500
Start
End S2S
Start Turn1
End Turn1
Start Turn2
End Turn2
End
Duration of Phases (s)
Total
S2S
Walk
Turn
S2S
0
5
10
15
20
25
30
Lateral view S2S & T2S
Upward
2000
1500
1000
500
0
-1000
-500
0
500
1000
1500
2000
2500
3000
Forward
Start
End S2S
End Turn2
End

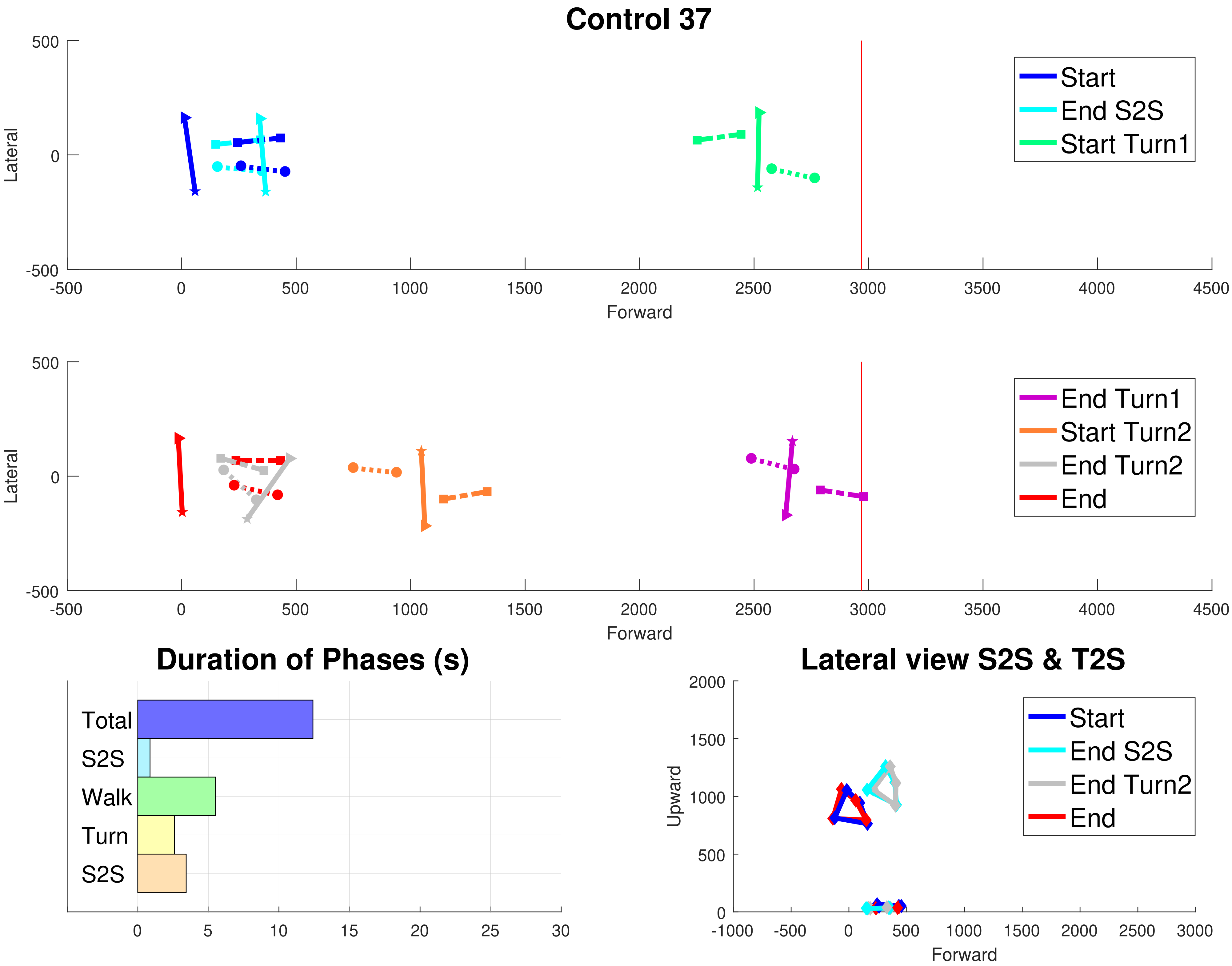
Control 37
Lateral
Forward
Start
End S2S
Start Turn1
End Turn1
Start Turn2
End Turn2
End
Duration of Phases (s)
Total
S2S
Walk
Turn
S2S
Lateral view S2S & T2S
Upward
Forward
Start
End S2S
End Turn2
End

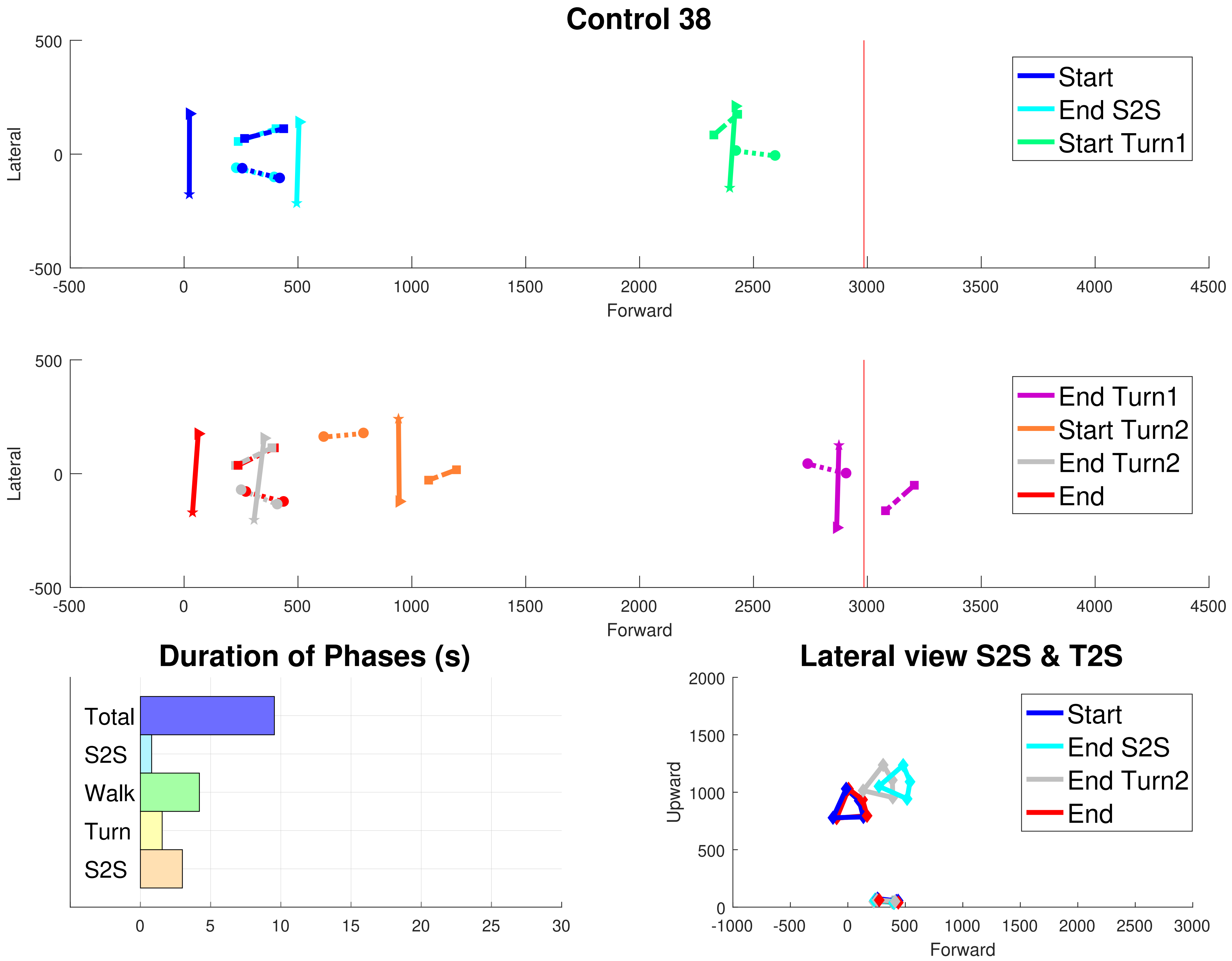

Control 38
Lateral
Forward
Start
End S2S
Start Turn1
End Turn1
Start Turn2
End Turn2
End
Duration of Phases (s)
Total
S2S
Walk
Turn
S2S
Lateral view S2S & T2S
Upward
Forward
Start
End S2S
End Turn2
End

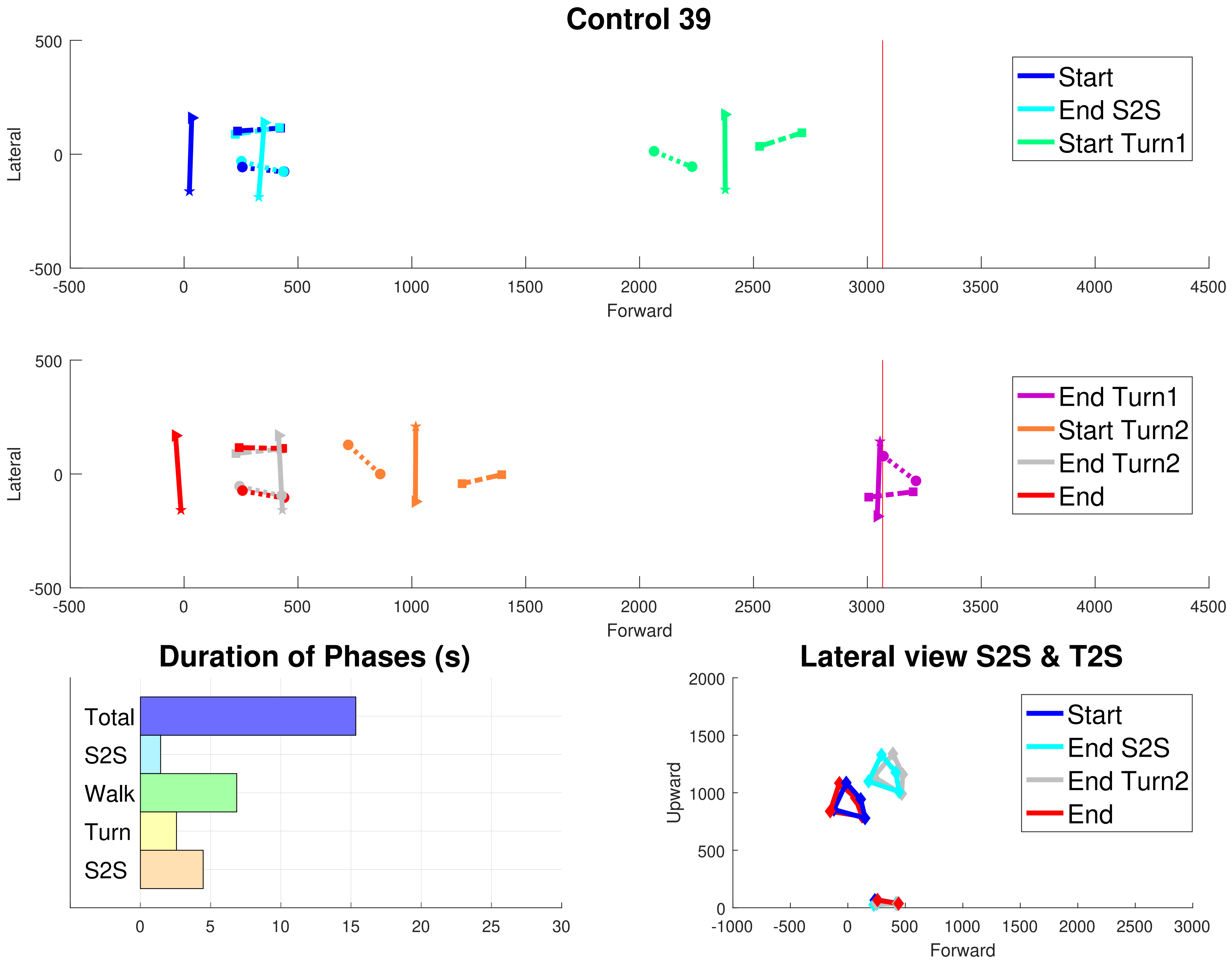
Control 39
Start
End S2S
Start Turn1
Lateral
Forward
500
0
-500
-500
0
500
1000
1500
2000
2500
3000
3500
4000
4500
End Turn1
Start Turn2
End Turn2
End
Lateral
Forward
Duration of Phases (s)
Total
S2S
Walk
Turn
S2S
0
5
10
15
20
25
30
Lateral view S2S & T2S
Start
End S2S
End Turn2
End
Upward
2000
1500
1000
500
0
-1000
-500
0
500
1000
1500
2000
2500
3000
Forward

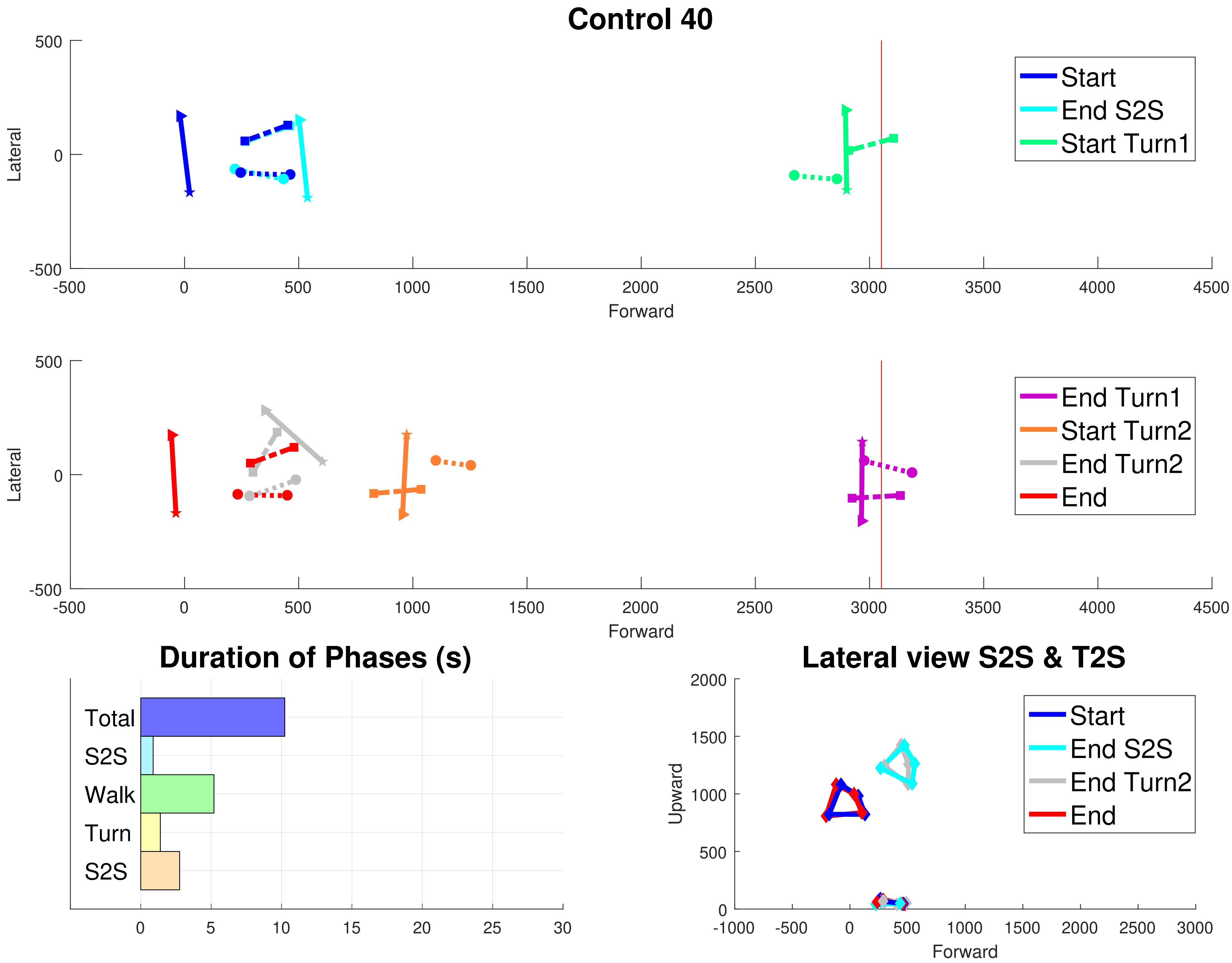
Control 40
Lateral
Forward
Start
End S2S
Start Turn1
End Turn1
Start Turn2
End Turn2
End
500
0
-500
-500
0
500
1000
1500
2000
2500
3000
3500
4000
4500
Duration of Phases (s)
Total
S2S
Walk
Turn
S2S
0
5
10
15
20
25
30
Lateral view S2S & T2S
Upward
2000
1500
1000
500
0
-1000
-500
0
500
1000
1500
2000
2500
3000
Forward
Start
End S2S
End Turn2
End

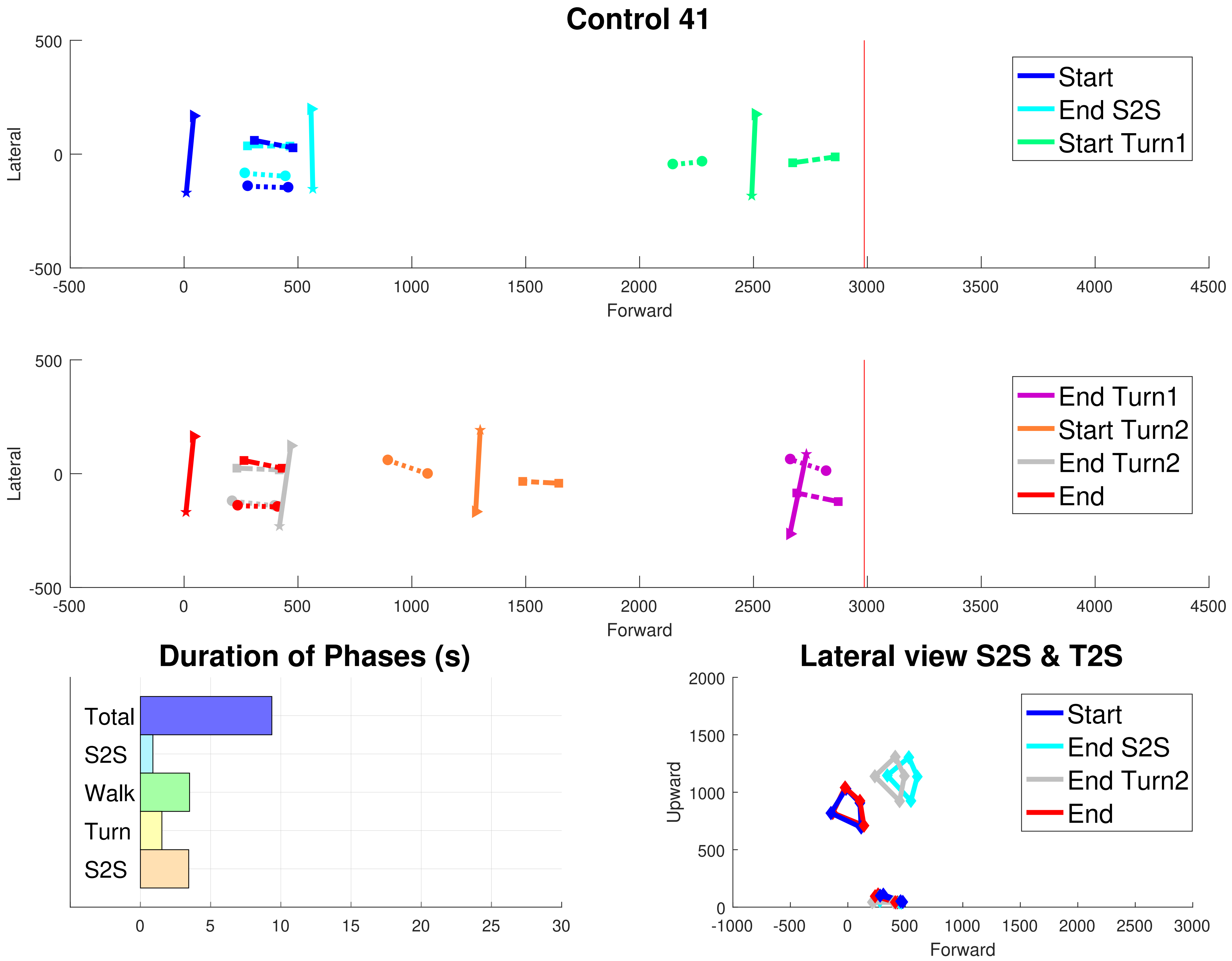

Control 41
Lateral
Forward
Start
End S2S
Start Turn1
End Turn1
Start Turn2
End Turn2
End
Duration of Phases (s)
Total
S2S
Walk
Turn
S2S
Lateral view S2S & T2S
Upward
Forward
Start
End S2S
End Turn2
End

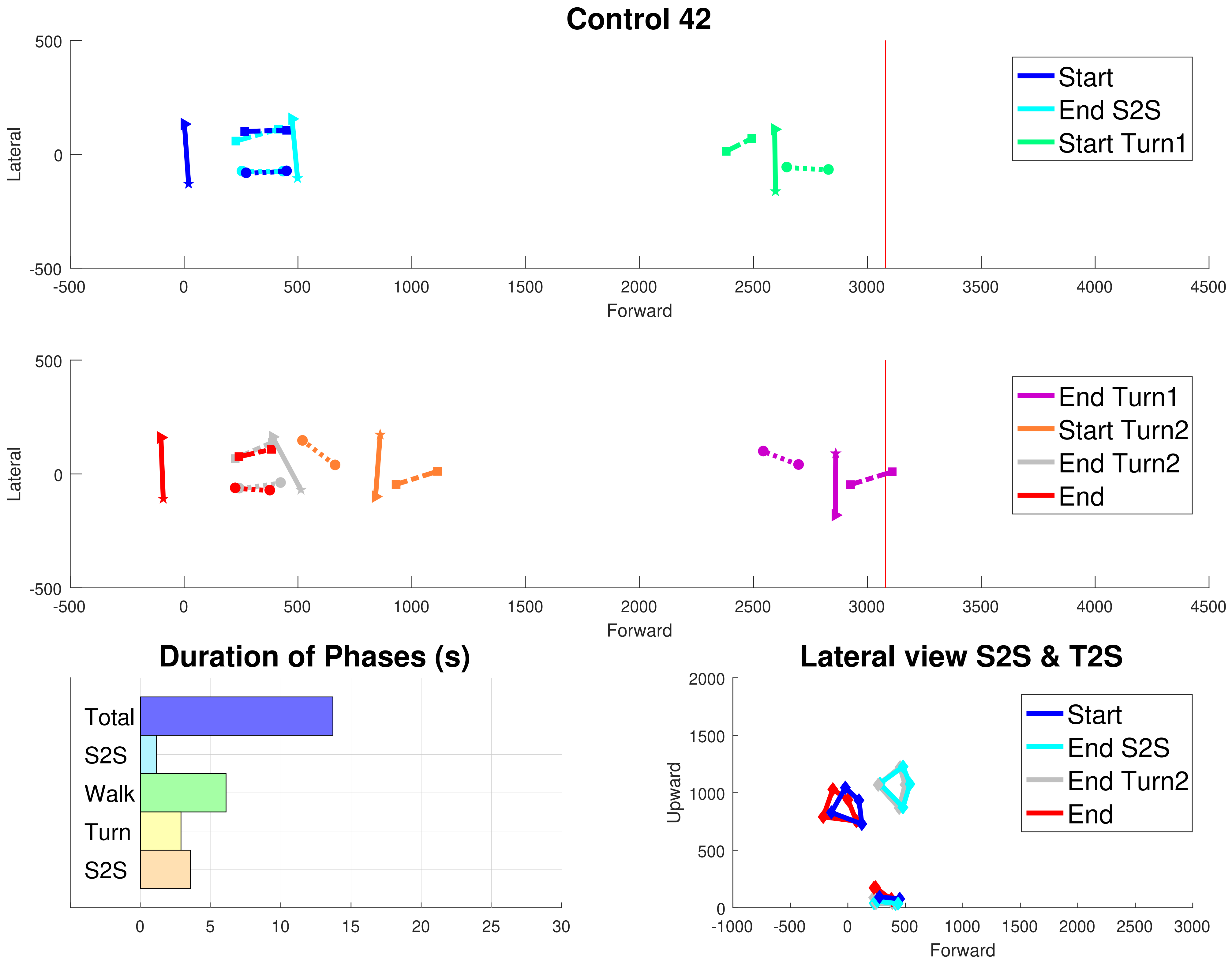
Control 42
Lateral
Forward
Start
End S2S
Start Turn1
End Turn1
Start Turn2
End Turn2
End
500
0
-500
-500
0
500
1000
1500
2000
2500
3000
3500
4000
4500
Duration of Phases (s)
Total
S2S
Walk
Turn
S2S
0
5
10
15
20
25
30
Lateral view S2S & T2S
Upward
Forward
2000
1500
1000
500
0
-1000
-500
0
500
1000
1500
2000
2500
3000
Start
End S2S
End Turn2
End

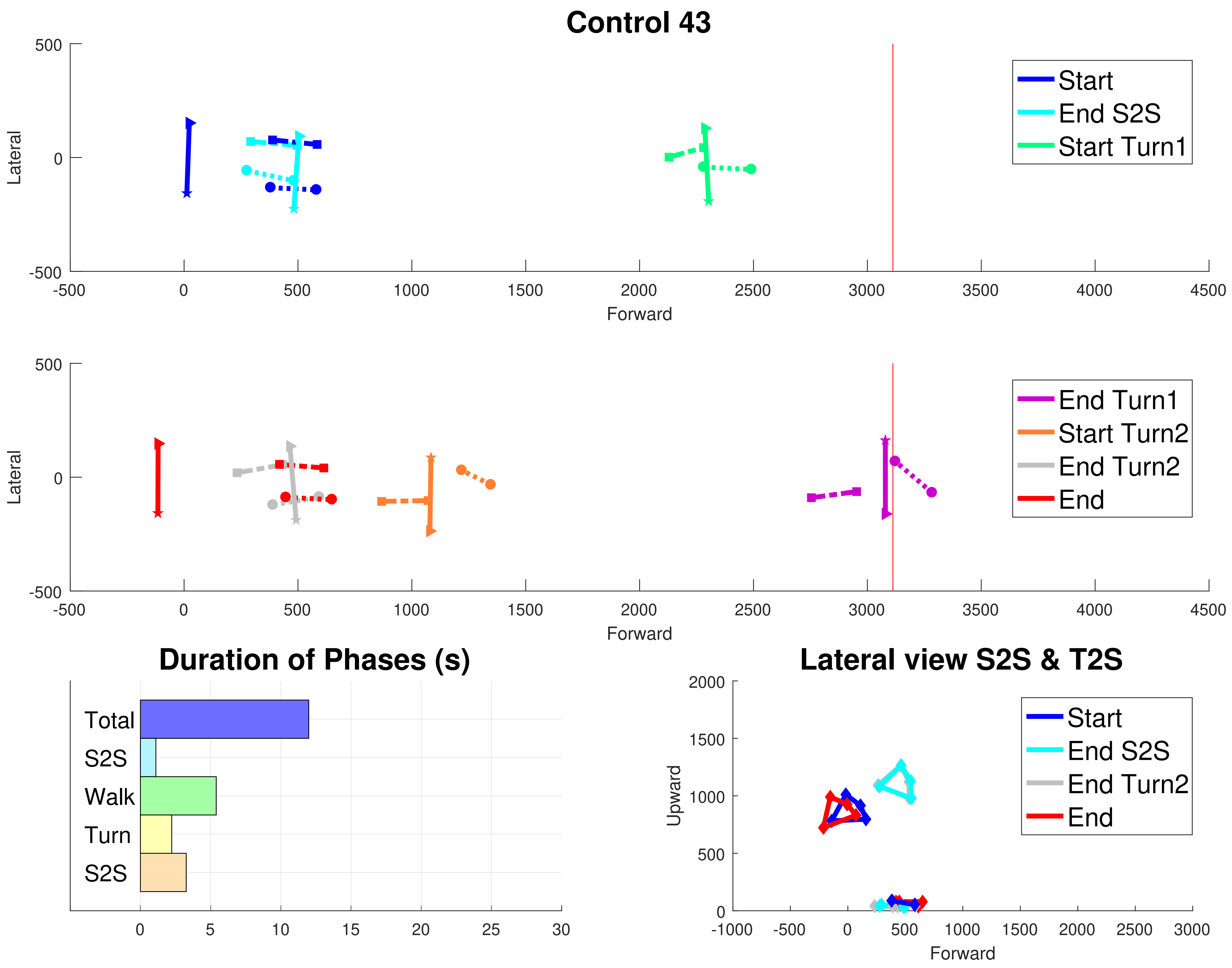

Control 43
Lateral
Forward
Start
End S2S
Start Turn1
End Turn1
Start Turn2
End Turn2
End
Duration of Phases (s)
Total
S2S
Walk
Turn
S2S
Lateral view S2S & T2S
Upward
Forward
Start
End S2S
End Turn2
End

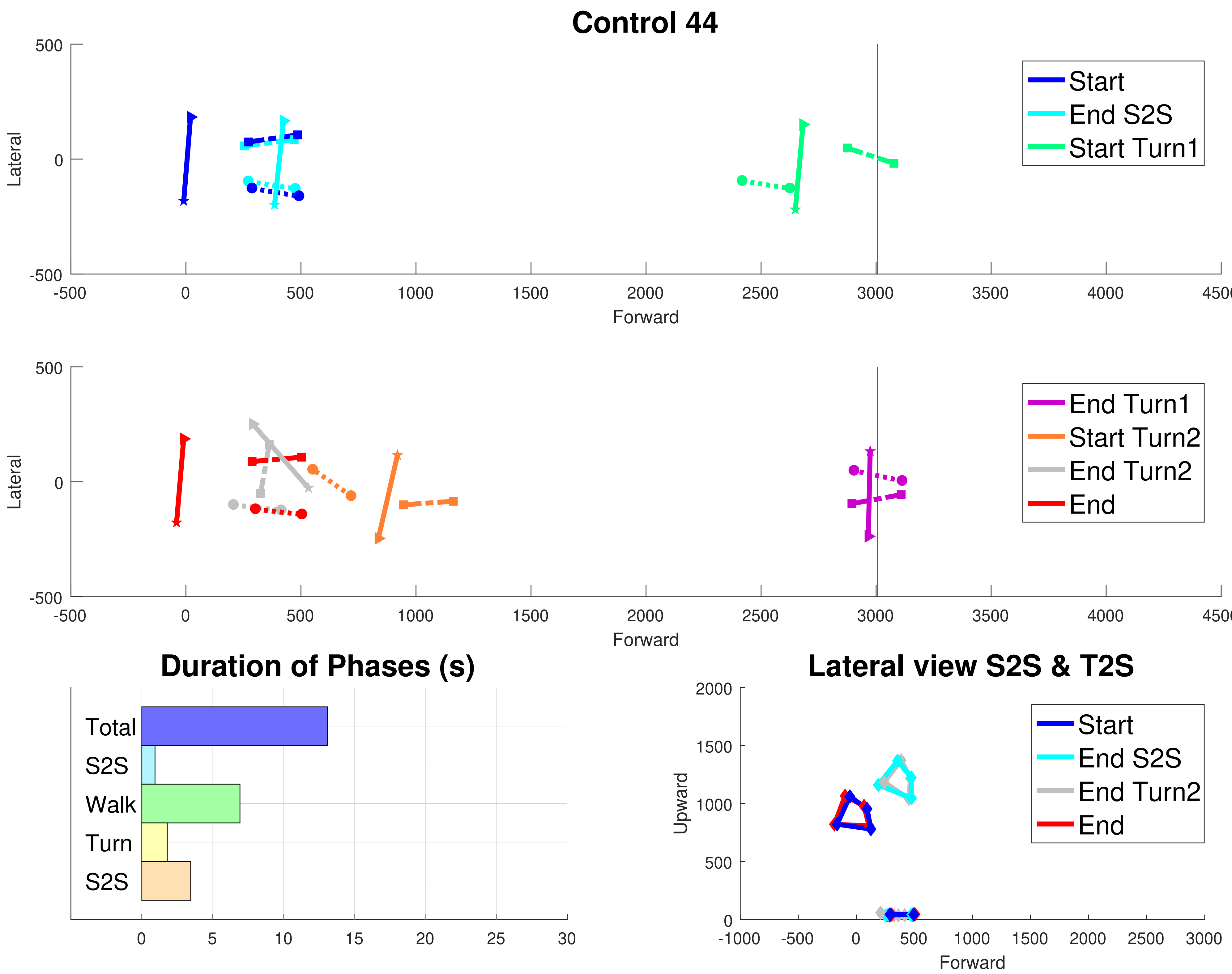
Control 44
Lateral
Forward
Start
End S2S
Start Turn1
End Turn1
Start Turn2
End Turn2
End
Duration of Phases (s)
Total
S2S
Walk
Turn
S2S
Lateral view S2S & T2S
Upward
Start
End S2S
End Turn2
End

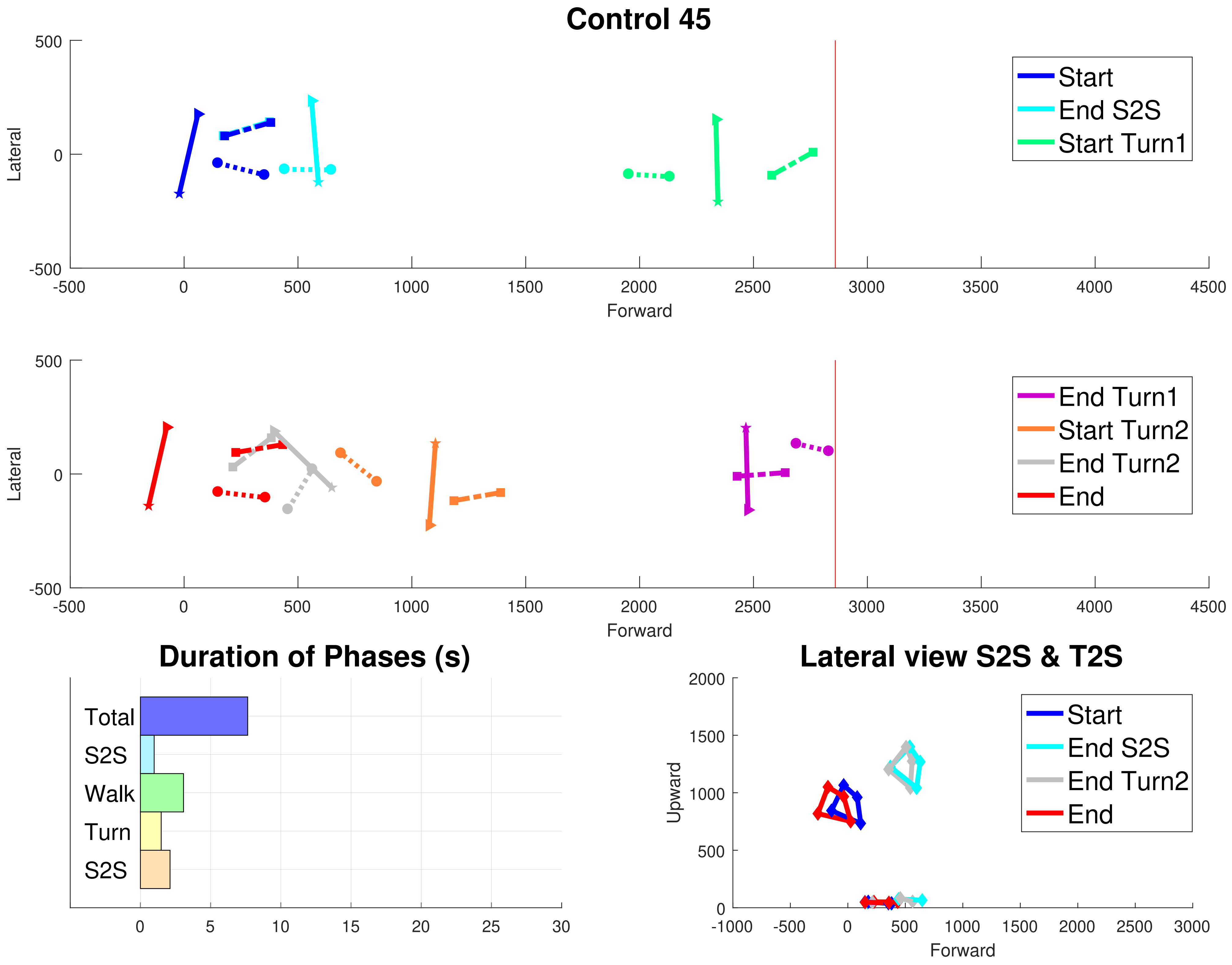

Control 45
Lateral
Forward
Start
End S2S
Start Turn1
End Turn1
Start Turn2
End Turn2
End
Duration of Phases (s)
Total
S2S
Walk
Turn
S2S
Lateral view S2S & T2S
Upward

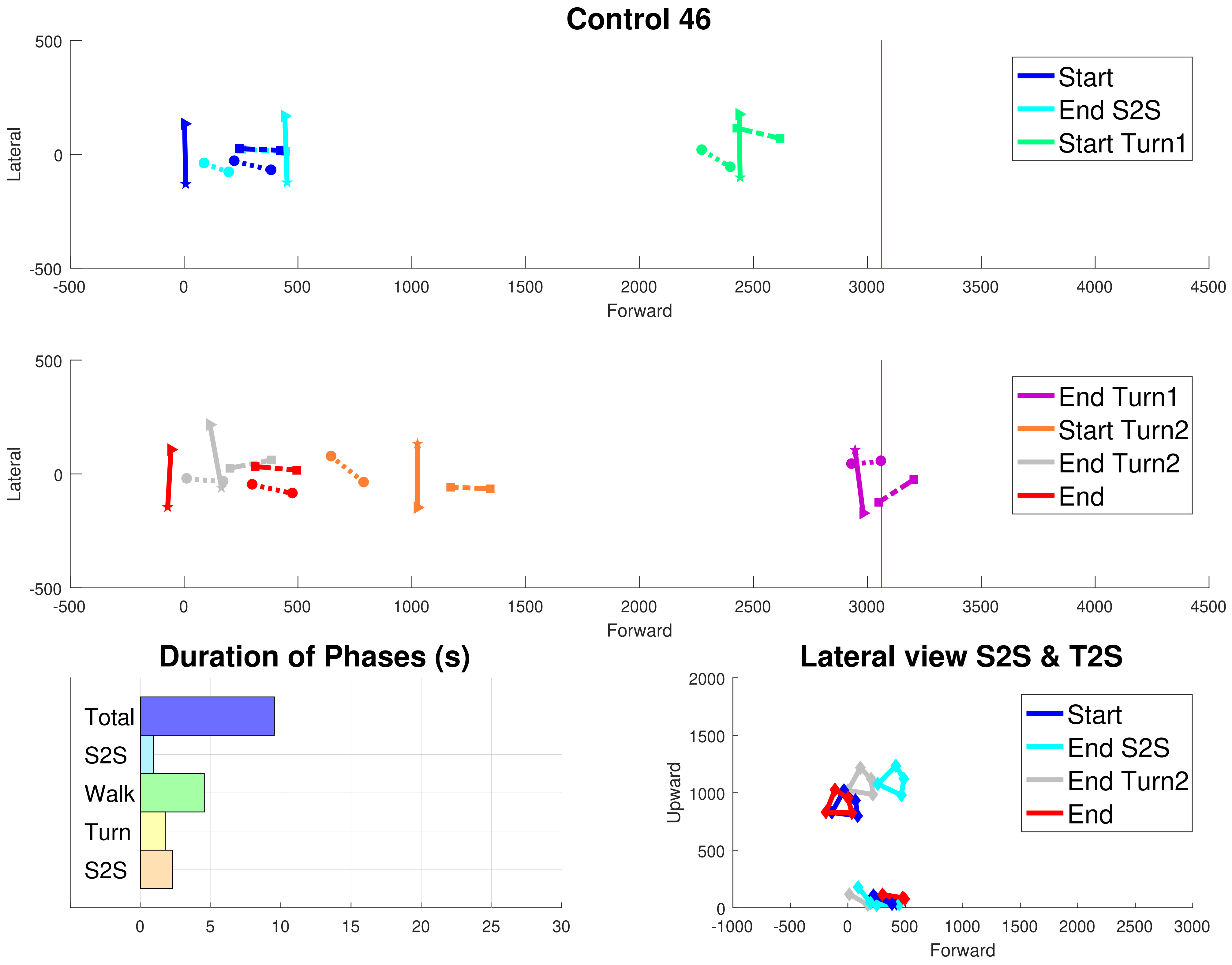

Control 46
Lateral
Forward
Start
End S2S
Start Turn1
End Turn1
Start Turn2
End Turn2
End
Duration of Phases (s)
Total
S2S
Walk
Turn
S2S
Lateral view S2S & T2S
Upward

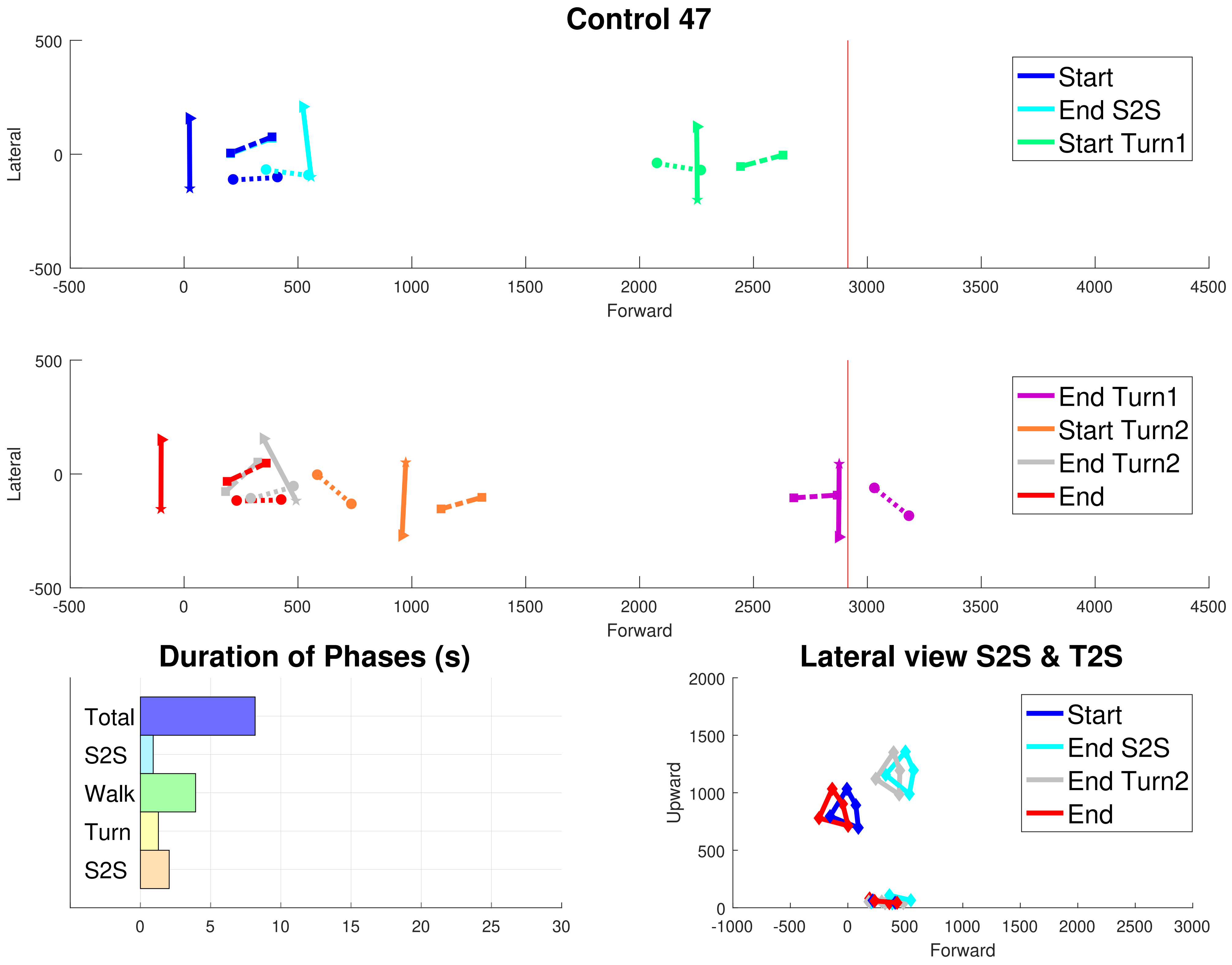

Control 47
Start
End S2S
Start Turn1
Lateral
Forward
End Turn1
Start Turn2
End Turn2
End
Duration of Phases (s)
Total
S2S
Walk
Turn
S2S
Lateral view S2S & T2S
Upward

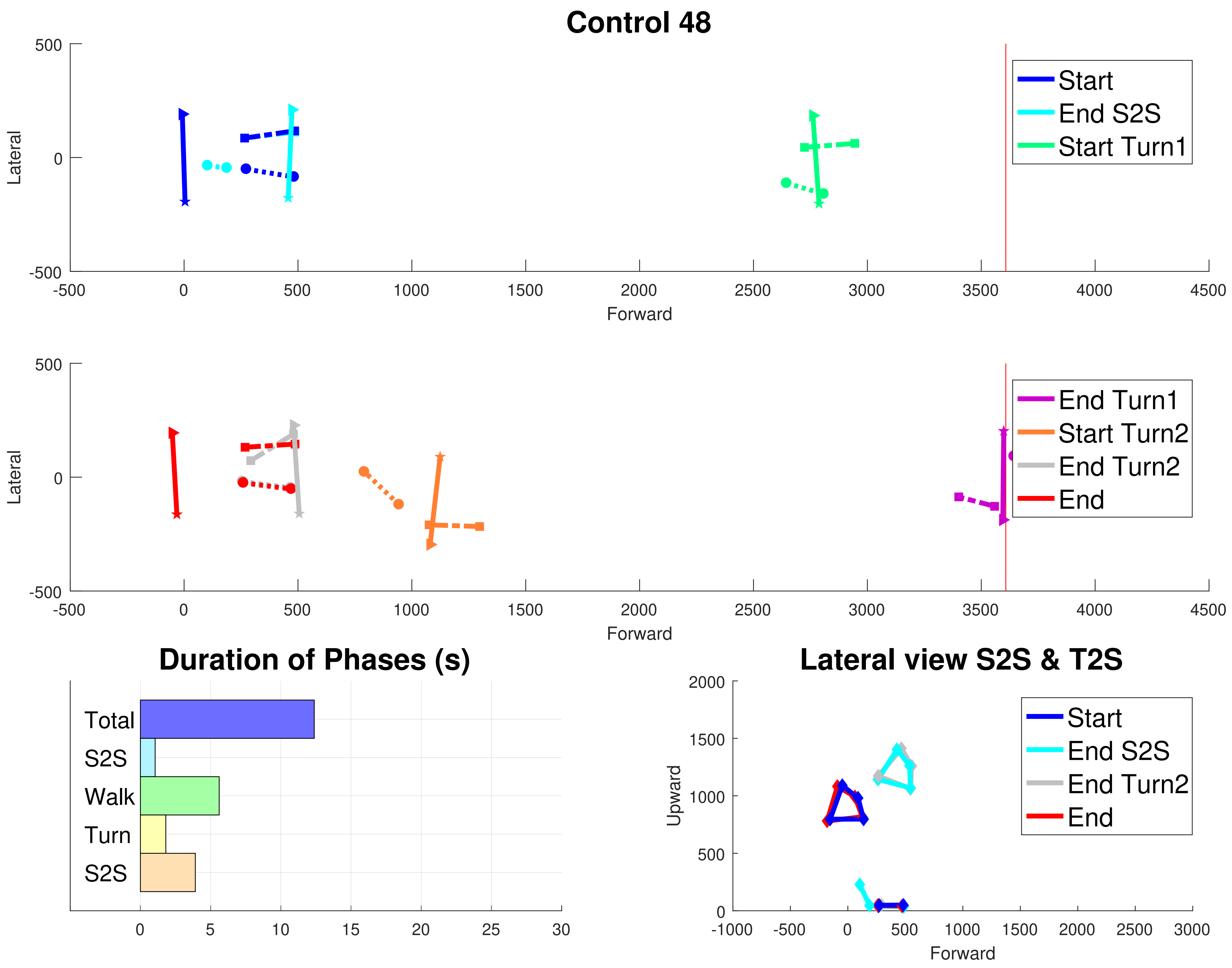

Control 48
Lateral
Forward
Start
End S2S
Start Turn1
End Turn1
Start Turn2
End Turn2
End
Duration of Phases (s)
Total
S2S
Walk
Turn
S2S
Lateral view S2S & T2S
Upward
Forward
Start
End S2S
End Turn2
End

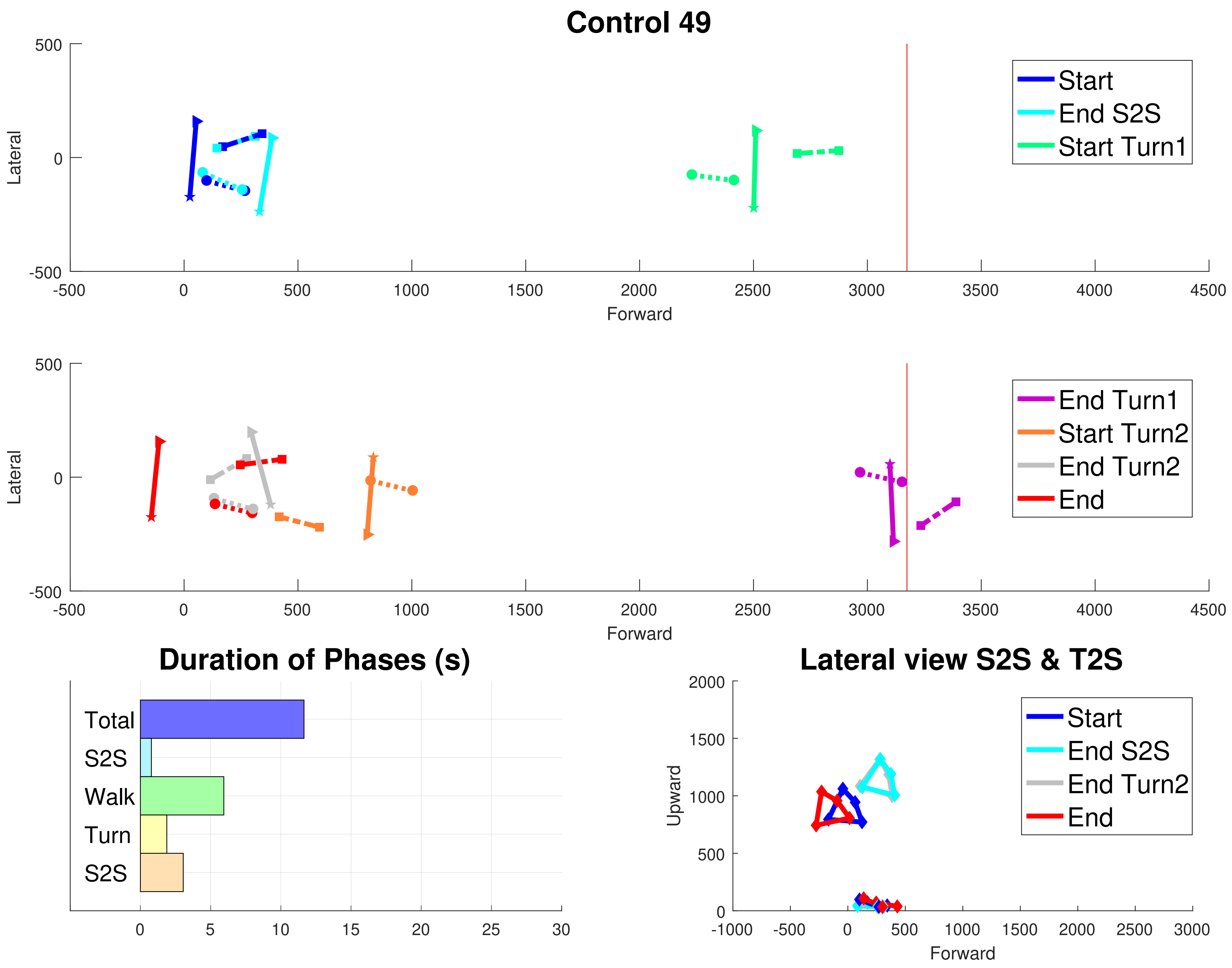
Control 49
Lateral
Forward
Start
End S2S
Start Turn1
End Turn1
Start Turn2
End Turn2
End
Duration of Phases (s)
Total
S2S
Walk
Turn
S2S
Lateral view S2S & T2S
Upward
Forward
Start
End S2S
End Turn2
End

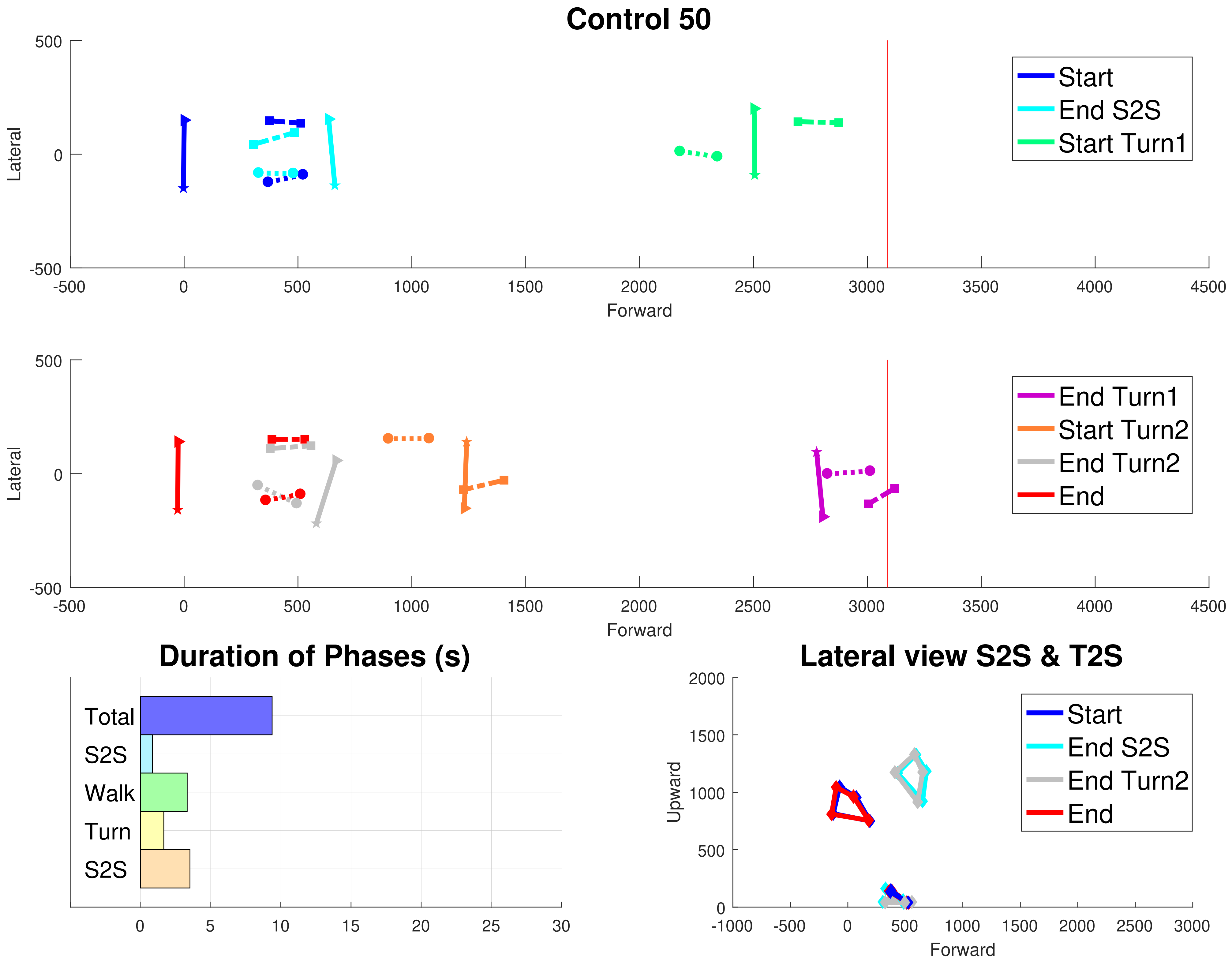

Control 50
Lateral
Forward
Start
End S2S
Start Turn1
End Turn1
Start Turn2
End Turn2
End
Duration of Phases (s)
Total
S2S
Walk
Turn
S2S
Lateral view S2S & T2S
Upward

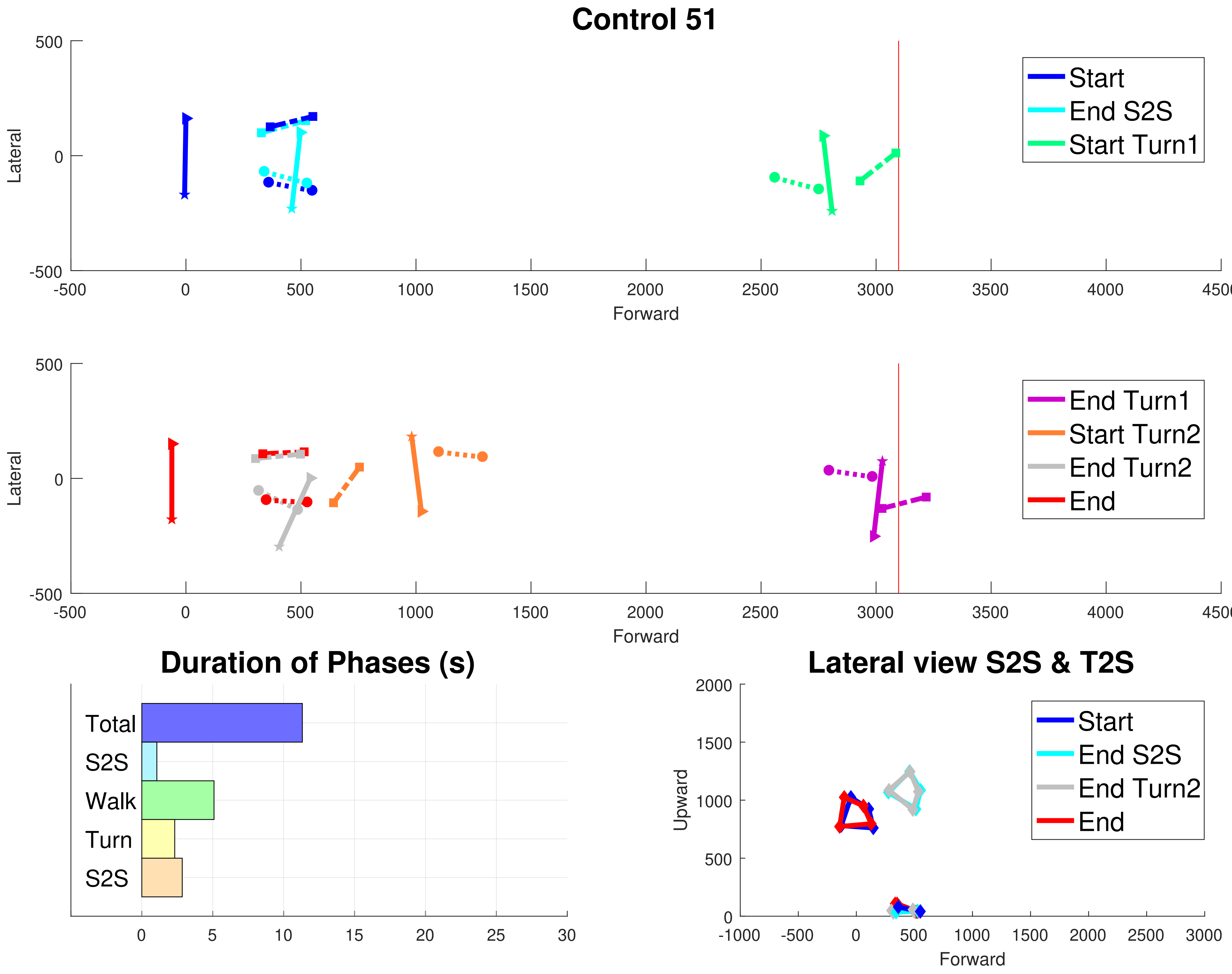

Control 51
Lateral
Forward
Start
End S2S
Start Turn1
End Turn1
Start Turn2
End Turn2
End
500
0
-500
-500
0
500
1000
1500
2000
2500
3000
3500
4000
4500
Duration of Phases (s)
Total
S2S
Walk
Turn
S2S
0
5
10
15
20
25
30
Lateral view S2S & T2S
Upward
2000
1500
1000
500
0
-1000
-500
0
500
1000
1500
2000
2500
3000

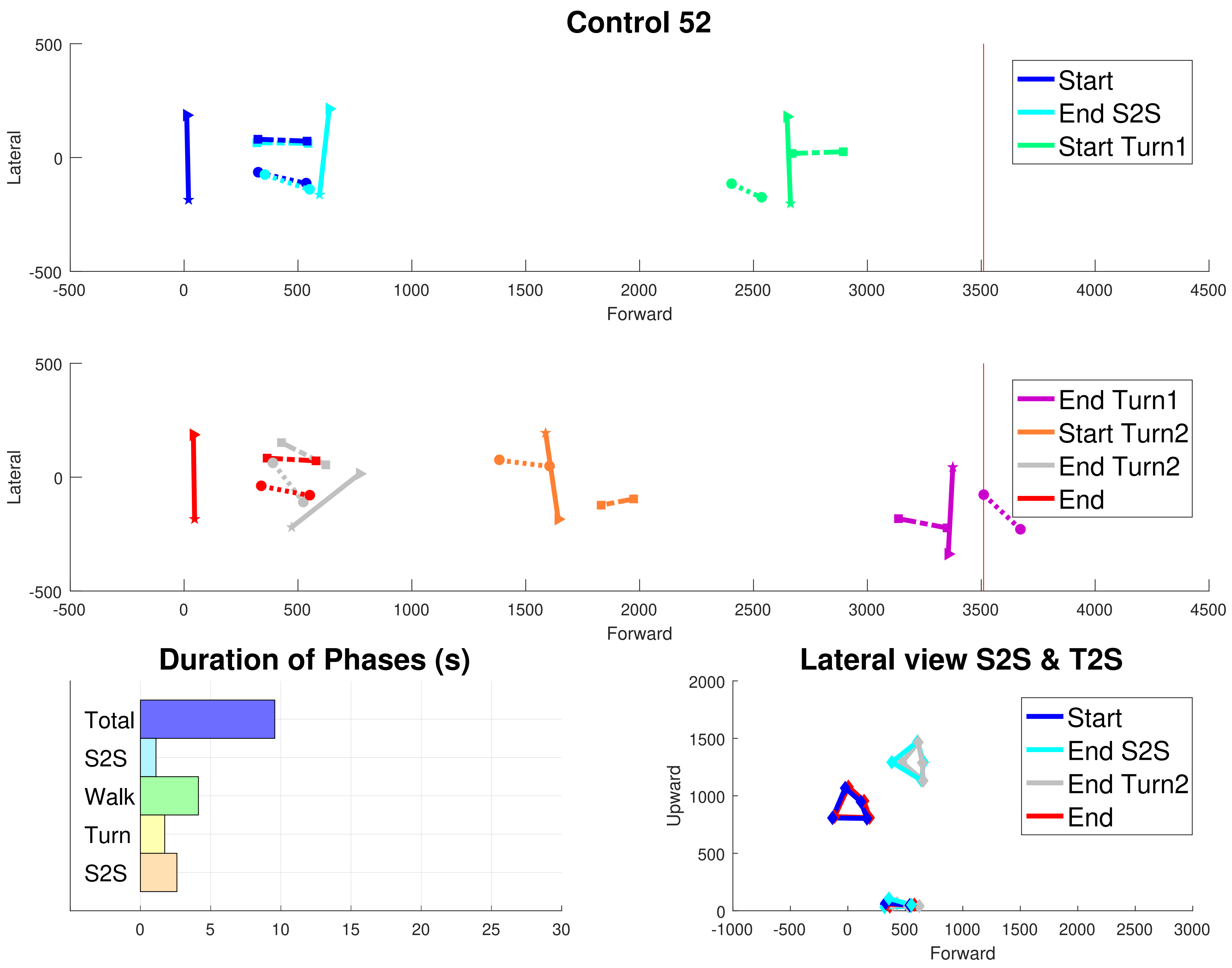
Control 52
Lateral
Forward
500
0
-500
-500
0
500
1000
1500
2000
2500
3000
3500
4000
4500
Start
End S2S
Start Turn1
End Turn1
Start Turn2
End Turn2
End
Duration of Phases (s)
Total
S2S
Walk
Turn
S2S
0
5
10
15
20
25
30
Lateral view S2S & T2S
Upward
2000
1500
1000
500
-1000
-500
3000
