# Supplementary material for: Which functional tasks present the largest deficits for patients with total hip arthroplasty before and six months after surgery? A study of the timed up-and-go test phases
Source: PLoS One. 2021 Sep 10;16(9):e0255037. doi: 10.1371/journal.pone.0255037 (PMC8432811; doi:10.1371/journal.pone.0255037)
Supplement: S3 Fig — (PDF) [file pone.0255037.s003.pdf]

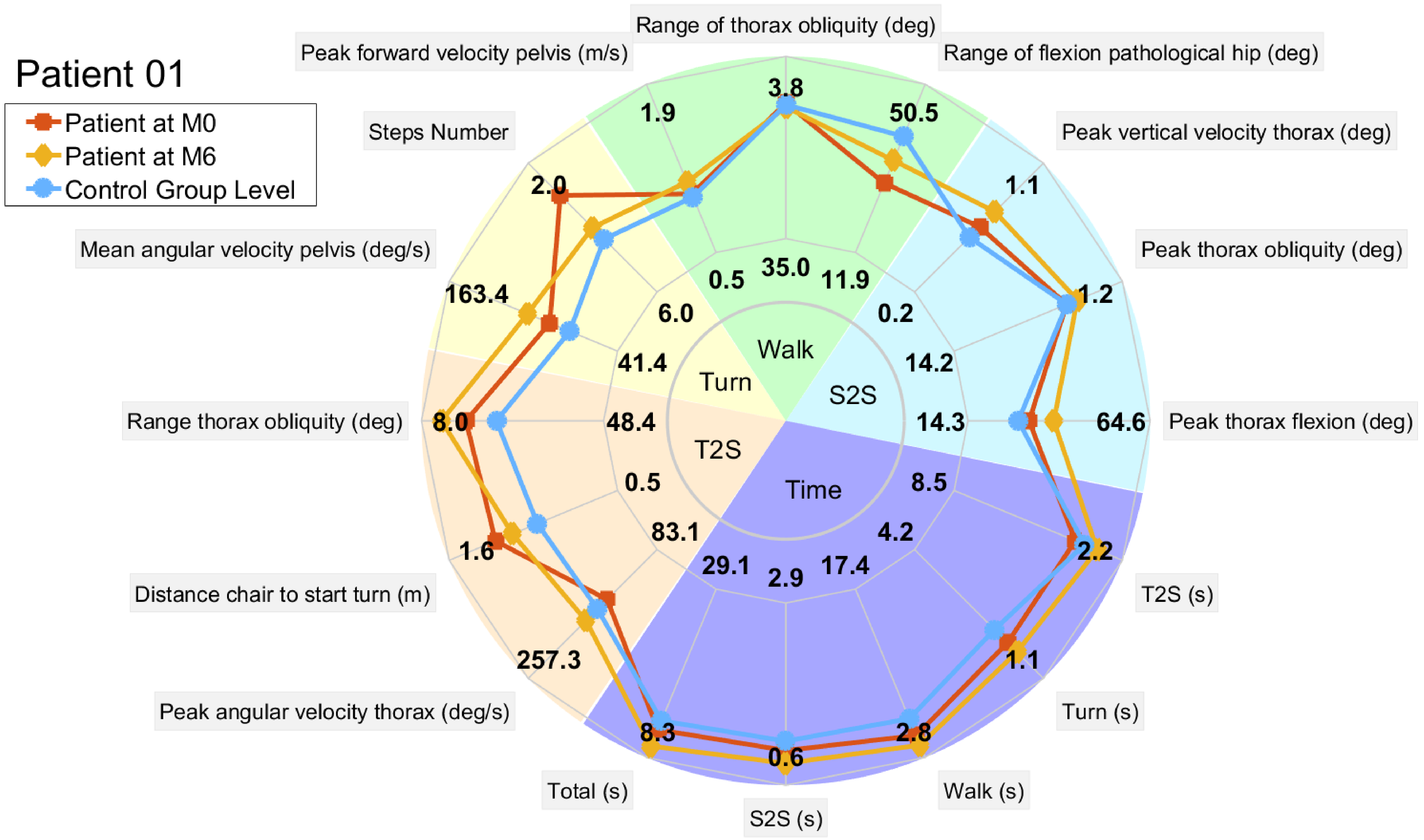

Patient 01
Patient at M0
Patient at M6
Control Group Level
Range of thorax obliquity (deg)
Range of flexion pathological hip (deg)
Peak vertical velocity thorax (deg)
Peak thorax obliquity (deg)
Peak thorax flexion (deg)
T2S (s)
Turn (s)
Walk (s)
S2S (s)
Total (s)
Peak angular velocity thorax (deg/s)
Distance chair to start turn (m)
Range thorax obliquity (deg)
Mean angular velocity pelvis (deg/s)
Steps Number
Peak forward velocity pelvis (m/s)
Walk
S2S
Time
T2S
Turn
3.8
50.5
1.1
1.2
64.6
2.2
1.1
2.8
0.6
8.3
257.3
1.6
8.0
163.4
2.0
1.9
35.0
11.9
0.2
14.2
14.3
8.5
4.2
17.4
2.9
29.1
83.1
0.5
48.4
41.4
6.0
0.5

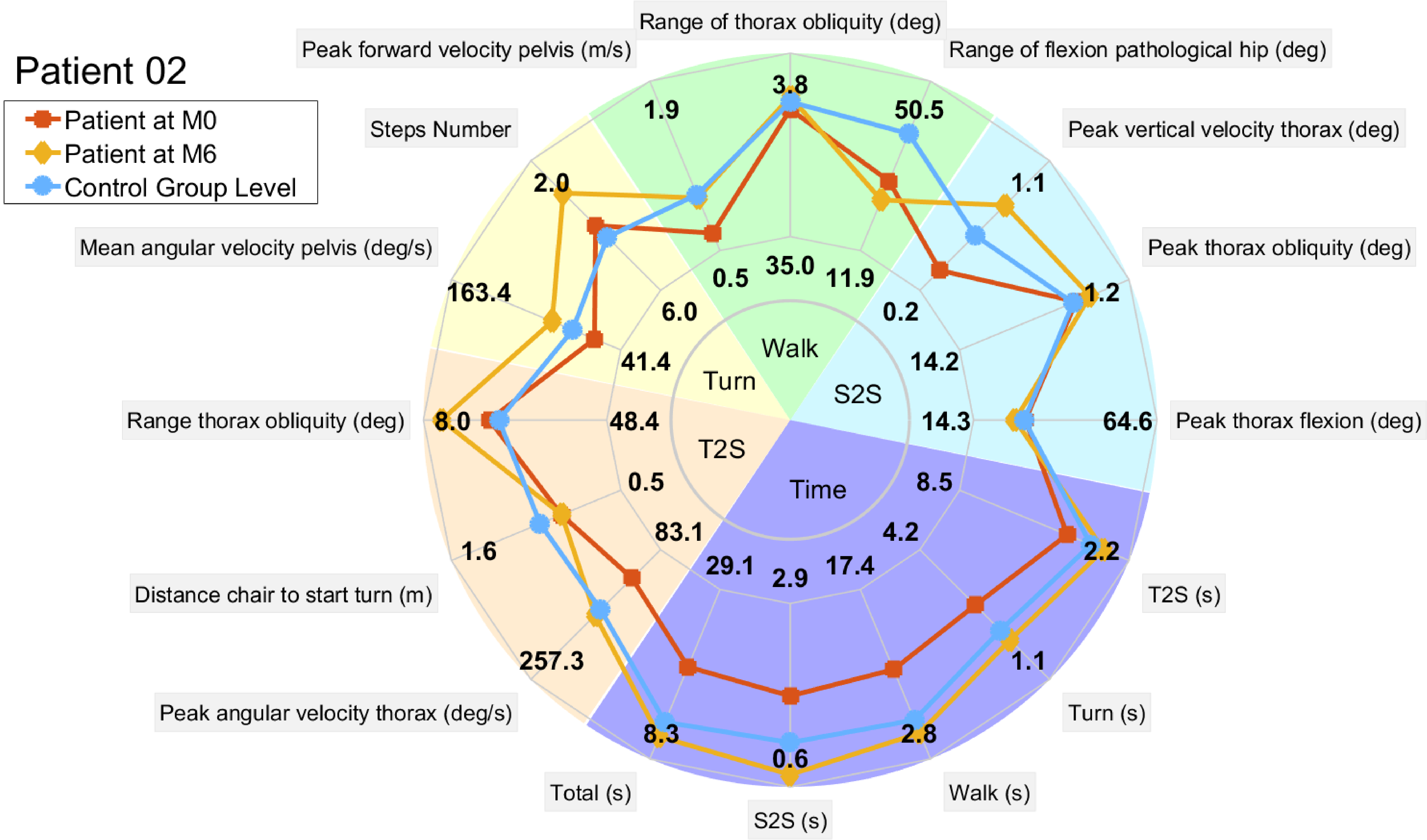

Patient 02
Patient at M0
Patient at M6
Control Group Level
Range of thorax obliquity (deg)
Range of flexion pathological hip (deg)
Peak vertical velocity thorax (deg)
Peak thorax obliquity (deg)
Peak thorax flexion (deg)
T2S (s)
Turn (s)
Walk (s)
S2S (s)
Total (s)
Peak angular velocity thorax (deg/s)
Distance chair to start turn (m)
Range thorax obliquity (deg)
Mean angular velocity pelvis (deg/s)
Steps Number
Peak forward velocity pelvis (m/s)
Walk
S2S
Time
T2S
Turn
3.8
50.5
1.1
1.2
64.6
2.2
1.1
2.8
0.6
8.3
257.3
1.6
8.0
163.4
2.0
1.9
35.0
11.9
0.2
14.2
14.3
8.5
4.2
17.4
2.9
29.1
83.1
0.5
48.4
41.4
6.0
0.5

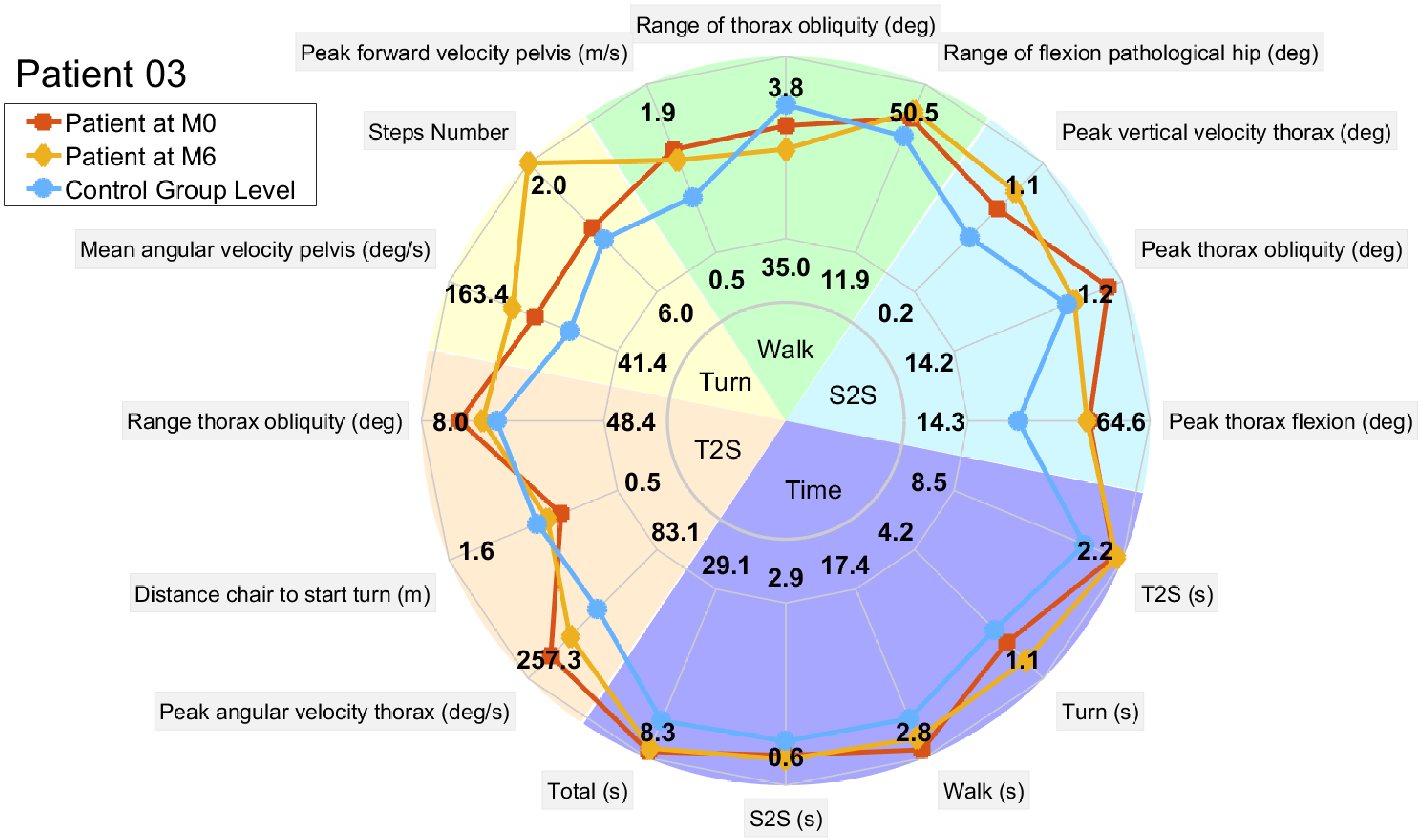

Patient 03
Patient at M0
Patient at M6
Control Group Level
Range of thorax obliquity (deg)
Peak forward velocity pelvis (m/s)
Range of flexion pathological hip (deg)
Steps Number
Peak vertical velocity thorax (deg)
Mean angular velocity pelvis (deg/s)
Peak thorax obliquity (deg)
Range thorax obliquity (deg)
Peak thorax flexion (deg)
Distance chair to start turn (m)
T2S (s)
Peak angular velocity thorax (deg/s)
Turn (s)
Total (s)
S2S (s)
Walk (s)
Walk
Turn
S2S
T2S
Time
3.8
1.9
50.5
2.0
1.1
163.4
1.2
8.0
64.6
1.6
2.2
257.3
1.1
8.3
0.6
2.8
35.0
0.5
11.9
6.0
0.2
41.4
14.2
48.4
14.3
0.5
8.5
83.1
4.2
29.1
2.9
17.4

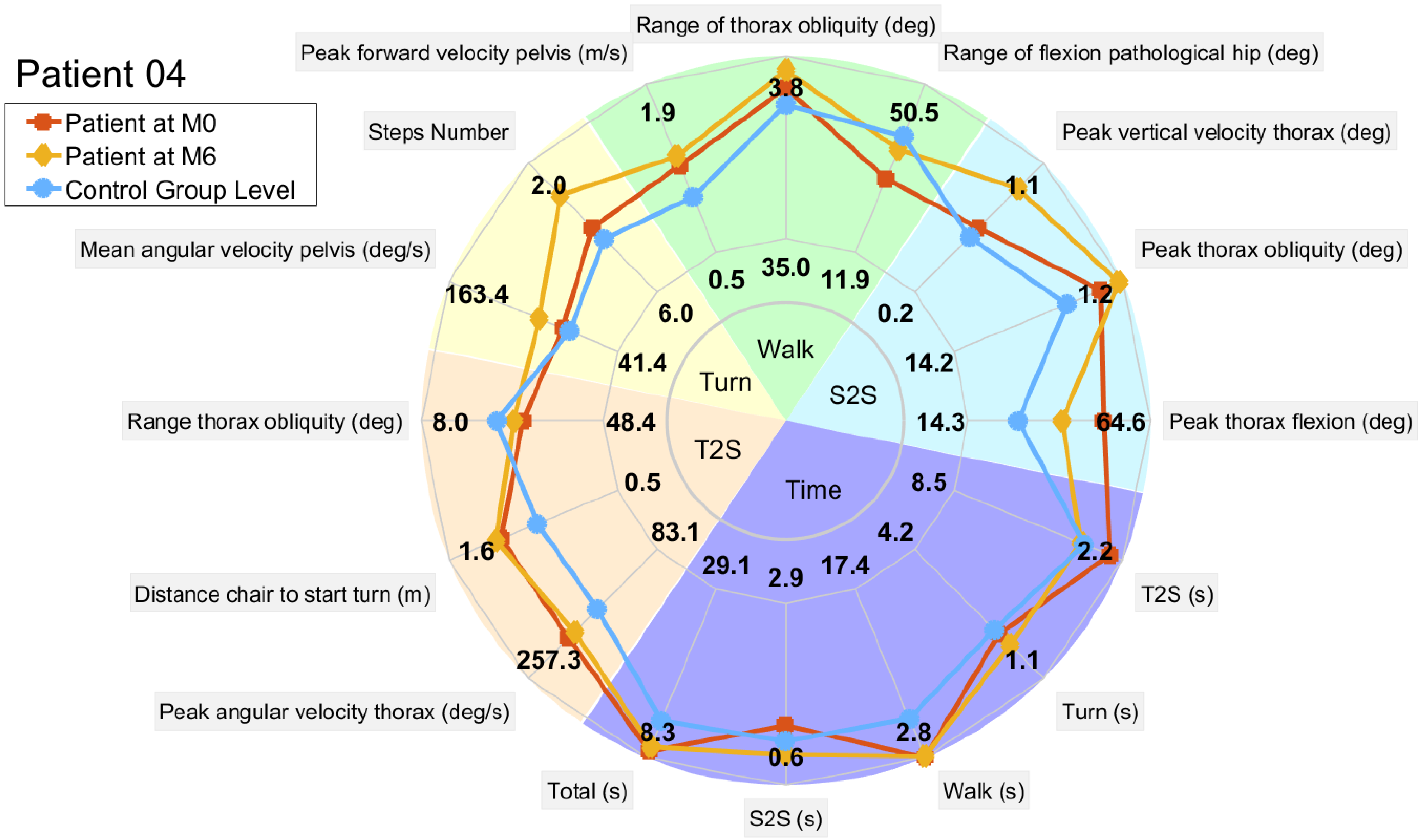

Patient 04
Patient at M0
Patient at M6
Control Group Level
Range of thorax obliquity (deg)
Range of flexion pathological hip (deg)
Peak vertical velocity thorax (deg)
Peak thorax obliquity (deg)
Peak thorax flexion (deg)
T2S (s)
Turn (s)
Walk (s)
S2S (s)
Total (s)
Peak angular velocity thorax (deg/s)
Distance chair to start turn (m)
Range thorax obliquity (deg)
Mean angular velocity pelvis (deg/s)
Steps Number
Peak forward velocity pelvis (m/s)
Walk
S2S
Time
T2S
Turn
3.8
50.5
1.1
1.2
64.6
2.2
1.1
2.8
0.6
8.3
257.3
1.6
8.0
163.4
2.0
1.9
35.0
11.9
0.2
14.2
14.3
8.5
4.2
17.4
2.9
29.1
83.1
0.5
48.4
41.4
6.0
0.5

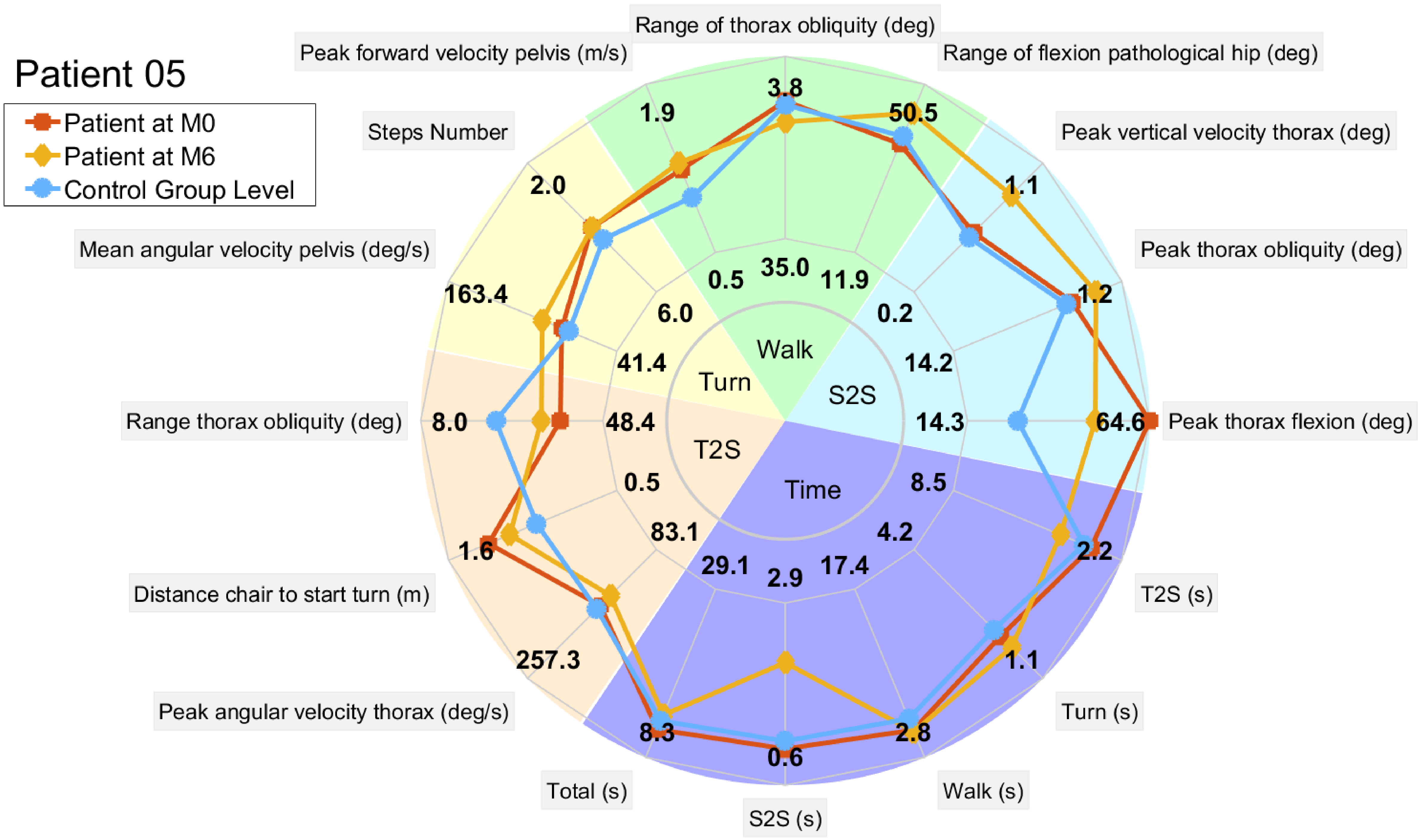
Patient 05
Patient at M0
Patient at M6
Control Group Level
Range of thorax obliquity (deg)
Peak forward velocity pelvis (m/s)
Range of flexion pathological hip (deg)
Steps Number
Peak vertical velocity thorax (deg)
Mean angular velocity pelvis (deg/s)
Peak thorax obliquity (deg)
Range thorax obliquity (deg)
Peak thorax flexion (deg)
Distance chair to start turn (m)
T2S (s)
Peak angular velocity thorax (deg/s)
Turn (s)
Total (s)
Walk (s)
S2S (s)
Walk
Turn
S2S
T2S
Time
3.8
1.9
50.5
2.0
1.1
163.4
1.2
8.0
64.6
1.6
2.2
257.3
1.1
8.3
0.6
2.8
0.5
35.0
11.9
6.0
0.2
41.4
14.2
48.4
14.3
0.5
8.5
83.1
4.2
29.1
2.9
17.4

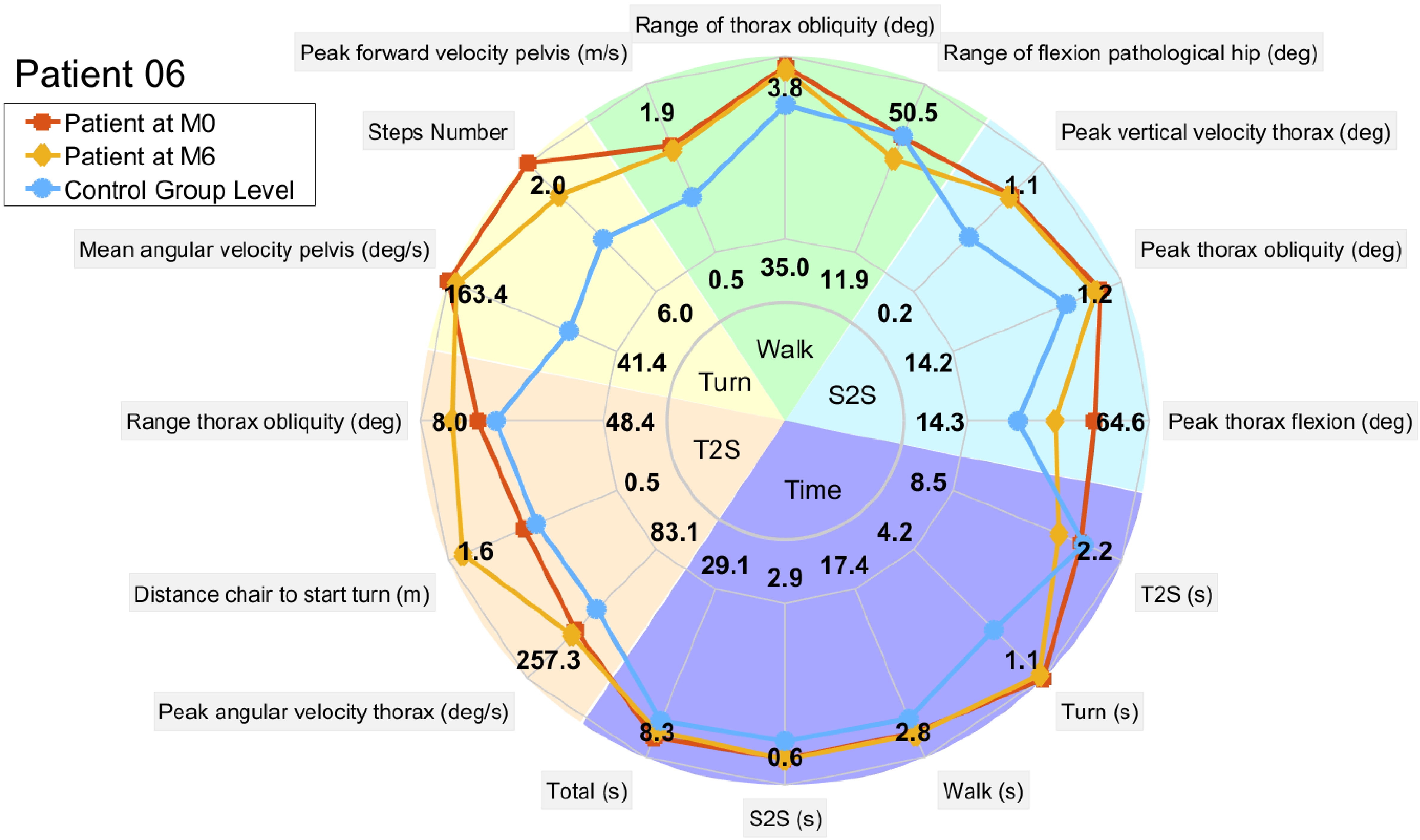

Patient 06
Patient at M0
Patient at M6
Control Group Level
Range of thorax obliquity (deg)
Peak forward velocity pelvis (m/s)
Range of flexion pathological hip (deg)
Steps Number
Peak vertical velocity thorax (deg)
Mean angular velocity pelvis (deg/s)
Peak thorax obliquity (deg)
Range thorax obliquity (deg)
Peak thorax flexion (deg)
Distance chair to start turn (m)
T2S (s)
Peak angular velocity thorax (deg/s)
Turn (s)
Total (s)
S2S (s)
Walk (s)
Walk
Turn
S2S
T2S
Time
3.8
1.9
50.5
2.0
1.1
163.4
1.2
8.0
64.6
1.6
2.2
257.3
1.1
8.3
0.6
2.8
0.5
35.0
11.9
6.0
0.2
41.4
14.2
48.4
14.3
0.5
8.5
83.1
4.2
29.1
2.9
17.4

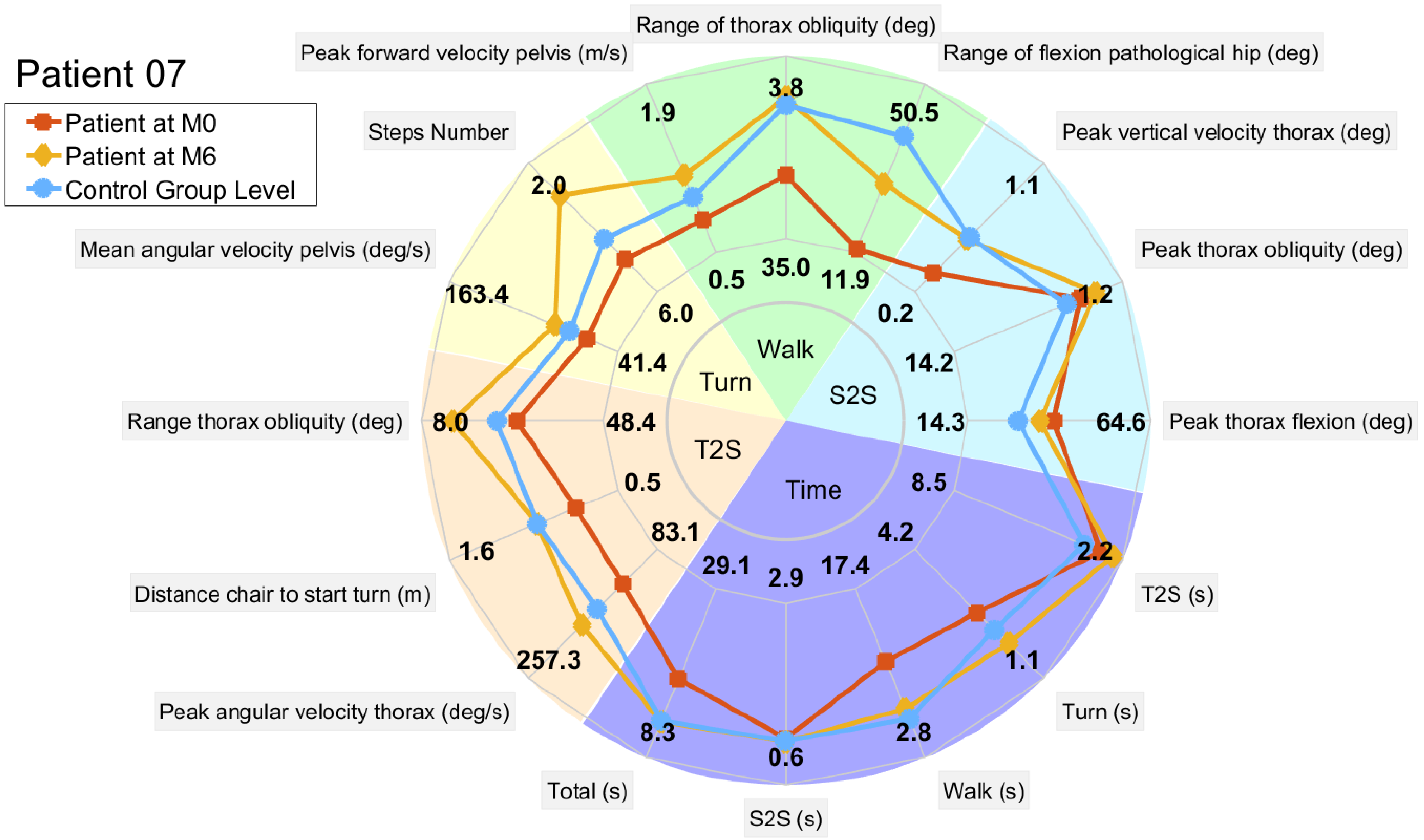

Patient 07
Patient at M0
Patient at M6
Control Group Level
Range of thorax obliquity (deg)
Range of flexion pathological hip (deg)
Peak vertical velocity thorax (deg)
Peak thorax obliquity (deg)
Peak thorax flexion (deg)
T2S (s)
Turn (s)
Walk (s)
S2S (s)
Total (s)
Peak angular velocity thorax (deg/s)
Distance chair to start turn (m)
Range thorax obliquity (deg)
Mean angular velocity pelvis (deg/s)
Steps Number
Peak forward velocity pelvis (m/s)
Walk
S2S
Time
T2S
Turn
3.8
50.5
1.1
1.2
64.6
2.2
1.1
2.8
0.6
8.3
257.3
1.6
8.0
163.4
2.0
1.9
35.0
11.9
0.2
14.2
14.3
8.5
4.2
17.4
2.9
29.1
83.1
0.5
48.4
41.4
6.0
0.5

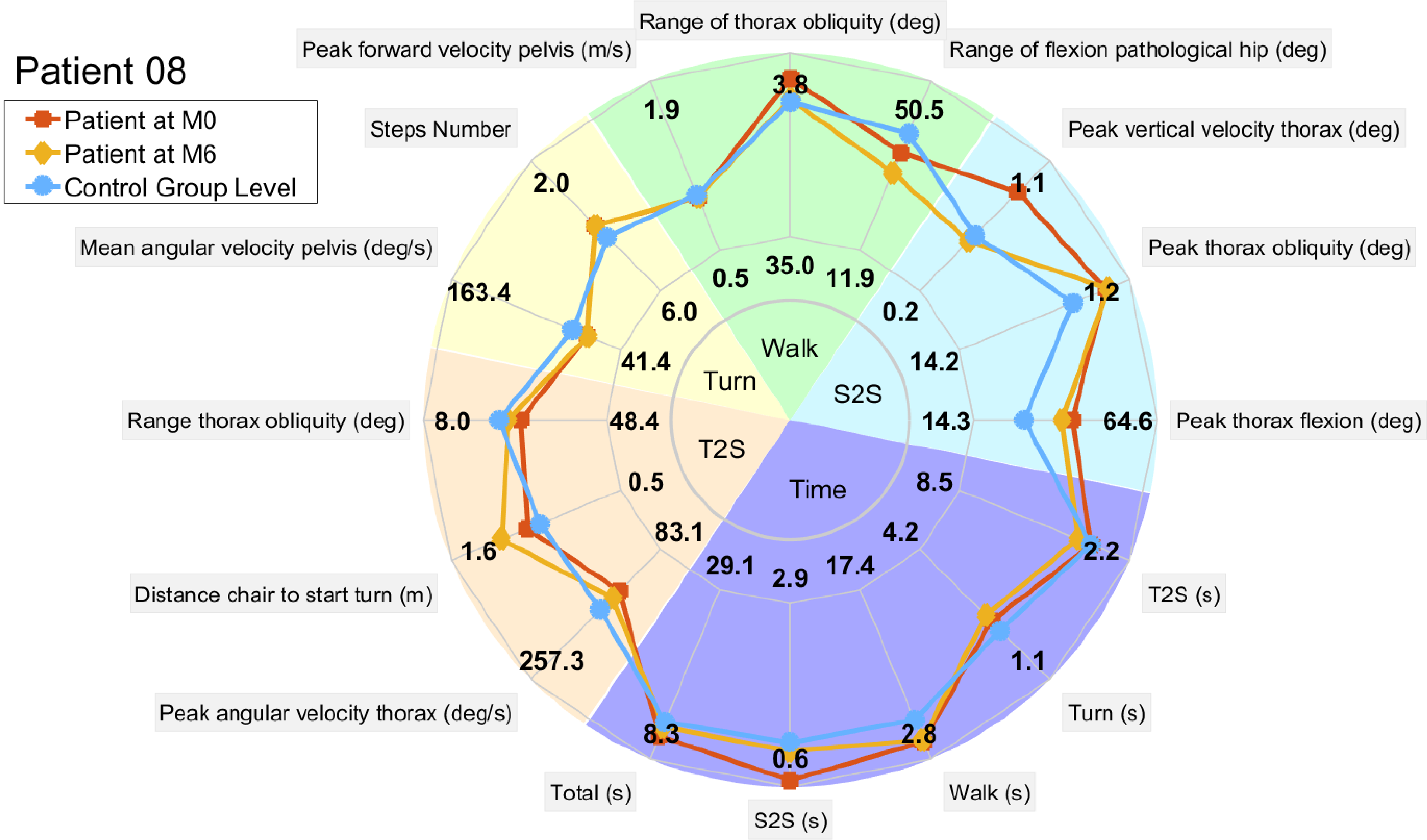
Patient 08
Patient at M0
Patient at M6
Control Group Level
Range of thorax obliquity (deg)
Range of flexion pathological hip (deg)
Peak vertical velocity thorax (deg)
Peak thorax obliquity (deg)
Peak thorax flexion (deg)
T2S (s)
Turn (s)
Walk (s)
S2S (s)
Total (s)
Peak angular velocity thorax (deg/s)
Distance chair to start turn (m)
Range thorax obliquity (deg)
Mean angular velocity pelvis (deg/s)
Steps Number
Peak forward velocity pelvis (m/s)
Walk
Turn
S2S
T2S
Time
3.8
50.5
1.1
1.2
64.6
2.2
1.1
2.8
0.6
8.3
257.3
1.6
8.0
163.4
2.0
1.9
35.0
11.9
0.2
14.2
14.3
8.5
4.2
17.4
2.9
29.1
83.1
0.5
48.4
41.4
6.0
0.5

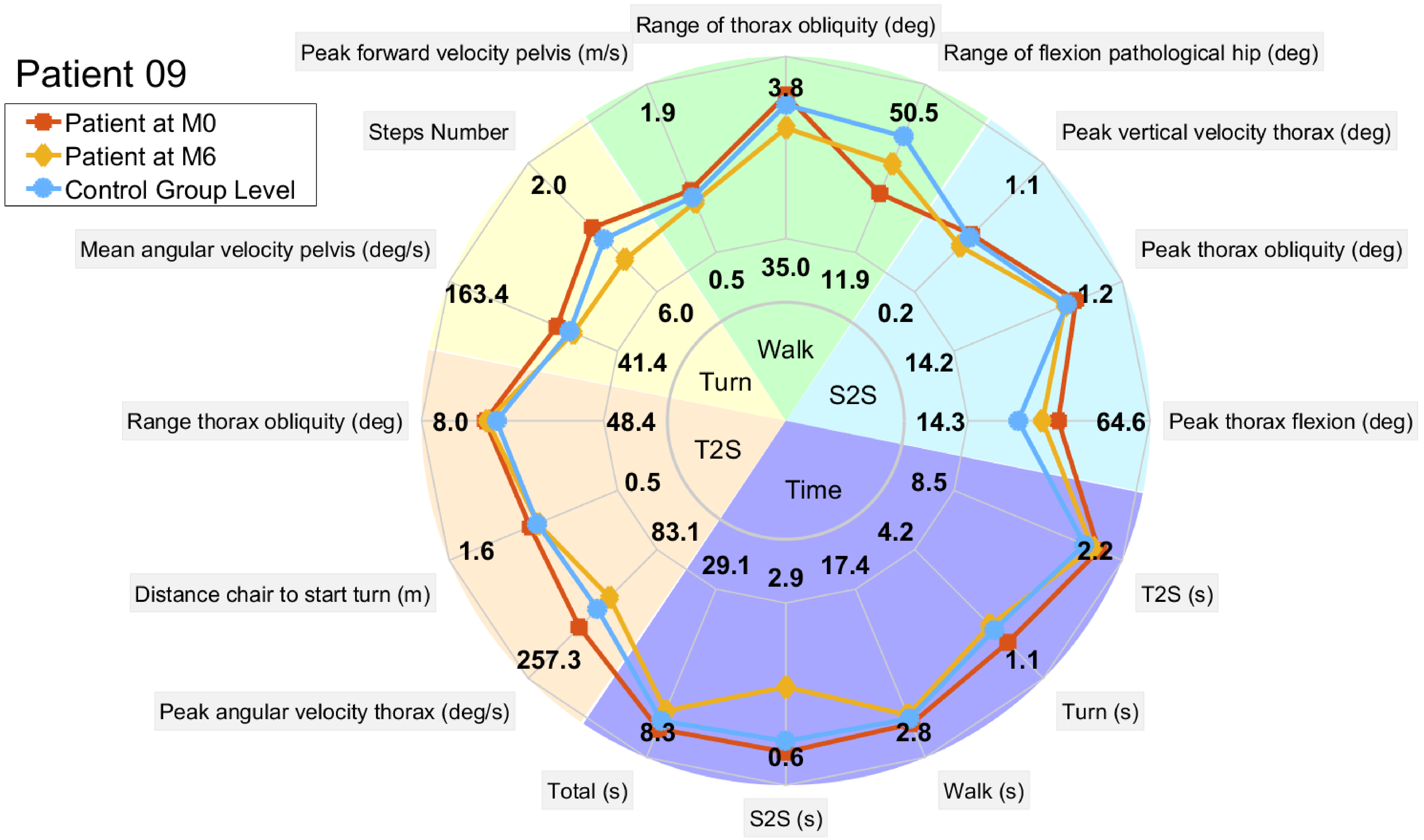

Patient 09
Patient at M0
Patient at M6
Control Group Level
Range of thorax obliquity (deg)
Range of flexion pathological hip (deg)
Peak vertical velocity thorax (deg)
Peak thorax obliquity (deg)
Peak thorax flexion (deg)
T2S (s)
Turn (s)
Walk (s)
S2S (s)
Total (s)
Peak angular velocity thorax (deg/s)
Distance chair to start turn (m)
Range thorax obliquity (deg)
Mean angular velocity pelvis (deg/s)
Steps Number
Peak forward velocity pelvis (m/s)
Walk
Turn
S2S
T2S
Time
3.8
50.5
1.1
1.2
64.6
2.2
1.1
2.8
0.6
8.3
257.3
1.6
8.0
163.4
2.0
1.9
0.5
35.0
11.9
0.2
14.2
14.3
8.5
4.2
17.4
2.9
29.1
83.1
0.5
48.4
41.4
6.0

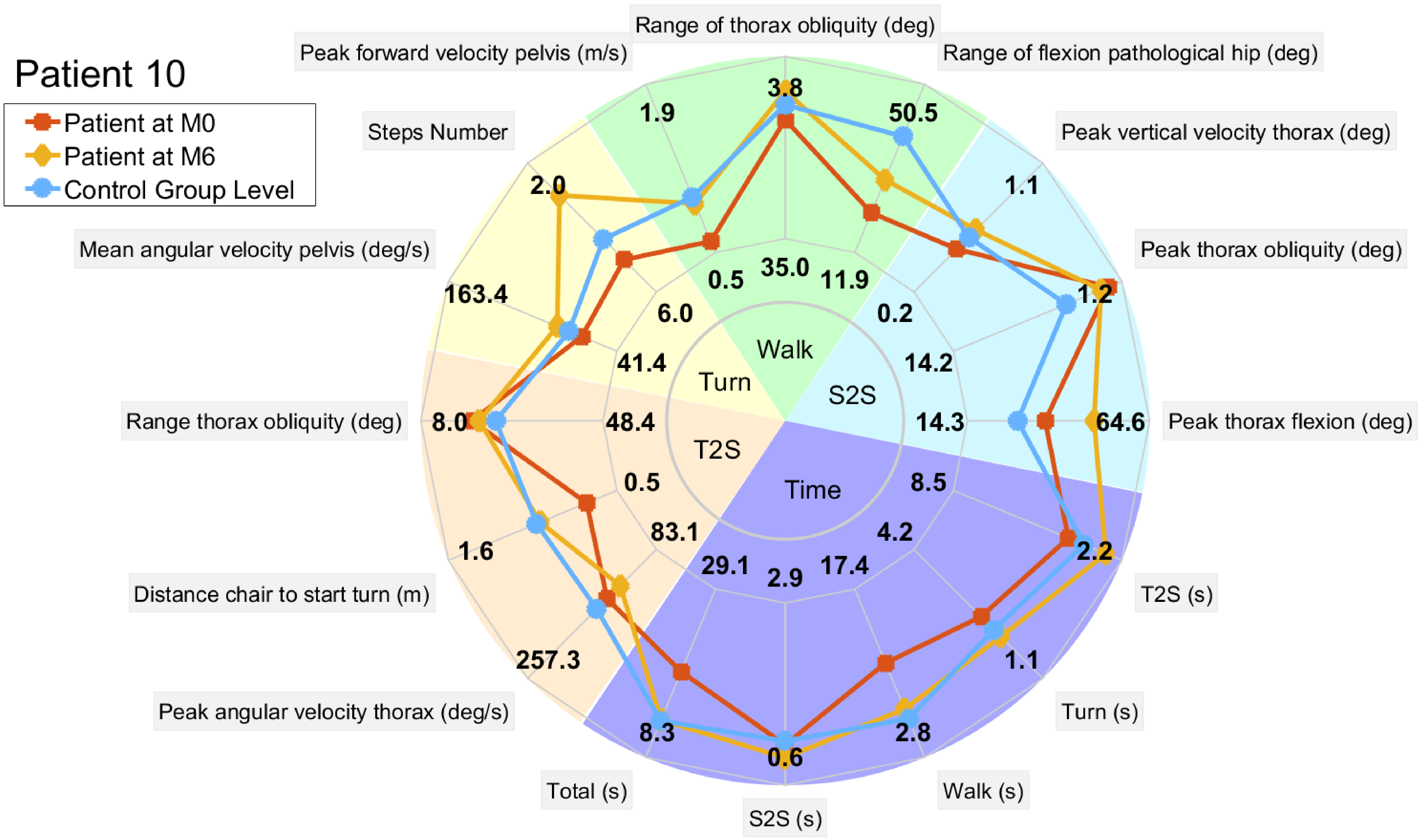

Patient 10
Patient at M0
Patient at M6
Control Group Level
Range of thorax obliquity (deg)
Peak forward velocity pelvis (m/s)
Range of flexion pathological hip (deg)
Steps Number
Peak vertical velocity thorax (deg)
Mean angular velocity pelvis (deg/s)
Peak thorax obliquity (deg)
Range thorax obliquity (deg)
Peak thorax flexion (deg)
Distance chair to start turn (m)
T2S (s)
Peak angular velocity thorax (deg/s)
Turn (s)
Total (s)
Walk (s)
S2S (s)
Walk
Turn
S2S
T2S
Time
3.8
1.9
50.5
2.0
1.1
163.4
1.2
8.0
64.6
1.6
2.2
257.3
1.1
8.3
2.8
0.6
0.5
35.0
11.9
6.0
0.2
41.4
14.2
48.4
14.3
0.5
8.5
83.1
4.2
29.1
2.9
17.4

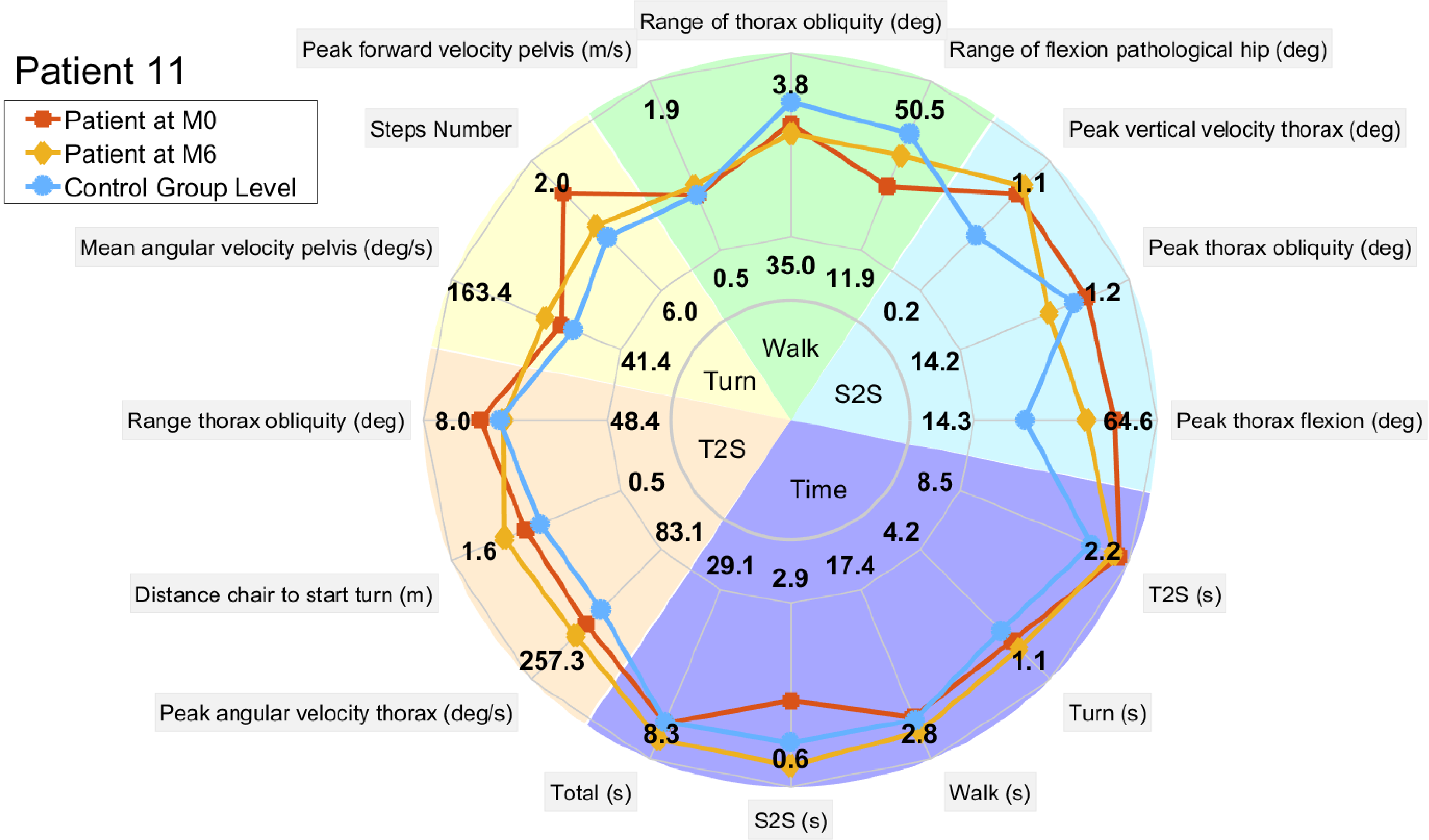
Patient 11
Patient at M0
Patient at M6
Control Group Level
Range of thorax obliquity (deg)
Peak forward velocity pelvis (m/s)
Range of flexion pathological hip (deg)
Steps Number
Peak vertical velocity thorax (deg)
Mean angular velocity pelvis (deg/s)
Peak thorax obliquity (deg)
Range thorax obliquity (deg)
Peak thorax flexion (deg)
Distance chair to start turn (m)
T2S (s)
Peak angular velocity thorax (deg/s)
Turn (s)
Total (s)
Walk (s)
S2S (s)
Walk
Turn
S2S
T2S
Time
3.8
1.9
50.5
2.0
1.1
163.4
1.2
8.0
64.6
1.6
2.2
257.3
1.1
8.3
2.8
0.6
35.0
0.5
11.9
6.0
0.2
41.4
14.2
48.4
14.3
0.5
8.5
83.1
4.2
29.1
2.9
17.4

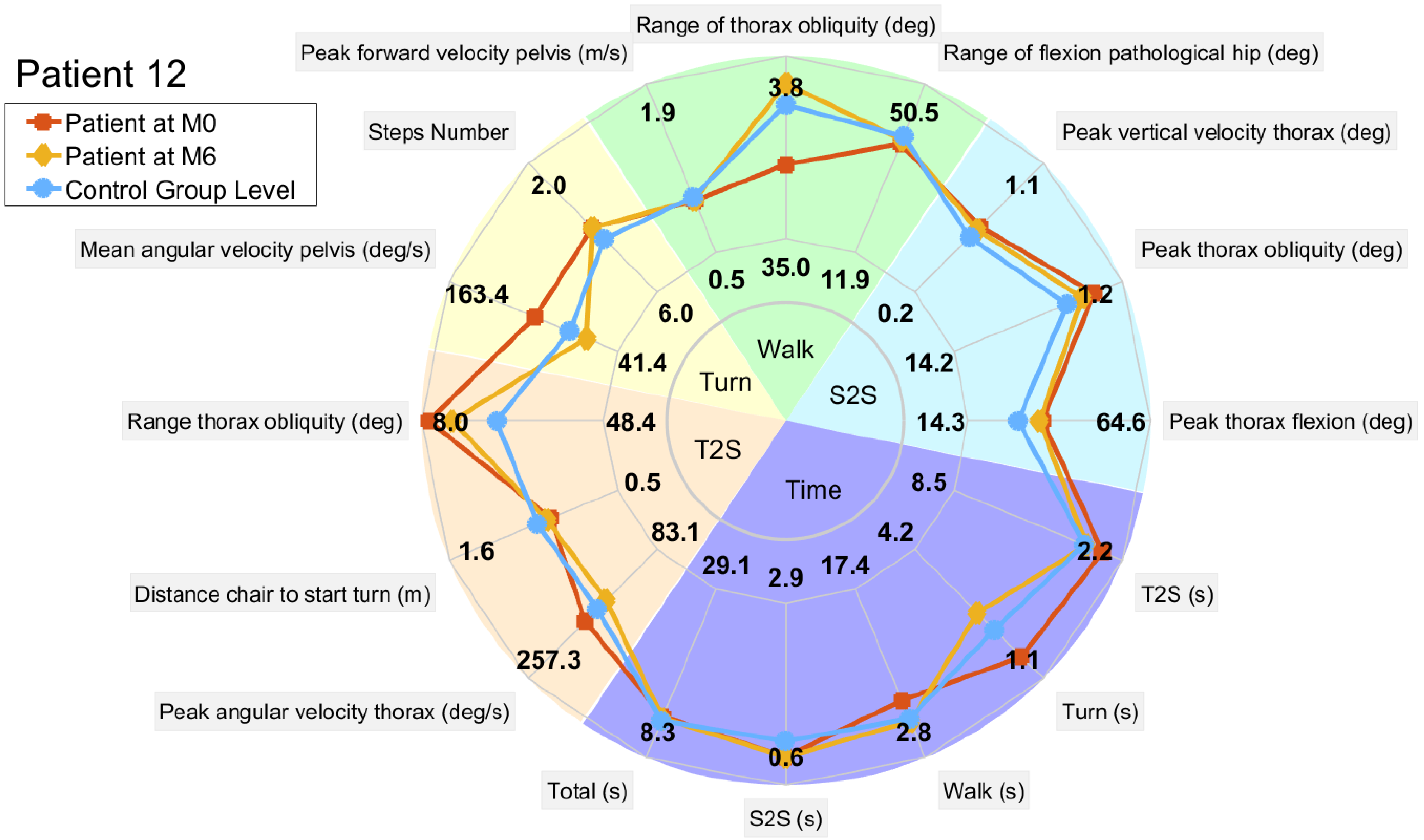

Patient 12
Patient at M0
Patient at M6
Control Group Level
Range of thorax obliquity (deg)
Range of flexion pathological hip (deg)
Peak vertical velocity thorax (deg)
Peak thorax obliquity (deg)
Peak thorax flexion (deg)
T2S (s)
Turn (s)
Walk (s)
S2S (s)
Total (s)
Peak angular velocity thorax (deg/s)
Distance chair to start turn (m)
Range thorax obliquity (deg)
Mean angular velocity pelvis (deg/s)
Steps Number
Peak forward velocity pelvis (m/s)
Walk
S2S
Time
T2S
Turn
3.8
50.5
1.1
1.2
64.6
2.2
1.1
2.8
0.6
8.3
257.3
1.6
8.0
163.4
2.0
1.9
0.5
35.0
11.9
0.2
14.2
14.3
8.5
4.2
17.4
2.9
29.1
83.1
0.5
48.4
41.4
6.0

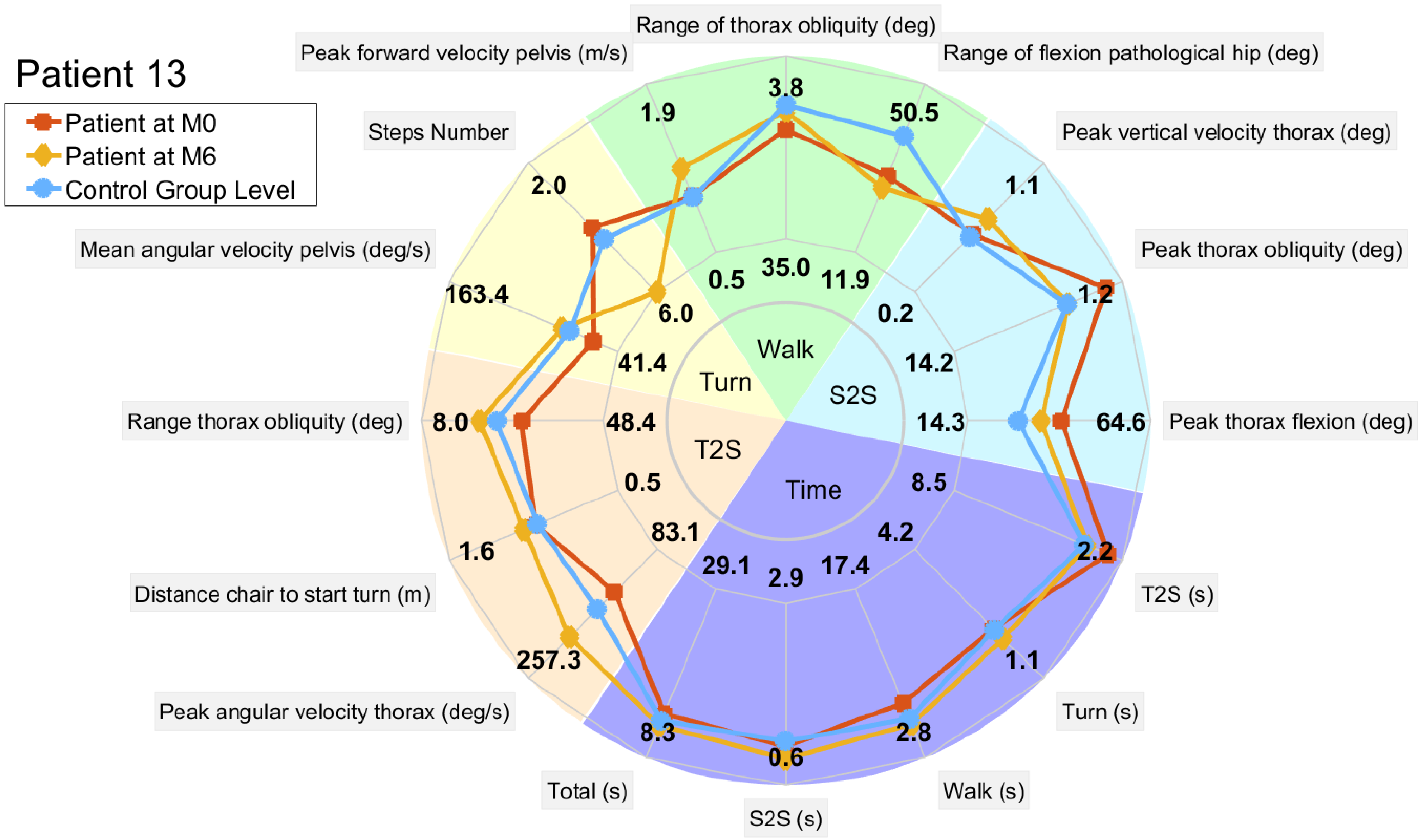

Patient 13
Patient at M0
Patient at M6
Control Group Level
Range of thorax obliquity (deg)
Range of flexion pathological hip (deg)
Peak vertical velocity thorax (deg)
Peak thorax obliquity (deg)
Peak thorax flexion (deg)
T2S (s)
Turn (s)
Walk (s)
S2S (s)
Total (s)
Peak angular velocity thorax (deg/s)
Distance chair to start turn (m)
Range thorax obliquity (deg)
Mean angular velocity pelvis (deg/s)
Steps Number
Peak forward velocity pelvis (m/s)
Walk
S2S
Time
T2S
Turn
3.8
50.5
1.1
1.2
64.6
2.2
1.1
2.8
0.6
8.3
257.3
1.6
8.0
163.4
2.0
1.9
35.0
11.9
0.2
14.2
14.3
8.5
4.2
17.4
2.9
29.1
83.1
0.5
48.4
41.4
6.0
0.5

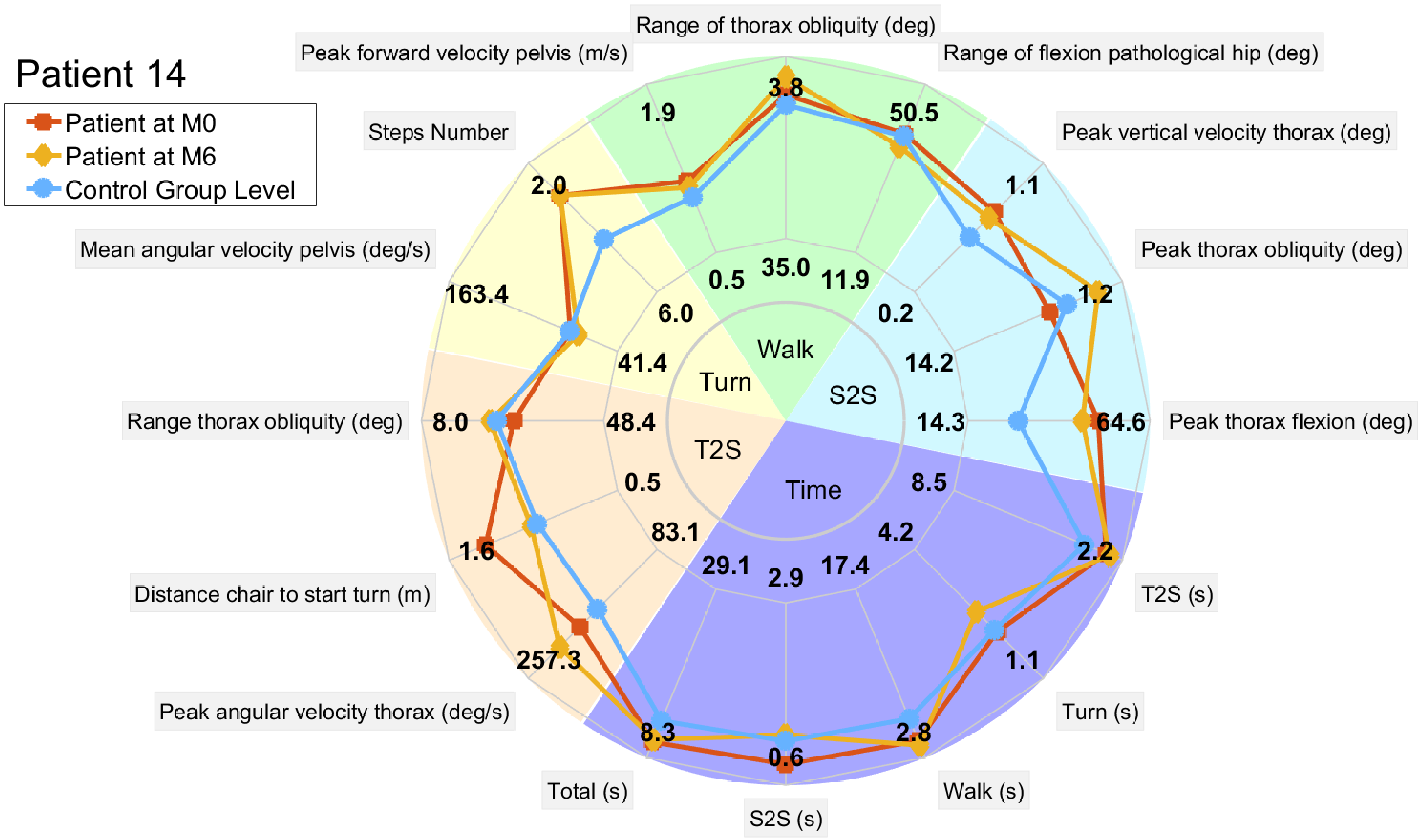

Patient 14
Patient at M0
Patient at M6
Control Group Level
Range of thorax obliquity (deg)
Range of flexion pathological hip (deg)
Peak vertical velocity thorax (deg)
Peak thorax obliquity (deg)
Peak thorax flexion (deg)
T2S (s)
Turn (s)
Walk (s)
S2S (s)
Total (s)
Peak angular velocity thorax (deg/s)
Distance chair to start turn (m)
Range thorax obliquity (deg)
Mean angular velocity pelvis (deg/s)
Steps Number
Peak forward velocity pelvis (m/s)
Walk
S2S
Time
T2S
Turn
3.8
50.5
1.1
1.2
64.6
2.2
1.1
2.8
0.6
8.3
257.3
1.6
8.0
163.4
2.0
1.9
35.0
11.9
0.2
14.2
14.3
8.5
4.2
17.4
2.9
29.1
83.1
0.5
48.4
41.4
6.0
0.5

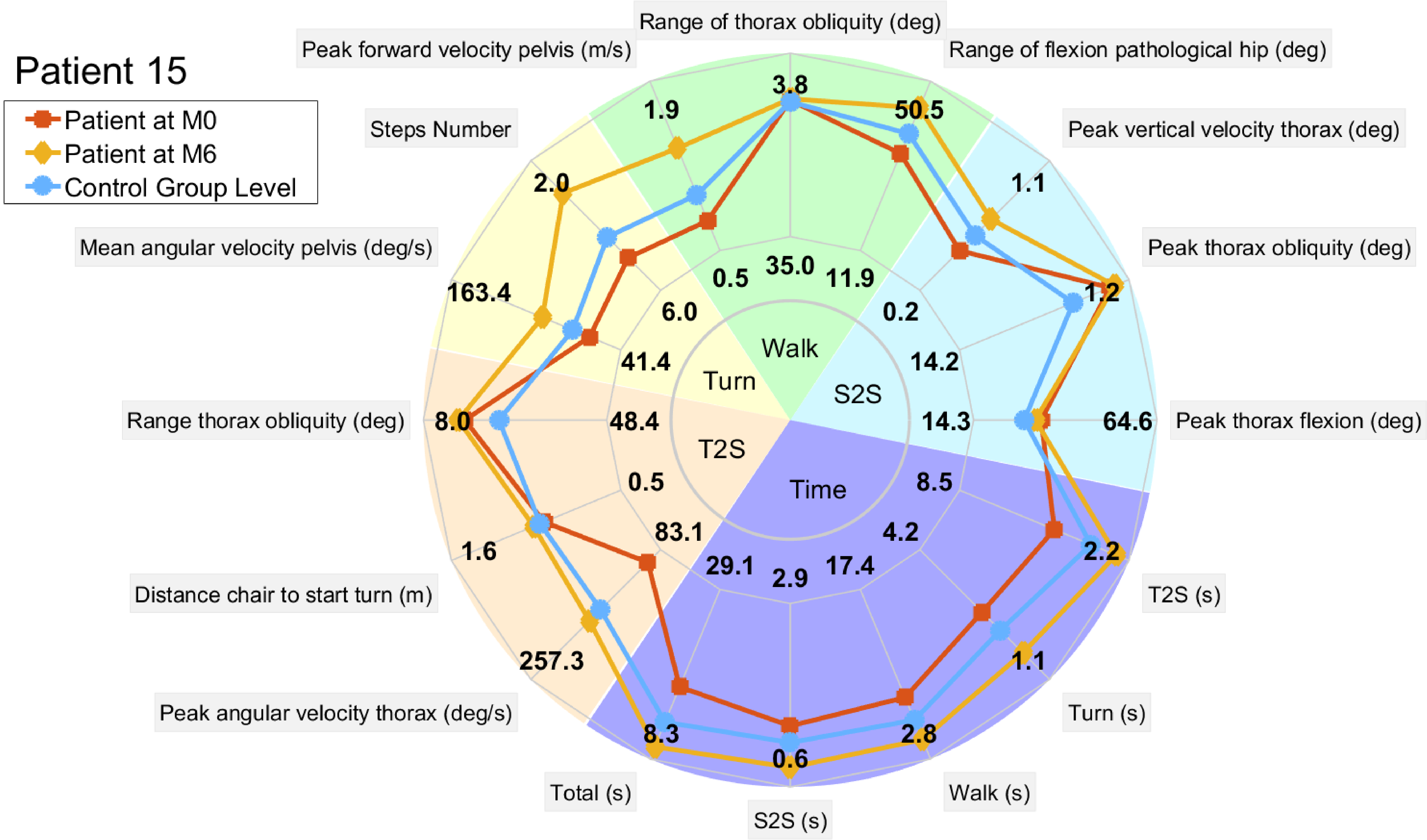
Patient 15
Patient at M0
Patient at M6
Control Group Level
Range of thorax obliquity (deg)
Peak forward velocity pelvis (m/s)
Range of flexion pathological hip (deg)
Steps Number
Peak vertical velocity thorax (deg)
Mean angular velocity pelvis (deg/s)
Peak thorax obliquity (deg)
Range thorax obliquity (deg)
Peak thorax flexion (deg)
Distance chair to start turn (m)
T2S (s)
Peak angular velocity thorax (deg/s)
Turn (s)
Total (s)
Walk (s)
S2S (s)
Walk
S2S
Turn
T2S
Time
3.8
1.9
50.5
2.0
1.1
163.4
1.2
8.0
64.6
1.6
2.2
257.3
1.1
8.3
2.8
0.6
0.5
35.0
11.9
6.0
0.2
41.4
14.2
48.4
14.3
0.5
8.5
83.1
4.2
29.1
2.9
17.4

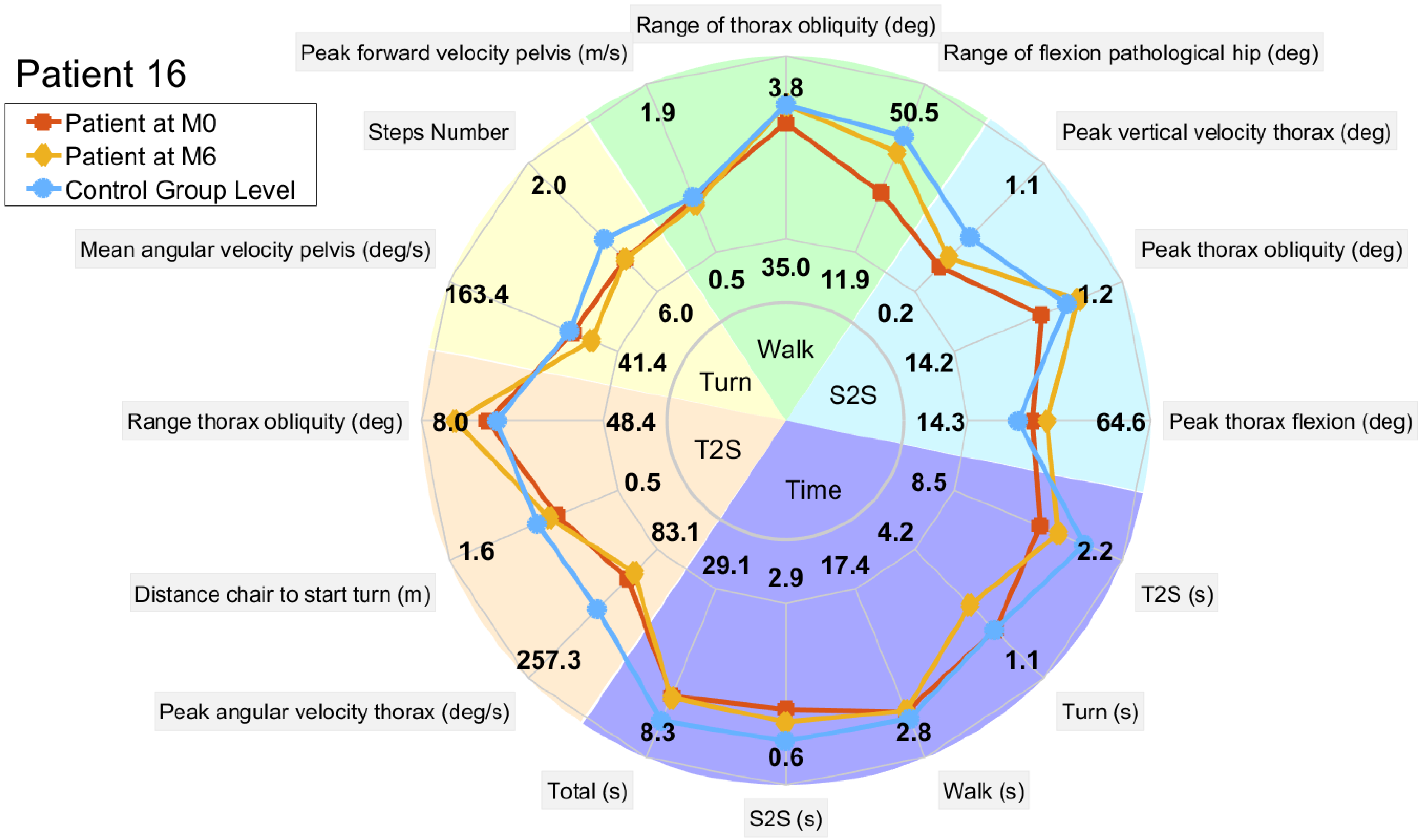

Patient 16
Patient at M0
Patient at M6
Control Group Level
Range of thorax obliquity (deg)
Peak forward velocity pelvis (m/s)
Range of flexion pathological hip (deg)
Steps Number
Peak vertical velocity thorax (deg)
Mean angular velocity pelvis (deg/s)
Peak thorax obliquity (deg)
Range thorax obliquity (deg)
Peak thorax flexion (deg)
Distance chair to start turn (m)
T2S (s)
Peak angular velocity thorax (deg/s)
Turn (s)
Total (s)
Walk (s)
S2S (s)
Walk
Turn
S2S
T2S
Time
3.8
1.9
50.5
2.0
1.1
163.4
1.2
8.0
64.6
1.6
2.2
257.3
1.1
8.3
0.6
2.8
0.5
35.0
11.9
6.0
0.2
41.4
14.2
48.4
14.3
0.5
8.5
83.1
4.2
29.1
2.9
17.4

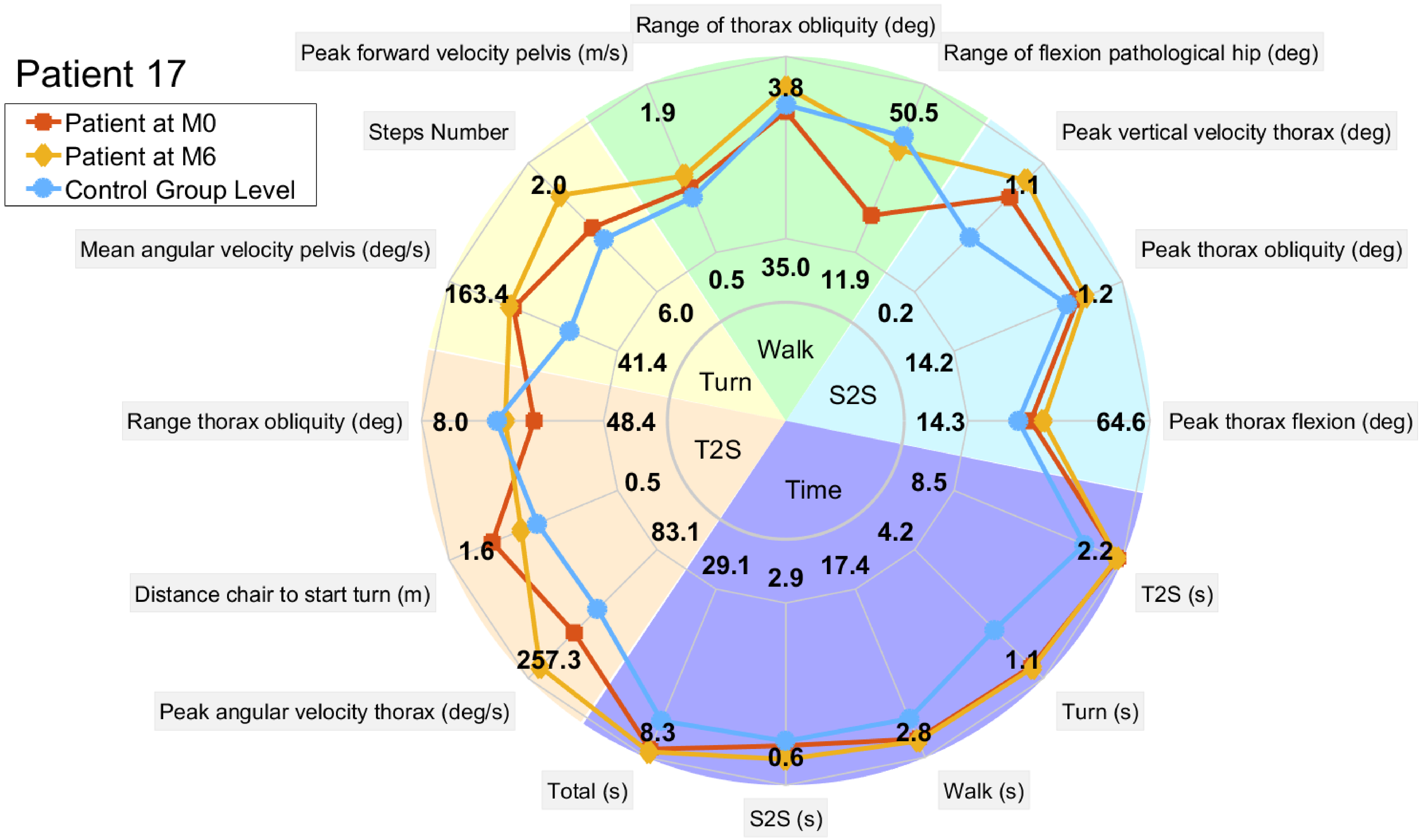

Patient 17
Patient at M0
Patient at M6
Control Group Level
Range of thorax obliquity (deg)
Peak forward velocity pelvis (m/s)
Range of flexion pathological hip (deg)
Steps Number
Peak vertical velocity thorax (deg)
Mean angular velocity pelvis (deg/s)
Peak thorax obliquity (deg)
Range thorax obliquity (deg)
Peak thorax flexion (deg)
Distance chair to start turn (m)
T2S (s)
Peak angular velocity thorax (deg/s)
Turn (s)
Total (s)
Walk (s)
S2S (s)
Walk
Turn
S2S
T2S
Time
3.8
1.9
50.5
2.0
1.1
163.4
1.2
8.0
64.6
1.6
2.2
257.3
1.1
8.3
2.8
0.6
35.0
0.5
11.9
6.0
0.2
41.4
14.2
48.4
14.3
0.5
8.5
83.1
4.2
29.1
2.9
17.4

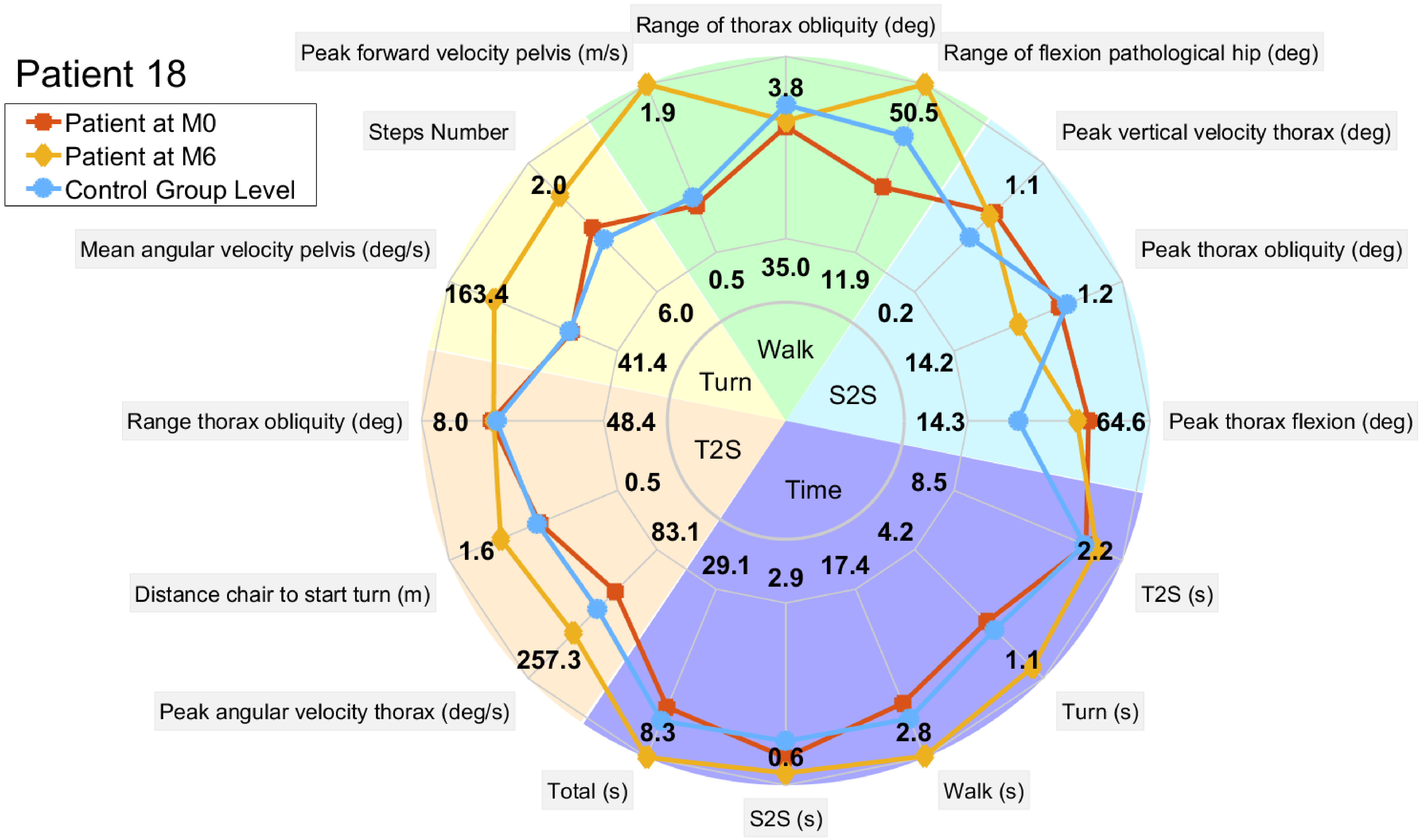

Patient 18
Patient at M0
Patient at M6
Control Group Level
Range of thorax obliquity (deg)
Range of flexion pathological hip (deg)
Peak vertical velocity thorax (deg)
Peak thorax obliquity (deg)
Peak thorax flexion (deg)
T2S (s)
Turn (s)
Walk (s)
S2S (s)
Total (s)
Peak angular velocity thorax (deg/s)
Distance chair to start turn (m)
Range thorax obliquity (deg)
Mean angular velocity pelvis (deg/s)
Steps Number
Peak forward velocity pelvis (m/s)
Walk
Turn
S2S
T2S
Time
3.8
50.5
1.1
1.2
64.6
2.2
1.1
2.8
0.6
8.3
257.3
1.6
8.0
163.4
2.0
1.9
35.0
11.9
0.2
14.2
14.3
8.5
4.2
17.4
2.9
29.1
83.1
0.5
48.4
41.4
6.0
0.5

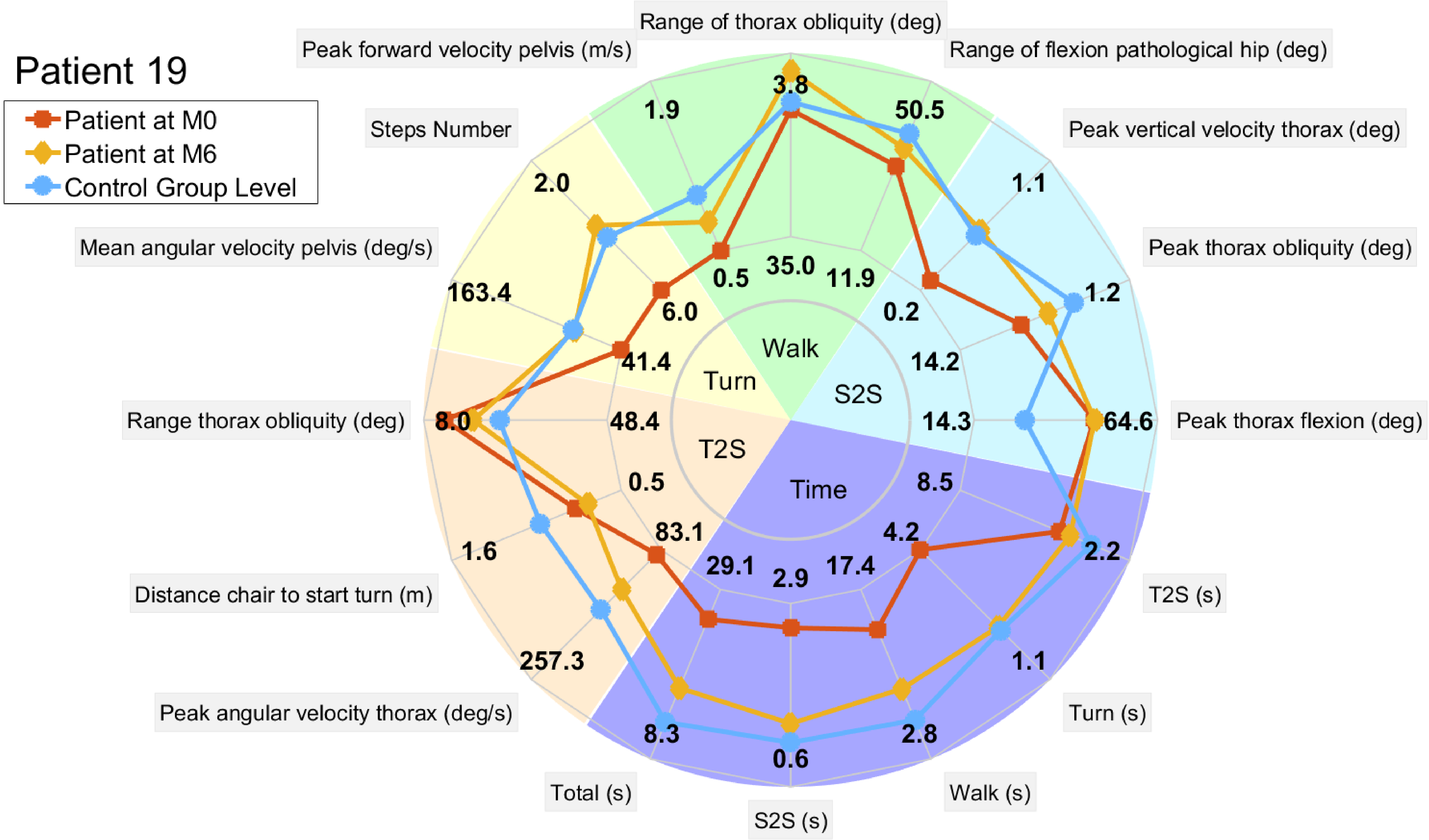
Patient 19
Patient at M0
Patient at M6
Control Group Level
Range of thorax obliquity (deg)
Range of flexion pathological hip (deg)
Peak vertical velocity thorax (deg)
Peak thorax obliquity (deg)
Peak thorax flexion (deg)
T2S (s)
Turn (s)
Walk (s)
S2S (s)
Total (s)
Peak angular velocity thorax (deg/s)
Distance chair to start turn (m)
Range thorax obliquity (deg)
Mean angular velocity pelvis (deg/s)
Steps Number
Peak forward velocity pelvis (m/s)
Walk
S2S
Time
T2S
Turn
3.8
50.5
1.1
1.2
64.6
2.2
1.1
2.8
0.6
8.3
257.3
1.6
8.0
163.4
2.0
1.9
35.0
11.9
0.2
14.2
14.3
8.5
4.2
17.4
2.9
29.1
83.1
0.5
48.4
41.4
6.0
0.5

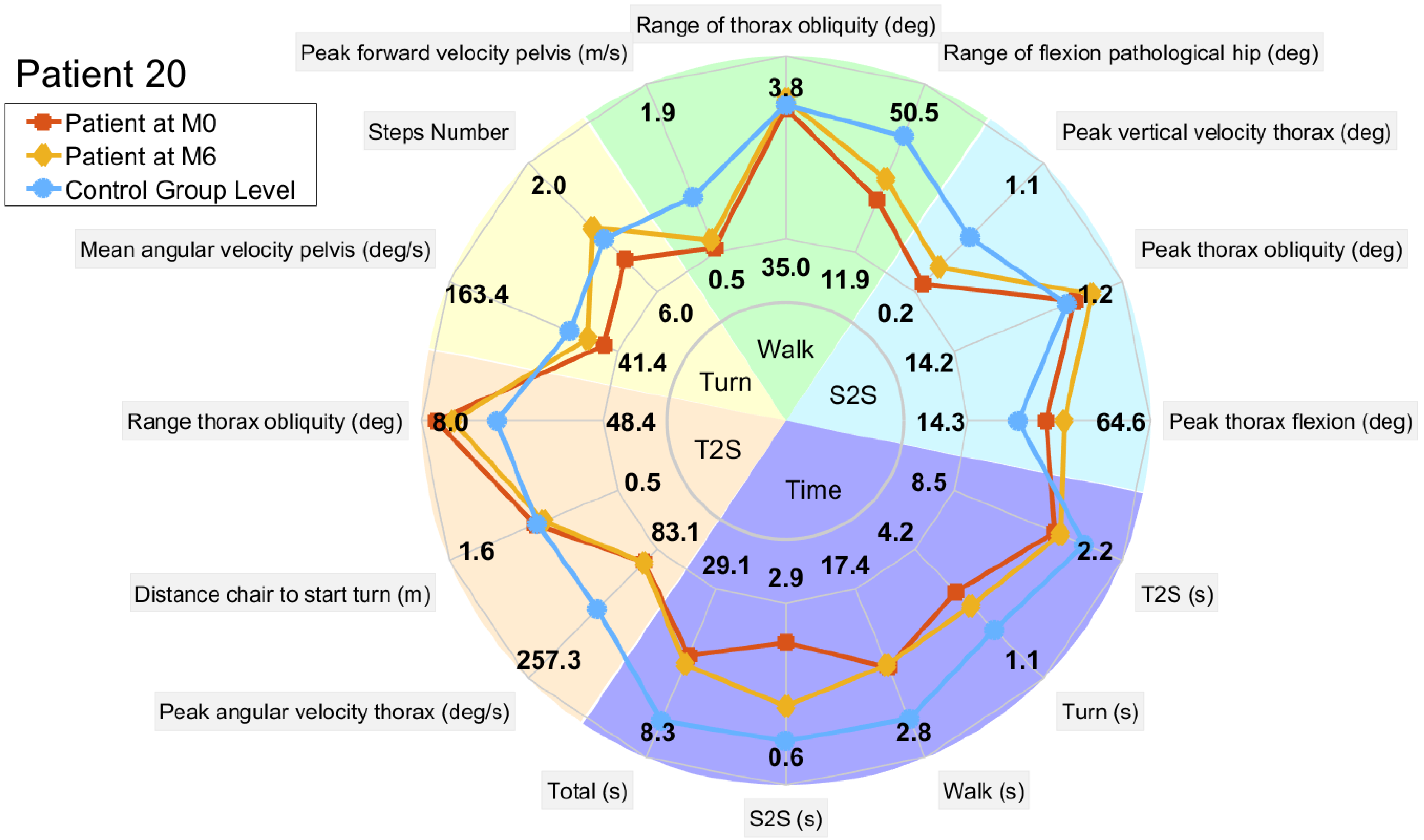

Patient 20
Patient at M0
Patient at M6
Control Group Level
Range of thorax obliquity (deg)
Range of flexion pathological hip (deg)
Peak vertical velocity thorax (deg)
Peak thorax obliquity (deg)
Peak thorax flexion (deg)
T2S (s)
Turn (s)
Walk (s)
S2S (s)
Total (s)
Peak angular velocity thorax (deg/s)
Distance chair to start turn (m)
Range thorax obliquity (deg)
Mean angular velocity pelvis (deg/s)
Steps Number
Peak forward velocity pelvis (m/s)
Walk
S2S
Time
T2S
Turn
3.8
35.0
50.5
11.9
1.1
0.2
1.2
14.2
64.6
14.3
2.2
8.5
1.1
4.2
2.8
17.4
0.6
2.9
8.3
29.1
257.3
83.1
1.6
0.5
8.0
48.4
163.4
41.4
2.0
6.0
1.9
0.5

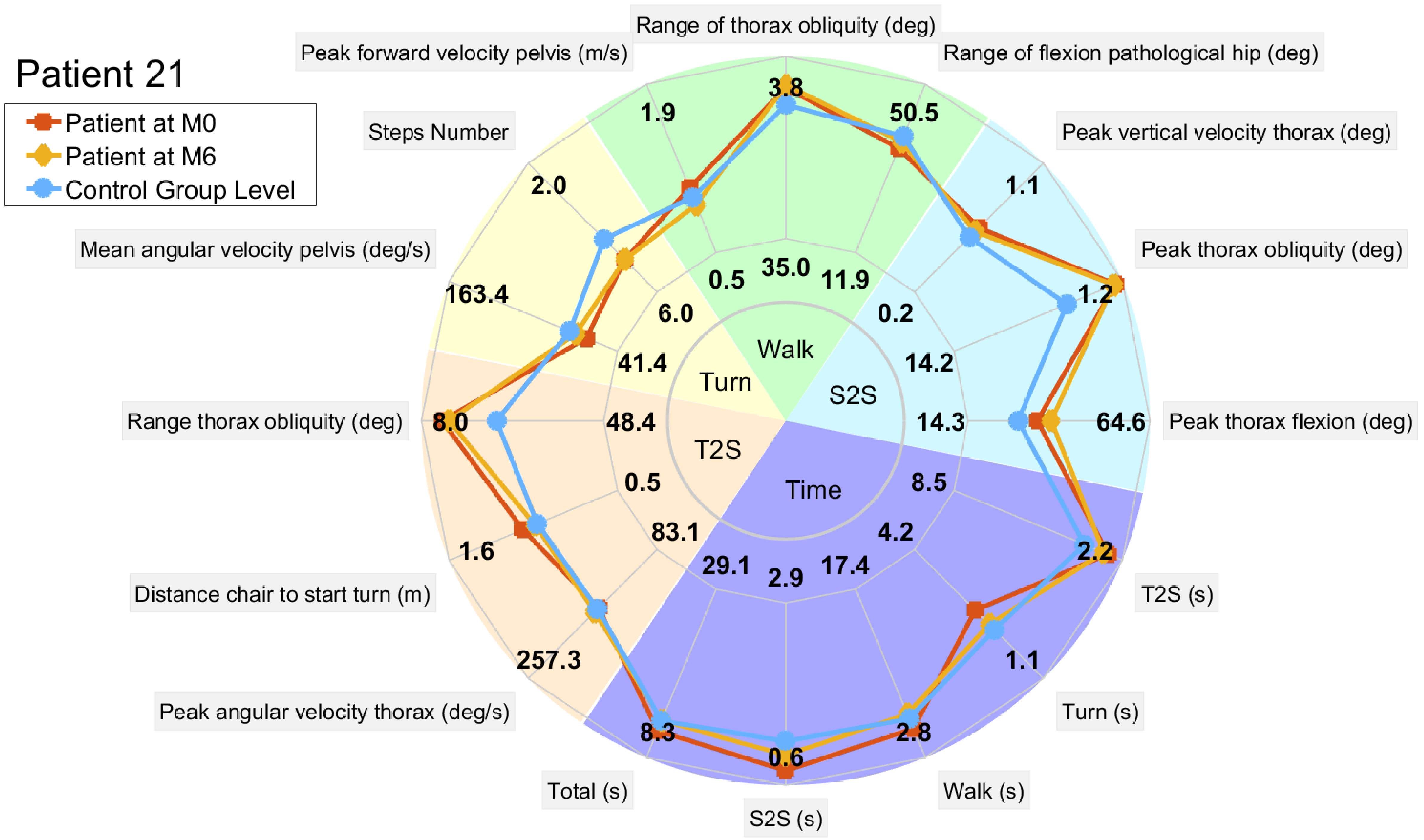
Patient 21
Patient at M0
Patient at M6
Control Group Level
Range of thorax obliquity (deg)
Range of flexion pathological hip (deg)
Peak vertical velocity thorax (deg)
Peak thorax obliquity (deg)
Peak thorax flexion (deg)
T2S (s)
Turn (s)
Walk (s)
S2S (s)
Total (s)
Peak angular velocity thorax (deg/s)
Distance chair to start turn (m)
Range thorax obliquity (deg)
Mean angular velocity pelvis (deg/s)
Steps Number
Peak forward velocity pelvis (m/s)
Walk
S2S
Time
T2S
Turn
3.8
50.5
1.1
1.2
64.6
2.2
1.1
2.8
0.6
8.3
257.3
1.6
8.0
163.4
2.0
1.9
35.0
11.9
0.2
14.2
14.3
8.5
4.2
17.4
2.9
29.1
83.1
0.5
48.4
41.4
6.0
0.5

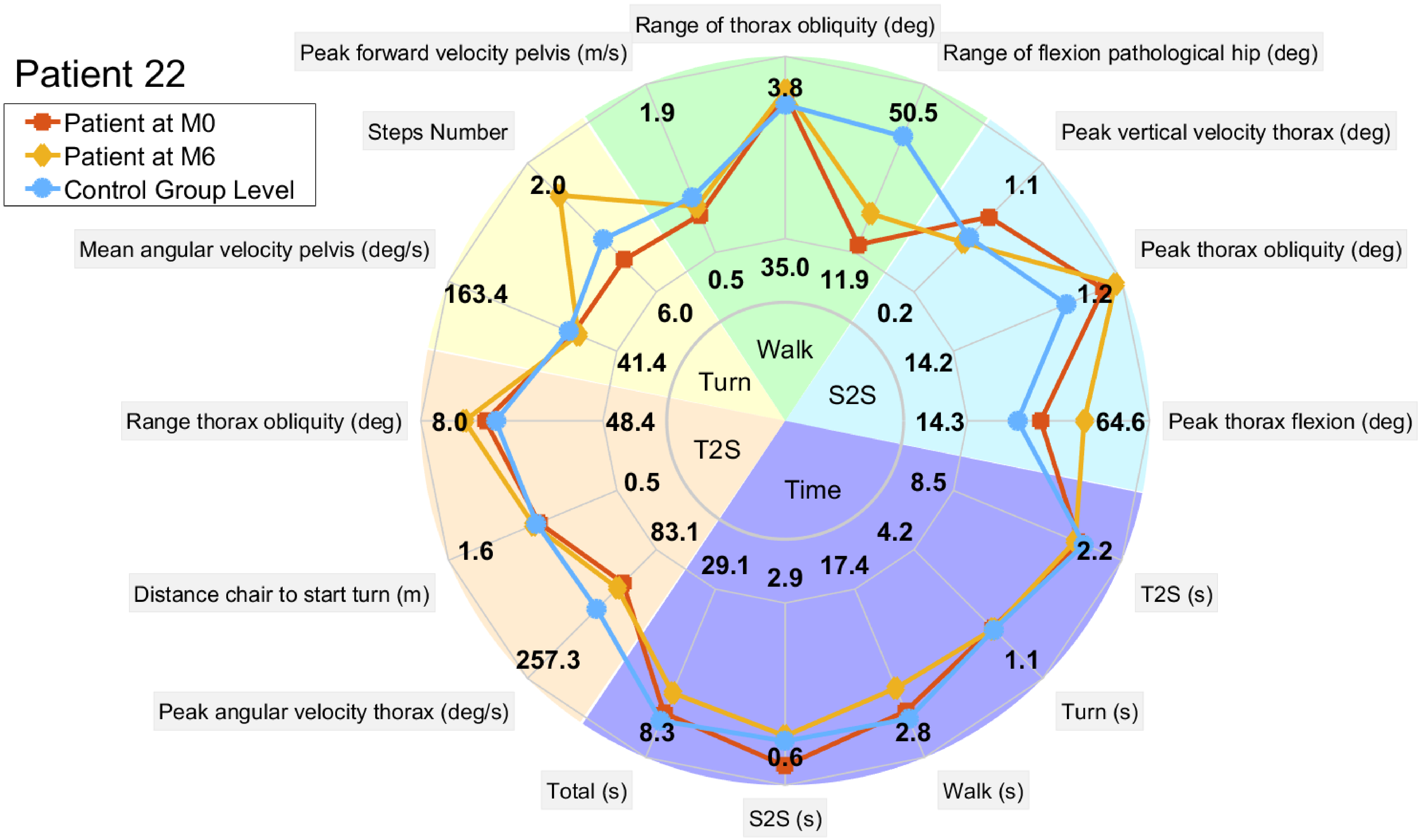
Patient 22
Patient at M0
Patient at M6
Control Group Level
Range of thorax obliquity (deg)
Peak forward velocity pelvis (m/s)
Range of flexion pathological hip (deg)
Steps Number
Peak vertical velocity thorax (deg)
Mean angular velocity pelvis (deg/s)
Peak thorax obliquity (deg)
Range thorax obliquity (deg)
Peak thorax flexion (deg)
Distance chair to start turn (m)
T2S (s)
Peak angular velocity thorax (deg/s)
Turn (s)
Total (s)
Walk (s)
S2S (s)
Walk
Turn
S2S
T2S
Time
3.8
1.9
50.5
2.0
1.1
163.4
1.2
8.0
64.6
1.6
2.2
257.3
1.1
8.3
2.8
0.6
0.5
35.0
11.9
6.0
0.2
41.4
14.2
48.4
14.3
0.5
8.5
83.1
4.2
29.1
2.9
17.4

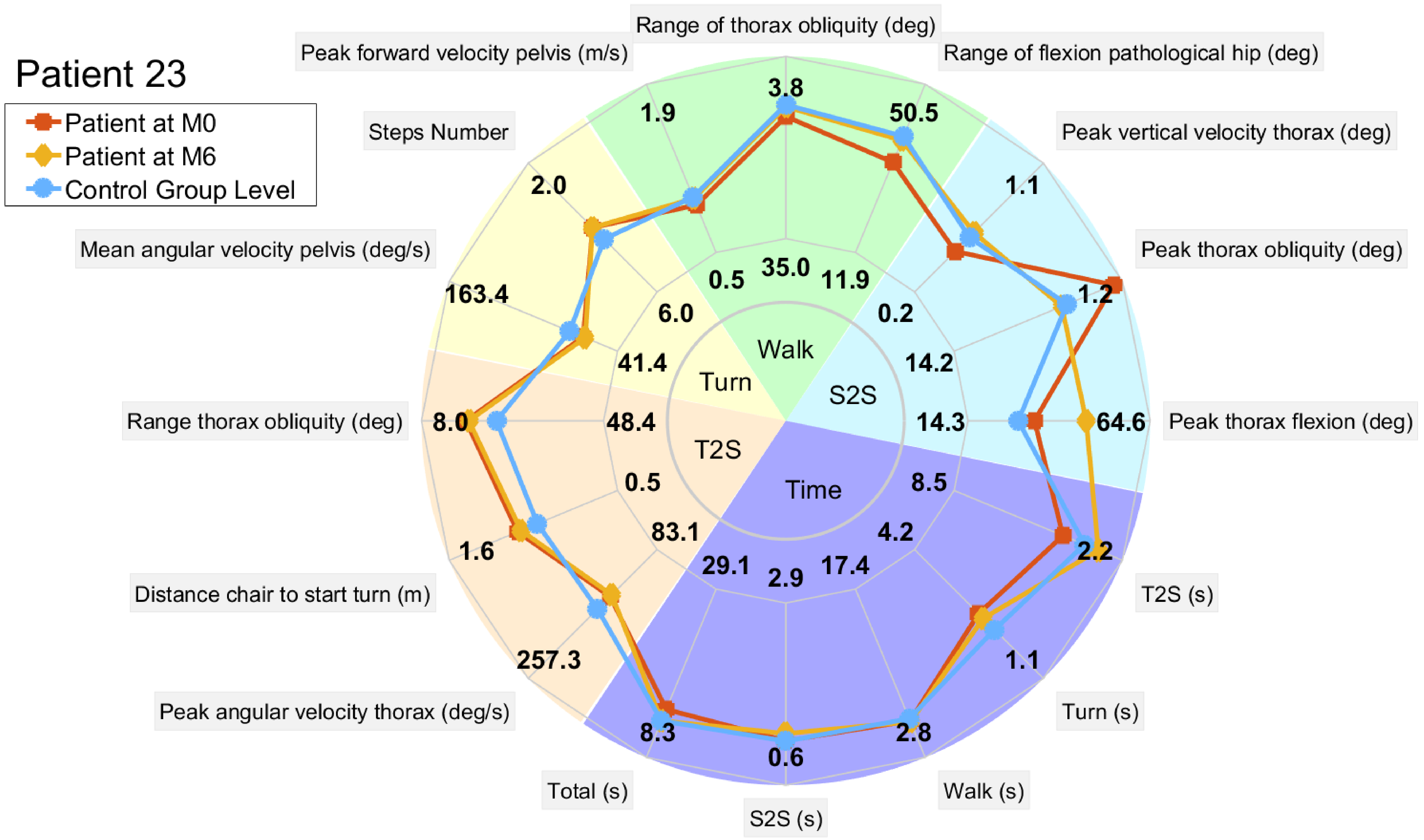

Patient 23
Patient at M0
Patient at M6
Control Group Level
Range of thorax obliquity (deg)
Range of flexion pathological hip (deg)
Peak vertical velocity thorax (deg)
Peak thorax obliquity (deg)
Peak thorax flexion (deg)
T2S (s)
Turn (s)
Walk (s)
S2S (s)
Total (s)
Peak angular velocity thorax (deg/s)
Distance chair to start turn (m)
Range thorax obliquity (deg)
Mean angular velocity pelvis (deg/s)
Steps Number
Peak forward velocity pelvis (m/s)
Walk
S2S
Time
T2S
Turn
3.8
50.5
1.1
1.2
64.6
2.2
1.1
2.8
0.6
8.3
257.3
1.6
8.0
163.4
2.0
1.9
35.0
11.9
0.2
14.2
14.3
8.5
4.2
17.4
2.9
29.1
83.1
0.5
48.4
41.4
6.0
0.5

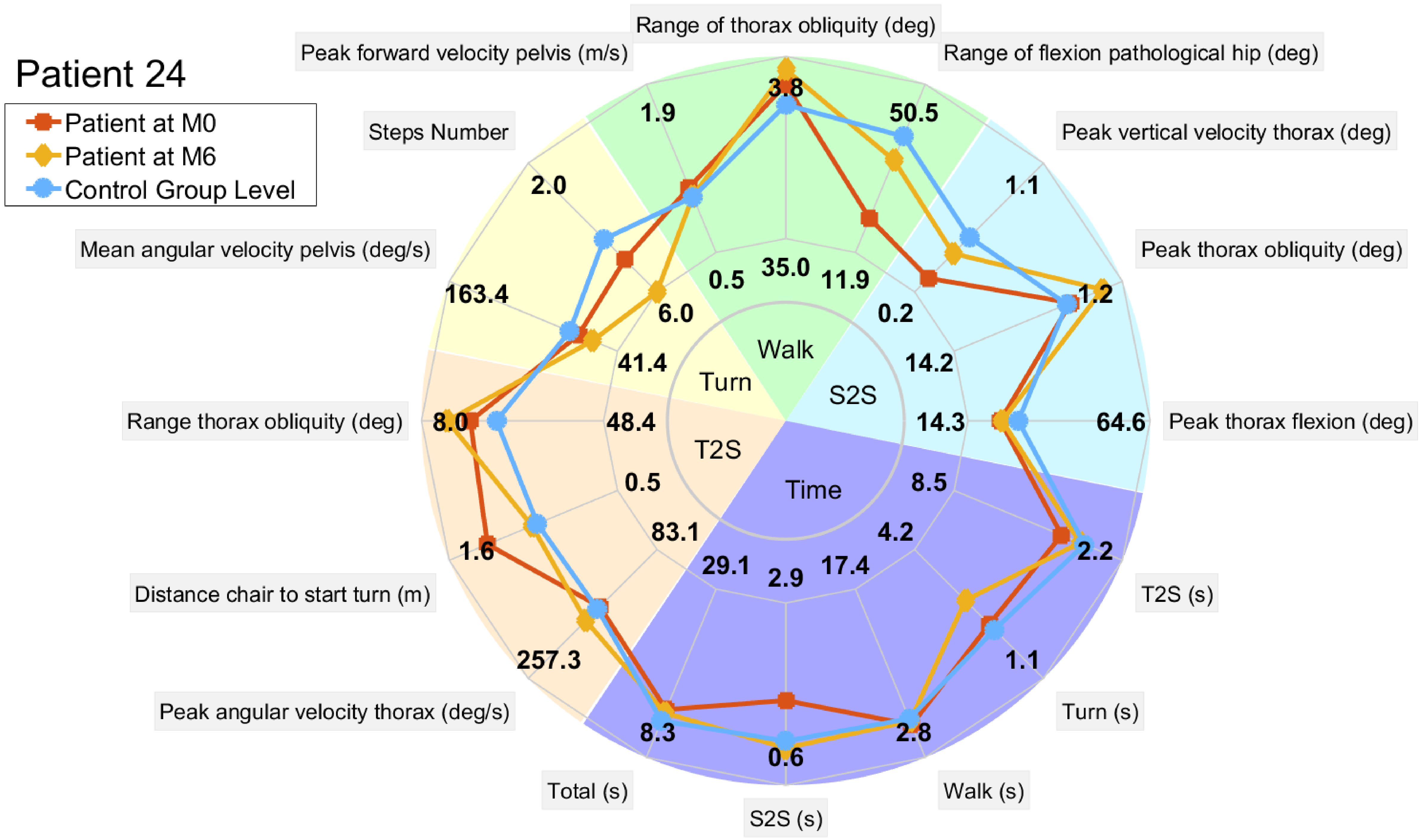

Patient 24
Patient at M0
Patient at M6
Control Group Level
Range of thorax obliquity (deg)
Peak forward velocity pelvis (m/s)
Range of flexion pathological hip (deg)
Steps Number
Peak vertical velocity thorax (deg)
Mean angular velocity pelvis (deg/s)
Peak thorax obliquity (deg)
Range thorax obliquity (deg)
Peak thorax flexion (deg)
Distance chair to start turn (m)
T2S (s)
Peak angular velocity thorax (deg/s)
Turn (s)
Total (s)
Walk (s)
S2S (s)
Walk
Turn
S2S
T2S
Time
3.8
1.9
50.5
2.0
1.1
163.4
1.2
8.0
64.6
1.6
2.2
257.3
1.1
8.3
0.6
2.8
0.5
35.0
11.9
6.0
0.2
41.4
14.2
48.4
14.3
0.5
8.5
83.1
4.2
29.1
2.9
17.4

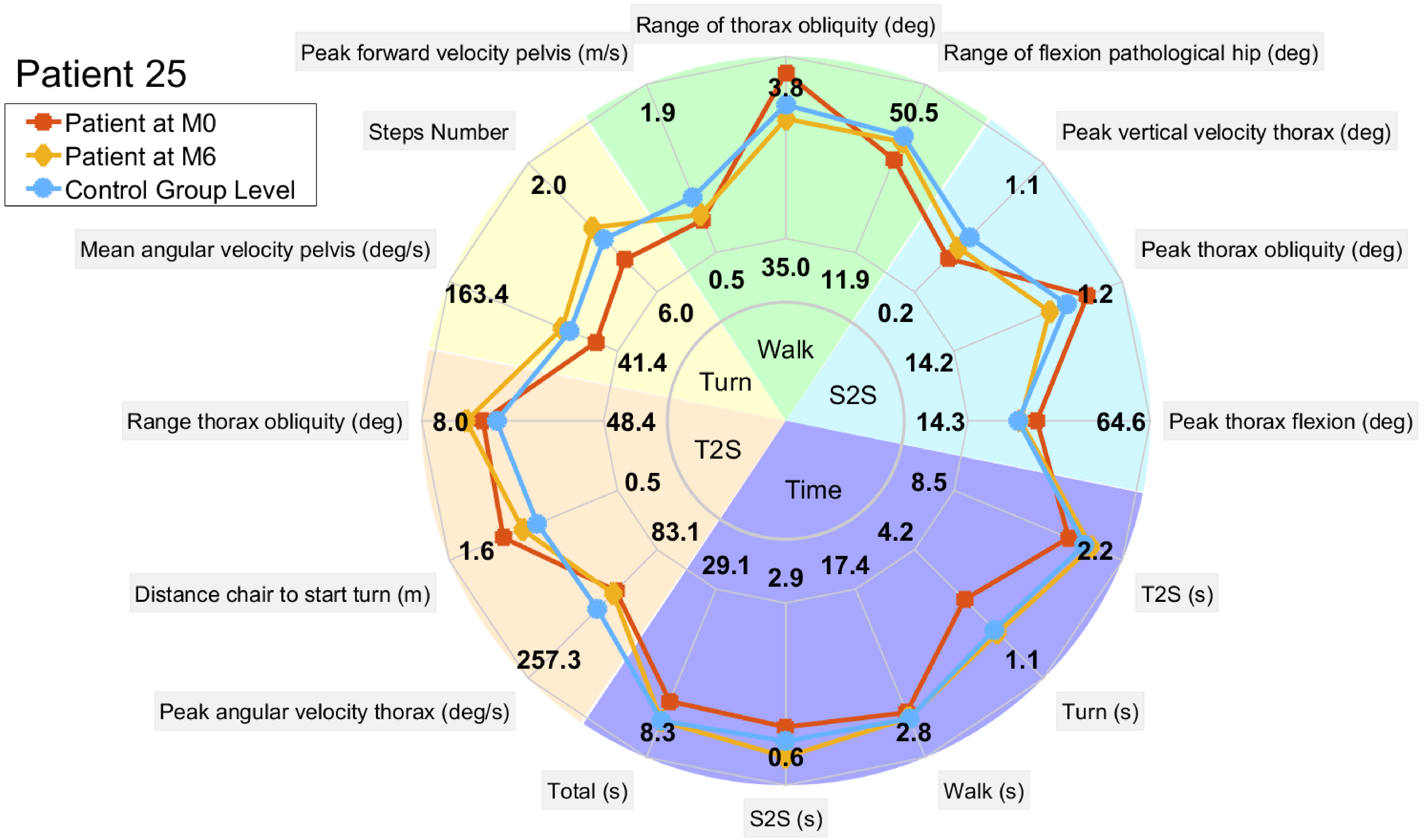
Patient 25
Patient at M0
Patient at M6
Control Group Level
Range of thorax obliquity (deg)
Range of flexion pathological hip (deg)
Peak vertical velocity thorax (deg)
Peak thorax obliquity (deg)
Peak thorax flexion (deg)
T2S (s)
Turn (s)
Walk (s)
S2S (s)
Total (s)
Peak angular velocity thorax (deg/s)
Distance chair to start turn (m)
Range thorax obliquity (deg)
Mean angular velocity pelvis (deg/s)
Steps Number
Peak forward velocity pelvis (m/s)
Walk
S2S
Time
T2S
Turn
3.8
35.0
50.5
11.9
1.1
0.2
1.2
14.2
64.6
14.3
2.2
8.5
1.1
4.2
2.8
17.4
0.6
2.9
8.3
29.1
257.3
83.1
1.6
0.5
8.0
48.4
163.4
41.4
2.0
6.0
1.9
0.5

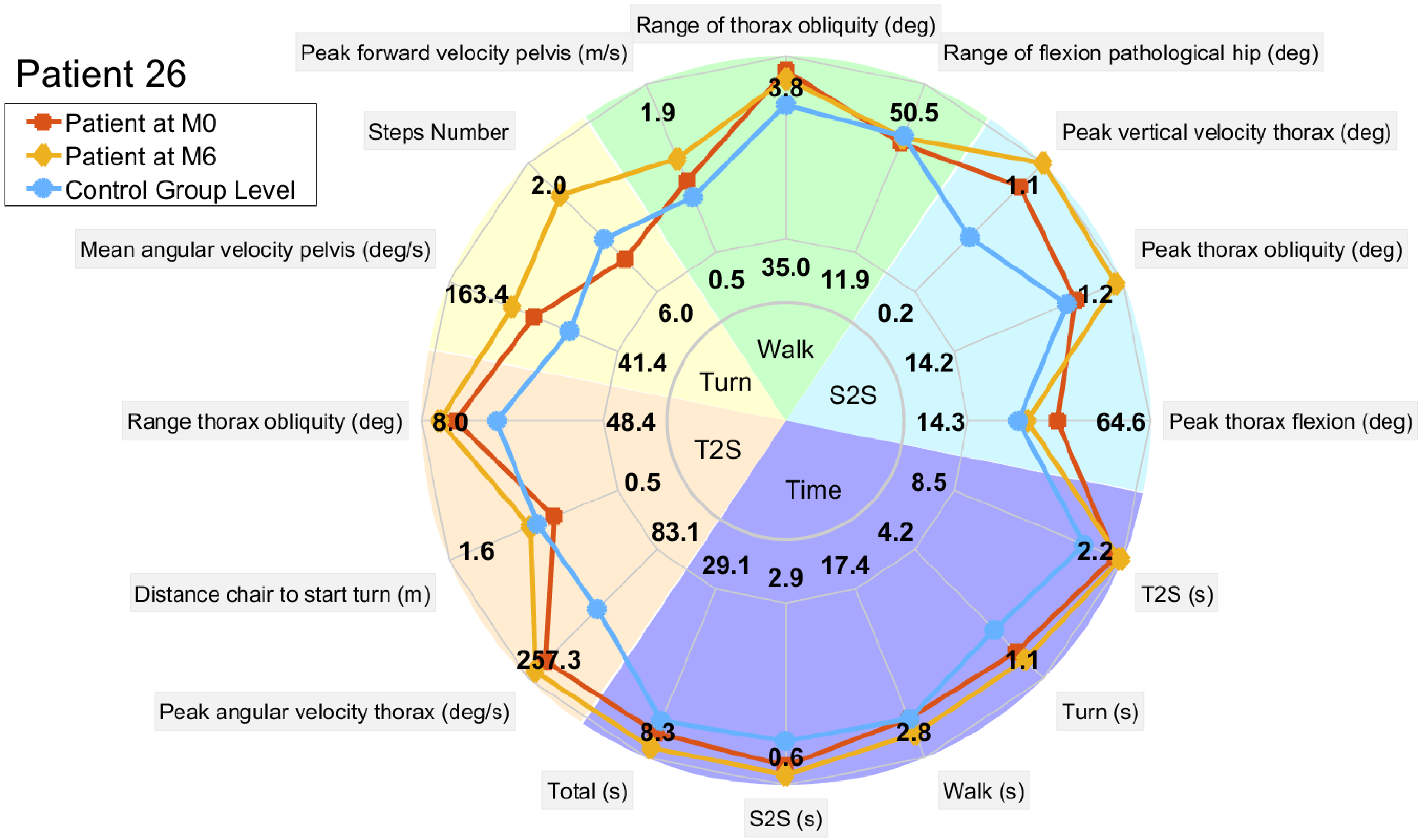

Patient 26
Patient at M0
Patient at M6
Control Group Level
Range of thorax obliquity (deg)
Range of flexion pathological hip (deg)
Peak vertical velocity thorax (deg)
Peak thorax obliquity (deg)
Peak thorax flexion (deg)
T2S (s)
Turn (s)
Walk (s)
S2S (s)
Total (s)
Peak angular velocity thorax (deg/s)
Distance chair to start turn (m)
Range thorax obliquity (deg)
Mean angular velocity pelvis (deg/s)
Steps Number
Peak forward velocity pelvis (m/s)
Walk
S2S
Time
T2S
Turn
3.8
50.5
1.1
1.2
64.6
2.2
1.1
2.8
0.6
8.3
257.3
1.6
8.0
163.4
2.0
1.9
35.0
11.9
0.2
14.2
14.3
8.5
4.2
17.4
2.9
29.1
83.1
0.5
48.4
41.4
6.0
0.5

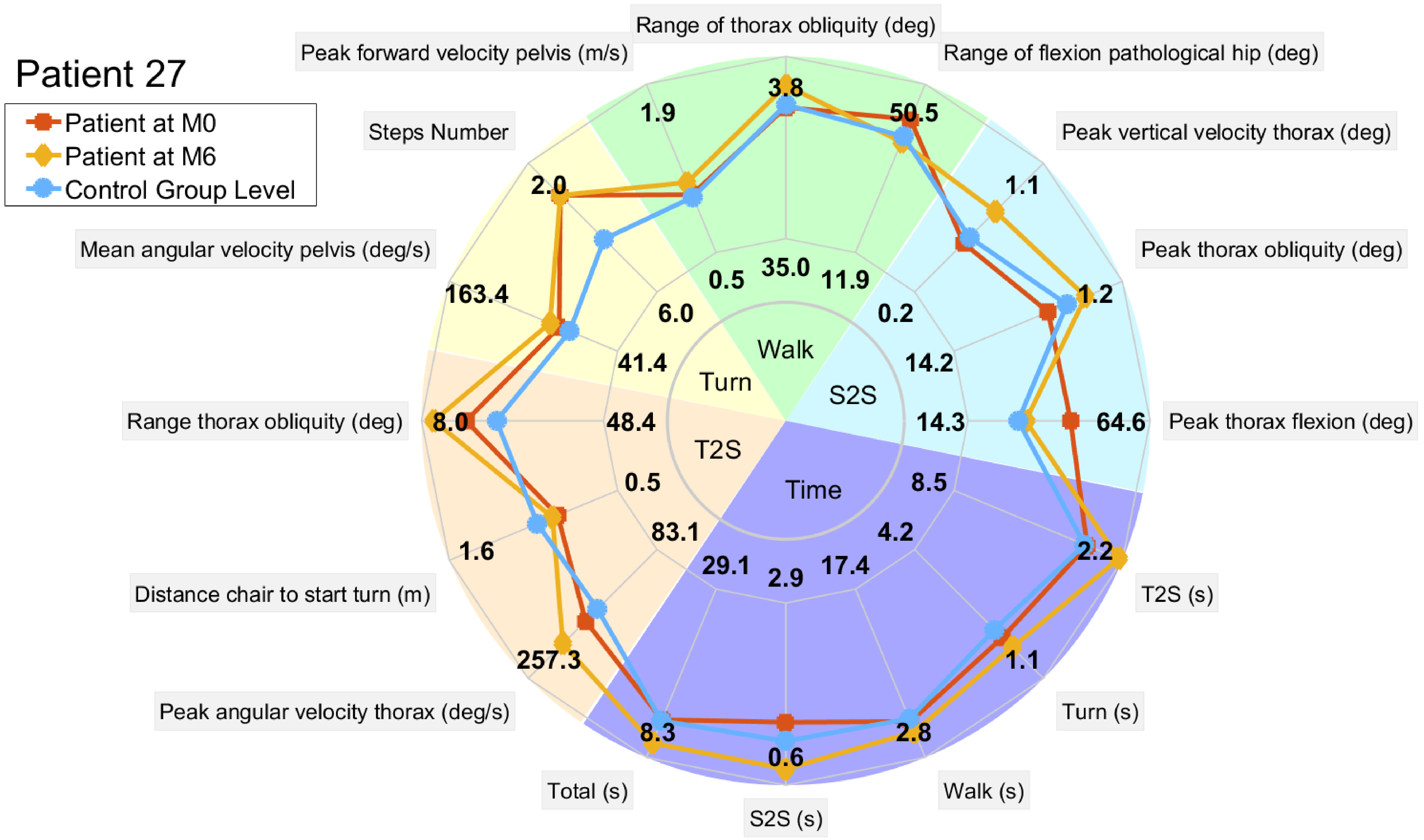

Patient 27
Patient at M0
Patient at M6
Control Group Level
Range of thorax obliquity (deg)
Range of flexion pathological hip (deg)
Peak vertical velocity thorax (deg)
Peak thorax obliquity (deg)
Peak thorax flexion (deg)
T2S (s)
Turn (s)
Walk (s)
S2S (s)
Total (s)
Peak angular velocity thorax (deg/s)
Distance chair to start turn (m)
Range thorax obliquity (deg)
Mean angular velocity pelvis (deg/s)
Steps Number
Peak forward velocity pelvis (m/s)
Walk
S2S
Time
T2S
Turn
3.8
50.5
1.1
1.2
64.6
2.2
1.1
2.8
0.6
8.3
257.3
1.6
8.0
163.4
2.0
1.9
35.0
11.9
0.2
14.2
14.3
8.5
4.2
17.4
2.9
29.1
83.1
0.5
48.4
41.4
6.0
0.5

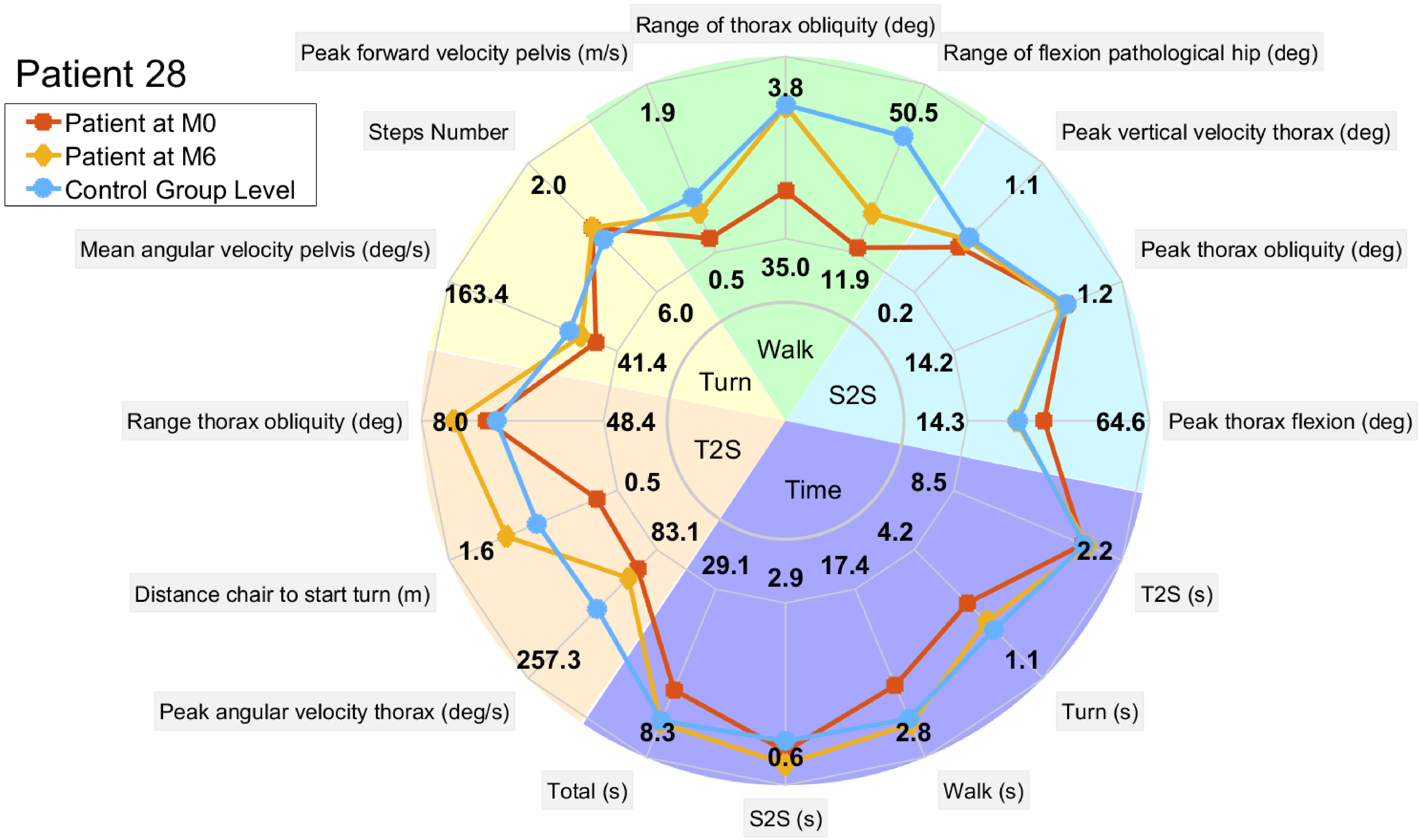

Patient 28
Patient at M0
Patient at M6
Control Group Level
Range of thorax obliquity (deg)
Range of flexion pathological hip (deg)
Peak vertical velocity thorax (deg)
Peak thorax obliquity (deg)
Peak thorax flexion (deg)
T2S (s)
Turn (s)
Walk (s)
S2S (s)
Total (s)
Peak angular velocity thorax (deg/s)
Distance chair to start turn (m)
Range thorax obliquity (deg)
Mean angular velocity pelvis (deg/s)
Steps Number
Peak forward velocity pelvis (m/s)
Walk
S2S
Time
T2S
Turn
3.8
50.5
1.1
1.2
64.6
2.2
1.1
2.8
0.6
8.3
257.3
1.6
8.0
163.4
2.0
1.9
35.0
11.9
0.2
14.2
14.3
8.5
4.2
17.4
2.9
29.1
83.1
0.5
48.4
41.4
6.0
0.5

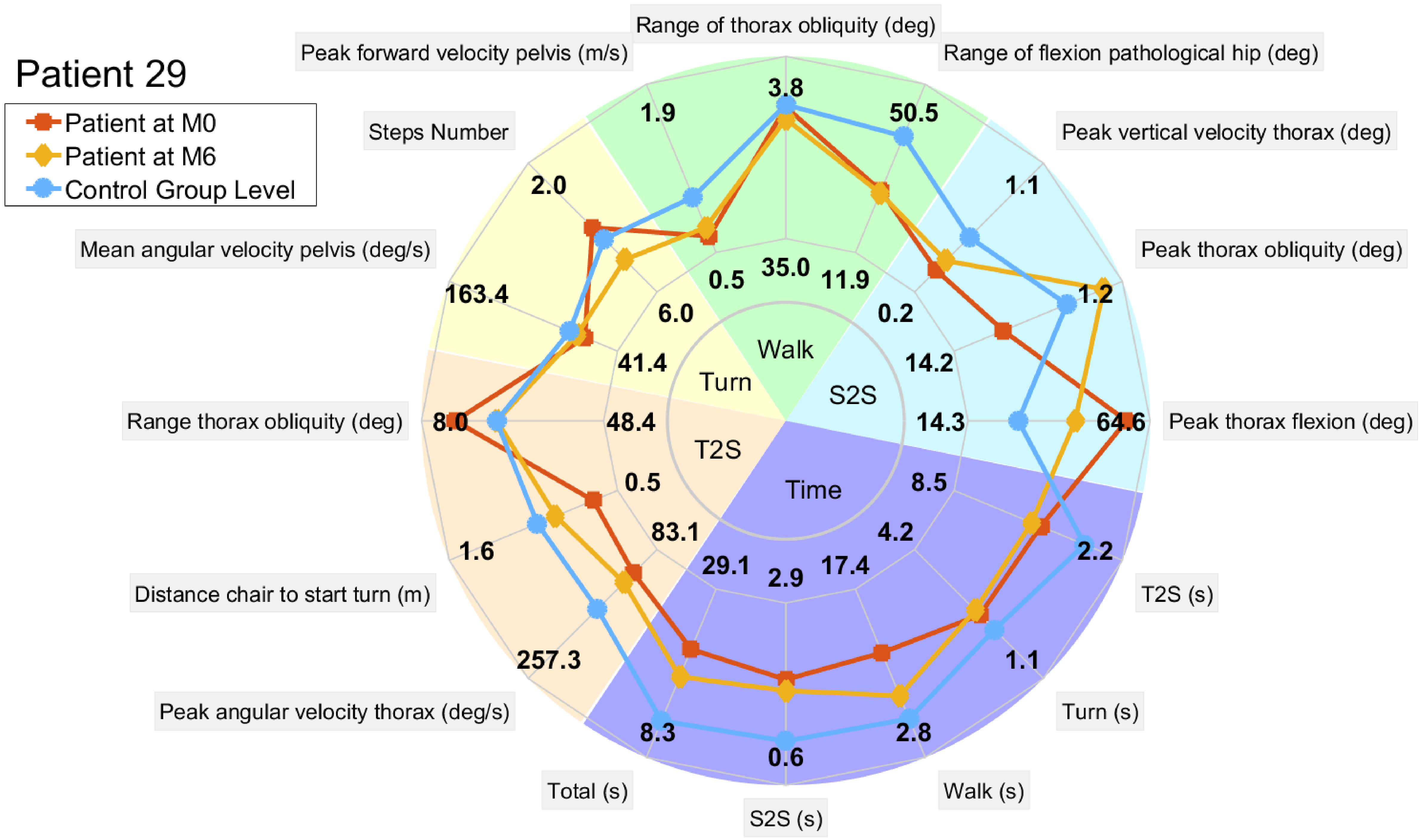
Patient 29
Patient at M0
Patient at M6
Control Group Level
Range of thorax obliquity (deg)
Peak forward velocity pelvis (m/s)
Range of flexion pathological hip (deg)
Steps Number
Peak vertical velocity thorax (deg)
Mean angular velocity pelvis (deg/s)
Peak thorax obliquity (deg)
Range thorax obliquity (deg)
Peak thorax flexion (deg)
Distance chair to start turn (m)
T2S (s)
Peak angular velocity thorax (deg/s)
Turn (s)
Total (s)
Walk (s)
S2S (s)
Walk
Turn
S2S
T2S
Time
3.8
1.9
50.5
2.0
1.1
163.4
1.2
8.0
64.6
1.6
2.2
257.3
1.1
8.3
0.6
2.8
0.5
35.0
11.9
6.0
0.2
41.4
14.2
48.4
14.3
0.5
8.5
83.1
4.2
29.1
2.9
17.4

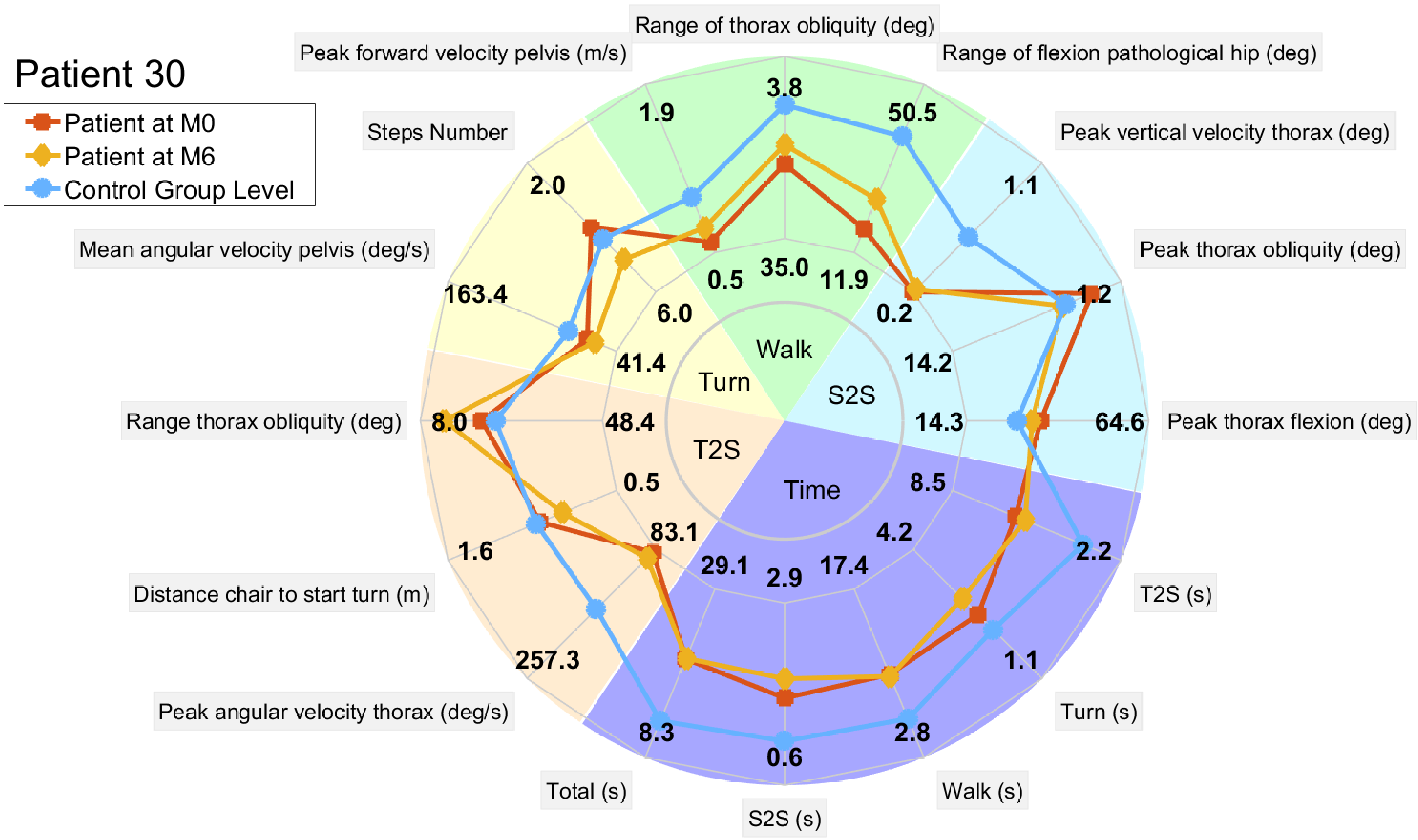

Patient 30
Patient at M0
Patient at M6
Control Group Level
Range of thorax obliquity (deg)
Peak forward velocity pelvis (m/s)
Range of flexion pathological hip (deg)
Steps Number
Peak vertical velocity thorax (deg)
Mean angular velocity pelvis (deg/s)
Peak thorax obliquity (deg)
Range thorax obliquity (deg)
Peak thorax flexion (deg)
Distance chair to start turn (m)
T2S (s)
Peak angular velocity thorax (deg/s)
Turn (s)
Total (s)
Walk (s)
S2S (s)
Walk
Turn
S2S
T2S
Time
3.8
1.9
50.5
2.0
1.1
163.4
1.2
8.0
64.6
1.6
2.2
257.3
1.1
8.3
2.8
0.6
35.0
0.5
11.9
6.0
0.2
41.4
14.2
48.4
14.3
0.5
8.5
83.1
4.2
29.1
2.9
17.4

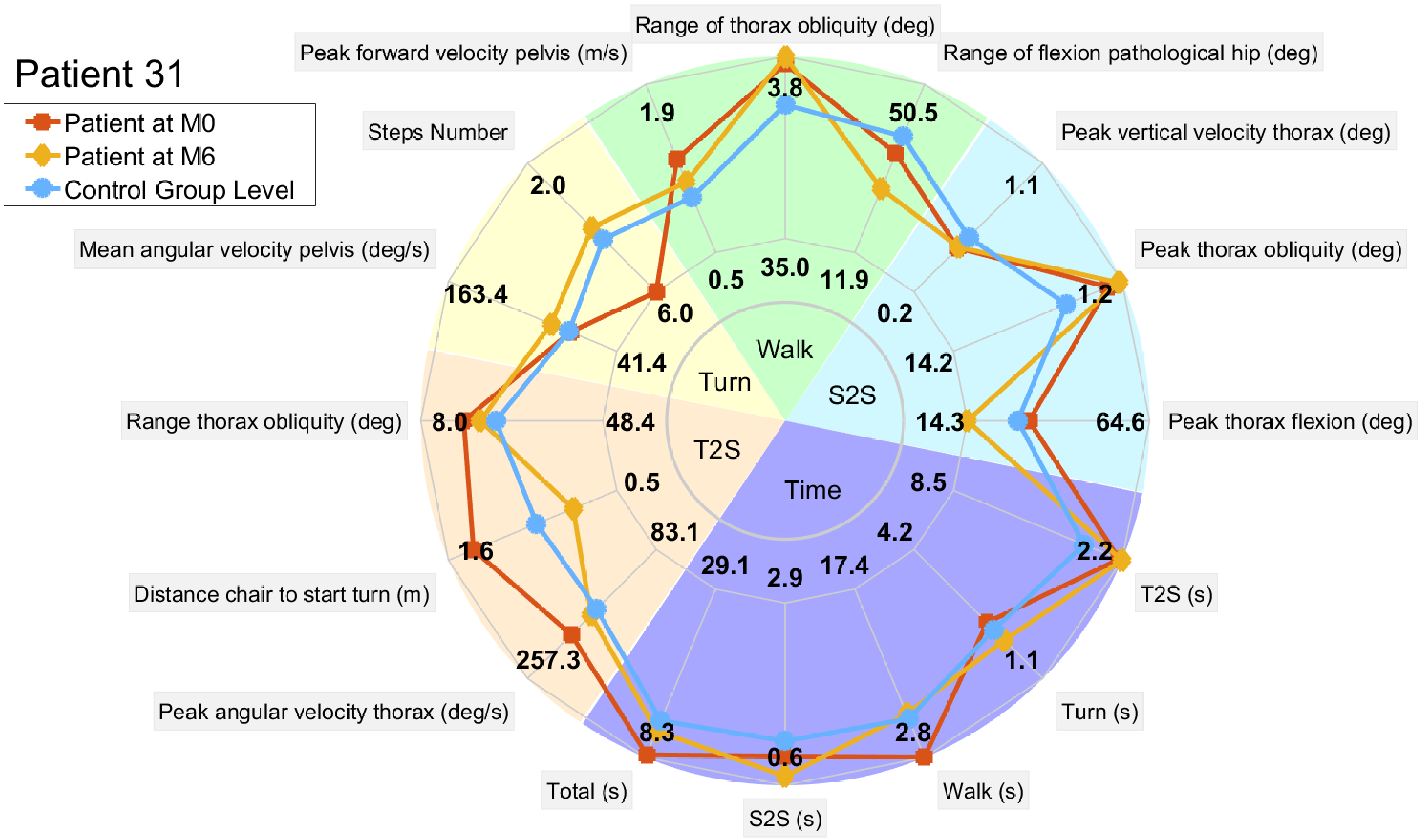
Patient 31
Patient at M0
Patient at M6
Control Group Level
Range of thorax obliquity (deg)
Peak forward velocity pelvis (m/s)
Range of flexion pathological hip (deg)
Steps Number
Peak vertical velocity thorax (deg)
Mean angular velocity pelvis (deg/s)
Peak thorax obliquity (deg)
Range thorax obliquity (deg)
Peak thorax flexion (deg)
Distance chair to start turn (m)
T2S (s)
Peak angular velocity thorax (deg/s)
Turn (s)
Total (s)
Walk (s)
S2S (s)
Walk
Turn
S2S
T2S
Time
3.8
1.9
50.5
2.0
1.1
163.4
1.2
8.0
64.6
1.6
2.2
257.3
1.1
8.3
0.6
2.8
35.0
0.5
11.9
6.0
0.2
41.4
14.2
48.4
14.3
0.5
8.5
83.1
4.2
29.1
2.9
17.4

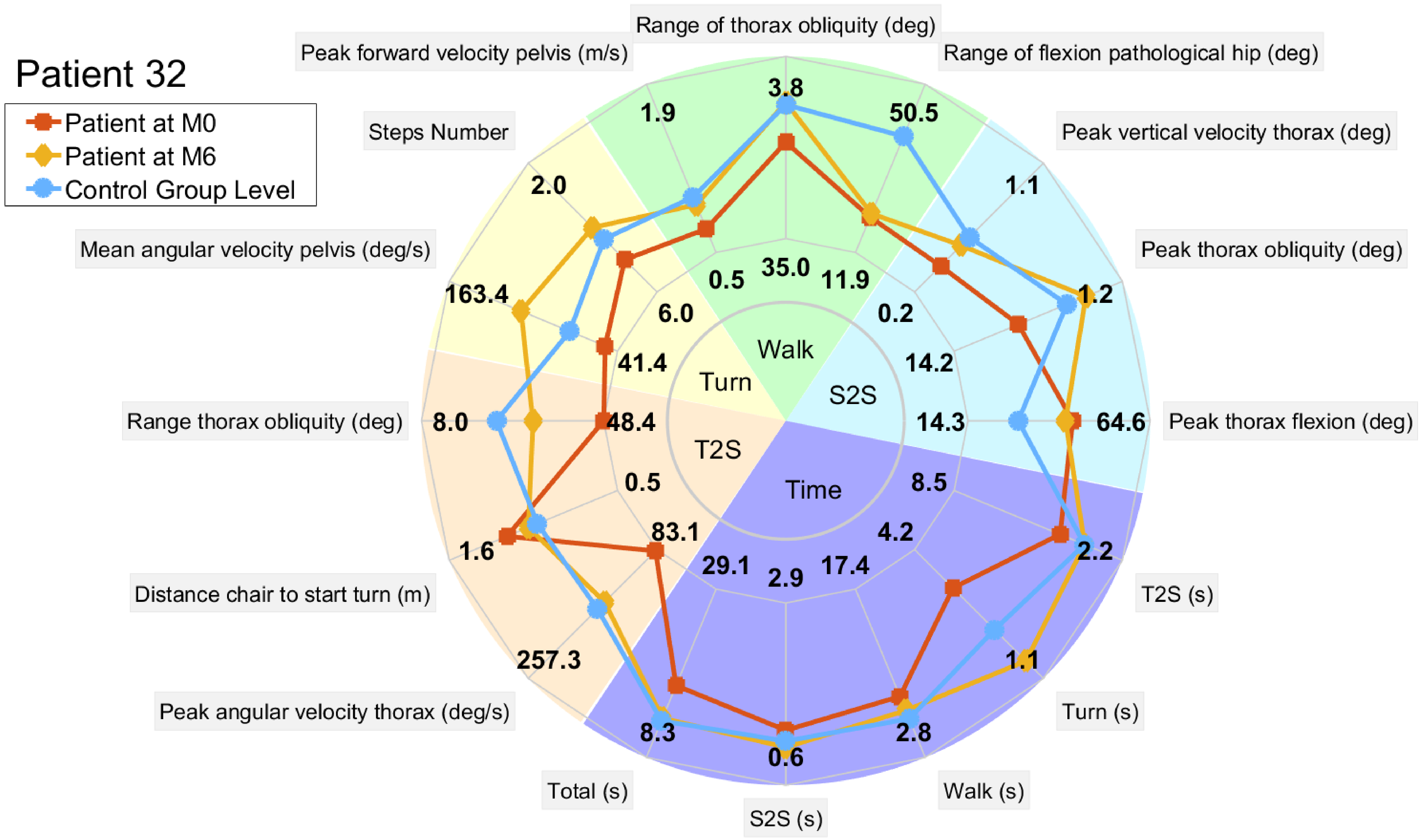

Patient 32
Patient at M0
Patient at M6
Control Group Level
Range of thorax obliquity (deg)
Range of flexion pathological hip (deg)
Peak vertical velocity thorax (deg)
Peak thorax obliquity (deg)
Peak thorax flexion (deg)
T2S (s)
Turn (s)
Walk (s)
S2S (s)
Total (s)
Peak angular velocity thorax (deg/s)
Distance chair to start turn (m)
Range thorax obliquity (deg)
Mean angular velocity pelvis (deg/s)
Steps Number
Peak forward velocity pelvis (m/s)
Walk
S2S
Time
T2S
Turn
3.8
50.5
1.1
1.2
64.6
2.2
1.1
2.8
0.6
8.3
257.3
1.6
8.0
163.4
2.0
1.9
35.0
11.9
0.2
14.2
14.3
8.5
4.2
17.4
2.9
29.1
83.1
0.5
48.4
41.4
6.0
0.5

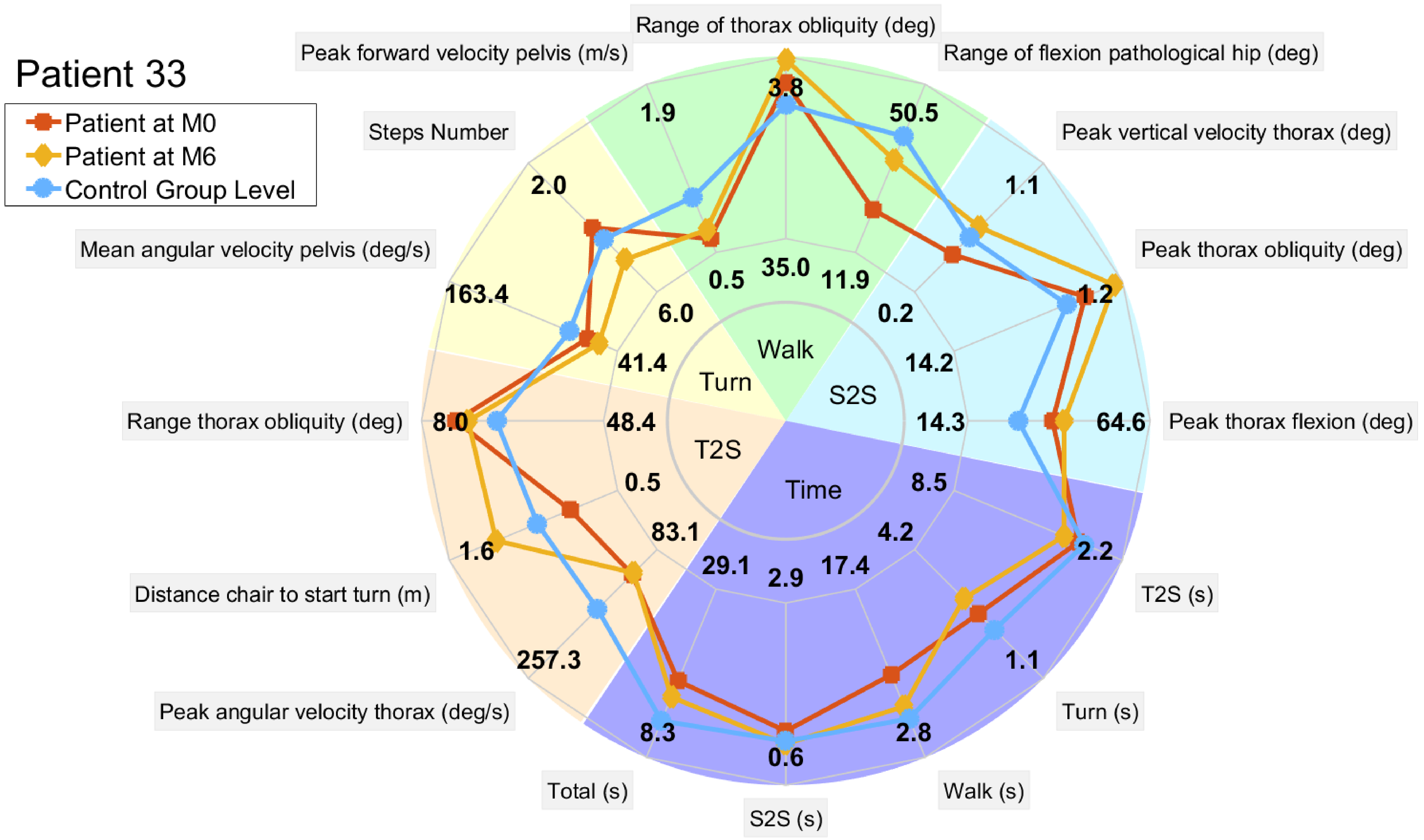

Patient 33
Patient at M0
Patient at M6
Control Group Level
Range of thorax obliquity (deg)
Range of flexion pathological hip (deg)
Peak vertical velocity thorax (deg)
Peak thorax obliquity (deg)
Peak thorax flexion (deg)
T2S (s)
Turn (s)
Walk (s)
S2S (s)
Total (s)
Peak angular velocity thorax (deg/s)
Distance chair to start turn (m)
Range thorax obliquity (deg)
Mean angular velocity pelvis (deg/s)
Steps Number
Peak forward velocity pelvis (m/s)
Walk
S2S
Time
T2S
Turn
3.8
50.5
1.1
1.2
64.6
2.2
1.1
2.8
0.6
8.3
257.3
1.6
8.0
163.4
2.0
1.9
35.0
11.9
0.2
14.2
14.3
8.5
4.2
17.4
2.9
29.1
83.1
0.5
48.4
41.4
6.0
0.5

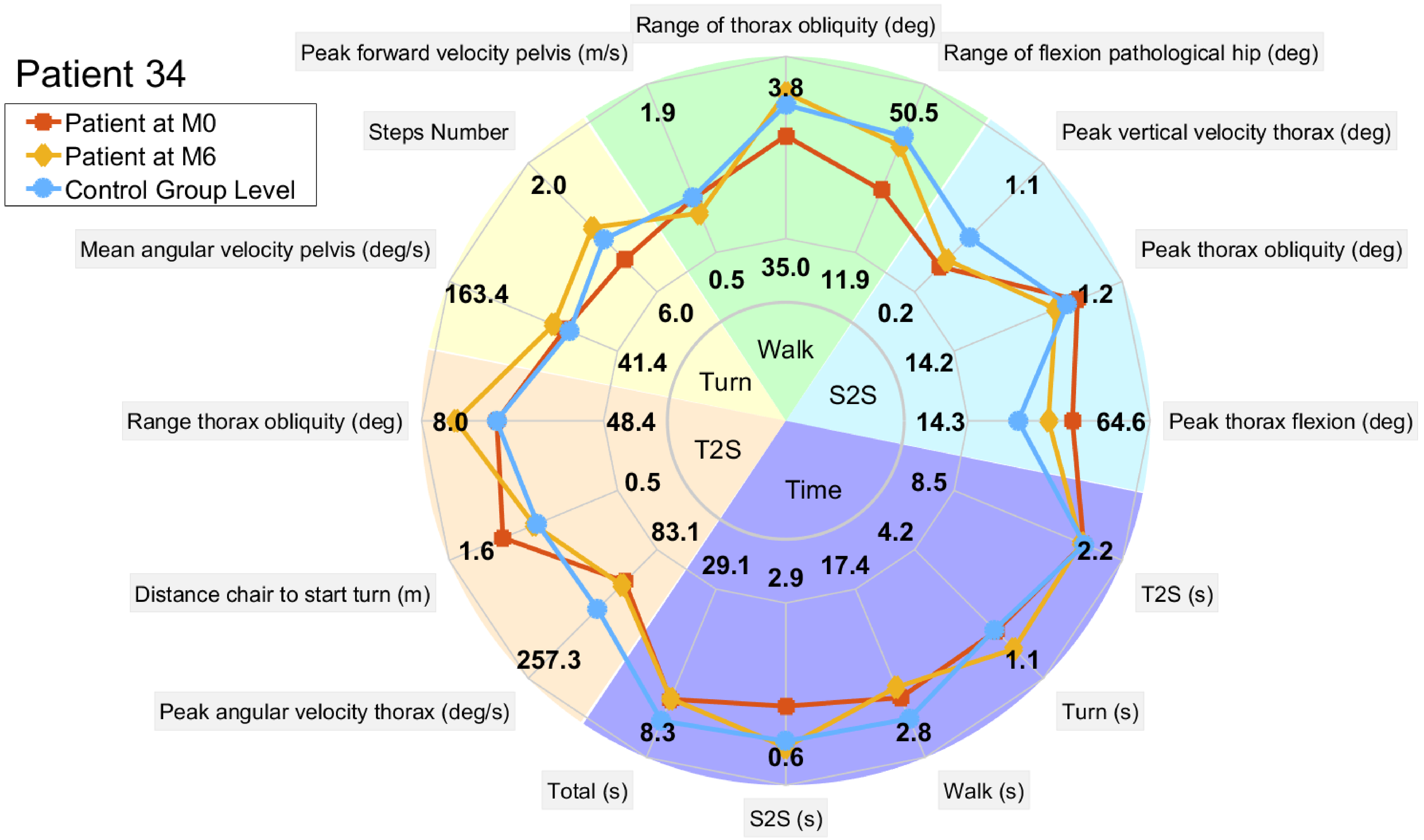

Patient 34
Patient at M0
Patient at M6
Control Group Level
Range of thorax obliquity (deg)
Range of flexion pathological hip (deg)
Peak vertical velocity thorax (deg)
Peak thorax obliquity (deg)
Peak thorax flexion (deg)
T2S (s)
Turn (s)
Walk (s)
S2S (s)
Total (s)
Peak angular velocity thorax (deg/s)
Distance chair to start turn (m)
Range thorax obliquity (deg)
Mean angular velocity pelvis (deg/s)
Steps Number
Peak forward velocity pelvis (m/s)
Walk
Turn
S2S
T2S
Time
3.8
50.5
1.9
1.1
2.0
1.2
163.4
64.6
8.0
2.2
1.6
1.1
257.3
2.8
8.3
0.6
35.0
11.9
0.5
0.2
6.0
14.2
41.4
14.3
48.4
0.5
8.5
83.1
4.2
29.1
2.9
17.4

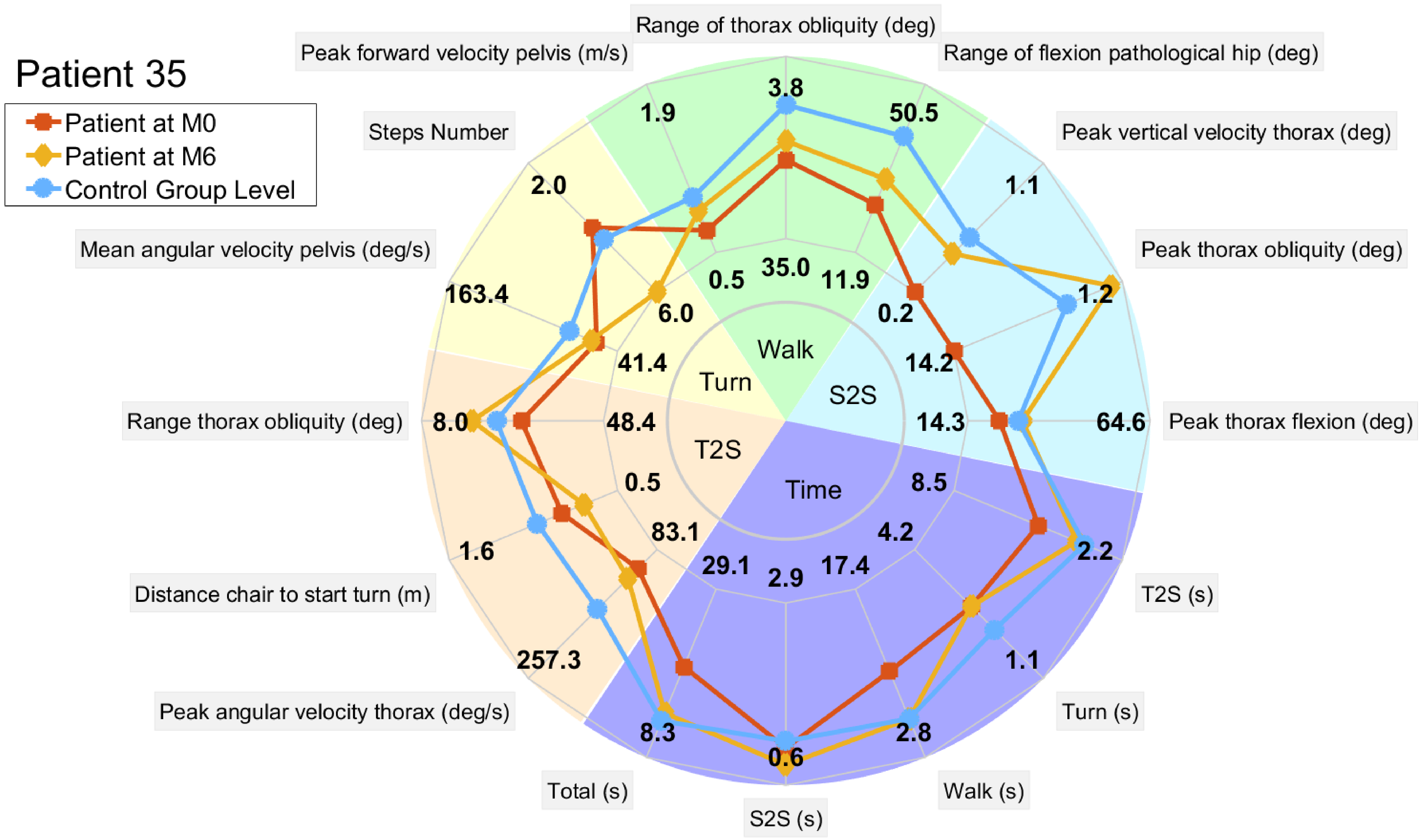

Patient 35
Patient at M0
Patient at M6
Control Group Level
Range of thorax obliquity (deg)
Peak forward velocity pelvis (m/s)
Range of flexion pathological hip (deg)
Steps Number
Peak vertical velocity thorax (deg)
Mean angular velocity pelvis (deg/s)
Peak thorax obliquity (deg)
Range thorax obliquity (deg)
Peak thorax flexion (deg)
Distance chair to start turn (m)
T2S (s)
Peak angular velocity thorax (deg/s)
Turn (s)
Total (s)
Walk (s)
S2S (s)
3.8
1.9
50.5
2.0
1.1
163.4
1.2
8.0
64.6
1.6
2.2
257.3
1.1
8.3
2.8
0.6
0.5
35.0
11.9
6.0
0.2
41.4
14.2
48.4
14.3
0.5
8.5
83.1
4.2
29.1
2.9
17.4
Walk
Turn
S2S
T2S
Time

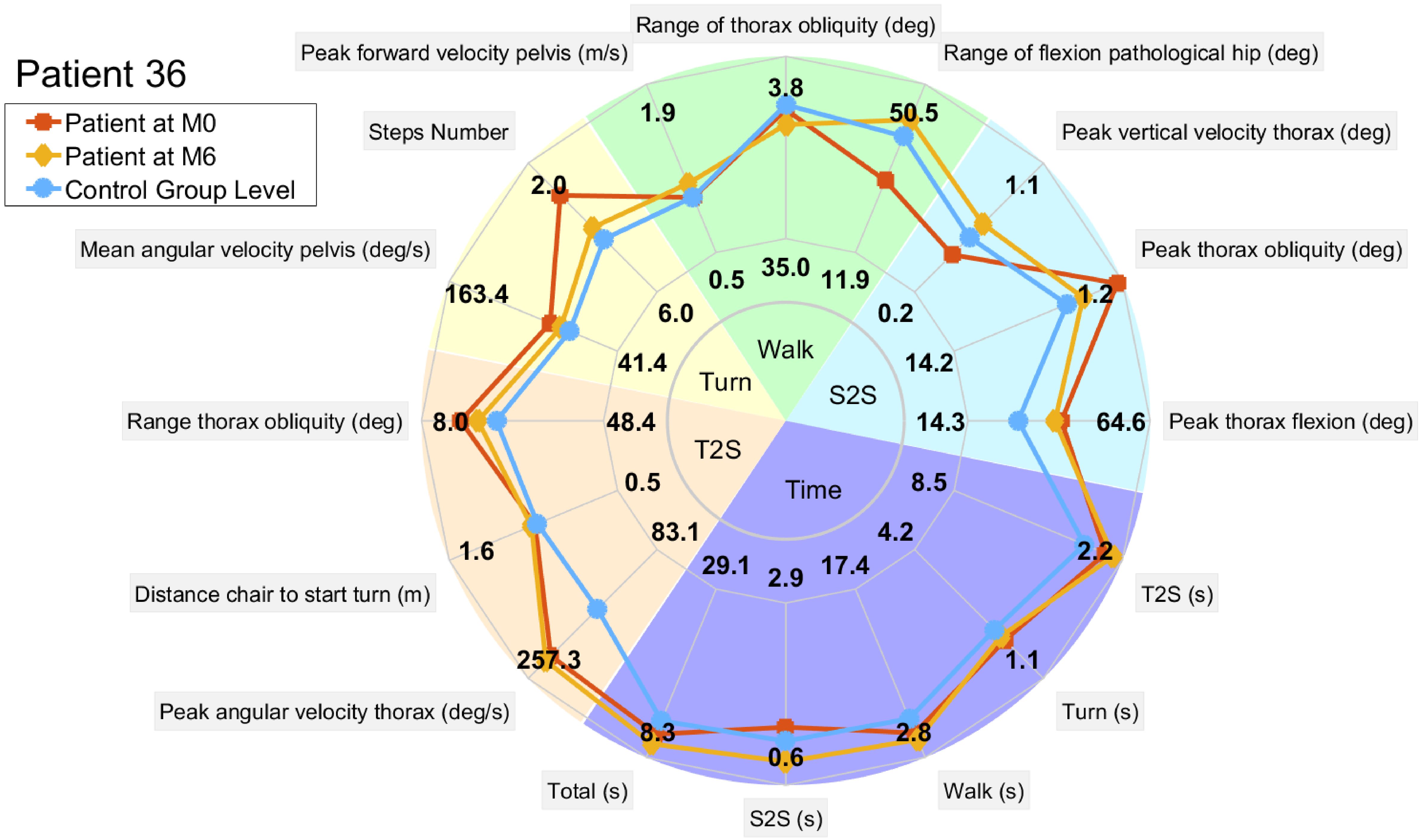

Patient 36
Patient at M0
Patient at M6
Control Group Level
Range of thorax obliquity (deg)
Range of flexion pathological hip (deg)
Peak vertical velocity thorax (deg)
Peak thorax obliquity (deg)
Peak thorax flexion (deg)
T2S (s)
Turn (s)
Walk (s)
S2S (s)
Total (s)
Peak angular velocity thorax (deg/s)
Distance chair to start turn (m)
Range thorax obliquity (deg)
Mean angular velocity pelvis (deg/s)
Steps Number
Peak forward velocity pelvis (m/s)
Walk
S2S
Time
T2S
Turn
3.8
50.5
1.1
1.2
64.6
2.2
1.1
2.8
0.6
8.3
257.3
1.6
8.0
163.4
2.0
1.9
35.0
11.9
0.2
14.2
14.3
8.5
4.2
17.4
2.9
29.1
83.1
0.5
48.4
41.4
6.0
0.5

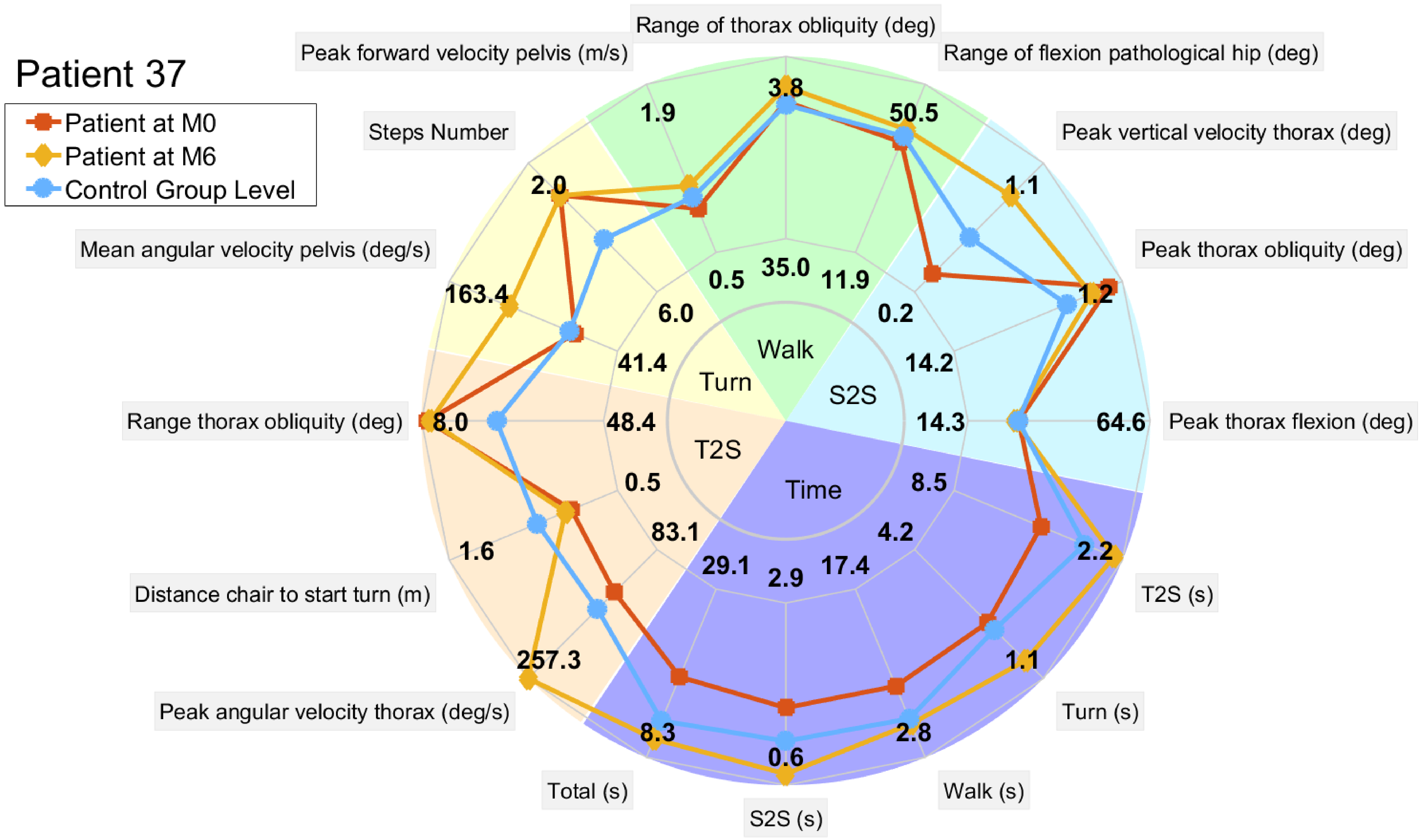

Patient 37
Patient at M0
Patient at M6
Control Group Level
Range of thorax obliquity (deg)
Range of flexion pathological hip (deg)
Peak forward velocity pelvis (m/s)
Steps Number
Peak vertical velocity thorax (deg)
Peak thorax obliquity (deg)
Mean angular velocity pelvis (deg/s)
Range thorax obliquity (deg)
Peak thorax flexion (deg)
Distance chair to start turn (m)
T2S (s)
Peak angular velocity thorax (deg/s)
Turn (s)
Total (s)
Walk (s)
S2S (s)
Walk
Turn
S2S
T2S
Time
3.8
50.5
1.9
2.0
1.1
1.2
163.4
8.0
64.6
1.6
2.2
257.3
1.1
8.3
2.8
0.6
35.0
0.5
11.9
6.0
0.2
41.4
14.2
48.4
14.3
0.5
8.5
83.1
4.2
29.1
2.9
17.4

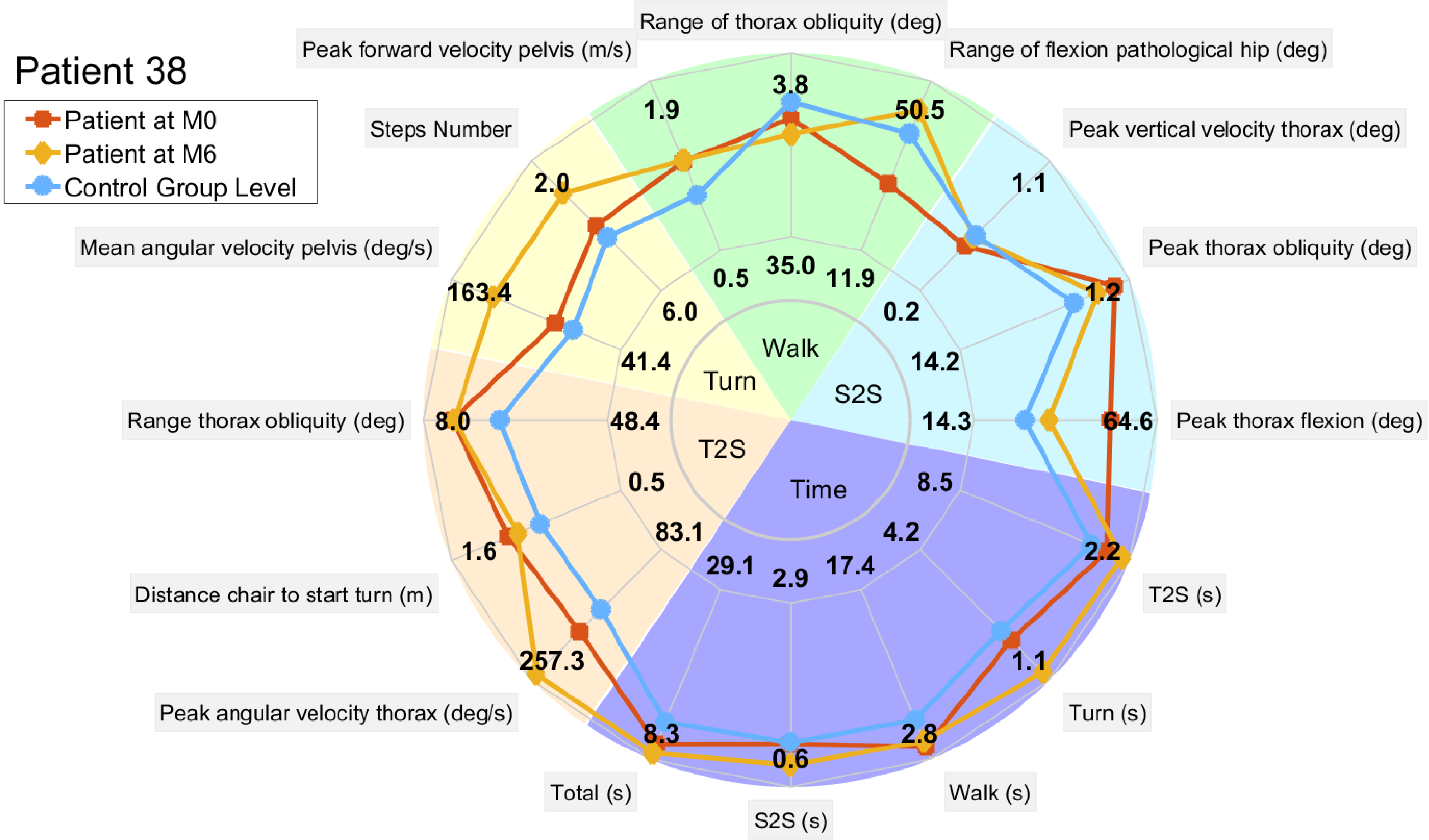
Patient 38
Patient at M0
Patient at M6
Control Group Level
Range of thorax obliquity (deg)
Range of flexion pathological hip (deg)
Peak vertical velocity thorax (deg)
Peak thorax obliquity (deg)
Peak thorax flexion (deg)
T2S (s)
Turn (s)
Walk (s)
S2S (s)
Total (s)
Peak angular velocity thorax (deg/s)
Distance chair to start turn (m)
Range thorax obliquity (deg)
Mean angular velocity pelvis (deg/s)
Steps Number
Peak forward velocity pelvis (m/s)
Walk
Turn
S2S
T2S
Time
3.8
50.5
1.1
1.2
64.6
2.2
1.1
2.8
0.6
8.3
257.3
1.6
8.0
163.4
2.0
1.9
35.0
11.9
0.2
14.2
14.3
8.5
4.2
17.4
2.9
29.1
83.1
0.5
48.4
41.4
6.0
0.5

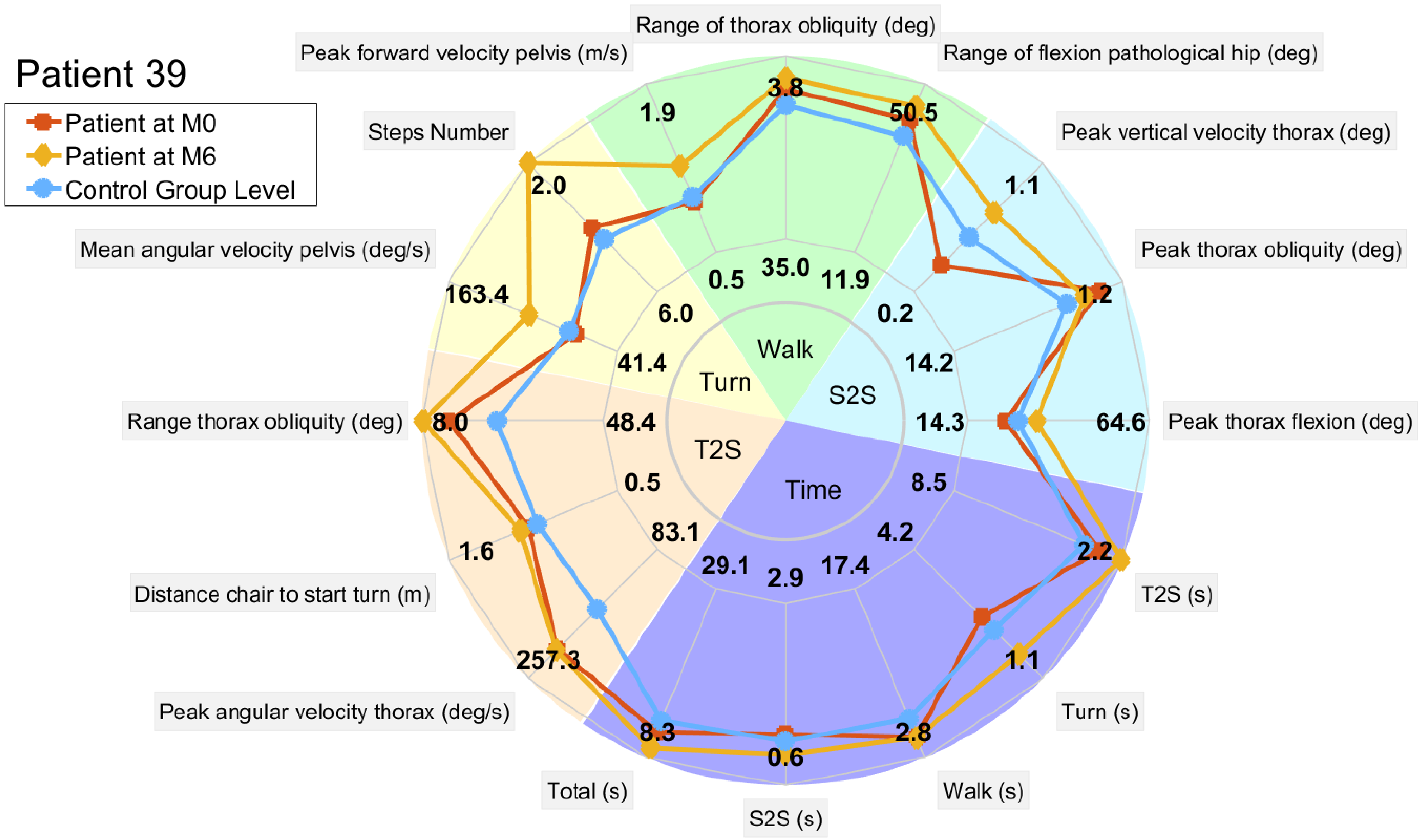
Patient 39
Patient at M0
Patient at M6
Control Group Level
Range of thorax obliquity (deg)
Range of flexion pathological hip (deg)
Peak vertical velocity thorax (deg)
Peak thorax obliquity (deg)
Peak thorax flexion (deg)
T2S (s)
Turn (s)
Walk (s)
S2S (s)
Total (s)
Peak angular velocity thorax (deg/s)
Distance chair to start turn (m)
Range thorax obliquity (deg)
Mean angular velocity pelvis (deg/s)
Steps Number
Peak forward velocity pelvis (m/s)
Walk
Turn
S2S
T2S
Time
3.8
50.5
1.1
1.2
64.6
2.2
1.1
2.8
0.6
8.3
257.3
1.6
8.0
163.4
2.0
1.9
35.0
11.9
0.2
14.2
14.3
8.5
4.2
17.4
2.9
29.1
83.1
0.5
48.4
41.4
6.0
0.5

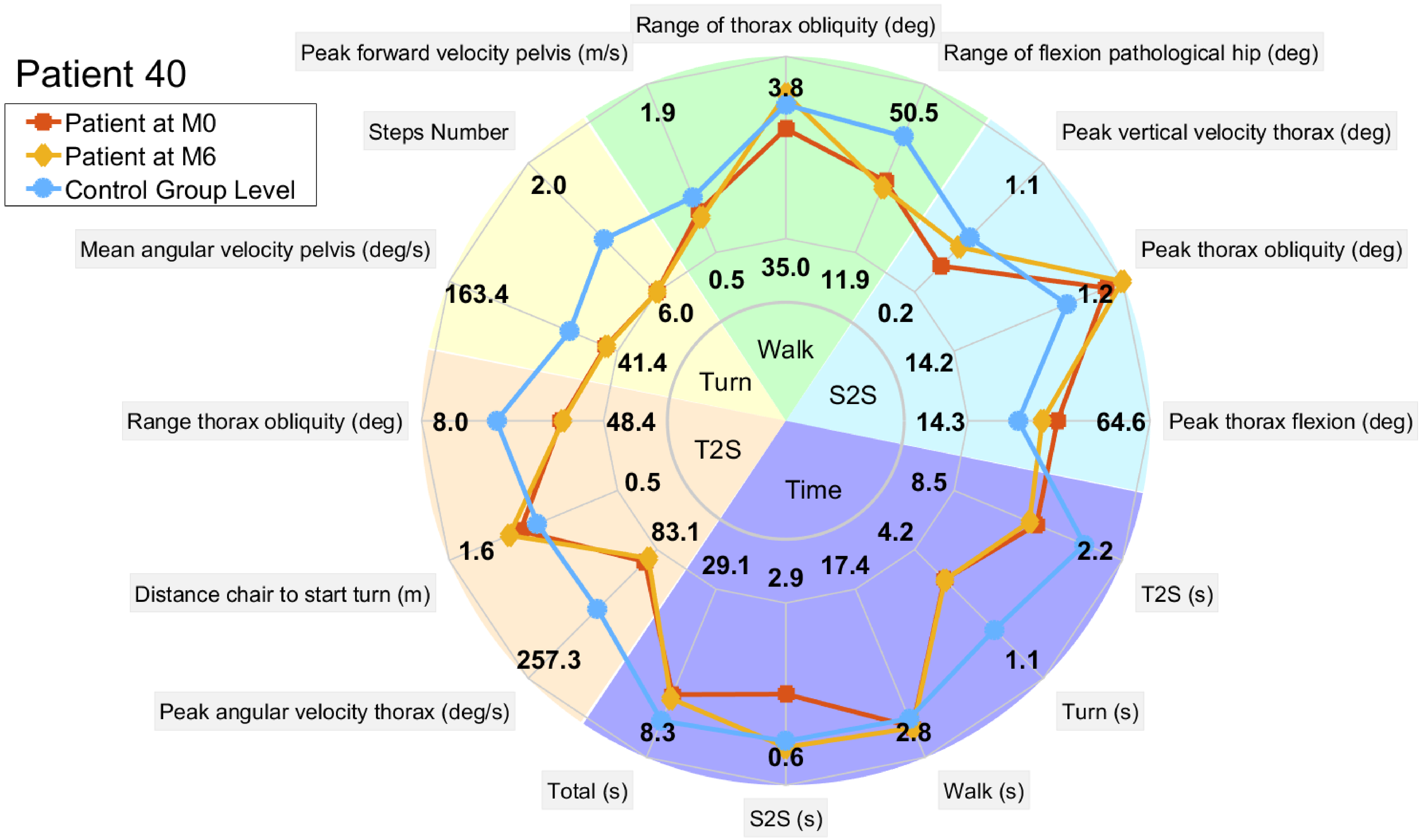
Patient 40
Patient at M0
Patient at M6
Control Group Level
Range of thorax obliquity (deg)
Peak forward velocity pelvis (m/s)
Range of flexion pathological hip (deg)
Steps Number
Peak vertical velocity thorax (deg)
Mean angular velocity pelvis (deg/s)
Peak thorax obliquity (deg)
Range thorax obliquity (deg)
Peak thorax flexion (deg)
Distance chair to start turn (m)
T2S (s)
Peak angular velocity thorax (deg/s)
Turn (s)
Total (s)
S2S (s)
Walk (s)
Walk
Turn
S2S
T2S
Time
3.8
1.9
50.5
2.0
1.1
163.4
1.2
8.0
64.6
1.6
2.2
257.3
1.1
8.3
0.6
2.8
0.5
35.0
11.9
6.0
0.2
41.4
14.2
48.4
14.3
0.5
8.5
83.1
4.2
29.1
2.9
17.4

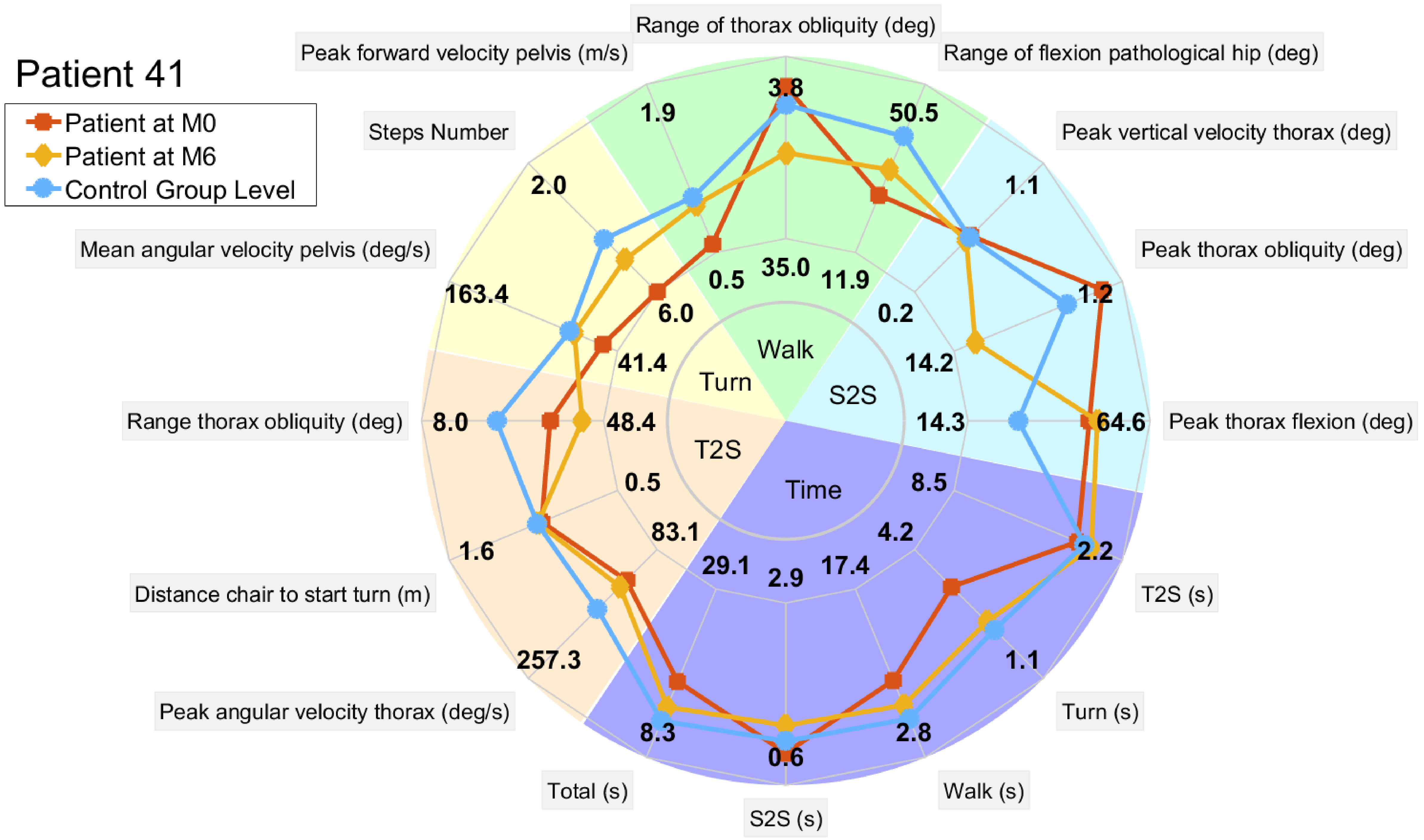
Patient 41
Patient at M0
Patient at M6
Control Group Level
Range of thorax obliquity (deg)
Peak forward velocity pelvis (m/s)
Range of flexion pathological hip (deg)
Steps Number
Peak vertical velocity thorax (deg)
Peak thorax obliquity (deg)
Mean angular velocity pelvis (deg/s)
Range thorax obliquity (deg)
Peak thorax flexion (deg)
Distance chair to start turn (m)
T2S (s)
Turn (s)
Peak angular velocity thorax (deg/s)
Total (s)
Walk (s)
S2S (s)
Walk
Turn
S2S
T2S
Time
3.8
1.9
50.5
2.0
1.1
163.4
1.2
8.0
64.6
1.6
2.2
257.3
1.1
8.3
2.8
0.6
0.5
35.0
11.9
6.0
0.2
41.4
14.2
48.4
14.3
0.5
8.5
83.1
4.2
29.1
2.9
17.4

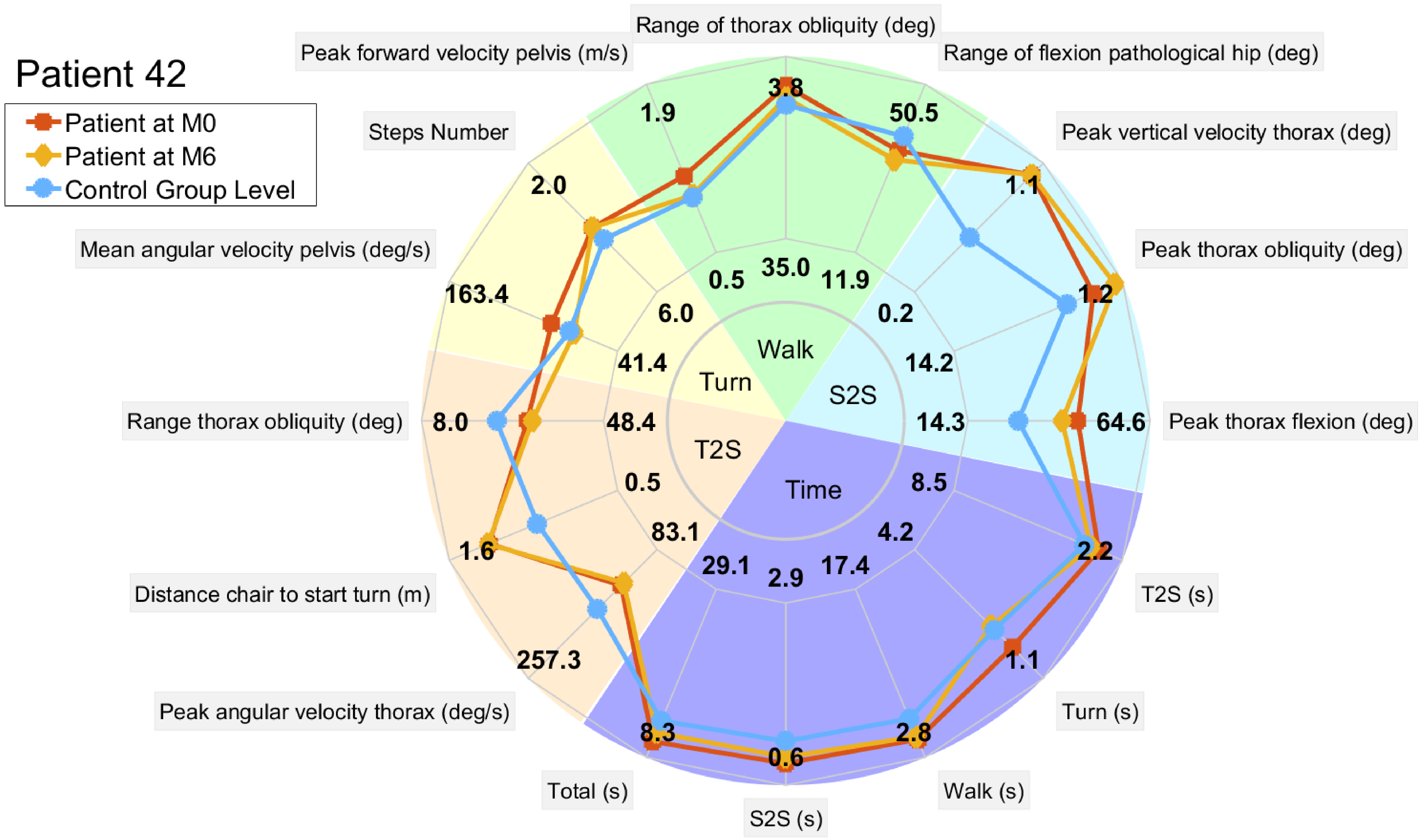

Patient 42
Patient at M0
Patient at M6
Control Group Level
Range of thorax obliquity (deg)
Range of flexion pathological hip (deg)
Peak vertical velocity thorax (deg)
Peak thorax obliquity (deg)
Peak thorax flexion (deg)
T2S (s)
Turn (s)
Walk (s)
S2S (s)
Total (s)
Peak angular velocity thorax (deg/s)
Distance chair to start turn (m)
Range thorax obliquity (deg)
Mean angular velocity pelvis (deg/s)
Steps Number
Peak forward velocity pelvis (m/s)
Walk
S2S
Time
T2S
Turn
3.8
50.5
1.1
1.2
64.6
2.2
1.1
2.8
0.6
8.3
257.3
1.6
8.0
163.4
2.0
1.9
35.0
11.9
0.2
14.2
14.3
8.5
4.2
17.4
2.9
29.1
83.1
0.5
48.4
41.4
6.0
0.5

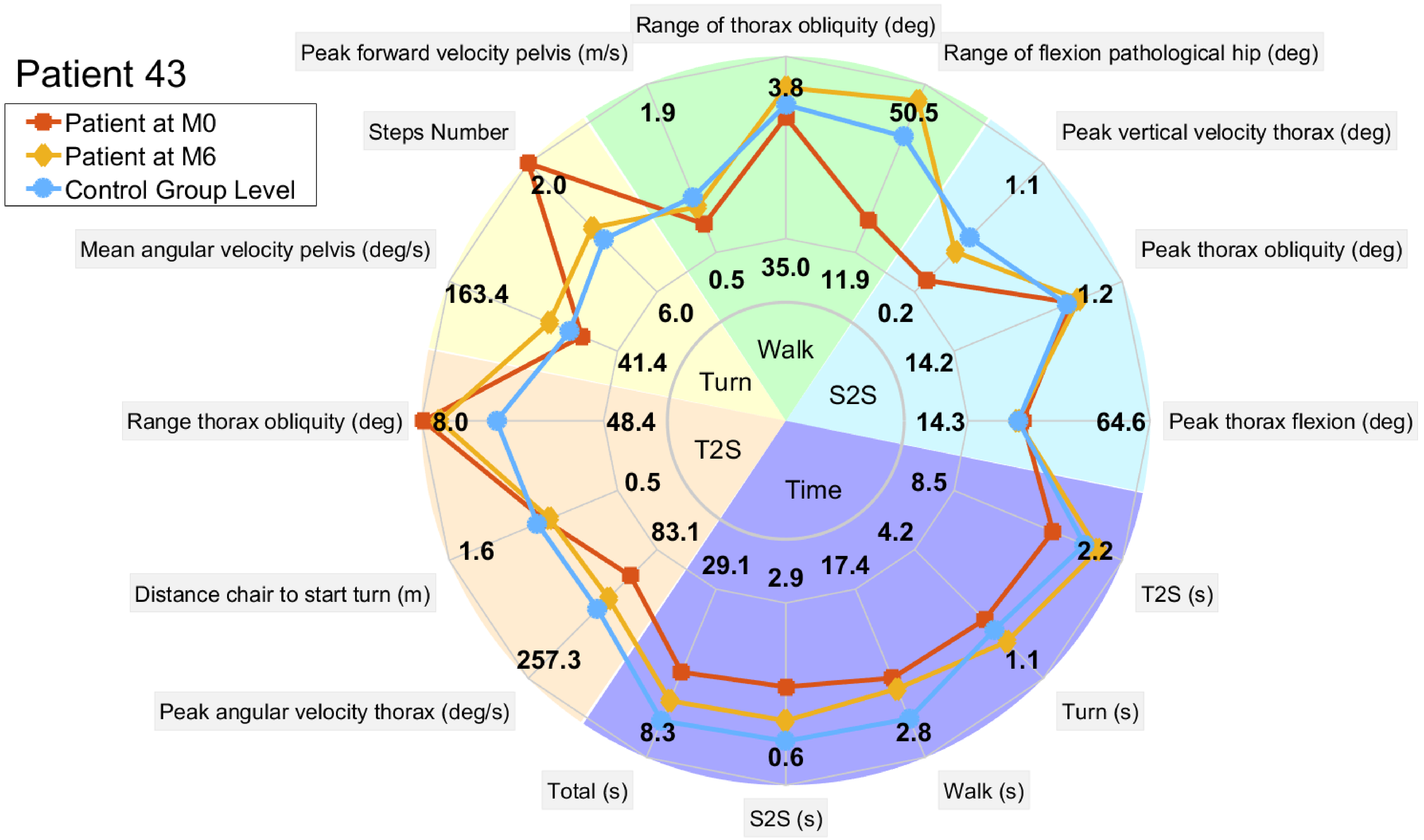

Patient 43
Patient at M0
Patient at M6
Control Group Level
Range of thorax obliquity (deg)
Range of flexion pathological hip (deg)
Peak vertical velocity thorax (deg)
Peak thorax obliquity (deg)
Peak thorax flexion (deg)
T2S (s)
Turn (s)
Walk (s)
S2S (s)
Total (s)
Peak angular velocity thorax (deg/s)
Distance chair to start turn (m)
Range thorax obliquity (deg)
Mean angular velocity pelvis (deg/s)
Steps Number
Peak forward velocity pelvis (m/s)
Walk
S2S
Time
T2S
Turn
3.8
35.0
50.5
11.9
1.1
0.2
1.2
14.2
64.6
14.3
2.2
8.5
1.1
4.2
2.8
17.4
0.6
2.9
8.3
29.1
257.3
83.1
1.6
0.5
8.0
48.4
163.4
41.4
2.0
6.0
1.9
0.5

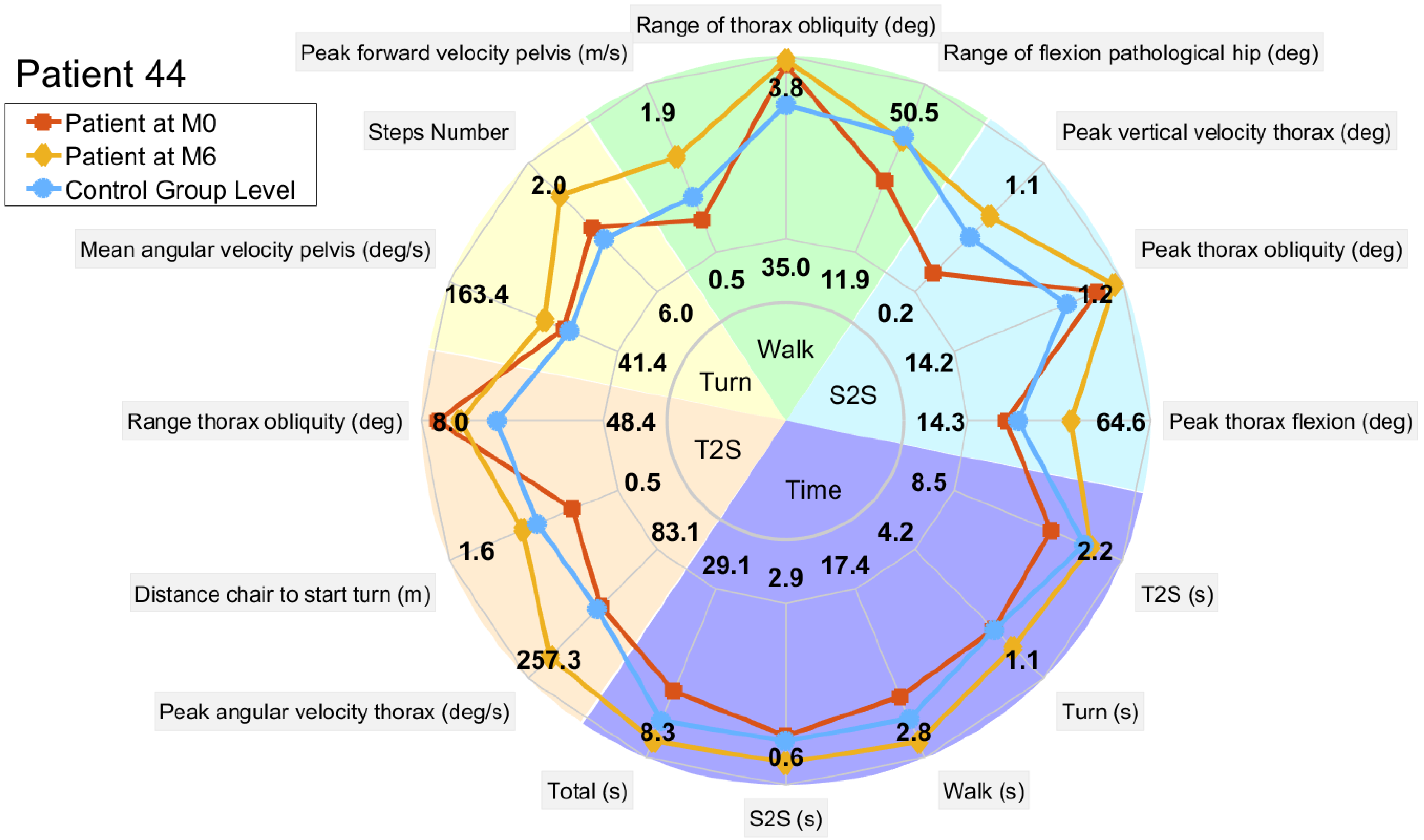

Patient 44
Patient at M0
Patient at M6
Control Group Level
Range of thorax obliquity (deg)
Range of flexion pathological hip (deg)
Peak vertical velocity thorax (deg)
Peak thorax obliquity (deg)
Peak thorax flexion (deg)
T2S (s)
Turn (s)
Walk (s)
S2S (s)
Total (s)
Peak angular velocity thorax (deg/s)
Distance chair to start turn (m)
Range thorax obliquity (deg)
Mean angular velocity pelvis (deg/s)
Steps Number
Peak forward velocity pelvis (m/s)
Walk
S2S
Time
T2S
Turn
3.8
50.5
1.1
1.2
64.6
2.2
1.1
2.8
0.6
8.3
257.3
1.6
8.0
163.4
2.0
1.9
35.0
11.9
0.2
14.2
14.3
8.5
4.2
17.4
2.9
29.1
83.1
0.5
48.4
41.4
6.0
0.5

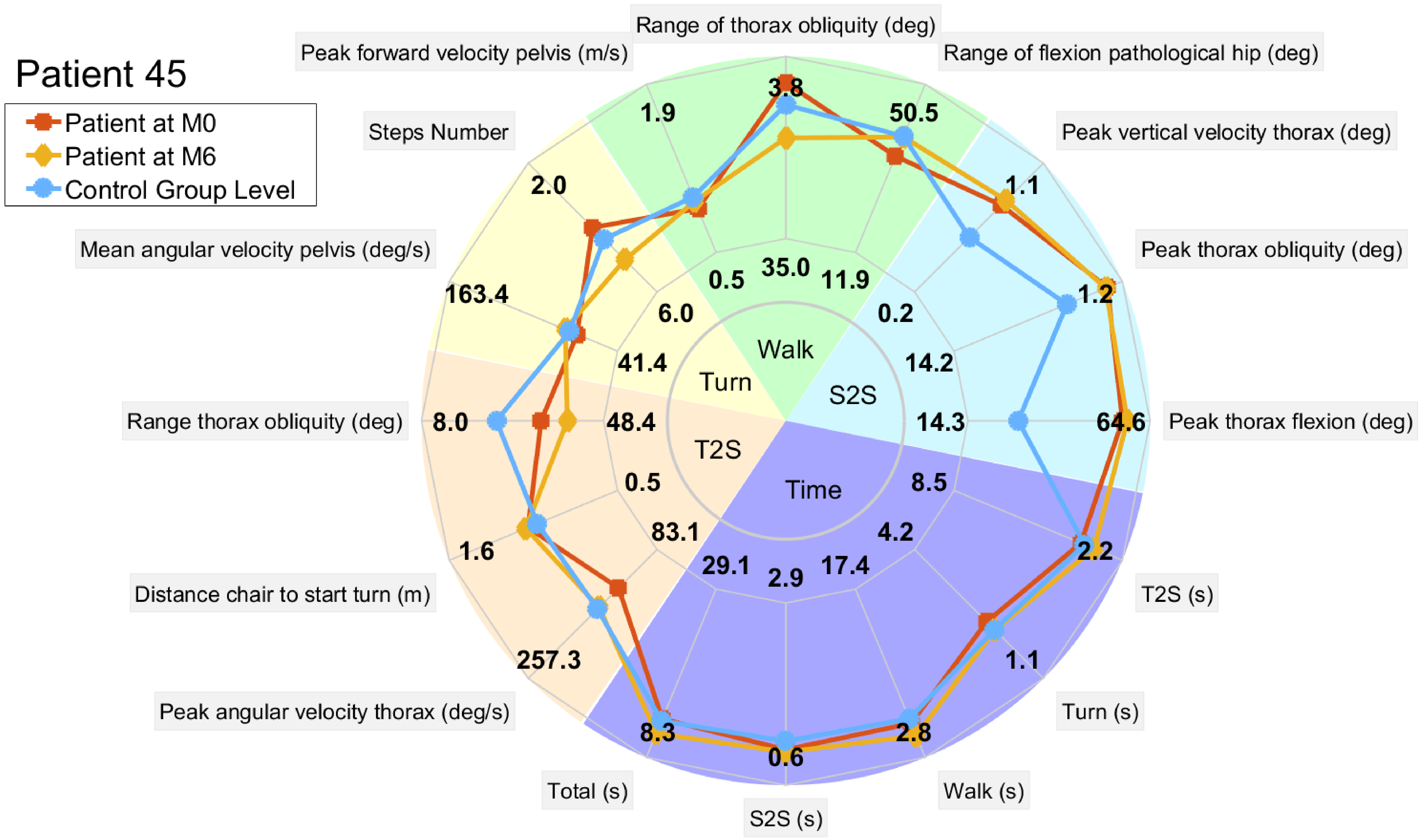
Patient 45
Patient at M0
Patient at M6
Control Group Level
Range of thorax obliquity (deg)
Range of flexion pathological hip (deg)
Peak vertical velocity thorax (deg)
Peak thorax obliquity (deg)
Peak thorax flexion (deg)
T2S (s)
Turn (s)
Walk (s)
S2S (s)
Total (s)
Peak angular velocity thorax (deg/s)
Distance chair to start turn (m)
Range thorax obliquity (deg)
Mean angular velocity pelvis (deg/s)
Steps Number
Peak forward velocity pelvis (m/s)
Walk
Turn
S2S
T2S
Time
3.8
50.5
1.1
1.2
64.6
2.2
1.1
2.8
0.6
8.3
257.3
1.6
8.0
163.4
2.0
1.9
0.5
35.0
11.9
0.2
14.2
14.3
8.5
4.2
17.4
2.9
29.1
83.1
0.5
48.4
41.4
6.0

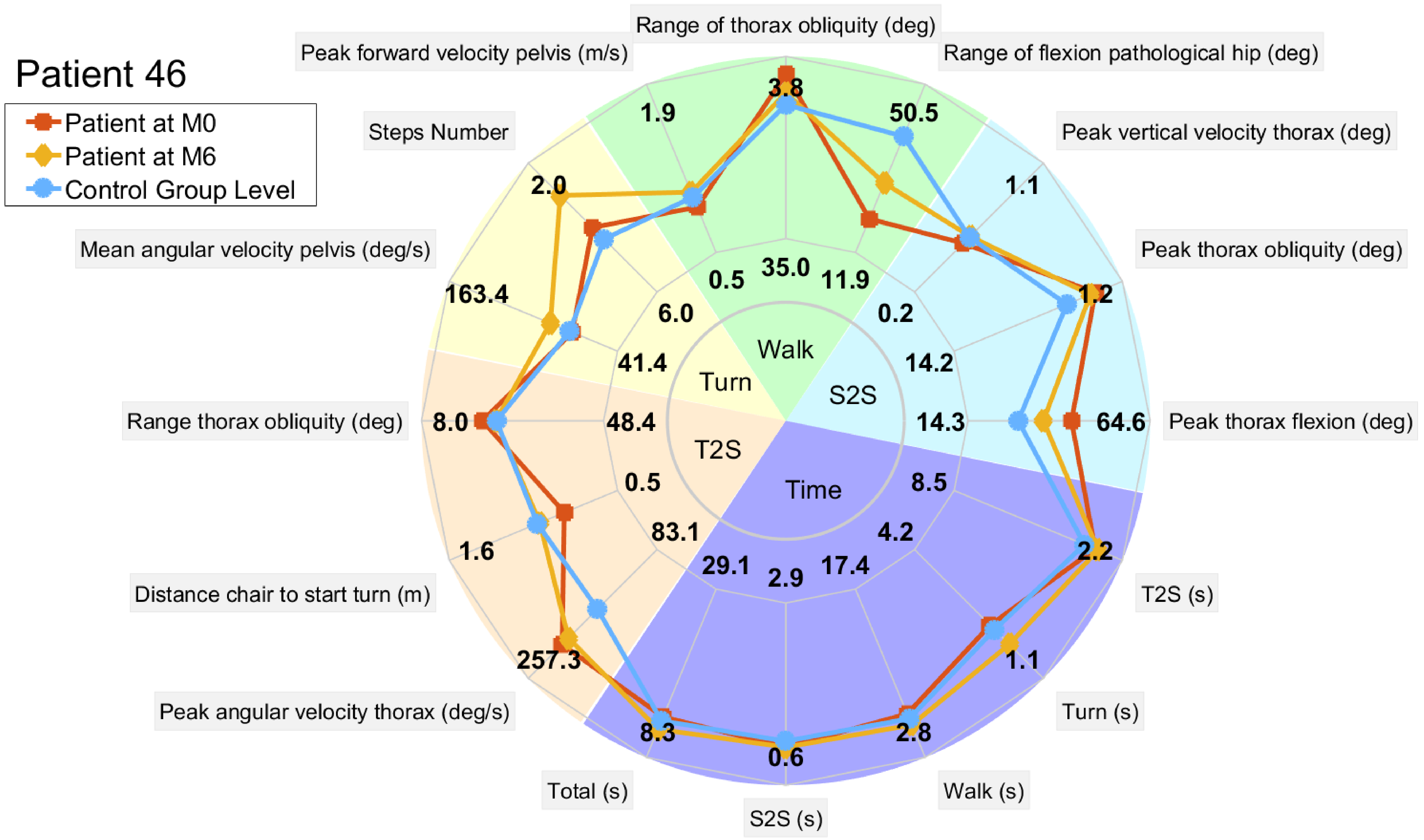
Patient 46
Patient at M0
Patient at M6
Control Group Level
Range of thorax obliquity (deg)
Range of flexion pathological hip (deg)
Peak vertical velocity thorax (deg)
Peak thorax obliquity (deg)
Peak thorax flexion (deg)
T2S (s)
Turn (s)
Walk (s)
S2S (s)
Total (s)
Peak angular velocity thorax (deg/s)
Distance chair to start turn (m)
Range thorax obliquity (deg)
Mean angular velocity pelvis (deg/s)
Steps Number
Peak forward velocity pelvis (m/s)
Walk
S2S
Time
T2S
Turn
3.8
50.5
1.1
1.2
64.6
2.2
1.1
2.8
0.6
8.3
257.3
1.6
8.0
163.4
2.0
1.9
0.5
35.0
11.9
0.2
14.2
14.3
8.5
4.2
17.4
2.9
29.1
83.1
0.5
48.4
41.4
6.0

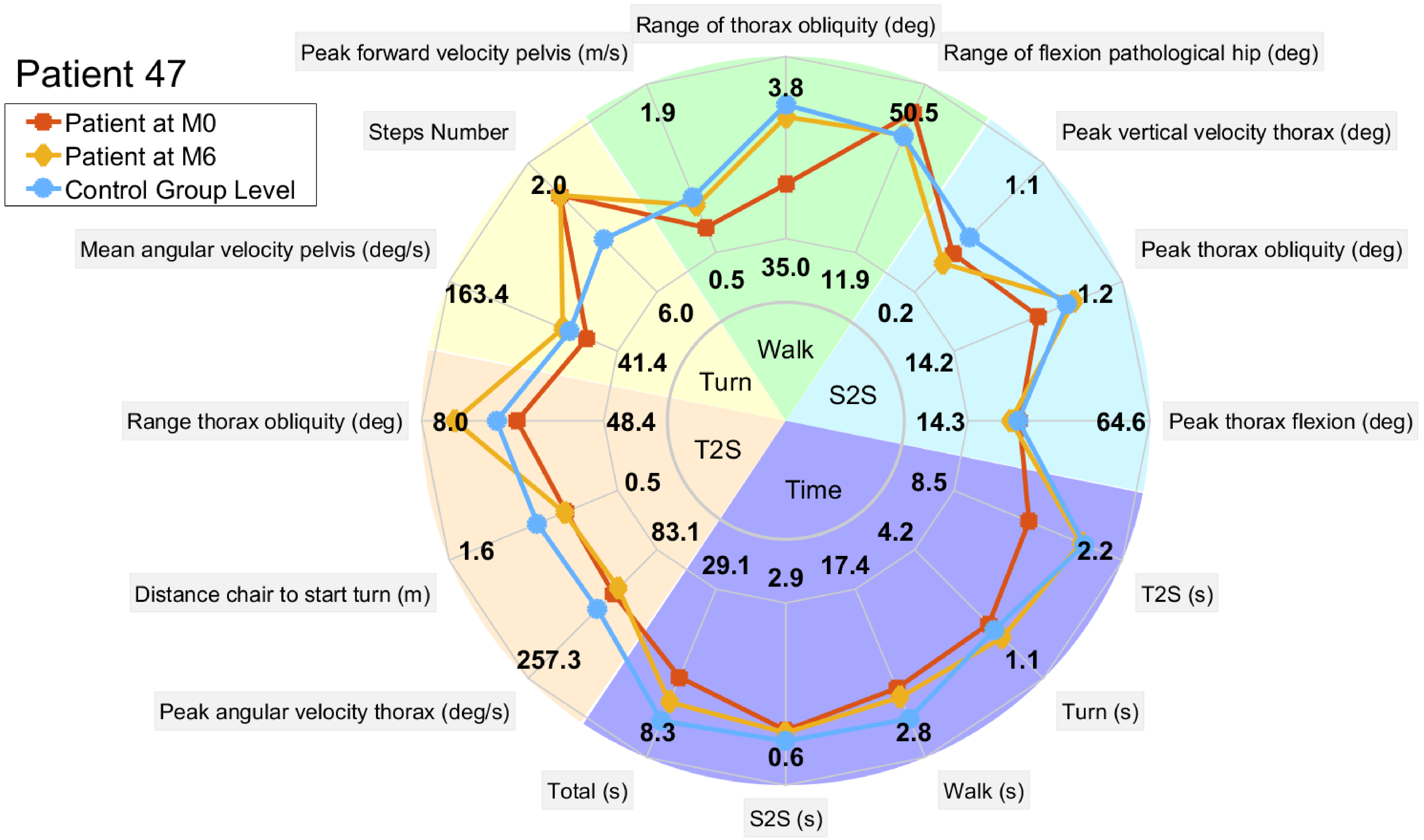

Patient 47
Patient at M0
Patient at M6
Control Group Level
Range of thorax obliquity (deg)
Peak forward velocity pelvis (m/s)
Range of flexion pathological hip (deg)
Steps Number
Peak vertical velocity thorax (deg)
Mean angular velocity pelvis (deg/s)
Peak thorax obliquity (deg)
Range thorax obliquity (deg)
Peak thorax flexion (deg)
Distance chair to start turn (m)
T2S (s)
Peak angular velocity thorax (deg/s)
Turn (s)
Total (s)
Walk (s)
S2S (s)
Walk
Turn
S2S
T2S
Time
3.8
1.9
50.5
2.0
1.1
163.4
1.2
8.0
64.6
1.6
2.2
257.3
1.1
8.3
0.6
2.8
35.0
0.5
11.9
6.0
0.2
41.4
14.2
48.4
14.3
0.5
8.5
83.1
4.2
29.1
2.9
17.4

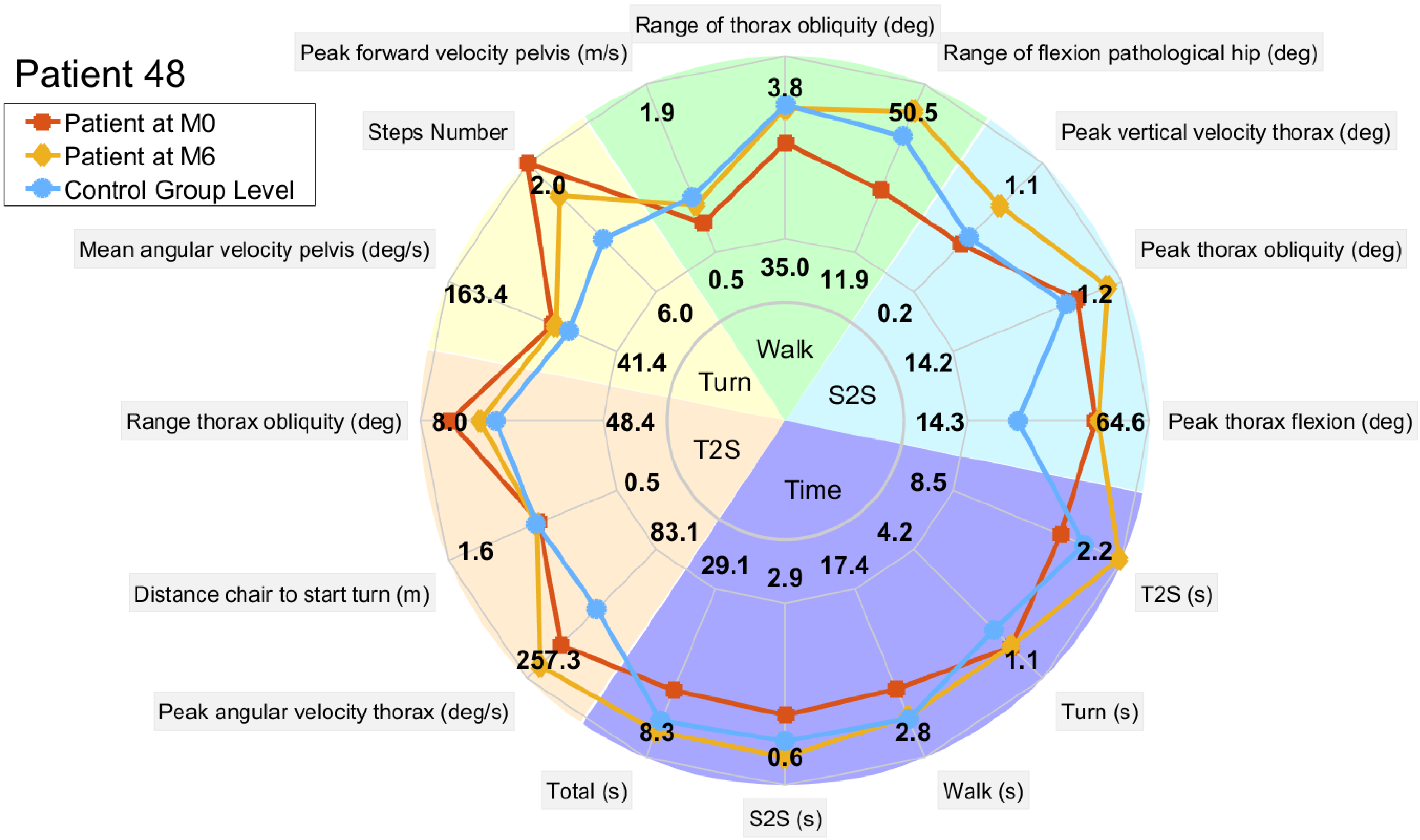
Patient 48
Patient at M0
Patient at M6
Control Group Level
Range of thorax obliquity (deg)
Range of flexion pathological hip (deg)
Peak vertical velocity thorax (deg)
Peak thorax obliquity (deg)
Peak thorax flexion (deg)
T2S (s)
Turn (s)
Walk (s)
S2S (s)
Total (s)
Peak angular velocity thorax (deg/s)
Distance chair to start turn (m)
Range thorax obliquity (deg)
Mean angular velocity pelvis (deg/s)
Steps Number
Peak forward velocity pelvis (m/s)
Walk
S2S
Time
T2S
Turn
3.8
50.5
1.1
1.2
64.6
2.2
1.1
2.8
0.6
8.3
257.3
1.6
8.0
163.4
2.0
1.9
35.0
11.9
0.2
14.2
14.3
8.5
4.2
17.4
2.9
29.1
83.1
0.5
48.4
41.4
6.0
0.5

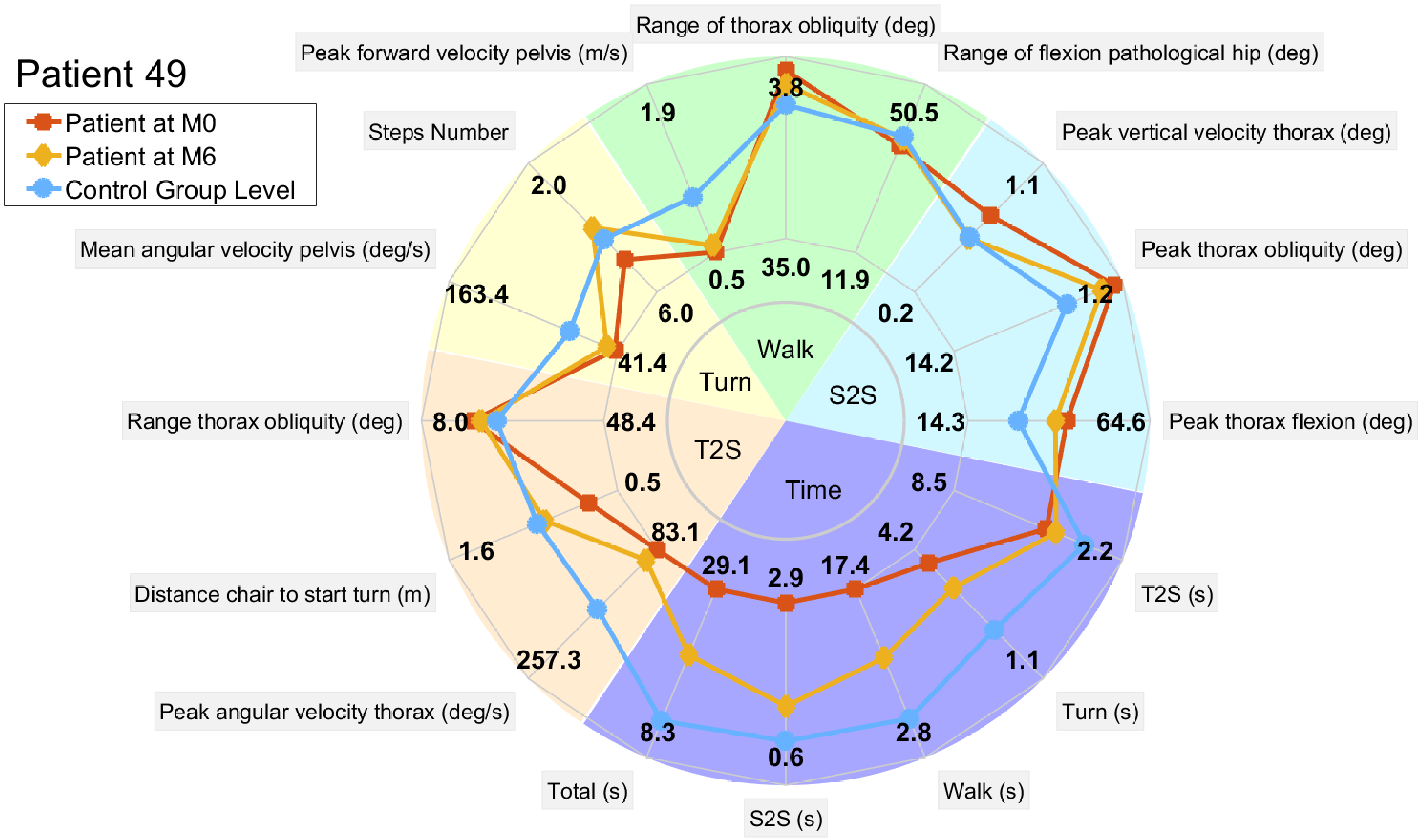
Patient 49
Patient at M0
Patient at M6
Control Group Level
Range of thorax obliquity (deg)
Peak forward velocity pelvis (m/s)
Range of flexion pathological hip (deg)
Steps Number
Peak vertical velocity thorax (deg)
Mean angular velocity pelvis (deg/s)
Peak thorax obliquity (deg)
Range thorax obliquity (deg)
Peak thorax flexion (deg)
Distance chair to start turn (m)
T2S (s)
Peak angular velocity thorax (deg/s)
Turn (s)
Total (s)
Walk (s)
S2S (s)
Walk
Turn
S2S
T2S
Time
3.8
1.9
50.5
2.0
1.1
0.5
35.0
11.9
163.4
6.0
0.2
1.2
41.4
14.2
8.0
48.4
14.3
64.6
0.5
8.5
1.6
83.1
4.2
2.2
29.1
2.9
17.4
257.3
1.1
8.3
0.6
2.8

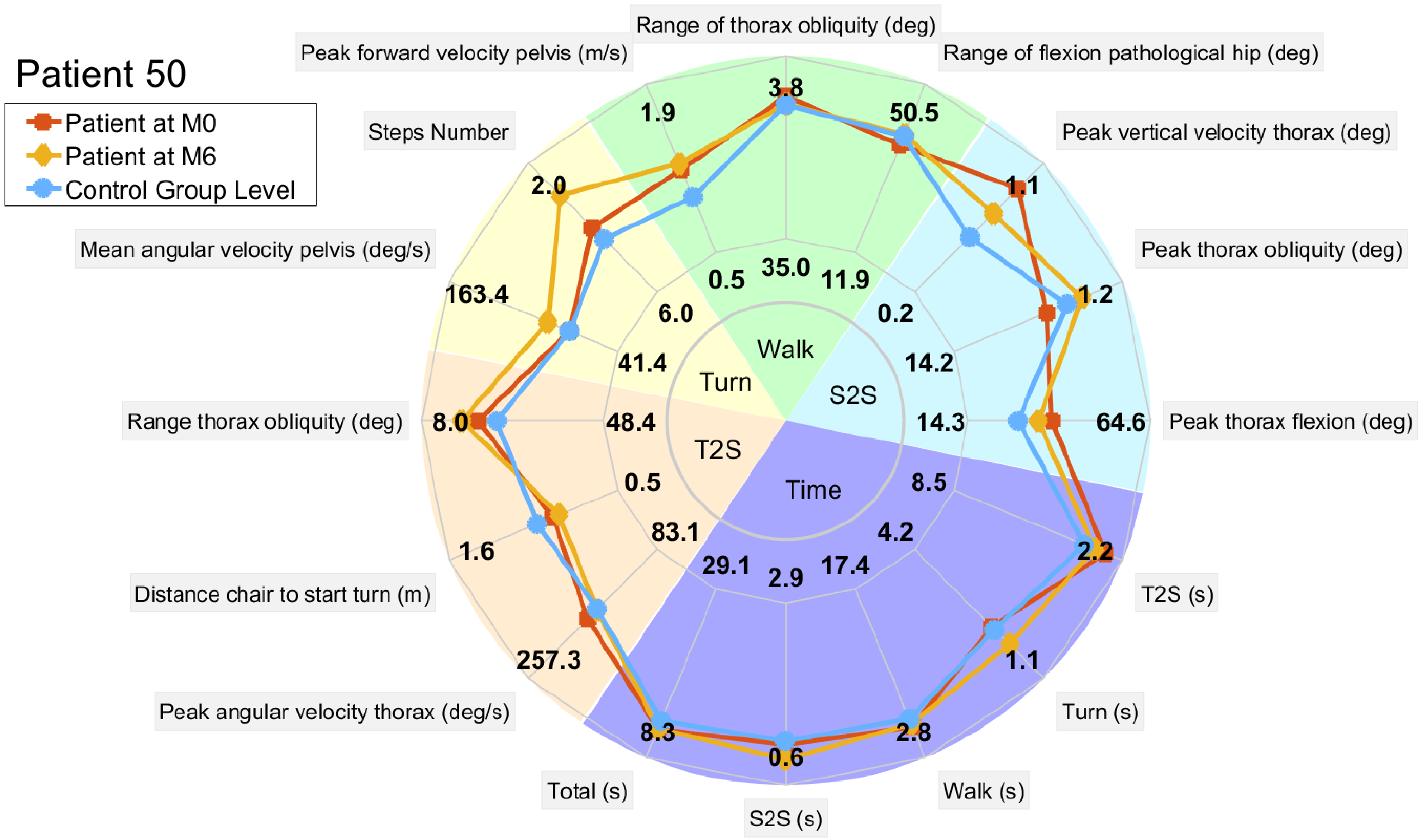

Patient 50
Patient at M0
Patient at M6
Control Group Level
Range of thorax obliquity (deg)
Range of flexion pathological hip (deg)
Peak vertical velocity thorax (deg)
Peak thorax obliquity (deg)
Peak thorax flexion (deg)
T2S (s)
Turn (s)
Walk (s)
S2S (s)
Total (s)
Peak angular velocity thorax (deg/s)
Distance chair to start turn (m)
Range thorax obliquity (deg)
Mean angular velocity pelvis (deg/s)
Steps Number
Peak forward velocity pelvis (m/s)
Walk
S2S
Time
T2S
Turn
3.8
50.5
1.1
1.2
64.6
2.2
1.1
2.8
0.6
8.3
257.3
1.6
8.0
163.4
2.0
1.9
35.0
11.9
0.2
14.2
14.3
8.5
4.2
17.4
2.9
29.1
83.1
0.5
48.4
41.4
6.0
0.5

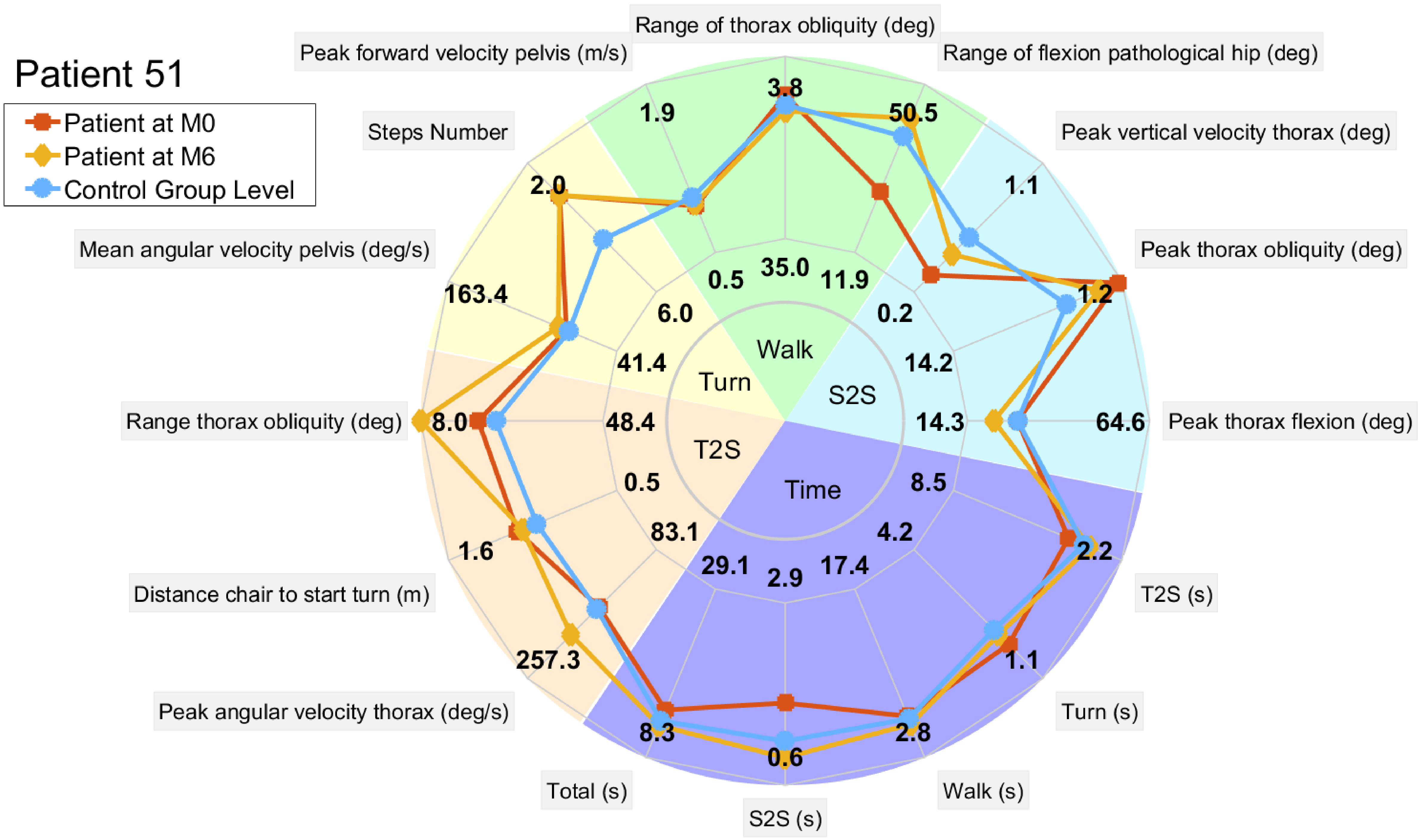

Patient 51
Patient at M0
Patient at M6
Control Group Level
Range of thorax obliquity (deg)
Peak forward velocity pelvis (m/s)
Range of flexion pathological hip (deg)
Steps Number
Peak vertical velocity thorax (deg)
Mean angular velocity pelvis (deg/s)
Peak thorax obliquity (deg)
Range thorax obliquity (deg)
Peak thorax flexion (deg)
Distance chair to start turn (m)
T2S (s)
Peak angular velocity thorax (deg/s)
Turn (s)
Total (s)
S2S (s)
Walk (s)
Walk
Turn
S2S
T2S
Time
3.8
1.9
50.5
2.0
1.1
163.4
1.2
8.0
64.6
1.6
2.2
257.3
1.1
8.3
0.6
2.8
0.5
35.0
11.9
6.0
0.2
41.4
14.2
48.4
14.3
0.5
8.5
83.1
4.2
29.1
2.9
17.4

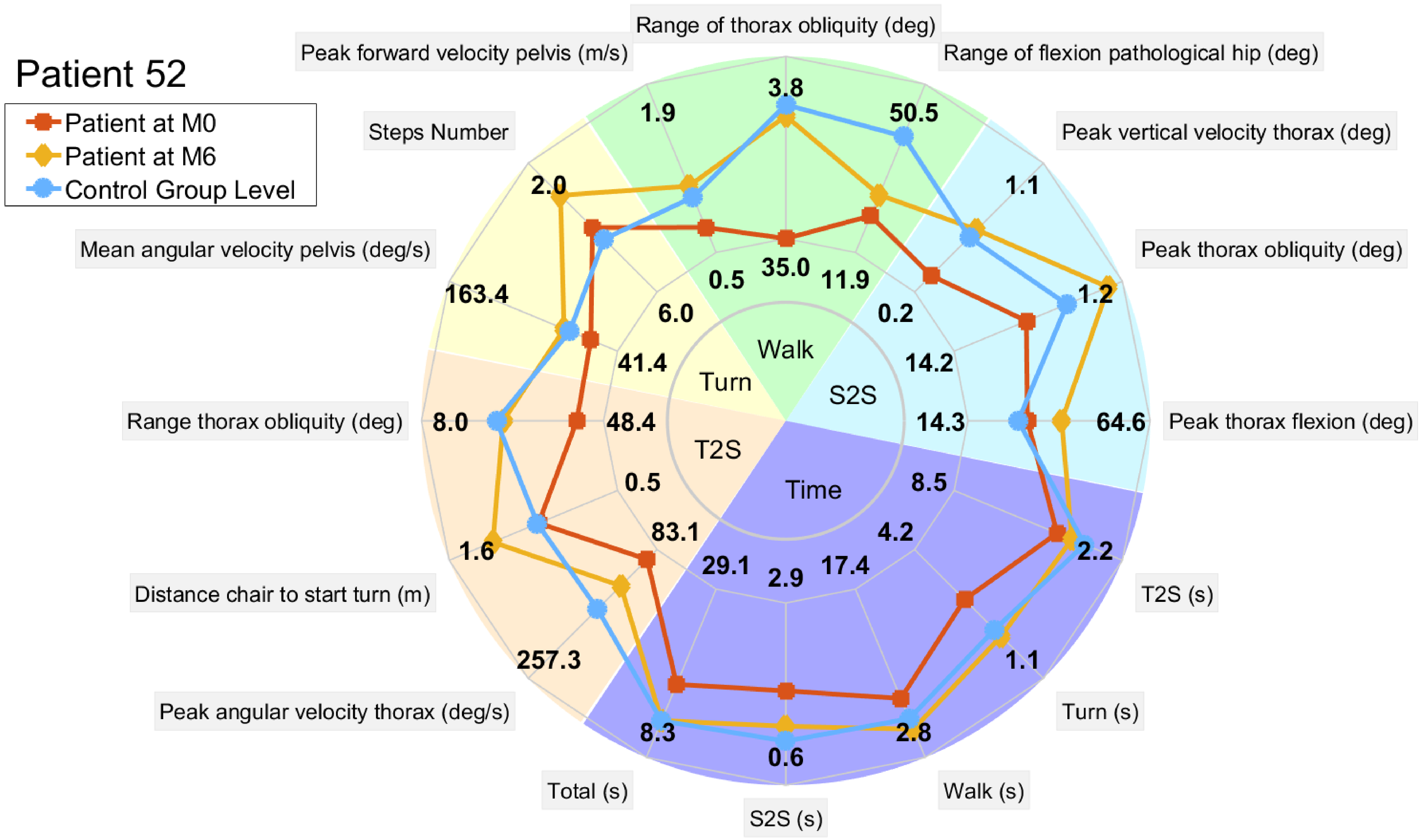
Patient 52
Patient at M0
Patient at M6
Control Group Level
Range of thorax obliquity (deg)
Peak forward velocity pelvis (m/s)
Range of flexion pathological hip (deg)
Steps Number
Peak vertical velocity thorax (deg)
Mean angular velocity pelvis (deg/s)
Peak thorax obliquity (deg)
Range thorax obliquity (deg)
Peak thorax flexion (deg)
Distance chair to start turn (m)
T2S (s)
Peak angular velocity thorax (deg/s)
Turn (s)
Total (s)
S2S (s)
Walk (s)
Walk
Turn
S2S
T2S
Time
3.8
1.9
50.5
2.0
1.1
0.5
35.0
11.9
163.4
6.0
0.2
1.2
41.4
14.2
8.0
48.4
14.3
64.6
0.5
8.5
83.1
4.2
1.6
29.1
2.9
17.4
2.2
257.3
1.1
8.3
0.6
2.8

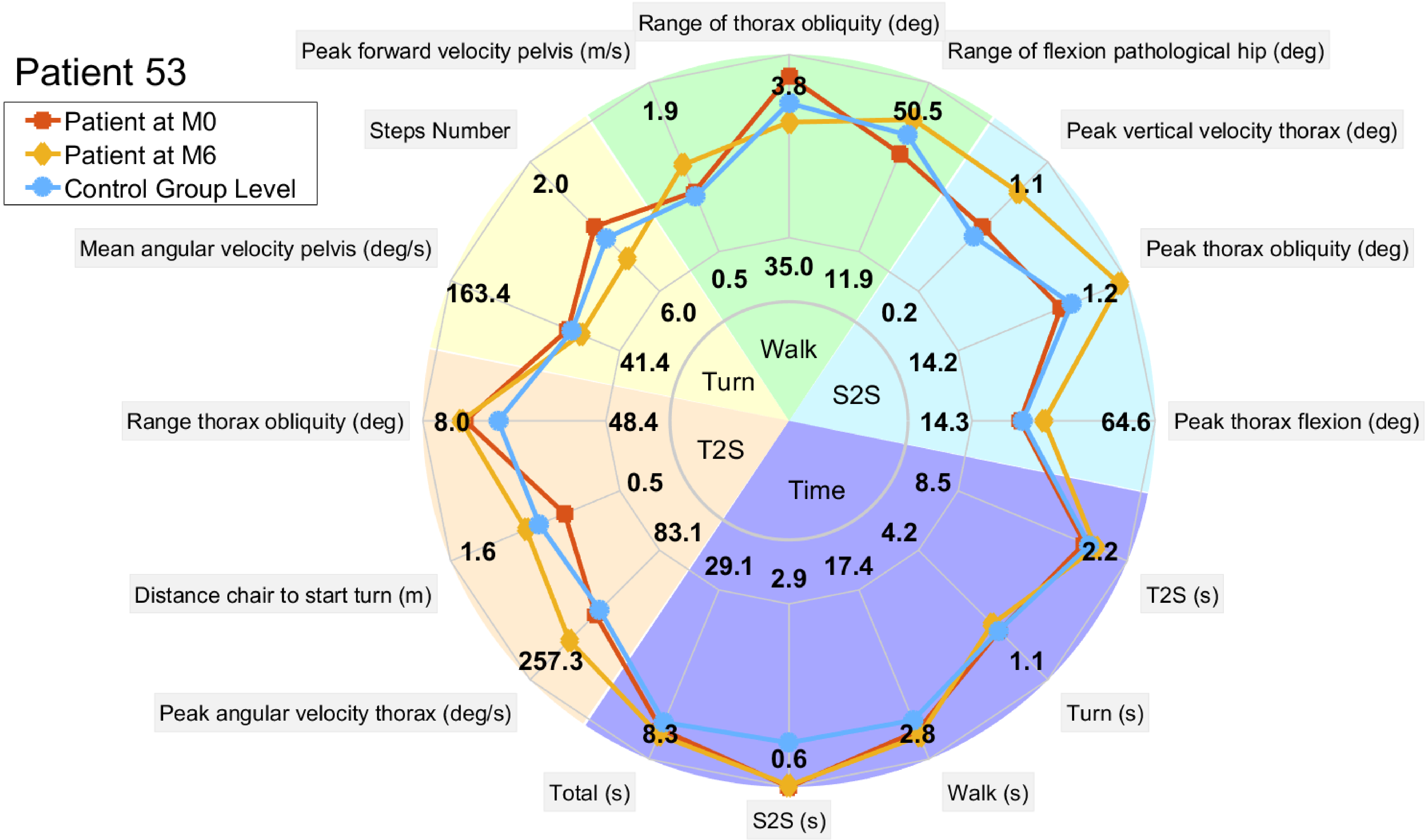

Patient 53
Patient at M0
Patient at M6
Control Group Level
Range of thorax obliquity (deg)
Range of flexion pathological hip (deg)
Peak vertical velocity thorax (deg)
Peak thorax obliquity (deg)
Peak thorax flexion (deg)
T2S (s)
Turn (s)
Walk (s)
S2S (s)
Total (s)
Peak angular velocity thorax (deg/s)
Distance chair to start turn (m)
Range thorax obliquity (deg)
Mean angular velocity pelvis (deg/s)
Steps Number
Peak forward velocity pelvis (m/s)
Walk
S2S
Time
T2S
Turn
3.8
50.5
1.1
1.2
64.6
2.2
1.1
2.8
0.6
8.3
257.3
1.6
8.0
163.4
2.0
1.9
35.0
11.9
0.2
14.2
14.3
8.5
4.2
17.4
2.9
29.1
83.1
0.5
48.4
41.4
6.0
0.5

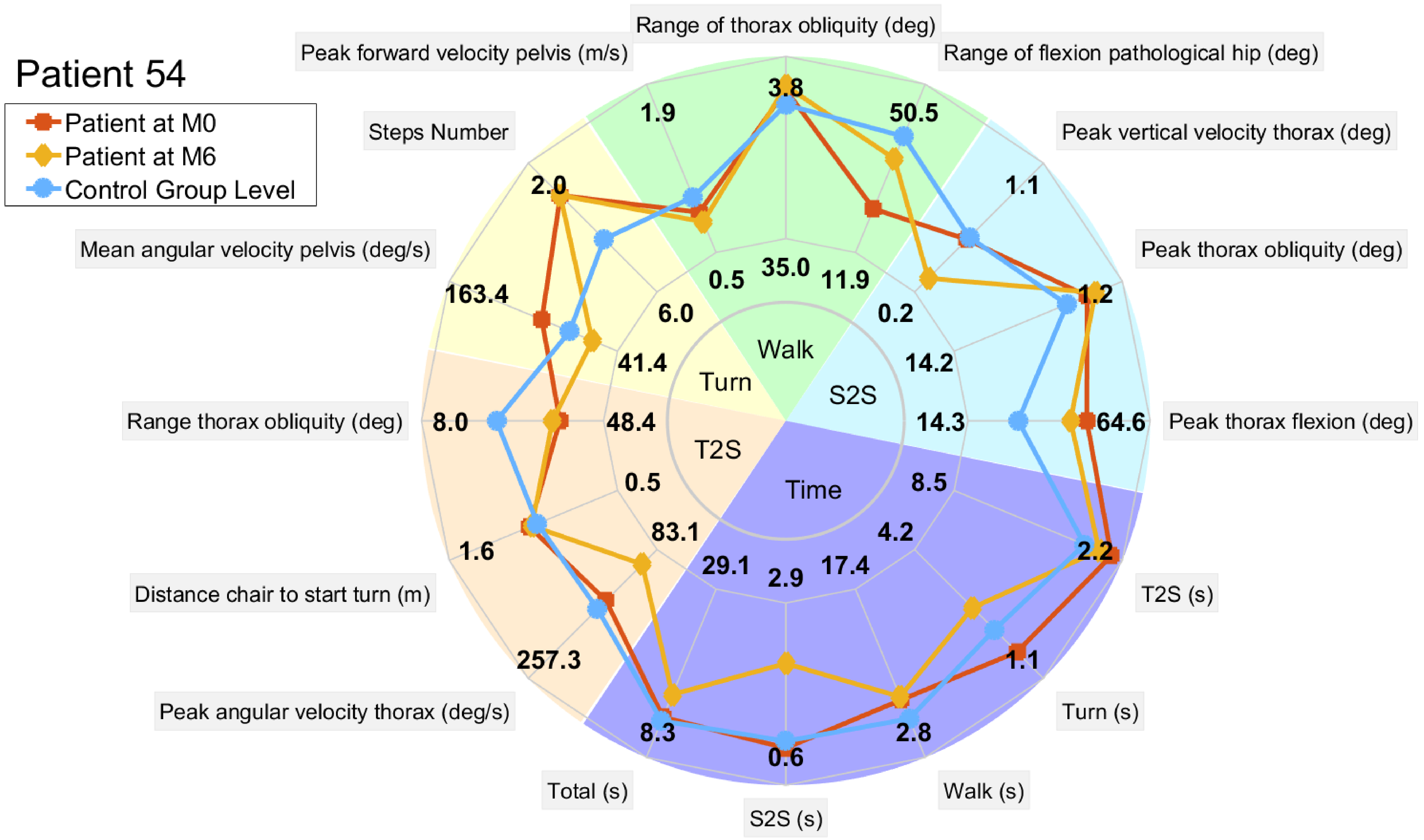

Patient 54
Patient at M0
Patient at M6
Control Group Level
Range of thorax obliquity (deg)
Peak forward velocity pelvis (m/s)
Range of flexion pathological hip (deg)
Steps Number
Peak vertical velocity thorax (deg)
Mean angular velocity pelvis (deg/s)
Peak thorax obliquity (deg)
Range thorax obliquity (deg)
Peak thorax flexion (deg)
Distance chair to start turn (m)
T2S (s)
Peak angular velocity thorax (deg/s)
Turn (s)
Total (s)
Walk (s)
S2S (s)
Walk
Turn
S2S
T2S
Time
3.8
1.9
50.5
2.0
1.1
163.4
1.2
8.0
64.6
1.6
2.2
257.3
1.1
8.3
2.8
0.6
35.0
0.5
11.9
6.0
0.2
41.4
14.2
48.4
14.3
0.5
8.5
83.1
4.2
29.1
2.9
17.4

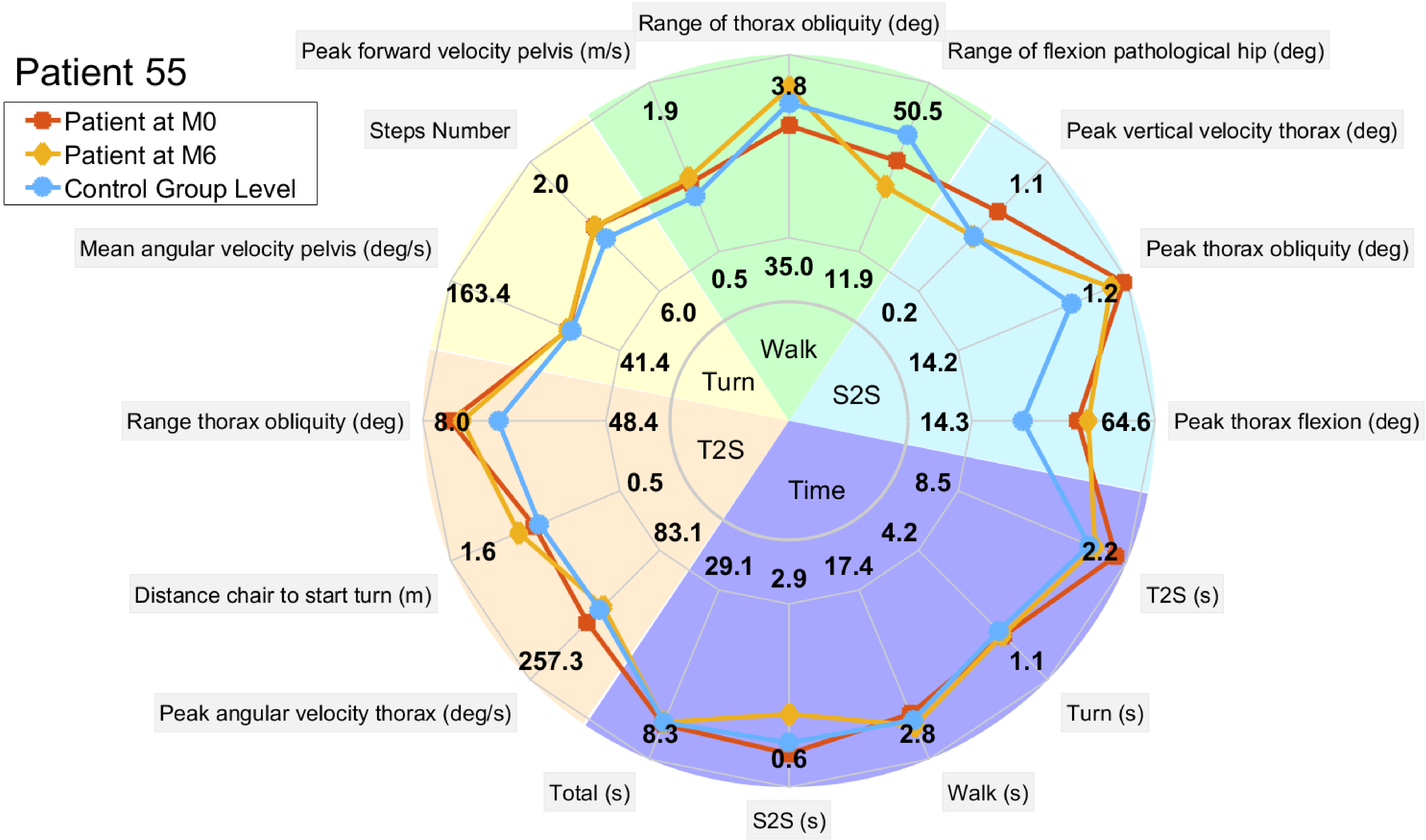

Patient 55
Patient at M0
Patient at M6
Control Group Level
Range of thorax obliquity (deg)
Peak forward velocity pelvis (m/s)
Range of flexion pathological hip (deg)
Steps Number
Peak vertical velocity thorax (deg)
Mean angular velocity pelvis (deg/s)
Peak thorax obliquity (deg)
Range thorax obliquity (deg)
Peak thorax flexion (deg)
Distance chair to start turn (m)
T2S (s)
Peak angular velocity thorax (deg/s)
Turn (s)
Total (s)
Walk (s)
S2S (s)
Walk
Turn
S2S
T2S
Time
3.8
1.9
50.5
2.0
1.1
163.4
1.2
8.0
64.6
1.6
2.2
257.3
1.1
8.3
2.8
0.6
35.0
0.5
11.9
6.0
0.2
41.4
14.2
48.4
14.3
0.5
8.5
83.1
4.2
29.1
2.9
17.4

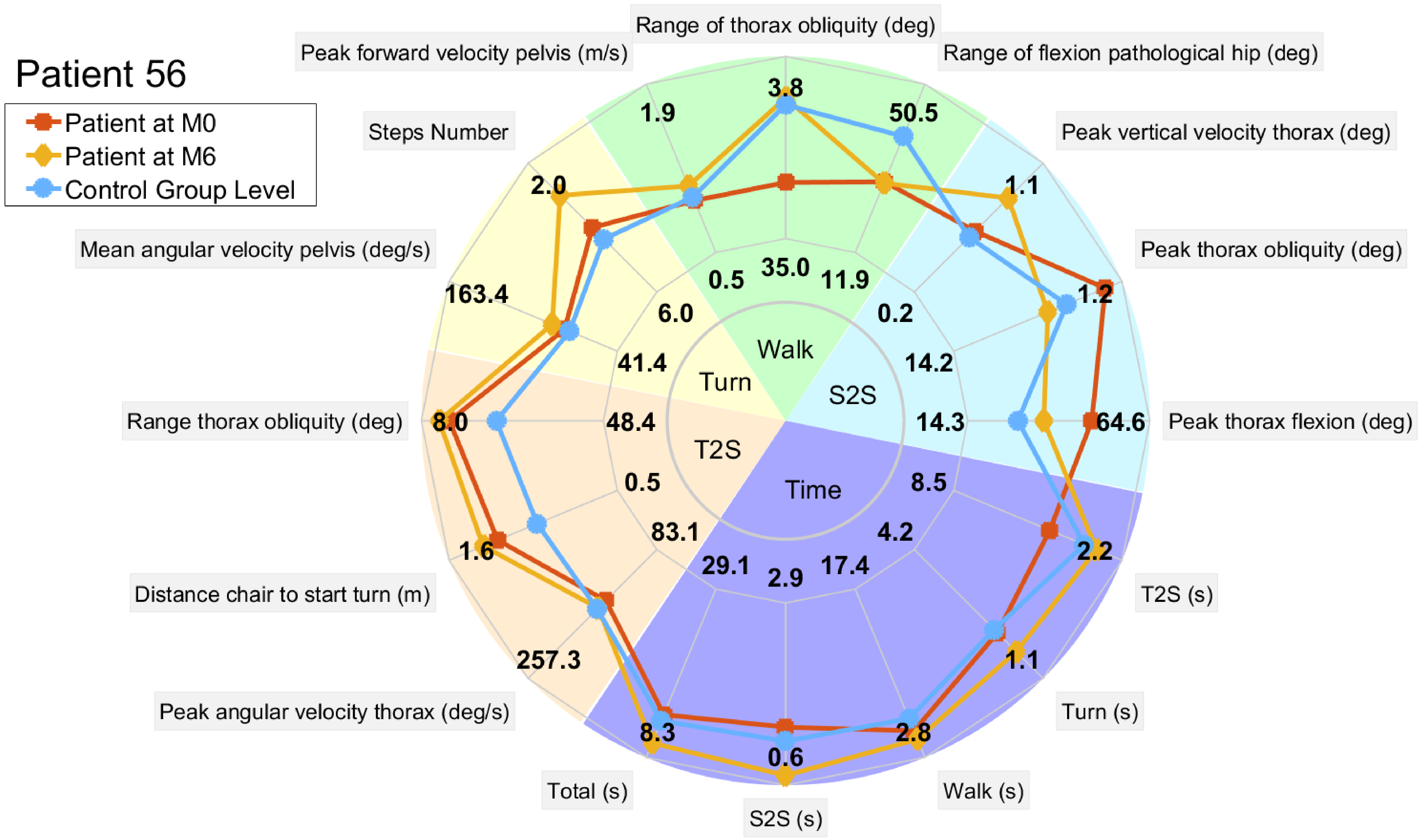

Patient 56
Patient at M0
Patient at M6
Control Group Level
Range of thorax obliquity (deg)
Peak forward velocity pelvis (m/s)
Range of flexion pathological hip (deg)
Steps Number
Peak vertical velocity thorax (deg)
Mean angular velocity pelvis (deg/s)
Peak thorax obliquity (deg)
Range thorax obliquity (deg)
Peak thorax flexion (deg)
Distance chair to start turn (m)
T2S (s)
Peak angular velocity thorax (deg/s)
Turn (s)
Total (s)
S2S (s)
Walk (s)
Walk
Turn
S2S
T2S
Time
3.8
1.9
50.5
2.0
1.1
163.4
1.2
8.0
64.6
1.6
2.2
257.3
1.1
8.3
0.6
2.8
35.0
0.5
11.9
6.0
0.2
41.4
14.2
48.4
14.3
0.5
8.5
83.1
4.2
29.1
2.9
17.4

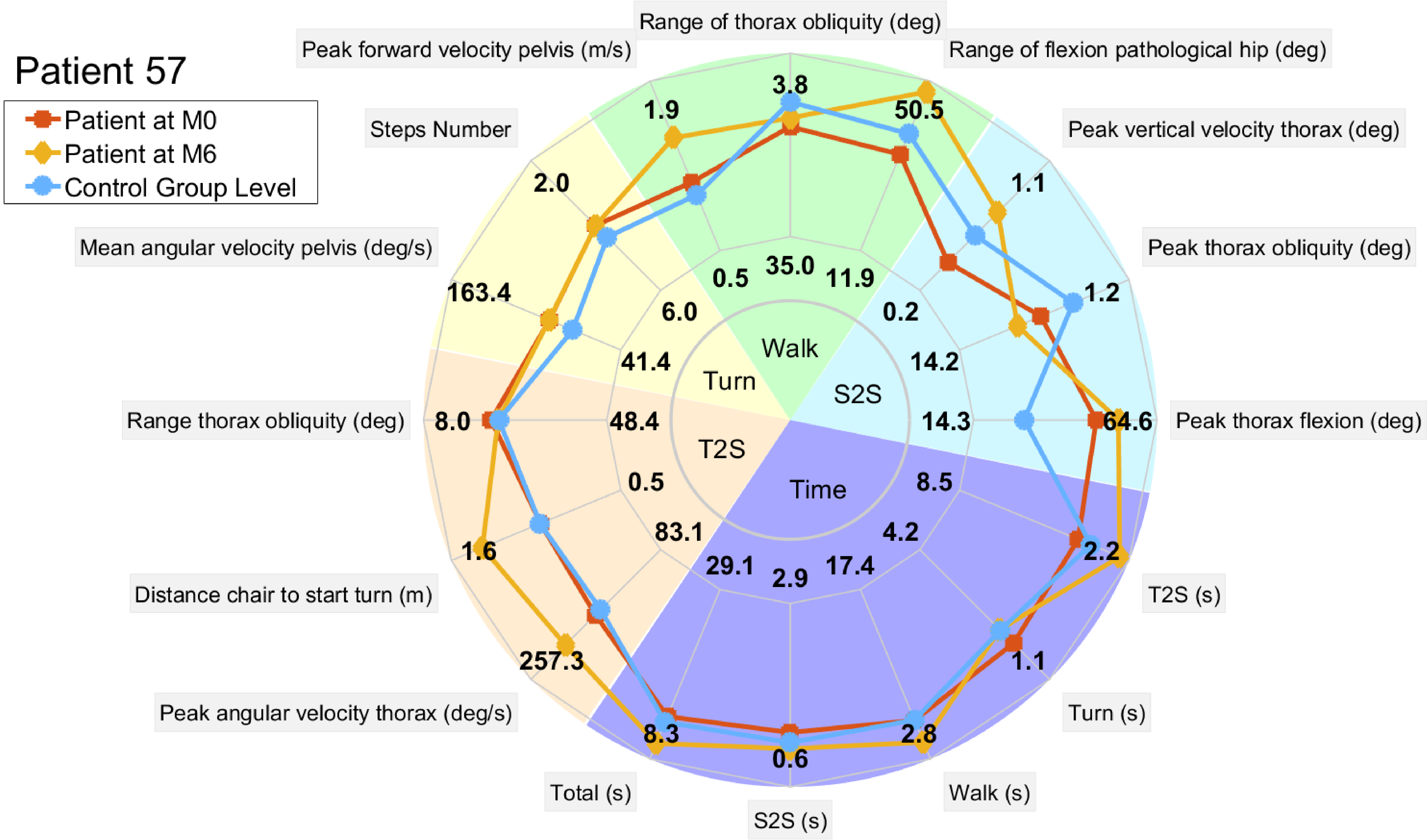
Patient 57
Patient at M0
Patient at M6
Control Group Level
Range of thorax obliquity (deg)
Peak forward velocity pelvis (m/s)
Range of flexion pathological hip (deg)
Steps Number
Peak vertical velocity thorax (deg)
Mean angular velocity pelvis (deg/s)
Peak thorax obliquity (deg)
Range thorax obliquity (deg)
Peak thorax flexion (deg)
Distance chair to start turn (m)
T2S (s)
Peak angular velocity thorax (deg/s)
Turn (s)
Total (s)
Walk (s)
S2S (s)
Walk
Turn
S2S
T2S
Time
3.8
35.0
1.9
0.5
50.5
11.9
2.0
6.0
1.1
0.2
163.4
41.4
1.2
14.2
8.0
48.4
64.6
14.3
0.5
8.5
1.6
83.1
2.2
4.2
29.1
2.9
17.4
257.3
1.1
8.3
0.6
2.8

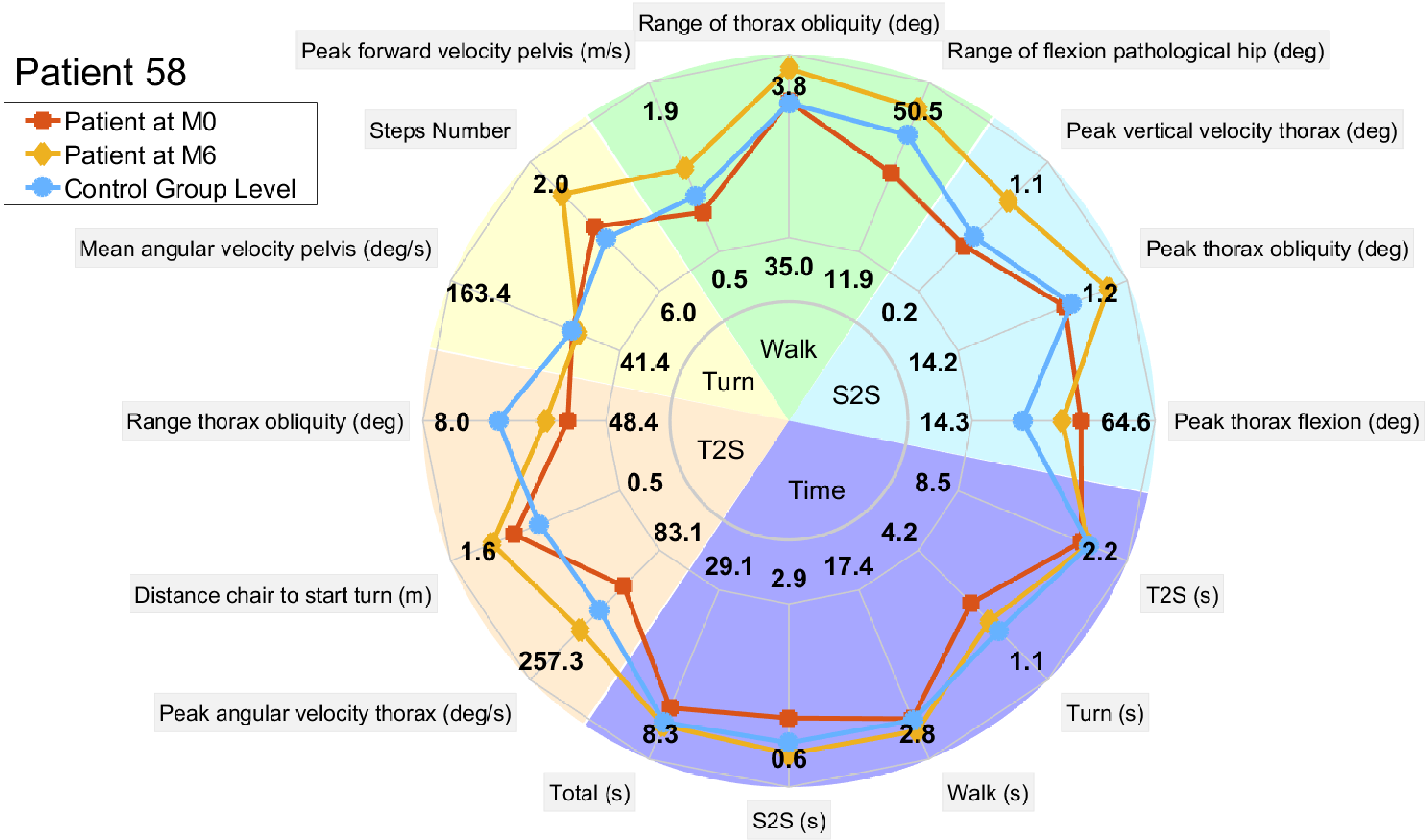
Patient 58
Patient at M0
Patient at M6
Control Group Level
Range of thorax obliquity (deg)
Peak forward velocity pelvis (m/s)
Range of flexion pathological hip (deg)
Steps Number
Peak vertical velocity thorax (deg)
Mean angular velocity pelvis (deg/s)
Peak thorax obliquity (deg)
Range thorax obliquity (deg)
Peak thorax flexion (deg)
Distance chair to start turn (m)
T2S (s)
Peak angular velocity thorax (deg/s)
Turn (s)
Total (s)
Walk (s)
S2S (s)
Walk
Turn
S2S
T2S
Time
3.8
1.9
50.5
2.0
1.1
163.4
1.2
8.0
64.6
1.6
2.2
257.3
1.1
8.3
0.6
2.8
0.5
35.0
11.9
6.0
0.2
41.4
14.2
48.4
14.3
0.5
8.5
83.1
4.2
29.1
2.9
17.4

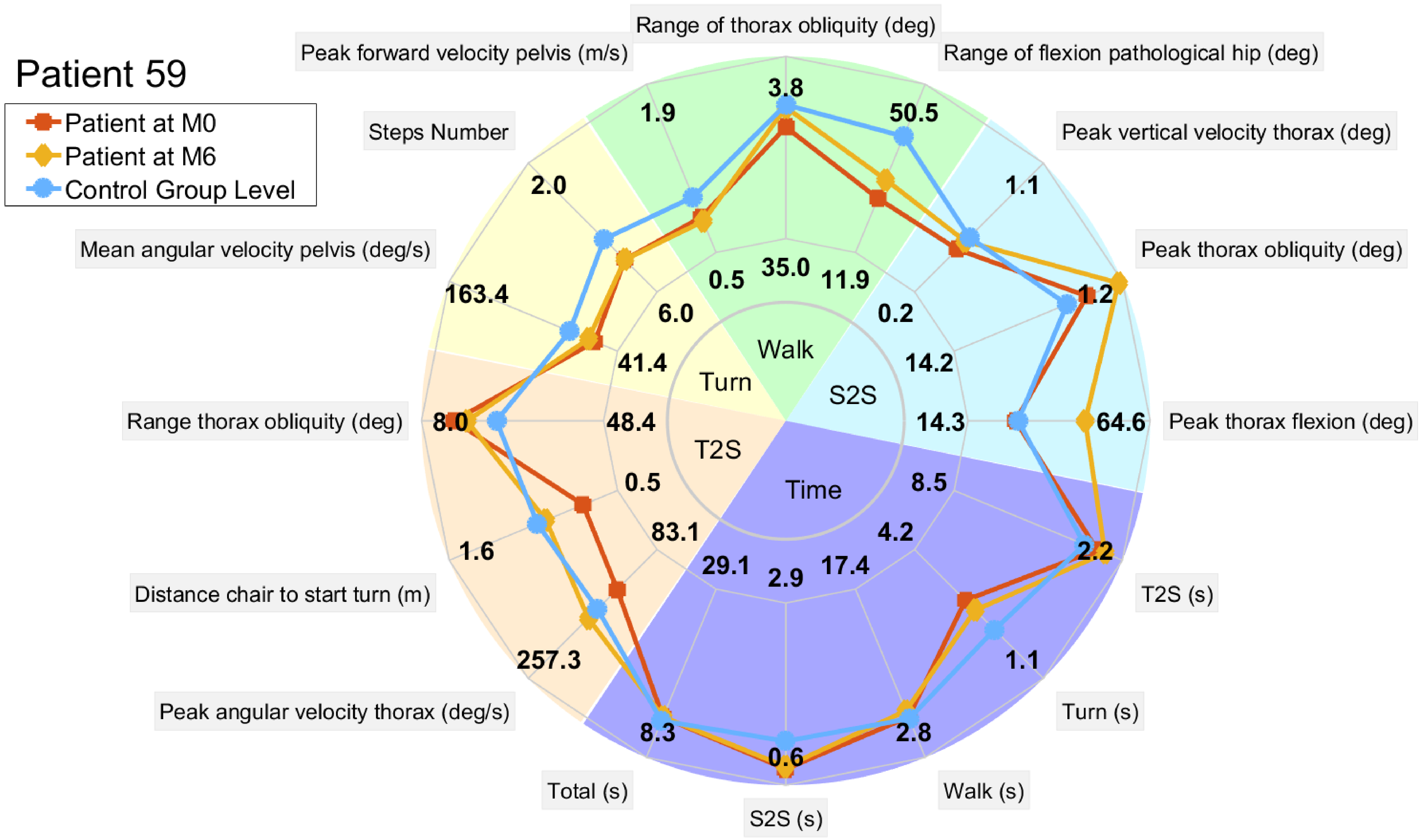
Patient 59
Patient at M0
Patient at M6
Control Group Level
Range of thorax obliquity (deg)
Range of flexion pathological hip (deg)
Peak vertical velocity thorax (deg)
Peak thorax obliquity (deg)
Peak thorax flexion (deg)
T2S (s)
Turn (s)
Walk (s)
S2S (s)
Total (s)
Peak angular velocity thorax (deg/s)
Distance chair to start turn (m)
Range thorax obliquity (deg)
Mean angular velocity pelvis (deg/s)
Steps Number
Peak forward velocity pelvis (m/s)
Walk
Turn
S2S
T2S
Time
3.8
50.5
1.1
1.2
64.6
2.2
1.1
2.8
0.6
8.3
257.3
1.6
8.0
163.4
2.0
1.9
35.0
11.9
0.2
14.2
14.3
8.5
4.2
17.4
2.9
29.1
83.1
0.5
48.4
41.4
6.0
0.5

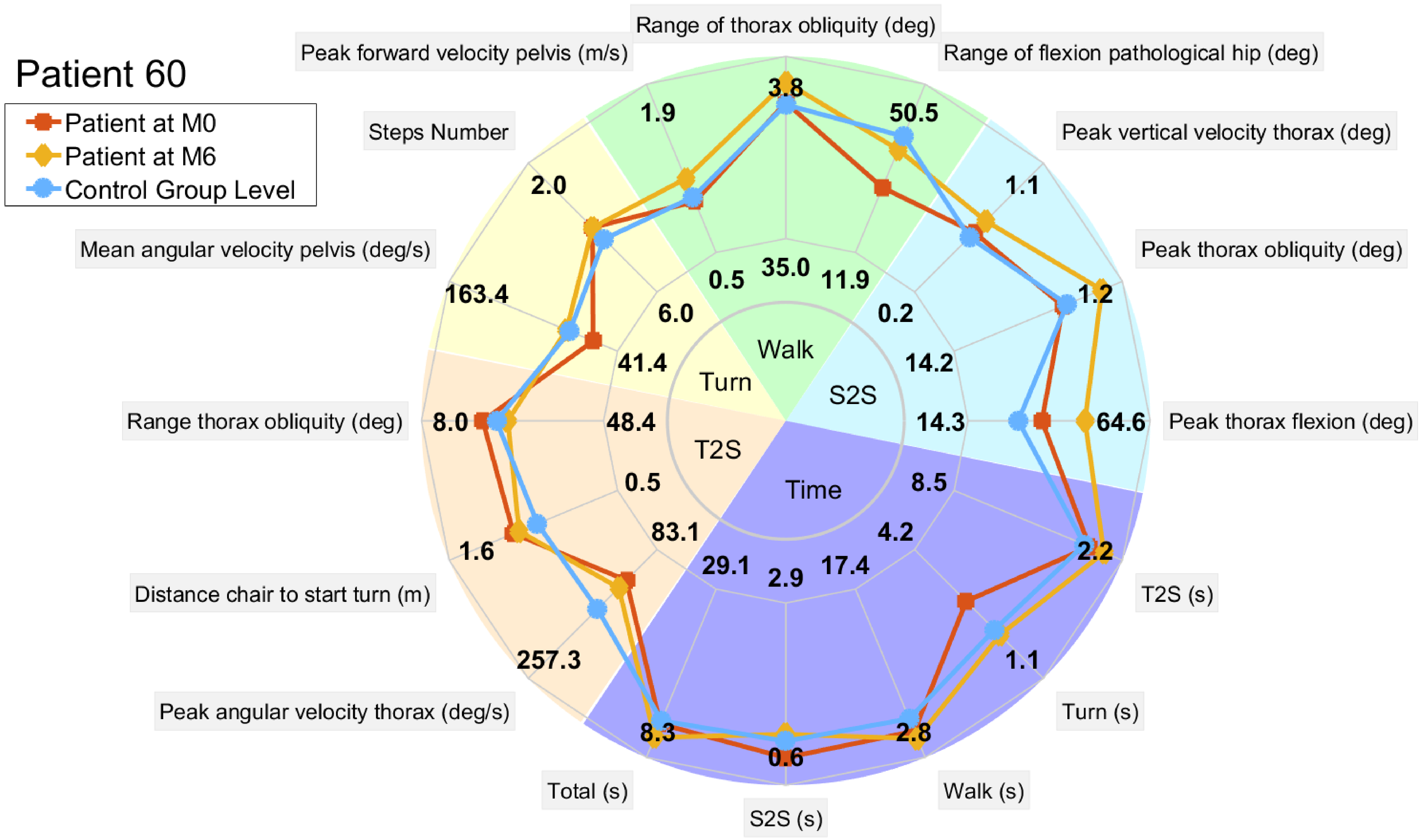
Patient 60
Patient at M0
Patient at M6
Control Group Level
Range of thorax obliquity (deg)
Range of flexion pathological hip (deg)
Peak vertical velocity thorax (deg)
Peak thorax obliquity (deg)
Peak thorax flexion (deg)
T2S (s)
Turn (s)
Walk (s)
S2S (s)
Total (s)
Peak angular velocity thorax (deg/s)
Distance chair to start turn (m)
Range thorax obliquity (deg)
Mean angular velocity pelvis (deg/s)
Steps Number
Peak forward velocity pelvis (m/s)
Walk
Turn
S2S
T2S
Time
3.8
50.5
1.1
1.2
64.6
2.2
1.1
2.8
0.6
8.3
257.3
1.6
8.0
163.4
2.0
1.9
35.0
11.9
0.2
14.2
14.3
8.5
4.2
17.4
2.9
29.1
83.1
0.5
48.4
41.4
6.0
0.5

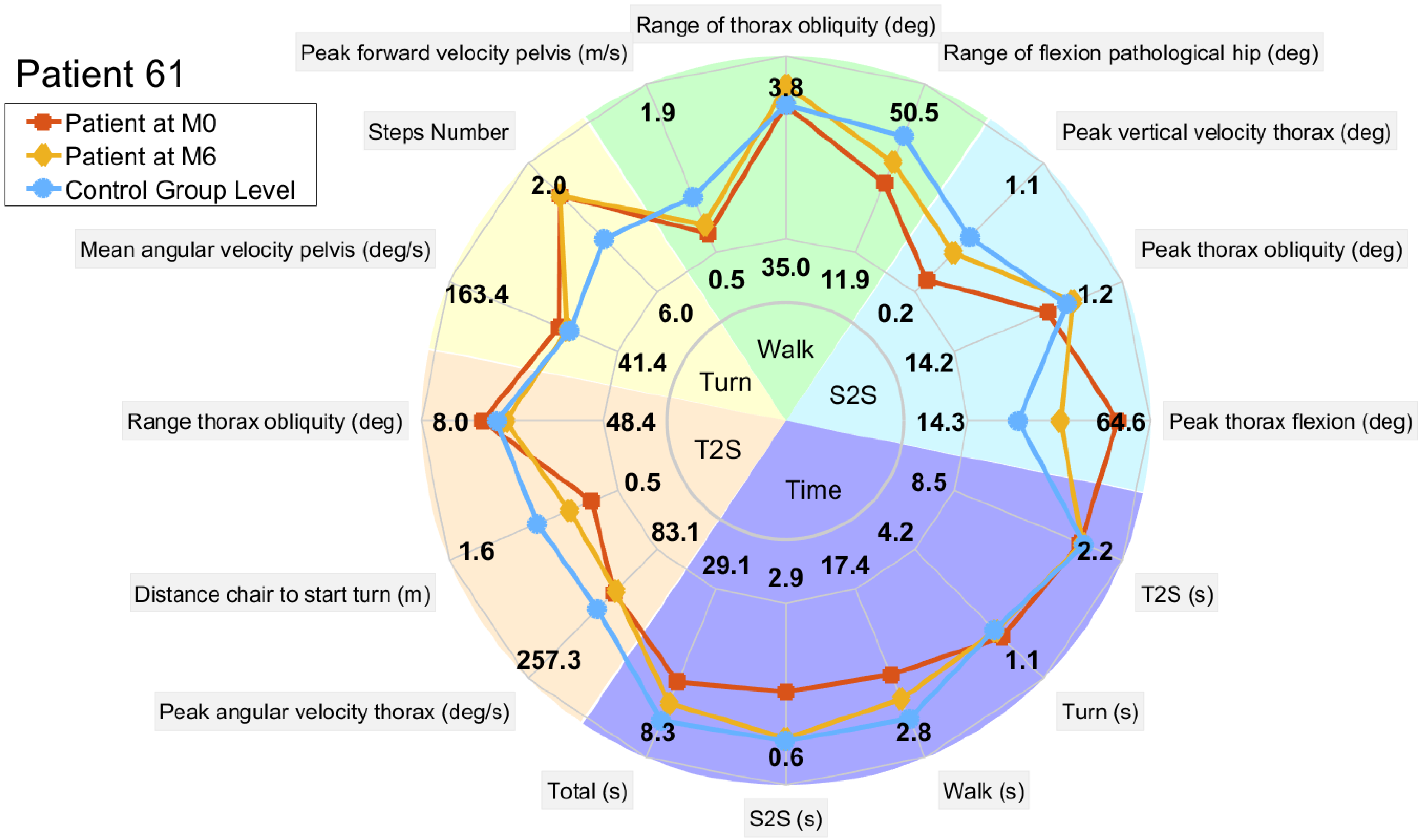
Patient 61
Patient at M0
Patient at M6
Control Group Level
Range of thorax obliquity (deg)
Range of flexion pathological hip (deg)
Peak vertical velocity thorax (deg)
Peak thorax obliquity (deg)
Peak thorax flexion (deg)
T2S (s)
Turn (s)
Walk (s)
S2S (s)
Total (s)
Peak angular velocity thorax (deg/s)
Distance chair to start turn (m)
Range thorax obliquity (deg)
Mean angular velocity pelvis (deg/s)
Steps Number
Peak forward velocity pelvis (m/s)
Walk
S2S
Time
T2S
Turn
3.8
35.0
50.5
11.9
1.1
0.2
1.2
14.2
64.6
14.3
2.2
8.5
1.1
4.2
2.8
17.4
0.6
2.9
8.3
29.1
257.3
83.1
1.6
0.5
8.0
48.4
163.4
41.4
2.0
6.0
1.9
0.5

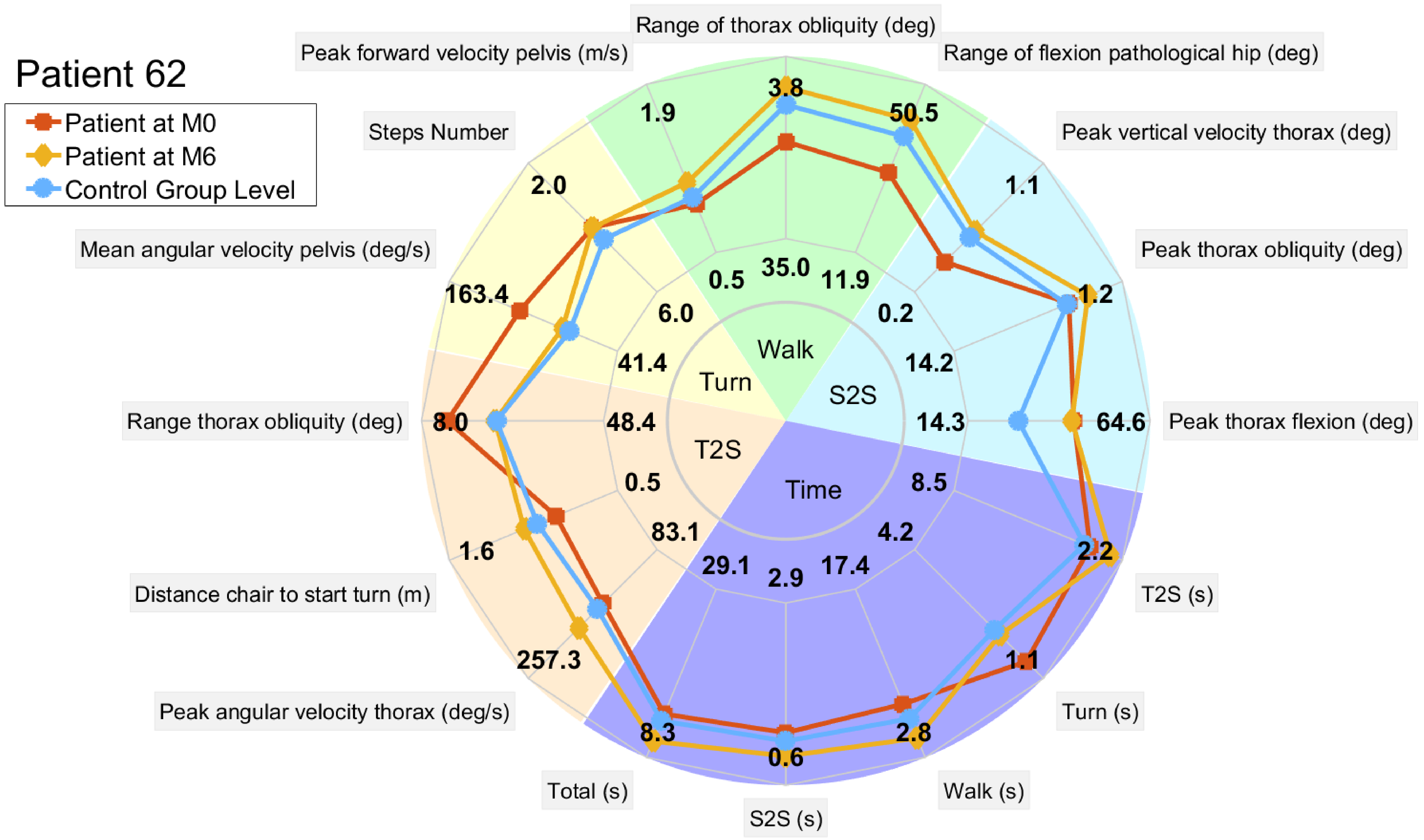

Patient 62
Patient at M0
Patient at M6
Control Group Level
Range of thorax obliquity (deg)
Range of flexion pathological hip (deg)
Peak vertical velocity thorax (deg)
Peak thorax obliquity (deg)
Peak thorax flexion (deg)
T2S (s)
Turn (s)
Walk (s)
S2S (s)
Total (s)
Peak angular velocity thorax (deg/s)
Distance chair to start turn (m)
Range thorax obliquity (deg)
Mean angular velocity pelvis (deg/s)
Steps Number
Peak forward velocity pelvis (m/s)
Walk
S2S
Time
T2S
Turn
3.8
50.5
1.1
1.2
64.6
2.2
1.1
2.8
0.6
8.3
257.3
1.6
8.0
163.4
2.0
1.9
35.0
11.9
0.2
14.2
14.3
8.5
4.2
17.4
2.9
29.1
83.1
0.5
48.4
41.4
6.0
0.5

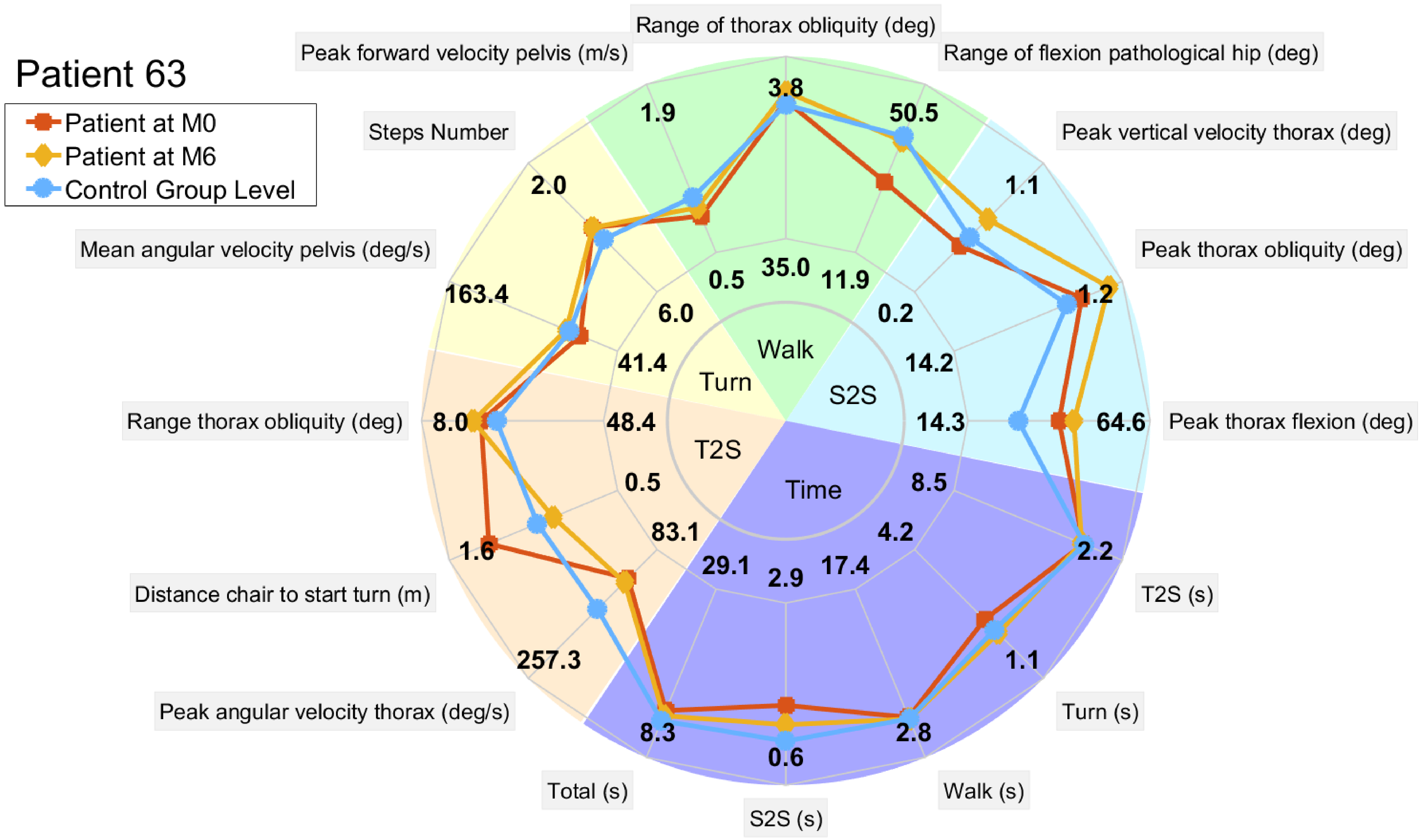

Patient 63
Patient at M0
Patient at M6
Control Group Level
Range of thorax obliquity (deg)
Range of flexion pathological hip (deg)
Peak vertical velocity thorax (deg)
Peak thorax obliquity (deg)
Peak thorax flexion (deg)
T2S (s)
Turn (s)
Walk (s)
S2S (s)
Total (s)
Peak angular velocity thorax (deg/s)
Distance chair to start turn (m)
Range thorax obliquity (deg)
Mean angular velocity pelvis (deg/s)
Steps Number
Peak forward velocity pelvis (m/s)
Walk
S2S
Time
T2S
Turn
3.8
50.5
1.1
1.2
64.6
2.2
1.1
2.8
0.6
8.3
257.3
1.6
8.0
163.4
2.0
1.9
35.0
11.9
0.2
14.2
14.3
8.5
4.2
17.4
2.9
29.1
83.1
0.5
48.4
41.4
6.0
0.5

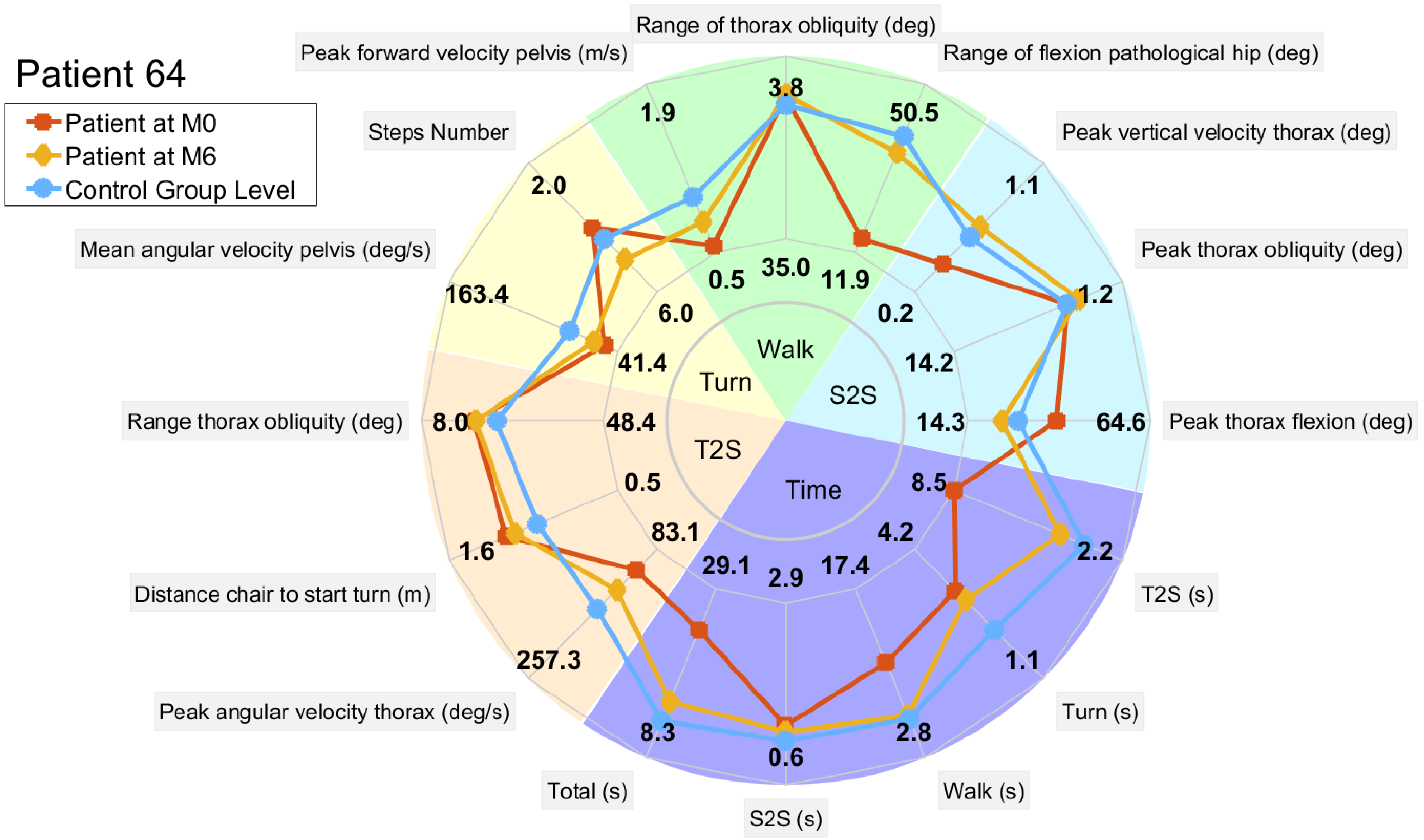
Patient 64
Patient at M0
Patient at M6
Control Group Level
Range of thorax obliquity (deg)
Range of flexion pathological hip (deg)
Peak vertical velocity thorax (deg)
Peak thorax obliquity (deg)
Peak thorax flexion (deg)
T2S (s)
Turn (s)
Walk (s)
S2S (s)
Total (s)
Peak angular velocity thorax (deg/s)
Distance chair to start turn (m)
Range thorax obliquity (deg)
Mean angular velocity pelvis (deg/s)
Steps Number
Peak forward velocity pelvis (m/s)
Walk
Turn
S2S
T2S
Time
3.8
35.0
50.5
11.9
1.1
0.2
1.2
14.2
64.6
14.3
8.5
4.2
2.2
17.4
1.1
2.8
2.9
0.6
29.1
8.3
83.1
257.3
0.5
1.6
48.4
8.0
41.4
163.4
6.0
2.0
0.5
1.9

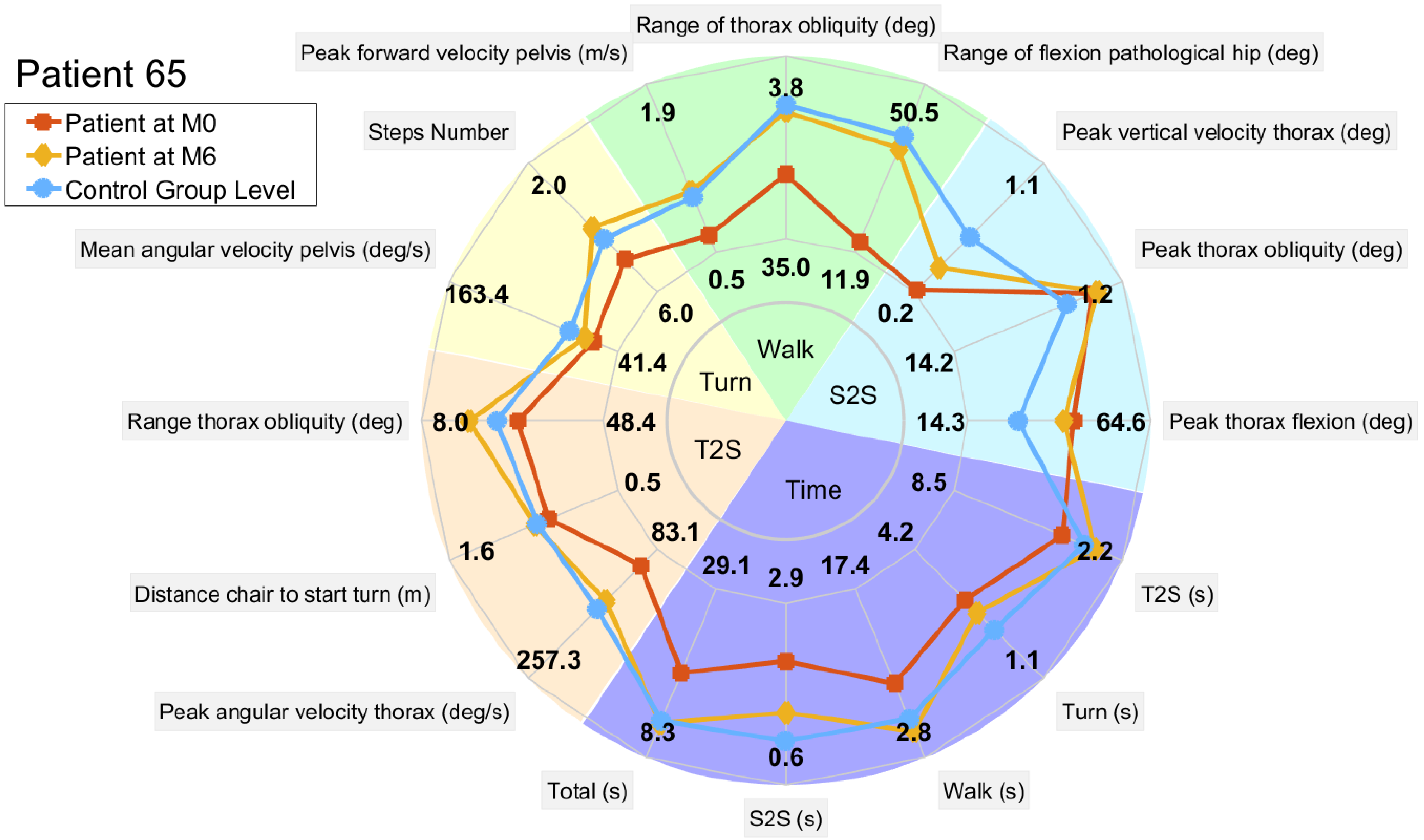

Patient 65
Patient at M0
Patient at M6
Control Group Level
Range of thorax obliquity (deg)
Peak forward velocity pelvis (m/s)
Range of flexion pathological hip (deg)
Steps Number
Peak vertical velocity thorax (deg)
Mean angular velocity pelvis (deg/s)
Peak thorax obliquity (deg)
Range thorax obliquity (deg)
Peak thorax flexion (deg)
Distance chair to start turn (m)
T2S (s)
Peak angular velocity thorax (deg/s)
Turn (s)
Total (s)
Walk (s)
S2S (s)
Walk
Turn
S2S
T2S
Time
3.8
1.9
50.5
2.0
1.1
0.5
35.0
11.9
0.2
163.4
6.0
1.2
41.4
14.2
8.0
48.4
14.3
64.6
0.5
8.5
1.6
83.1
4.2
2.2
29.1
2.9
17.4
257.3
1.1
8.3
0.6
2.8

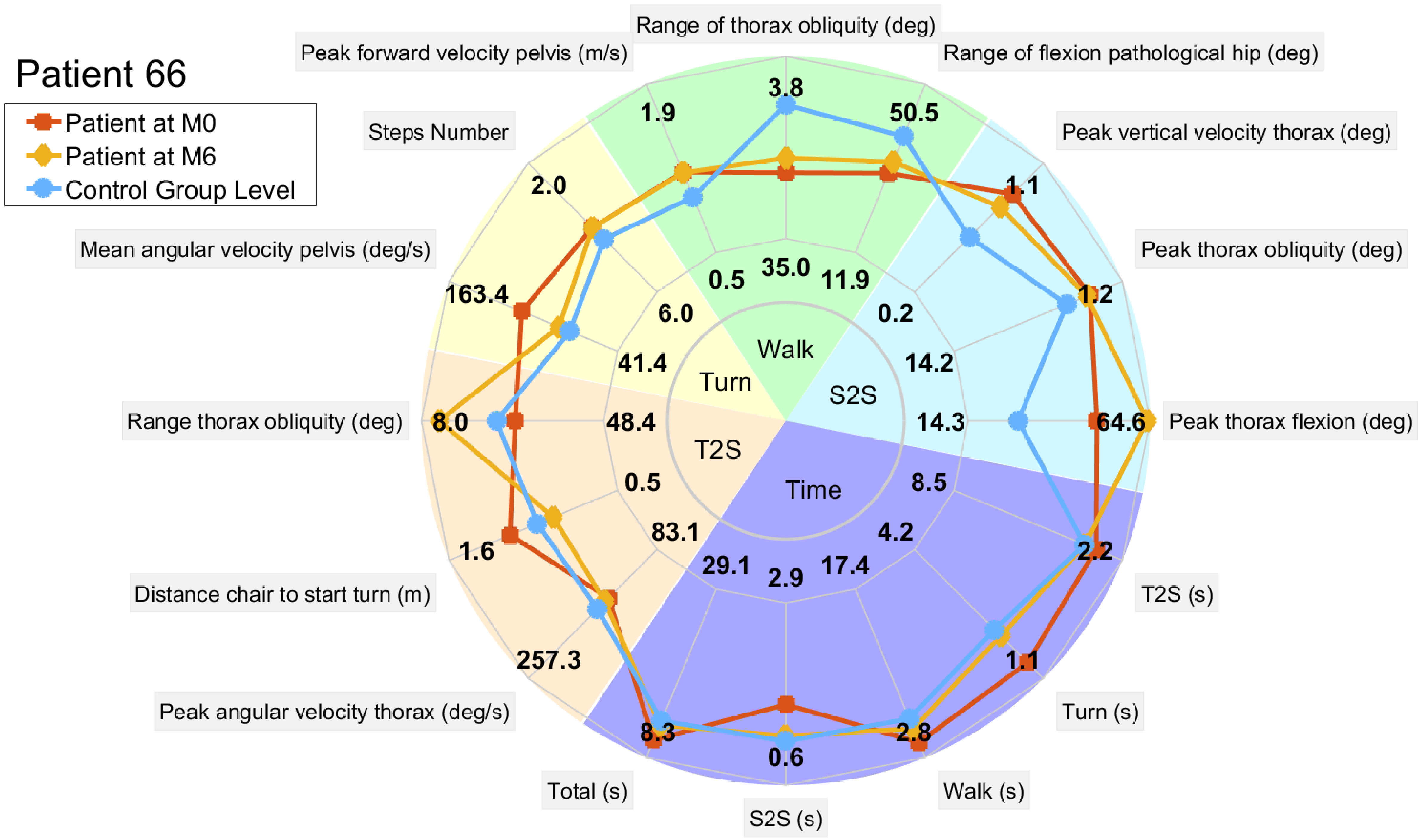

Patient 66
Patient at M0
Patient at M6
Control Group Level
Range of thorax obliquity (deg)
Range of flexion pathological hip (deg)
Peak vertical velocity thorax (deg)
Peak thorax obliquity (deg)
Peak thorax flexion (deg)
T2S (s)
Turn (s)
Walk (s)
S2S (s)
Total (s)
Peak angular velocity thorax (deg/s)
Distance chair to start turn (m)
Range thorax obliquity (deg)
Mean angular velocity pelvis (deg/s)
Steps Number
Peak forward velocity pelvis (m/s)
Walk
S2S
Time
T2S
Turn
3.8
50.5
1.1
1.2
64.6
2.2
1.1
2.8
0.6
8.3
257.3
1.6
8.0
163.4
2.0
1.9
35.0
11.9
0.2
14.2
14.3
8.5
4.2
17.4
2.9
29.1
83.1
0.5
48.4
41.4
6.0
0.5

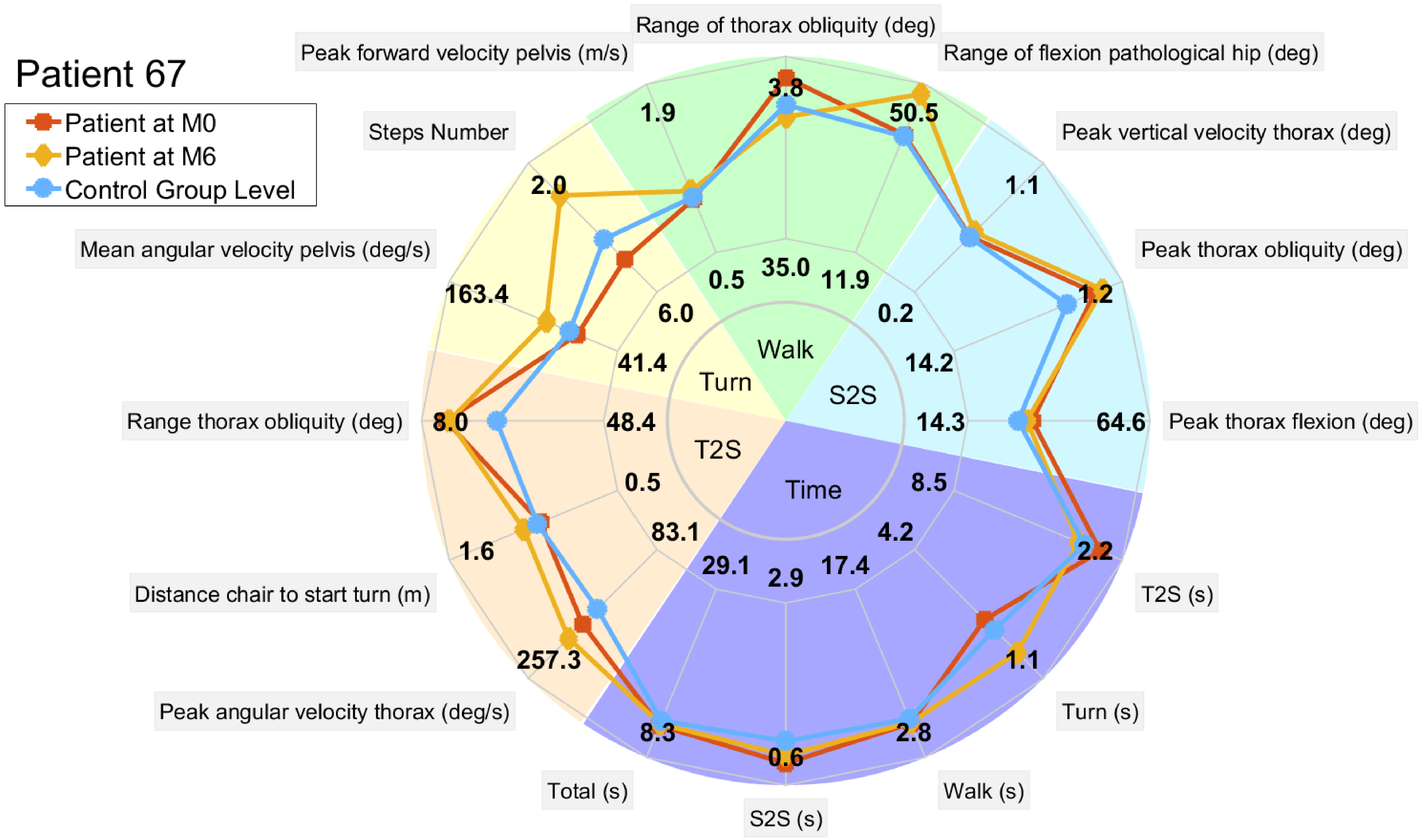

Patient 67
Patient at M0
Patient at M6
Control Group Level
Range of thorax obliquity (deg)
Range of flexion pathological hip (deg)
Peak vertical velocity thorax (deg)
Peak thorax obliquity (deg)
Peak thorax flexion (deg)
T2S (s)
Turn (s)
Walk (s)
S2S (s)
Total (s)
Peak angular velocity thorax (deg/s)
Distance chair to start turn (m)
Range thorax obliquity (deg)
Mean angular velocity pelvis (deg/s)
Steps Number
Peak forward velocity pelvis (m/s)
Walk
S2S
Time
T2S
Turn
3.8
50.5
1.1
1.2
64.6
2.2
1.1
2.8
0.6
8.3
257.3
1.6
8.0
163.4
2.0
1.9
35.0
11.9
0.2
14.2
14.3
8.5
4.2
17.4
2.9
29.1
83.1
0.5
48.4
41.4
6.0
0.5

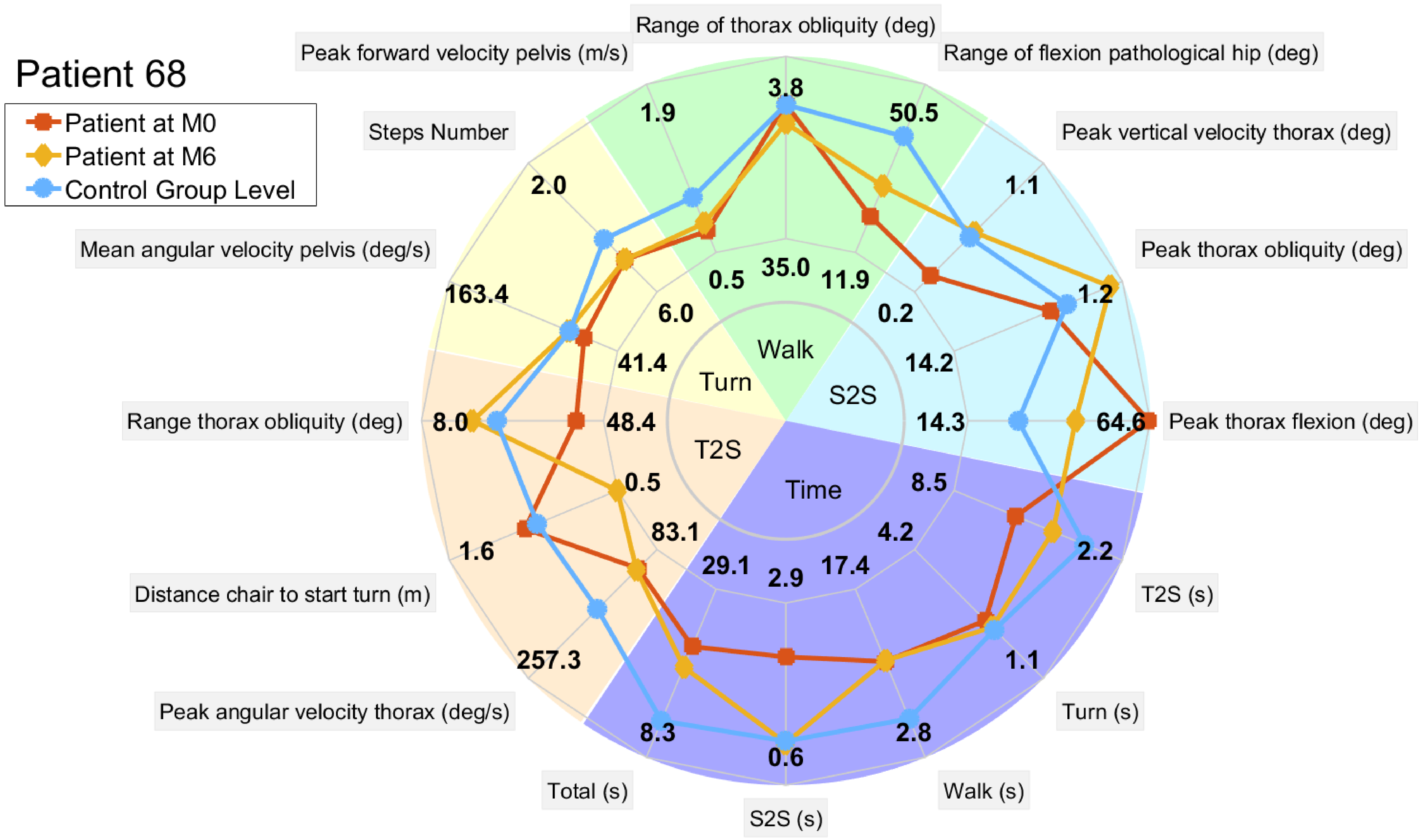

Patient 68
Patient at M0
Patient at M6
Control Group Level
Range of thorax obliquity (deg)
Range of flexion pathological hip (deg)
Peak vertical velocity thorax (deg)
Peak thorax obliquity (deg)
Peak thorax flexion (deg)
T2S (s)
Turn (s)
Walk (s)
S2S (s)
Total (s)
Peak angular velocity thorax (deg/s)
Distance chair to start turn (m)
Range thorax obliquity (deg)
Mean angular velocity pelvis (deg/s)
Steps Number
Peak forward velocity pelvis (m/s)
3.8
50.5
1.1
1.2
64.6
2.2
1.1
2.8
0.6
8.3
257.3
1.6
8.0
163.4
2.0
1.9
35.0
11.9
0.2
14.2
14.3
8.5
4.2
17.4
2.9
29.1
83.1
0.5
48.4
41.4
6.0
0.5
Walk
S2S
Time
T2S
Turn

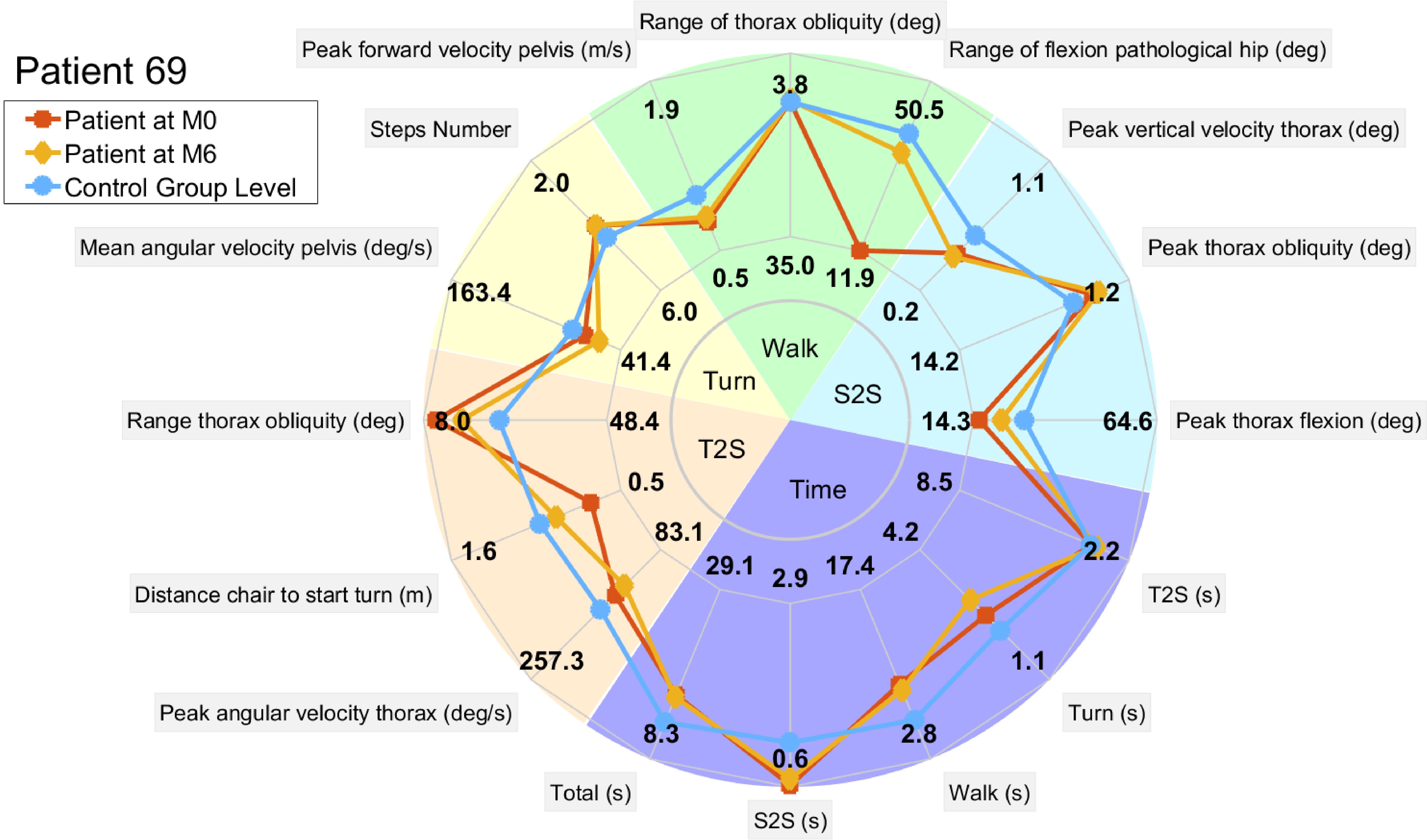

Patient 69
Patient at M0
Patient at M6
Control Group Level
Range of thorax obliquity (deg)
Peak forward velocity pelvis (m/s)
Range of flexion pathological hip (deg)
Steps Number
Peak vertical velocity thorax (deg)
Mean angular velocity pelvis (deg/s)
Peak thorax obliquity (deg)
Range thorax obliquity (deg)
Peak thorax flexion (deg)
Distance chair to start turn (m)
T2S (s)
Peak angular velocity thorax (deg/s)
Turn (s)
Total (s)
Walk (s)
S2S (s)
Walk
Turn
S2S
T2S
Time
3.8
1.9
50.5
2.0
1.1
163.4
1.2
8.0
64.6
1.6
2.2
257.3
1.1
8.3
2.8
0.6
35.0
0.5
11.9
6.0
0.2
41.4
14.2
48.4
14.3
0.5
8.5
83.1
4.2
29.1
2.9
17.4

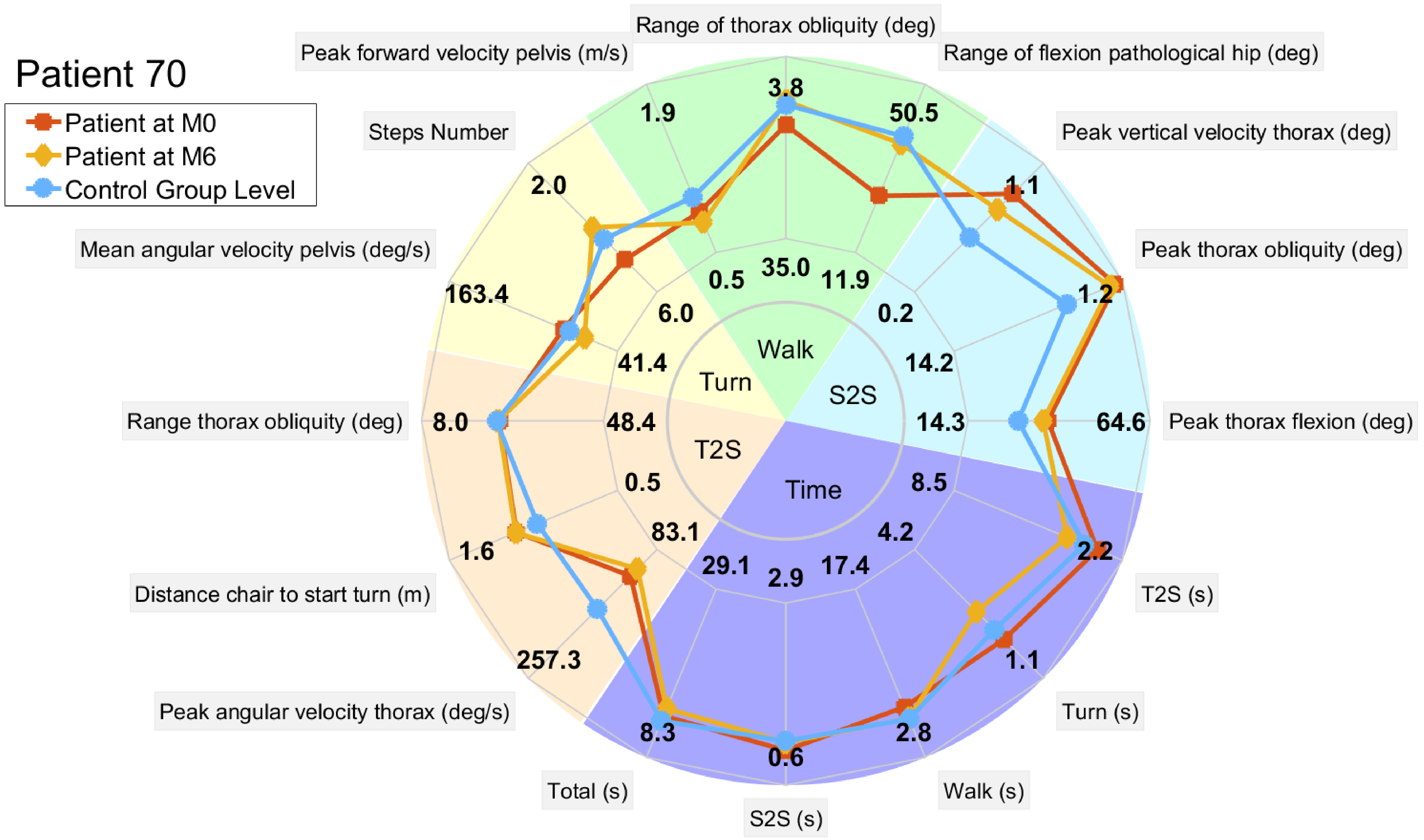
Patient 70
Patient at M0
Patient at M6
Control Group Level
Range of thorax obliquity (deg)
Peak forward velocity pelvis (m/s)
Range of flexion pathological hip (deg)
Peak vertical velocity thorax (deg)
Steps Number
Peak thorax obliquity (deg)
Mean angular velocity pelvis (deg/s)
Peak thorax flexion (deg)
Range thorax obliquity (deg)
Distance chair to start turn (m)
T2S (s)
Turn (s)
Peak angular velocity thorax (deg/s)
Walk (s)
Total (s)
S2S (s)
Walk
Turn
S2S
T2S
Time
3.8
1.9
50.5
1.1
2.0
1.2
163.4
64.6
8.0
2.2
1.6
1.1
257.3
8.3
2.8
0.6
35.0
0.5
11.9
6.0
0.2
41.4
14.2
48.4
14.3
0.5
8.5
83.1
4.2
29.1
2.9
17.4

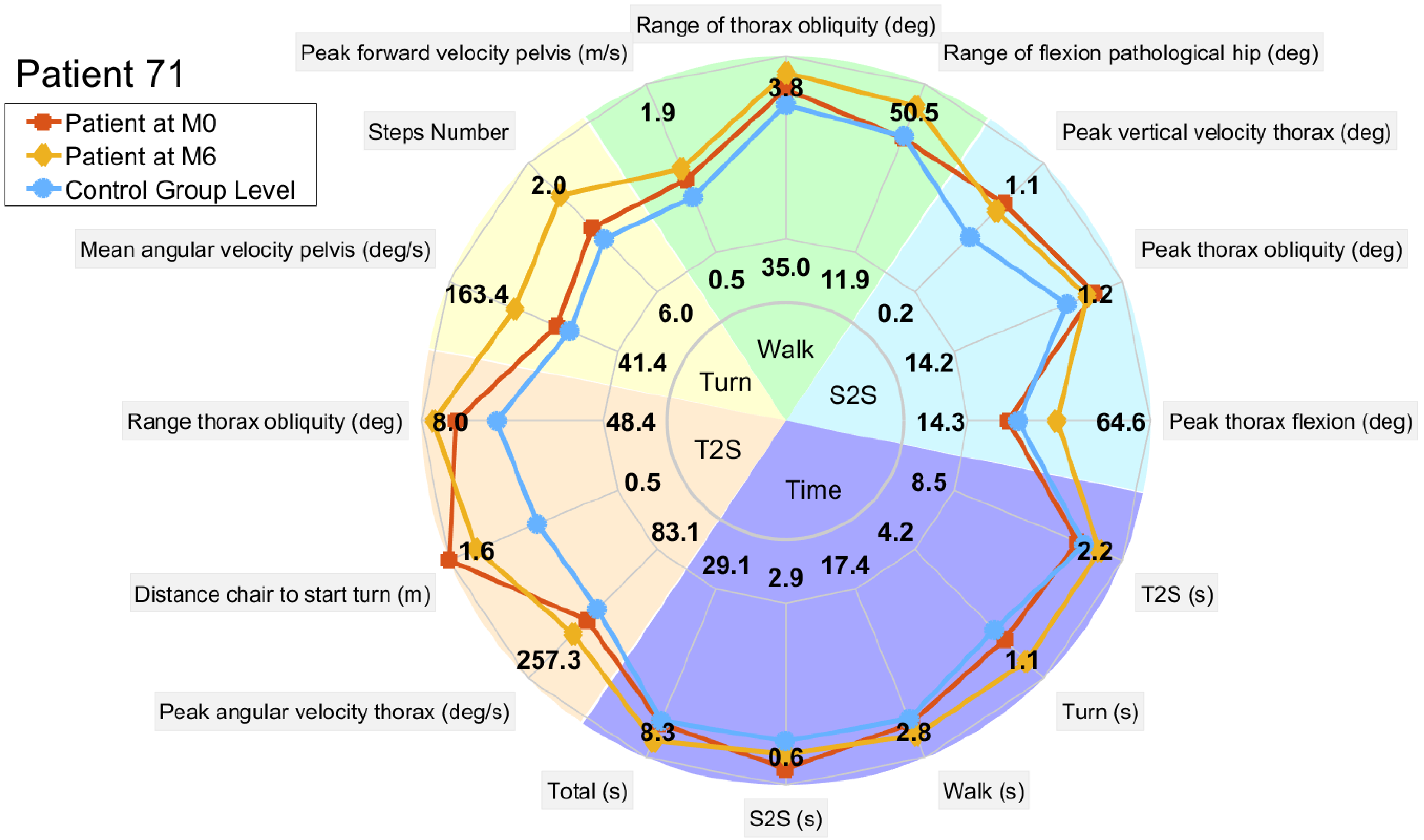

Patient 71
Patient at M0
Patient at M6
Control Group Level
Range of thorax obliquity (deg)
Range of flexion pathological hip (deg)
Peak vertical velocity thorax (deg)
Peak thorax obliquity (deg)
Peak thorax flexion (deg)
T2S (s)
Turn (s)
Walk (s)
S2S (s)
Total (s)
Peak angular velocity thorax (deg/s)
Distance chair to start turn (m)
Range thorax obliquity (deg)
Mean angular velocity pelvis (deg/s)
Steps Number
Peak forward velocity pelvis (m/s)
Walk
S2S
Time
T2S
Turn
3.8
50.5
1.1
1.2
64.6
2.2
1.1
2.8
0.6
8.3
257.3
1.6
8.0
163.4
2.0
1.9
35.0
11.9
0.2
14.2
14.3
8.5
4.2
17.4
2.9
29.1
83.1
0.5
48.4
41.4
6.0
0.5
