# Supplementary material for: Which functional tasks present the largest deficits for patients with total hip arthroplasty before and six months after surgery? A study of the timed up-and-go test phases
Source: PLoS One. 2021 Sep 10;16(9):e0255037. doi: 10.1371/journal.pone.0255037 (PMC8432811; doi:10.1371/journal.pone.0255037)
Supplement: S1 Table — (PDF) [file pone.0255037.s004.pdf]

1 - Selection of number of Principal Component with Cattell's scree test (Cattell, 1966)

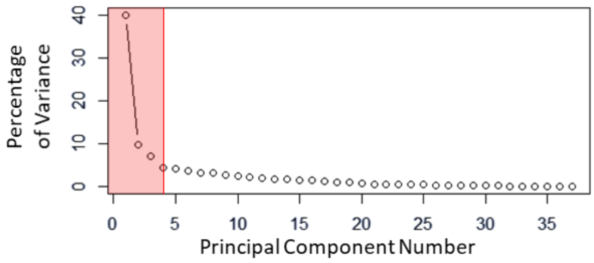

	% Variance	cumulative % Variance
PC 1	39.1	39.1
PC 2	10.0	49.0
PC 3	7.3	56.3
PC 4	4.7	61.0
PC 5	4.3	65.3
PC 6	3.8	69.0
PC 7	3.4	72.4
PC 8	3.2	75.6
PC 9	2.6	78.2
PC 10	2.4	80.6

2 – Selection of parameters based on cos2

	**Category**	**Feature**	**PC 1**	**PC 2**	**PC 3**
**Sit to Stand**	Quality	**Peak obliquity thorax**	0.03	0.12	**0.28**
		Range obliquity thorax	0.06	0.17	0.26
		**Peak flexion thorax**	0.03	**0.45**	0.02
		Width base of support	0.01	0.07	0.01
		Length base of support	0.00	0.00	0.16
	Speed	**Peak vertical velocity thorax**	**0.61**	0.03	0.07
		Peak vertical velocity pelvis	0.56	0.05	0.07
		Peak extension velocity of pathological hip	0.53	0.02	0.01
		Peak extension velocity of contralateral hip	0.52	0.03	0.01
**Walking**	Quality	RMS obliquity thorax	0.08	0.19	0.27
		**Range obliquity thorax**	0.13	0.20	**0.30**
		Lateral RMS C7	0.08	0.07	0.24
		Lateral range C7	0.20	0.04	0.21
		**Range of flexion pathological hip**	**0.50**	0.03	0.00
		Range of flexion contralateral hip	0.47	0.01	0.00
	Speed	Mean forward velocity pelvis	0.84	0.01	0.00
		**Peak forward velocity pelvis**	**0.86**	0.01	0.01
		Mean forward velocity thorax	0.83	0.01	0.00
		Peak forward velocity thorax	0.83	0.01	0.01
**Turning**	Quality	**Step number**	**0.21**	0.03	0.05
	Speed	Peak angular velocity thorax	0.16	0.01	0.10
		Mean angular velocity thorax	0.65	0.00	0.08
		Peak angular velocity pelvis	0.58	0.01	0.07
		**Mean angular velocity pelvis**	**0.66**	0.00	0.08
**Turn to sit**	Quality	Peak obliquity thorax	0.02	0.65	0.09
		**Range obliquity thorax**	0.02	**0.66**	0.04
		Peak flexion thorax	0.00	0.58	0.13
		**Distance chair to start of turn**	**0.23**	0.04	0.02
		Step number	0.09	0.04	0.01
	Speed	Peak vertical velocity thorax	0.47	0.03	0.01
		Peak vertical velocity pelvis	0.44	0.06	0.01
		Peak extension velocity of pathological hip	0.51	0.00	0.00
		Peak extension velocity of contralateral hip	0.39	0.03	0.00
		**Peak angular velocity thorax**	**0.76**	0.03	0.02
		Mean angular velocity thorax	0.65	0.00	0.01
		Peak angular velocity pelvis	0.74	0.01	0.03
		Mean angular velocity pelvis	0.68	0.00	0.02

Cattell, R.B., 1966. The Scree Test For The Number Of Factors. Multivariate Behav. Res. 1, 245–276
