# Supplementary material for: Which functional tasks present the largest deficits for patients with total hip arthroplasty before and six months after surgery? A study of the timed up-and-go test phases
Source: PLoS One. 2021 Sep 10;16(9):e0255037. doi: 10.1371/journal.pone.0255037 (PMC8432811; doi:10.1371/journal.pone.0255037)
Supplement: S1 File — (PDF) [file pone.0255037.s005.pdf]

## Algorithm to detect the events of the Timed Up-And-Go

**A - Start of the movement** [1]

1. **mSHO** = (**LSHO** + **RSHO**) / 2 — Were R/LSHO the right/left shoulder markers
2. **v** = d(**mSHO**)/dt — vx = velocity of mSHO in forward direction
3. **a** = $d^2$(**mSHO**)/$dt^2$ — ax = acceleration of mSHO in forward direction
4. VAX = ax * vx
5. VAX100 = mean(VAX(1:100)) Average of VAX on first 100 frames
6. Start of motion is first frame were VAX > VAX100 + $10^5$

**B - End of sit-to-stand** (Beyea et al, 2017)

First frame were height of mSHO is above 95% of the average height of mSHO during a static trial where the participant stands straight for 3 seconds.

**C - Start / End of turns**

1. Identification of T1 & T2 the midpoints of the 1st and 2nd turn
   - T1 is the first frame where (**LSHO** – **RSHO**).**y** = 0
   - **y** is the lateral component of the global frame
   - T2 the first frame where (**LSHO** – **RSHO**).**y** = 0
2. **VT** = d(**RSHO - LSHO**)/dt
3. **VTy** = **VT . y**
4. The start of the first turn is the first frame before T1 were **VTy** = **0**
5. The end of the first turn is the first frame after T1 were **VTy** = **0**
6. The start of the second turn is the first frame before T2 were **VTy** = **0**
7. The end of the second turn is the first frame after T2 were **VTy** = **0**

**D - End of motion**

1. The thorax flexion angle is computed according to the Conventional Gait Model [2]
2. T3 is the frame with the maximal thorax flexion angle after T2
3. The end of motion is the frame with the minimal thorax flexion angle after T3


[1] Beyea, J., Mcgibbon, C.A., Sexton, A., Noble, J., Connell, C.O., 2017. *Convergent Validity of a Wearable Sensor System for Measuring Sub-Task Performance during the Timed Up-And-Go Test.* Sensors 17, 1–18. https://doi.org/10.3390/s17040934

[2] Baker, R., Leboeuf, F., Reay, J., Sangeux, M., 2017. *The Conventional Gait Model - Success and Limitations*, in: Müller, B., Wolf, S.I. (Eds.), Handbook of Human Motion. Springer International Publishing AG, pp. 1–19. https://doi.org/10.1007/978-3-319-30808-1_25-2
