# Supplementary material for: Which functional tasks present the largest deficits for patients with total hip arthroplasty before and six months after surgery? A study of the timed up-and-go test phases
Source: PLoS One. 2021 Sep 10;16(9):e0255037. doi: 10.1371/journal.pone.0255037 (PMC8432811; doi:10.1371/journal.pone.0255037)
Supplement: S2 File — (PDF) [file pone.0255037.s006.pdf]

Feature computations & selection

1. Marker set

The marker set of the Conventional Gait Model was used [1] (Fig. 1). The hip joint centre was defined with the method of Hara [2] and the knee joint centre (KJC) with the chord method used by the Conventional Gait Model (Fig. 2).

The chord method consist in defining a plane with the markers HJC, THI and KNE, and then finding KJC as the 3rd point of a right triangle within this plane, with the distance KJC to KNE equal to half of the knee width.

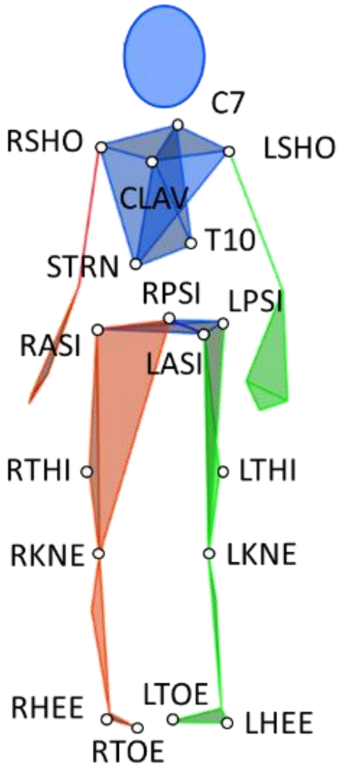

Figure 1: Skin markers

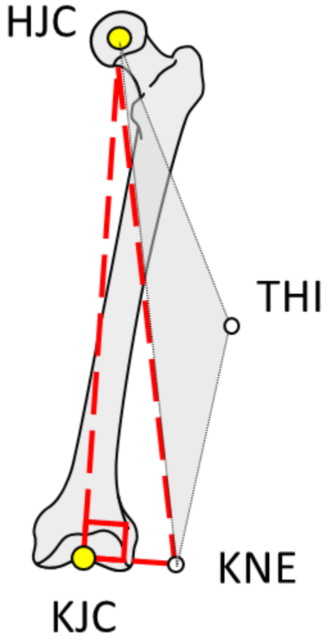

Figure 2: Representation of the chord method

2. Local Coordinate Systems

- The **thorax** LCS was based on [3] and was defined as follow:

$$\begin{cases} \mathbf{z}_{t/g} = \left((\mathbf{CLAV}+\mathbf{C7})|_g - (\mathbf{STRN}+\mathbf{T10})|_g \right) / \left\| \left((\mathbf{CLAV}+\mathbf{C7})|_g - (\mathbf{STRN}+\mathbf{T10})|_g \right) \right\| \\ \quad \mathbf{x}_{tmp} = (\mathbf{CLAV}+\mathbf{STRN})|_g - (\mathbf{C7}+\mathbf{T10})|_g) \\ \mathbf{y}_{t/g} = \left(\mathbf{z}_{t/g} \times \mathbf{x}_{tmp} \right) / \left\| \mathbf{z}_{t/g} \times \mathbf{x}_{tmp} \right\| \\ \mathbf{x}_{t/g} = \mathbf{y}_{t/g} \times \mathbf{z}_{t/g} \\ \mathbf{O}_{t/g} = \mathbf{CLAV}|_g - \mathbf{x}_{t/g} * r_m \end{cases}$$

$$\mathbf{R}_{g\to t} = \left[\mathbf{x}_{t/g} \quad \mathbf{y}_{t/g} \quad \mathbf{z}_{t/g} \right]$$

Where r_m is the radius of the skin markers

- The **pelvis** LCS was based on CGM conventions [1] and was defined as follow:

$$\begin{cases} \mathbf{y}_{p/g} = \left(\mathbf{LASI}|_g - \mathbf{RASI}|_g \right) / \left\| \left(\mathbf{LASI}|_g - \mathbf{RASI}|_g \right) \right\| \\ \quad \mathbf{x}_{tmp} = (\mathbf{RASI}|_g - (\mathbf{RPSI}+\mathbf{LPSI})|_g / 2) \\ \mathbf{z}_{p/g} = \left(\mathbf{x}_{tmp} \times \mathbf{y}_{p/g} \right) / \left\| \mathbf{x}_{tmp} \times \mathbf{y}_{p/g} \right\| \\ \mathbf{x}_{p/g} = \mathbf{y}_{p/g} \times \mathbf{z}_{p/g} \\ \mathbf{O}_{p/g} = (\mathbf{RPSI}+\mathbf{LPSI})|_g / 2 - \mathbf{x}_{p/g} * r_m \end{cases}$$

$$\mathbf{R}_{g\to p} = \left[\mathbf{x}_{p/g} \quad \mathbf{y}_{p/g} \quad \mathbf{z}_{p/g} \right]$$

- The **thigh** LCS was based on CGM conventions [1] and was defined as follow:
  The Knee Joint Centre (KJC) was defined with the chord method [4]

$$\begin{cases} \mathbf{z}_{thi/g} = \left(\mathbf{HJC}|_g - \mathbf{KJC}|_g \right) / \left\| \left(\mathbf{HJC}|_g - \mathbf{KJC}|_g \right) \right\| \\ \mathbf{y}_{thi/g} = \left(\mathbf{KNE}|_g - \mathbf{KJC}|_g \right) / \left\| \mathbf{KNE}|_g - \mathbf{KJC}|_g \right\| \\ \mathbf{x}_{thi/g} = \mathbf{y}_{thi/g} \times \mathbf{z}_{thi/g} \\ \mathbf{O}_{t/g} = \mathbf{KJC}|_g \end{cases}$$

$$\mathbf{R}_{g\to t} = \left[\mathbf{x}_{p/t} \quad \mathbf{y}_{p/t} \quad \mathbf{z}_{p/t} \right]$$

- The thigh rotation matrix was computed as follow: $\mathbf{R}_{p\to thi} = \left(\mathbf{R}_{g\to p} \right)^{-1} . \mathbf{R}_{g\to thi}$

3. Joint Angles

- Thorax: sequence of Cardan angle YXZ: Tilt / Obliquity / Axial Rotation
- Hip: Sequence of Cardan angle YXZ: Flex-Ext / Add-Abd / Int-Ext Rot

4. Overview of features

Feature	Phase	Definition	References & hypotheses
**Quality**			
Peak obliquity thorax	S2S & T2S	Maximal value of thorax obliquity.	Trunk movement was defined as the 3rd determinant of sit-to-stand [5]. More specifically Abujaber et al [6] reported that patients with THA had "significant lateral trunk angle toward the operated side" during sit-to-stand before and 3 months after surgery. Thorax obliquity seems relevant to represent the asymmetry during standing and sitting.<br>The peak value (adapted from [6]) and range of obliquity [7] were selected in the study.<br>It is expected that patients will have higher values than the control group (CG) and will reduce obliquity at 6 month after the surgery (M6).
Range obliquity thorax	S2S & T2S	Difference between the maximal values of obliquity on the right and left side.	
Peak flexion thorax	S2S & T2S	Maximal value of thorax flexion.	Trunk movement was defined as the 3rd determinant of sit-to-stand [5]. Older adults were reported to use a stabilisation strategy during S2S [8] where the upper body is positioned above the base of support, thus increasing thorax flexion [9].<br>It is expected that patients will have increased thorax flexion with respect to the CG and that thorax flexion will be reduced at M6.
Width base of support	S2S	Vector between the midpoint of the TOE and HEE markers of the right and left foot projected on the lateral axis of the laboratory. The value is taken at time of maximal trunk flexion.	Foot position was defined as the 2nd determinant of sit-to-stand [5]. Older adults were reported to use a stabilisation strategy during S2S [8] where the upper body is positioned above the base of support. Increasing the width of the base of support will improve the stability.<br>It is expected that patients will have a wider base of support than the CG and that this width will be reduced at M6.
Length base of support	S2S	Vector between the midpoint of the TOE and HEE markers of the right and left foot projected on the anterior axis of the laboratory. The value is taken at time of maximal trunk flexion.	After observing patients during measurements, it seems that patients have feet aligned in the anterior direction to have a square base of support and increase stability. CG tend to use a momentum transfer strategy with one foot forward and one close to the chair to initiate gait while standing up. It is expected that patients will have the feet aligned (smaller length than CG).

RMS obliquity thorax	Walking	Root mean square of the obliquity of the thorax during the walking phase.	Patients with hip osteoarthritis show increased amplitude of lateral trunk displacement [10] when compared to a control group. Those parameters were selected to assess this phenomenon. C7 was chosen instead of the midpoint between the acromions used in [10].<br><br>It is expected that the range and RMS will be higher for patients when compared to the CG and that it will be reduced at M6.
Lateral RMS C7	Walking	Root mean square of the trajectory of C7 projected on the lateral axis of the laboratory.	
Lateral range C7 lateral	Walking	Range of the trajectory of C7 projected on the lateral axis of the laboratory.	
Range of flex/ext pathological hip	Walking	For the control group, the average of the ranges of the right and left hip.	The hip range of flexion/extension is among the main limitations in patients with THA [11]. It is expected that the range of the pathological hip will be lower than the CG and that it will increase at M6. The range of the contralateral hip was also considered.
Range of flex/ext contralateral hip	Walking	For the control group, the average of the ranges of the right and left hip.	
Step number	Turn	The step number was based on the peak values of the right and left heel markers vertical velocity (RHEE and LHEE).	The step number during turns was used in previous iTUG studies [7,12].<br><br>It is expected that patients will have higher number of steps than CG and will reduce number at M6.
Side of turn	Turn	The side of turn was identified by checking which shoulder was the farthest from the chair. If the right shoulder is the farthest, the rotation is around the left leg and conversely.	It was hypothesised that patients would prefer turning on the side of the pathological hip to reduce hip loading and pain.
Distance chair to start of turn	T2S	The distance between the positions of the midpoint of the pelvis markers at the start of the turn and at the end of the sitting phase in the forward direction.	Two strategies were described in the literature [13] : distinct transition strategy and overlapping transition strategy. During measurements, it was observed that patients tended to turn closer to the chair than CG before sitting.<br><br>A larger distance will indicate a tendency toward overlapping transition strategy and a shorter distance a tendency toward distinct transition strategy. It is expected that patients will have a smaller distance than CG and that the distance will increase at M6.

**Speed**			
Peak vertical velocity thorax / pelvis	S2S & T2S	Peak vertical velocity of the midpoint of the thorax/pelvis markers expressed in the coordinate system of the laboratory	This feature was previously used to quantify the performance of S2S in elderly patients [14]. It is expected that patients will have lower peak vertical velocities than CG and that it will increase at M6.
Peak extension velocity of pathological / contralateral hip	S2S & T2S	Peak value of the first time derivative of the pathological/contralateral hip flexion-extension angle during sit-to-stand	This feature was previously used to study S2S in patients with THA [15]. It is expected that patients will have reduced peak extension velocity of the hip when compared to CG and that it will increase at M6.
Mean forward velocity thorax / pelvis	Walking	Mean forward velocity of the midpoint of the thorax/pelvis markers expressed in the coordinate system of the laboratory	Gait speed is significantly reduced in patients with THA [11] and is a key factor when studying gait. Two methods of computation of the gait speed were used, mean value and peak value [7].<br>It is expected that patients will have a lower walking speed than CG and that this feature will increase at M6.
Peak forward velocity thorax / pelvis	Walking	Peak forward velocity of the midpoint of the thorax/pelvis markers expressed in the coordinate system of the laboratory	
Peak angular velocity thorax / pelvis	Turn & T2S	Peak angular velocity of the thorax/pelvis expressed around the Z-axis (vertical) of the segment	The angular velocity of the turn is commonly used to assess turn in iTUG studies. Salarian et al. [7] used the peak angular velocity while Zampieri et al. [16] use mean angular velocity.<br>It is expected that patients will have lower turning velocities than patients and that this feature will increase at M6.
Mean angular velocity thorax / pelvis	Turn & T2S	Mean angular velocity of the thorax/pelvis expressed around the Z-axis (vertical) of the segment	

5. References

1. Baker R, Leboeuf F, Reay J, Sangeux M. The Conventional Gait Model - Success and Limitations. In: Müller B, Wolf SI, editors. Handbook of Human Motion. Springer International Publishing AG; 2017. pp. 1–19. doi:10.1007/978-3-319-30808-1_25-2
2. Hara R, McGinley J, Briggs C, Baker R, Sangeux M. Predicting the location of the hip joint centres, impact of age group and sex. Sci Rep. 2016;6: 1–9. doi:10.1038/srep37707
3. Gutierrez EM, Bartonek Å, Haglund-Åkerlind Y, Saraste H. Centre of mass motion during gait in persons with myelomeningocele. Gait Posture. 2003;18: 37–46. doi:10.1016/S0966-6362(02)00192-3
4. Baker R. Measuring Walking: A Handbook of Clinical Gait Analysis. Mac Keith Press; 2013.

5. Janssen WGM, Bussmann HBJ, Stam HJ. Determinants of the sit-to-stand movement: A review. Phys Ther. 2002;82: 866–879. doi:10.1093/ptj/82.9.866

6. Abujaber SB, Marmon AR, Pozzi F, Rubano JJ, Zeni JA. Sit-To-Stand Biomechanics Before and After Total Hip Arthroplasty. J Arthroplasty. 2015;30: 2027–2033. doi:10.1016/j.arth.2015.05.024

7. Salarian A, Horak FB, Zampieri C, Carlson-kuhta P, Nutt JG, Aminian K. iTUG , a Sensitive and Reliable Measure of Mobility. IEEE Trans neural Syst Rehabil Eng. 2010;18: 303–310.

8. Aissaoui R, Dansereau J. Biomechanical analysis and modelling of sit to stand task: a literature review. Proc IEEE Int Conf Syst Man Cybern. 1999;1: 141–146. doi:10.1109/icsmc.1999.814072

9. Alexander NB, Schultz AB, Warwich DN. Rising from a chair: Effects of age functional ability on performance biomechanics. Journals Gerontol. 1991;46: 91–98. doi:10.1093/geronj/46.3.m91

10. Nankaku M, Tsuboyama T, Kakinoki R, Kawanabe K, Kanzaki H, Mito Y, et al. Gait analysis of patients in early stages after total hip arthroplasty: Effect of lateral trunk displacement on walking efficiency. J Orthop Sci. 2007;12: 550–554. doi:10.1007/s00776-007-1178-2

11. Bahl JS, Nelson MJ, Taylor M, Solomon LB, Arnold JB, Thewlis D. Biomechanical changes and recovery of gait function after total hip arthroplasty for osteoarthritis : a systematic review and meta-analysis. Osteoarthr Cartil. 2018;26: 847–863. doi:10.1016/j.joca.2018.02.897

12. Hollands KL, Hollands MA, Zietz D, Miles Wing A, Wright C, Van Vliet P. Kinematics of turning 180° during the timed up and go in stroke survivors with and without falls history. Neurorehabil Neural Repair. 2010;24: 358–367. doi:10.1177/1545968309348508

13. Weiss A, Mirelman A, Giladi N, Barnes LL, Bennett DA, Buchman AS, et al. Transition between the Timed Up and Go turn to sit subtasks: is timing everything? J Am Med Dir Assoc. 2016;17. doi:10.1016/j.physbeh.2017.03.040

14. Schot PK, Knutzen KM, Poole SM, Mrotek LA. Sit-to-stand performance of older adults following strength training. Res Q Exerc Sport. 2003;74: 1–8. doi:10.1080/02701367.2003.10609058

15. Boonstra MC, Schreurs BW, Verdonschot N. The Sit-to-Stand Movement : Patients After Primary Total Hip. Phys Ther. 2011;91: 547–554.

16. Zampieri C, Salarian A, Carlson-kuhta P, Aminian K, Nutt JG, Horak FB. The instrumented timed up and go test : potential outcome measure for disease modifying therapies in Parkinson's disease. J Neurol Neurosurg Psychiatry. 2010;81: 171–176. doi:10.1136/jnnp.2009.173740
