# Supplementary material for: Which functional tasks present the largest deficits for patients with total hip arthroplasty before and six months after surgery? A study of the timed up-and-go test phases
Source: PLoS One. 2021 Sep 10;16(9):e0255037. doi: 10.1371/journal.pone.0255037 (PMC8432811; doi:10.1371/journal.pone.0255037)
Supplement: Attachment — Submitted filename: Response to reviewers.docx [file pone.0255037.s007.docx]

Dear editor and reviewers,

We would like to thank the reviewers for their thorough analysis of our paper and the highly relevant observations made on the method, presentation of results, and points to clarify. These comments helped us increase significantly the quality of the paper as well as clarifying the outcome of the study.

The following document gives a detailed answer of the comments of each reviewers. Our answers are written in blue after each comments of the reviewers.

We hope that these answer will satisfy the reviewers and we remain at your disposal for any additional clarifications,

Best regards,

The authors

**Reviewer #1:**

**General**

The study investigated changes in an instrumented TUG test in patients before and 6 months after total hip replacement and compared them to healthy controls. They predefined biomechanical features according to the literature and used a PCA analysis to reduce the number of features. The selected features were then compared between groups. However, the description of the biomechanical features and especially the statistical analysis section is insufficient and makes it difficult completely follow and understand the analysis and results. If clarified, the paper presents interesting and clinically relevant data of a simple test that could be used in clinical practice to assess the functional outcome after total hip replacement.

The statistical analysis and its description was modified based on the comments of reviewer 1 and 2 and we added a supporting information file (S4) to give details on the computation of the biomechanical features and their selection.

**Major comments:**

Analysis/Table 2: The biomechanical features are insufficiently explained. Abbreviations are not explained (table 2). How was angular velocity of thorax/pelvis defined? In a plane/resultant? With respect to what point/segment? How was side of turn defined? How was pathological/contralateral defined in controls?

A supplementary material that defines the various features and gives more details in the selection was added and mentioned in the manuscript.

L 152: “The choice of the features and their definitions are detailed in Supporting Information S4.”

We believe that including the full definition in the manuscript would impede the readability of the paper. For the control group, the features of both hips were averaged, thus the “pathological” and “contralateral” features are equal.

**Statistics**

How were the biomechanical features reduced?

The description of the PCA was modified as follow to give more details:

L 157: “A principal component analysis (PCA) was performed to keep only the most relevant biomechanical features in the analysis (excluding the time of the phases). The feature were centred and reduced by subtracting the mean and dividing by the standard deviation, to account for the difference of scales. All three groups (patients at M0, patients at M6 and control group) were included in the analysis.”

You state that you kept one feature per component and per subtask. What do you mean by component? PC component?

Yes, we mean PC component, it was added to the manuscript for clarity (L 162).

Why do you keep Peak angular velocity thorax and peak vertical velocity thorax in turn to sit which are both associated with PC1 (table 3)?

Indeed, both features are associated with PC1. The turn-to-sit phase is composed of two movements, turning and sitting, thus although we grouped those two movements in one phase, we wanted to keep a feature per movement. However, we did this for the speed features but not quality feature. This is questionable and thus we removed the feature “peak vertical velocity of thorax” from the analysis for methodological consistency.

Bonferroni correction: what is the number of tests?

For the differences in features between groups (Table 4), there were 16 tests per group comparison (one for each feature) and 3 group comparisons. The number of tests was 48 leading to a corrected p-value of 0.05/48 = 0.001.

For the comparison of the differences between groups at phase level, there were 4 tests per group comparison and 3 group comparisons. The number of tests was 12 leading to a corrected p-value of 0.05/12 = 0.004.

Reviewer 2 suggested that we use the Holm method instead of the Bonferroni method. We removed the mentions to Bonferroni and used the Holm method instead (L 166).

It is unclear why calculate the percentage of variation. From my understanding of the results, this is not presented anywhere and not related to the aims of the study. Please clarify.

What we meant by “percentage of variation” was the change in patient’s function between M0 and M6 expressed in percentage of M0. These results are presented in the results, in the section “Evolution of function six months after surgery”. The manuscript was modified as follow to avoid misunderstandings:

L 172: “The change between M0 and M6 was expressed in percentage of the feature value at M0 to express the increase or decrease in function after surgery.”

**Results**

You often write about a deficit. But it is not explained in the methods how this deficit was defined.

An explanation of the deficits was added to the method in the Statistics section:

L 169: “A lower function of the patients with respect to the control group was described as a deficit. Depending on the feature, a lower function can be a higher value (e.g. duration of phase) or a lower value (e.g. peak vertical velocity of the thorax for the S2S).”

Numbers in tables, figures and text are repeatedly different from each other even though they should be same (i.e. Fig.2 and Table 4 values for control group).

The consistency of the numbers in the text, figures and tables were checked.

Why do you use different thresholds for presenting p-values (i.e. Table 4 and Fig 2. once p<0.001, once p<0.003)?

This is a typo, the correct value was p < 0.001, since the Holm method is now used, it was corrected in Fig. 2 as p < Holm correction.

You write about a statistically larger deficit in some phases. It is difficult to understand how you derive these results. It would be helpful to directly show the results of the statistical comparison between subtasks (incl p-values and corresponding threshold of Bonferroni correction)

As suggested by reviewer 2, we are now using the Holm correction, thus we removed mentions to the Bonferroni correction. To clarify, we mentioned in the manuscript, that the deficits were significant with the Holm method (L 214, L 230 and L 236). The Holm method as a varying level of p-value to assess significance of the test and it seemed that reporting both number would impede readability of the manuscript. However, if the reviewer sees fit, we can report both values for the tests of the differences between phases.

Figure 2: The figure caption states that Fig 2 presents an overview about the features selected by the PCA. However, it also contains the duration of the different TUG phases. From my understanding, these were not included in the PCA. Please clarify.

Indeed, the duration of the phases was not included in the PCA, we changed slightly the title of Fig2 as follow: “Overview of the duration of the phases and features selected by the PCA for the three groups […]”

Moreover, an explanation on how the points in the plot were selected is missing (sometimes larger number is inside, sometimes outside. How was position of point for M6 defined?).

To avoid having lines crossing each other and make the figure easier to read, we chose to have the control group values on the outside and M0 value on the inside. In this way, an improvement of patients is visually seen as their score by going toward the outside part of the plot. In some cases a lower value will be an improvement (e.g. shorter duration of phase), in other cases a higher value will be an improvement (e.g. higher peak linear velocity of pelvis).

A short explanation was added to the legend of Fig.2:

L 196: “Feature values representing higher function are on the outer edge and lower function values are on the inner edge.”

**Minor comments:**

**Abstract**

Line 38: Do you mean “improve”? What parameter? Shorter duration of the phases?

Yes, we changed to ‘improved’. This statement is related to the results with “one global figure per phase” (Table 5). We kept this statement general for the abstract due to the word limitations but if the reviewer thinks it is needed, we can add some more details.

Line 39-41: You conclude that the iTUG could be instrumented with a wearable sensor in a clinical setting. However, from your paper, it’s not clear how that would be achieved. Please revise.

Three inertial measurement units taped to the thorax, pelvis and thigh on the side of surgery could be used to compute the same features that we computed in this paper. This was mentioned in the discussion in the section “Implementation in clinical settings” (L 327).

The mention of the Inertial Measurement Units was removed from the abstract to avoid confusion, but kept in the discussion. The abstract was modified as follow:

L 39: “This simple test could be relevant to quantify patient-specific deficits of function to guide and follow post-operative rehabilitation in clinical settings.”

**Introduction**

Line 47/48: This should be “6 months and 1 year after THA”

The manuscript was changed accordingly.

Lines 61-62: Please write “test” after each test (6minute walk test, 30s chair stand test or TUG test), or only after TUG test.

The manuscript was changed accordingly.

Lines 78ff: Please specify what parameters are measured/analyzed in the iTUG.

The parameters measured/analysed in the iTUG depend on the studies. The point was to say that the iTUG provides biomechanical parameters that can lead to a deeper understanding of the pathologies that are studied. The relevant parameters will differ from pathology to pathology.

**Methods**

Lines 107-109: You state that all patients had either stage II, III or IV hip OA, but in Table 1 K/L classification is missing for 4 patients. Why are these data missing?

Those four patients performed X-rays outside of the Hospital and didn’t bring the X-rays during their visits. It was not possible to evaluate their OA stage with the K/L classification. If the reviewer sees it appropriate, we can mention it in the manuscript.

Line 118: Please change to “destabilised” (you used British English spelling in the rest of the manuscript).

The manuscript was changed accordingly.

Line 119-120: Please change to “between groups” (or “between populations”)

The manuscript was changed accordingly.

Line 131: There is a space missing between “chair” and “without”

The manuscript was changed accordingly.

Lines 133ff: Why did you perform 10 TUGs but only analyze the fastest trial of the first 3 TUGs? Please explain.

The standard way of performing the TUG test is to perform two tests and keep the fastest time (Podsialdo et al. 1991). Ten measurements were performed for a secondary study that would evaluate the variability of the test. We kept only the first 3 for this analysis to be close to the initial definition of the TUG test and, because for some patients, the measurement of the first or second trial failed. If the reviewer sees it appropriate, we can mention it in the manuscript.

- Podsiadlo D, Richardson S. The Timed “Up & Go”: A Test of Basic Functional Mobility for Frail Elderly Persons. J Am Geriatr Soc. 1991;39: 142–148.

Line 147/S1: Is there any information on what S1 shows? The figure is not self-explanatory, it’s not clear what the sticks indicate.

We added comments on the first figure of S1 and S2 to guide the reader. If the reviewer sees fit we can add further explanations.

Line 175: Please change to “one global figure per phase”.

The manuscript was changed accordingly.

Line 176: I think you mean “development” instead of “evolution”. Please change throughout the manuscript (evolution and evolve).

We modified “evolution” by “change” throughout the manuscript.

**Results** (there are no line numbers after page 14, so comments were made on paragraphs)

Line numbers were added to the whole document.

Table 4: Please check that you use the same precision for mean and SD.

This was corrected in the table.

Was the SD of mean angular velocity pelvis (turn) and peak angular velocity thorax (turn to sit) really below 1?

No, this is a typo.

Line 206: Table 6 appears before Table 5 in the text. Please change

The manuscript was changed accordingly, the number of Table 6 was changed to 5 and conversely.

Line 209: This should be “Bonferroni correction”

The method to assess the significant differences was changed, the “Holm method” is now mentioned.

Table 5: What do bold numbers indicate? What is parameter corr. total time?

The bold numbers were used to outline results during our analysis and were removed from the manuscript.

The parameter “corr. total time” is the coefficient of correlation of the considered feature with the total time of the TUG. It was changed to “R total time” in the table and a description was added to the legend.

Table 6: Please specify in legend, what parameter is meant by percentage.

The definition of the percentages was done in the Statistics section of the method:

L 168: “To evaluate the average difference of the patients’ function with the control group, the values of the features at M0 and M6 were reported in percentage of the mean of the control group. A lower function of the patients with respect to the control group was described as a deficit. Depending on the feature, a lower function was either a higher value (e.g. duration of phase) or a lower value (e.g. peak vertical velocity of the thorax for the S2S). The change between M0 and M6 was expressed in percentage of the feature value at M0 to express the increase or decrease in function after surgery. Furthermore, the percentage of patients at the level of the control group (i.e. within 1 standard deviation of the control group) was evaluated, as well as the percentage of patients with an increase in function between M0 and M6. The percentages of differences with the control group and the percentage of change between M0 and M6 were correlated with the differences and change of the total time.”

The legend of the table was modified as follow for clarity:

“Table 5: Mean difference ±SD (95% confidence interval) between groups at phase level expressed in percentages”

Table 5/6: Were the 95% CI of the difference really that much smaller than the SD of the difference?

Yes, we used the formula 95%CI = mean ± 1.96 * SD / sqrt(n) with n the number of patients. As we had 71 patients it reduces the expected range of the 95% CI.

As an example, for the values of the S2S comparison of patients M0 vs CG, the mean and SD of the difference for the 71 patients are respectively -30% and 37% which lead to:

- A lower bound of: -30 – 1.96 * 37 / sqrt(71) = -39

- An upper bound of: -30 + 1.96 * 37 / sqrt(71) = -21

P.14, paragraph 1, last sentence (49-86%): why do you here include all parameters and in previous paragraph only those with significant differences?

This is a mistake, we modified the manuscript as follow:

L 231: “Only 27% of patients had a range of hip flexion-extension during walking at the level of the controls while the other significant features had 46% to 62% of patients at this level (Table 5).”

**Discussion**

P.15, paragraph 2: what scores of the patients? This implies to me that you compared PC scores, but from the results it’s not what you did. Please clarify.

With “scores of the patients”, we meant the values of the features for the patients. As this might be misleading, we modified the manuscript as follow:

L 259: “To that end, the TUG test was divided into four phases [15] representing different daily living activities (sit-to-stand, walking, turning and turn-to-sit), multiple features were selected by PCA and finally, the features of the patients were compared before surgery and at 6 months after surgery and to a control group for each phase.”

P.15, paragraph 3, last sentence: You state that this (hip flex/ext range walking phase) has a strong positive effect. However, only 20% of patients reach level of controls. Isn’t this contradictory?

We meant that this was the feature with the largest number of patients with an increase (82%). We modified this part of the discussion as follow:

L 269: “This feature had the lowest number of patients at the level of the control groups before and 6 months after surgery (8% and 27% respectively). However, this feature showed the highest number of patients with a positive change in function 6 months after surgery (82%). This suggests that, despite the deficits 6 months after surgery, THA has a positive effect on the sagittal hip kinematics during walking.”

P.17, variability of outcomes: From the presented results, I don’t understand how you analysed variability.

The SD values on the percentage of deficits at feature level and phase level were very high. The SD values were often at the same level as the mean and even larger than the mean for some cases (Table5 and 6). In our opinion, this indicates a high variability in the profiles of the patients.

This idea was clarified in the manuscript:

L 299: “However, the SD levels of the percentage of differences between groups where very high, e.g. the SD of the deficits of the total TUG time at M0 was 38% while the mean deficits was 39%. These high levels of SD suggests the high variability of profiles in patients. Indeed, some patients were at the level of control groups while others did not improve their function at M6 (Supporting Information S6).”

P.17, paragraph 1: were there any patients that had decreased performance 6 months postoperative?

Yes, some patients had decreased performance in multiple feature. We did not see patients with a clear decrease on all feature. The supplementary file S6 shows the values of the features at M0 and M6 of all patients compared to the control groups. As examples, patient 22 had a decrease in the duration of most phases and patient 42 had a decrease on most of the features. On the contrary, patient 7 had an increase for most of the features.

P.17, paragraph 2: please change “strong post-surgery deficit” to “large post-surgery deficit”

The manuscript was changed accordingly.

P.18, paragraph 1: Please change to “three synchronized IMUs…”

The manuscript was changed accordingly.

P.18, paragraph 2: you state that a simple 1 IMU/smartphone provides enough parameters to assess the function of the patients. However, from your analysis this is not valid. The presented parameters were not measured with IMUs and would need several sensors (data on pelvis, hip, thorax…). Please elaborate on how such a sensor should be set up according to your results.

To obtain the same features we would need IMUs on thorax, pelvis and thigh of the pathological side. However, this setup could be cumbersome in clinical settings and we suggest that a setup with one IMU would have more chances to be used in such settings. We expect that one IMU on the thorax could provide sufficient information to assess the function of the patients, but it could not provide the same features as the one described in this study. We removed this part from the manuscript as it is more a speculation and because the present paper does not have results supporting this claim.

P.18, paragraph 2, last 4 lines: Please use either “a test” or “tests”. Please change to “However, further studies are needed…”

The manuscript was changed accordingly.

P.18, paragraph 4. Please change to “duration of the phases” instead of “time of the phases”.

The manuscript was changed accordingly.

P.19, paragraph 1: You state that there was a study on minimal detectable changes of iTUG parameters. How do the difference between pre and post patients compare to these minimal detectable changes? Please elaborate.

The sentence we used could lead to misinterpretation. The study by Smith et al. (2020) reported in the paper assessed only the MDC of the total time evaluated measured with a commercial iTUG system but did not report MDC for the parameters of the TUG. However, they reported the effect size “to quantify the change observed in each of the [i]TUG parameters”. We modified the sentence in the manuscript as follow:

L 336: “Recently, the responsiveness to change of multiple parameters of the iTUG and minimal detectable change of the total time have been evaluated among older adults (63.9 ± 6.1 years) undergoing a six-week physiotherapy program with encouraging results [36]. Indeed, the responsiveness to change was shown with small to moderate effect sizes and the MDC of the total time was 0.77s.”

P.20, paragraph 1: You abbreviated timed-up and go test as TUG test, please change.

The manuscript was changed accordingly.

Reviewer #2

The study explores functional deficits before and its change after total hip arthroplasty with an instrumented timed up-and-go test, analyzing phase specific differences between patients and a control group. The topic is of clinical relevance and the sample size adequate to produce meaningful results. The relevance and experimental setup are well described and introduced. Limitations are within the study design, where no post-measurement of the control group is available, resulting in multiple testing and a reduction in statistical power. Further methodological shortcomings, including the variable selection, reduce the significance of the results. In some cases, false or non-ideal statistical tests are used and in other cases (e.g. PCA), methods are not well enough described to judge the correctness of the results. Combined with further language and typing errors, major revisions are necessary to improve the manuscript. Nevertheless, the study has the potential to come to good and meaningful results.

**Major Revisions:** To improve the study/manuscript following main issues should be revised:

1. The preselection of variables is arbitrary and not theory or hypothesis driven. The sole explanation are previous studies, who investigated other disorders and included further variables which have not been considered in the current study (e.g. spatiotemporal variables, such as cadence, stride length, length of single support…). Ideally the variables should be based on known limitations in hip OA patients, identified in previous studies. Furthermore, the joint angles over the complete range of motion (ROM) are unspecific, making conclusions about deficits for specific movements or walking phases difficult. For example, dividing hip ROM in flexion and extension would help to interpret whether deficits during walking are more related to the late stance phase (restricted hip extension), indicating problems to generating step length or swing phase (restricted hip flexion), which might result in higher fall risk due to lower foot clearance. It is unclear why peak thorax obliquity was only analysed during sit2stand/turn2sit and not walking/turning and why peak thorax obliquity was not directly investigated of the pathological side? Conversely, what is the rationale behind investigating different mean velocities of the pelvis and thorax. In which scenario do the authors expect to find notable differences in the mean velocities of these segments?

A table was added to supporting information with more details on the choice of the features.

The spatiotemporal parameters of gait were not selected due to the very short distance of walking that was below or equal to 3m. The target for turn was at 3m from the table, but, when considering that some participants started turning before the target and some started walking while standing up, the distance of walking was often below 3m. Previous studies showed that 5 steps are required to have stable variability of gait parameters [1] and a modified TUG with 7m of walking was recommended to assess spatiotemporal parameters during iTUG [2]. However a distance of 2.4m to 3m was found sufficient to measure gait speed [3].

Regarding the hip ROM of flexion-extension, this parameters was selected as it is commonly used in THA literature [4]. We agree that dividing the ROM in flexion and extension would be an interesting to understand patient’s limitation but that it would be more appropriate for a specific study focused on gait.

Regarding thorax and pelvis velocities, iTUG studies sometimes use angular/linear velocities of the pelvis [5] (or waist [6]), and sometime velocities of the thorax [2]. We chose to take both in the analysis and to identify the most relevant in this specific study with PCA.

1.	Sangeux M, Passmore E, Graham HK, Tirosh O. The gait standard deviation, a single measure of kinematic variability. Gait Posture. 2016;46: 194–200. doi:10.1016/j.gaitpost.2016.03.015

2.	Salarian A, Horak FB, Zampieri C, Carlson-kuhta P, Nutt JG, Aminian K. iTUG , a Sensitive and Reliable Measure of Mobility. IEEE Trans neural Syst Rehabil Eng. 2010;18: 303–310.

3.	Stuck AK, Bachmann M, Füllemann P, Josephson KR, Stuck AE. Effect of testing procedures on gait speed measurement: A systematic review. PLoS One. 2020;15. doi:10.1371/journal.pone.0234200

4.	Bahl JS, Nelson MJ, Taylor M, Solomon LB, Arnold JB, Thewlis D. Biomechanical changes and recovery of gait function after total hip arthroplasty for osteoarthritis : a systematic review and meta-analysis. Osteoarthr Cartil. 2018;26: 847–863. doi:10.1016/j.joca.2018.02.897

5.	Mellone S, Tacconi C, Chiari L. Validity of a Smartphone-based instrumented Timed Up and Go. Gait Posture. 2012;36: 163–165. doi:10.1016/j.gaitpost.2012.02.006

6.	Zakaria NA, Kuwae Y, Tamura T, Minato K, Kanaya S. Quantitative analysis of fall risk using TUG test. Comput Methods Biomech Biomed Engin. 2015;18: 426–437. doi:10.1080/10255842.2013.805211

2. PCA:

The PCA might be a good approach to identify important variables. However, insufficient clarity and scarce description make it impossible to judge the results. Especially in the presence of differently scaled variables the standardization and re-scaling is of utmost importance for reliable results. It is unclear how the variables were centered and what is meant by “reduced”. Please clarify.

It is not described if both groups were included in the PCA analysis.

The description of the PCA was modified as follow to give more details:

L 157: “A principal component analysis (PCA) was performed to keep only the most relevant biomechanical features in the analysis (excluding the time of the phases). The feature were centred and reduced by subtracting the mean and dividing by the standard deviation, to account for the difference of scales. All three groups (patients at M0, patients at M6 and control group) were included in the analysis.”

Why was the time not included? Wouldn’t that give important information which quality features are related to the time? Doing so might also spare the correlations, which further complicate the analysis.

The duration of the phases are the most commonly reported parameters in iTUG studies. We chose beforehand to keep them in the analysis to be able to compare our results to the literature. Thus, the PCA guided only the selection of biomechanical features.

The choice of variables based on the cos² values is inconsistent. For example, in quality during walking “Range of flexion contralateral hip (0.47)” and “Range obliquity thorax (0.30)” have higher cos² value than “RMS obliquity thorax (0.27)”. The selection method or the description needs clarification.

For the quality during walking, the selected feature was “Range obliquity thorax (0.30)” and not “RMS obliquity thorax (0.27)”. The bold font is a typo in the Supplementary file. The “range of flexion contralateral hip” does have a higher cos2 than the range of obliquity of the thorax but it is on the same PC as the “range of flexion of pathological hip” which is why it was excluded. In the first version of the manuscript we selected one feature per component apart for the turn-to-sit phase that regroups two movements. However, as suggested by reviewer 1, we removed the peak vertical velocity of the thorax for consistency in the method.

After checking the PCA results the feature “Distance chair to start of turn” of the turn to sit phase was added to the analysis to have a consistent selection of parameters.

3. The Kolmogorov-Smirnoff test is inappropriate to compare the patient characteristics. Age distribution can be the same in a group of children and adults. The interesting information is, whether the patient and control group are of similar age. Therefore, t-tests are needed, and the K-S tests only serves to test the assumption of normal distribution for the t-tests. For weight, a non-parametric test like the Mann-Whitney U test is necessary.

Unpaired Student’s t-tests were added to table 1 to assess the differences between groups. However, we do not understand why the test for the weight should be non-parametric. Could the reviewer develop on this point?

4. Number of tests and p-value correction:

The fact that the study design does not allow for repeated measures of variance and the downside of multiple testing is a problem. The current approach with the Bonferroni method is very conservative: if I count right 16*3 t-test were calculated for the variables plus 4 for the phases, resulting in a significance level of 0.05/52 <= 0.001, which conversely inflates the false negative (type II error) risk.

I would recommend controlling the FDR with the Benjamini-Hochberg correction or the Holm method.

The Holm method was used instead of the Bonferroni correction and all data updated. As expected, this correction included more significant differences (see Table 4):

- Difference in range of thorax obliquity during walking between M0 and M6
- Difference in duration of turn between M0 and M6
- Difference in range of thorax obliquity during turn to sit between M0 and CG

At phase level, we obtained the following modifications:

- Walking had a significantly larger deficit than the three other phases at M0
- Walking had a significantly larger deficit than the S2S and turn phases at M6

These new results strengthen the conclusion of the first version of our analysis, i.e. walking seems to present the largest deficit before and 6 months after surgery for patients undergoing THA.

To further strengthen the study effect sizes with CI could be calculated.

Effect sizes and 95% Confidence Interval were added to Table 4.

5. Overall deficit in percent:

The approach to calculate the overall deficit only on the significantly different variables within a phase is questionable. What if there was no significant variable within a phase? Has this phase gets no overall rating? What about cases where significant differences exist only pre- or post-surgery? Is the variable only included in one of the overall scores but not in the other?

Overall score should include all important variables (already identified by the PCA) and differences tested afterwards.

From our point of view, if the feature was not significantly different, there was no deficit/difference to investigate which is why we only took into account the significant features. If there was no significant variable, then there would be no percentage of deficit/difference. However, this method will increase the average deficit of the considered phase.

The point of view of the reviewer seems to be more robust from a methodological standpoint. We implemented these recommendations in the paper and modified the Statistics section of the method as follow:

L 179: “Finally, the percentages of the features selected with PCA were averaged for each phase to have one global figure per phase.”

**Further minor revisions:**

Note: These corrections are not exhaustive since it can be expected that major changes to the manuscript will follow the main revisions. Therefore, the corrections are rather examples hinting towards deficits requiring attention.

**1. Further remarks:**

• “52 aged-matched controls” if they were age-matched, there would not be a 3y difference in age.

The mention “aged-matched” was removed from the manuscript.

• “pregnancy or breast-feeding patients” relevant in this age group?

It is not relevant for the patients of this study. However, during the recruitment of patients, the range of age was between 25 and 85 years old which made this criterion relevant. For this new version of the manuscript we mentioned the age at the start of the exclusion criteria:

L 110: “Exclusion criteria included patients younger than 25 years old or older than 85 years old”

If the reviewer prefers, we can remove the mention of “pregnancy or breast-feeding patients” from the manuscript.

• “The patients and control groups did not differ in sex, […]” as pointed out earlier, the K-S test does only test distribution differences and gives no insight about mean group differences.

An unpaired Student’s t-test was added to the analysis and did not show significant differences.

• Table 4: Please revise/double-check. SD of Mean angular velocity pelvis (deg/s) (turn) and Peak angular velocity thorax (deg/s) (turn2sit) at M6 appear wrong.

The consistency of the numbers in the text, figures and tables were checked an updated when needed.

Use the same number of digits for a variable mean and SD.

The manuscript was changed accordingly.

• Table 5 is inconsistent. Sometime coloring is false (e.g. SD from Range thorax obliquity (%) during walking pre-post comparison). Also reporting of correlations is inconsistent (sometimes left out despite sig. diff and sometimes displayed without sig. diff.)

The consistency was checked and results were modified accordingly.

• “This deficit was statistically larger than the other phases (p < 0.05) but not with the Bonferonni correction.“ Reporting „significant“ results, which are insignificant after p-value correction goes against the logic of doing a correction.

The manuscripts now refers only to the Holm method.

• “[…] selected by PCA and the scores of the patients […]” implies that PCA scores were compared, which is not the case.

This was not the case indeed. The “score” here relates to the features and not to the values of the PCA. This seems to be a formulation issue, we modified the sentence:

L 260: “multiple features were selected by PCA and finally, the features of the patients were compared …”

• “[…] presence of a limp (indicated by the range of thorax obliquity).” Limping might not be very well represented by the thorax ROM. It does not reveal if the trunk lean was one-sided. Furthermore, thorax ROM might not be a good measure of limping because there are probably ways of limping that don’t involve an excessive trunk lean. Letter is a good strategy to unload the hip and is a known compensatory movement for weak hip abductors or hip pain. For limping, however, a better and more direct measure would be the single support phase.

We changed “limp” to “compensatory movements” to be more general.

• The section “Implementation in clinical settings” might be well-meant, however, has too little relation to the results.

This study was a first step toward the development of clinical tools for the measurements of function. Thus, we believe that this section can be of interest to provide a broader context and expected applications of the study to the reader. However, if the reviewer thinks this is section should be left out we can remove it from the paper.

• “The reliability, validity and responsiveness should be assessed for the time of the tasks and for the features of this study before using them in clinical practice, […]” Limitations should relate to the study.

This part was moved to the section “Implementation in clinical settings”.

• Last paragraph of Limitations: “Surprisingly, the PCA identified parameters…” is not a limitation and belongs in the discussion.

This paragraph was moved to the section “Variability of outcomes” where the PCA is discussed.

**2. Variable names:**

The variables should be named more precisely.

“Lateral distance between feet”. Is that step width? There is no lateral distance between the feet.

This parameter is the distance between the midpoint of the 2 markers of the right and left foot in the lateral direction of the lab reference frame when the patient is sitting in the chair. It was changed to “Width base of support”. A thorough description of the features was added in Supporting Information S4.

“Only 8% of patients had a range of hip flexion extension during walking […]” variable name in tables implies only hip flexion was analyzed. If the variable comprises hip flexion/extension it should be named accordingly.

This variable takes into account hip flexion and extension. It was modified throughout the manuscript.

**3. Language and typos**

Language and wording are not quite yet on a publication level and should get revised by a native speaker or expert.

Examples for awkward phrasing, wording, grammar, and typos:

• “Exclusion criteria included a previous surgery to the hip”  previous hip surgery.

The manuscript was changed accordingly.

• “hospital length of stay after surgery”  hospitalization?

This term is commonly used in medical journals, it represents the time from the day of admission to the day of discharge. See reference [12] in the manuscript: Petis et al. (2016).

- Petis SM, Howard JL, Lanting BA, Somerville LE, Vasarhelyi EM. Perioperative Predictors of Length of Stay After Total Hip Arthroplasty. J Arthroplasty. 2016;31: 1427–1430. doi:10.1016/j.arth.2016.01.005

• “interfere or be destabilised by gait analysis”  interfere with or be worsened by …

The manuscript was changed accordingly.

• “(i.e. above the mean of the control group minus one standard deviation)”  within 1SD of the control group

The manuscript was changed accordingly.

• “Evolution” is the wrong word to describe the change/difference between pre/post

We modified “evolution” by “change” throughout the manuscript.

• “pace” unusual in this context. Speed is probably more appropriate.

We changed “pace” by “speed” as suggested.

• “TUG test was not performed previously for patients with end stage”  previously in patients.

The manuscript was changed accordingly.

• “up-an-go”  up-and-go.

The manuscript was changed accordingly.

In conclusion, there is much room for improvement and besides the major issues all the little errors throughout the manuscript and tables indicate that the manuscript is not ready for publication. Nevertheless, the study might provide very useful information to the scientific community.
